# *N*‑Mesyl-Enabled Cu_2_O Catalysis: Synthesis of (*E*)‑3-Alkylideneisoindolin-1-ones and 3,4-Unsubstituted Isoquinolones via Sequential Alkynylation/Annulation

**DOI:** 10.1021/acs.joc.6c00362

**Published:** 2026-04-07

**Authors:** Ahmed R. Ali, Longqin Hu

**Affiliations:** † Department of Medicinal Chemistry, Ernest Mario School of Pharmacy, 242612 Rutgers, The State University of New Jersey, 160 Frelinghuysen Road, Piscataway, New Jersey 08854, United States; ‡ Department of Medicinal Chemistry, Faculty of Pharmacy, 158395 Mansoura University, Mansoura 35516, Egypt; § Rutgers Cancer Institute of New Jersey, New Brunswick, New Jersey 08901, United States

## Abstract

A highly regio- and stereoselective synthesis of (*E*)-3-alkylideneisoindolin-1-ones has been developed via a Cu_2_O-catalyzed ligand- and base-free Sonogashira alkynylation/5-*exo*-*dig* cyclization of *o*-iodo-*N*-mesylbenzamides with terminal alkynes. In this process, the *N*-mesyl group serves as a dual directing and activating group, ensuring the exclusive formation of the (*E*)-exocyclic double bond in good-to-excellent yields with broad functional group tolerance. The mesyl group can be subsequently removed with TBAF to afford NH-free 3-alkylideneisoindolin-1-ones. When TIPS-acetylenes were used as the terminal alkyne, the reaction stopped at the Sonogashira intermediate due to steric hindrance; however, a subsequent TBAF-mediated deprotection of both TIPS and mesyl groups triggered a 6-*endo*-*dig* cyclization. This divergent approach provides an efficient route to 3,4-unsubstituted isoquinolin-1­(2*H*)-ones. Operationally simple and scalable, this method avoids expensive additives and offers a practical, selective entry to two privileged heterocyclic scaffolds.

## Graphical abstract

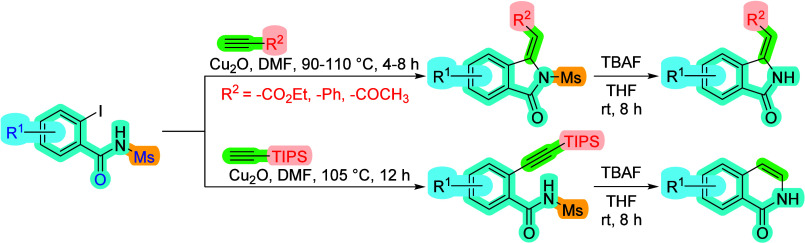

## 1 Introduction

Isoindolinones, or phthalimidines, are a vital class of nitrogen-containing heterocycles and a privileged scaffold in numerous natural products and synthetic pharmaceuticals. They exhibit a wide range of biological activities, including antimycobacterial, antifungal, and antiplatelet properties.^1^ Significant examples (Figure 1) include natural products, such as fumaridine (**I**),^2^ narceine imide (**II**),^3^ fumaramidine (**III**),^4^ fumaramine (**IV**),^5^ aristolactam alkaloids (**V**),^6^ and lennoxamine (**VI**).^7^ Within the realm of synthetic bioactive compounds, this scaffold is central to AKS 186 (**VII**),^8^ dopamine D4 antagonist (*R*)-PD 172939 (**VIII**),^9^ and lenalidomide (**IX**),^10^ a thalidomide analog used to treat multiple myeloma through inhibition of tumor necrosis factor (TNF)-α.^10^ Furthermore, the isoindolinone core is found in the anxiolytic agent (*S*)-pazinaclone (**X**).^11^ Notably, the phthalazin-1­(2*H*)-one scaffold in the FDA-approved anticancer drug olaparib (**XI**), used for the treatment of germline BRCA mutated (gBRCAm) advanced ovarian cancer,^12^ can be synthesized via the hydrazinolysis of 3-alkylideneisoindolin-1-ones.^13^

**Figure 1:**
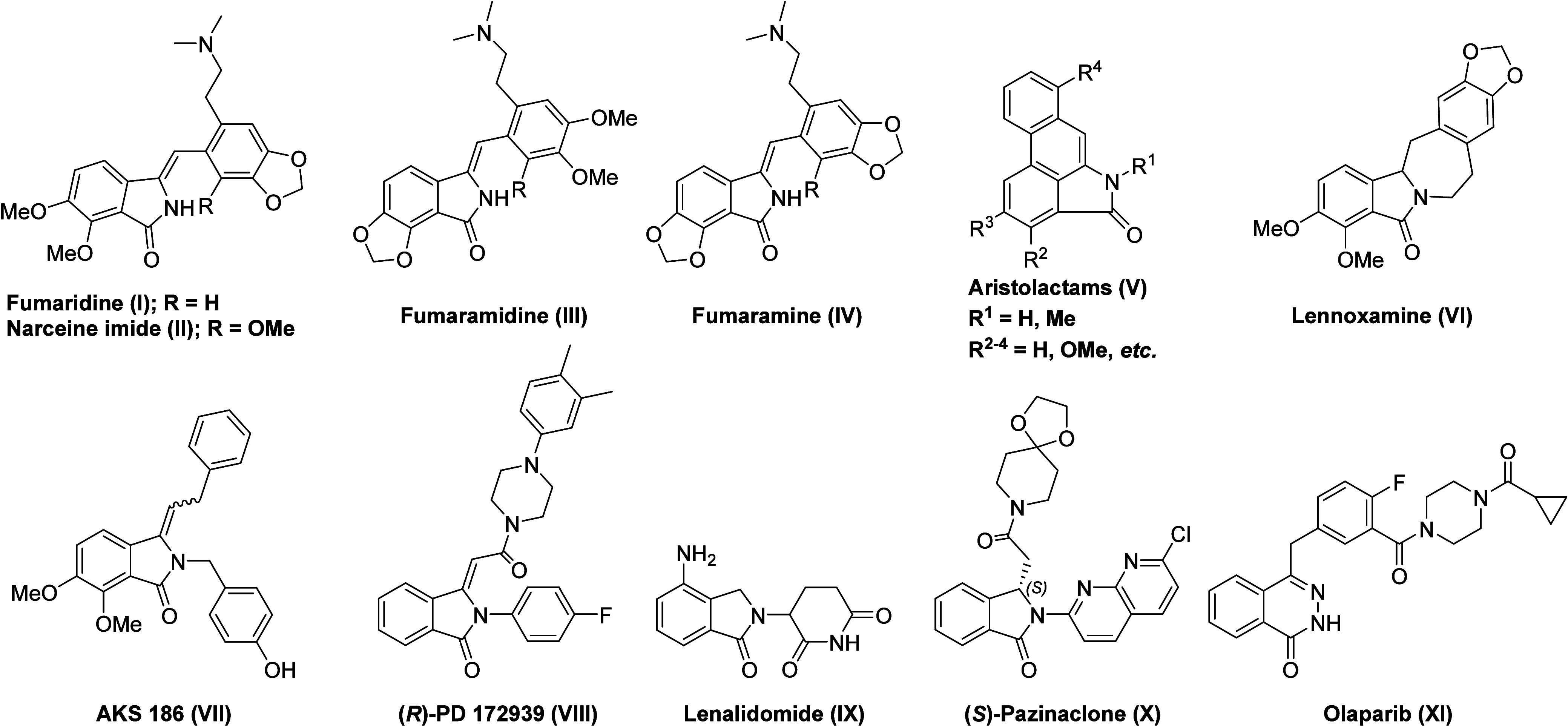
Biologically active compounds containing isoindolin-1-one and phthalazin-1­(2*H*)-one scaffolds.

Many methods have been developed as shown in Scheme 1 for the synthesis and assembly of 3-alkylideneisoindolin-1-ones, such as (a) Cu­(I) or Cu­(II)/ligand- or copper-free/palladium-catalyzed-Sonogashira coupling/5-*exo*-*dig* cycloisomerization reaction of ortho-halo-*N*-substituted benzamide with terminal alkynes under basic conditions,^14^ (b) Cu­(I) or Co­(II)-promoted decarbonylative Sonogashira coupling–cyclization strategy between ortho-halo-*N*-substituted benzamide (for Cu­(I)) or *N*-substituted benzamide (CO­(II)) and 3-arylpropiolic acid,^15^ (c) base-mediated aminocyclization of 2-(1-alkynyl)­benzamides,^16^ (d) palladium-catalyzed carboxamidation-hydroamination one-pot tandem process of (*ortho*-haloaryl)­alkynes with isocyanides as amide surrogates or amine and carbon monoxide,^17^ (e) C­(*sp* ^*2*^)–H tandem alkynylation/annulation of (un)­substituted benzamides with *N*-diverse directing groups and terminal alkynes,^13,18^ (f) C­(*sp* ^*2*^)–H olefination/annulation cascades of aryl carboxamides assisted by the *N*-tethered auxiliary group with activated alkenes (e.g., styrenes or acrylates),^19^ (g) nickel­(II)/rhodium­(III)-catalyzed tandem C­(*sp* ^*2*^)–H bond activation and annulation of arenes with *gem*-dihaloalkenes,^20^ (h) intermolecular condensation reactions of electron-deficient alkynes/arylacetate/alkylidenephosphoranes with phthalimides analogs,^8b,21^ (i) reductive coupling of phthalimides with carbonyl compounds,^22^ and (j) palladium-catalyzed Heck-Suzuki-Miyaura domino reactions of ynamide with arylboronic acids.^23^

**Scheme 1:**
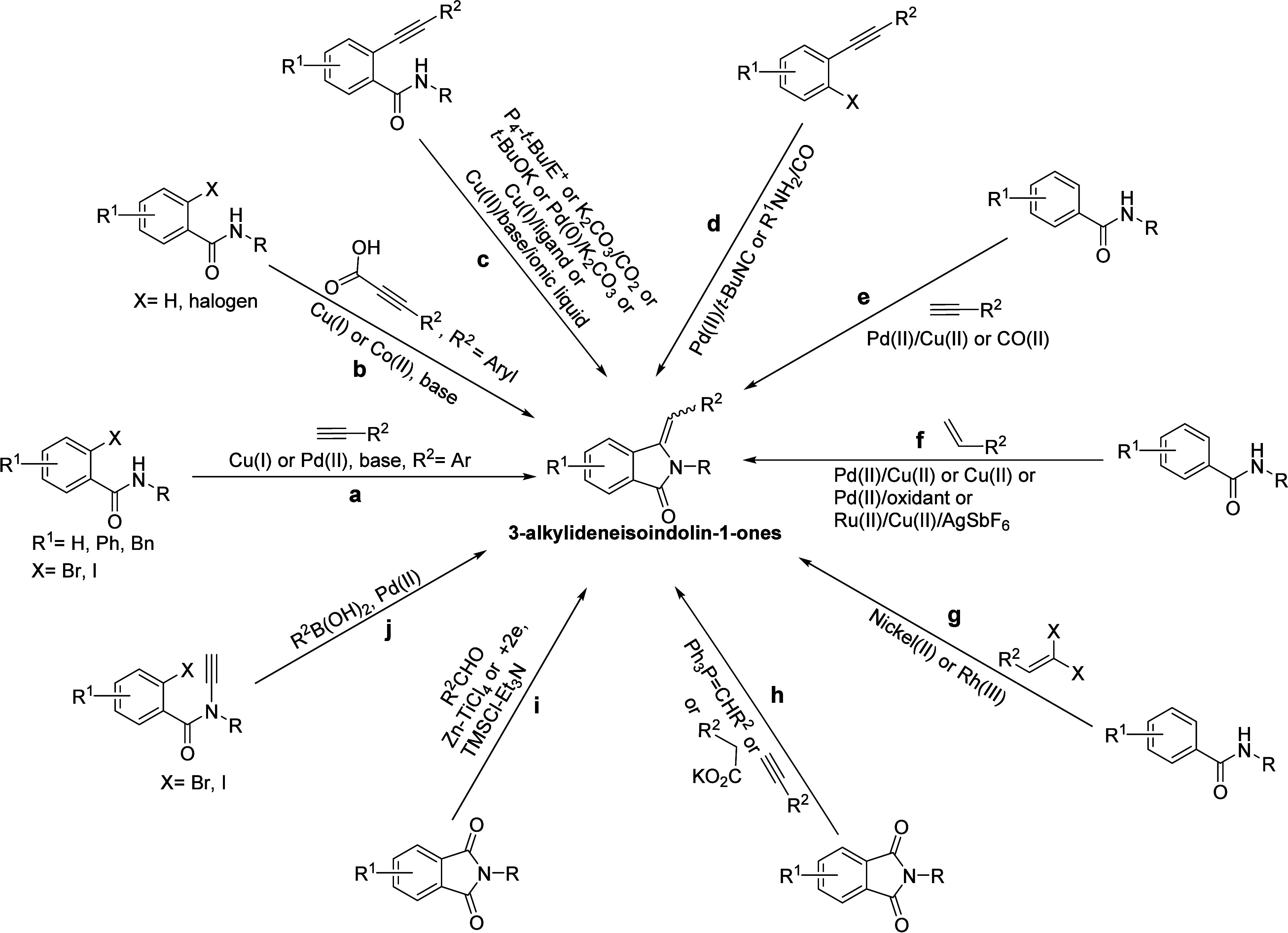
Synthetic Procedures Previously Reported for 3-Alkylideneisoindolin-1-ones

Despite these advances, many current methodologies suffer from significant limitations, such as the competitive formation of 6-*endo* cyclization byproducts and poor *E*/*Z*-stereoselectivity, which often result in diminished yields. Furthermore, many procedures require harsh acidic or basic conditions, expensive transition metal catalysts, or prefunctionalized starting materials, often with limited substrate scope, excessive oxidant requirements, and significant byproduct formation.^24^ Consequently, there is a clear need for a simple and efficient protocol that operates under mild conditions to provide high yields of these biologically relevant intermediates and products.

We were particularly intrigued by the abundant nature of the amide group. Accomplishing efficient *N*-cyclization of the *o*-iodobenzamides with terminal alkynes remains challenging due to unresolved issues regarding regioselectivity (5-*exo* vs 6-*endo* cyclization) and chemoselectivity (*O*- vs *N*-nucleophilicity of the amide moiety). Building on our ongoing program for the cost-effective synthesis of heterocyclic scaffolds,^25^ we report herein a Cu_2_O-mediated *sp* ^*2*^ C–I bond alkynylation/annulation of *N*-mesylbenzamide derivatives with terminal alkynes, utilizing the *N*-mesyl group as a new directing group to efficiently furnish 3-alkylideneisoindolin-1-ones.

## 2 Results and Discussion

### 2.1 Optimization of the Reaction Conditions

Although several methods are known for the synthesis of isoindolinones and isoquinolinones, a highly efficient route involving a domino coupling–cyclization sequence between *N*-substituted *o*-iodobenzamide and terminal alkynes is still needed. As illustrated in Scheme 2, this coupling/cyclization sequence can theoretically yield four distinct isomers through competing cyclization routes: *N*- vs *O*-cyclization and 5-*exo* vs 6-*endo* cyclization. Existing literature reports varying selectivity: palladium­(0/II) catalysts often favor the formation of functionalized iminoisobenzofurans **C**,^16d,26^ while synergistic Pd/Cu systems facilitate cyclative dimerization.^27^ In contrast, the use of CuCl_2_ with *N*-chlorosuccinimide (NCS) can lead to isobenzofuranones with loss of the nitrogen-containing moiety.^28^ Selectivity for iminoisocoumarin derivatives **D** via *O*-6-*endo*-*dig* cyclization has been achieved using FeCl_3_ with diaryl diselenides^29^ or catalytic silver salts.^30^

**Scheme 2:**
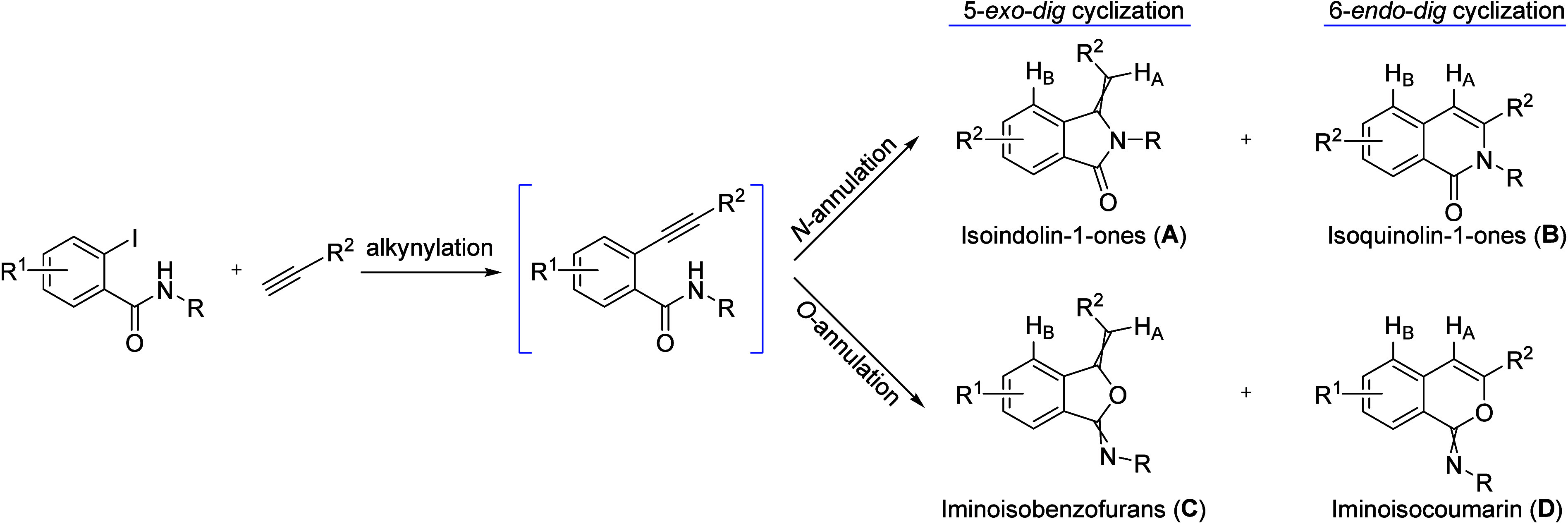
Cyclization Routes of *N*-Substituted *o*-Iodobenzamides with Terminal Alkynes

Building on our previous work with copper-catalyzed cycloisomerization, we investigated the impact of various copper catalysts on the regioselectivity of the coupling/cycloisomerization reaction.^25^ We previously demonstrated that Cu_2_O favors 5-*exo*-*dig* cyclization in aryl iodides with *ortho*-nucleophiles. Initially, we aimed to apply these ligand-free, air-stable Cu_2_O conditions to *o*-iodobenzamides (**1a**). However, screening various conditions (Table 1) revealed a highly specific set of conditions that were needed to achieve the desired coupling. Using **1a** and ethyl propiolate as a model system, we found that Cu_2_O (30 mol %) in DMF at 90 °C for 8 h resulted in minimal conversion, with a majority of the starting material remaining unreacted (entry 1). Extending the reaction time to 24 h only moderately improved conversion (entries 2 and 3). Efficiency was improved by increasing the temperature; heating to 105 °C for 8 h provided a 70% conversion (entry 4), which reached 80% after 12 h (entry 5). Notably, the reaction stopped entirely at the Sonogashira C–C coupling stage, yielding *o*-alkynylbenzamide **2a** without any observed cyclization. Switching to other copper catalysts, including Cu(0), Cu­(I), or Cu­(II) salts, resulted in no reaction, leaving the starting materials completely unchanged (entries 6–12). Transitioning from DMF to other solvents, such as methanol, *tert*-butanol, THF, dioxane, 1,2-dimethoxyethane, acetonitrile, or toluene, led to a significant decrease in conversion or total loss of reactivity (entries 13–19). DMF was established as the optimal solvent, providing the maximum conversion of 80% at 105 °C. Attempts to reduce the catalyst loading or shorten the reaction time consistently yielded inferior results.

**Table 1:**
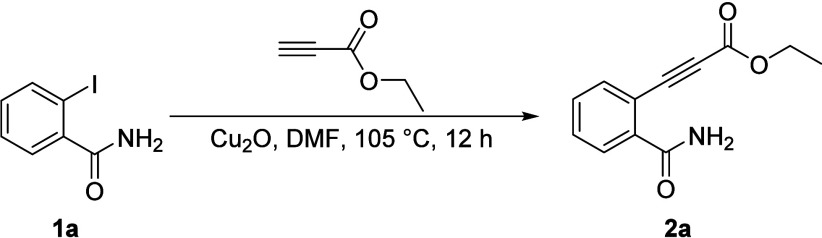
Screening of the Reaction Conditions for Sonogashira Alkynylation^a^

entry	catalyst	solvent	time (h)	temperature (°C)	%conversion^b^
1	Cu_2_O	DMF	8	90	14
2	Cu_2_O	DMF	12	90	43
3	Cu_2_O	DMF	24	90	61
4	Cu_2_O	DMF	8	105	70
5	Cu_2_O	DMF	12	105	80
6	CuCl	DMF	12	105	N.R^c^
7	CuBr	DMF	12	105	N.R.
8	CuI	DMF	12	105	N.R.
9	Cu(OAc)_2_	DMF	12	105	N.R.
10	CuSO_4_	DMF	12	105	N.R.
11	Cu(OTf)_2_	DMF	12	105	N.R.
12	Cu	DMF	12	105	N.R.
13	Cu_2_O	MeOH	12	105	N.R.
14	Cu_2_O	*t*-BuOH	12	105	23
15	Cu_2_O	THF	12	105	17
16	Cu_2_O	dioxane	12	105	30
17	Cu_2_O	DME	12	105	29
18	Cu_2_O	MeCN	12	105	23
19	Cu_2_O	PhMe	12	105	11

a Reactions were performed on a 0.1 mmol scale with the indicated catalyst (30 mol %) and solvent (0.5 mL) in sealed pressure-relief borosilicate glass vials.

b Yields of conversion were based on HPLC.

c No reaction.

This lack of cyclization products persisted across a broad library of substrates (**1a**–**e**) and various alkynes, regardless of their electronic properties (Table 2). As we had previously observed, the reaction outcome in these domino sequences is highly sensitive to the specific nature of the nucleophile and the alkyne species. In this instance, we found that Cu_2_O efficiently promoted Sonogashira C–C coupling within 12 h, generating **2a** as the major product. Crucially, no cyclized product was isolated under these optimized conditions. This result suggests an exceptionally high efficiency for the initial C–C coupling between the aryl iodide and alkyne when an *ortho*-carboxamide group is present, which may inadvertently stabilize the linear intermediate and hinder subsequent ring closure. As summarized in Table 2, the coupling of substrates **1a**–**e** exhibiting various electronic properties with ethyl propiolate (entries 1–5), phenylacetylene (entries 6 and 7), or TIPS-acetylene (entries 8 and 9) exclusively furnished *o*-alkynylbenzamide analogs **2a**–**i** in good-to-excellent yield (70–79%).

**Table 2:**
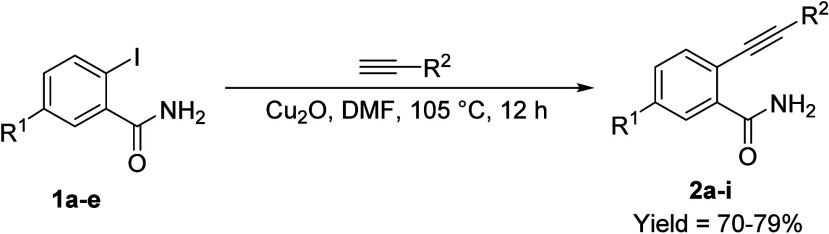
Coupling of Diverse *o*-Iodoaryl Carboxamides with Terminal Alkynes under Cu_2_O-Catalyzed Conditions^a^

entry	subs./prod.	R^1^	R^2^	yield^b^ (%)
1	**1a**/**2a**	–H	–CO_2_Et	79
2	**1b**/**2b**	–Cl		77
3	**1c**/**2c**	–Br		71
4	**1d**/**2d**	–NO_2_		79
5	**1e**/**2e**	–CH_3_		71
6	**1a**/**2f**	–H	–Ph	75
7	**1c**/**2g**	–Br		65
8	**1a**/**2h**	–H	–TIPS	74
9	**1d**/**2i**	–NO_2_		70

a Reactions were carried out in pressure-relief borosilicate glass vials.

b Isolated yields.

Inspired by our previous work on aromatic C­(*sp* ^*2*^)–X functionalization assisted by *N*-sulfonyl groups,^25^ we reasoned that the *N*-mesyl group could serve as an effective directing group to facilitate the domino Sonogashira/aza-cyclization sequence. We hypothesized that this modification would drive the reaction toward 3-alkylideneisoindolin-1-ones with high efficiency. Our investigation revealed that the weaker acidic NH protons in benzamides **1a**–**e** were insufficient for cyclization, which is consistent with literature precedents regarding the necessity of electron-withdrawing *N*-protecting groups in related C–C/C–N coupling/cyclization reactions.^14f,15b,14b^ The *N*-tethered mesyl group would act as a directing group for the domino sequence and lead to enhanced nucleophilicity of the nitrogen by increasing the acidity of the NH proton, facilitating deprotonation to form a more reactive nitrogen anion. The mesyl group was chosen specifically to enhance the NH’s acidity, thereby facilitating the C–N bond formation required for ring closure. We found that the synergy between the carbonyl and mesyl substituents was essential for the reaction to proceed, and DMF remained the optimal solvent for this *sp* ^*2*^ C–X bond alkynylation/annulation cascade.

To validate this approach, we focused on the reaction between *o*-iodo-*N*-mesylbenzamide **3a** and ethyl propiolate. The starting material, **3a**, was synthesized in one step via the EDC·HCl and DMAP-mediated coupling of commercially available *o*-iodobenzoic acid with methanesulfonamide (Scheme 3). Subjecting **3a** to our optimized conditions (30 mmol % Cu_2_O in DMF)^25^ successfully yielded 3-alkylideneisoindolin-1-one analog **4a**. This transformation achieved simultaneous C–C and C–N bond formation in good yield, confirming the *N*-mesyl group’s role as an effective promoter. Furthermore, we demonstrated that the *N*-mesyl group in **4a** could be easily removed under mild conditions using tetrabutylammonium fluoride (TBAF) in THF at room temperature to provide the deprotected product **5a**.

**Scheme 3:**
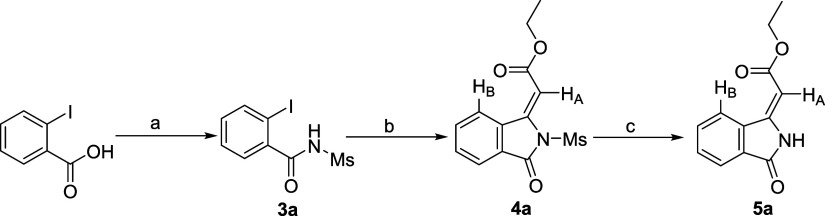
Synthesis of *o*-Iodo-*N*-mesylbenzamide (**3a**) and (*E*)-3-Alkylideneisoindolin-1-ones (**4a** and **5a**) via Cu_2_O-Catalyzed Alkynylation/Cyclization and Subsequent Mesyl Deprotection^a^

a Reagents and conditions: (a) methanesulfonamide, EDC.HCl, DMAP, DCM, rt, overnight; (b) ethyl propiolate (1 equiv), Cu_2_O (30 mmol %), DMF, 105 °C, 12 h; (c) TBAF, THF, rt, 8 h.

The formation of **5a** was confirmed on the basis of ^1^H and ^13^C NMR and mass spectrometry, with all data consistent with literature reports.^21c^ In the ^1^H NMR spectrum, the vinylic proton (H_A_) of 3-methyleneisoindolin-1-one ring appeared as a singlet at δ 6.08 (lit. 6.1 ppm^21c^). A key differentiating feature for stereochemical assignment is the chemical shift of the NH proton. The literature suggests a significant difference between the *Z*-isomer (δ 10.95) vs *E*-isomer (δ 10.34) due to the deshielding effect of the nearby ester group in the *Z*-configuration.^21c^ Our isolated compound **5a** exhibited a singlet at δ 10.33, confirming the formation of *E*-isomer via an *N*-5-*exo*-*dig* cyclization. While no synthetic precedence exists for compound **4a** to serve as a direct reference, an evaluation of the possible pathways shown in Scheme 2 confirms as discussed below that our one-pot reaction is completely regio- and stereoselective, yielding exclusively the *E*-isomer.

Our reaction conditions favored the formation of five-membered ring over six-membered ring analogs (**B** and **D**) via 6-*endo*-*dig* cyclization supported by both the chemical shift of 6.08 ppm for the olefinic proton (H_A_) in ^1^H NMR as well as HMBC (Scheme 2). In 6-*endo*-*dig* analogs, this same proton typically resonates near δ 7.0 ppm due to the deshielding effect of the benzene ring.^31^ Further confirmation was obtained by the fact that H_A_ shows only one correlation with a quaternary carbon in HMBC (Supplementary data), which is consistent with the *exo*-cyclic double bond produced by 5-*exo*-*dig* cyclization. On the other hand, a 6-*endo*-*dig* product would show three correlations, including one with the ester carbonyl carbon.

The *E*-stereochemistry was further substantiated by comparing the chemical shifts of compounds **4a** and **5a**. The vinylic proton (H_A_) resonates at δ 6.70 in **4a** and at δ 6.08 in **5a**. The downfield shift in *E*-isomer **4a** is attributed to the deshielding effect of the *N*-mesyl group. When the mesyl group was removed by TBAF, this effect was relieved, confirming that H_A_ and the *N*-mesyl group are on the same side. The *E*-stereochemistry was also corroborated by the deshielding of the benzene proton (H_B_) by the carbonyl ester, causing it to resonate downfield at δ = 8.1–8.2 ppm.

To distinguish between *N*- vs *O*-cyclization, we utilized comparison of the resulting isoindolin-1-ones (**A**) and iminoisobenzofurans (**C**) with compounds reported in the literature^21c^ and further confirmation of chemical shift changes upon *N*-mesyl removal (Scheme 2). In an *O*-5-*exo*-*dig* cyclization, the distance between the mesyl group and the olefinic proton would be too great for removal of the mesyl group to significantly impact the H_A_ chemical shift. The high stereospecificity observed appears to be driven by the steric repulsion between the *N*-mesyl group and the alkylidene group, which energetically favors the formation of the *E*-isomer.

### 2.2 Scope and Generality for the Synthesis of 3-Alkyl/Arylideneisoindolin-1-ones

To explore the scope and generality of this methodology, the reactions between various *o*-iodo-*N*-mesylbenzamides and terminal alkynes were investigated (Table 3). The domino Sonogashira/5-*exo*-*dig* cycloisomerization process proved to be completely regio- and stereoselective, exclusively yielding the *E*-isomer in all instances. The transformation proceeded smoothly regardless of the electronic nature or substitution pattern of the benzamide starting material. Both electron-withdrawing groups (e.g., nitro, **3g**) and electron-donating groups (e.g., methoxy, **3f**) provided comparable yields, suggesting that electronic effects have a negligible influence on the reaction efficiency. *Meta*- and *para*-substituted benzamides exhibited similar reactivities under our optimized catalytic conditions. Halogenated substrates (F, Cl, Br) were tolerated. Notably, no dehalogenated byproducts were detected, leaving these sites available for further synthetic transformations.

**Table 3:**
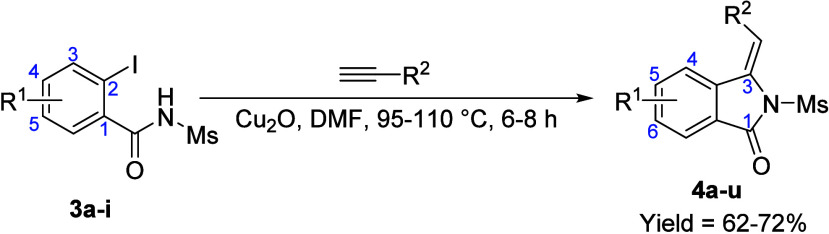
Substrate Scope for Cu_2_O-Mediated Synthesis of 3-Alkyl/Arylideneisoindolin-1-ones^a^

entry	subs./prod.	R^1^	R^2^	yield^b^ (%)
1	**3a**/**4a**	–H	–CO_2_Et	72
2	**3b**/**4b**	5/6–F		72
3	**3c**/**4c**	5/6–Cl		70
4	**3d**/**4d**	5/6–Br		68
5	**3e**/**4e**	5/6–CH_3_		70
6	**3f**/**4f**	5/6–OCH_3_		73
7	**3g**/**4g**	5/6–NO_2_		70
8	**3h**/**4h**	4/5–Cl		70
9	**3i**/**4i**	4/5–NO_2_		69
10	**3a**/**4j**	–H	–Ph	72
11	**3b**/**4k**	5/6–F		74
12	**3c**/**4l**	5/6–Cl		68
13	**3d**/**4m**	5/6–Br		62
15	**3e**/**4n**	5/6–CH_3_		73
16	**3f**/**4o**	5/6–OCH_3_		69
18	**3g**/**4p**	5/6–NO_2_		71
19	**3h**/**4q**	4/5–Cl		70
20	**3i**/**4r**	4/5–NO_2_		67
21	**3a**/**4s**	–H	–COCH_3_	70
22	**3e**/**4t**	5/6–CH_3_		69
23	**3f**/**4u**	5/6–OCH_3_		71

a Reactions were performed using different combinations of reaction temperature and time depending on the coupled alkyne, for ethyl propiolate (entries 1–9) and 3-butyn-2-one (entries 21–23): 95 °C for 6 h and for phenylacetylene (entries 10–20): 110 °C for 8 h. All the reactions were carried out in pressure-relief borosilicate glass vials.

b Isolated yields.

The scope of the terminal acetylenes was equally broad (Table 3). Successful annulation was achieved using ethyl propiolate (entries 1–9), phenylacetylene (entries 10–20), and 3-butyn-2-one (entries 21–23); notably, TIPS-acetylene proved to be an exception (discussed below). In all successful cases, the reaction proceeded with complete regioselectivity and stereoselectivity, exclusively yielding the (*E*)-configured exocyclic C=C bond. A gram-scale reaction was performed, resulting in a similarly good yield (**4a**, 74%), which highlights the practical synthetic utility of this methodology. Another significant advantage of this method is the absence of alkyne dimerization products, which are common competitive byproducts in heteroannulation reactions. The reaction is highly efficient, utilizing DMF as a solvent without the requirement of additional ligands or bases.

### 2.3 *N*-Mesyl Deprotection: Synthesis of Free (NH)-3-Alkylideneisoindolin-1-ones

Upon establishing the optimal annulation conditions, we investigated the removal of the *N*-mesyl directing group from the 3-alkylideneisoindolin-1-one analogs synthesized above. Developing a deprotection strategy that avoids harsh acidic or basic hydrolytic conditions was a primary objective as the exocyclic enamide moiety is often susceptible to decomposition under such rigorous environments.

We employed TBAF as a mild alternative to the traditional hydrolysis. TBAF facilitates clean N–S bond cleavage under neutral at room temperature,^32^ preserving the integrity of the sensitive scaffold. As shown in Table 4, this deprotection method is robust, accommodating substrates bearing both electron-withdrawing and electron-donating substituents. In addition, TBAF-mediated removal of the *N*-mesyl group proceeded smoothly without any decomposition for the central fused ring system, affording the corresponding (NH)-isoindolin-1-one derivatives **5a**–**i** in high yields, ranging from 80 to 86%.

**Table 4:**
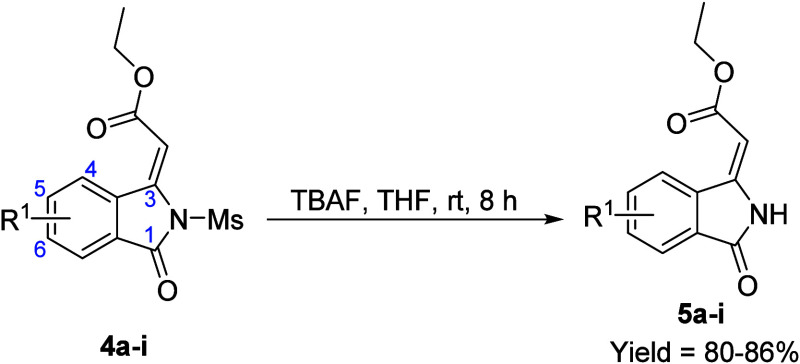
TBAF-Mediated Removal of the *N*-Mesyl Group from 3-Alkylideneisoindolin-1-ones^a^

entry	subs./prod.	R^1^	yield^b^ (%)
1	**4a**/**5a**	–H	83
2	**4b**/**5b**	6–F	85
3	**4c**/**5c**	6–Cl	82
4	**4d**/**5d**	6–Br	82
5	**4e**/**5e**	6–CH_3_	86
6	**4f**/**5f**	6–OCH_3_	85
7	**4g**/**5g**	6–NO_2_	80
8	**4h**/**5h**	5–Cl	84
9	**4i**/**5i**	5–NO_2_	85

a Reactions were carried out in pressure-relief borosilicate glass vials.

b Isolated yields.

### 2.4 Cu_2_O-Mediated Coupling of *o*-Iodo-*N*-mesylaryl Carboxamide with TIPS-Acetylene Followed by TBAF-Medicated Deprotection and Cyclization to Form 3,4-Unsubstituted Isoquinolin-1­(2*H*)-one Derivatives

Intrigued by the versatility of our C–C/C–N bond-forming conditions with diverse aliphatic and aromatic alkynes, we optimized the reaction of various *o*-iodo-*N*-mesylbenzamides with TIPS-acetylene. Based on literature precedence,^25^ we anticipated two potential pathways: the formation of the terminal C–C coupled product or a tandem C–C coupling/cyclization (5-*exo*-*dig* or *6-endo-dig*). The one-pot conditions proved to be completely regioselective for the C–C coupling intermediate, with no cyclized products isolated. This outcome could be explained by the significant steric hindrance caused by the bulky TIPS and *N*-mesyl groups, which impede C–N bond formation. Furthermore, the absence of an electron-withdrawing functional group directly tethered to the alkyne reduces its electrophilicity, thereby suppressing the nucleophilic attack by the acylsulfonamide and preventing subsequent cyclization.

We next explored the substrate scope regarding the aromatic ring of *N*-mesylbenzamide (Table 5). Both electron-rich and electron-deficient substrates were compatible, exclusively affording the noncyclized C–C coupled product **6a**–**h** in moderate-to-good yields (70–79%). With these C–C coupled compounds **6a**−**h** in hand, we investigated the feasibility of synthesizing 3-methyleneisoindolin-1-one or isoquinolinones. We envisioned that treatment of these compounds with TBAF would affect both the *N*-mesyl deprotection and removal of TIPS, leading to activation of the *ortho*-tethered alkyne. This transformation successfully yielded isoquinolin-1­(2*H*)-ones (**7a**–**g**) exclusively; notably, no 5-*exo*-*dig* products or uncyclized *o*-ethynylaryl carboxamides were isolated. This one-pot deprotection/cyclization proceeds well in THF at room temperature, where treatment with TBAF simultaneously removes the TIPS and *N*-mesyl groups and promotes 6-*endo*-*dig* hydroamidation of the resulting terminal *o*-alkynyl-*N*-mesylbenzamide without the need for an additional copper catalyst or water.

**Table 5:**
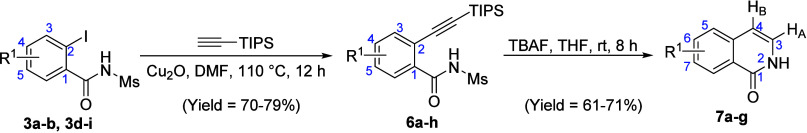
Cu_2_O-Mediated Coupling between *o*-Iodo-*N*-mesylaryl Carboxamide and TIPS-Acetylene and TBAF-Deprotection/Cyclization^a^

			step 1			step 2		
entry	subs	R^1^	prod	R^1^	yield (%)^b^	prod	R^1^	yield (%)^b^
1	**3a**	–H	**6a**	–H	79	**7a**	–H	69
2	**3b**	5–F	**6b**	5–F	78	**7b**	7–F	63
3	**3d**	5–Br	**6c**	5–Br	70	**7c**	7–Br	61
4	**3e**	5–CH_3_	**6d**	5–CH_3_	72	**7d**	7–CH_3_	62
5	**3f**	5–OCH_3_	**6e**	5–OCH_3_	73	**7e**	7–OCH_3_	65
6	**3g**	5–NO_2_	**6f**	5–NO_2_	74			
7	**3h**	4–Cl	**6g**	4–Cl	73	**7f**	6–Cl	61
8	**3i**	4–NO_2_	**6h**	4–NO_2_	71	**7g**	6–NO_2_	71

a Reactions were carried out in pressure-relief borosilicate glass vials.

b Isolated yields.

The *N*-mesyl group plays a pivotal role in this divergent strategy. While it directs the regioselective 5-*exo*-*dig* cyclization for standard alkynes, it serves as a temporary protecting group when using TIPS-acetylene, preventing premature cyclization. Upon TBAF-mediated dual deprotection (removal of both TIPS and Ms groups), the resulting free *o*-ethynylbenzamide undergoes a 6-*endo*-*dig* cyclization. This shift in regioselectivity is driven by the unmasked nucleophilic NH and the terminal alkyne under basic fluoride conditions. This dual-purpose use of the *N*-mesyl group provides an efficient, indirect route to the isoquinolin-1­(2*H*)-one scaffold that would be inaccessible via nondeprotectable precursors.

The structure of **7a** was unambiguously confirmed by ^1^H NMR and mass spectral analyses. Specifically, proton H_A_ appeared as a triplet with COSY correlations to both H_B_ and the NH proton. This methodology provides efficient access to the 3,4-unsubstituted isoquinolin-1­(2*H*)-one scaffold—a core with few reported synthetic routes.^33^ Further investigations demonstrated that a wide range of aromatic substituents were tolerated (Table 5), providing functional groups for further conversion into more complex building blocks.

### 2.5 Plausible Reaction Mechanism

Based on these results and our previously reported methodologies,^25^ we propose a reaction mechanism for the synthesis of 3-alkylideneisoindolin-1-ones as depicted in Scheme 4. We postulate a copper­(I)-catalyzed process mediated by the heterogeneous surface of the Cu_2_O catalyst, which facilitates alkyne activation, C–C coupling, and subsequent cyclization. The catalytic cycle initiates with the coordination of terminal alkynes **I** to Cu­(I) sites on the Cu_2_O catalyst surface. Deprotonation occurs *in situ* without requiring an external base, likely facilitated by the intrinsic basicity of surface-adsorbed oxygen or trace CuOH species, as previously proposed in our phthalide synthesis.^25^ While solvents like methanol, THF, and toluene are compatible, DMF was found to be superior. DMF likely enhances the stability of the surface-bound Cu-alkynyl intermediates while preventing deleterious side reactions.

**Scheme 4:**
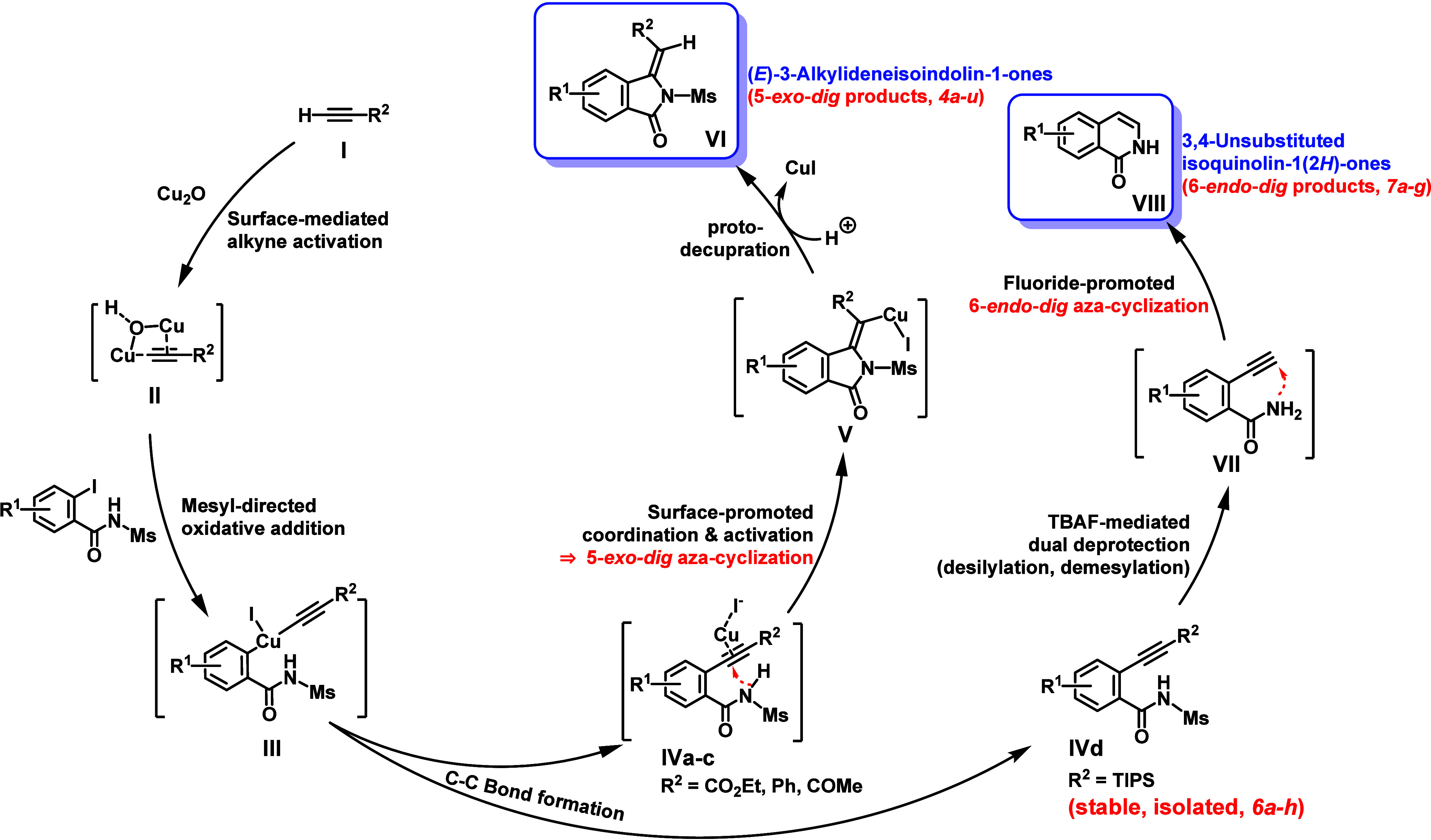
Proposed Reaction Mechanism

The resulting Cu-alkynyl species **II** undergoes oxidative addition with *o*-iodo-*N*-mesylbenzamide. We propose that the *N*-mesylamido group directs the substrate to the catalyst surface, positioning the C–I bond in the proximity of the Cu-alkynyl fragment. This surface-localized interaction stabilizes the transition state, facilitating C–C bond formation. Subsequent reductive elimination of resulting intermediate **III** affords *o*-alkynyl-*N*-mesylbenzamides **IVa**–**c** when R^2^ is CO_2_Et, Ph, or COMe. Notably, the catalytic cycle proceeds efficiently without the detection of stable intermediates or byproducts during this phase.

To initiate cyclization, *o*-alkynyl-*N*-mesylbenzamide intermediates **IVa**–**c** must be further activated. Coordination of the C≡C triple bond to Cu­(I) sites polarizes the alkyne, increasing its electrophilicity. Concurrently, the intrinsic basicity of the Cu_2_O catalyst surface promotes deprotonation of the NH group, enhancing its nucleophilicity. The subsequent 5-*exo*-*dig* aza-cyclization affords the five-membered isoindolin-1-one ring as in **V**. The exclusive regioselectivity for the 5-*exo*-*dig* pathway over the 6-*endo*-*dig* alternative is likely governed by geometric constraints imposed by the chelation of the mesylamide to the Cu_2_O surface.

The exclusive (*E*)-stereoselectivity observed in the formation of 3-alkylideneisoindolin-1-ones is proposed to arise during the 5-*exo*-*dig* cyclization. Following the Cu_2_O-catalyzed formation of the *o*-alkynyl-*N*-mesylbenzamide, the activated amide nitrogen attacks the Cu-coordinated alkyne as in **IVa**–**c**. The transition state leading to (*E*)-vinyl-copper intermediate **V** is energetically preferred, as it minimizes steric repulsion between the *N*-mesyl group and the alkyne R^2^ substituent. Subsequent protodemetalation preserves this geometry, delivering the observed (*E*)-product **VI**. This model is consistent with the absence of detectable (*Z*)-isomers across a broad range of alkyne substrates, supporting kinetic control at the cyclization stage.

Finally, the cyclized Cu-alkenyl intermediate **V** undergoes protodemetalation to release the final 3-alkylideneisoindolin-1-ones **VI** and regenerate the Cu­(I) active sites. Protons in this step may be derived from the substrate itself or residual moisture within the DMF solvent. The Cu_2_O surface acts catalytically, with active Cu­(I) sites regenerated through intrinsic redox equilibria. To maintain the integrity of the catalytic system and avoid thermal decomposition of DMF (∼150 °C), the reactions were optimized between 100 and 130 °C. This surface-mediated methodology demonstrates the dual role of Cu_2_O as both a coupling catalyst and a basic activator, ensuring high regioselectivity, stereocontrol, and yield.

In contrast, when a TIPS-protected acetylene is used as the terminal alkyne, a two-step process leads to the formation of 3,4-unsubstituted isoquinolin-1­(2*H*)-ones **VIII**. Initially, Cu_2_O-catalyzed Sonogashira coupling proceeds in an analogous manner, forming *o*-(TIPS-alkynyl)-*N*-mesylbenzamide intermediates **IVd**. However, the steric bulkiness of the TIPS group prevents coordination of the alkyne to the Cu­(I) site, thus preventing the final cyclization under these reaction conditions and arresting the reaction at the linear intermediate stage (as corroborated by entries in Table 5). Intermediates **IVd** are stable and were isolated; however, they underwent dual deprotection upon treatment with TBAF in THF at room temperature. In this process, the fluoride ion acts as a nucleophile to cleave both the Si–C (desilylation) and N–S bond (demesylation). This requirement for fluoride is supported by control reactions using *N*-unprotected *o*-iodobenzamides with TIPS-acetylene (**2h** and **2i**, Table 2), which afforded only the uncyclized Sonogashira product. The lack of isoquinolinone formation in the absence of TBAF treatment further confirms that fluoride-mediated deprotection is the requisite trigger for 6-*endo*-*dig* cyclization. While the deprotected *o*-alkynylbenzamides **VII** are expected to be stable under neutral conditions, the fluoride ion acting as a strong base in aprotic THF, induces the formation of a transient amidate anion. Without the mesyl directing group to enforce 5-*exo-dig* selectivity, intermediates **VII** undergo an efficient, base-facilitated 6-*endo*-*dig* cyclization. This results in the formation of a six-membered dihydroisoquinolinone ring, which is then released by protonation to yield the final isoquinolin-1­(2*H*)-ones **VIII**. This divergence in regioselectivity underscores the crucial role of the mesyl group in dictating the mode of cyclization, with its removal favoring the thermodynamically more stable six-membered ring.

In summary, we have developed an efficient and robust process for the synthesis of (*E*)-3-alkylideneisoindolin-1-ones via Cu_2_O-catalyzed tandem C–C coupling/5-*exo-dig* cycloisomerization. This methodology utilizes readily available *o*-iodo-*N*-mesylbenzamides and terminal alkynes to afford a diverse library of substituted isoindolin-1-ones in moderate-to-excellent yields. Furthermore, we report an efficient two-step protocol for the synthesis of 3,4-unsubstituted isoquinolin-1­(2*H*)-ones. This approach leverages an initial Cu_2_O-mediated coupling of *o*-iodo-*N*-mesylbenzamides with TIPS-acetylene, which exclusively affords the noncyclized C–C coupled intermediates. A subsequent TBAF-mediated one-pot deprotection and cyclization sequence effects the simultaneous removal of both the TIPS and *N*-mesyl groups, promoting a 6-*endo-dig* hydroamidation to yield the isoquinolinone scaffold.

Notable features of these methodologies include the use of a cost-effective, heterogeneous catalyst and the absence of external bases or ligands, significantly enhancing its practicality for large-scale synthetic applications. Given the commercial accessibility of the starting materials and the pharmacological relevance of the isoindolin-1-one and isoquinolinone scaffolds, this approach provides a streamlined and sustainable methodology for both medicinal chemistry and complex molecule synthesis.

## 3 Experimental Section

### 3.1 General Procedure for Copper-Mediated Coupling and Cyclization Reaction

To a solution of *o*-iodoaryl carboxamides or *o*-iodo-*N*-mesylaryl carboxamides (1.0 equiv) in DMF (1.5 mL for 1 mmol) were added alkyne (1 equiv) and copper­(I) oxide (0.3 equiv), and then the reaction mixture was heated at 90–110 °C for 4–12 h. The crude mixture was cooled to room temperature, quenched with 1 N HCl (20 mL), and then extracted with ethyl acetate (3 × 20 mL). The organic phase was separated, dried over anhydrous Na_2_SO_4_, and concentrated under reduced pressure. The residue was purified by flash column chromatography (ethyl acetate/hexane) to give the desired product.

## Supplementary Material



## Data Availability

The data underlying this study are available in the published article and its Supporting Information.

## Notes

Experimental details and characterization data of the new compounds and copies of ^1^H and ^13^C NMR spectra with HRMS data of all new compounds (PDF)
