# Supplementary material for: N‑Mesyl-Enabled Cu2O Catalysis: Synthesis of (E)‑3-Alkylideneisoindolin-1-ones and 3,4-Unsubstituted Isoquinolones via Sequential Alkynylation/Annulation
Source: J Org Chem. 2026 Apr 7;91(15):5402–11. doi: 10.1021/acs.joc.6c00362 (PMC13097261; doi:10.1021/acs.joc.6c00362)
Supplement: Supplementary file 1 [file jo6c00362_si_001.pdf]

Supporting Information

N-Mesyl-Enabled Cu_2O Catalysis: Synthesis of (*E*)-3-Alkylideneisoindolin-1-ones and 3,4-Unsubstituted Isoquinolones *via* Sequential Alkynylation/Annulation

Ahmed R. Ali,[a,b] and Longqin Hu[a,c,*]

[a]Department of Medicinal Chemistry, Ernest Mario School of Pharmacy, Rutgers, The State University of New Jersey, 160 Frelinghuysen Road, Piscataway, New Jersey 08854, USA.

[b]Department of Medicinal Chemistry, Faculty of Pharmacy, Mansoura University, Mansoura 35516, Egypt.

[c]Rutgers Cancer Institute of New Jersey, New Brunswick, NJ 08901, USA.

Table of Contents

Experimental Part	**S2**
NMR and HRMS Data	**S35**
References	**S157**

Experimental Part

1. General Methods

All solvents were acquired as either ACS reagent or HPLC grade and used exactly as supplied unless otherwise noted. The reagents employed in the synthetic processes were purchased as ACS grade and used straight away without additional pretreatment or purification. Reactions requiring heating were conducted in sealed pressure-relief borosilicate glass vials using a temperature-controlled dry bath block as the heat source. Analytical thin-layer chromatography (TLC) was used to track the development of the reactions using Silica G DC Kieselgel 60 F254-coated TLC plates with an aluminum backing (Merck or Sigma-Aldrich). TLC stain, such as potassium permanganate or iodine, along with heating, or ultraviolet light (UV) illumination were used to view TLC plates. The Shimadzu 2010 LC-MS system and/or the Agilent 1200 HPLC system in conjunction with an Agilent 6140 single quadrupole MS system (Santa Clara, CA) operating a multimode source were used to monitor the progress of the reaction. The LC-MS system used an Inertsil ODS-3 C18 column (3 mm × 33 mm, 3 μM) that was maintained at 40 °C. The mobile phase A was water/0.1% formic acid, and the mobile phase B was methanol/0.1% formic acid. The applied gradient program involved a flow rate of 0.8 mL/min with a linear increase in mobile phase B from 10% to 90% over 5 minutes. The UV absorbance at 280 nm was used to track the eluted peaks. Using pre-packaged RediSep normal phase silica cartridges (230-400 mesh) as the stationary phase and hexane, ethyl acetate, methanol, and dichloromethane as the mobile phases, compound purification was carried out using combiflash column chromatography on a Teledyne ISCO Companion, ^{1}H NMR spectra (400 MHz) and ^{13}C NMR spectra (100 MHz) were acquired on a Bruker 400 MHz Multinuclear NMR spectrometer (Billerica, MA). The NMR data were presented as follows: coupling constant (*J* values) in Hertz (Hz) and chemical shift in parts per million (ppm) in relation to the nondeuterated residual solvent signals. Spin multiplicities are represented by the following symbols in the NMR tabulation: brs (broad singlet), q (quartet), t (triplet), d (doublet), dd (doublet of doublets), and s (singlet). Structural assignments were made with additional information from gCOSY, gHSQC, and gHMBC experiments. Minor impurities (<5%) occasionally observed in the ^{1}H NMR spectra of certain products do not correspond to the (*Z*)-isomer. No characteristic second set of olefinic signals or separable isomeric components were detected across the series by ^{1}H NMR, 2D NMR, or chromatography. These trace signals are attributed to non-isomeric side products or minor decomposition artifacts. Intermediate **3f** was obtained with minor impurities following ISCO flash chromatography and was not further isolated in pure form. However, LC-MS analysis of the material confirmed the presence of the expected molecular ion. The partially purified intermediate was used directly in the subsequent cyclization step; final purification and full spectroscopic characterization (^{1}H NMR, ^{13}C NMR and HRMS) confirmed the identity and purity of the resulting final product.

To ascertain the precise mass values of the final compounds, HRMS analyses were carried out using the Waters ACQUITY UPLC–Synapt G2 HRMS (Milford, MA), which employs a hybrid quadrupole time-of-flight (Q-TOF) mass analyzer. An ACQUITY UPLC BEH C18 column (2.1 mm × 50 mm, 1.7 µm) was used for the UPLC separation, and the mobile phases A and B were 0.1% formic acid in water and 0.1% formic acid in methanol, respectively. The temperature of the column was kept at 40 °C. At a flow rate of 0.25 mL/min, the gradient was designed to increase from 5 to 95% B over three minutes.

General procedure for copper-mediated coupling and cyclization reaction (Method A):

To a solution of *o*-iodoaryl carboxamides or *o*-iodo-*N*-mesylaryl carboxamides (1.0 mmol, 1.0 equiv.) in DMF (1.5 mL for 1 mmol), alkyne (1.0 mmol, 1 equiv.) and copper(I) oxide (0.043 g, 0.3 mmol, 0.3 equiv.) were added and then the reaction mixture was heated at 90-110 °C for 4-12 hours. The crude mixture was cooled to room temperature, quenched with 1N HCl (20 mL), and then extracted with ethyl acetate (20 mL X 3). The organic phase was separated, dried over anhydrous Na_2SO_4, and concentrated under reduced pressure. The residue was purified by flash column chromatography (ethyl acetate/hexane) to give the desired product.

General procedure for formation of *o*-iodo-*N*-mesylaryl carboxamides (Method B):

o-Iodobenzoic acid derivatives (1.0 mmol, 1.0 equiv.) were dissolved in dichloromethane (DCM) (5.0 mL for 1 mmol) followed by cooling to 0 °C and addition of methanesulfonamide (0.095 g, 1.0 mmol, 1.0 equiv.) and 4-dimethylaminopyridine (DMAP) (0.012 g, 0.1 mmol, 0.1 equiv.). After stirring at 0 °C for 5 minutes, 1-ethyl-3-(3-dimethylaminopropyl)carbodiimide hydrochloride (EDC-HCl) (0.23 g, 1.2 mmol, 1.2 equiv.) was added and then the reaction mixture was stirred with gradual warming to room temperature and then heated at room temperature for 12 hours. After the reaction is complete, the reaction solution was diluted with DCM (20 mL) and washed with 1N HCl solution (10 mL). The organic phase was separated, dried over anhydrous Na_2SO_4, and concentrated under reduced pressure. The residue was purified by flash column chromatography (0-20% ethyl acetate/hexane) to give the product.

General procedure for removal of *N*-mesyl group using TBAF (Method C):

Compounds **4a-i**, **6a-e**, and **6g-h** (1.0 mmol, 1.0 equiv.) were dissolved in THF (3.0 mL) followed by addition of TBAF (1M in THF) (2 mL, 2.0 mmol, 2.0 equiv.). After that, the reaction mixture was stirred at room temperature for 8 hours. After the reaction was complete as indicated by TLC and LC-MS, the reaction mixture was concentrated under reduced pressure. The obtained oily residue was purified by flash column chromatography (0-20% ethyl acetate/hexane) to get the desired product.

Ethyl 3-(2-carbamoylphenyl)propiolate (2a)

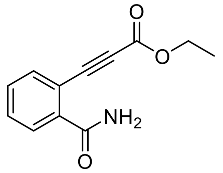

To a solution of 2-iodobenzamide (**1a**) (0.175 g, 0.71 mmol, 1.0 equiv.) in DMF (1.0 mL), ethyl propiolate (0.071 mL, 0.71 mmol, 1.0 equiv.), and copper(I) oxide (0.03 g, 0.21 mmol, 0.3 equiv.) were added and then the reaction mixture was heated at 105 °C for 12 hours. The crude mixture was cooled to room temperature, quenched with 1N HCl (20 mL), and then extracted with ethyl acetate (20 mL X 3). The organic phase was separated, dried over anhydrous Na_2SO_4, and concentrated under reduced pressure. The residue was purified by Combiflash ISCO column (ethyl acetate/hexane; %elution of desired compound= 50% ethyl acetate in hexane) to give the desired product as colorless oil in 79% yield (0.12 g); ^{1}H NMR (400 MHz, DMSO-d_6) δ 9.64 (s, 1H), 9.03 (s, 1H), 7.97 (q, J = 4.7 Hz, 1H), 7.61 – 7.53 (m, 2H), 7.46 (t, J = 4.0 Hz, 1H), 4.18 (q, J = 7.0 Hz, 2H), 1.25 (t, J = 7.1 Hz, 3H); ^{13}C NMR (100 MHz, DMSO-d_6) δ 186.2, 170.6, 164.9, 135.6, 135.2, 132.8, 131.8, 121.1, 120.8, 93.4, 58.3, 14.5; MS (ESI) m/z: $[M + H]^+$ Calcd for $C_{12}H_{12}NO_3$ 218.08; Found 218.10; HRMS (ESI-TOF) m/z: $[M + H]^+$ Calcd for $C_{12}H_{12}NO_3$ 218.0812; Found 218.0814.

Ethyl 3-(2-carbamoyl-4-chlorophenyl)propiolate (2b)

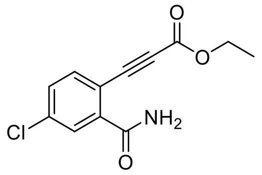

Starting materials: 5-Chloro-2-iodobenzamide (**1b**) (0.28 g, 1 mmol, 1.0 equiv.), ethyl propiolate (0.1 mL, 1 mmol, 1.0 equiv.), and copper(I) oxide (0.043 g, 0.3 mmol, 0.3 equiv.) using the same method for **2a**.
Purification conditions: Combiflash ISCO column using hexane/ethyl acetate as mobile phase (%elution of desired compound= 10% ethyl acetate in hexane).
Yield: 0.19 g (77%); colorless oil.
^{1}H NMR (400 MHz, DMSO-d_6) δ 9.67 – 9.62 (m, 1H), 9.10 (s, 1H), 8.11 (d, J = 1.8 Hz, 1H), 7.64 (dd, J = 7.8, 1.8 Hz, 1H), 7.46 (d, J = 7.8 Hz, 1H), 4.18 (q, J = 7.0 Hz, 2H), 1.25 (t, J = 7.0 Hz, 3H); ^{13}C NMR (100 MHz, DMSO-d_6) δ 185.1, 169.2, 164.7, 137.5, 136.5, 134.0, 132.4, 122.5, 121.6, 94.1, 58.5, 14.6; MS (ESI) m/z: $[M + H]^+$ Calcd for $C_{12}H_{11}ClNO_3$ 252.04; Found 252.00; HRMS (ESI-TOF) m/z: $[M + H]^+$ Calcd for $C_{12}H_{11}ClNO_3$ 252.0422; Found 252.0425.

Ethyl 3-(4-bromo-2-carbamoylphenyl)propiolate (2c)

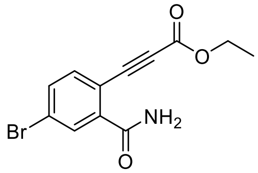

Starting materials: 5-Bromo-2-iodobenzamide (**1c**) (0.325 g, 1.0 mmol, 1.0 equiv.), ethyl propiolate (0.1 mL, 1.0 mmol, 1.0 equiv.), and copper(I) oxide (0.043 g, 0.3 mmol, 0.3 equiv.) using the same method for **2a**.

Purification conditions: Combiflash ISCO column using hexane/ethyl acetate as mobile phase (%elution of desired compound= 20% ethyl acetate in hexane).

Yield: 0.21 g (71%); colorless oil.

^{1}H NMR (400 MHz, DMSO-d_6) δ 9.62 (s, 1H), 9.06 (s, 1H), 8.25 (d, J = 1.6 Hz, 1H), 7.78 (dd, J = 7.7, 1.6 Hz, 1H), 7.39 (d, J = 7.7 Hz, 1H), 4.18 (q, J = 7.1 Hz, 2H), 1.25 (t, J = 7.1 Hz, 3H); ^{13}C NMR (100 MHz, DMSO-d_6) δ 185.0, 169.1, 164.5, 137.4, 135.2, 125.0, 124.2, 122.6, 96.3, 84.0, 58.4, 14.4; MS (ESI) m/z : [M + H]$^+$ Calcd for $C_{12}H_{11}BrNO_3$ 295.99; Found 296.00; HRMS (ESI-TOF) m/z: [M + H]$^+$ Calcd for $C_{12}H_{11}BrNO_3$ 295.9917; Found 295.9922.

Ethyl 3-(2-carbamoyl-4-nitrophenyl)propiolate (2d)

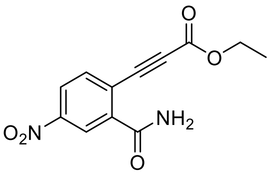

Starting materials: 2-Iodo-5-nitrobenzamide (**1d**) (0.15 g, 0.51 mmol, 1.0 equiv.), ethyl propiolate (0.051 mL, 0.51 mmol, 1.0 equiv.), and copper(I) oxide (0.022 g, 0.15 mmol, 0.3 equiv.) using the same method for **2a**.

Purification conditions: Combiflash ISCO column using hexane/ethyl acetate as mobile phase (%elution of desired compound= 40% ethyl acetate in hexane).

Yield: 0.11 g (79%); pale-yellow oil.

^{1}H NMR (400 MHz, DMSO-d_6) δ 9.97 (s, 1H), 9.23 (s, 1H), 8.95 (s, 1H), 8.45 (d, J = 7.4 Hz, 1H), 7.68 (d, J = 7.4 Hz, 1H), 4.20 (q, J = 7.0 Hz, 2H), 1.24 (q, J = 6.6 Hz, 3H); ^{13}C NMR (100 MHz, DMSO-d_6) δ 168.3, 164.2, 149.8, 136.4, 128.5, 121.5, 116.4, 95.4, 58.5, 14.3; MS (ESI) m/z: [M + H]$^+$ Calcd for $C_{12}H_{11}N_2O_5$ 263.07; Found 263.10; HRMS (ESI-TOF) m/z: [M + H]$^+$ Calcd for $C_{12}H_{11}BrNO_3$ 263.0663; Found 263.0667.

Ethyl 3-(2-carbamoyl-4-methylphenyl)propiolate (2e)

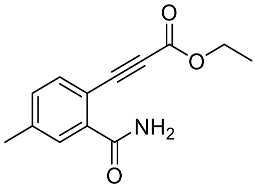

Starting materials: 2-Iodo-5-methylbenzamide (**1e**) (0.18 g, 0.69 mmol, 1.0 equiv.), ethyl propiolate (0.07 mL, 0.69 mmol, 1.0 equiv.), and copper(I) oxide (0.03 g, 0.21 mmol, 0.3 equiv.) using the same method for **2a**.

Purification conditions: Combiflash ISCO column using hexane/ethyl acetate as mobile phase (%elution of desired compound= 20% ethyl acetate in hexane).

Yield: 0.11 g (71%); pale-yellow oil.

^{1}H NMR (400 MHz, DMSO-*d$_6$*) δ 9.52 (s, 1H), 8.92 (s, 1H), 7.80 (s, 1H), 7.35 (q, *J* = 7.4 Hz, 2H), 4.15 (q, *J* = 6.8 Hz, 2H), 2.37 (s, 3H), 1.23 (t, *J* = 6.7 Hz, 3H); ^{13}C NMR (100 MHz, DMSO-*d$_6$*) δ 185.8, 170.1, 141.6, 135.2, 132.5, 121.5, 120.4, 117.5, 93.1, 85.3, 57.8, 21.0, 14.1; MS (ESI) m/z: $[M + H]^+$ Calcd for $C_{13}H_{14}NO_3$ 232.10; Found 232.10; HRMS (ESI-TOF) m/z: $[M + H]^+$ Calcd for $C_{13}H_{14}NO_3$ 232.0969; Found 232.0966.

2-(Phenylethynyl)benzamide (2f)[1]

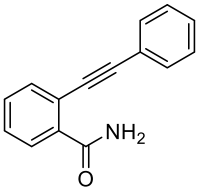

To a solution of 2-iodobenzamide (**1a**) (0.26 g, 1.05 mmol, 1.0 equiv.) in DMF (1.5 mL), phenylacetylene (0.12 mL, 1.05 mmol, 1.0 equiv.), and copper(I) oxide (0.044 g, 0.31 mmol, 0.3 equiv.) were added and then the reaction mixture was heated at 105 °C for 12 hours. The crude mixture was cooled to room temperature, quenched with 1N HCl (20 mL), and then extracted with ethyl acetate (20 mL X 3). The organic phase was separated, dried over anhydrous Na_2SO_4, and concentrated under reduced pressure. The residue was purified by Combiflash ISCO column (ethyl acetate/hexane; %elution of desired compound= 20% ethyl acetate in hexane) to give the desired product as colorless oil in 75% yield (0.16 g); ^{1}H NMR (400 MHz, DMSO-*d$_6$*) δ 7.83 (s, 1H), 7.58 (dd, *J* = 16.9, 7.1 Hz, 3H), 7.56 – 7.41 (m, 7H); ^{13}C NMR (100 MHz, DMSO-*d$_6$*) δ 169.0, 139.4, 132.4, 131.2, 129.6, 128.8, 128.7, 128.5, 127.7, 122.4, 119.7, 92.7, 88.1; MS (ESI) m/z: $[M + H]^+$ Calcd for $C_{15}H_{12}NO$ 222.09; Found 222.10; HRMS (ESI-TOF) m/z: $[M + H]^+$ Calcd for $C_{15}H_{12}NO$ 222.0914; Found 222.0914.

5-Bromo-2-(phenylethynyl)benzamide (2g)

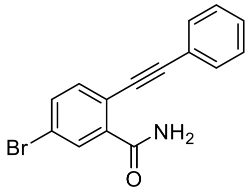

Starting materials: 5-Bromo-2-iodobenzamide (**1c**) (0.3 g, 0.92 mmol, 1.0 equiv.), phenylacetylene (0.1 mL, 0.5 mmol, 1.0 equiv.), and copper(I) oxide (0.04 g, 0.28 mmol, 0.3 equiv.) using the same method for **2f**.

Purification conditions: Combiflash ISCO column using hexane/ethyl acetate as mobile phase (%elution of desired compound= 20% ethyl acetate in hexane).

Yield: 0.179 g (65%); white solid.

^{1}H NMR (400 MHz, DMSO-d_6) δ 7.96 (s, 1H), 7.74 – 7.65 (m, 3H), 7.58 – 7.48 (m, 3H), 7.52 – 7.40 (m, 3H); ^{13}C NMR (100 MHz, DMSO-d_6) δ 167.6, 141.3, 134.3, 132.5, 131.3, 130.3, 129.1, 128.8, 122.2, 121.6, 119.1, 93.8, 87.1; MS (ESI) m/z: $[M + H]^+$ Calcd for $C_{15}H_{11}BrNO$ 300.00; Found 300.00; HRMS (ESI-TOF) m/z: $[M + H]^+$ Calcd for $C_{15}H_{11}BrNO$ 300.0019; Found 300.0015.

2-((Triisopropylsilyl)ethynyl)benzamide (2h)[2]

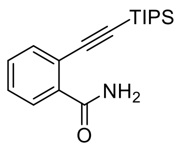

To a solution of 2-iodobenzamide (**1a**) (0.12 g, 0.49 mmol, 1.0 equiv.) in DMF (0.75 mL), (triisopropylsilyl)acetylene (0.11 mL, 0.49 mmol, 1.0 equiv.), and copper(I) oxide (0.021 g, 0.15 mmol, 0.3 equiv.) were added and then the reaction mixture was heated at 105 °C for 12 hours. The crude mixture was cooled to room temperature, quenched with 1N HCl (20 mL), and then extracted with ethyl acetate (20 mL X 3). The organic phase was separated, dried over anhydrous Na_2SO_4, and concentrated under reduced pressure. The residue was purified by Combiflash ISCO column (ethyl acetate/hexane; %elution of desired compound= 15% ethyl acetate in hexane) to give the desired product as pale-yellow oil in 74% yield (0.11 g); ^{1}H NMR (400 MHz, DMSO-d_6) δ 7.71 (s, 1H), 7.57 (s, 1H), 7.54 – 7.49 (m, 2H), 7.49 – 7.38 (m, 2H), 1.09 (s, 21H); ^{13}C NMR (100 MHz, DMSO-d_6) δ 168.7, 139.6, 133.2, 129.5, 128.7, 127.6, 119.7, 105.2, 94.3, 18.5, 10.8; MS (ESI) m/z: $[M + H]^+$ Calcd for $C_{18}H_{28}NOSi$ 302.19; Found 302.20; HRMS (ESI-TOF) m/z: $[M + H]^+$ Calcd for $C_{18}H_{28}NOSi$ 302.1935; Found 302.1933.

5-Nitro-2-((triisopropylsilyl)ethynyl)benzamide (2i)

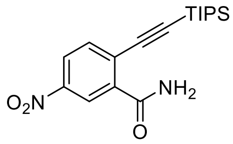

Starting materials: 2-Iodo-5-nitrobenzamide (**1d**) (0.146 g, 0.5 mmol, 1.0 equiv.), (triisopropylsilyl)acetylene (0.11 mL, 0.5 mmol, 1.0 equiv.), and copper(I) oxide (0.021 g, 0.15 mmol, 0.3 equiv.) using the same method for **2h**.

Purification conditions: Combiflash ISCO column using hexane/ethyl acetate as mobile phase (%elution of desired compound= 25% ethyl acetate in hexane).

Yield: 0.12 g (70%); pale-yellow oil.

^{1}H NMR (400 MHz, DMSO-d_6) δ 8.29 – 8.21 (m, 2H), 7.99 (s, 1H), 7.82 (s, 1H), 7.78 (d, J = 8.3 Hz, 1H), 1.10 (d, J = 3.5 Hz, 21H); ^{13}C NMR (100 MHz, DMSO-d_6) δ 166.8, 146.4, 140.9, 134.7, 126.3, 124.1, 122.3, 103.3, 100.5, 18.4, 10.7; MS (ESI) m/z: $[M + H]^+$ Calcd for $C_{18}H_{27}N_2O_3Si$ 347.18; Found 347.20; HRMS (ESI-TOF) m/z: $[M + H]^+$ Calcd for $C_{18}H_{27}N_2O_3Si$ 347.1786; Found 347.1791.

2-Iodo-*N*-(methylsulfonyl)benzamide (3a)[3]

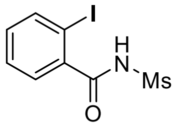

2-Iodobenzoic acid (0.744 g, 3.0 mmol, 1.0 equiv.) was dissolved in dichloromethane (DCM) (15.0 mL) followed by cooling to 0 °C and addition of methanesulfonamide (0.285 g, 3.0 mmol, 1.0 equiv.), 4-dimethylaminopyridine (DMAP) (0.037 g, 0.3 mmol, 0.1 equiv.). After stirring at 0 °C for 5 minutes, 1-ethyl-3-(3-dimethylaminopropyl)carbodiimide hydrochloride (EDC-HCl) (0.689 g, 3.6 mmol, 1.2 equiv.) was added and then the reaction mixture was stirred with gradual warming to room temperature and then heated at room temperature for 12 hours. After the reaction is complete, the reaction solution was diluted with DCM (20 mL) and washed with 1N HCl solution (10 mL). The organic phase was separated, dried over anhydrous Na_2SO_4, and concentrated under reduced pressure. The residue was purified by Combiflash ISCO column (ethyl acetate/hexane; %elution of desired compound= 20% ethyl acetate in hexane) to give the desired product as white solid in 84% yield (0.82 g); ^{1}H NMR (400 MHz, DMSO-d_6) δ 12.35 (s, 1H), 7.93 (d, J = 7.9 Hz, 1H), 7.54 – 7.44 (m, 2H), 7.25 (ddd, J = 8.5, 6.6, 2.6 Hz, 1H), 3.40 (s, 3H); ^{13}C NMR (100 MHz, DMSO-d_6) δ 168.3, 139.9, 139.3, 132.0, 128.3, 128.1, 92.9, 41.0; MS (ESI) m/z: $[M - H]^-$ Calcd for $C_8H_7INO_3S$ 323.92; Found 324.90.

5-Fluoro-2-iodo-*N*-(methylsulfonyl)benzamide (3b)

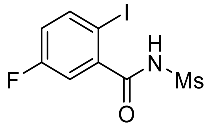

Starting materials: 5-Fluoro-2-iodobenzoic acid (0.798 g, 3.0 mmol, 1.0 equiv.), methanesulfonamide (0.285 g, 3.0 mmol, 1.0 equiv.), 4-dimethylaminopyridine (DMAP) (0.037 g, 0.3 mmol, 0.1 equiv.), and 1-ethyl-3-(3-dimethylaminopropyl)carbodiimide hydrochloride (EDC-HCl) (0.689 g, 3.6 mmol, 1.2 equiv.) using the same method for **3a**.

Purification conditions: Combiflash ISCO column using hexane/ethyl acetate as mobile phase (%elution of desired compound= 20% ethyl acetate in hexane).

Yield: 0.84 g (82%); yellowish-white solid.

^{1}H NMR (400 MHz, DMSO-d_6) δ 12.41 (s, 1H), 7.93 (dd, J = 8.8, 5.3 Hz, 1H), 7.48 (dd, J = 8.9, 3.0 Hz, 1H), 7.17 (td, J = 8.7, 3.0 Hz, 1H), 3.38 (s, 3H); ^{13}C NMR (100 MHz, DMSO-d_6) δ 167.0, 160.4, 141.7, 141.1, 119.1, 115.8, 86.9, 40.9; MS (ESI) m/z: [M - H]$^-$ Calcd for $C_8H_6FINO_3S$ 341.91; Found 341.90.

5-Chloro-2-iodo-*N*-(methylsulfonyl)benzamide (3c)

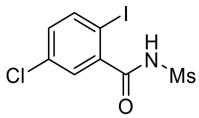

Starting materials: 5-Chloro-2-iodobenzoic acid (0.848 g, 3.0 mmol, 1.0 equiv.), methanesulfonamide (0.285 g, 3.0 mmol, 1.0 equiv.), 4-dimethylaminopyridine (DMAP) (0.037 g, 0.3 mmol, 0.1 equiv.), and 1-ethyl-3-(3-dimethylaminopropyl)carbodiimide hydrochloride (EDC-HCl) (0.689 g, 3.6 mmol, 1.2 equiv.) using the same method for **3a**.

Purification conditions: Combiflash ISCO column using hexane/ethyl acetate as mobile phase (%elution of desired compound= 20% ethyl acetate in hexane).

Yield: 0.9 g (87%); white solid.

^{1}H NMR (400 MHz, DMSO-d_6) δ 12.41 (s, 1H), 7.91 (d, J = 8.3 Hz, 1H), 7.62 (d, J = 2.6 Hz, 1H), 7.32 (dd, J = 8.4, 2.5 Hz, 1H), 3.38 (s, 3H); ^{13}C NMR (100 MHz, DMSO-d_6) δ 167.0, 141.7, 140.9, 133.1, 131.7, 128.2, 91.1, 40.9; MS (ESI) m/z: [M - H]$^-$ Calcd for $C_8H_6ClINO_3S$ 357.88; Found 357.90.

5-Bromo-2-iodo-*N*-(methylsulfonyl)benzamide (3d)

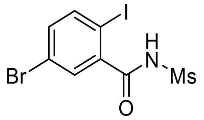

Starting materials: 5-Bromo-2-iodobenzoic acid (0.981 g, 3.0 mmol, 1.0 equiv.), methanesulfonamide (0.285 g, 3.0 mmol, 1.0 equiv.), 4-dimethylaminopyridine (DMAP) (0.037 g, 0.3 mmol, 0.1 equiv.), and 1-ethyl-3-(3-dimethylaminopropyl)carbodiimide hydrochloride (EDC-HCl) (0.689 g, 3.6 mmol, 1.2 equiv.) using the same method for **3a**.

Purification conditions: Combiflash ISCO column using hexane/ethyl acetate as mobile phase (%elution of desired compound= 15% ethyl acetate in hexane).

Yield: 1.37 g (86%); white solid.

^{1}H NMR (400 MHz, DMSO-d_6) δ 12.41 (s, 1H), 7.84 (d, J = 8.5 Hz, 1H), 7.73 (d, J = 2.4 Hz, 1H), 7.43 (dd, J = 8.4, 2.4 Hz, 1H), 3.37 (s, 3H); ^{13}C NMR (100 MHz, DMSO-d_6) δ 166.9, 142.0, 141.1, 134.5, 130.9, 121.3, 91.8, 40.9; MS (ESI) m/z: [M - H]$^-$ Calcd for $C_8H_6BrINO_3S$ 401.83; Found 401.80.

2-Iodo-5-methyl-*N*-(methylsulfonyl)benzamide (3e)

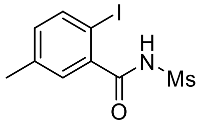

Starting materials: 2-Iodo-5-methylbenzoic acid (0.786 g, 3.0 mmol, 1.0 equiv.), methanesulfonamide (0.285 g, 3.0 mmol, 1.0 equiv.), 4-dimethylaminopyridine (DMAP) (0.037 g, 0.3 mmol, 0.1 equiv.), and 1-ethyl-3-(3-dimethylaminopropyl)carbodiimide hydrochloride (EDC-HCl) (0.689 g, 3.6 mmol, 1.2 equiv.) using the same method for **3a**.

Purification conditions: Combiflash ISCO column using hexane/ethyl acetate as mobile phase (%elution of desired compound= 20% ethyl acetate in hexane).

Yield: 0.85 g (84%); white solid.

^{1}H NMR (400 MHz, DMSO-d_6) δ 12.29 (s, 1H), 7.78 (d, J = 8.0 Hz, 1H), 7.31 (d, J = 2.1 Hz, 1H), 7.08 (dd, J = 8.1, 2.1 Hz, 1H), 3.37 (s, 3H), 2.29 (s, 3H); ^{13}C NMR (100 MHz, DMSO-d_6) δ 168.2, 139.6, 139.0, 137.8, 132.7, 129.0, 88.8, 40.9, 20.2; MS (ESI) m/z: [M - H]$^-$ Calcd for $C_9H_9INO_3S$ 337.94; Found 337.90.

2-Iodo-5-methoxy-*N*-(methylsulfonyl)benzamide (3f)

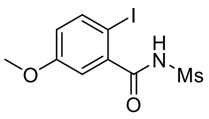

Starting materials: 2-Iodo-5-methoxybenzoic acid (0.834 g, 3.0 mmol, 1.0 equiv.), methanesulfonamide (0.285 g, 3.0 mmol, 1.0 equiv.), 4-dimethylaminopyridine (DMAP) (0.037 g, 0.3 mmol, 0.1 equiv.), and 1-

ethyl-3-(3-dimethylaminopropyl)carbodiimide hydrochloride (EDC-HCl) (0.689 g, 3.6 mmol, 1.2 equiv.) using the same method for **3a**.

Purification conditions: Combiflash ISCO column using hexane/ethyl acetate as mobile phase (%elution of desired compound= 20% ethyl acetate in hexane).

Yield: 0.88 g (83%); white solid.

MS (ESI) m/z: [M - H]$^-$ Calcd for $C_9H_9INO_4S$ 353.93; Found 353.90.

2-Iodo-*N*-(methylsulfonyl)-5-nitrobenzamide (3g)

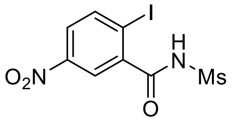

Starting materials: 2-Iodo-5-nitrobenzoic acid (0.879 g, 3.0 mmol, 1.0 equiv.), methanesulfonamide (0.285 g, 3.0 mmol, 1.0 equiv.), 4-dimethylaminopyridine (DMAP) (0.037 g, 0.3 mmol, 0.1 equiv.), and 1-ethyl-3-(3-dimethylaminopropyl)carbodiimide hydrochloride (EDC-HCl) (0.689 g, 3.6 mmol, 1.2 equiv.) using the same method for **3a**.

Purification conditions: Combiflash ISCO column using hexane/ethyl acetate as mobile phase (%elution of desired compound= 20% ethyl acetate in hexane).

Yield: 0.89 g (81%); yellowish-white solid.

^{1}H NMR (400 MHz, DMSO-d_6) δ 12.56 (s, 1H), 8.35 (d, J = 2.6 Hz, 1H), 8.23 (d, J = 8.6 Hz, 1H), 8.03 (dd, J = 8.7, 2.7 Hz, 1H), 3.42 (s, 3H); ^{13}C NMR (100 MHz, DMSO-d_6) δ 166.5, 147.1, 141.1, 141.0, 125.7, 122.8, 102.9, 41.1; MS (ESI) m/z: [M - H]$^-$ Calcd for $C_8H_6IN_2O_5S$ 368.90; Found 368.90.

4-Chloro-2-iodo-*N*-(methylsulfonyl)benzamide (3h)

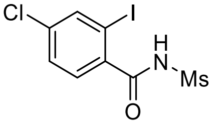

Starting materials: 4-Chloro-2-iodobenzoic acid (0.848 g, 3.0 mmol, 1.0 equiv.), methanesulfonamide (0.285 g, 3.0 mmol, 1.0 equiv.), 4-dimethylaminopyridine (DMAP) (0.037 g, 0.3 mmol, 0.1 equiv.), and 1-ethyl-3-(3-dimethylaminopropyl)carbodiimide hydrochloride (EDC-HCl) (0.689 g, 3.6 mmol, 1.2 equiv.) using the same method for **3a**.

Purification conditions: Combiflash ISCO column using hexane/ethyl acetate as mobile phase (%elution of desired compound= 20% ethyl acetate in hexane).

Yield: 0.86 g (80%); yellowish-white solid.

^{1}H NMR (400 MHz, DMSO-d_6) δ 12.39 (s, 1H), 8.02 (d, J = 2.0 Hz, 1H), 7.58 (dd, J = 8.2, 2.0 Hz, 1H), 7.50 (d, J = 8.2 Hz, 1H), 3.39 (s, 3H); ^{13}C NMR (100 MHz, DMSO-d_6) δ 167.5, 138.8, 138.2, 135.5, 129.6, 128.1, 94.2, 41.0; MS (ESI) m/z: [M - H]$^-$ Calcd for $C_8H_6ClINO_3S$ 357.88; Found 357.90.

2-Iodo-*N*-(methylsulfonyl)-4-nitrobenzamide (3i)

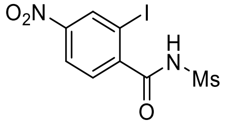

Starting materials: 2-Iodo-4-nitrobenzoic acid (0.879 g, 3.0 mmol, 1.0 equiv.), methanesulfonamide (0.285 g, 3.0 mmol, 1.0 equiv.), 4-dimethylaminopyridine (DMAP) (0.037 g, 0.3 mmol, 0.1 equiv.), and 1-ethyl-3-(3-dimethylaminopropyl)carbodiimide hydrochloride (EDC-HCl) (0.689 g, 3.6 mmol, 1.2 equiv.) using the same method for **3a**.

Purification conditions: Combiflash ISCO column using hexane/ethyl acetate as mobile phase (%elution of desired compound= 20% ethyl acetate in hexane).

Yield: 0.92 g (83%); yellowish-white solid.

^{1}H NMR (400 MHz, DMSO-*d₆*) δ 12.60 (s, 1H), 8.63 (d, *J* = 2.2 Hz, 1H), 8.30 (dd, *J* = 8.5, 2.2 Hz, 1H), 7.74 (d, *J* = 8.4 Hz, 1H), 3.41 (s, 3H); ^{13}C NMR (100 MHz, DMSO-d_6) δ 167.4, 148.2, 146.0, 133.4, 129.1, 123.1, 93.5, 41.1; MS (ESI) m/z: [M - H]$^-$ Calcd for $C_8H_6IN_2O_5S$ 368.90; Found 368.90.

Ethyl (*E*)-2-(2-(methylsulfonyl)-3-oxoisoindolin-1-ylidene)acetate (4a)

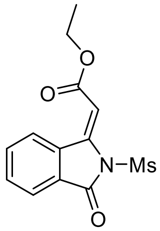

To a solution of 2-Iodo-*N*-(methylsulfonyl)benzamide (**3a**) (0.162 g, 0.5 mmol, 1.0 equiv.) in DMF (0.75 mL), ethyl propiolate (0.1 mL, 0.5 mmol, 1.0 equiv.), and copper(I) oxide (0.021 g, 0.15 mmol, 0.3 equiv.) were added and then the reaction mixture was heated at 95 °C for 6 hours. The crude mixture was cooled to room temperature, quenched with 1N HCl (20 mL), and then extracted with ethyl acetate (20 mL X 3). The organic phase was separated, dried over anhydrous Na_2SO_4, and concentrated under reduced pressure. The residue was purified by Combiflash ISCO column (ethyl acetate/hexane; %elution of desired compound= 20% ethyl acetate in hexane) to give the desired product as pale-yellow oil in 72% yield (0.107 g); ^{1}H NMR (400 MHz, DMSO-d_6) δ 8.18 (d, *J* = 7.9 Hz, 1H), 7.91 (d, *J* = 7.7 Hz, 1H), 7.86 (t, *J* = 7.6 Hz, 1H), 7.71 (t, *J* = 7.5 Hz, 1H), 6.70 (s, 1H), 4.20 (q, *J* = 7.1 Hz, 2H), 3.63 (s, 3H), 1.27 (t, *J* = 7.1 Hz, 3H); ^{13}C NMR (100 MHz, DMSO-d_6) δ 166.2, 165.2, 138.3, 136.5, 135.3, 131.7, 125.8, 124.4, 121.6, 103.6, 60.6, 42.1, 13.9; MS (ESI) m/z: [M + H]$^+$ Calcd for $C_{13}H_{14}NO_5S$ 296.06; Found 296.10; HRMS (ESI-TOF) m/z: [M + H]$^+$ Calcd for $C_{13}H_{14}NO_5S$ 296.0588; Found 296.0595.

Ethyl (*E*)-2-(5-fluoro-2-(methylsulfonyl)-3-oxoisoindolin-1-ylidene)acetate (4b)

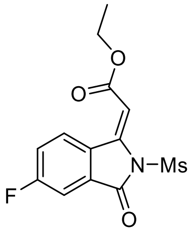

Starting materials: 5-Fluoro-2-iodo-*N*-(methylsulfonyl)benzamide (**3b**) (0.24 g, 0.7 mmol, 1.0 equiv.), ethyl propiolate (0.07 mL, 0.7 mmol, 1.0 equiv.), and copper(I) oxide (0.03 g, 0.21 mmol, 0.3 equiv.) using the same method for **4a**.

Purification conditions: Combiflash ISCO column using hexane/ethyl acetate as mobile phase (%elution of desired compound= 15% ethyl acetate in hexane).

Yield: 0.157 g (72%); pale-yellow oil.

^{1}H NMR (400 MHz, DMSO-d_6) δ 8.25 (dd, J = 8.6, 4.4 Hz, 1H), 7.81 – 7.70 (m, 2H), 6.71 (s, 1H), 4.19 (q, J = 7.1 Hz, 2H), 3.63 (s, 3H), 1.27 (t, J = 7.1 Hz, 3H); ^{13}C NMR (100 MHz, DMSO-d_6) δ 165.2, 165.1, 162.6, 135.8, 134.6, 128.0, 124.2, 123.0, 111.1, 103.7, 60.6, 42.1, 13.9; MS (ESI) m/z: $[M + H]^+$ Calcd for $C_{13}H_{13}FNO_5S$ 314.05; Found 314.00; HRMS (ESI-TOF) m/z: $[M + H]^+$ Calcd for $C_{13}H_{13}FNO_5S$ 314.0493; Found 314.0493.

Ethyl (*E*)-2-(5-chloro-2-(methylsulfonyl)-3-oxoisoindolin-1-ylidene)acetate (4c)

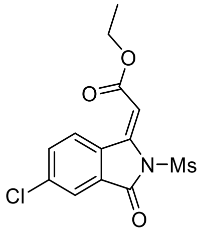

Starting materials: 5-Chloro-2-iodo-*N*-(methylsulfonyl)benzamide (**3c**) (0.18 g, 0.5 mmol, 1.0 equiv.), ethyl propiolate (0.5 mL, 0.5 mmol, 1.0 equiv.), and copper(I) oxide (0.021 g, 0.15 mmol, 0.3 equiv.) using the same method for **4a**.

Purification conditions: Combiflash ISCO column using hexane/ethyl acetate as mobile phase (%elution of desired compound= 15% ethyl acetate in hexane).

Yield: 0.12 g (70%); white solid.

^{1}H NMR (400 MHz, DMSO-d_6) δ 8.19 (d, J = 8.3 Hz, 1H), 7.95 (d, J = 2.0 Hz, 1H), 7.90 (dd, J = 8.3, 2.0 Hz, 1H), 6.73 (s, 1H), 4.19 (q, J = 7.1 Hz, 2H), 3.63 (s, 3H), 1.27 (t, J = 7.1 Hz, 3H); ^{13}C NMR (100 MHz, DMSO-d_6) δ 165.1, 164.9, 136.9, 136.3, 135.7, 135.2, 127.6, 124.0, 123.5, 104.3, 60.6, 42.0, 13.9; MS (ESI) m/z: $[M + H]^+$ Calcd for $C_{13}H_{13}ClNO_5S$ 330.02; Found 330.00; HRMS (ESI-TOF) m/z: $[M + H]^+$ Calcd for $C_{13}H_{13}ClNO_5S$ 330.0198; Found 330.0200.

Ethyl (*E*)-2-(5-bromo-2-(methylsulfonyl)-3-oxoisoindolin-1-ylidene)acetate (4d)

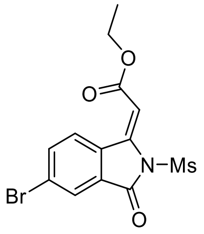

Starting materials: 5-Bromo-2-iodo-*N*-(methylsulfonyl)benzamide (**3d**) (0.202 g, 0.5 mmol, 1.0 equiv.), ethyl propiolate (0.05 mL, 0.5 mmol, 1.0 equiv.), and copper(I) oxide (0.021 g, 0.15 mmol, 0.3 equiv.) using the same method for **4a**.

Purification conditions: Combiflash ISCO column using hexane/ethyl acetate as mobile phase (%elution of desired compound= 10% ethyl acetate in hexane).

Yield: 0.126 g (68%); pale-yellow oil.

^{1}H NMR (400 MHz, DMSO-d_6) δ 8.16 – 8.00 (m, 3H), 6.75 (s, 1H), 4.19 (q, J = 7.1 Hz, 2H), 3.63 (s, 3H), 1.26 (t, J = 7.1 Hz, 3H); ^{13}C NMR (100 MHz, DMSO-d_6) δ 165.1, 164.9, 137.9, 137.3, 135.8, 127.8, 126.9, 124.7, 123.6, 104.3, 60.6, 42.0, 13.9; MS (ESI) m/z: Calcd for $C_{13}H_{13}BrNO_5S$ 373.97; Found 374.00; HRMS (ESI-TOF) m/z: $[M + H]^+$ Calcd for $C_{13}H_{13}BrNO_5S$ 373.9693; Found 373.9691.

Ethyl (*E*)-2-(5-methyl-2-(methylsulfonyl)-3-oxoisoindolin-1-ylidene)acetate (4e)

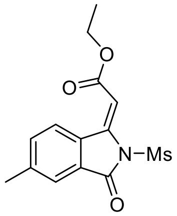

Starting materials: 2-Iodo-5-methyl-*N*-(methylsulfonyl)benzamide (**3e**) (0.17 g, 0.5 mmol, 1.0 equiv.), ethyl propiolate (0.05 mL, 0.5 mmol, 1.0 equiv.), and copper(I) oxide (0.021 g, 0.15 mmol, 0.3 equiv.) using the same method for **4a**.

Purification conditions: Combiflash ISCO column using hexane/ethyl acetate as mobile phase (%elution of desired compound= 20% ethyl acetate in hexane).

Yield: 0.108 g (70%); pale-yellow oil.

^{1}H NMR (400 MHz, DMSO-d_6) δ 8.03 (d, J = 8.0 Hz, 1H), 7.70 (s, 1H), 7.65 (d, J = 8.0 Hz, 1H), 6.60 (s, 1H), 4.19 (q, J = 7.1 Hz, 2H), 3.62 (s, 3H), 2.44 (s, 3H), 1.26 (t, J = 7.1 Hz, 3H); ^{13}C NMR (100 MHz, DMSO-d_6) δ 166.3, 165.3, 142.2, 136.7, 136.3, 135.9, 126.0, 124.2, 121.4, 102.8, 60.5, 42.1, 20.9, 13.9; MS (ESI) m/z: $[M + H]^+$ Calcd for $C_{14}H_{16}NO_5S$ 310.07; Found 310.10; HRMS (ESI-TOF) m/z: $[M + H]^+$ Calcd for $C_{14}H_{16}NO_5S$ 310.0744; Found 310.0752.

Ethyl (*E*)-2-(5-methoxy-2-(methylsulfonyl)-3-oxoisoindolin-1-ylidene)acetate (4f)

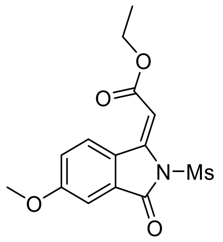

Starting materials: 2-Iodo-5-methoxy-*N*-(methylsulfonyl)benzamide (**3f**) (0.225 g, 0.63 mmol, 1.0 equiv.), ethyl propiolate (0.063 mL, 0.63 mmol, 1.0 equiv.), and copper(I) oxide (0.027 g, 0.19 mmol, 0.3 equiv.) using the same method for **4a**.

Purification conditions: Combiflash ISCO column using hexane/ethyl acetate as mobile phase (%elution of desired compound= 60% ethyl acetate in hexane).

Yield: 0.149 g (73%); white solid.

^{1}H NMR (400 MHz, DMSO-d_6) δ 8.09 (d, J = 8.6 Hz, 1H), 7.47 – 7.35 (m, 2H), 6.57 (s, 1H), 4.18 (q, J = 7.2 Hz, 2H), 3.91 (s, 3H), 3.62 (s, 3H), 1.26 (t, J = 7.3 Hz, 3H); ^{13}C NMR (100 MHz, DMSO-d_6) δ 166.2, 165.4, 162.2, 136.8, 131.0, 127.6, 123.5, 123.2, 106.9, 102.1, 60.4, 56.1, 42.1, 14.0; MS (ESI) m/z: [M + H]$^+$ Calcd for $C_{14}H_{16}NO_6S$ 326.07; Found 326.10; HRMS (ESI-TOF) m/z: [M + H]$^+$ Calcd for $C_{14}H_{16}NO_6S$ 326.0693; Found 326.0697.

Ethyl (*E*)-2-(2-(methylsulfonyl)-5-nitro-3-oxoisoindolin-1-ylidene)acetate (4g)

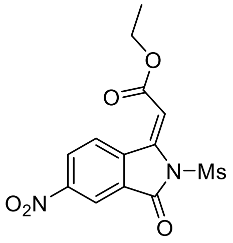

Starting materials: 2-Iodo-*N*-(methylsulfonyl)-5-nitrobenzamide (**3g**) (0.125 g, 0.34 mmol, 1.0 equiv.), ethyl propiolate (0.034 mL, 0.34 mmol, 1.0 equiv.), and copper(I) oxide (0.014 g, 0.1 mmol, 0.3 equiv.) using the same method for **4a**.

Purification conditions: Combiflash ISCO column using hexane/ethyl acetate as mobile phase (%elution of desired compound= 30% ethyl acetate in hexane).

Yield: 0.081 g (70%); colorless oil.

^{1}H NMR (400 MHz, DMSO-d_6) δ 8.65 (dd, J = 8.6, 2.1 Hz, 1H), 8.55 (d, J = 2.1 Hz, 1H), 8.44 (d, J = 8.6 Hz, 1H), 6.96 (s, 1H), 4.22 (q, J = 7.1 Hz, 2H), 3.67 (d, J = 9.0 Hz, 3H), 1.28 (t, J = 7.1 Hz, 3H); ^{13}C NMR (100 MHz, DMSO-d_6) δ 164.9, 164.4, 149.4, 143.0, 134.9, 129.7, 127.1, 123.3, 119.6, 106.9, 60.9, 42.0, 13.9; MS (ESI) m/z: [M + H]$^+$ Calcd for $C_{13}H_{13}N_2O_7S$ 341.04; Found 341.05; HRMS (ESI-TOF) m/z: [M + H]$^+$ Calcd for $C_{13}H_{13}N_2O_7S$ 341.0438; Found 341.0441.

Ethyl (*E*)-2-(6-chloro-2-(methylsulfonyl)-3-oxoisoindolin-1-ylidene)acetate (4h)

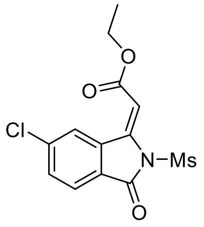

Starting materials: 4-Chloro-2-iodo-*N*-(methylsulfonyl)benzamide (**3h**) (0.18 g, 0.5 mmol, 1.0 equiv.), ethyl propiolate (0.05 mL, 0.5 mmol, 1.0 equiv.), and copper(I) oxide (0.021 g, 0.15 mmol, 0.3 equiv.) using the same method for **4a**.

Purification conditions: Combiflash ISCO column using hexane/ethyl acetate as mobile phase (%elution of desired compound= 20% ethyl acetate in hexane).

Yield: 0.11 g (70%); pale-yellow oil.

^{1}H NMR (400 MHz, DMSO-d_6) δ 8.40 (d, J = 1.7 Hz, 1H), 7.93 (d, J = 8.2 Hz, 1H), 7.76 (dd, J = 8.2, 1.7 Hz, 1H), 6.80 (s, 1H), 4.20 (q, J = 7.1 Hz, 2H), 3.62 (s, 3H), 1.26 (t, J = 7.1 Hz, 3H); ^{13}C NMR (100 MHz, DMSO-d_6) δ 165.3, 165.1, 140.3, 140.1, 135.3, 131.8, 126.2, 124.6, 121.9, 104.9, 60.7, 42.0, 13.9; MS (ESI) m/z: $[M + H]^+$ Calcd for $C_{13}H_{13}ClNO_5S$ 330.02; Found 330.00; HRMS (ESI-TOF) m/z: $[M + H]^+$ Calcd for $C_{13}H_{13}ClNO_5S$ 330.0198; Found 330.0198.

Ethyl (*E*)-2-(2-(methylsulfonyl)-6-nitro-3-oxoisoindolin-1-ylidene)acetate (4i)

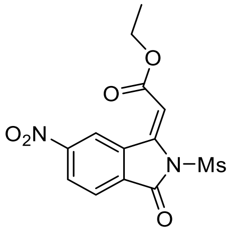

Starting materials: 2-Iodo-*N*-(methylsulfonyl)-4-nitrobenzamide (**3i**) (0.185 g, 0.5 mmol, 1.0 equiv.), ethyl propiolate (0.05 mL, 0.5 mmol, 1.0 equiv.), and copper(I) oxide (0.021 g, 0.15 mmol, 0.3 equiv.) using the same method for **4a**.

Purification conditions: Combiflash ISCO column using hexane/ethyl acetate as mobile phase (%elution of desired compound= 20% ethyl acetate in hexane).

Yield: 0.12 g (69%); pale-yellow oil.

^{1}H NMR (400 MHz, DMSO-d_6) δ 9.11 (d, J = 1.9 Hz, 1H), 8.44 (dd, J = 8.4, 1.9 Hz, 1H), 8.13 (d, J = 8.4 Hz, 1H), 7.06 (s, 1H), 4.21 (q, J = 7.1 Hz, 2H), 3.65 (s, 3H), 1.28 (t, J = 7.1 Hz, 3H); ^{13}C NMR (100 MHz, DMSO-d_6) δ 165.0, 164.6, 151.9, 139.4, 134.8, 130.4, 126.2, 126.0, 117.5, 105.9, 60.8, 42.0, 13.9; MS (ESI) m/z: $[M + H]^+$ Calcd for $C_{13}H_{13}N_2O_7S$ 341.04; Found 341.05; HRMS (ESI-TOF) m/z: $[M + H]^+$ Calcd for $C_{13}H_{13}N_2O_7S$ 341.0438; Found 341.0441.

(*E*)-3-Benzylidene-2-(methylsulfonyl)isoindolin-1-one (4j)[4]

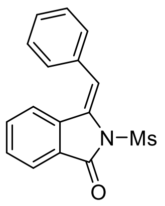

To a solution of 2-Iodo-*N*-(methylsulfonyl)benzamide (**3a**) (0.162 g, 0.5 mmol, 1.0 equiv.) in DMF (0.75 mL), phenylacetylene (0.055 mL, 0.5 mmol, 1.0 equiv.), and copper(I) oxide (0.021 g, 0.15 mmol, 0.3 equiv.) were added and then the reaction mixture was heated at 110 °C for 8 hours. The crude mixture was cooled to room temperature, quenched with 1N HCl (20 mL), and then extracted with ethyl acetate (20 mL X 3). The organic phase was separated, dried over anhydrous Na_2SO_4, and concentrated under reduced pressure. The residue was purified by Combiflash ISCO column (ethyl acetate/hexane; %elution of desired compound= 25% ethyl acetate in hexane) to give the desired product as pale-yellow oil in 72% yield (0.17 g); ^{1}H NMR (400 MHz, DMSO-d_6) δ 8.23 (d, J = 8.1 Hz, 1H), 8.10 – 8.02 (m, 2H), 7.97 – 7.87 (m, 1H), 7.75 (d, J = 5.4 Hz, 2H), 7.68 – 7.62 (m, 1H), 7.60 – 7.43 (m, 3H), 3.25 (s, 3H); ^{13}C NMR (100 MHz, DMSO-d_6) δ 158.6, 151.7, 135.8, 135.5, 130.6, 130.4, 129.3, 129.0, 128.4, 128.3, 127.7, 126.8, 124.9, 119.6, 103.3, 42.2; MS (ESI) m/z: $[M + H]^+$ Calcd for $C_{16}H_{14}NO_3S$ 300.07; Found 300.1; HRMS (ESI-TOF) m/z: $[M + H]^+$ Calcd for $C_{16}H_{14}NO_3S$ 300.0689; Found 300.0691.

(*E*)-3-Benzylidene-6-fluoro-2-(methylsulfonyl)isoindolin-1-one (4k)

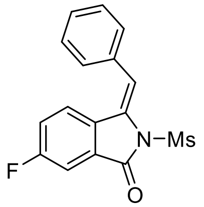

Starting materials: 5-Fluoro-2-iodo-*N*-(methylsulfonyl)benzamide (**3b**) (0.1 g, 0.44 mmol, 1.0 equiv.), phenylacetylene (0.048 g, 0.44 mmol, 1.0 equiv.), and copper(I) oxide (0.019 g, 0.13 mmol, 0.3 equiv.) using the same method for **4j**.

Purification conditions: Combiflash ISCO column using hexane/ethyl acetate as mobile phase (%elution of desired compound= 30% ethyl acetate in hexane).

Yield: 0.103 g (74%); colorless oil.

^{1}H NMR (400 MHz, DMSO-d_6) δ 8.06 – 8.00 (m, 2H), 7.94 – 7.87 (m, 1H), 7.83 (dd, J = 7.3, 2.1 Hz, 2H), 7.78 (s, 1H), 7.61 – 7.46 (m, 3H), 3.25 (s, 3H); ^{13}C NMR (100 MHz, DMSO-d_6) δ 162.8, 160.3, 157.8, 151.4, 132.5, 130.4, 129.8, 129.1, 124.9, 124.2, 121.4, 112.7, 102.7, 42.2; MS (ESI) m/z: $[M + H]^+$ Calcd for $C_{16}H_{13}FNO_3S$ 318.06; Found 318.10; HRMS (ESI-TOF) m/z: $[M + H]^+$ Calcd for $C_{16}H_{13}FNO_3S$ 318.0595; Found 318.0592.

(*E*)-3-Benzylidene-6-chloro-2-(methylsulfonyl)isoindolin-1-one (4l)

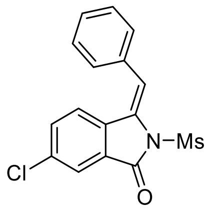

Starting materials: 5-Chloro-2-iodo-*N*-(methylsulfonyl)benzamide (**3c**) (0.18 g, 0.5 mmol, 1.0 equiv.), phenylacetylene (0.055 mL, 0.5 mmol, 1.0 equiv.), and copper(I) oxide (0.021 g, 0.15 mmol, 0.3 equiv.) using the same method for **4j**.

Purification conditions: Combiflash ISCO column using hexane/ethyl acetate as mobile phase (%elution of desired compound= 20% ethyl acetate in hexane).

Yield: 0.113 g (68%); white solid.

^{1}H NMR (400 MHz, DMSO-d_6) δ 8.15 (d, J = 2.3 Hz, 1H), 8.06 – 7.81 (m, 3H), 7.80 – 7.64 (m, 2H), 7.55 (dt, J = 10.9, 6.5 Hz, 3H), 3.26 (s, 3H); ^{13}C NMR (100 MHz, DMSO-d_6) δ 165.2, 157.4, 152.1, 135.8, 134.4, 133.3, 132.8, 130.6, 129.1, 129.0, 127.1, 126.5, 125.0, 12102, 102.6, 42.3; MS (ESI) m/z: [M + H]$^+$ Calcd for $C_{16}H_{13}ClNO_3S$ 334.03; Found 334.00; HRMS (ESI-TOF) m/z: [M + H]$^+$ Calcd for $C_{16}H_{13}ClNO_3S$ 334.0300; Found 334.0304.

(*E*)-3-Benzylidene-6-bromo-2-(methylsulfonyl)isoindolin-1-one (4m)

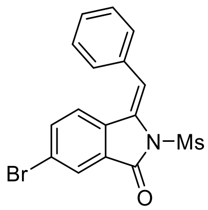

Starting materials: 5-Bromo-2-iodo-*N*-(methylsulfonyl)benzamide (**3d**) (0.202 g, 0.5 mmol, 1.0 equiv.), phenylacetylene (0.055 mL, 0.5 mmol, 1.0 equiv.), and copper(I) oxide (0.021 g, 0.15 mmol, 0.3 equiv.) using the same method for **4j**.

Purification conditions: Combiflash ISCO column using hexane/ethyl acetate as mobile phase (%elution of desired compound= 20% ethyl acetate in hexane).

Yield: 0.116 g (62%); colorless oil.

^{1}H NMR (400 MHz, DMSO-d_6) δ 8.29 (d, J = 2.1 Hz, 1H), 8.16 – 8.00 (m, 3H), 7.75 (s, 1H), 7.69 (d, J = 8.4 Hz, 1H), 7.54 (dq, J = 13.7, 7.1 Hz, 3H), 3.26 (s, 3H); ^{13}C NMR (100 MHz, DMSO-d_6) δ 157.2, 152.2, 138.5, 134.6, 130.6, 130.4, 129.5, 129.1, 129.0, 125.0, 121.6, 121.4, 102.7, 42.3; MS (ESI) m/z: [M + H]$^+$ Calcd for $C_{16}H_{13}BrNO_3S$ 377.98; Found 378.0; HRMS (ESI-TOF) m/z: [M + H]$^+$ Calcd for $C_{16}H_{13}BrNO_3S$ 377.9795; Found 377.9794.

(*E*)-3-Benzylidene-6-methyl-2-(methylsulfonyl)isoindolin-1-one (4n)

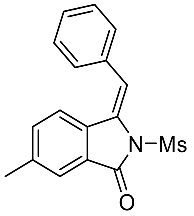

Starting materials: 2-Iodo-5-methyl-*N*-(methylsulfonyl)benzamide (**3e**) (0.17 g, 0.5 mmol, 1.0 equiv.), phenylacetylene (0.055 mL, 0.5 mmol, 1.0 equiv.), and copper(I) oxide (0.021 g, 0.15 mmol, 0.3 equiv.) using the same method for **4j**.

Purification conditions: Combiflash ISCO column using hexane/ethyl acetate as mobile phase (%elution of desired compound= 15% ethyl acetate in hexane).

Yield: 0.114 g (73%); white solid.

^{1}H NMR (400 MHz, DMSO-*d*$_6$) δ 8.05 (dd, *J* = 6.5, 2.4 Hz, 3H), 7.79 – 7.70 (m, 2H), 7.66 (d, *J* = 8.1 Hz, 1H), 7.54 (dt, *J* = 15.3, 7.2 Hz, 3H), 3.24 (s, 3H), 2.47 (s, 3H); ^{13}C NMR (100 MHz, DMSO-*d*$_6$) δ 158.7, 151.1, 139.5, 137.2, 133.2, 130.7, 130.2, 129.1, 127.2, 126.9, 124.8, 119.5, 103.3, 42.2, 21.0; MS (ESI) m/z: [M + H]$^+$ Calcd for $C_{17}H_{16}NO_3S$ 314.08; Found 314.10; HRMS (ESI-TOF) m/z: [M + H]$^+$ Calcd for $C_{17}H_{16}NO_3S$ 314.0846; Found 314.0841.

(*E*)-3-Benzylidene-6-methoxy-2-(methylsulfonyl)isoindolin-1-one (4o)

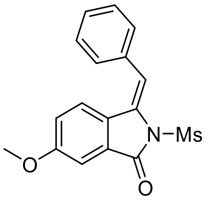

Starting materials: 2-Iodo-5-methoxy-*N*-(methylsulfonyl)benzamide (**3f**) (0.15 g, 0.42 mmol, 1.0 equiv.), phenylacetylene (0.046 mL, 0.42 mmol, 1.0 equiv.), and copper(I) oxide (0.018 g, 0.13 mmol, 0.3 equiv.) using the same method for **4j**.

Purification conditions: Combiflash ISCO column using hexane/ethyl acetate as mobile phase (%elution of desired compound= 25% ethyl acetate in hexane).

Yield: 0.095 g (69%); colorless oil.

^{1}H NMR (400 MHz, DMSO-*d*$_6$) δ 8.03 (d, *J* = 7.7 Hz, 2H), 7.72 (d, *J* = 8.3 Hz, 2H), 7.61 (d, *J* = 2.6 Hz, 1H), 7.59 – 7.49 (m, 3H), 7.48 (d, *J* = 7.4 Hz, 1H), 3.91 (s, 3H), 3.25 (s, 3H); ^{13}C NMR (100 MHz, DMSO-*d*$_6$) δ 159.7, 158.6, 150.2, 130.8, 130.0, 129.4, 129.1, 128.8, 125.3, 124.6, 120.9, 108.4, 103.2, 55.8, 42.1; MS (ESI) m/z: [M + H]$^+$ Calcd for $C_{17}H_{16}NO_4S$ 330.08; Found 330.1; HRMS (ESI-TOF) m/z: [M + H]$^+$ Calcd for $C_{17}H_{16}NO_4S$ 330.0795; Found 330.0794.

(*E*)-3-Benzylidene-2-(methylsulfonyl)-6-nitroisoindolin-1-one (4p)

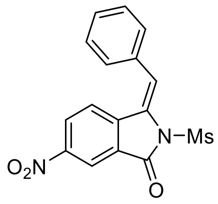

Starting materials: 2-Iodo-5-nitro-*N*-(methylsulfonyl)benzamide (**3g**) (0.125 g, 0.34 mmol, 1.0 equiv.), phenylacetylene (0.037 mL, 0.34 mmol, 1.0 equiv.), and copper(I) oxide (0.014 g, 0.1 mmol, 0.3 equiv.) using the same method for **4j**.

Purification conditions: Combiflash ISCO column using hexane/ethyl acetate as mobile phase (%elution of desired compound= 20% ethyl acetate in hexane).

Yield: 0.083 g (71%); pale-yellow oil.

^{1}H NMR (400 MHz, DMSO-d_6) δ 8.85 (d, J = 2.4 Hz, 1H), 8.63 (dd, J = 8.7, 2.4 Hz, 1H), 8.12 – 8.07 (m, 2H), 7.94 (d, J = 8.6 Hz, 1H), 7.90 (s, 1H), 7.60 (d, J = 6.9 Hz, 3H), 3.31 (s, 3H); ^{13}C NMR (100 MHz, DMSO-d_6) δ 157.3, 154.5, 146.7, 140.5, 131.4, 130.0, 129.5, 129.3, 129.2, 129.2, 128.6, 125.5, 123.0, 120.4, 102.5, 42.3; MS (ESI) m/z: $[M + H]^+$ Calcd for $C_{16}H_{13}N_2O_5S$ 345.05; Found 345.10 $[M + H]^+$; HRMS (ESI-TOF) m/z: $[M + H]^+$ Calcd for $C_{16}H_{13}N_2O_5S$ 345.0540; Found 345.0539.

(*E*)-3-Benzylidene-5-chloro-2-(methylsulfonyl)isoindolin-1-one (4q)

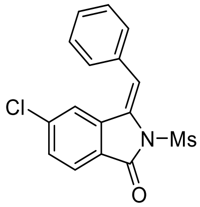

Starting materials: 4-Chloro-2-Iodo-*N*-(methylsulfonyl)benzamide (**3h**) (0.18 g, 0.5 mmol, 1.0 equiv.), phenylacetylene (0.055 mL, 0.5 mmol, 1.0 equiv.), and copper(I) oxide (0.021 g, 0.15 mmol, 0.3 equiv.) using the same method for **4j**.

Purification conditions: Combiflash ISCO column using hexane/ethyl acetate as mobile phase (%elution of desired compound= 20% ethyl acetate in hexane).

Yield: 0.117 g (70%); pale-yellow oil.

^{1}H NMR (400 MHz, DMSO-d_6) δ 8.20 (d, J = 8.6 Hz, 1H), 8.02 (d, J = 7.5 Hz, 2H), 7.84 (s, 1H), 7.68 (d, J = 9.6 Hz, 2H), 7.56 (dd, J = 10.7, 7.0 Hz, 3H), 3.24 (s, 3H); ^{13}C NMR (100 MHz, DMSO-d_6) δ 158.0, 152.8, 140.7, 137.2, 130.8, 130.4, 129.9, 129.4, 129.2, 126.0, 125.1, 118.5, 102.4, 42.2; MS (ESI) m/z: $[M + H]^+$ Calcd for $C_{16}H_{13}ClNO_3S$ 334.03; Found 334.00; HRMS (ESI-TOF) m/z: $[M + H]^+$ Calcd for $C_{16}H_{13}ClNO_3S$ 334.0300; Found 334.0299.

(*E*)-3-Benzylidene-2-(methylsulfonyl)-5-nitroisoindolin-1-one (4r)

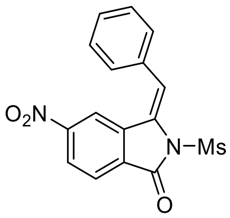

Starting materials: 2-Iodo-4-nitro-*N*-(methylsulfonyl)benzamide (**3i**) (0.185 g, 0.5 mmol, 1.0 equiv.), phenylacetylene (0.055 mL, 0.5 mmol, 1.0 equiv.), and copper(I) oxide (0.021 g, 0.15 mmol, 0.3 equiv.) using the same method for **4j**.

Purification conditions: Combiflash ISCO column using hexane/ethyl acetate as mobile phase (%elution of desired compound= 30% ethyl acetate in hexane).

Yield: 0.12 g (67%); pale-yellow oil.

^{1}H NMR (400 MHz, DMSO-*d*$_6$) δ 8.56 (d, *J* = 2.2 Hz, 1H), 8.39 (d, *J* = 8.8 Hz, 1H), 8.31 (td, *J* = 5.9, 2.8 Hz, 1H), 8.15 (d, *J* = 8.5 Hz, 1H), 8.05 – 7.98 (m, 1H), 7.90 (s, 1H), 7.67 – 7.45 (m, 3H), 3.29 (s, 3H); ^{13}C NMR (100 MHz, DMSO-*d*$_6$) δ 157.2, 153.1, 151.4, 136.7, 131.0, 130.1, 129.9, 129.8, 129.2, 125.1, 124.2, 123.4, 122.8, 121.6, 102.7, 42.3; MS (ESI) m/z: [M + H]$^+$ Calcd for $C_{16}H_{13}N_2O_5S$ 345.05; Found 345.1; HRMS (ESI-TOF) m/z: [M + H]$^+$ Calcd for $C_{16}H_{13}N_2O_5S$ 345.0540; Found 345.0545.

(*E*)-2-(Methylsulfonyl)-3-(2-oxopropylidene)isoindolin-1-one (4s)

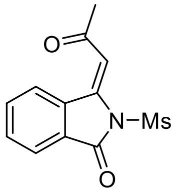

To a solution of 2-Iodo-*N*-(methylsulfonyl)benzamide (**3a**) (0.162 g, 0.5 mmol, 1.0 equiv.) in DMF (0.75 mL), 3-butyn-2-one (0.04 mL, 0.5 mmol, 1.0 equiv.), and copper(I) oxide (0.021 g, 0.15 mmol, 0.3 equiv.) were added and then the reaction mixture was heated at 95 °C for 6 hours. The crude mixture was cooled to room temperature, quenched with 1N HCl (20 mL), and then extracted with ethyl acetate (20 mL X 3). The organic phase was separated, dried over anhydrous Na_2SO_4, and concentrated under reduced pressure. The residue was purified by Combiflash ISCO column (ethyl acetate/hexane; %elution of desired compound= 25% ethyl acetate in hexane) to give the desired product as pale-yellow oil in 70% yield (0.093 g); ^{1}H NMR (400 MHz, DMSO-*d*$_6$) δ 8.09 (d, *J* = 7.8 Hz, 1H), 7.95 – 7.81 (m, 2H), 7.71 (t, *J* = 7.5 Hz, 1H), 6.94 (s, 1H), 3.57 (s, 3H), 2.32 (s, 3H); ^{13}C NMR (100 MHz, DMSO-*d*$_6$) δ 199.0, 166.0, 138.4, 135.3, 133.3, 131.5, 126.0, 124.5, 121.3, 111.8, 41.7, 30.6, 20.9; MS (ESI) m/z: [M + H]$^+$ Calcd for $C_{12}H_{12}NO_4S$ 266.05; Found 266.0; HRMS (ESI-TOF) m/z: [M + H]$^+$ Calcd for $C_{12}H_{12}NO_4S$ 266.0482; Found 266.0476.

(*E*)-6-Methyl-2-(methylsulfonyl)-3-(2-oxopropylidene)isoindolin-1-one (4t)

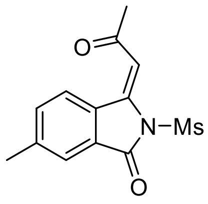

Starting materials: 2-Iodo-5-methyl-*N*-(methylsulfonyl)benzamide (**3e**) (0.17 g, 0.5 mmol, 1.0 equiv.), 3-butyn-2-one (0.039 mL, 0.5 mmol, 1.0 equiv.), and copper(I) oxide (0.021 g, 0.15 mmol, 0.3 equiv.) using the same method for **4s**.

Purification conditions: Combiflash ISCO column using hexane/ethyl acetate as mobile phase (%elution of desired compound= 20% ethyl acetate in hexane).

Yield: 0.096 g (69%); white solid.

^{1}H NMR (400 MHz, DMSO-*d₆*) δ 7.96 (d, J = 8.0 Hz, 1H), 7.70 (q, J = 8.0, 7.5 Hz, 2H), 6.86 (s, 1H), 3.56 (s, 3H), 2.45 (s, 3H), 2.30 (s, 3H); ^{13}C NMR (100 MHz, DMSO-d_6) δ 199.0, 166.2, 142.1, 136.3, 136.0, 133.6, 126.2, 124.3, 121.2, 111.0, 41.8, 30.6, 20.9; MS (ESI) m/z: [M + H]$^+$ Calcd for $C_{13}H_{14}NO_4S$ 280.06; Found 280.10; HRMS (ESI-TOF) m/z: [M + H]$^+$ Calcd for $C_{13}H_{14}NO_4S$ 280.0639; Found 280.0648.

(*E*)-6-Methoxy-2-(methylsulfonyl)-3-(2-oxopropylidene)isoindolin-1-one (4u)

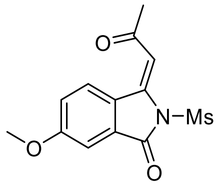

Starting materials: 2-Iodo-5-methoxy-*N*-(methylsulfonyl)benzamide (**3f**) (0.178 g, 0.5 mmol, 1.0 equiv.), 3-butyn-2-one (0.039 mL, 0.5 mmol, 1.0 equiv.), and copper(I) oxide (0.021 g, 0.15 mmol, 0.3 equiv.) using the same method for **4s**.

Purification conditions: Combiflash ISCO column using hexane/ethyl acetate as mobile phase (%elution of desired compound= 35% ethyl acetate in hexane).

Yield: 0.104 g (71%); white solid.

^{1}H NMR (400 MHz, DMSO-d_6) δ 7.99 (d, J = 8.6 Hz, 1H), 7.42 (dd, J = 8.6, 2.4 Hz, 1H), 7.36 (d, J = 2.4 Hz, 1H), 6.81 (s, 1H), 3.89 (s, 3H), 3.57 (s, 3H), 2.29 (s, 3H); ^{13}C NMR (100 MHz, DMSO-d_6) δ 198.8, 166.1, 162.1, 133.7, 131.0, 127.8, 123.5, 122.9, 110.2, 106.9, 56.1, 41.8, 30.6; MS (ESI) m/z: [M + H]$^+$ Calcd for $C_{13}H_{14}NO_5S$ 296.06; Found 296.10; HRMS (ESI-TOF) m/z: [M + H]$^+$ Calcd for $C_{13}H_{14}NO_5S$ 296.0588; Found 296.0587.

Ethyl (*E*)-2-(3-oxoisoindolin-1-ylidene)acetate (5a)[5]

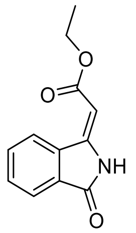

Compound (**4a**) (0.123 g, 0.42 mmol, 1.0 equiv.) was dissolved in THF (2.0 mL) followed by addition of TBAF TBAF (1M in THF) (0.84 mL, 0.84 mmol, 2.0 equiv.). After that, the reaction mixture was stirred at room temperature for 8 hours. After the reaction was complete as indicated by TLC and LC-MS, the reaction mixture was concentrated under reduced pressure. The obtained oily residue was purified by Combiflash ISCO column (ethyl acetate/hexane; %elution of desired compound= 10% ethyl acetate in hexane) to give the desired product as pale-yellow oil in 83% yield (0.076 g); ^{1}H NMR (400 MHz, DMSO-d_6) δ 10.33 (s, 1H), 8.09 (d, J = 7.5 Hz, 1H), 7.80 (d, J = 7.4 Hz, 1H), 7.70 (dt, J = 24.1, 7.3 Hz, 2H), 6.08 (s, 1H), 4.20 (q, J = 7.1 Hz, 2H), 1.26 (t, J = 7.1 Hz, 3H); ^{13}C NMR (100 MHz, DMSO-d_6) δ 168.0, 166.3, 146.6, 136.5, 133.1, 131.7, 128.8, 123.2, 122.0, 92.1, 60.0, 14.2; MS (ESI) m/z: $[M + H]^+$ Calcd for $C_{12}H_{12}NO_3$ 218.08; Found 218.10; HRMS (ESI) m/z: $[M + H]^+$ Calcd for $C_{12}H_{12}NO_3$ 218.0812; Found 218.0812.

Ethyl (*E*)-2-(5-fluoro-3-oxoisoindolin-1-ylidene)acetate (5b)

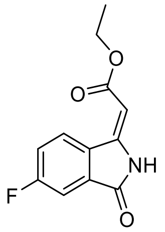

Starting materials: Compound (**4b**) (0.1 g, 0.32 mmol, 1.0 equiv.) and TBAF (1M in THF) (0.64 mL, 0.64 mmol, 2.0 equiv.) in 2 mL of THF using the same method for **5a**.

Purification conditions: Combiflash ISCO column using hexane/ethyl acetate as mobile phase (%elution of desired compound= 10% ethyl acetate in hexane).

Yield: 0.064 g (85%); pale-yellow oil.

^{1}H NMR (400 MHz, DMSO-d_6) δ 10.49 (s, 1H), 8.18 (dd, J = 8.4, 4.5 Hz, 1H), 7.61 (ddd, J = 17.4, 7.9, 2.4 Hz, 2H), 6.12 (s, 1H), 4.21 (q, J = 7.1 Hz, 2H), 1.26 (t, J = 7.1 Hz, 3H); ^{13}C NMR (100 MHz, DMSO-d_6) δ 166.8, 166.1, 162.9, 145.7, 132.6, 131.2, 124.6, 120.4, 110.1, 92.6, 60.0, 14.2; MS (ESI) m/z: $[M + H]^+$ Calcd for $C_{12}H_{11}FNO_3$ 236.07; Found 236.10; HRMS (ESI-TOF) m/z: $[M + H]^+$ Calcd for $C_{12}H_{11}FNO_3$ 236.0718; Found 236.0718.

Ethyl (*E*)-2-(5-chloro-3-oxoisoindolin-1-ylidene)acetate (5c)

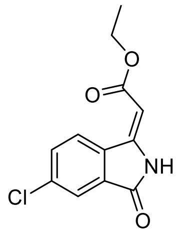

Starting materials: Compound (**4c**) (0.12 g, 0.36 mmol, 1.0 equiv.) and TBAF (1M in THF) (0.72 mL, 0.72 mmol, 2.0 equiv.) in 2 mL of THF using the same method for **5a**.

Purification conditions: Combiflash ISCO column using hexane/ethyl acetate as mobile phase (%elution of desired compound= 10% ethyl acetate in hexane).

Yield: 0.074 g (82%); colorless oil.

^{1}H NMR (400 MHz, DMSO-d_6) δ 10.53 (s, 1H), 8.15 (d, J = 8.1 Hz, 1H), 7.86 – 7.77 (m, 2H), 6.16 (s, 1H), 4.22 (q, J = 7.1 Hz, 2H), 1.27 (t, J = 7.1 Hz, 3H); ^{13}C NMR (100 MHz, DMSO-d_6) δ 166.7, 166.0, 145.5, 136.5, 135.2, 133.0, 130.7, 123.9, 123.1, 93.1, 60.0, 14.2; MS (ESI) m/z: [M + H]$^+$ Calcd for $C_{12}H_{11}ClNO_3$ 252.04; Found 252.00; HRMS (ESI-TOF) m/z: [M + H]$^+$ Calcd for $C_{12}H_{11}ClNO_3$ 252.0422; Found 252.0425.

Ethyl (*E*)-2-(5-bromo-3-oxoisoindolin-1-ylidene)acetate (5d)

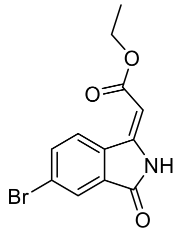

Starting materials: Compound (**4d**) (0.1 g, 0.27 mmol, 1.0 equiv.) and TBAF (1M in THF) (0.54 mL, 0.54 mmol, 2.0 equiv.) in 2 mL of THF using the same method for **5a**.

Purification conditions: Combiflash ISCO column using hexane/ethyl acetate as mobile phase (%elution of desired compound= 10% ethyl acetate in hexane).

Yield: 0.065 g (82%); white solid.

^{1}H NMR (400 MHz, DMSO-d_6) δ 10.52 (s, 1H), 8.08 (d, J = 8.0 Hz, 1H), 7.95 (d, J = 11.9 Hz, 2H), 6.16 (s, 1H), 4.22 (q, J = 7.0 Hz, 2H), 1.26 (t, J = 7.0 Hz, 3H); ^{13}C NMR (100 MHz, DMSO-d_6) δ 166.6, 166.0, 145.6, 135.8, 135.5, 130.9, 126.0, 125.0, 124.1, 93.1, 60.0, 14.2; MS (ESI) m/z: [M + H]$^+$ Calcd for $C_{12}H_{11}BrNO_3$ 295.99; Found 296.00; HRMS (ESI-TOF) m/z: [M + H]$^+$ Calcd for $C_{12}H_{11}BrNO_3$ 295.9917; Found 295.9919.

Ethyl (*E*)-2-(5-methyl-3-oxoisoindolin-1-ylidene)acetate (5e)

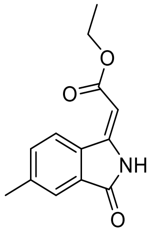

Starting materials: Compound (**4e**) (0.08 g, 0.26 mmol, 1.0 equiv.) and TBAF (1M in THF) (0.52 mL, 0.52 mmol, 2.0 equiv.) in 2 mL of THF using the same method for **5a**.

Purification conditions: Combiflash ISCO column using hexane/ethyl acetate as mobile phase (%elution of desired compound= 20% ethyl acetate in hexane).

Yield: 0.052 g (86%); white solid.

^{1}H NMR (400 MHz, DMSO-d_6) δ 10.24 (s, 1H), 7.96 (d, J = 7.8 Hz, 1H), 7.60 (s, 1H), 7.54 (d, J = 7.9 Hz, 1H), 6.02 (s, 1H), 4.20 (q, J = 7.1 Hz, 2H), 2.44 (s, 3H), 1.26 (t, J = 7.1 Hz, 3H); ^{13}C NMR (100 MHz, DMSO-d_6) δ 168.0, 166.4, 146.8, 142.2, 133.9, 133.8, 129.1, 123.4, 121.8, 91.4, 59.9, 21.1, 14.2; MS (ESI) m/z: $[M + H]^+$ Calcd for $C_{13}H_{14}NO_3$ 232.10; Found 232.10; HRMS (ESI-TOF) m/z: $[M + H]^+$ Calcd for $C_{13}H_{14}NO_3$ 232.0969; Found 232.0970.

Ethyl (*E*)-2-(5-methoxy-3-oxoisoindolin-1-ylidene)acetate (5f)

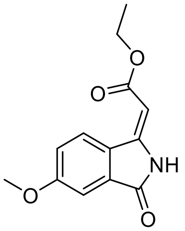

Starting materials: Compound (**4f**) (0.05 g, 0.15 mmol, 1.0 equiv.) and TBAF (1M in THF) (0.3 mL, 0.3 mmol, 2.0 equiv.) in 2 mL of THF using the same method for **5a**.

Purification conditions: Combiflash ISCO column using hexane/ethyl acetate as mobile phase (%elution of desired compound= 15% ethyl acetate in hexane).

Yield: 0.032 g (85%); white solid.

^{1}H NMR (400 MHz, DMSO-d_6) δ 10.26 (s, 1H), 8.00 (d, J = 8.4 Hz, 1H), 7.29 (d, J = 2.3 Hz, 1H), 7.26 (dd, J = 8.4, 2.4 Hz, 1H), 5.99 (s, 1H), 4.20 (q, J = 7.1 Hz, 2H), 3.88 (s, 3H), 1.26 (t, J = 7.1 Hz, 3H); ^{13}C NMR (100 MHz, DMSO-d_6) δ 167.7, 166.4, 162.4, 146.7, 130.9, 128.7, 123.6, 120.1, 107.0, 91.0, 59.8, 55.9, 14.2; MS (ESI) m/z: $[M + H]^+$ Calcd for $C_{13}H_{14}NO_4$ 248.09; Found 248.10; HRMS (ESI-TOF) m/z: $[M + H]^+$ Calcd for $C_{13}H_{14}NO_4$ 248.0918; Found 248.0919.

Ethyl (*E*)-2-(5-nitro-3-oxoisoindolin-1-ylidene)acetate (5g)

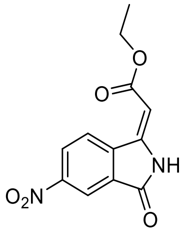

Starting materials: Compound (**4g**) (0.1 g, 0.29 mmol, 1.0 equiv.) and TBAF (1M in THF) (0.58 mL, 0.58 mmol, 2.0 equiv.) in 2 mL of THF using the same method for **5a**.

Purification conditions: Combiflash ISCO column using hexane/ethyl acetate as mobile phase (%elution of desired compound= 20% ethyl acetate in hexane).

Yield: 0.06 g (80%); pale-yellow oil.

^{1}H NMR (400 MHz, DMSO-d_6) δ 10.86 (s, 1H), 8.57 (dd, J = 8.3, 2.1 Hz, 1H), 8.47 – 8.38 (m, 2H), 6.35 (s, 1H), 4.23 (p, J = 7.1 Hz, 2H), 1.28 (t, J = 7.0 Hz, 3H); ^{13}C NMR (100 MHz, DMSO-d_6) δ 166.2, 165.7, 149.7, 144.5, 141.6, 130.1, 128.0, 123.6, 118.3, 95.6, 60.3, 14.2; MS (ESI) m/z: $[M + H]^+$ Calcd for $C_{12}H_{11}N_2O_5$ 263.07; Found 263.10; HRMS (ESI-TOF) m/z: $[M + H]^+$ Calcd for $C_{12}H_{11}N_2O_5$ 263.0663; Found 263.0664.

Ethyl (*E*)-2-(6-chloro-3-oxoisoindolin-1-ylidene)acetate (5h)

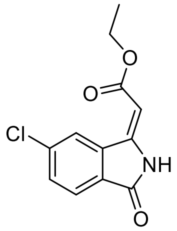

Starting materials: Compound (**4h**) (0.09 g, 0.27 mmol, 1.0 equiv.) and TBAF (1M in THF) (0.54 mL, 0.54 mmol, 2.0 equiv.) in 2 mL of THF using the same method for **5a**.

Purification conditions: Combiflash ISCO column using hexane/ethyl acetate as mobile phase (%elution of desired compound= 10% ethyl acetate in hexane).

Yield: 0.056 g (84%); white solid.

^{1}H NMR (400 MHz, DMSO-d_6) δ 10.50 (s, 1H), 8.32 (d, J = 1.7 Hz, 1H), 7.81 (d, J = 8.0 Hz, 1H), 7.72 (dd, J = 8.1, 1.7 Hz, 1H), 6.21 (s, 1H), 4.22 (q, J = 7.0 Hz, 2H), 1.27 (t, J = 7.1 Hz, 3H); ^{13}C NMR (100 MHz, DMSO-d_6) δ 167.0, 166.0, 145.3, 138.5, 138.1, 131.7, 127.5, 124.9, 122.4, 93.4, 60.1, 14.2; MS (ESI) m/z: $[M + H]^+$ Calcd for $C_{12}H_{11}ClNO_3$ 252.04; Found 252.00; HRMS (ESI-TOF) m/z: $[M + H]^+$ Calcd for $C_{12}H_{11}ClNO_3$ 252.0422; Found 252.0423.

Ethyl (*E*)-2-(6-nitro-3-oxoisoindolin-1-ylidene)acetate (5i)

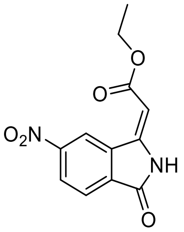

Starting materials: Compound (**4i**) (0.05 g, 0.15 mmol, 1.0 equiv.) and TBAF (1M in THF) (0.3 mL, 0.3 mmol, 2.0 equiv.) in 2 mL of THF using the same method for **5a**.

Purification conditions: Combiflash ISCO column using hexane/ethyl acetate as mobile phase (%elution of desired compound= 20% ethyl acetate in hexane).

Yield: 0.033 g (85%); pale-yellow oil.

^{1}H NMR (400 MHz, DMSO-*d$_6$*) δ 10.86 (s, 1H), 9.07 (d, *J* = 2.0 Hz, 1H), 8.47 (dd, *J* = 8.3, 1.9 Hz, 1H), 8.05 (d, *J* = 8.3 Hz, 1H), 6.49 (s, 1H), 4.24 (q, *J* = 7.1 Hz, 2H), 1.28 (t, *J* = 7.1 Hz, 3H); ^{13}C NMR (100 MHz, DMSO-*d$_6$*) δ 166.3, 165.9, 150.9, 144.6, 137.8, 133.5, 126.6, 124.6, 117.9, 94.9, 60.2, 14.2; MS (ESI) m/z: $[M + H]^+$ Calcd for $C_{12}H_{11}N_2O_5$ 263.07; Found 263.10; HRMS (ESI-TOF) m/z: $[M + H]^+$ Calcd for $C_{12}H_{11}N_2O_5$ 263.0663; Found 263.0665.

***N*-(Methylsulfonyl)-2-((triisopropylsilyl)ethynyl)benzamide (6a)**

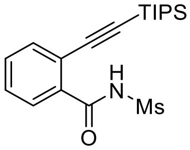

To a solution of 2-Iodo-*N*-(methylsulfonyl)benzamide (**3a**) (0.162 g, 0.5 mmol, 1.0 equiv.) in DMF (0.75 mL), (triisopropylsilyl)acetylene (0.112 mL, 0.5 mmol, 1.0 equiv.), and copper(I) oxide (0.021 g, 0.15 mmol, 0.3 equiv.) were added and then the reaction mixture was heated at 110 °C for 12 hours. The crude mixture was cooled to room temperature, quenched with 1N HCl (20 mL), and then extracted with ethyl acetate (20 mL X 3). The organic phase was separated, dried over anhydrous Na_2SO_4, and concentrated under reduced pressure. The residue was purified by Combiflash ISCO column (ethyl acetate/hexane; %elution of desired compound= 10% ethyl acetate in hexane) to give the desired product as colorless oil in 79% yield (0.149 g); ^{1}H NMR (400 MHz, DMSO-*d$_6$*) δ 12.18 (s, 1H), 7.61 – 7.45 (m, 4H), 3.33 (s, 3H), 1.10 (s, 21H); ^{13}C NMR (100 MHz, DMSO-*d$_6$*) δ 166.8, 136.3, 133.6, 131.1, 128.7, 128.1, 120.6, 104.0, 95.0, 41.0, 18.5, 10.7; MS (ESI) m/z: $[M + H]^+$ Calcd for $C_{19}H_{30}NO_3SSi$ 380.17; Found 380.20; HRMS (ESI) m/z: $[M + H]^+$ Calcd for $C_{19}H_{30}NO_3SSi$ 380.1711; Found 380.1716.

5-Fluoro-*N*-(methylsulfonyl)-2-((triisopropylsilyl)ethynyl)benzamide (6b)

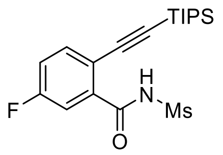

Starting materials: 5-Fluoro-2-iodo-*N*-(methylsulfonyl)benzamide (**3b**) (0.15 g, 0.44 mmol, 1.0 equiv.), (triisopropylsilyl)acetylene (0.1 mL, 0.44 mmol, 1.0 equiv.), and copper(I) oxide (0.019 g, 0.13 mmol, 0.3 equiv.) using the same method for **6a**.

Purification conditions: Combiflash ISCO column using hexane/ethyl acetate as mobile phase (%elution of desired compound= 5% ethyl acetate in hexane).

Yield: 0.136 g (78%); pale-yellow oil.

^{1}H NMR (400 MHz, DMSO-d_6) δ 12.27 (s, 1H), 7.62 (dd, J = 8.6, 5.4 Hz, 1H), 7.51 (dd, J = 9.0, 2.7 Hz, 1H), 7.40 (td, J = 8.6, 2.8 Hz, 1H), 3.34 (s, 3H), 1.09 (s, 21H); ^{13}C NMR (100 MHz, DMSO-d_6) δ 165.9, 162.8, 160.3, 139.0, 136.4, 118.6, 117.5, 116.1, 103.3, 95.2, 41.5, 18.9, 11.2; MS (ESI) m/z: $[M + H]^+$ Calcd for $C_{19}H_{29}FNO_3SSi$ 398.16; Found 398.20; HRMS (ESI-TOF) m/z: $[M + H]^+$ Calcd for $C_{19}H_{29}FNO_3SSi$ 398.1616; Found 398.1619.

5-Bromo-*N*-(methylsulfonyl)-2-((triisopropylsilyl)ethynyl)benzamide (6c)

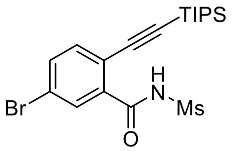

Starting materials: 5-Bromo-2-iodo-*N*-(methylsulfonyl)benzamide (**3d**) (0.202 g, 0.5 mmol, 1.0 equiv.), (triisopropylsilyl)acetylene (0.11 mL, 0.5 mmol, 1.0 equiv.), and copper(I) oxide (0.021 g, 0.15 mmol, 0.3 equiv.) using the same method for **6a**.

Purification conditions: Combiflash ISCO column using hexane/ethyl acetate as mobile phase (%elution of desired compound= 10% ethyl acetate in hexane).

Yield: 0.16 g (70%); pale-yellow oil.

^{1}H NMR (400 MHz, DMSO-d_6) δ 12.28 (s, 1H), 7.82 (d, J = 2.0 Hz, 1H), 7.73 (dd, J = 8.3, 2.1 Hz, 1H), 7.50 (d, J = 8.3 Hz, 1H), 3.33 (s, 3H), 1.08 (d, J = 2.5 Hz, 21H); ^{13}C NMR (100 MHz, DMSO-d_6) δ 165.2, 138.0, 135.2, 133.8, 130.8, 121.6, 119.7, 102.8, 96.5, 41.0, 18.4, 10.6; MS (ESI) m/z: $[M + H]^+$ Calcd for $C_{19}H_{29}BrNO_3SSi$ 458.08; Found 458.10; HRMS (ESI-TOF) m/z: $[M + H]^+$ Calcd for $C_{19}H_{29}BrNO_3SSi$ 458.0816; Found 458.0818.

5-Methyl-*N*-(methylsulfonyl)-2-((triisopropylsilyl)ethynyl)benzamide (6d)

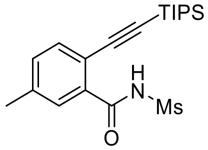

Starting materials: 2-Iodo-5-methyl-*N*-(methylsulfonyl)benzamide (**3e**) (0.17 g, 0.5 mmol, 1.0 equiv.), (triisopropylsilyl)acetylene (0.11 mL, 0.5 mmol, 1.0 equiv.), and copper(I) oxide (0.021 g, 0.15 mmol, 0.3 equiv.) using the same method for **6a**.

Purification conditions: Combiflash ISCO column using hexane/ethyl acetate as mobile phase (%elution of desired compound= 10% ethyl acetate in hexane).

Yield: 0.141 g (72%); pale-yellow oil.

^{1}H NMR (400 MHz, DMSO-d_6) δ 12.11 (s, 1H), 7.46 (d, J = 7.9 Hz, 1H), 7.41 (s, 1H), 7.35 (d, J = 7.9 Hz, 1H), 3.32 (s, 3H), 2.36 (s, 3H), 1.09 (s, 21H); ^{13}C NMR (100 MHz, DMSO-d_6) δ 166.8, 138.7, 136.1, 133.5, 131.6, 128.6, 117.7, 104.1, 94.0, 41.0, 20.7, 18.5, 10.7; MS (ESI) m/z: $[M + H]^+$ Calcd for $C_{20}H_{32}NO_3SSi$ 394.19; Found 394.20; HRMS (ESI-TOF) m/z: $[M + H]^+$ Calcd for $C_{20}H_{32}NO_3SSi$ 394.1867; Found 394.1871.

5-Methoxy-*N*-(methylsulfonyl)-2-((triisopropylsilyl)ethynyl)benzamide (6e)

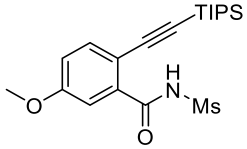

Starting materials: 2-Iodo-5-methoxy-*N*-(methylsulfonyl)benzamide (**3f**) (0.28 g, 0.79 mmol, 1.0 equiv.), (triisopropylsilyl)acetylene (0.18 mL, 0.79 mmol, 1.0 equiv.), and copper(I) oxide (0.043 g, 0.24 mmol, 0.3 equiv.) using the same method for **6a**.

Purification conditions: Combiflash ISCO column using hexane/ethyl acetate as mobile phase (%elution of desired compound= 10% ethyl acetate in hexane).

Yield: 0.236 g (73%); white solid.

^{1}H NMR (400 MHz, DMSO-d_6) δ 12.16 (s, 1H), 7.49 (d, J = 8.5 Hz, 1H), 7.15 – 7.05 (m, 2H), 3.84 (s, 3H), 3.33 (s, 3H), 1.08 (s, 21H); ^{13}C NMR (100 MHz, DMSO-d_6) δ 166.3, 158.9, 137.7, 135.2, 117.1, 113.3, 112.7, 104.1, 92.8, 55.7, 41.0, 18.5, 10.7; MS (ESI) m/z: $[M + H]^+$ Calcd for $C_{20}H_{32}NO_4SSi$ 410.18; Found 410.20; HRMS (ESI) m/z: $[M + H]^+$ Calcd for $C_{20}H_{32}NO_4SSi$ 410.1816; Found 410.1817.

_N_-(Methylsulfonyl)-5-nitro-2-((triisopropylsilyl)ethynyl)benzamide (6f)

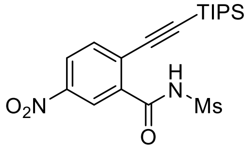

Starting materials: 2-Iodo-5-nitro-*N*-(methylsulfonyl)benzamide (**3g**) (0.185 g, 0.5 mmol, 1.0 equiv.), (triisopropylsilyl)acetylene (0.11 mL, 0.5 mmol, 1.0 equiv.) and copper(I) oxide (0.021 g, 0.15 mmol, 0.3 equiv.) using the same method for **6a**.

Purification conditions: Combiflash ISCO column using hexane/ethyl acetate as mobile phase (%elution of desired compound= 20% ethyl acetate in hexane).

Yield: 0.152 g (74%); pale-yellow oil.

^{1}H NMR (400 MHz, DMSO-d_6) δ 12.48 (s, 1H), 8.46 (d, J = 2.3 Hz, 1H), 8.34 (dd, J = 8.6, 2.4 Hz, 1H), 7.83 (d, J = 8.5 Hz, 1H), 3.38 (s, 3H), 1.10 (d, J = 4.7 Hz, 21H); ^{13}C NMR (100 MHz, DMSO-d_6) δ 164.8, 146.1, 137.5, 135.0, 126.8, 125.6, 123.3, 102.2, 101.5, 41.0, 18.4, 10.6; MS (ESI) m/z: $[M + H]^+$ Calcd for $C_{19}H_{29}N_2O_5SSi$ 425.17; Found 425.2; HRMS (ESI-TOF) m/z: $[M + H]^+$ Calcd for $C_{19}H_{29}N_2O_5SSi$ 425.1561; Found 425.1565.

4-Chloro-_N_-(methylsulfonyl)-2-((triisopropylsilyl)ethynyl)benzamide (6g)

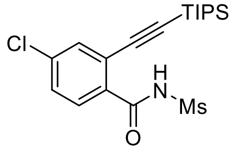

Starting materials: 4-Chloro-2-iodo-*N*-(methylsulfonyl)benzamide (**3h**) (0.18 g, 0.5 mmol, 1.0 equiv.), (triisopropylsilyl)acetylene (0.11 mL, 0.5 mmol, 1.0 equiv.), and copper(I) oxide (0.021 g, 0.15 mmol, 0.3 equiv.) using the same method for **6a**.

Purification conditions: Combiflash ISCO column using hexane/ethyl acetate as mobile phase (%elution of desired compound= 5% ethyl acetate in hexane).

Yield: 0.15 g (73%); pale-yellow oil.

^{1}H NMR (400 MHz, DMSO-d_6) δ 12.26 (s, 1H), 7.67 – 7.56 (m, 3H), 3.34 (s, 3H), 1.08 (s, 21H); ^{13}C NMR (100 MHz, DMSO-d_6) δ 165.9, 135.6, 135.1, 132.7, 130.0, 128.8, 122.5, 102.3, 97.0, 41.0, 18.4, 10.6; MS (ESI) m/z: $[M + H]^+$ Calcd for $C_{19}H_{29}ClNO_3SSi$ 414.13; Found 414.10; HRMS (ESI-TOF) m/z: $[M + H]^+$ Calcd for $C_{19}H_{29}ClNO_3SSi$ 414.1321; Found 414.1335.

N-(Methylsulfonyl)-4-nitro-2-((triisopropylsilyl)ethynyl)benzamide (6h)

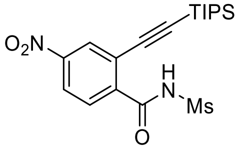

Starting materials: 2-Iodo-4-nitro-N-(methylsulfonyl)benzamide (**3i**) (0.185 g, 0.5 mmol, 1.0 equiv.), (triisopropylsilyl)acetylene (0.112 mL, 0.5 mmol, 1.0 equiv.), and copper(I) oxide (0.021 g, 0.15 mmol, 0.3 equiv.) using the same method for **6a**.

Purification conditions: Combiflash ISCO column using hexane/ethyl acetate as mobile phase (%elution of desired compound= 15% ethyl acetate in hexane).

Yield: 0.151 g (71%); pale-yellow oil.

^{1}H NMR (400 MHz, DMSO-d_6) δ 12.55 (s, 1H), 8.33 – 8.22 (m, 2H), 7.86 (d, J = 8.5 Hz, 1H), 3.36 (s, 3H), 1.10 (d, J = 4.1 Hz, 21H); ^{13}C NMR (100 MHz, DMSO-d_6) δ 165.6, 148.5, 141.8, 129.6, 127.6, 123.5, 121.8, 101.5, 98.1, 41.1, 18.4, 10.6; MS (ESI) m/z: $[M + H]^+$ Calcd for $C_{19}H_{29}N_2O_5SSi$ 425.17; Found 425.20; HRMS (ESI-TOF) m/z: $[M + H]^+$ Calcd for $C_{19}H_{29}N_2O_5SSi$ 425.1561; Found 425.1563.

Isoquinolin-1(2*H*)-one (7a)[6]

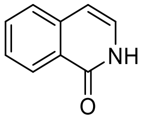

N-(Methylsulfonyl)-2-((triisopropylsilyl)ethynyl)benzamide (**6a**) (0.167 g, 0.44 mmol, 1.0 equiv.) was dissolved in THF (2.0 mL) followed by addition of TBAF (1M in THF) (0.88 mL, 0.88 mmol, 2.0 equiv.). After that, the reaction mixture was stirred at room temperature for 8 hours. After the reaction was complete as indicated by TLC and LC-MS, the reaction mixture was concentrated under reduced pressure. The obtained oily residue was purified by Combiflash ISCO column (ethyl acetate/hexane; %elution of desired compound= 50% ethyl acetate in hexane) to give the desired product as pale-yellow oil in 69% yield (0.044 g); ^{1}H NMR (400 MHz, DMSO-d_6) δ 11.22 (s, 1H), 8.18 (d, J = 8.0 Hz, 1H), 7.73 – 7.61 (m, 2H), 7.52 – 7.43 (m, 1H), 7.16 (t, J = 6.0 Hz, 1H), 6.54 (d, J = 7.1 Hz, 1H); ^{13}C NMR (100 MHz, DMSO-d_6) δ 161.8, 137.9, 132.3, 128.9, 126.6, 126.3, 126.2, 126.1, 104.6; LC/MS (ESI) m/z: $[M + H]^+$ Calcd for C_9H_8NO 146.06; Found 146.10; HRMS (ESI-TOF) m/z: $[M + H]^+$ Calcd for C_9H_8NO 146.0601; Found 146.0604.

7-Fluoroisoquinolin-1(2*H*)-one (7b)[7]

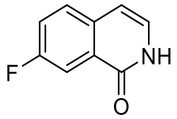

Starting materials: 5-Fluoro-*N*-(methylsulfonyl)-2-((triisopropylsilyl)ethynyl)benzamide (**6b**) (0.1 g, 0.25 mmol, 1.0 equiv.) and TBAF (1M in THF) (0.75 mL, 0.75 mmol, 3.0 equiv.) in 2 mL of THF using the same method for **7a**.

Purification conditions: Combiflash ISCO column using hexane/ethyl acetate as mobile phase (%elution of desired compound= 25% ethyl acetate in hexane).

Yield: 0.025 g (63%); pale-yellow oil.

^{1}H NMR (400 MHz, DMSO-d_6) δ 11.37 (s, 1H), 7.83 (dd, J = 9.6, 2.9 Hz, 1H), 7.76 (dd, J = 8.8, 5.3 Hz, 1H), 7.60 (td, J = 8.7, 2.9 Hz, 1H), 7.16 (t, J = 6.1 Hz, 1H), 6.59 (d, J = 7.1 Hz, 1H); ^{13}C NMR (100 MHz, DMSO-d_6) δ 161.1, 159.2, 134.8, 129.1, 128.2, 127.4, 120.9, 111.1, 104.2; MS (ESI) m/z: $[M + H]^+$ Calcd for C_9H_7FNO 164.05; Found 164.10; HRMS (ESI-TOF) m/z: $[M + H]^+$ Calcd for C_9H_7FNO 164.0507; Found 164.0505.

7-Bromoisoquinolin-1(2*H*)-one (7c)[7]

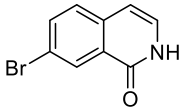

Starting materials: 5-Bromo-*N*-(methylsulfonyl)-2-((triisopropylsilyl)ethynyl)benzamide (**6c**) (0.121 g, 0.26 mmol, 1.0 equiv.) and TBAF (1M in THF) (0.78 mL, 0.78 mmol, 3.0 equiv.) in 2 mL of THF using the same method for **7a**.

Purification conditions: Combiflash ISCO column using hexane/ethyl acetate as mobile phase (%elution of desired compound= 20% ethyl acetate in hexane).

Yield: 0.035 g (61%); white solid.

^{1}H NMR (400 MHz, DMSO-d_6) δ 11.43 (s, 1H), 8.26 (d, J = 2.2 Hz, 1H), 7.85 (dd, J = 8.2, 2.4 Hz, 1H), 7.64 (d, J = 8.5 Hz, 1H), 7.22 (t, J = 6.3 Hz, 1H), 6.57 (d, J = 7.1 Hz, 1H); ^{13}C NMR (100 MHz, DMSO-d_6) δ 160.6, 136.8, 135.1, 129.7, 128.8, 128.7, 127.5, 119.0, 104.1; MS (ESI) m/z: $[M + H]^+$ Calcd for C_9H_7BrNO 223.97; Found 224.00; HRMS (ESI-TOF) m/z: $[M + H]^+$ Calcd for C_9H_7BrNO 223.9706; Found 223.9707.

7-Methylisoquinolin-1(2*H*)-one (7d)[8]

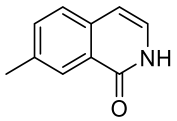

Starting materials: 5-Methyl-*N*-(methylsulfonyl)-2-((triisopropylsilyl)ethynyl)benzamide (**6d**) (0.14 g, 0.36 mmol, 1.0 equiv.) and TBAF (1M in THF) (1.1 mL, 1.08 mmol, 3.0 equiv.) in 2 mL of THF using the same method for **7a**.

Purification conditions: Combiflash ISCO column using hexane/ethyl acetate as mobile phase (%elution of desired compound= 40% ethyl acetate in hexane).

Yield: 0.035 g (62%); pale-yellow oil.

^{1}H NMR (400 MHz, DMSO-d_6) δ 11.14 (s, 1H), 7.98 (s, 1H), 7.56 – 7.50 (m, 2H), 7.09 (dd, J = 7.3, 4.3 Hz, 1H), 6.50 (d, J = 7.1 Hz, 1H), 2.42 (s, 3H); ^{13}C NMR (100 MHz, DMSO-d_6) δ 161.7, 135.8, 135.5, 133.6, 127.9, 126.1, 126.1, 126.0, 104.5, 21.0; MS (ESI) m/z: $[M + H]^+$ Calcd for $C_{10}H_{10}NO$ 160.08; Found 160.10; HRMS (ESI) m/z: $[M + H]^+$ Calcd for $C_{10}H_{10}NO$ 160.0757; Found 160.0759.

7-Methoxyisoquinolin-1(2*H*)-one (7e)[9]

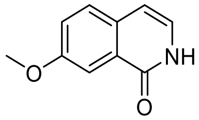

Starting materials: 5-Methoxy-*N*-(methylsulfonyl)-2-((triisopropylsilyl)ethynyl)benzamide (**6e**) (0.11 g, 0.26 mmol, 1.0 equiv.) and TBAF (1M in THF) (0.8 mL, 0.78 mmol, 3.0 equiv.) in 2 mL of THF using the same method for **7a**.

Purification conditions: Combiflash ISCO column using hexane/ethyl acetate as mobile phase (%elution of desired compound= 30% ethyl acetate in hexane).

Yield: 0.028 g (65%); pale-yellow oil.

^{1}H NMR (400 MHz, DMSO-d_6) δ 11.19 (s, 1H), 7.60 (dd, J = 5.8, 2.9 Hz, 2H), 7.31 (dd, J = 8.7, 2.8 Hz, 1H), 7.04 (t, J = 6.0 Hz, 1H), 6.51 (d, J = 7.1 Hz, 1H), 3.85 (s, 3H); ^{13}C NMR (100 MHz, DMSO-d_6) δ 161.5, 157.9, 131.8, 127.9, 127.2, 126.4, 122.1, 107.1, 104.5, 55.3; MS (ESI) m/z: $[M + H]^+$ Calcd for $C_{10}H_{10}NO_2$ 176.07; Found 176.10; HRMS (ESI-TOF) m/z: $[M + H]^+$ Calcd for $C_{10}H_{10}NO_2$ 176.0707; Found 176.0708.

6-Chloroisoquinolin-1(2*H*)-one (7f)[8]

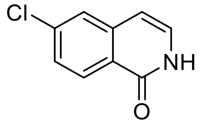

Starting materials: 4-Chloro-*N*-(methylsulfonyl)-2-((triisopropylsilyl)ethynyl)benzamide (**6g**) (0.11 g, 0.26 mmol, 1.0 equiv.) and TBAF (1M in THF) (0.81 mL, 0.81 mmol, 3.0 equiv.) in 2 mL of THF using the same method for **7a**.

Purification conditions: Combiflash ISCO column using hexane/ethyl acetate as mobile phase (%elution of desired compound= 20% ethyl acetate in hexane).

Yield: 0.03 g (61%); colorless oil.

^{1}H NMR (400 MHz, DMSO-d_6) δ 11.36 (s, 1H), 8.16 (d, J = 8.6 Hz, 1H), 7.79 (d, J = 2.1 Hz, 1H), 7.49 (dd, J = 8.6, 2.1 Hz, 1H), 7.23 (t, J = 6.4 Hz, 1H), 6.53 (d, J = 7.1 Hz, 1H); ^{13}C NMR (100 MHz, DMSO-d_6) δ 161.2, 139.4, 137.2, 130.6, 129.0, 126.4, 125.3, 124.6, 103.7; MS (ESI) m/z: $[M + H]^+$ Calcd for C_9H_7ClNO 180.02; Found 180.00; HRMS (ESI) m/z: $[M + H]^+$ Calcd for C_9H_7ClNO 180.0211; Found 180.0211.

6-Nitroisoquinolin-1(2*H*)-one (7g)[10]

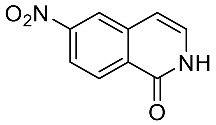

Starting materials: *N*-(Methylsulfonyl)-4-nitro-2-((triisopropylsilyl)ethynyl)benzamide (**6h**) (0.12 g, 0.31 mmol, 1.0 equiv.) and TBAF (1M in THF) (0.93 mL, 0.93 mmol, 3.0 equiv.) in 2 mL of THF using the same method for **7a**.

Purification conditions: Combiflash ISCO column using hexane/ethyl acetate as mobile phase (%elution of desired compound= 40% ethyl acetate in hexane).

Yield: 0.042 g (71%); pale-yellow oil.

^{1}H NMR (400 MHz, DMSO-d_6) δ 11.65 (s, 1H), 8.61 (d, J = 2.3 Hz, 1H), 8.37 (d, J = 8.8 Hz, 1H), 8.17 (dd, J = 8.9, 2.3 Hz, 1H), 7.35 (t, J = 6.3 Hz, 1H), 6.80 (d, J = 7.1 Hz, 1H); ^{13}C NMR (100 MHz, DMSO-d_6) δ 160.8, 149.7, 138.6, 131.3, 129.6, 129.0, 121.7, 119.7, 104.6; MS (ESI) m/z: $[M + H]^+$ Calcd for $C_9H_7N_2O_3$ 191.05; Found 191.00; HRMS (ESI-TOF) m/z: $[M + H]^+$ Calcd for $C_9H_7N_2O_3$ 191.0452; Found 191.0451.

NMR and HRMS Data

Ethyl 3-(2-carbamoylphenyl)propiolate (2a)

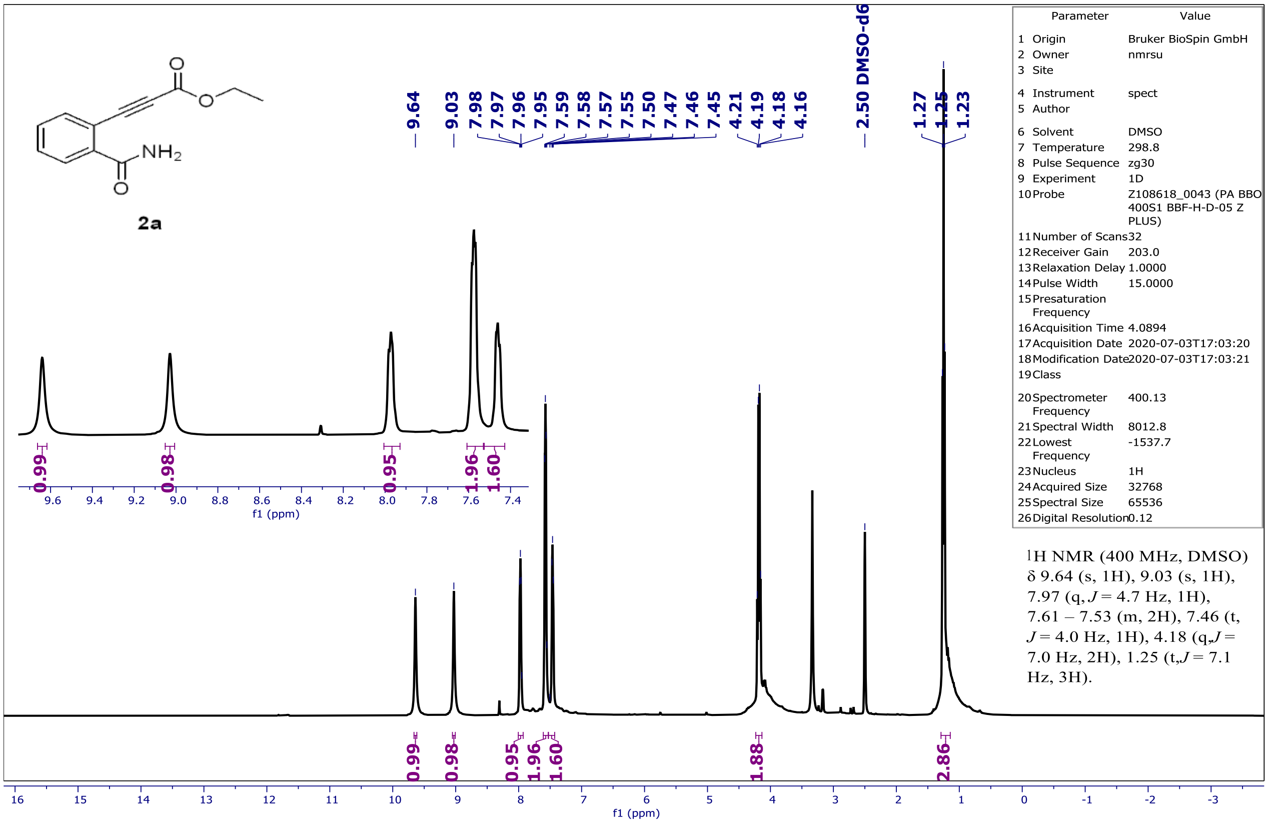

^{1}H NMR (400 MHz, DMSO) δ 9.64 (s, 1H), 9.03 (s, 1H), 7.97 (q, J = 4.7 Hz, 1H), 7.61 – 7.53 (m, 2H), 7.46 (t, J = 4.0 Hz, 1H), 4.18 (q, J = 7.0 Hz, 2H), 1.25 (t, J = 7.1 Hz, 3H).

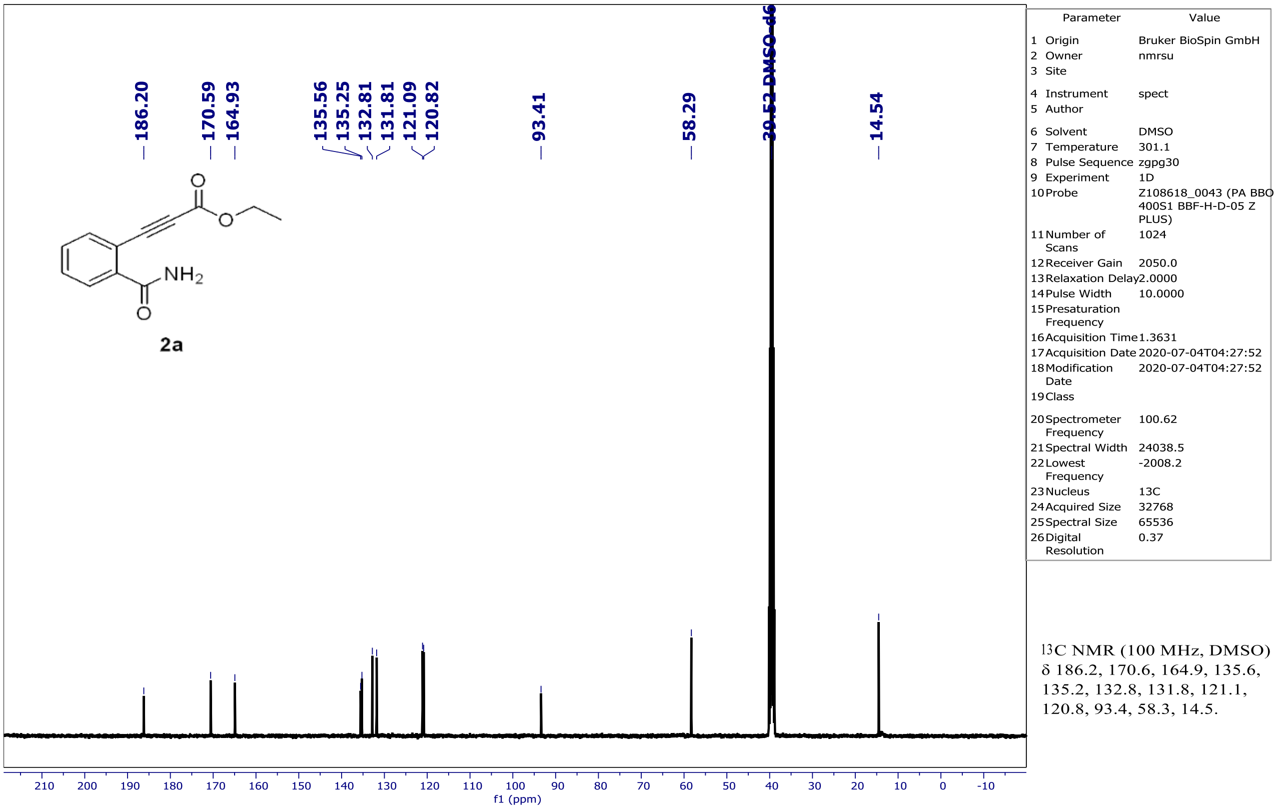

^{13}C NMR (100 MHz, DMSO) δ 186.2, 170.6, 164.9, 135.6, 135.2, 132.8, 131.8, 121.1, 120.8, 93.4, 58.3, 14.5.

Ethyl 3-(2-carbamoylphenyl)propiolate (2a)

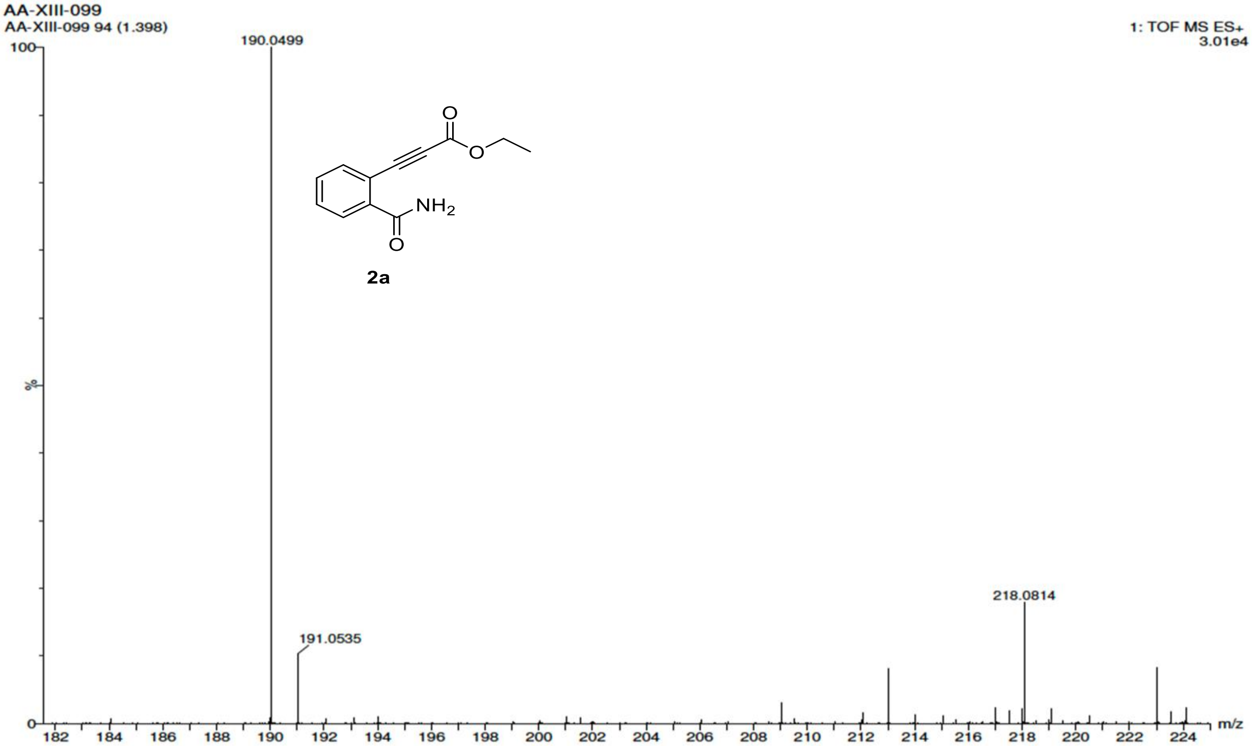

HRMS (ESI) *m/z* calcd for $C_{12}H_{11}NO_3$ $[M + H]^+$ 218.0812; found 218.0814.

Ethyl 3-(2-carbamoyl-4-chlorophenyl)propiolate (2b)

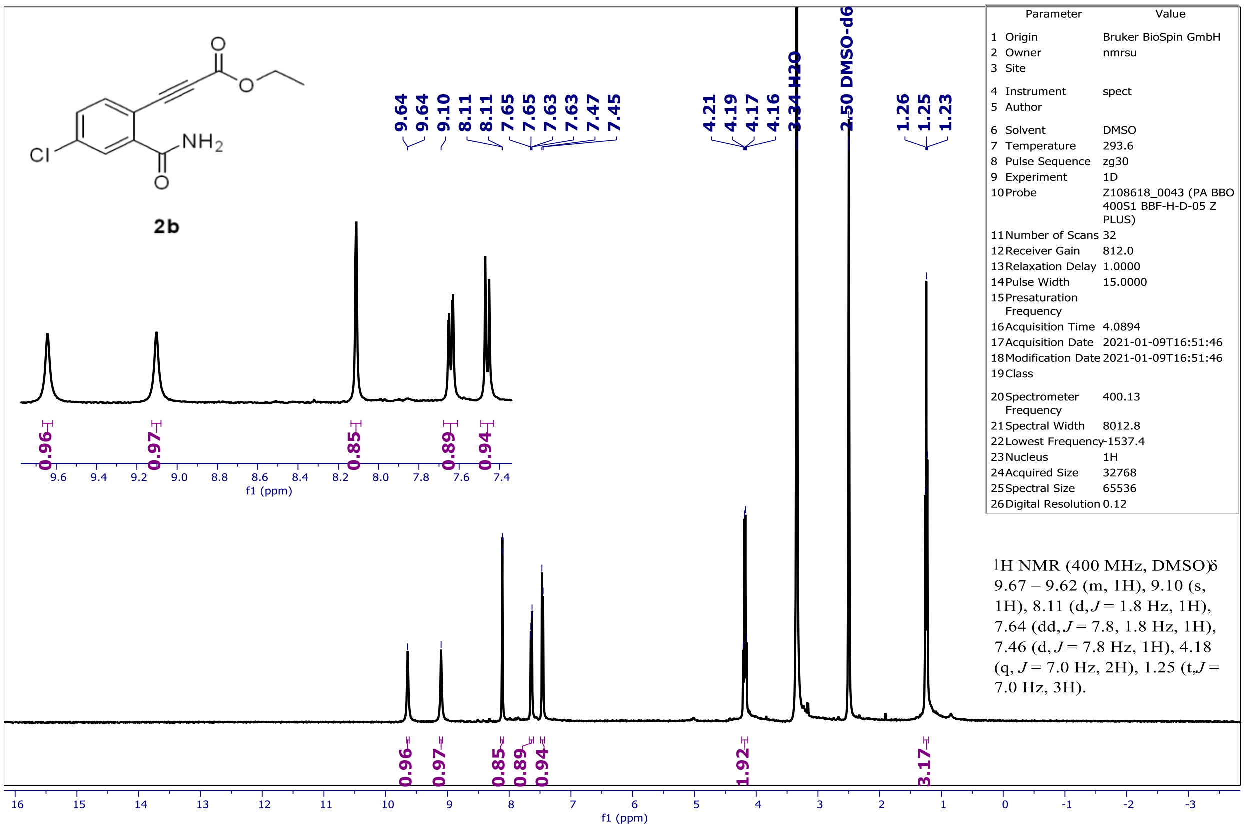

^{1}H NMR (400 MHz, DMSO) δ 9.67 – 9.62 (m, 1H), 9.10 (s, 1H), 8.11 (d, *J* = 1.8 Hz, 1H), 7.64 (dd, *J* = 7.8, 1.8 Hz, 1H), 7.46 (d, *J* = 7.8 Hz, 1H), 4.18 (q, *J* = 7.0 Hz, 2H), 1.25 (t, *J* = 7.0 Hz, 3H).

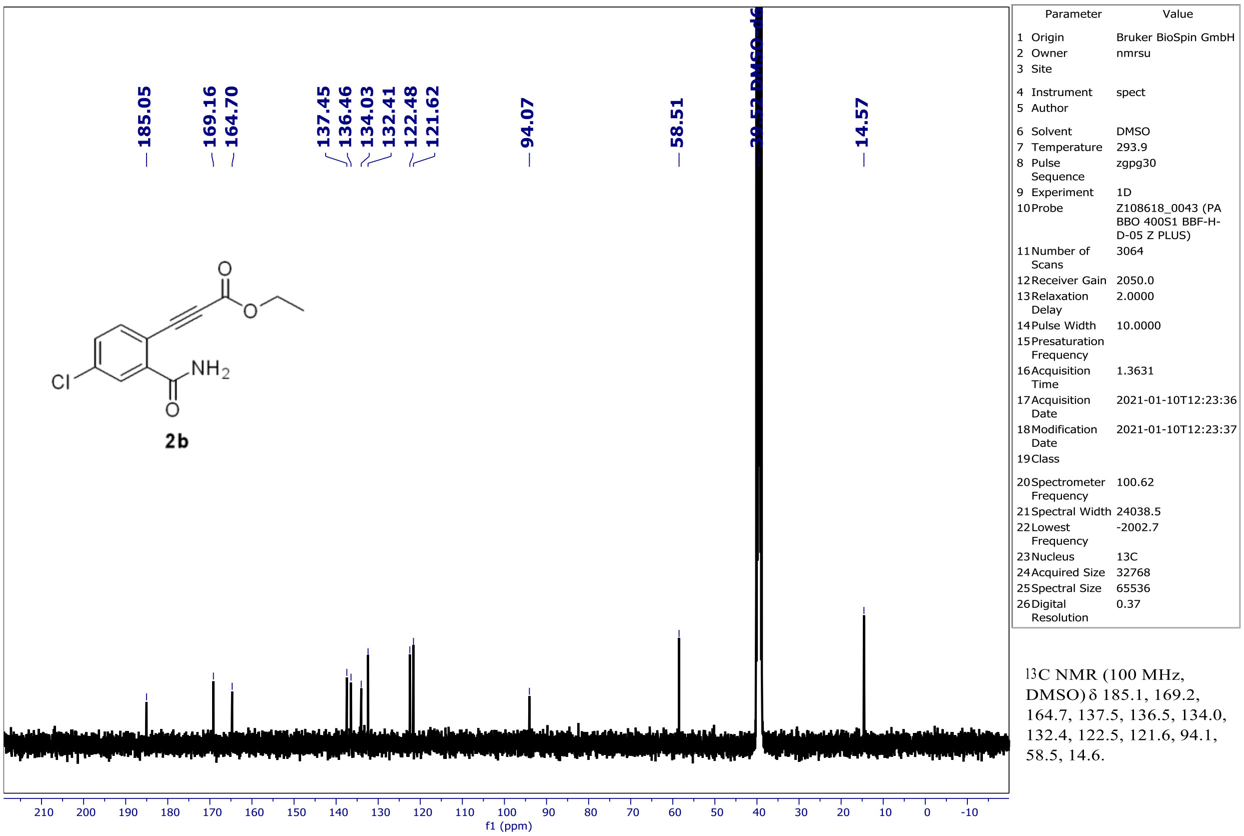

^{13}C NMR (100 MHz, DMSO) δ 185.1, 169.2, 164.7, 137.5, 136.5, 134.0, 132.4, 122.5, 121.6, 94.1, 58.5, 14.6.

Ethyl 3-(2-carbamoyl-4-chlorophenyl)propiolate (2b)

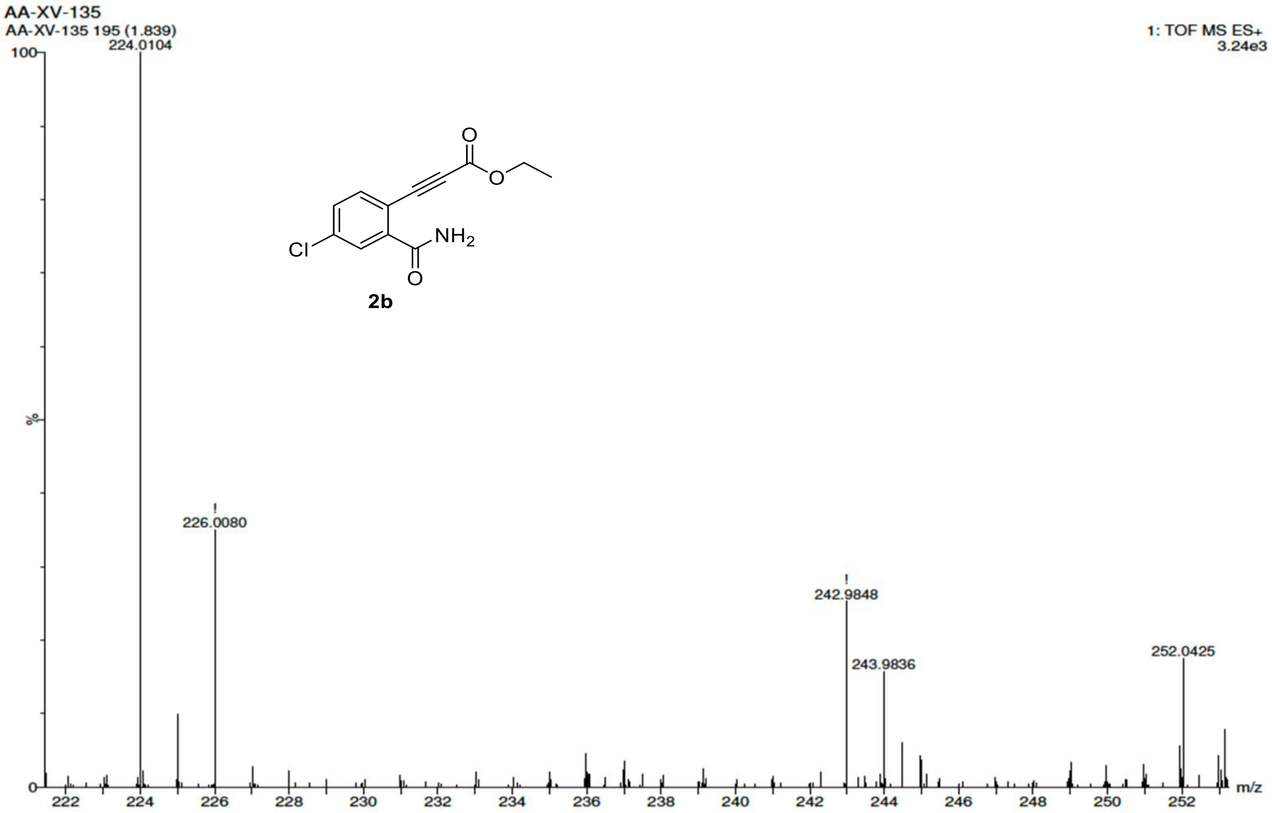

HRMS (ESI) *m/z* calcd for $C_{12}H_{10}ClNO_3$ $[M + H]^+$ 252.0422; found 252.0425.

Ethyl 3-(4-bromo-2-carbamoylphenyl)propiolate (2c)

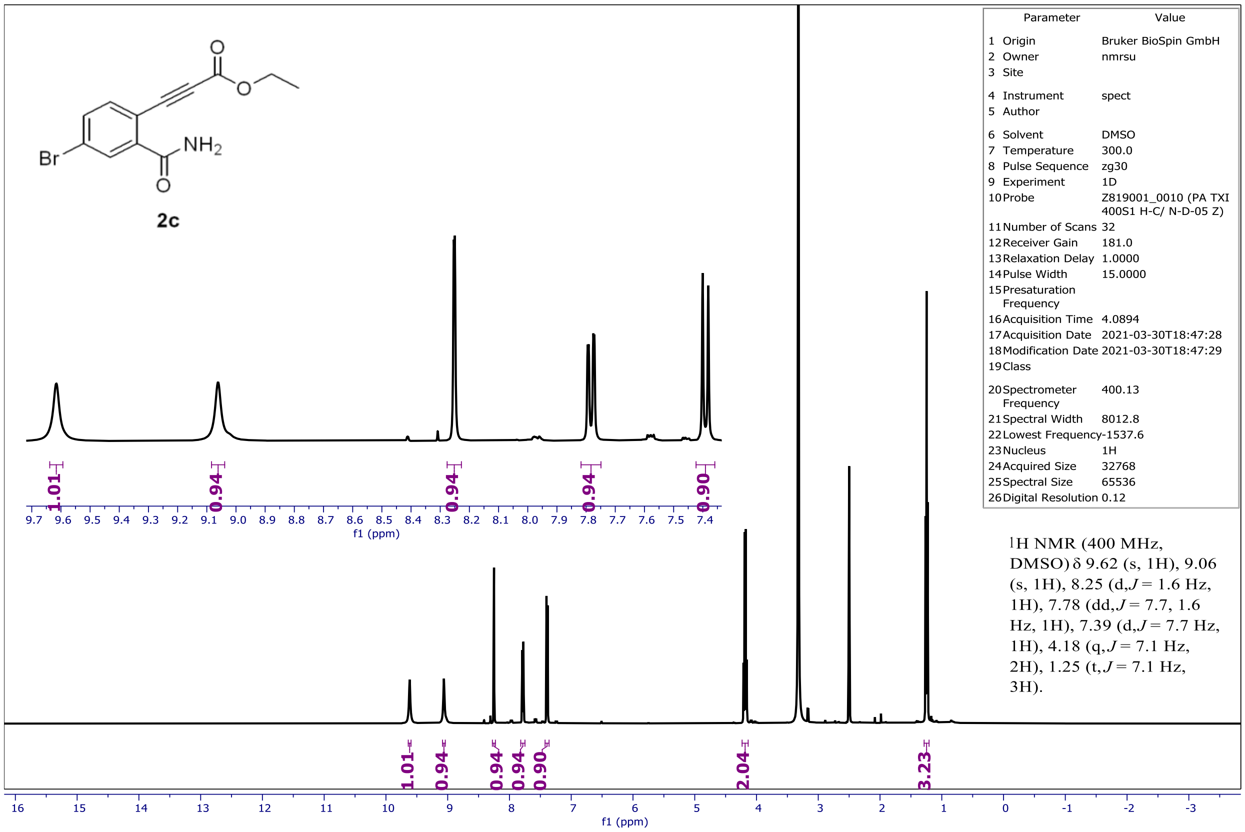

Parameter		Value
1	Origin	Bruker BioSpin GmbH
2	Owner	nmrsu
3	Site	
4	Instrument	spect
5	Author	
6	Solvent	DMSO
7	Temperature	300.0
8	Pulse Sequence	zg30
9	Experiment	1D
10	Probe	Z819001_0010 (PA TXI 400S1 H-C/ N-D-05 Z)
11	Number of Scans	32
12	Receiver Gain	181.0
13	Relaxation Delay	1.0000
14	Pulse Width	15.0000
15	Presaturation Frequency	
16	Acquisition Time	4.0894
17	Acquisition Date	2021-03-30T18:47:28
18	Modification Date	2021-03-30T18:47:29
19	Class	
20	Spectrometer Frequency	400.13
21	Spectral Width	8012.8
22	Lowest Frequency	-1537.6
23	Nucleus	1H
24	Acquired Size	32768
25	Spectral Size	65536
26	Digital Resolution	0.12

^{1}H NMR (400 MHz, DMSO) δ 9.62 (s, 1H), 9.06 (s, 1H), 8.25 (d, J = 1.6 Hz, 1H), 7.78 (dd, J = 7.7, 1.6 Hz, 1H), 7.39 (d, J = 7.7 Hz, 1H), 4.18 (q, J = 7.1 Hz, 2H), 1.25 (t, J = 7.1 Hz, 3H).

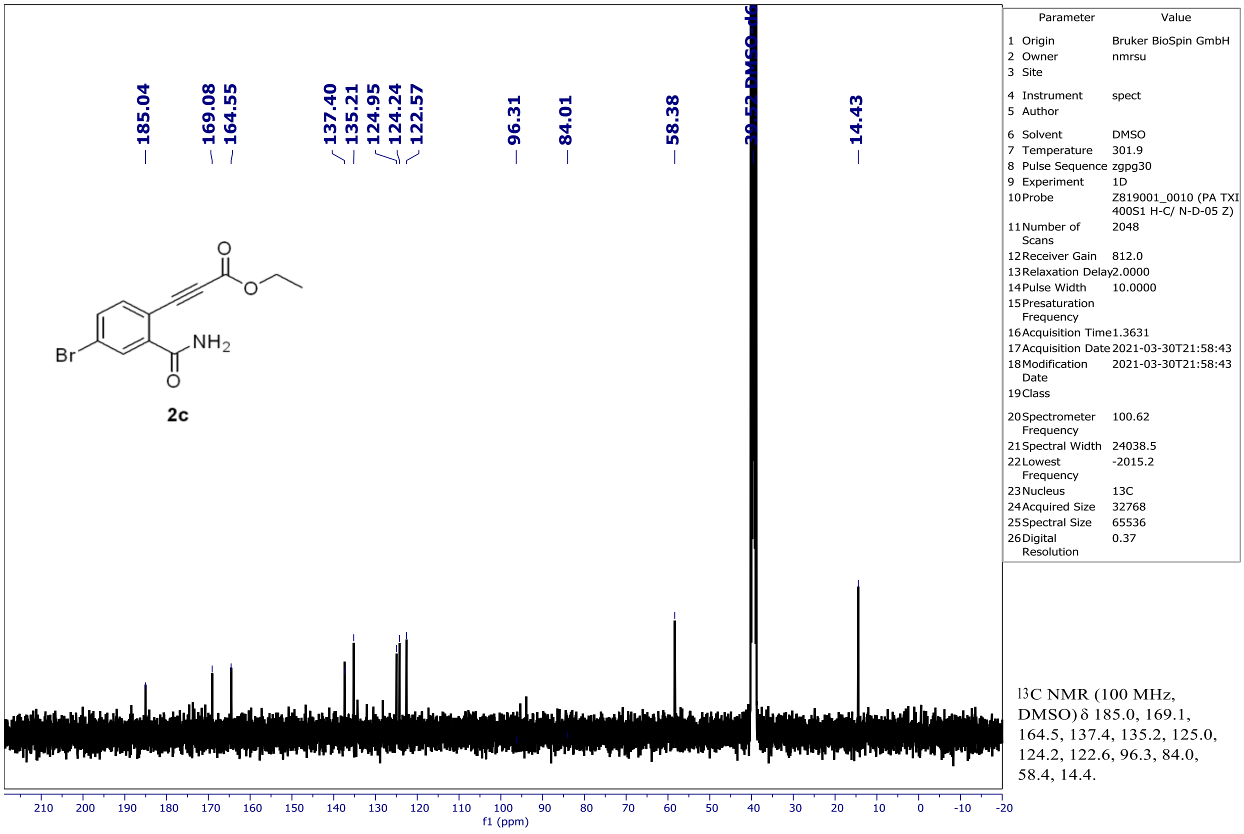

Parameter		Value
1	Origin	Bruker BioSpin GmbH
2	Owner	nmrsu
3	Site	
4	Instrument	spect
5	Author	
6	Solvent	DMSO
7	Temperature	301.9
8	Pulse Sequence	zgpg30
9	Experiment	1D
10	Probe	Z819001_0010 (PA TXI 400S1 H-C/ N-D-05 Z)
11	Number of Scans	2048
12	Receiver Gain	812.0
13	Relaxation Delay	2.0000
14	Pulse Width	10.0000
15	Presaturation Frequency	
16	Acquisition Time	1.3631
17	Acquisition Date	2021-03-30T21:58:43
18	Modification Date	2021-03-30T21:58:43
19	Class	
20	Spectrometer Frequency	100.62
21	Spectral Width	24038.5
22	Lowest Frequency	-2015.2
23	Nucleus	13C
24	Acquired Size	32768
25	Spectral Size	65536
26	Digital Resolution	0.37

^{13}C NMR (100 MHz, DMSO) δ 185.0, 169.1, 164.5, 137.4, 135.2, 125.0, 124.2, 122.6, 96.3, 84.0, 58.4, 14.4.

Ethyl 3-(4-bromo-2-carbamoylphenyl)propiolate (2c)

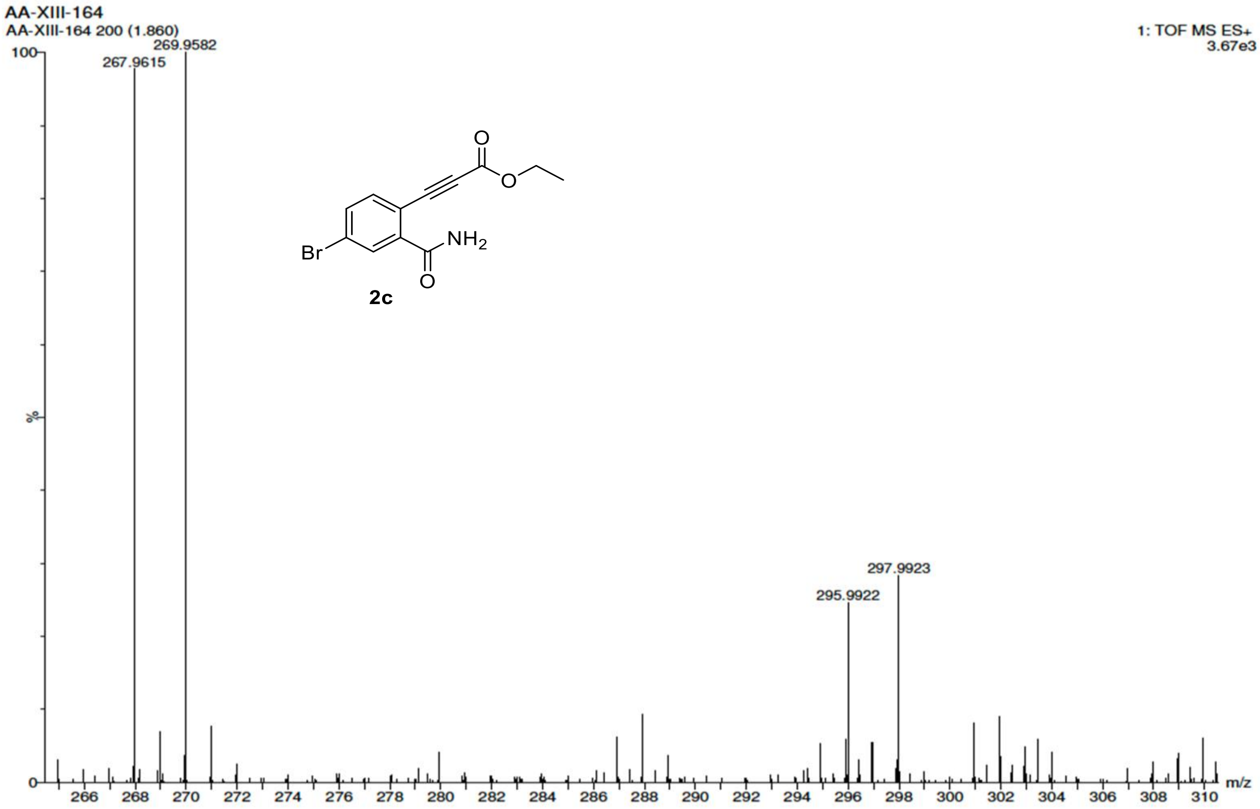

HRMS (ESI) *m/z* calcd for $C_{12}H_{10}BrNO_3$ $[M + H]^+$ 295.9917; found 295.9922.

Ethyl 3-(2-carbamoyl-4-nitrophenyl)propiolate (2d)

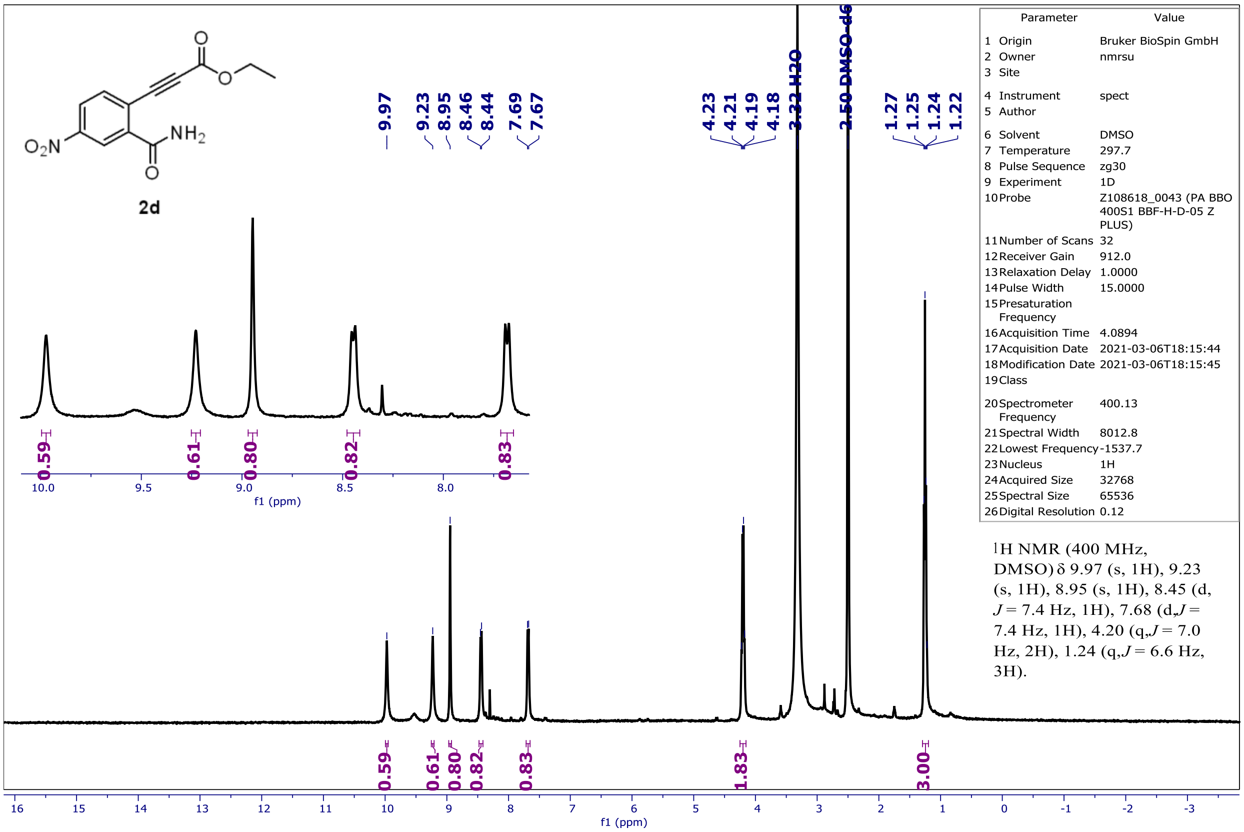

^{1}H NMR (400 MHz, DMSO) δ 9.97 (s, 1H), 9.23 (s, 1H), 8.95 (s, 1H), 8.45 (d, J = 7.4 Hz, 1H), 7.68 (d, J = 7.4 Hz, 1H), 4.20 (q, J = 7.0 Hz, 2H), 1.24 (q, J = 6.6 Hz, 3H).

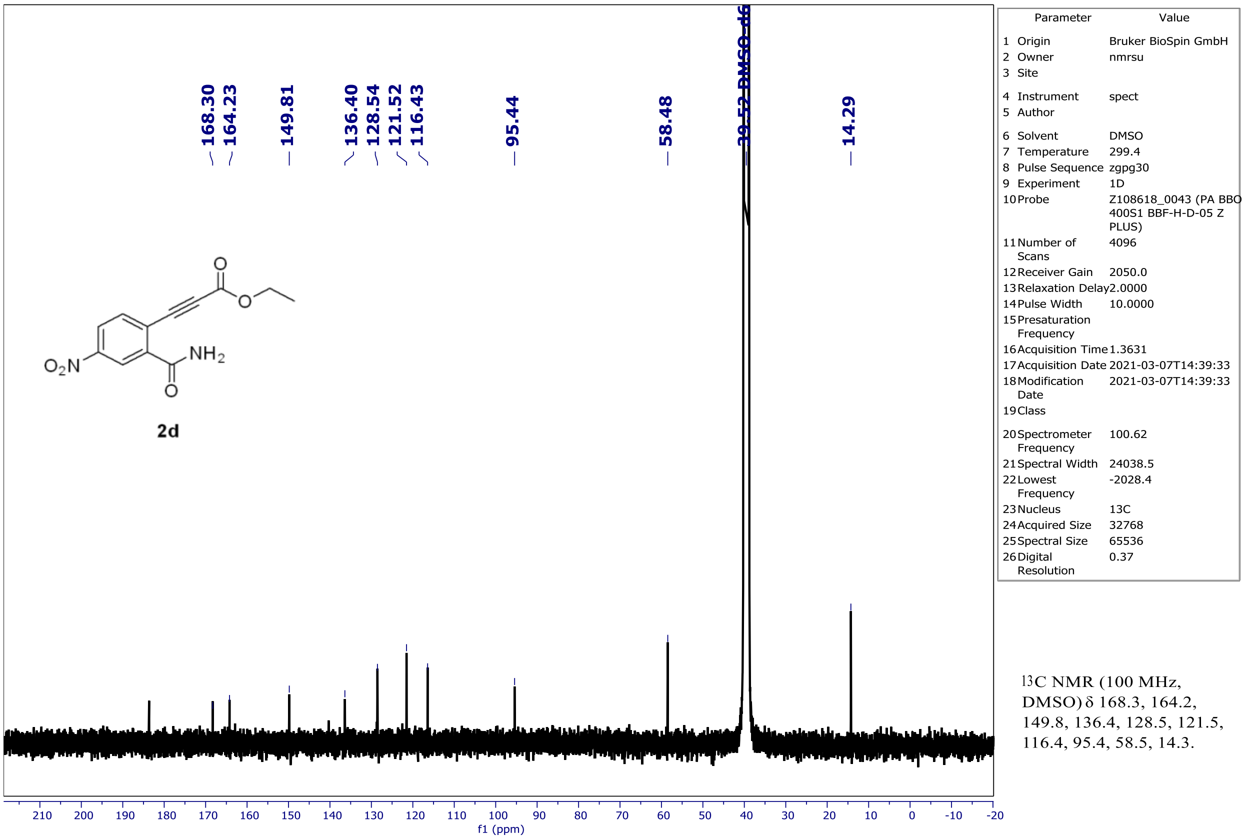

^{13}C NMR (100 MHz, DMSO) δ 168.3, 164.2, 149.8, 136.4, 128.5, 121.5, 116.4, 95.4, 58.5, 14.3.

Ethyl 3-(2-carbamoyl-4-nitrophenyl)propiolate (2d)

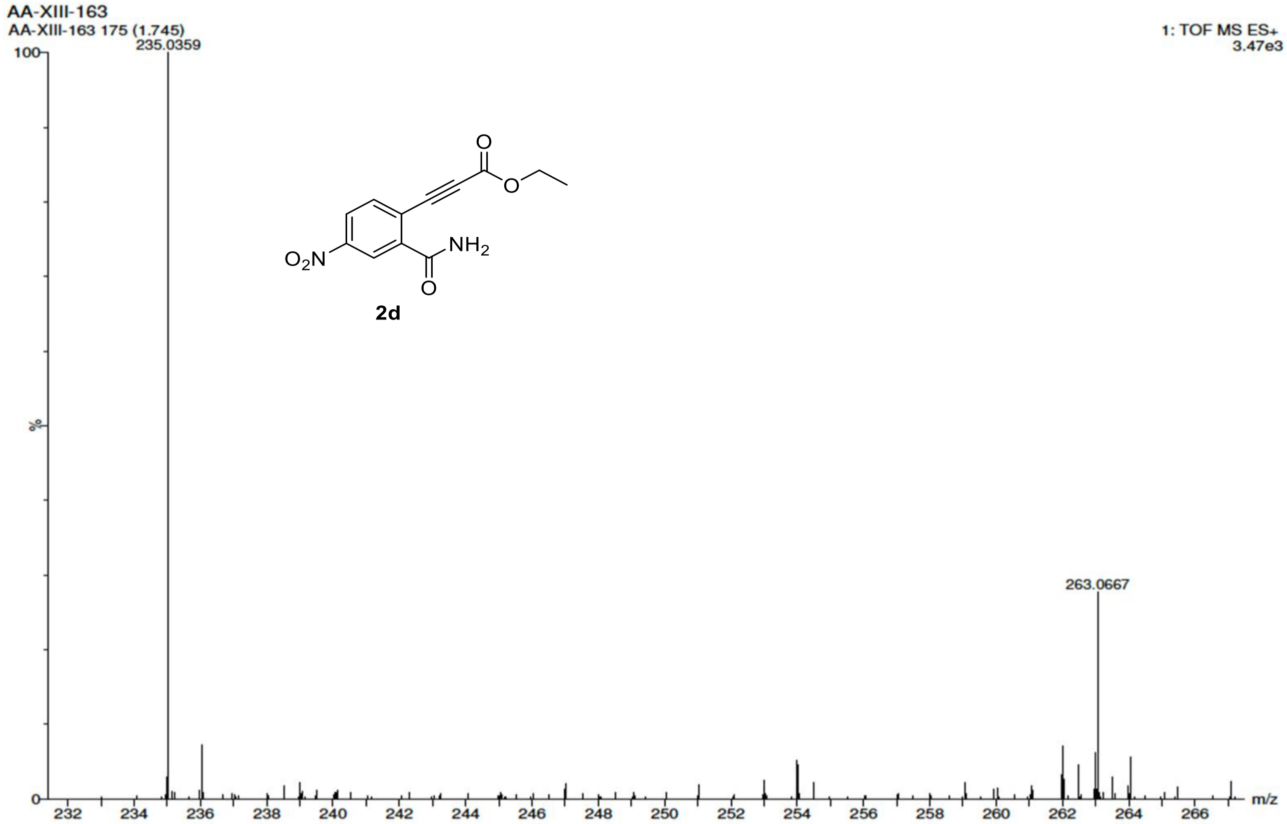

HRMS (ESI) *m/z* calcd for $C_{12}H_{10}BrNO_3$ $[M + H]^+$ 263.0662; found 263.0667.

Ethyl 3-(2-carbamoyl-4-methylphenyl)propiolate (2e)

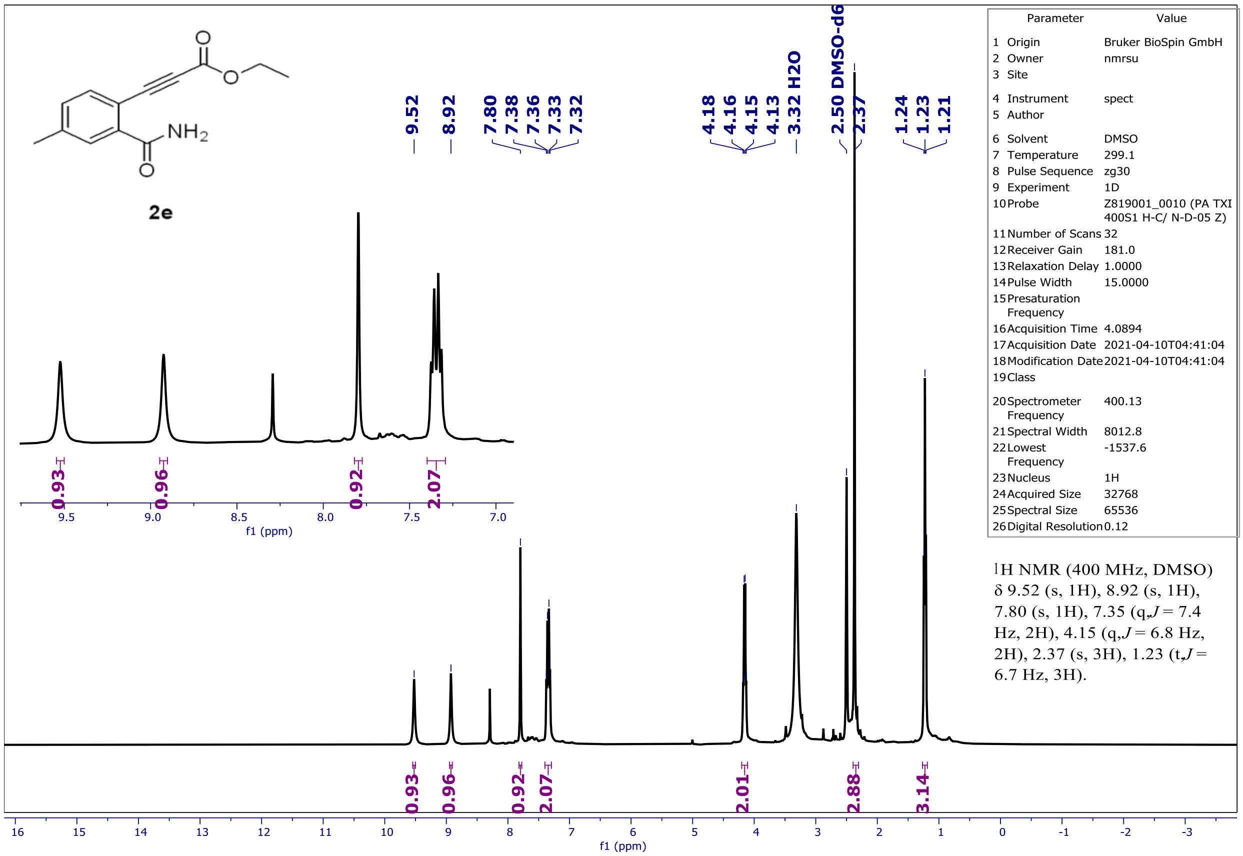

Parameter		Value
1	Origin	Bruker BioSpin GmbH
2	Owner	nmrsu
3	Site	
4	Instrument	spect
5	Author	
6	Solvent	DMSO
7	Temperature	299.1
8	Pulse Sequence	zg30
9	Experiment	1D
10	Probe	Z819001_0010 (PA TXI 400S1 H-C/ N-D-05 Z)
11	Number of Scans	32
12	Receiver Gain	181.0
13	Relaxation Delay	1.0000
14	Pulse Width	15.0000
15	Presaturation Frequency	
16	Acquisition Time	4.0894
17	Acquisition Date	2021-04-10T04:41:04
18	Modification Date	2021-04-10T04:41:04
19	Class	
20	Spectrometer Frequency	400.13
21	Spectral Width	8012.8
22	Lowest Frequency	-1537.6
23	Nucleus	1H
24	Acquired Size	32768
25	Spectral Size	65536
26	Digital Resolution	0.12

^{1}H NMR (400 MHz, DMSO) δ 9.52 (s, 1H), 8.92 (s, 1H), 7.80 (s, 1H), 7.35 (q, *J* = 7.4 Hz, 2H), 4.15 (q, *J* = 6.8 Hz, 2H), 2.37 (s, 3H), 1.23 (t, *J* = 6.7 Hz, 3H).

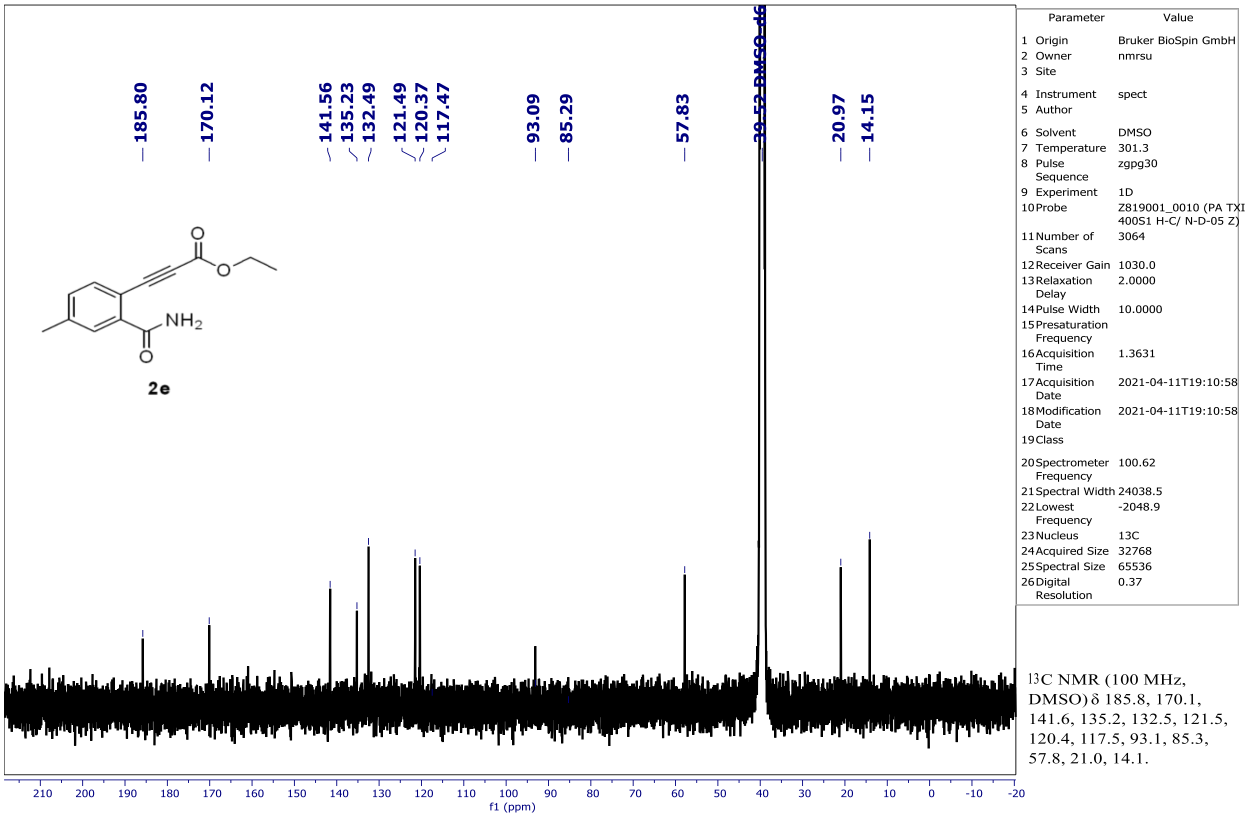

Parameter		Value
1	Origin	Bruker BioSpin GmbH
2	Owner	nmrsu
3	Site	
4	Instrument	spect
5	Author	
6	Solvent	DMSO
7	Temperature	301.3
8	Pulse Sequence	zgpg30
9	Experiment	1D
10	Probe	Z819001_0010 (PA TXI 400S1 H-C/ N-D-05 Z)
11	Number of Scans	3064
12	Receiver Gain	1030.0
13	Relaxation Delay	2.0000
14	Pulse Width	10.0000
15	Presaturation Frequency	
16	Acquisition Time	1.3631
17	Acquisition Date	2021-04-11T19:10:58
18	Modification Date	2021-04-11T19:10:58
19	Class	
20	Spectrometer Frequency	100.62
21	Spectral Width	24038.5
22	Lowest Frequency	-2048.9
23	Nucleus	13C
24	Acquired Size	32768
25	Spectral Size	65536
26	Digital Resolution	0.37

^{13}C NMR (100 MHz, DMSO) δ 185.8, 170.1, 141.6, 135.2, 132.5, 121.5, 120.4, 117.5, 93.1, 85.3, 57.8, 21.0, 14.1.

Ethyl 3-(2-carbamoyl-4-methylphenyl)propiolate (2e)

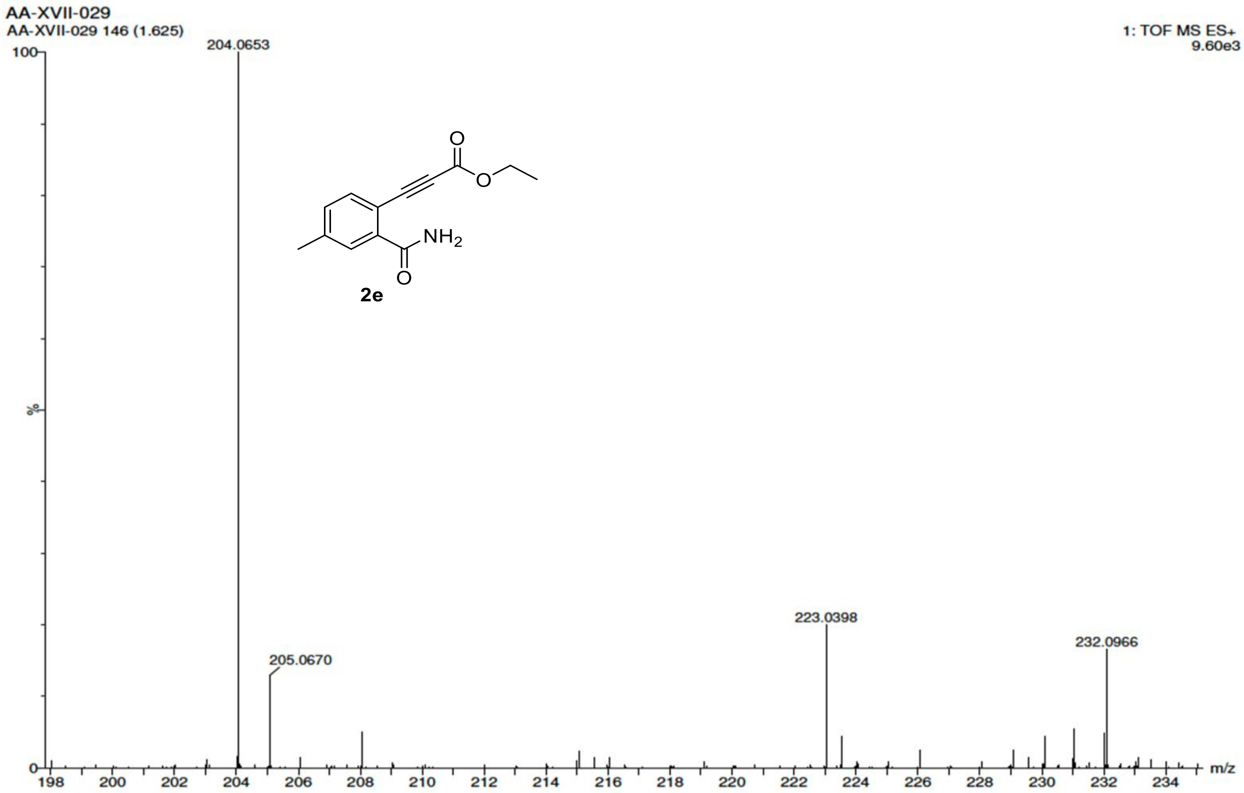

HRMS (ESI) *m/z* calcd for $C_{13}H_{13}NO_3$ $[M + H]^+$ 232.0968; found 232.0966.

2-(Phenylethynyl)benzamide (2f)

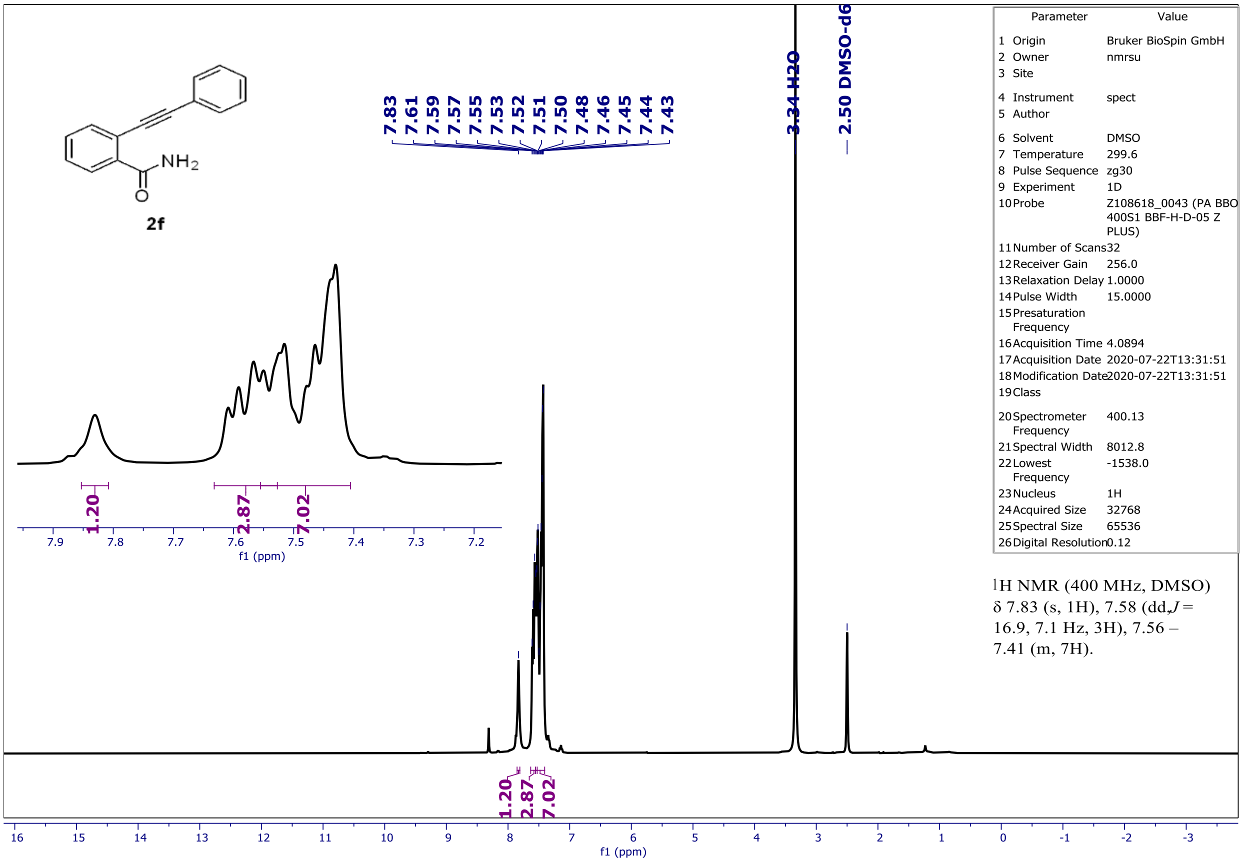

1H NMR (400 MHz, DMSO) δ 7.83 (s, 1H), 7.58 (dd, *J* = 16.9, 7.1 Hz, 3H), 7.56 – 7.41 (m, 7H).

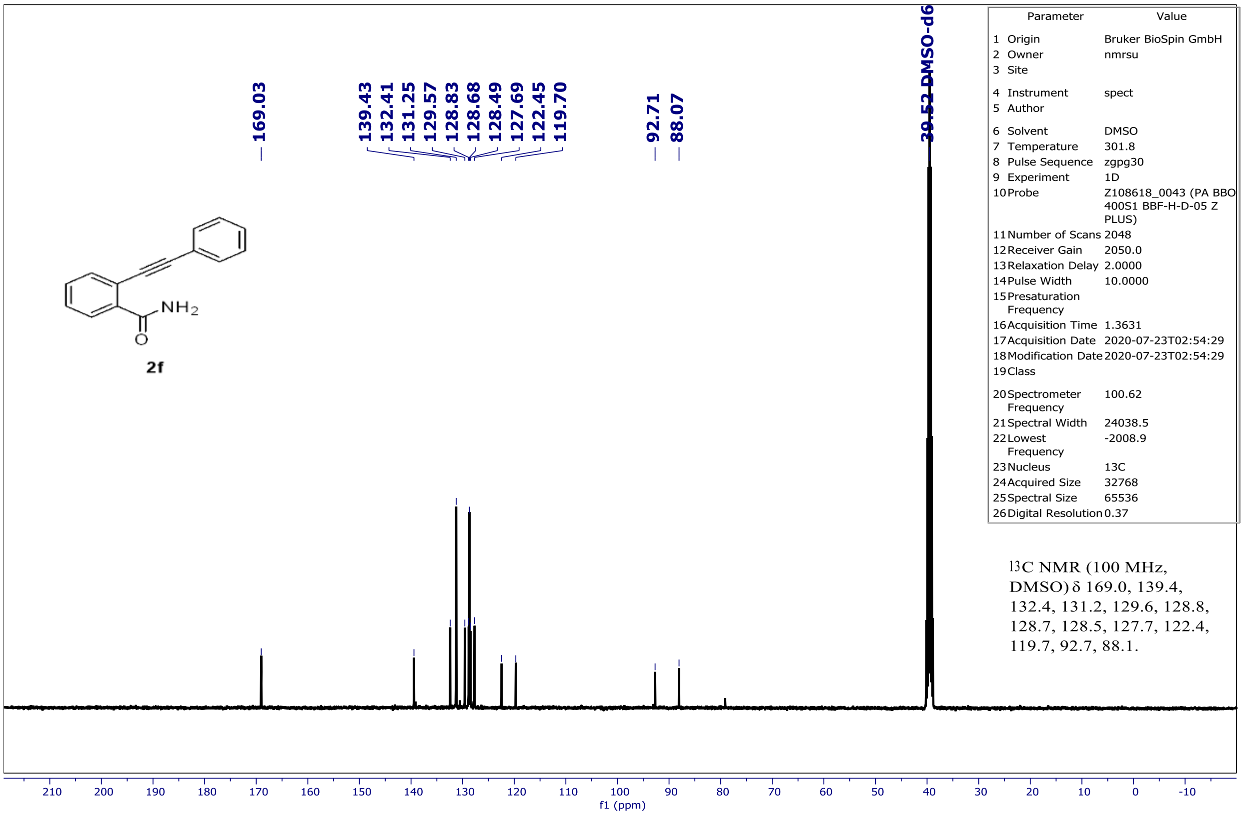

13C NMR (100 MHz, DMSO) δ 169.0, 139.4, 132.4, 131.2, 129.6, 128.8, 128.7, 128.5, 127.7, 122.4, 119.7, 92.7, 88.1.

2-(Phenylethynyl)benzamide (2f)

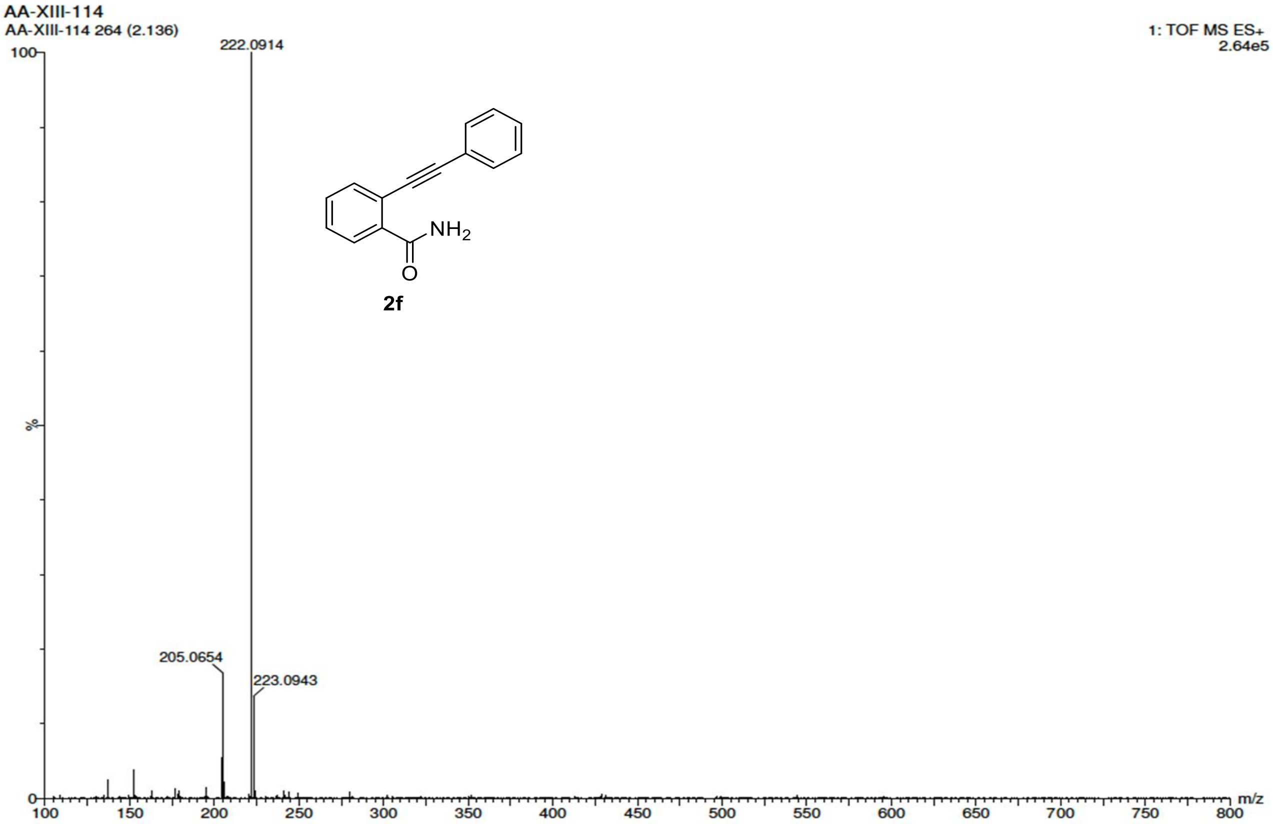

HRMS (ESI) *m/z* calcd for $C_{15}H_{11}NO$ $[M + H]^+$ 222.0913; found 222.0914.

5-Bromo-2-(phenylethynyl)benzamide (2g)

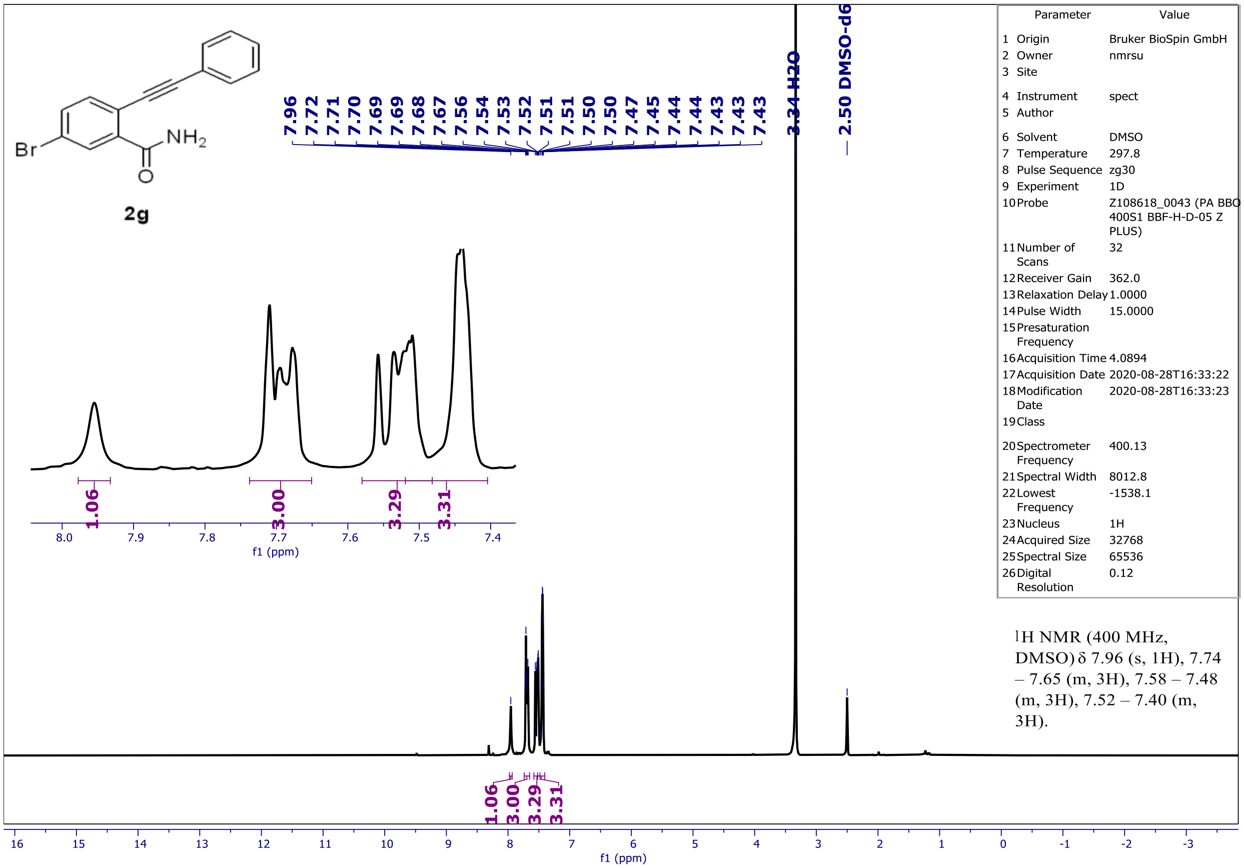

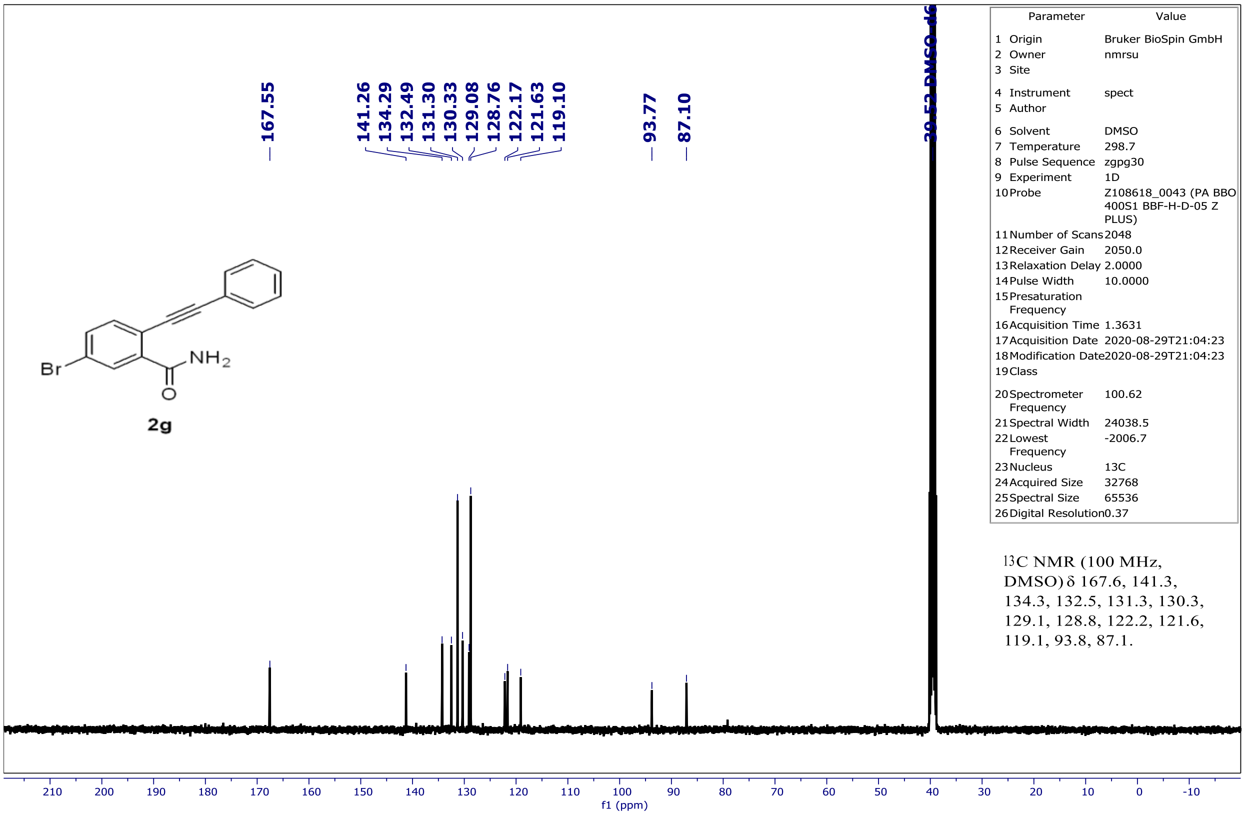

5-Bromo-2-(phenylethynyl)benzamide (2g)

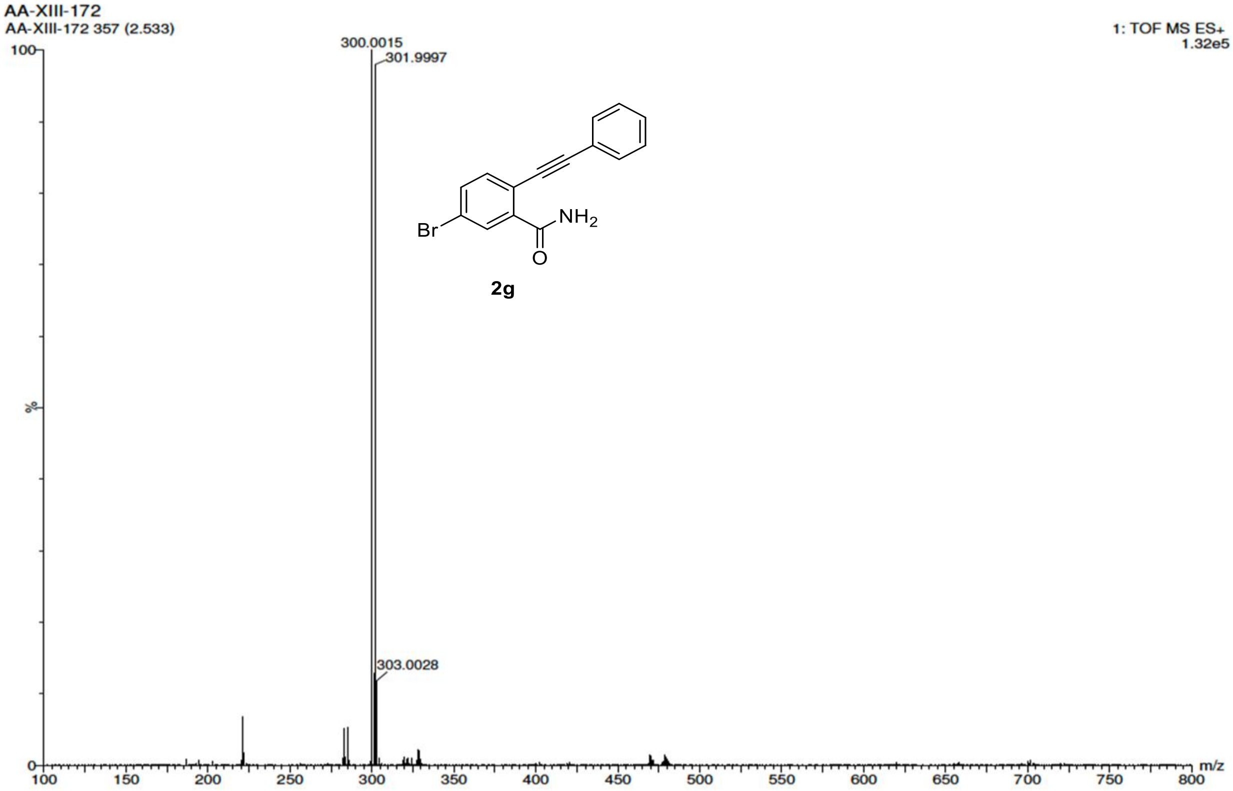

HRMS (ESI) *m/z* calcd for $C_{15}H_{11}NO$ $[M + H]^+$ 300.0019; found 300.0015.

2-((Triisopropylsilyl)ethynyl)benzamide (2h)

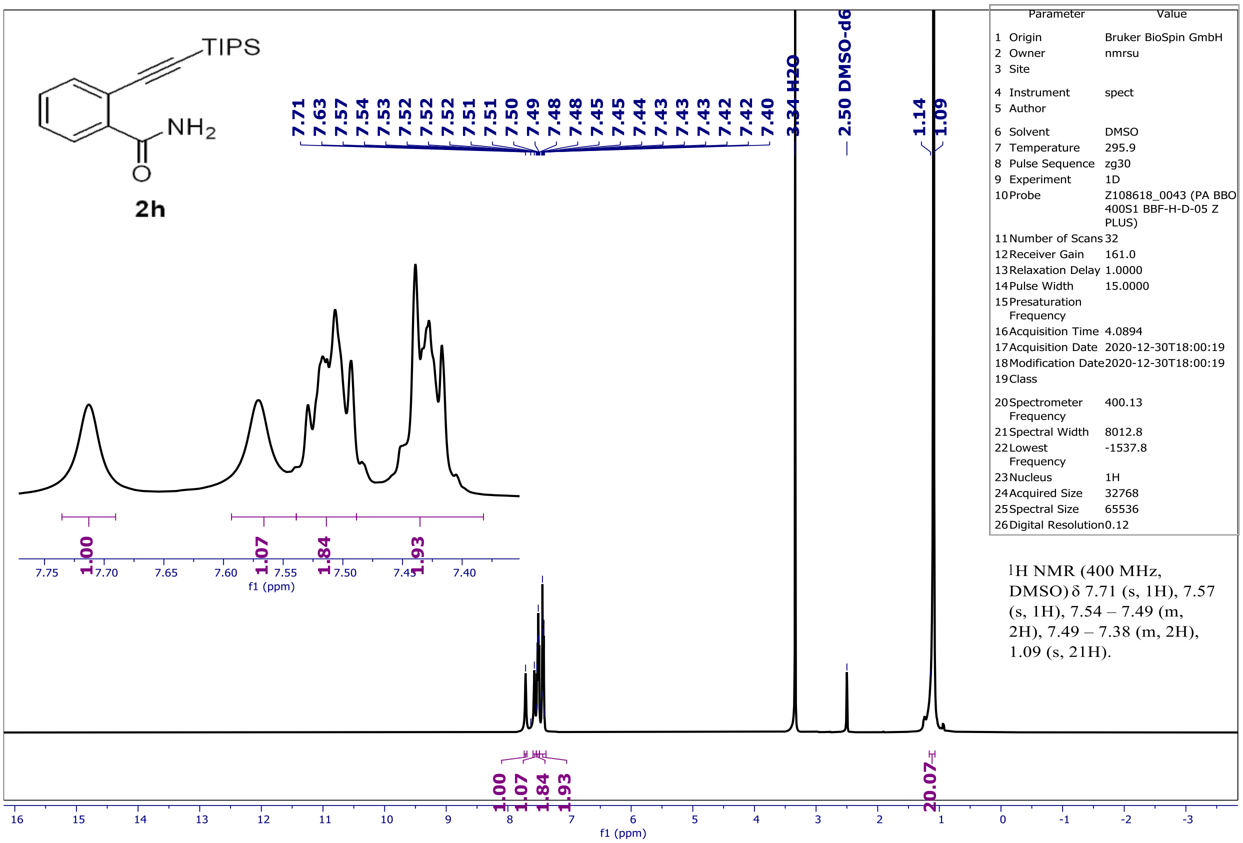

1H NMR (400 MHz, DMSO) δ 7.71 (s, 1H), 7.57 (s, 1H), 7.54 – 7.49 (m, 2H), 7.49 – 7.38 (m, 2H), 1.09 (s, 21H).

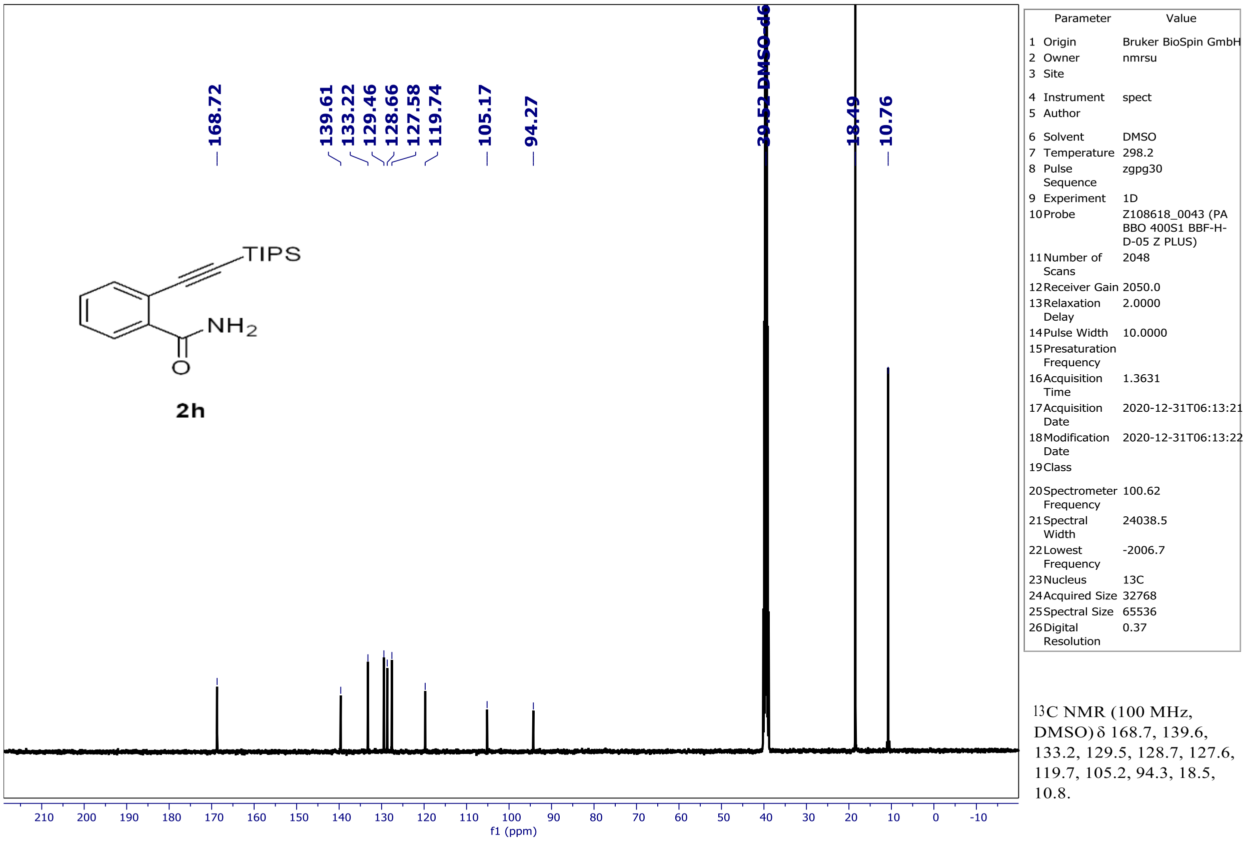

13C NMR (100 MHz, DMSO) δ 168.7, 139.6, 133.2, 129.5, 128.7, 127.6, 119.7, 105.2, 94.3, 18.5, 10.8.

2-((Triisopropylsilyl)ethynyl)benzamide (2h)

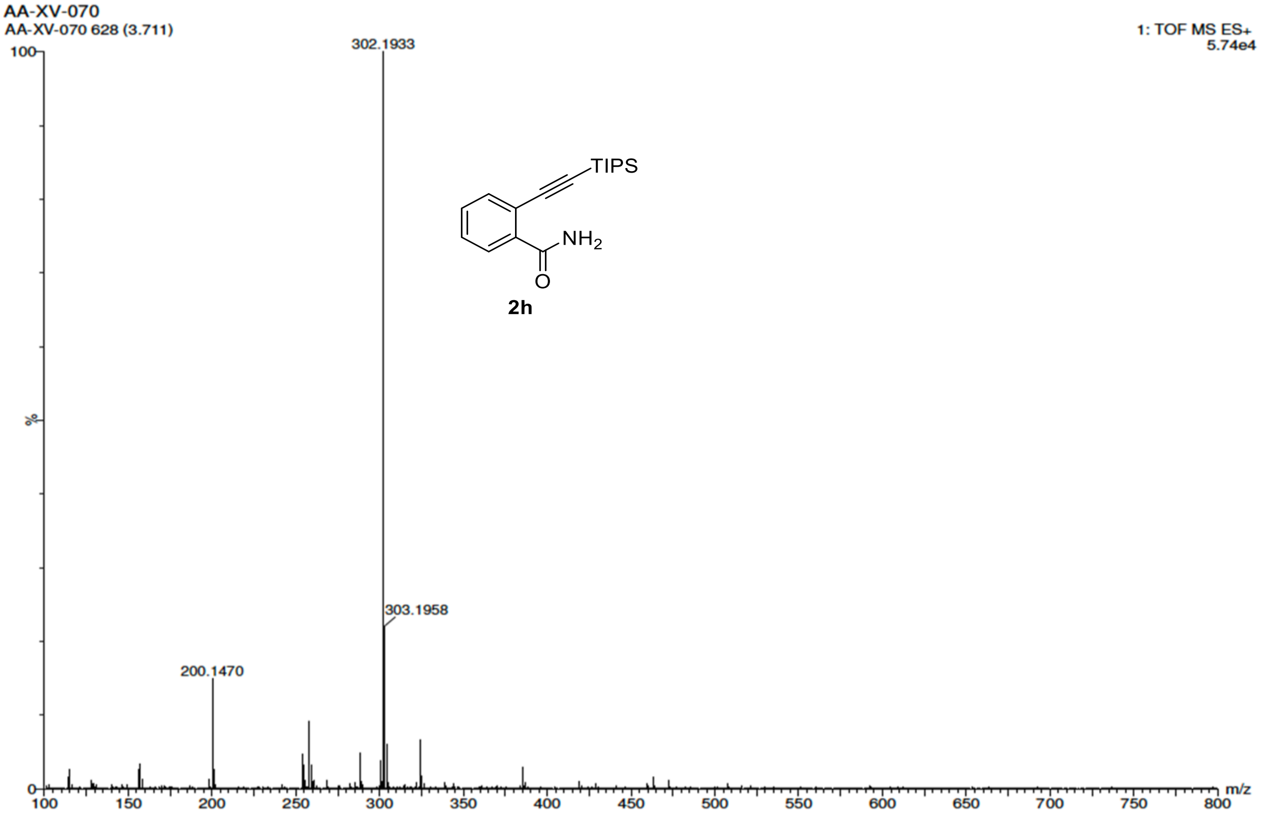

HRMS (ESI) *m/z* calcd for $C_{18}H_{27}NOSi$ $[M + H]^+$ 302.1935; found 302.1933.

5-Nitro-2-((triisopropylsilyl)ethynyl)benzamide (2i)

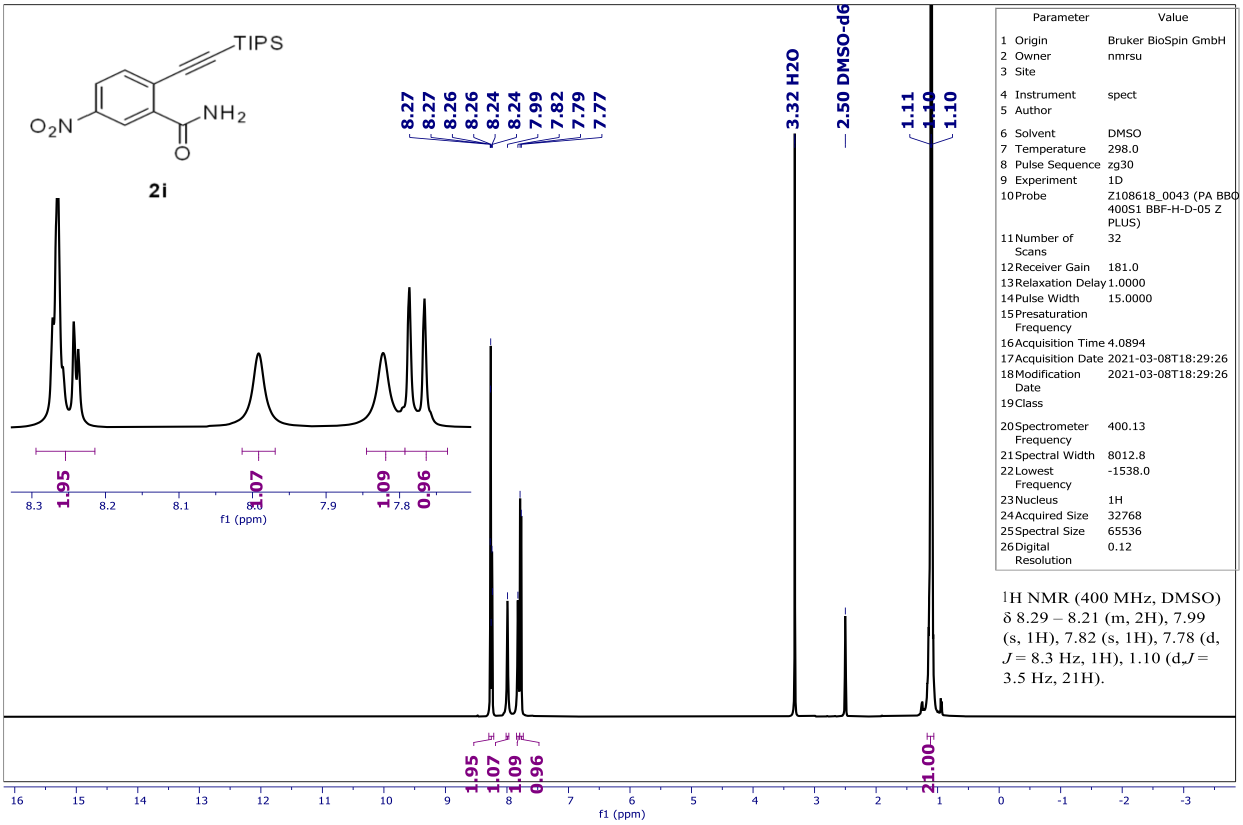

^{1}H NMR (400 MHz, DMSO) δ 8.29 – 8.21 (m, 2H), 7.99 (s, 1H), 7.82 (s, 1H), 7.78 (d, *J* = 8.3 Hz, 1H), 1.10 (d, *J* = 3.5 Hz, 21H).

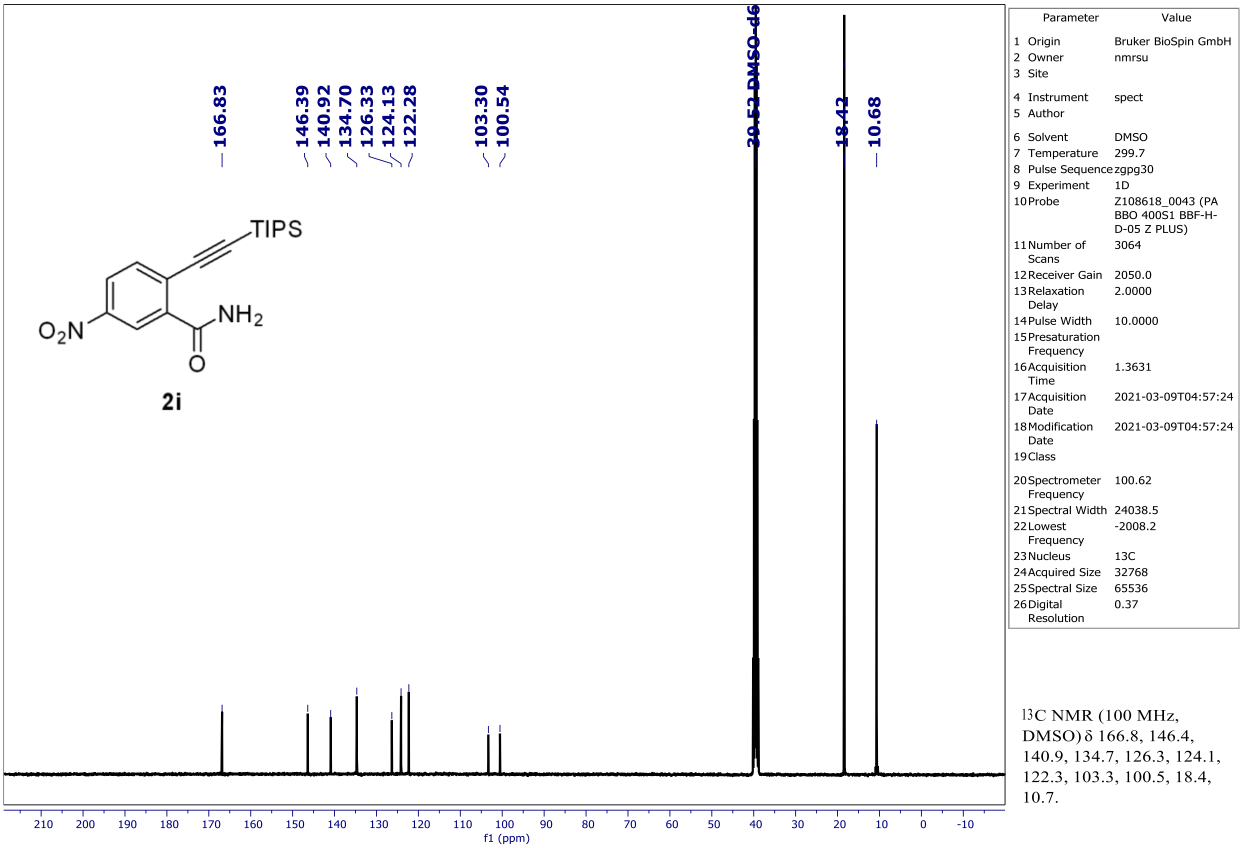

^{13}C NMR (100 MHz, DMSO) δ 166.8, 146.4, 140.9, 134.7, 126.3, 124.1, 122.3, 103.3, 100.5, 18.4, 10.7.

5-Nitro-2-((triisopropylsilyl)ethynyl)benzamide (2i)

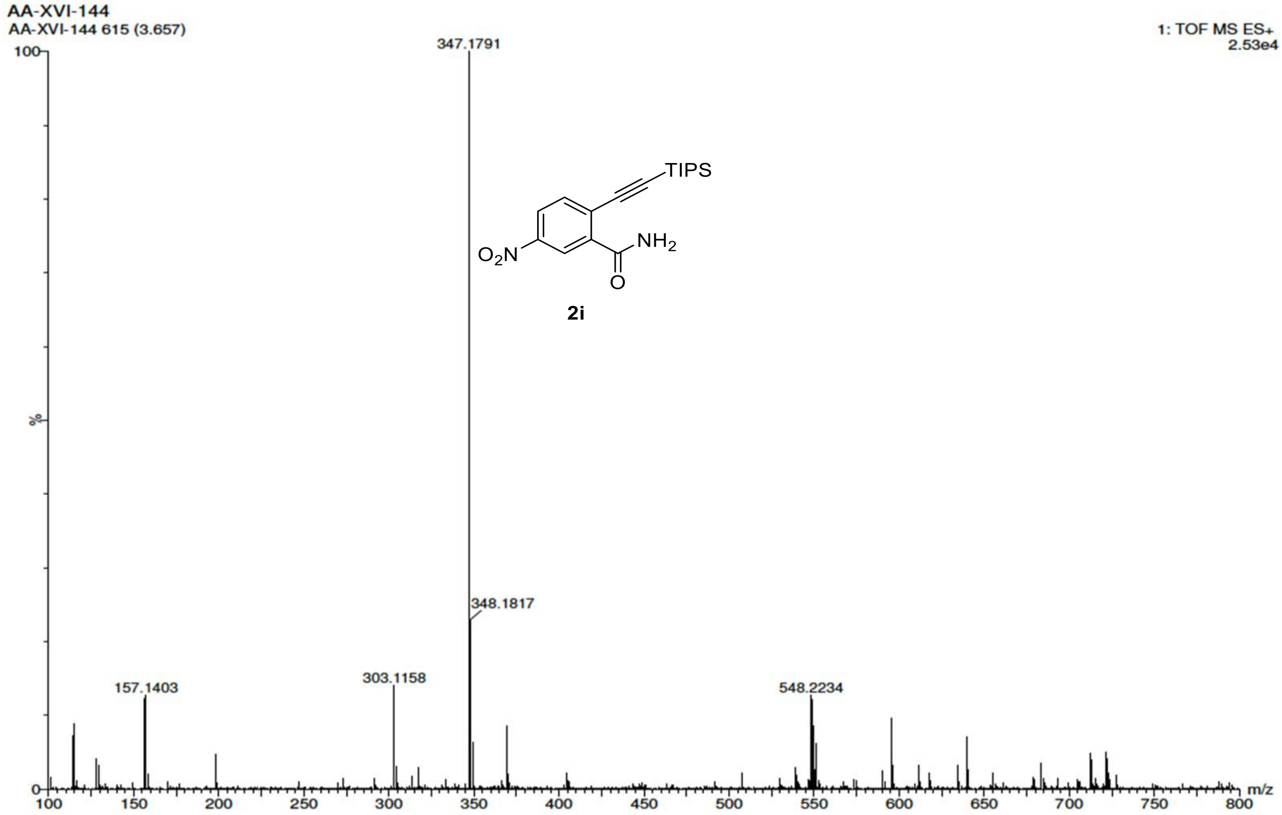

HRMS (ESI) *m/z* calcd for $C_{18}H_{26}N_2O_3Si$ $[M + H]^+$ 347.1785; found 347.1791.

2-Iodo-*N*-(methylsulfonyl)benzamide (3a)

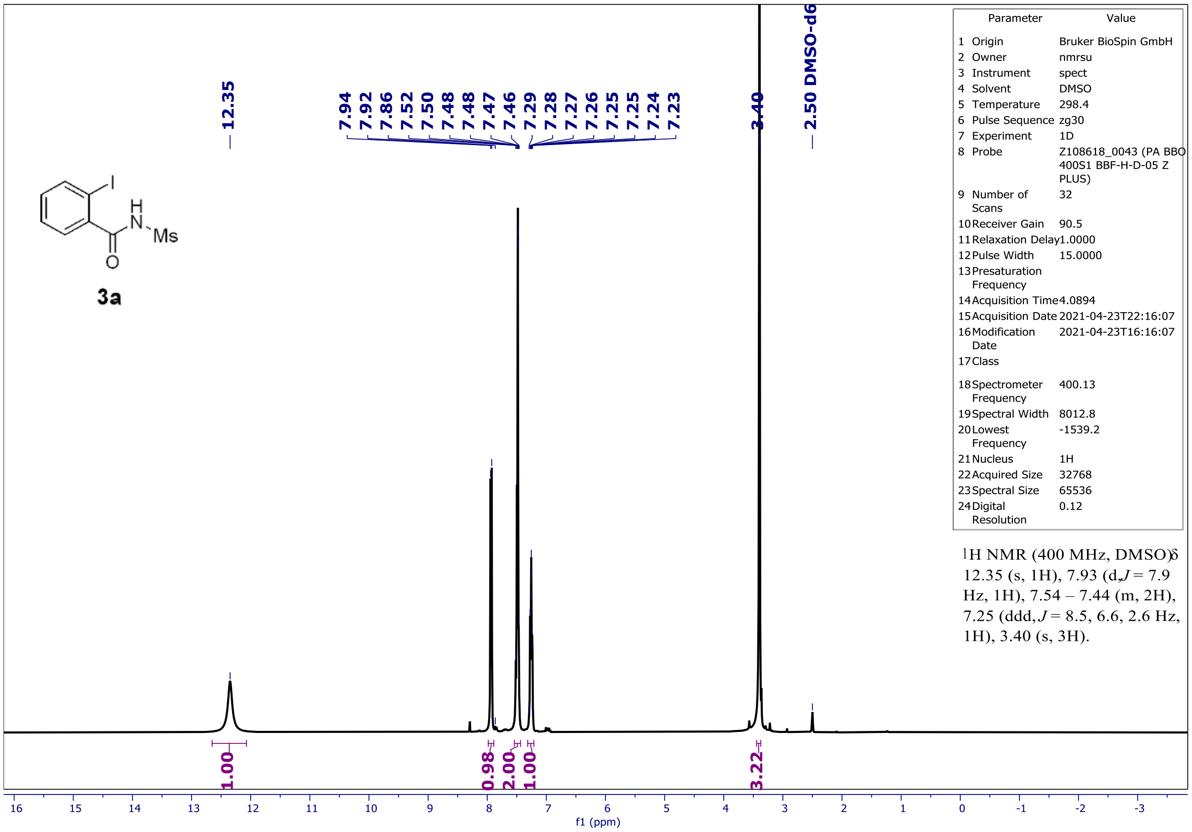

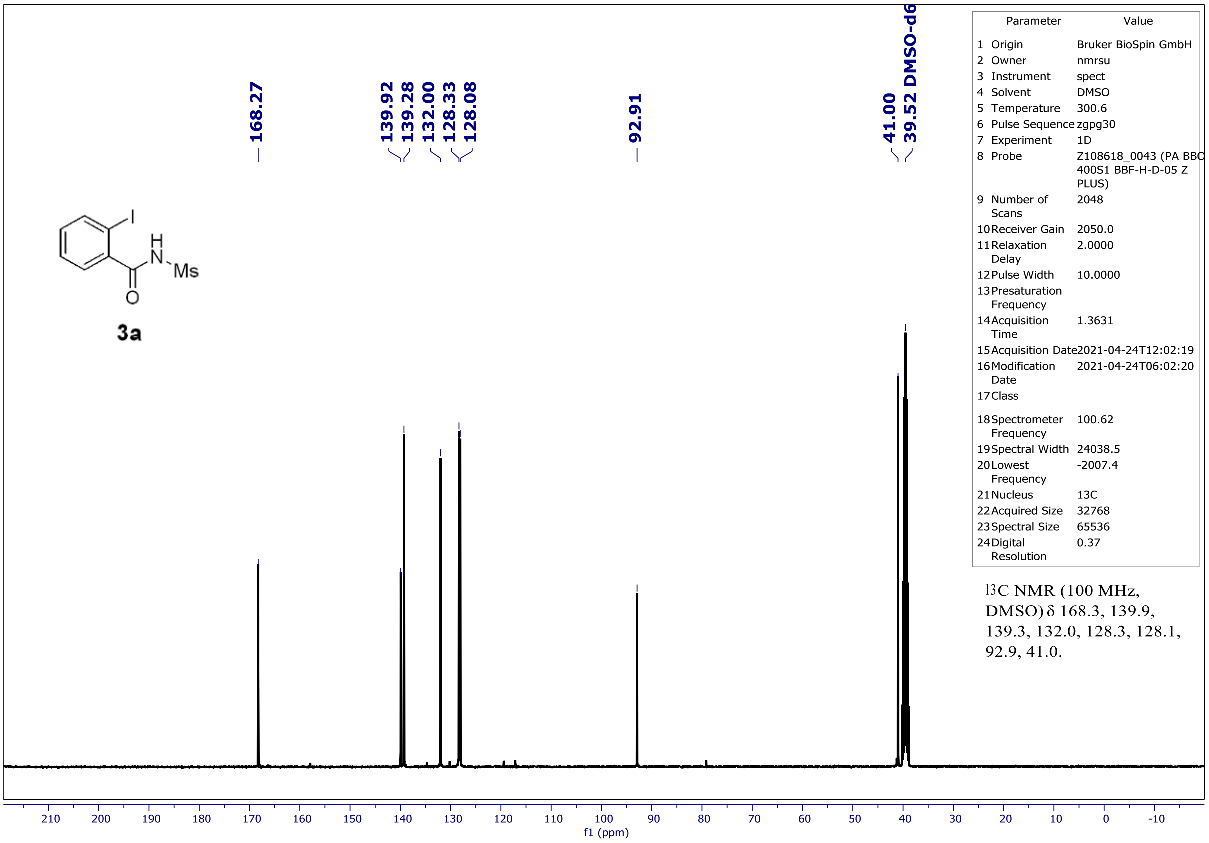

5-Fluoro-2-iodo-*N*-(methylsulfonyl)benzamide (3b)

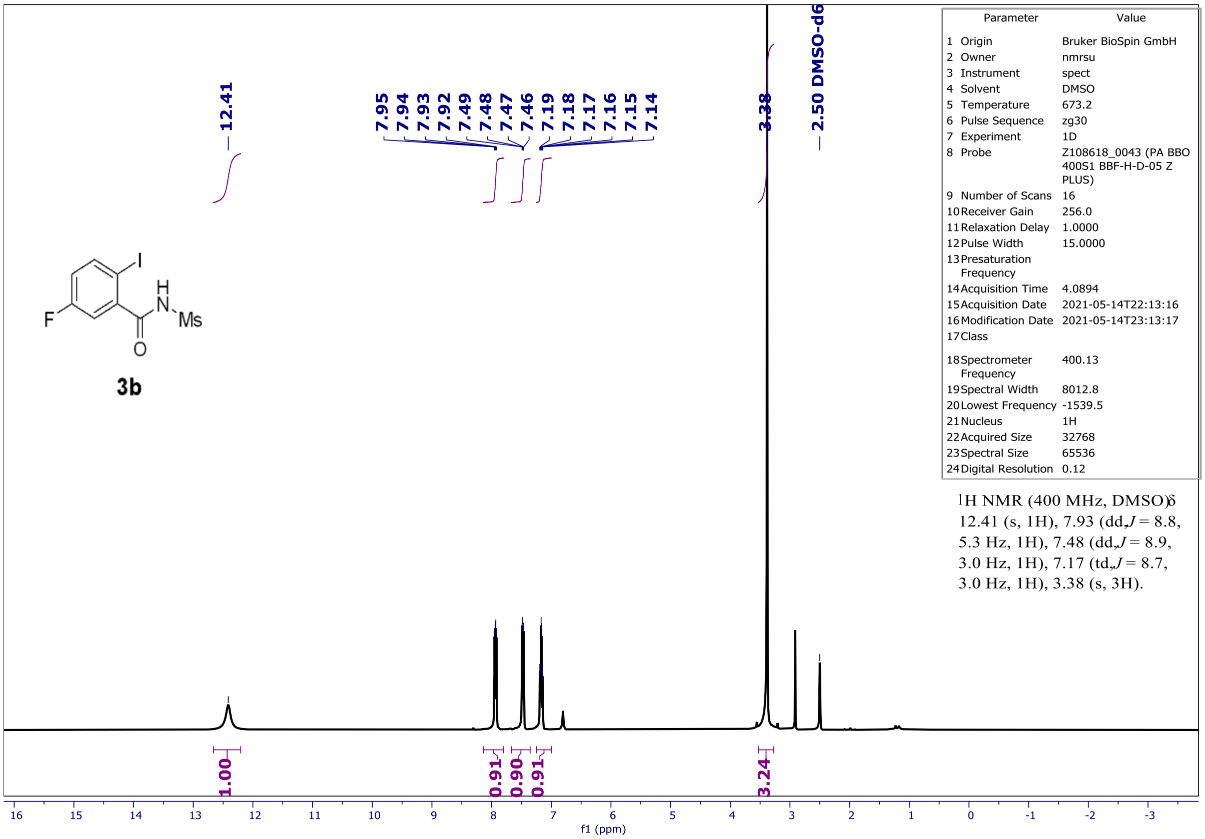

^{1}H NMR (400 MHz, DMSO) δ 12.41 (s, 1H), 7.93 (dd, J = 8.8, 5.3 Hz, 1H), 7.48 (dd, J = 8.9, 3.0 Hz, 1H), 7.17 (td, J = 8.7, 3.0 Hz, 1H), 3.38 (s, 3H).

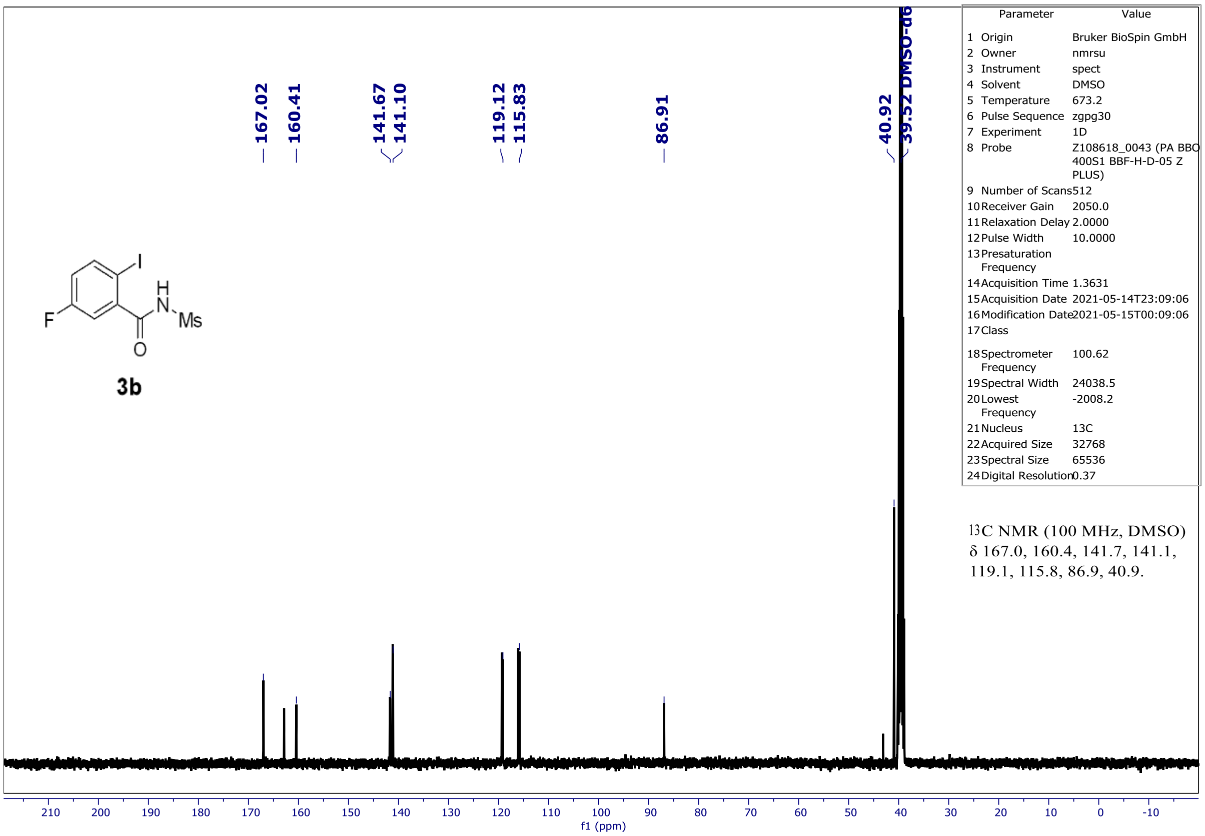

^{13}C NMR (100 MHz, DMSO) δ 167.0, 160.4, 141.7, 141.1, 119.1, 115.8, 86.9, 40.9.

5-Chloro-2-iodo-*N*-(methylsulfonyl)benzamide (3c)

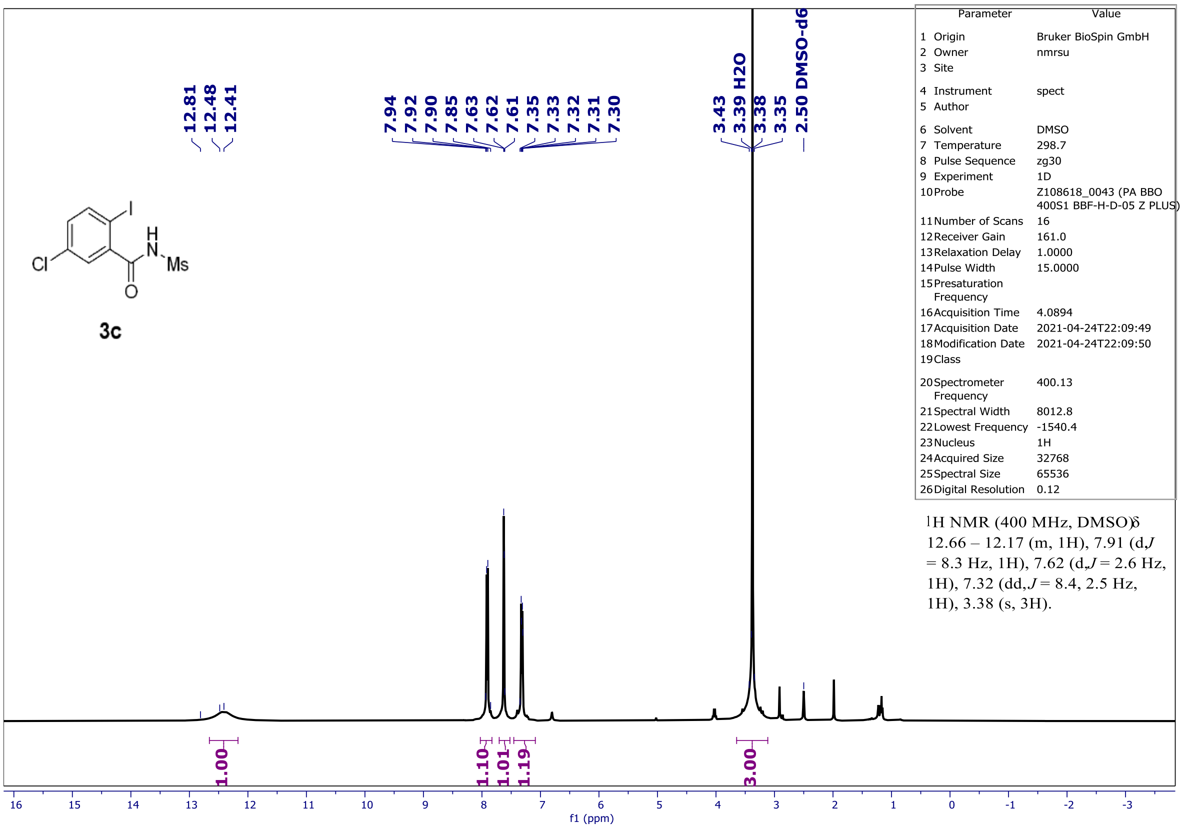

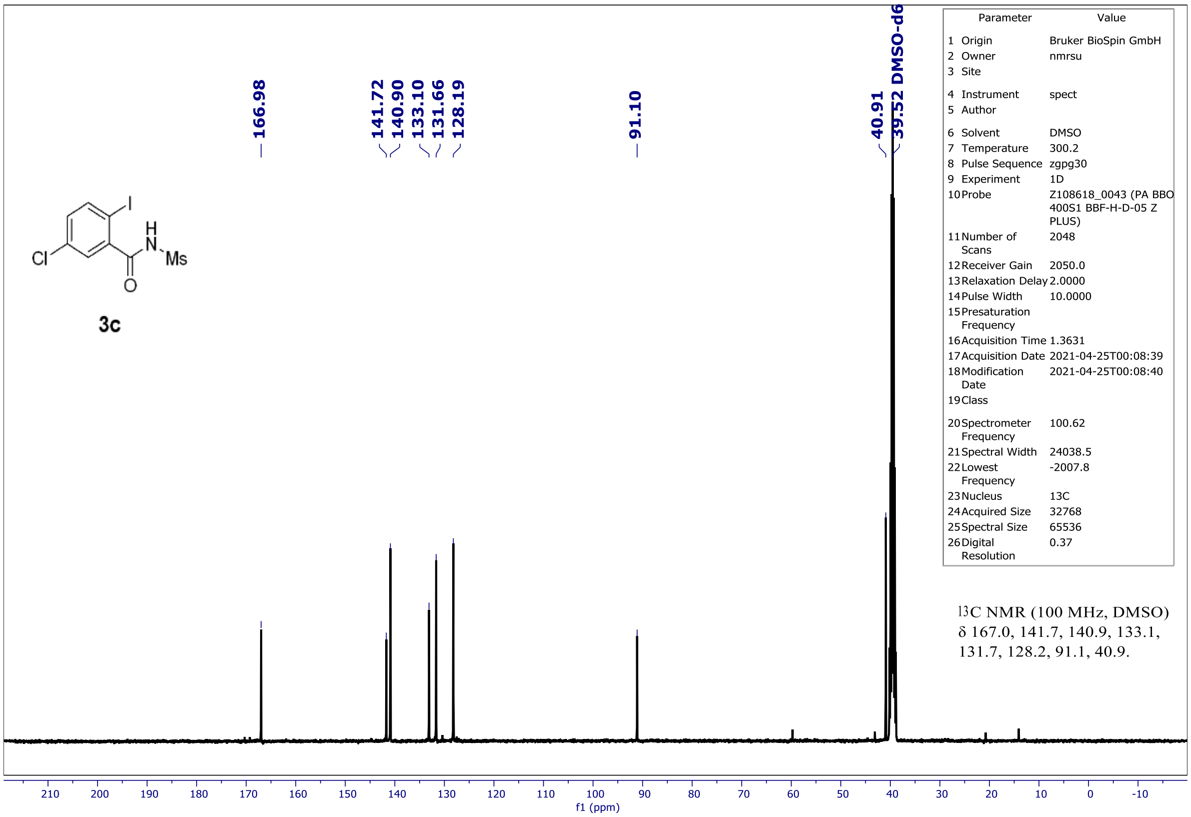

5-Bromo-2-iodo-*N*-(methylsulfonyl)benzamide (3d)

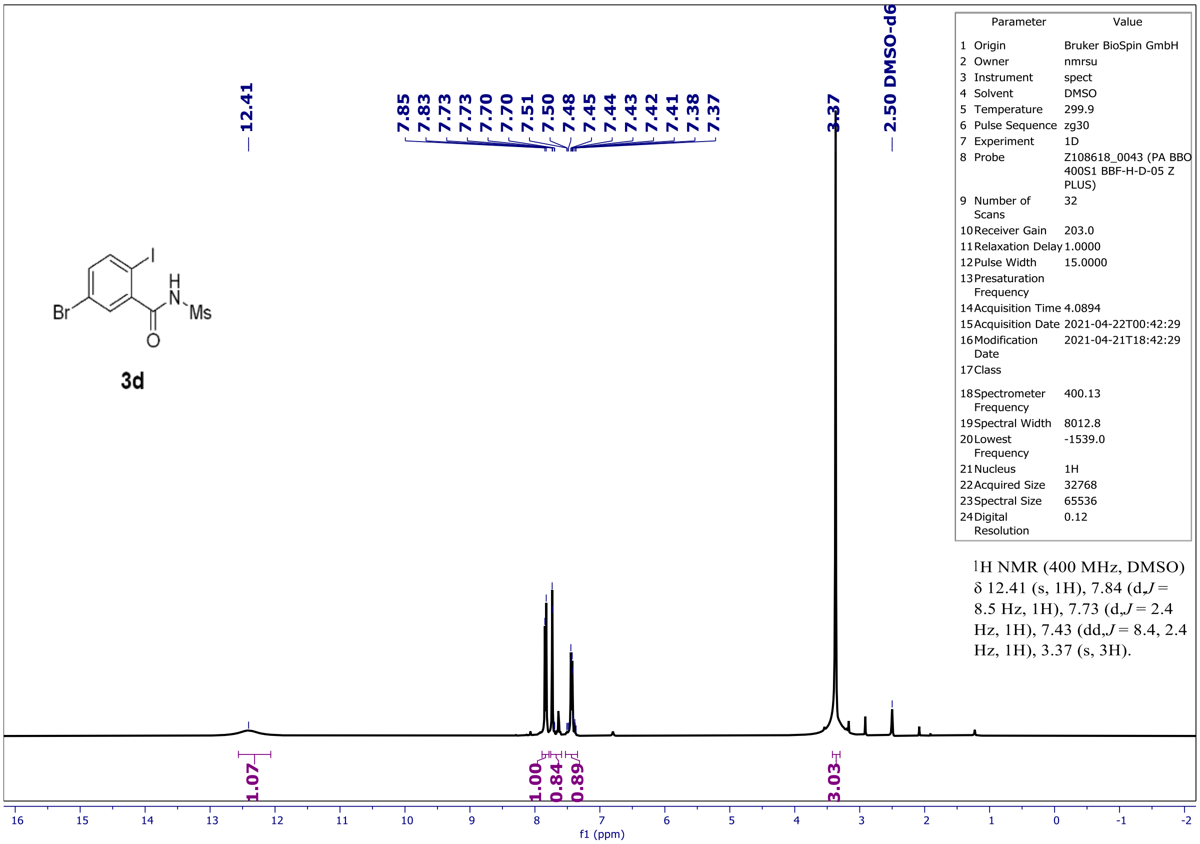

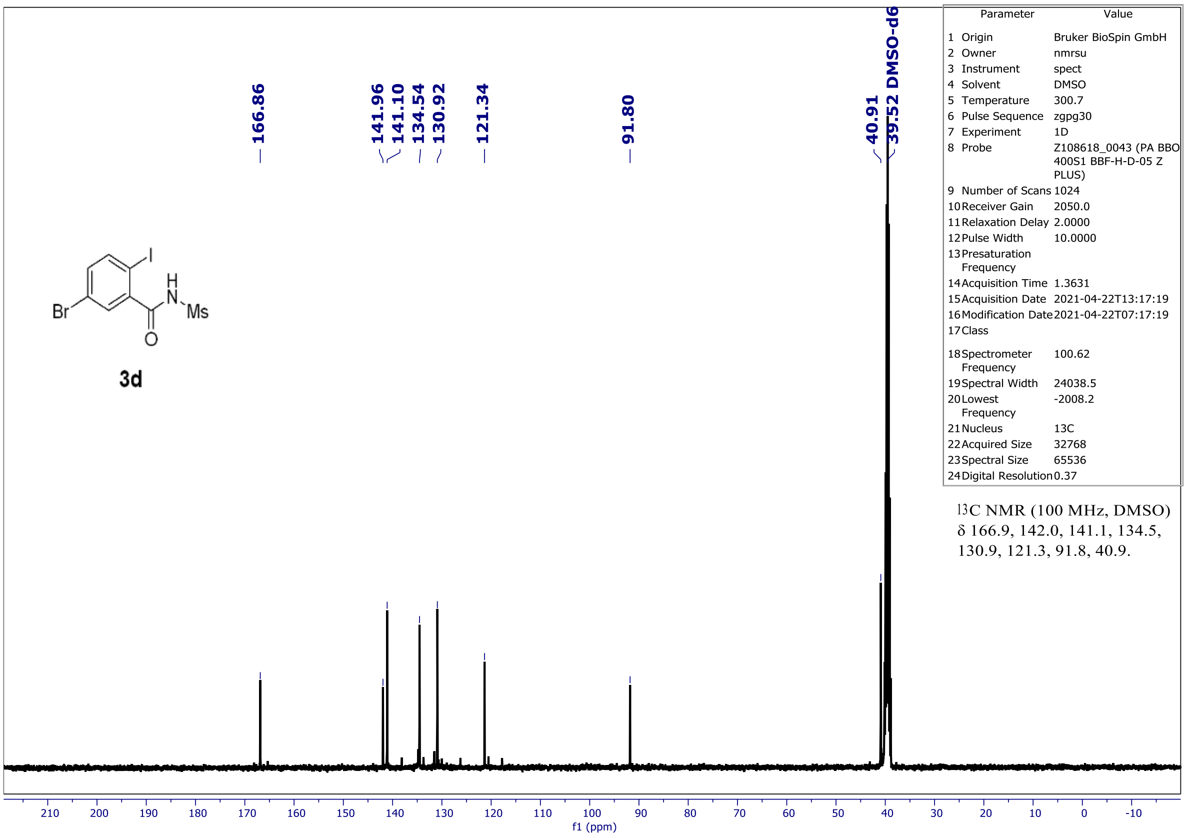

2-Iodo-5-methyl-*N*-(methylsulfonyl)benzamide (3e)

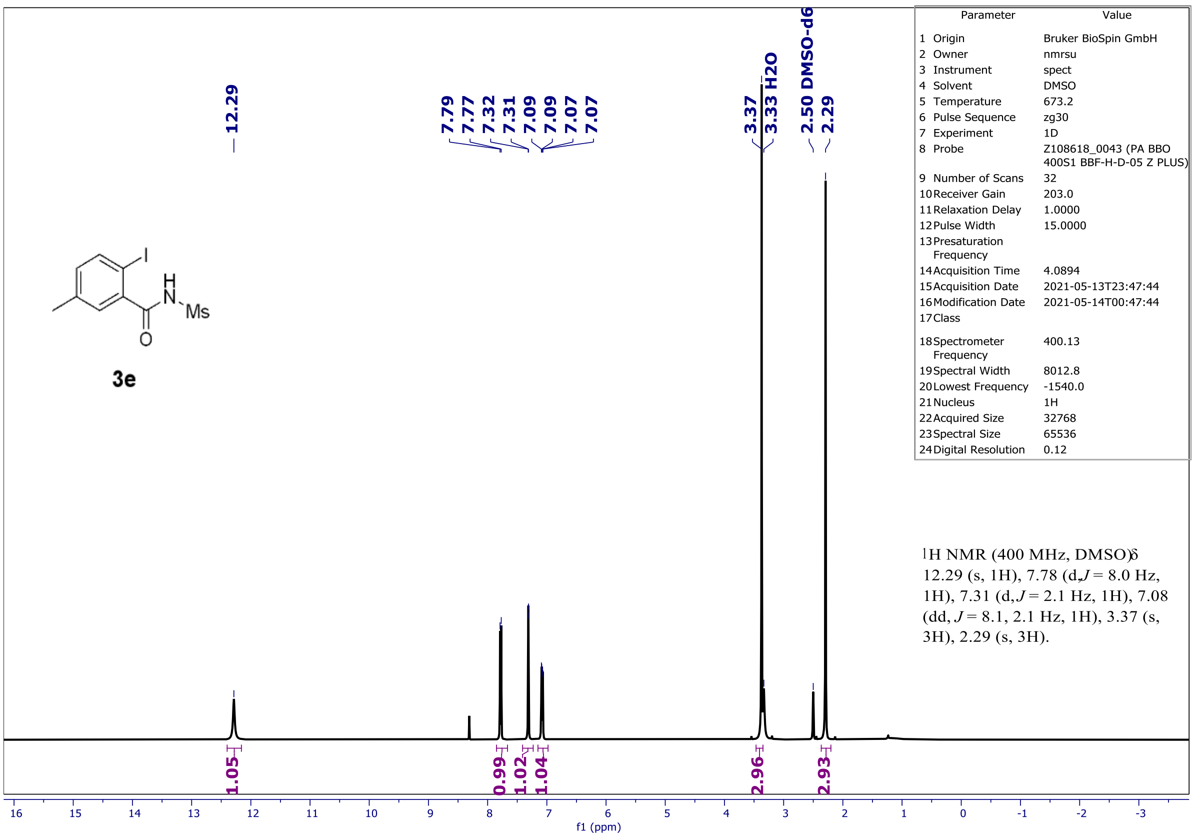

Parameter	Value
1 Origin	Bruker BioSpin GmbH
2 Owner	nmrsu
3 Instrument	spect
4 Solvent	DMSO
5 Temperature	673.2
6 Pulse Sequence	zg30
7 Experiment	1D
8 Probe	Z108618_0043 (PA BBO 400S1 BBF-H-D-05 Z PLUS)
9 Number of Scans	32
10 Receiver Gain	203.0
11 Relaxation Delay	1.0000
12 Pulse Width	15.0000
13 Presaturation Frequency	
14 Acquisition Time	4.0894
15 Acquisition Date	2021-05-13T23:47:44
16 Modification Date	2021-05-14T00:47:44
17 Class	
18 Spectrometer Frequency	400.13
19 Spectral Width	8012.8
20 Lowest Frequency	-1540.0
21 Nucleus	1H
22 Acquired Size	32768
23 Spectral Size	65536
24 Digital Resolution	0.12

^{1}H NMR (400 MHz, DMSO) δ 12.29 (s, 1H), 7.78 (d, J = 8.0 Hz, 1H), 7.31 (d, J = 2.1 Hz, 1H), 7.08 (dd, J = 8.1, 2.1 Hz, 1H), 3.37 (s, 3H), 2.29 (s, 3H).

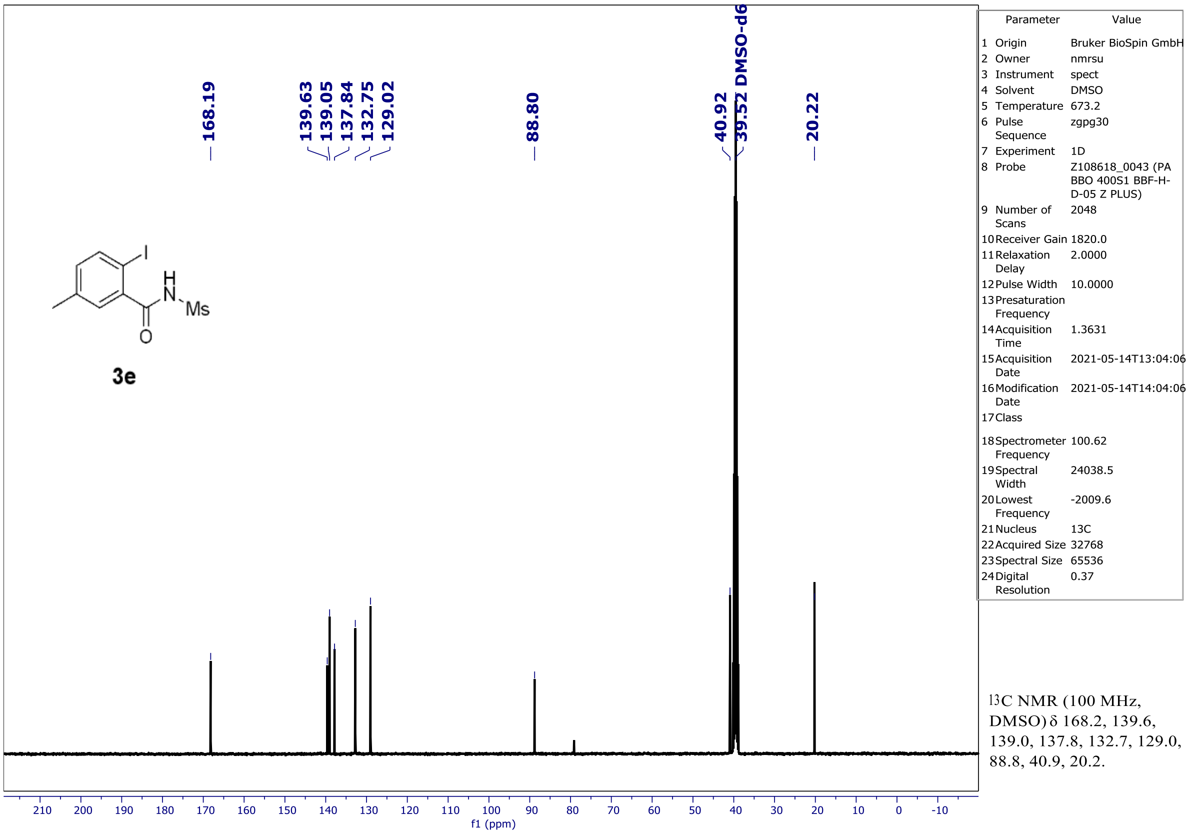

Parameter	Value
1 Origin	Bruker BioSpin GmbH
2 Owner	nmrsu
3 Instrument	spect
4 Solvent	DMSO
5 Temperature	673.2
6 Pulse Sequence	zgpg30
7 Experiment	1D
8 Probe	Z108618_0043 (PA BBO 400S1 BBF-H-D-05 Z PLUS)
9 Number of Scans	2048
10 Receiver Gain	1820.0
11 Relaxation Delay	2.0000
12 Pulse Width	10.0000
13 Presaturation Frequency	
14 Acquisition Time	1.3631
15 Acquisition Date	2021-05-14T13:04:06
16 Modification Date	2021-05-14T14:04:06
17 Class	
18 Spectrometer Frequency	100.62
19 Spectral Width	24038.5
20 Lowest Frequency	-2009.6
21 Nucleus	13C
22 Acquired Size	32768
23 Spectral Size	65536
24 Digital Resolution	0.37

^{13}C NMR (100 MHz, DMSO) δ 168.2, 139.6, 139.0, 137.8, 132.7, 129.0, 88.8, 40.9, 20.2.

2-Iodo-*N*-(methylsulfonyl)-5-nitrobenzamide (3g)

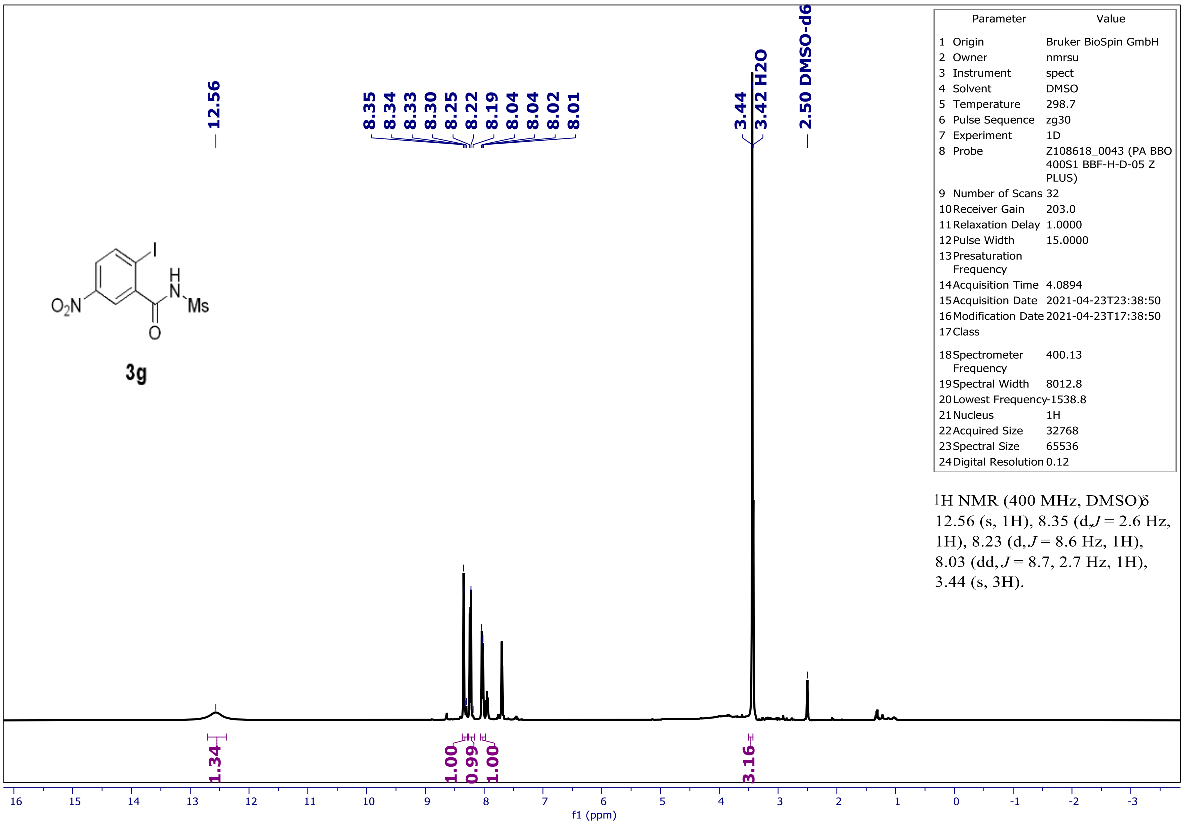

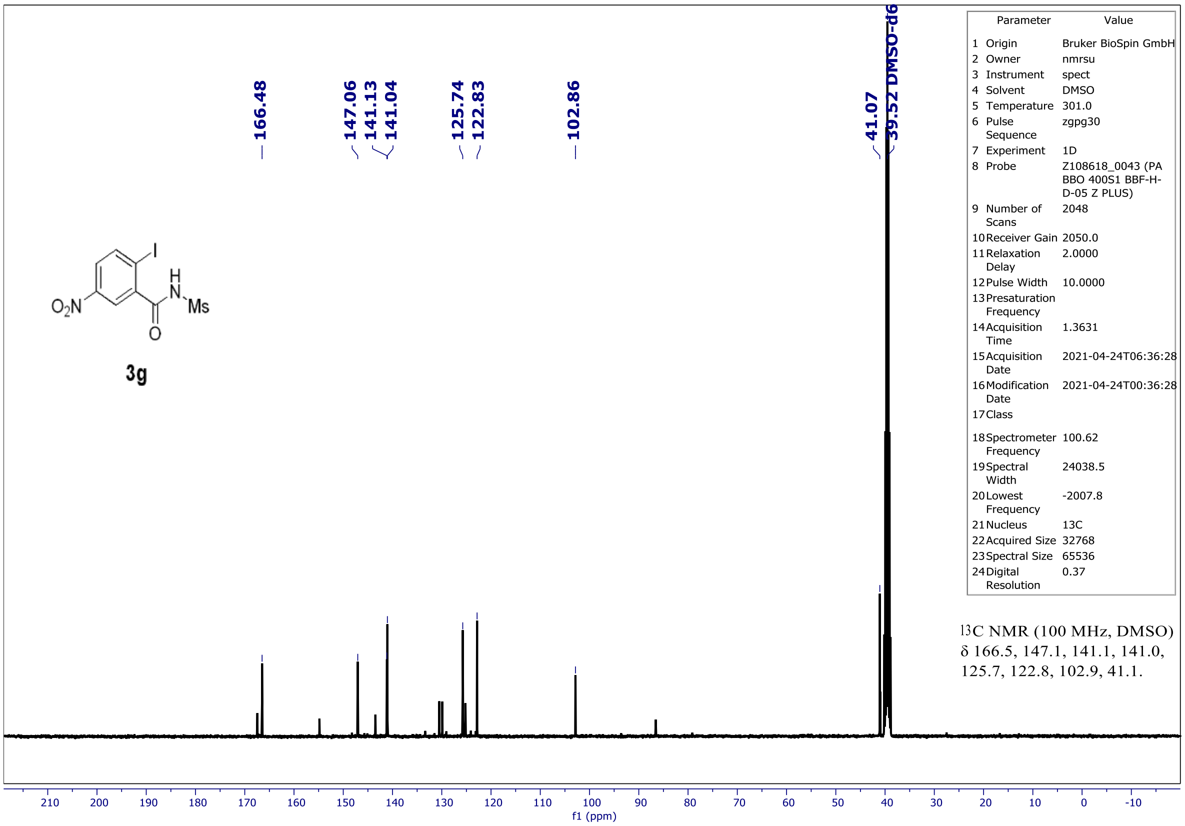

4-Chloro-2-iodo-*N*-(methylsulfonyl)benzamide (3h)

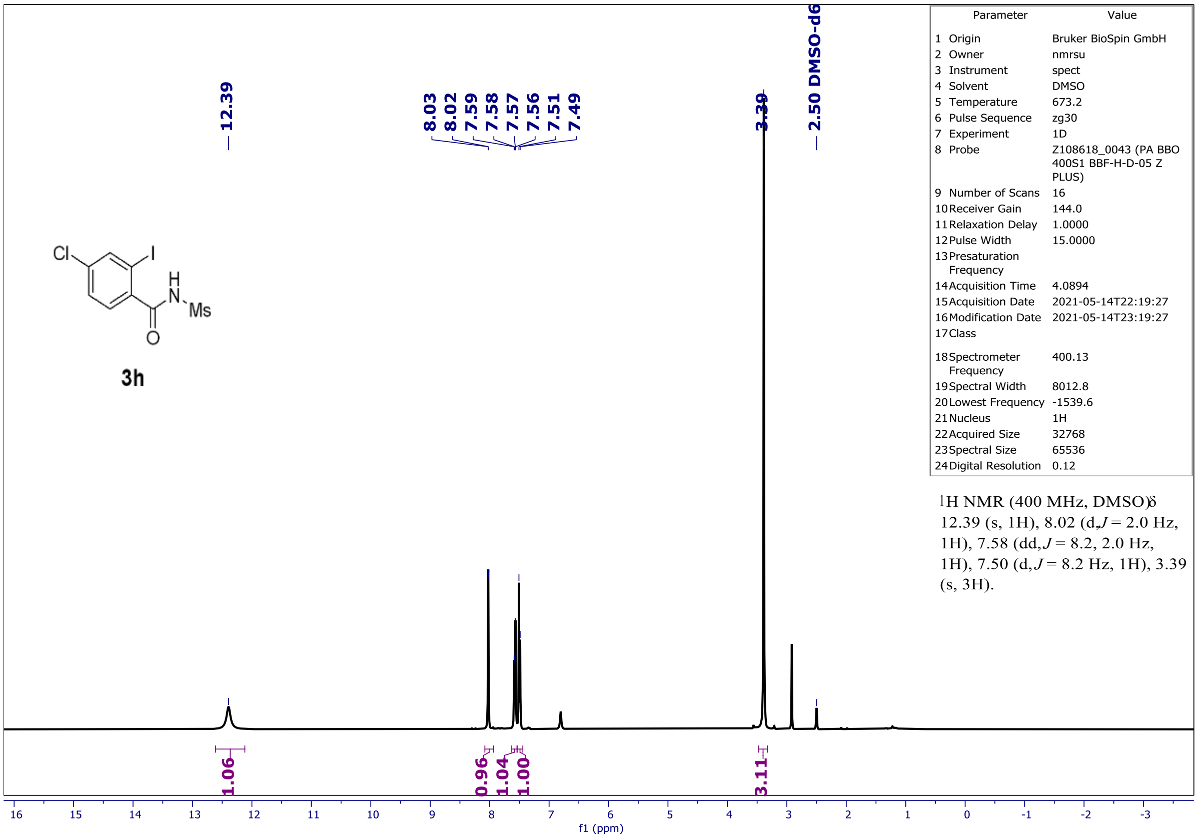

Parameter	Value
1 Origin	Bruker BioSpin GmbH
2 Owner	nmrsu
3 Instrument	spect
4 Solvent	DMSO
5 Temperature	673.2
6 Pulse Sequence	zg30
7 Experiment	1D
8 Probe	Z108618_0043 (PA BBO 400S1 BBF-H-D-05 Z PLUS)
9 Number of Scans	16
10 Receiver Gain	144.0
11 Relaxation Delay	1.0000
12 Pulse Width	15.0000
13 Presaturation Frequency	
14 Acquisition Time	4.0894
15 Acquisition Date	2021-05-14T22:19:27
16 Modification Date	2021-05-14T23:19:27
17 Class	
18 Spectrometer Frequency	400.13
19 Spectral Width	8012.8
20 Lowest Frequency	-1539.6
21 Nucleus	1H
22 Acquired Size	32768
23 Spectral Size	65536
24 Digital Resolution	0.12

^{1}H NMR (400 MHz, DMSO) δ 12.39 (s, 1H), 8.02 (d, J = 2.0 Hz, 1H), 7.58 (dd, J = 8.2, 2.0 Hz, 1H), 7.50 (d, J = 8.2 Hz, 1H), 3.39 (s, 3H).

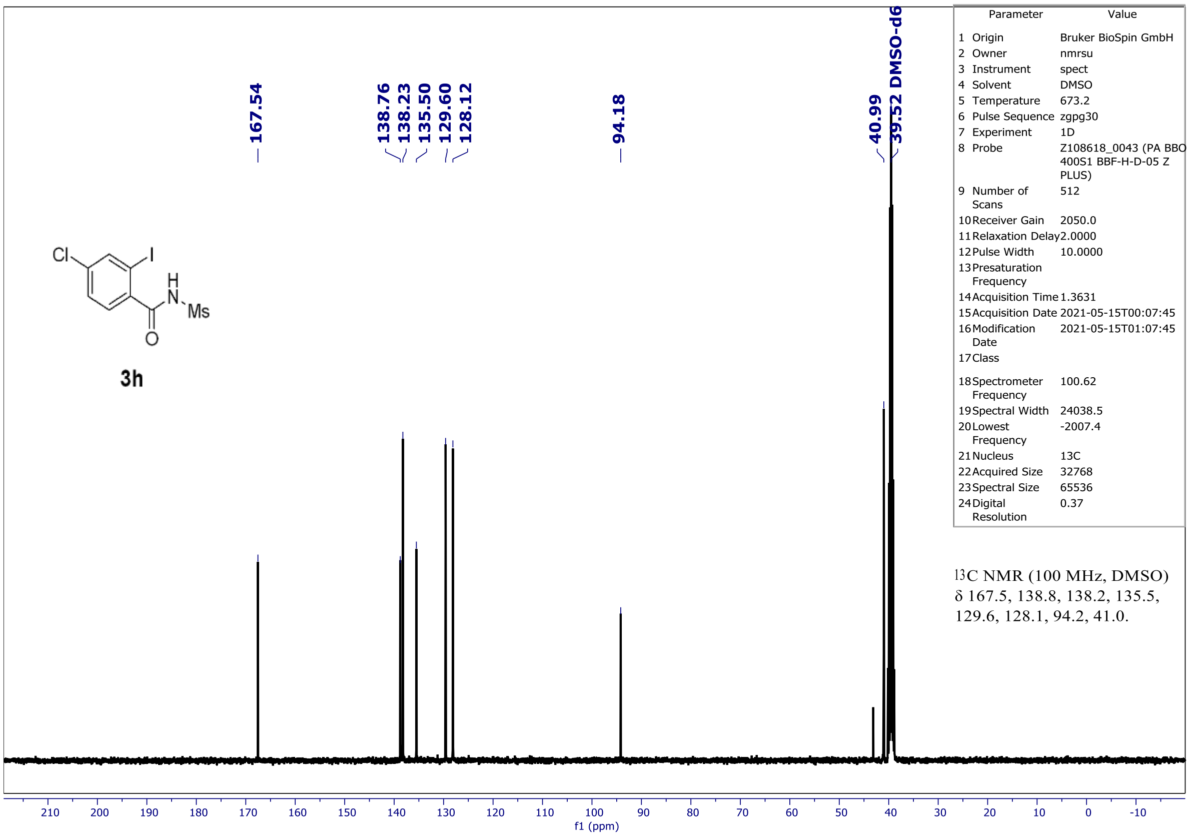

Parameter	Value
1 Origin	Bruker BioSpin GmbH
2 Owner	nmrsu
3 Instrument	spect
4 Solvent	DMSO
5 Temperature	673.2
6 Pulse Sequence	zgpg30
7 Experiment	1D
8 Probe	Z108618_0043 (PA BBO 400S1 BBF-H-D-05 Z PLUS)
9 Number of Scans	512
10 Receiver Gain	2050.0
11 Relaxation Delay	2.0000
12 Pulse Width	10.0000
13 Presaturation Frequency	
14 Acquisition Time	1.3631
15 Acquisition Date	2021-05-15T00:07:45
16 Modification Date	2021-05-15T01:07:45
17 Class	
18 Spectrometer Frequency	100.62
19 Spectral Width	24038.5
20 Lowest Frequency	-2007.4
21 Nucleus	13C
22 Acquired Size	32768
23 Spectral Size	65536
24 Digital Resolution	0.37

^{13}C NMR (100 MHz, DMSO) δ 167.5, 138.8, 138.2, 135.5, 129.6, 128.1, 94.2, 41.0.

2-Iodo-*N*-(methylsulfonyl)-4-nitrobenzamide (3i)

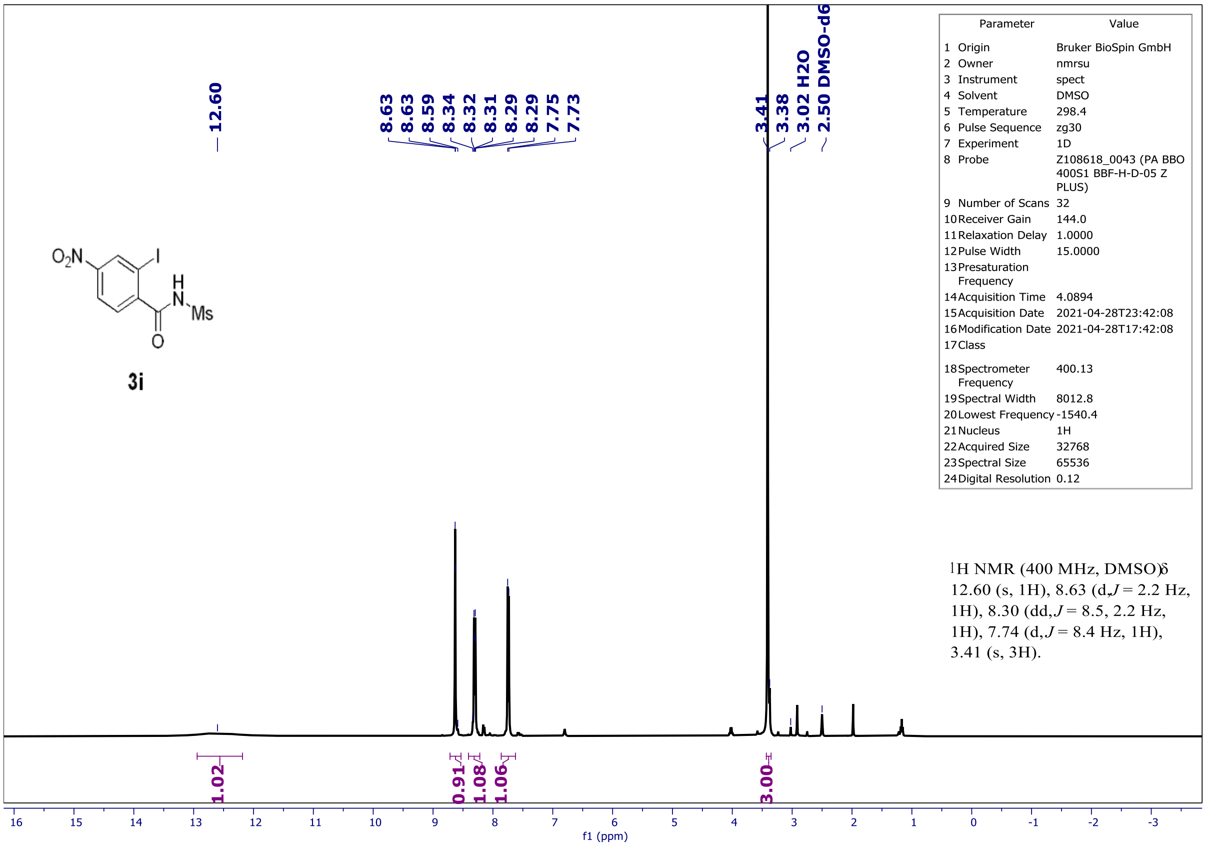

^{1}H NMR (400 MHz, DMSO) δ 12.60 (s, 1H), 8.63 (d, *J* = 2.2 Hz, 1H), 8.30 (dd, *J* = 8.5, 2.2 Hz, 1H), 7.74 (d, *J* = 8.4 Hz, 1H), 3.41 (s, 3H).

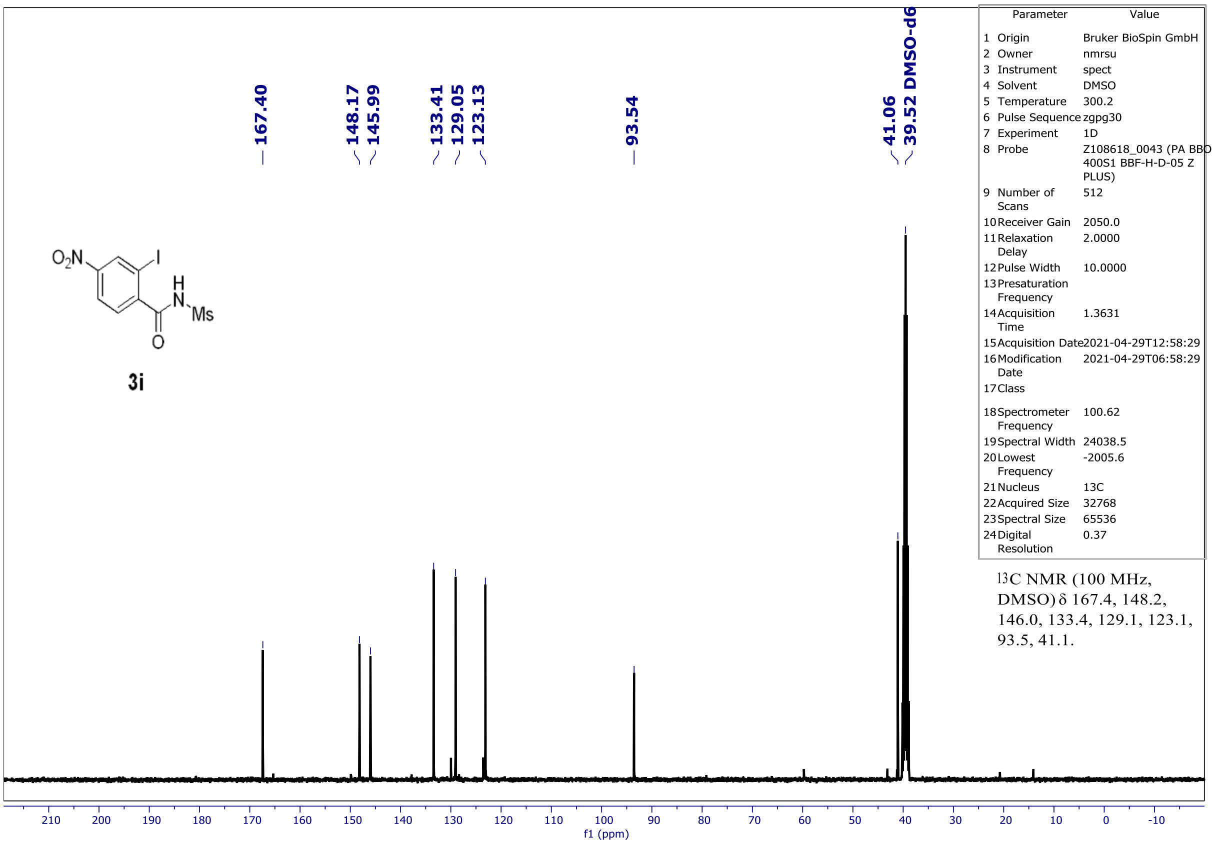

^{13}C NMR (100 MHz, DMSO) δ 167.4, 148.2, 146.0, 133.4, 129.1, 123.1, 93.5, 41.1.

Ethyl (*E*)-2-(2-(methylsulfonyl)-3-oxoisoindolin-1-ylidene)acetate (4a)

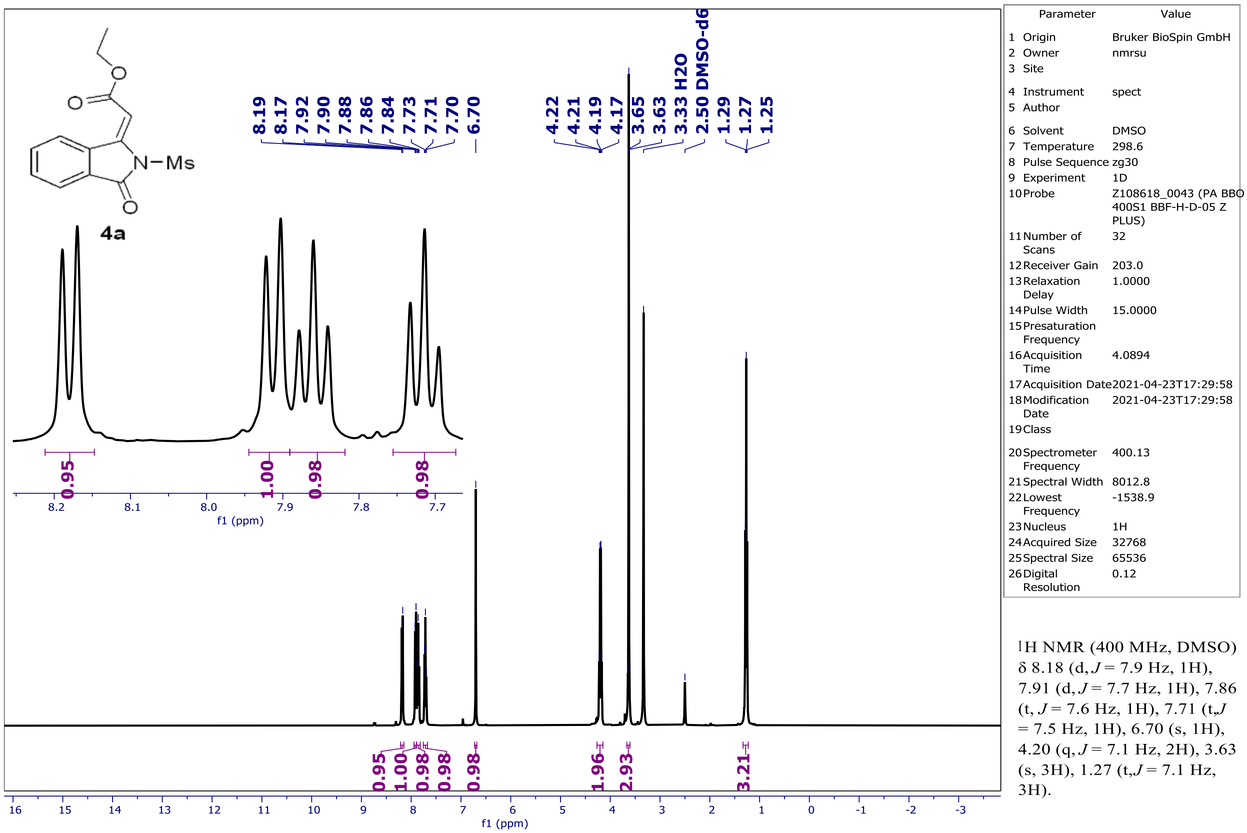

	Parameter	Value
1	Origin	Bruker BioSpin GmbH
2	Owner	nmrsu
3	Site	
4	Instrument	spect
5	Author	
6	Solvent	DMSO
7	Temperature	298.6
8	Pulse Sequence	zg30
9	Experiment	1D
10	Probe	Z108618_0043 (PA BBO 400S1 BBF-H-D-05 Z PLUS)
11	Number of Scans	32
12	Receiver Gain	203.0
13	Relaxation Delay	1.0000
14	Pulse Width	15.0000
15	Presaturation Frequency	
16	Acquisition Time	4.0894
17	Acquisition Date	2021-04-23T17:29:58
18	Modification Date	2021-04-23T17:29:58
19	Class	
20	Spectrometer Frequency	400.13
21	Spectral Width	8012.8
22	Lowest Frequency	-1538.9
23	Nucleus	1H
24	Acquired Size	32768
25	Spectral Size	65536
26	Digital Resolution	0.12

^{1}H NMR (400 MHz, DMSO) δ 8.18 (d, J = 7.9 Hz, 1H), 7.91 (d, J = 7.7 Hz, 1H), 7.86 (t, J = 7.6 Hz, 1H), 7.71 (t,, J = 7.5 Hz, 1H), 6.70 (s, 1H), 4.20 (q, J = 7.1 Hz, 2H), 3.63 (s, 3H), 1.27 (t,, J = 7.1 Hz, 3H).

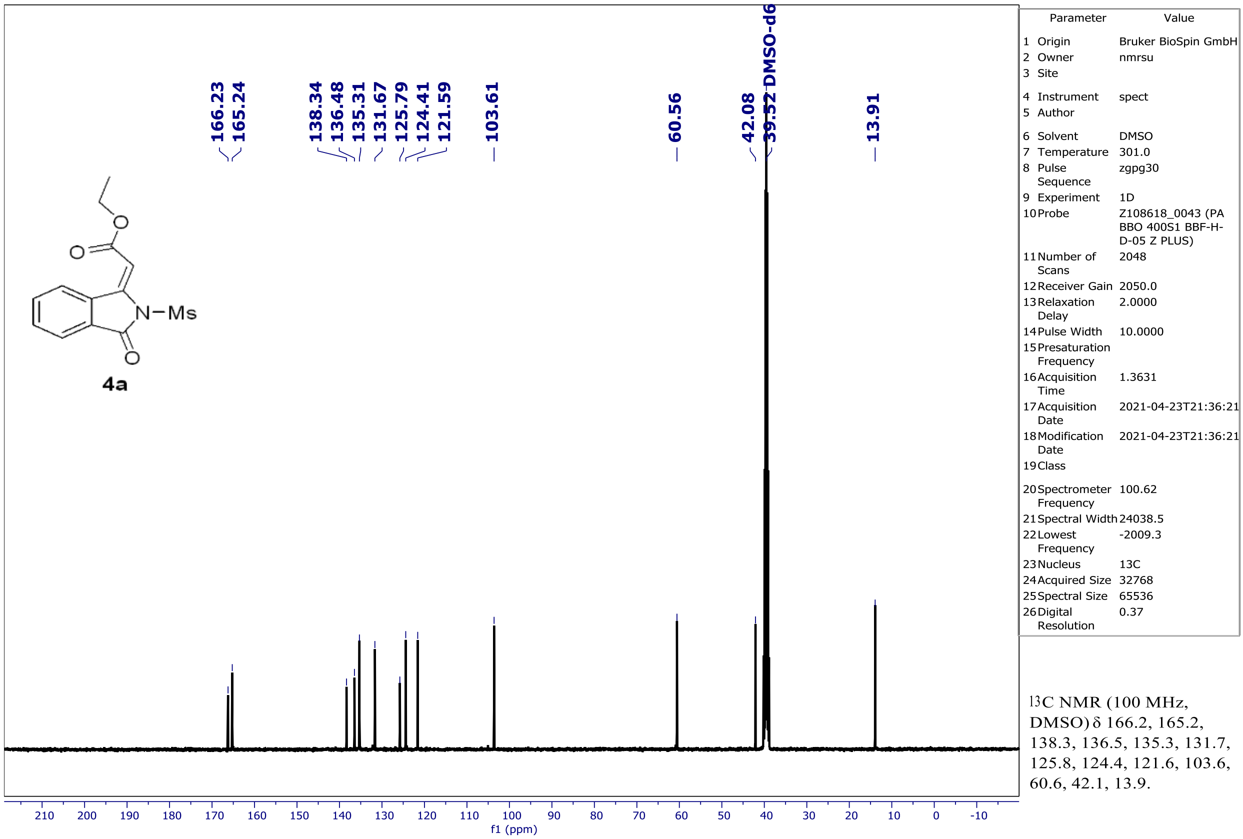

	Parameter	Value
1	Origin	Bruker BioSpin GmbH
2	Owner	nmrsu
3	Site	
4	Instrument	spect
5	Author	
6	Solvent	DMSO
7	Temperature	301.0
8	Pulse Sequence	zgpg30
9	Experiment	1D
10	Probe	Z108618_0043 (PA BBO 400S1 BBF-H-D-05 Z PLUS)
11	Number of Scans	2048
12	Receiver Gain	2050.0
13	Relaxation Delay	2.0000
14	Pulse Width	10.0000
15	Presaturation Frequency	
16	Acquisition Time	1.3631
17	Acquisition Date	2021-04-23T21:36:21
18	Modification Date	2021-04-23T21:36:21
19	Class	
20	Spectrometer Frequency	100.62
21	Spectral Width	24038.5
22	Lowest Frequency	-2009.3
23	Nucleus	13C
24	Acquired Size	32768
25	Spectral Size	65536
26	Digital Resolution	0.37

^{13}C NMR (100 MHz, DMSO) δ 166.2, 165.2, 138.3, 136.5, 135.3, 131.7, 125.8, 124.4, 121.6, 103.6, 60.6, 42.1, 13.9.

COSY

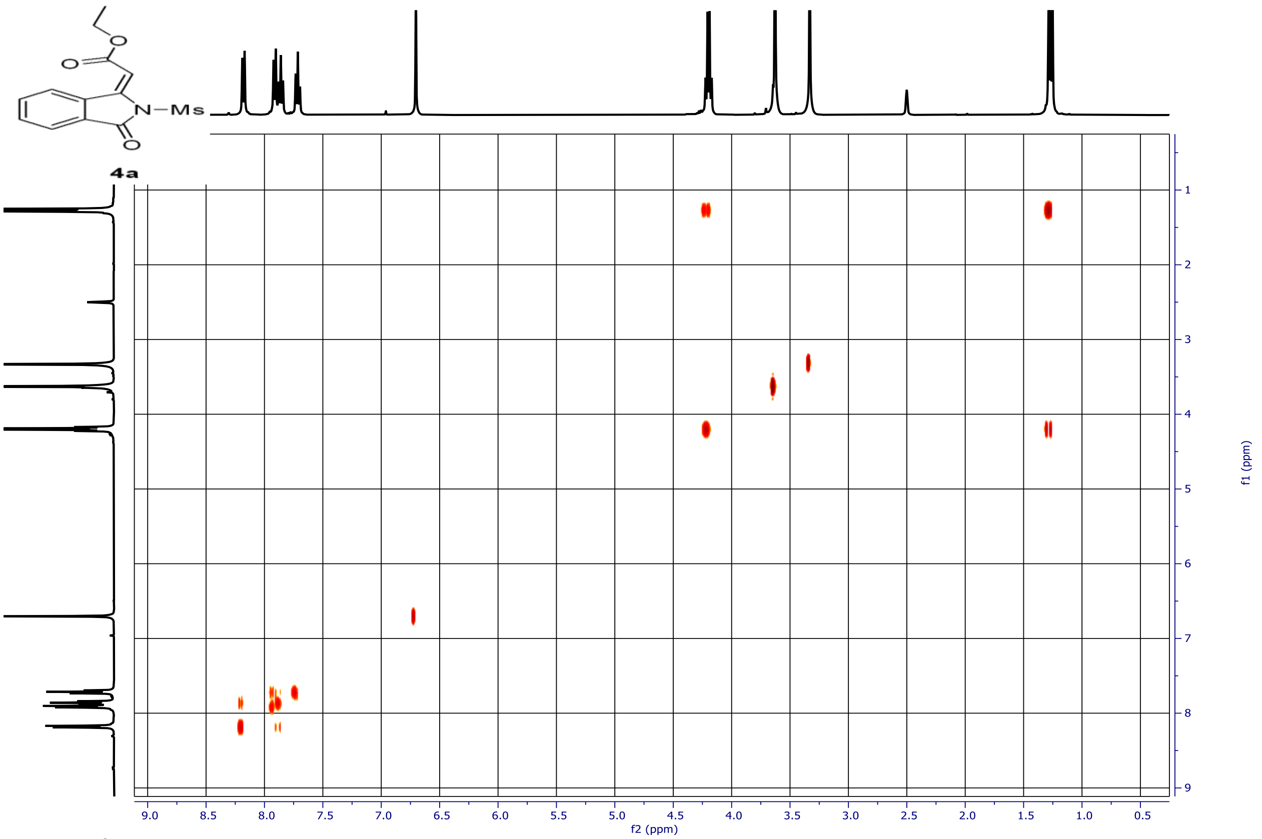

N−Ms
4a
f2 (ppm)
f1 (ppm)

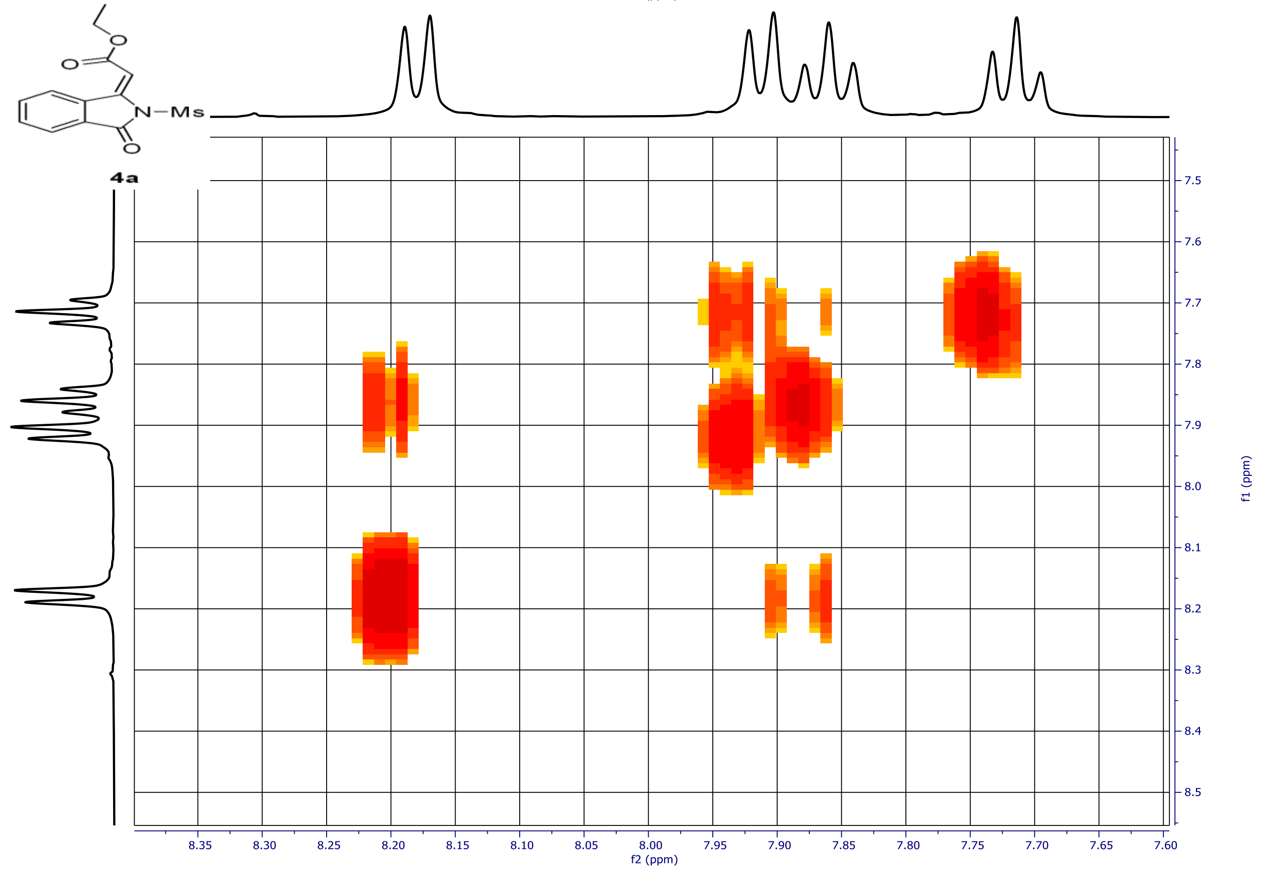

N−Ms
4a
f2 (ppm)
f1 (ppm)

HSQC

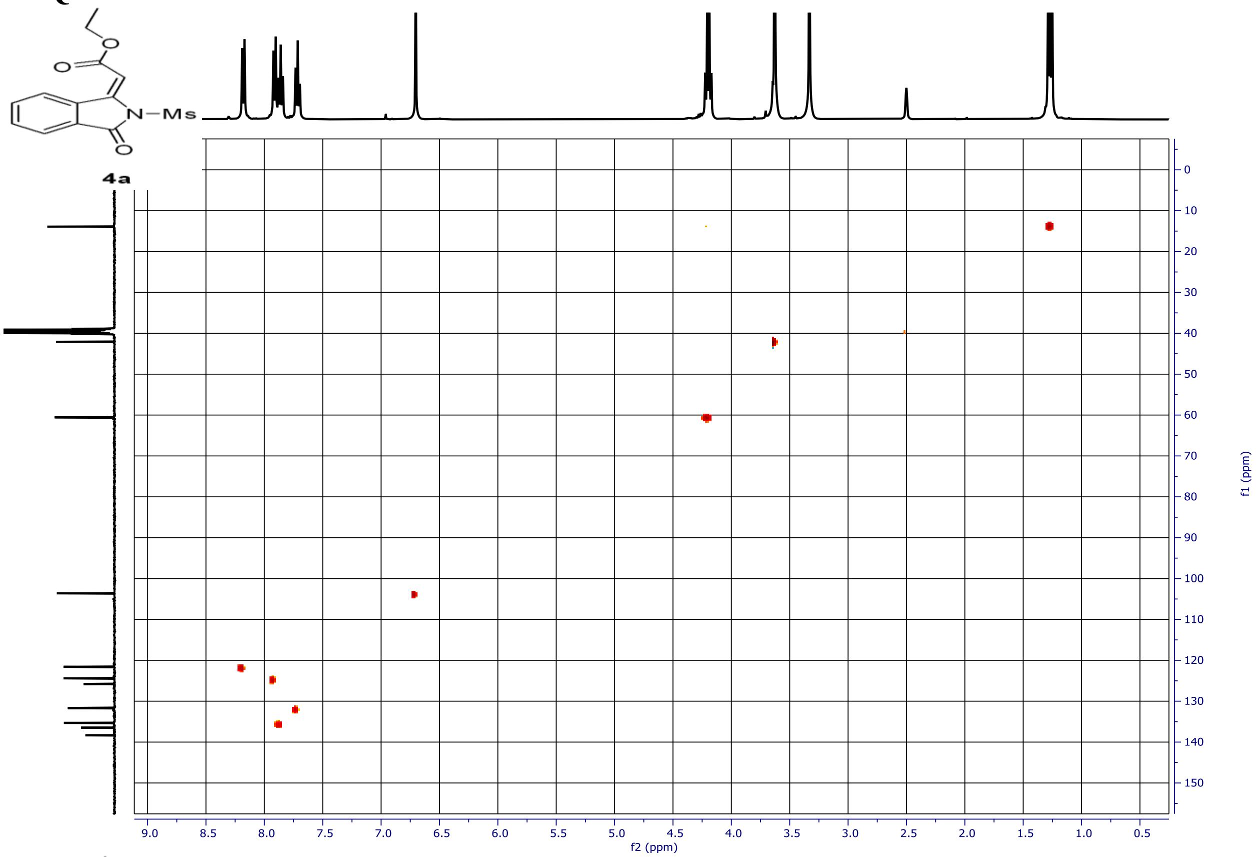

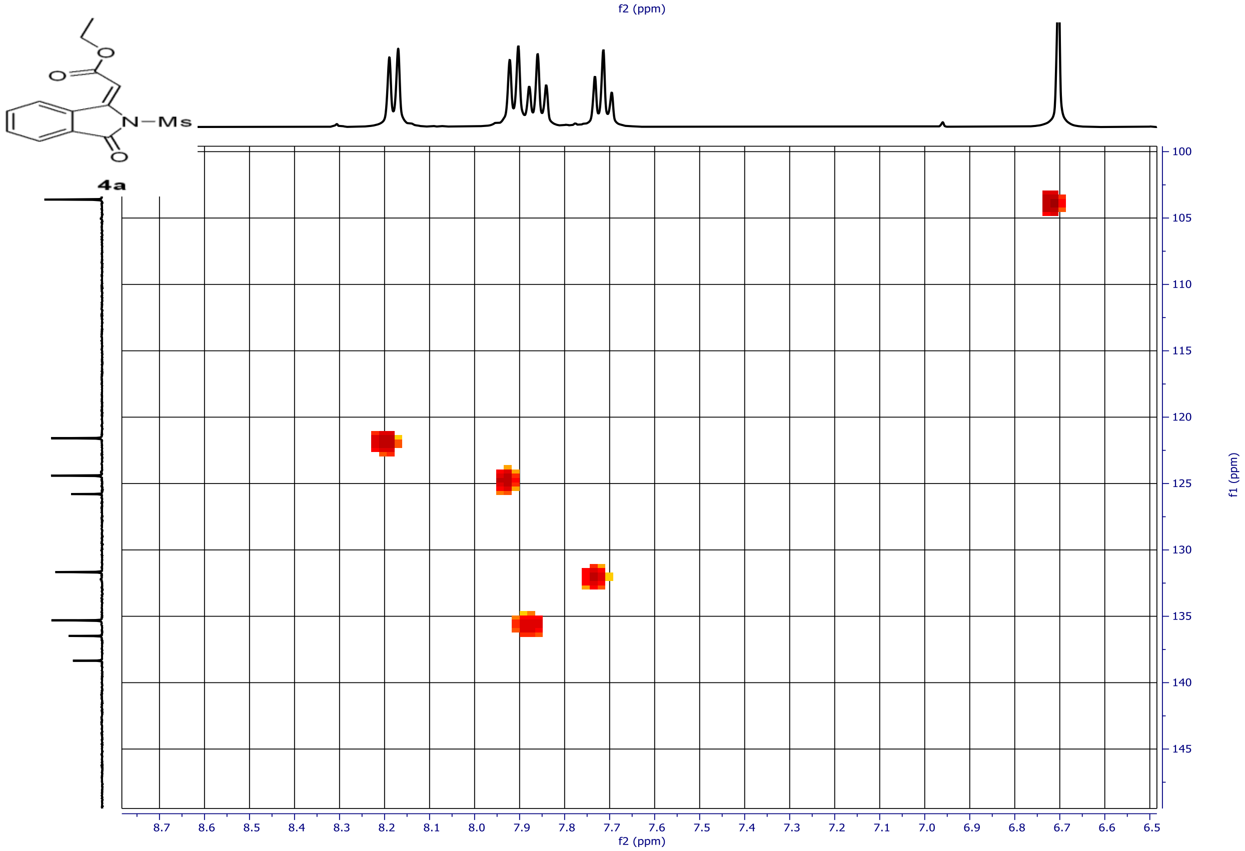

HMBC

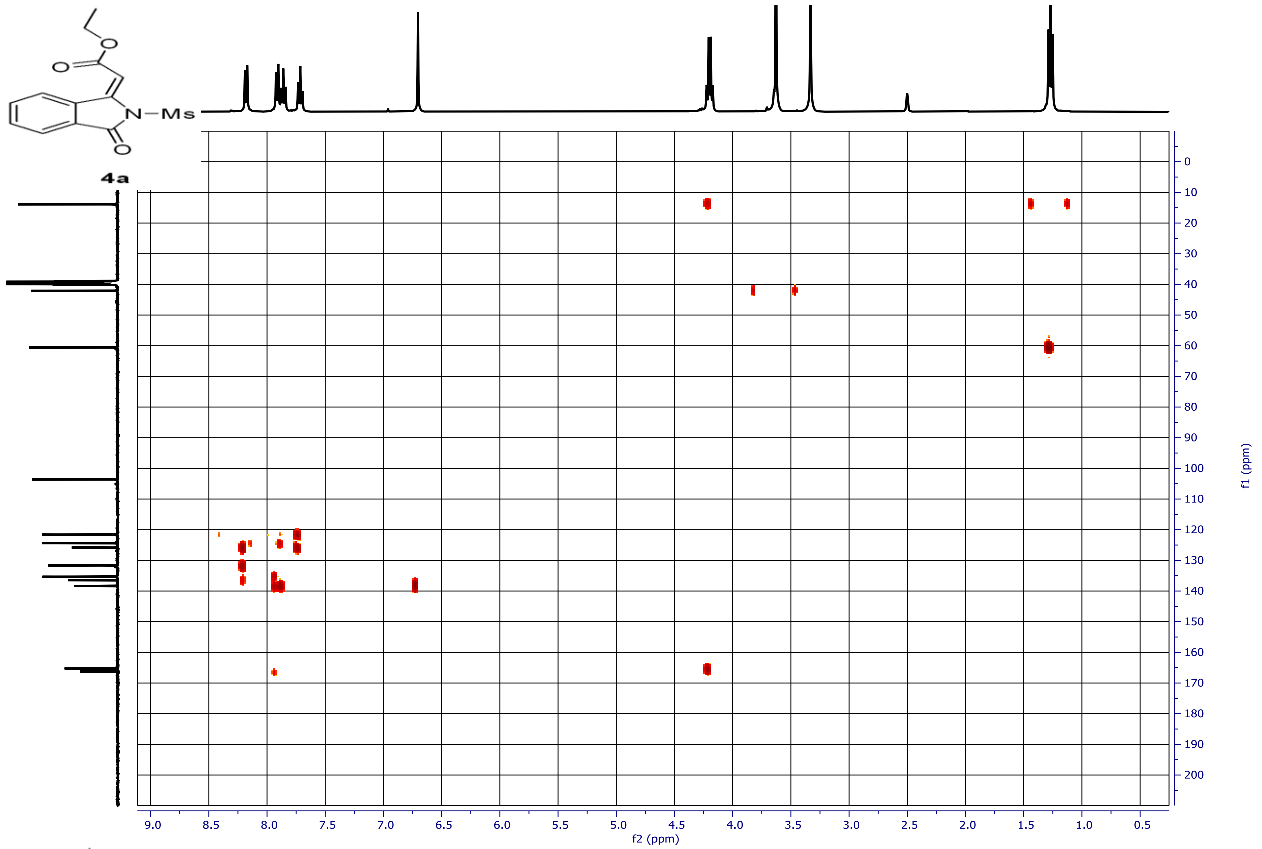

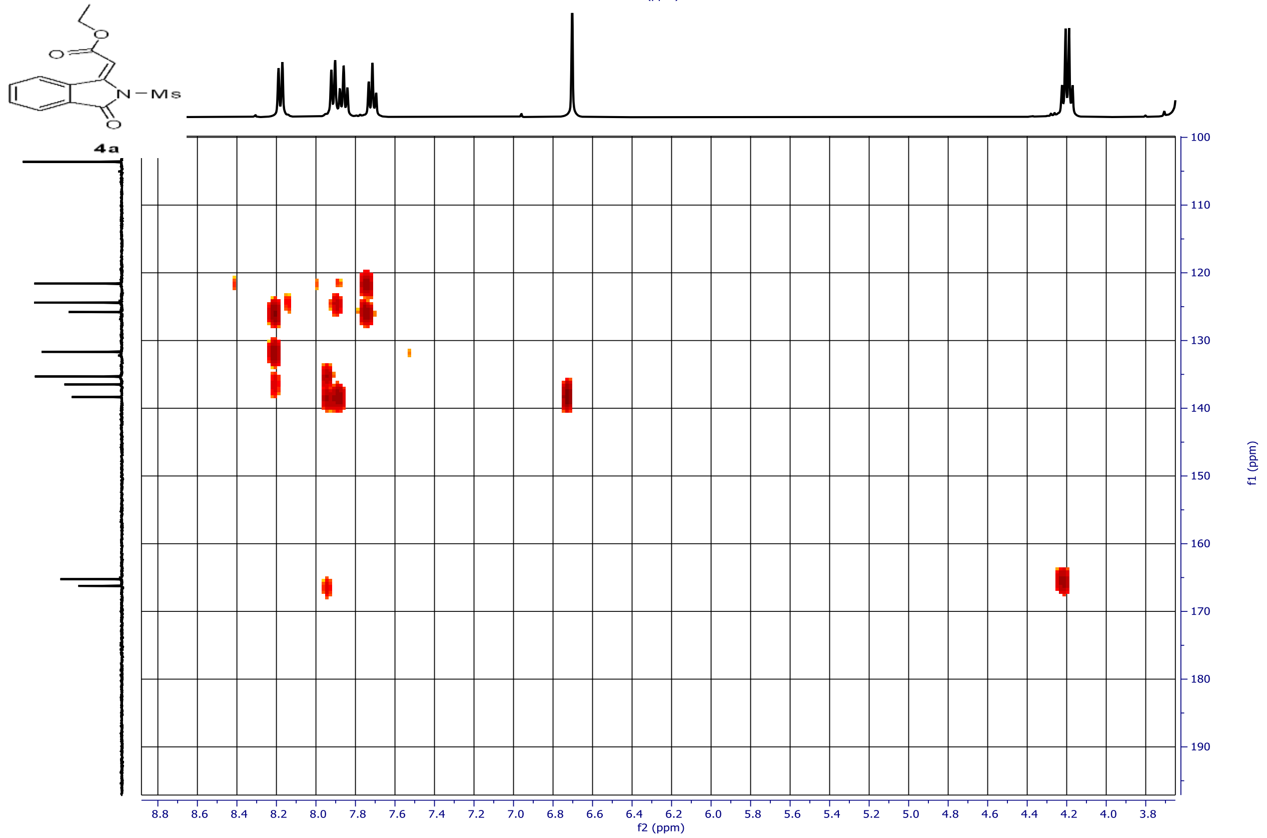

Ethyl (*E*)-2-(2-(methylsulfonyl)-3-oxoisoindolin-1-ylidene)acetate (4a)

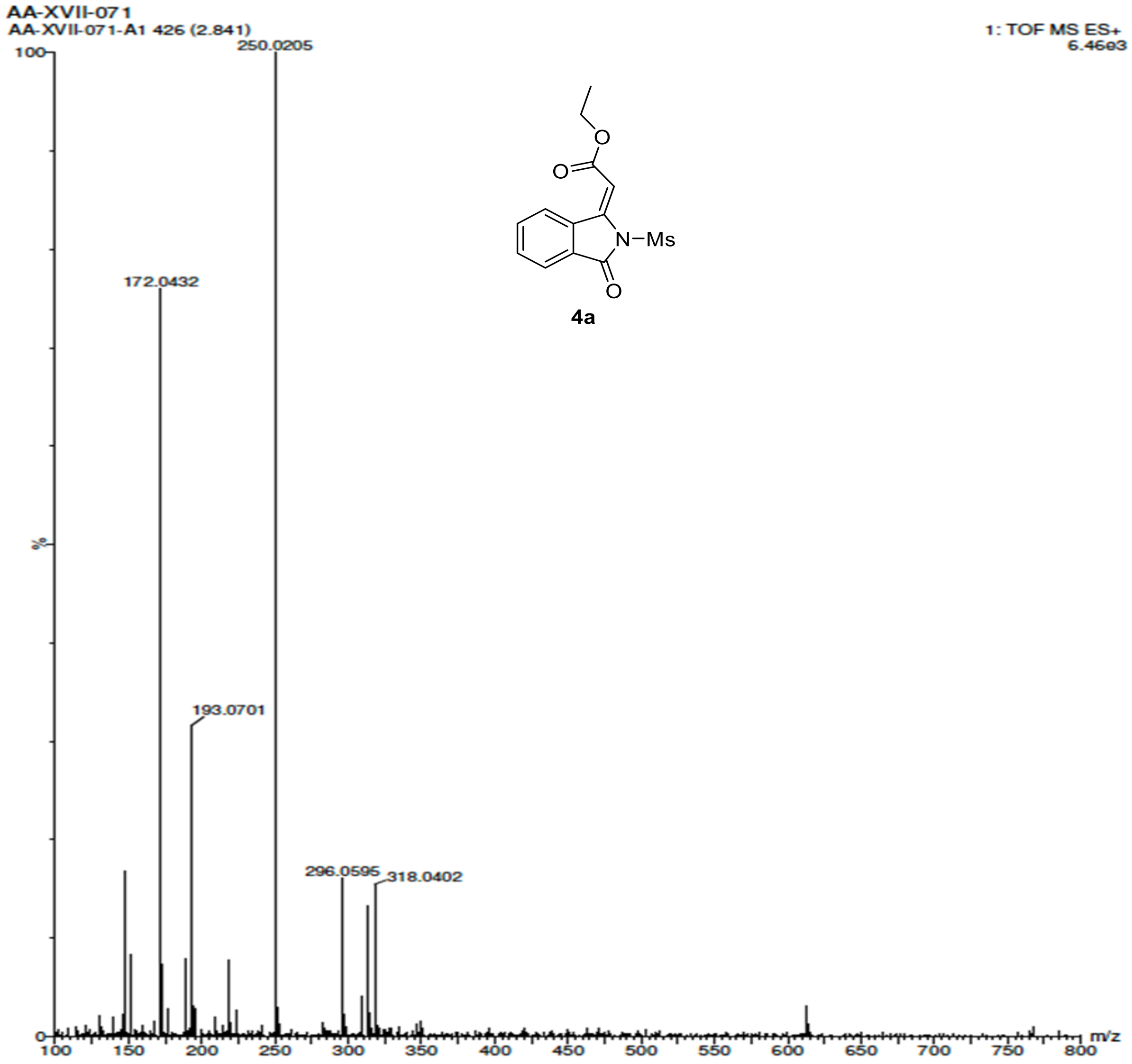

HRMS (ESI) *m/z* calcd for $C_{13}H_{13}NO_5S$ $[M + H]^+$ 296.0587; found 296.0595.

Ethyl (*E*)-2-(5-fluoro-2-(methylsulfonyl)-3-oxoisoindolin-1-ylidene)acetate (4b)

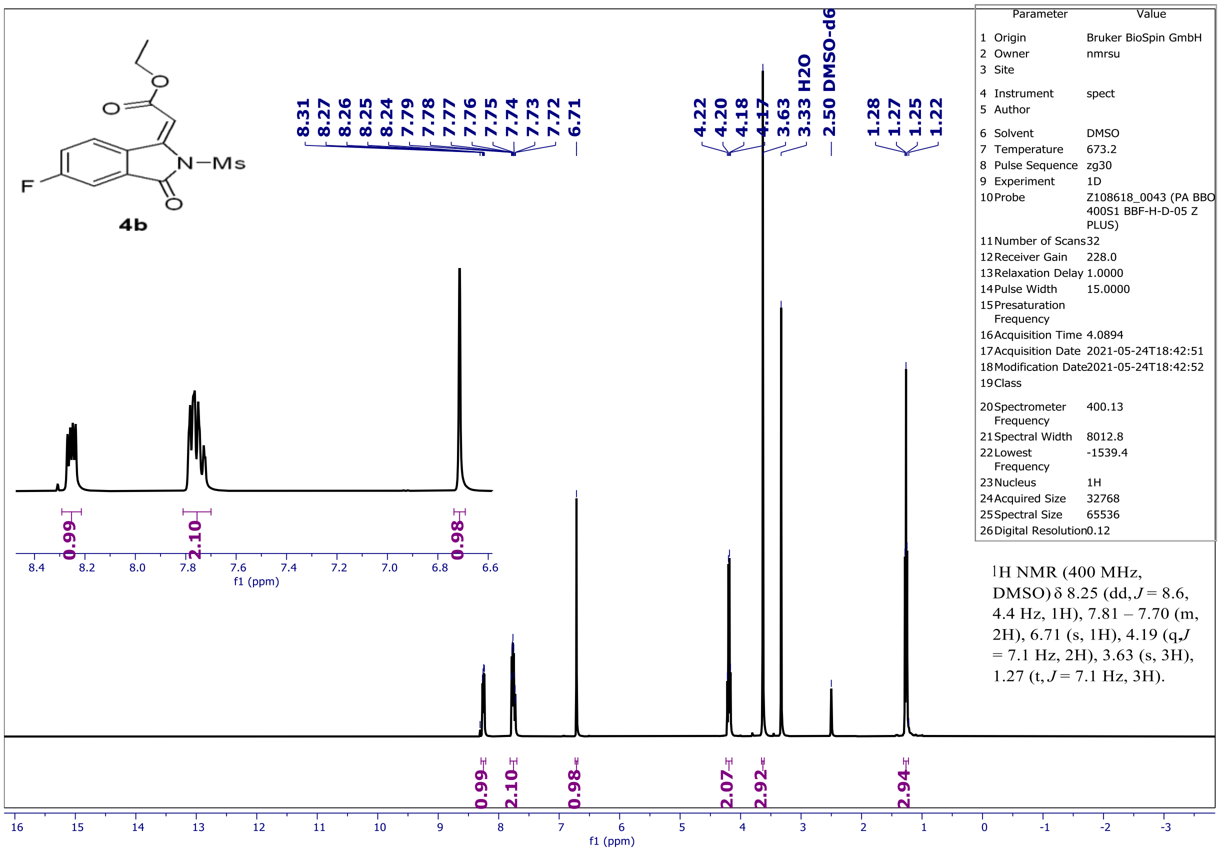

Parameter	Value
1 Origin	Bruker BioSpin GmbH
2 Owner	nmrsu
3 Site	
4 Instrument	spect
5 Author	
6 Solvent	DMSO
7 Temperature	673.2
8 Pulse Sequence	zg30
9 Experiment	1D
10 Probe	Z108618_0043 (PA BBO 400S1 BBF-H-D-05 Z PLUS)
11 Number of Scans	32
12 Receiver Gain	228.0
13 Relaxation Delay	1.0000
14 Pulse Width	15.0000
15 Presaturation Frequency	
16 Acquisition Time	4.0894
17 Acquisition Date	2021-05-24T18:42:51
18 Modification Date	2021-05-24T18:42:52
19 Class	
20 Spectrometer Frequency	400.13
21 Spectral Width	8012.8
22 Lowest Frequency	-1539.4
23 Nucleus	1H
24 Acquired Size	32768
25 Spectral Size	65536
26 Digital Resolution	0.12

^{1}H NMR (400 MHz, DMSO) δ 8.25 (dd, *J* = 8.6, 4.4 Hz, 1H), 7.81 – 7.70 (m, 2H), 6.71 (s, 1H), 4.19 (q, *J* = 7.1 Hz, 2H), 3.63 (s, 3H), 1.27 (t, *J* = 7.1 Hz, 3H).

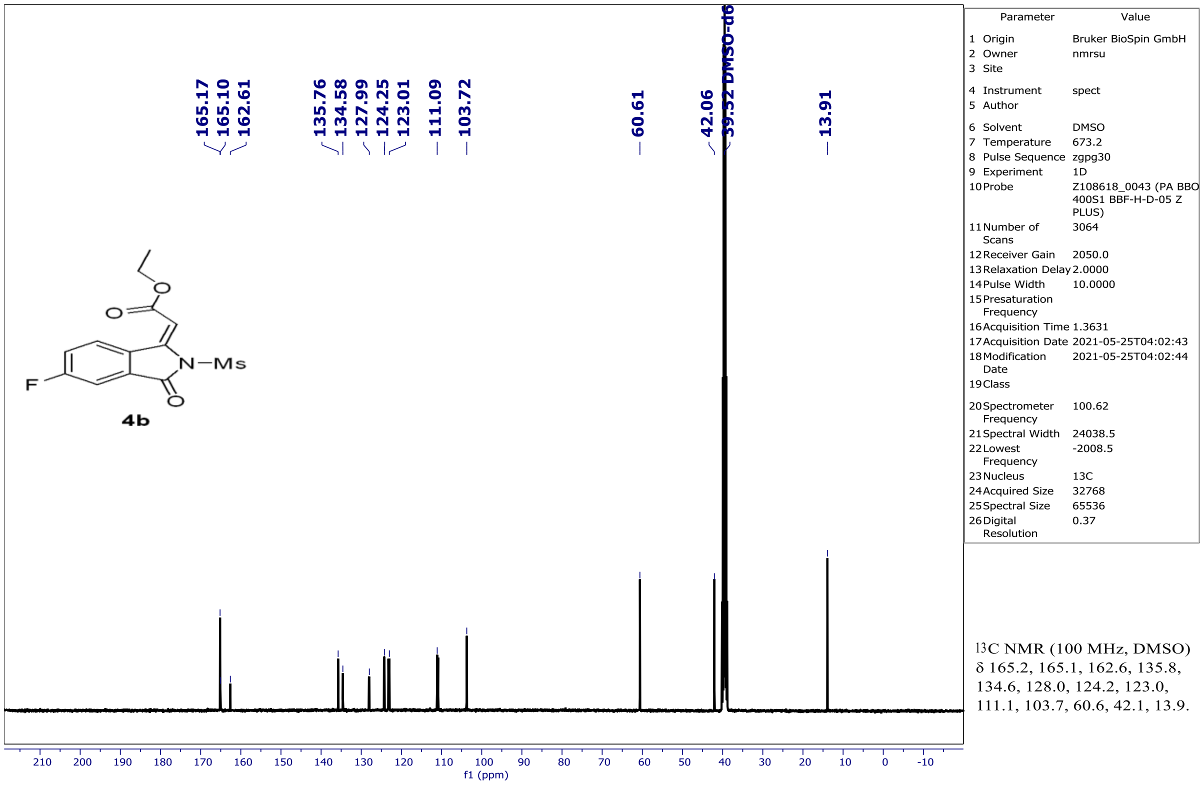

Parameter	Value
1 Origin	Bruker BioSpin GmbH
2 Owner	nmrsu
3 Site	
4 Instrument	spect
5 Author	
6 Solvent	DMSO
7 Temperature	673.2
8 Pulse Sequence	zgpg30
9 Experiment	1D
10 Probe	Z108618_0043 (PA BBO 400S1 BBF-H-D-05 Z PLUS)
11 Number of Scans	3064
12 Receiver Gain	2050.0
13 Relaxation Delay	2.0000
14 Pulse Width	10.0000
15 Presaturation Frequency	
16 Acquisition Time	1.3631
17 Acquisition Date	2021-05-25T04:02:43
18 Modification Date	2021-05-25T04:02:44
19 Class	
20 Spectrometer Frequency	100.62
21 Spectral Width	24038.5
22 Lowest Frequency	-2008.5
23 Nucleus	13C
24 Acquired Size	32768
25 Spectral Size	65536
26 Digital Resolution	0.37

^{13}C NMR (100 MHz, DMSO) δ 165.2, 165.1, 162.6, 135.8, 134.6, 128.0, 124.2, 123.0, 111.1, 103.7, 60.6, 42.1, 13.9.

Ethyl (*E*)-2-(5-fluoro-2-(methylsulfonyl)-3-oxoisoindolin-1-ylidene)acetate (4b)

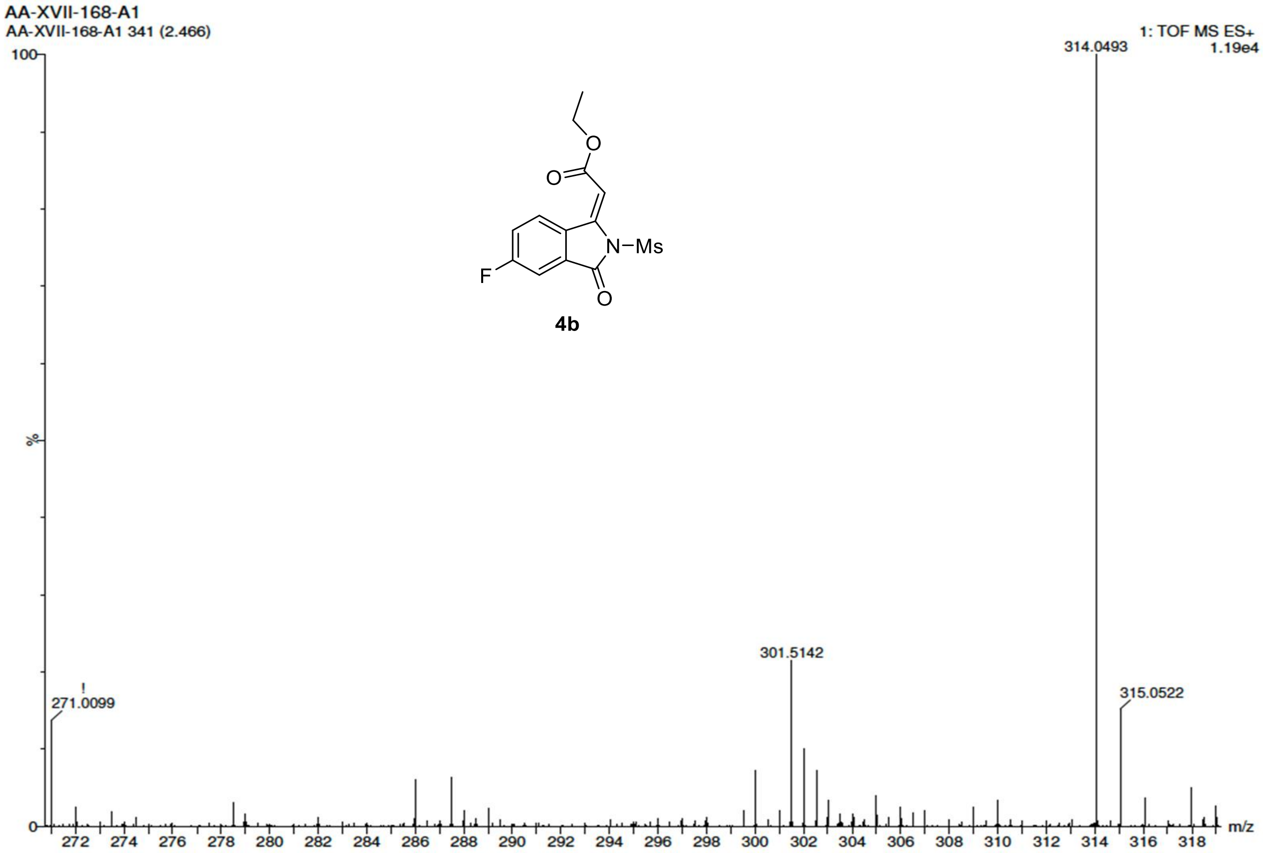

HRMS (ESI) *m/z* calcd for $C_{13}H_{12}FNO_5S$ $[M + H]^+$ 314.0493; found 314.0493.

Ethyl (*E*)-2-(5-chloro-2-(methylsulfonyl)-3-oxoisoindolin-1-ylidene)acetate (4c)

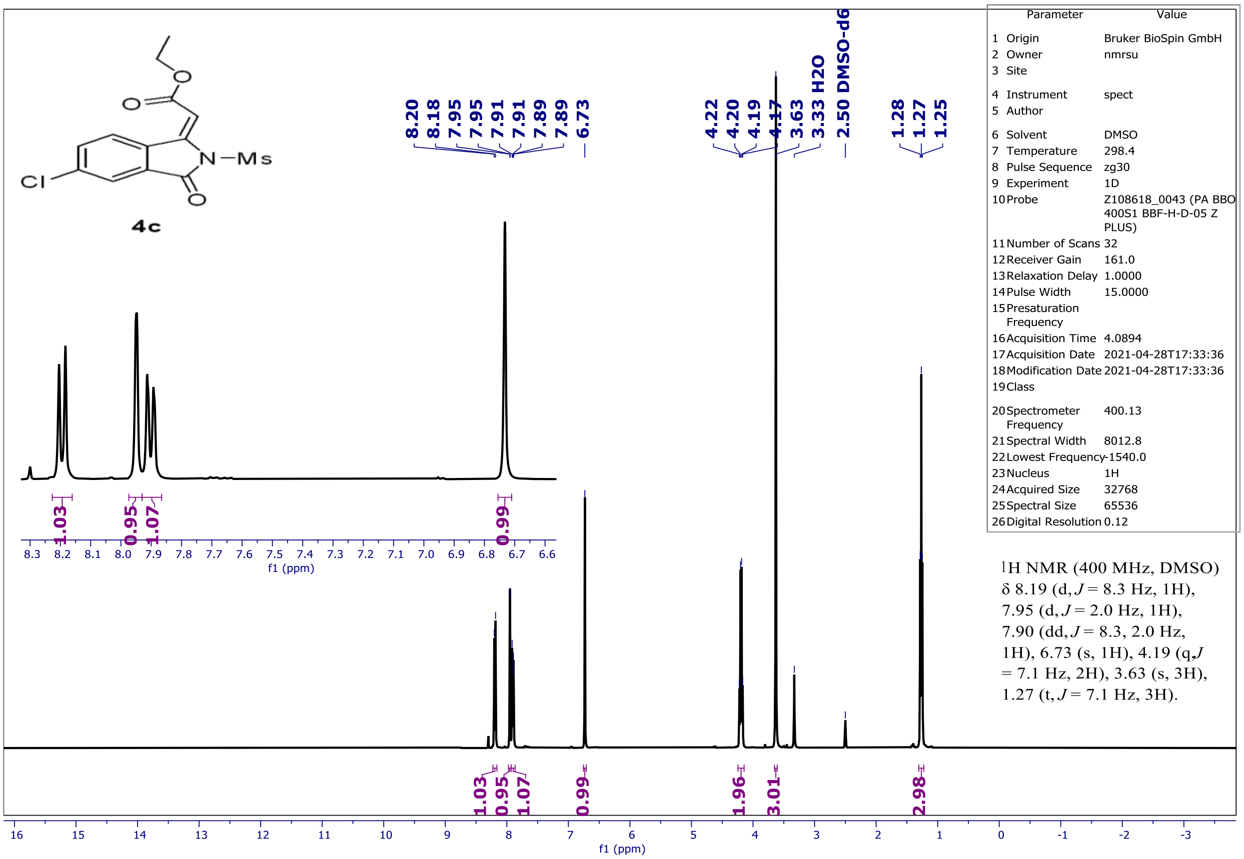

Parameter		Value
1	Origin	Bruker BioSpin GmbH
2	Owner	nmrsu
3	Site	
4	Instrument	spect
5	Author	
6	Solvent	DMSO
7	Temperature	298.4
8	Pulse Sequence	zg30
9	Experiment	1D
10	Probe	Z108618_0043 (PA BBO 400S1 BBF-H-D-05 Z PLUS)
11	Number of Scans	32
12	Receiver Gain	161.0
13	Relaxation Delay	1.0000
14	Pulse Width	15.0000
15	Presaturation Frequency	
16	Acquisition Time	4.0894
17	Acquisition Date	2021-04-28T17:33:36
18	Modification Date	2021-04-28T17:33:36
19	Class	
20	Spectrometer Frequency	400.13
21	Spectral Width	8012.8
22	Lowest Frequency	-1540.0
23	Nucleus	1H
24	Acquired Size	32768
25	Spectral Size	65536
26	Digital Resolution	0.12

^{1}H NMR (400 MHz, DMSO) δ 8.19 (d, *J* = 8.3 Hz, 1H), 7.95 (d, *J* = 2.0 Hz, 1H), 7.90 (dd, *J* = 8.3, 2.0 Hz, 1H), 6.73 (s, 1H), 4.19 (q, *J* = 7.1 Hz, 2H), 3.63 (s, 3H), 1.27 (t, *J* = 7.1 Hz, 3H).

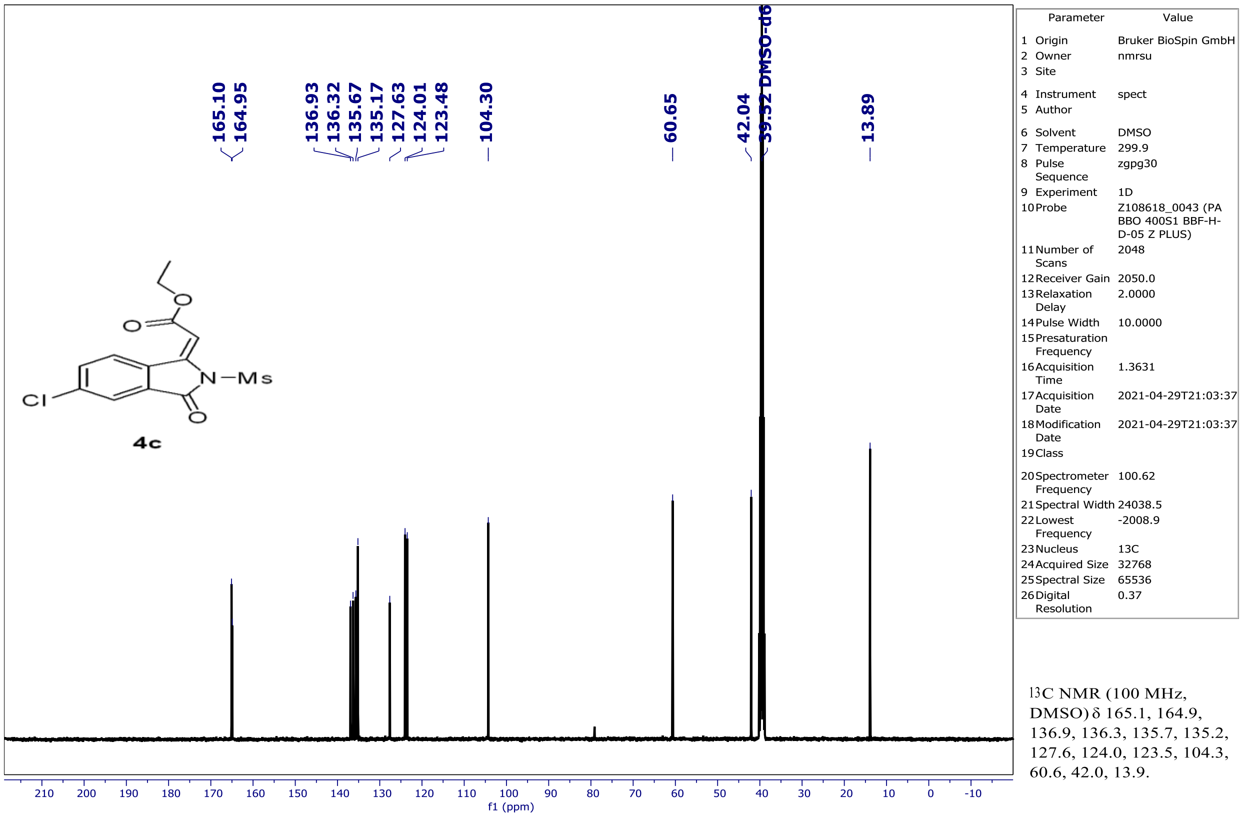

Parameter		Value
1	Origin	Bruker BioSpin GmbH
2	Owner	nmrsu
3	Site	
4	Instrument	spect
5	Author	
6	Solvent	DMSO
7	Temperature	299.9
8	Pulse Sequence	zgpg30
9	Experiment	1D
10	Probe	Z108618_0043 (PA BBO 400S1 BBF-H-D-05 Z PLUS)
11	Number of Scans	2048
12	Receiver Gain	2050.0
13	Relaxation Delay	2.0000
14	Pulse Width	10.0000
15	Presaturation Frequency	
16	Acquisition Time	1.3631
17	Acquisition Date	2021-04-29T21:03:37
18	Modification Date	2021-04-29T21:03:37
19	Class	
20	Spectrometer Frequency	100.62
21	Spectral Width	24038.5
22	Lowest Frequency	-2008.9
23	Nucleus	13C
24	Acquired Size	32768
25	Spectral Size	65536
26	Digital Resolution	0.37

^{13}C NMR (100 MHz, DMSO) δ 165.1, 164.9, 136.9, 136.3, 135.7, 135.2, 127.6, 124.0, 123.5, 104.3, 60.6, 42.0, 13.9.

Ethyl (*E*)-2-(5-chloro-2-(methylsulfonyl)-3-oxoisoindolin-1-ylidene)acetate (4c)

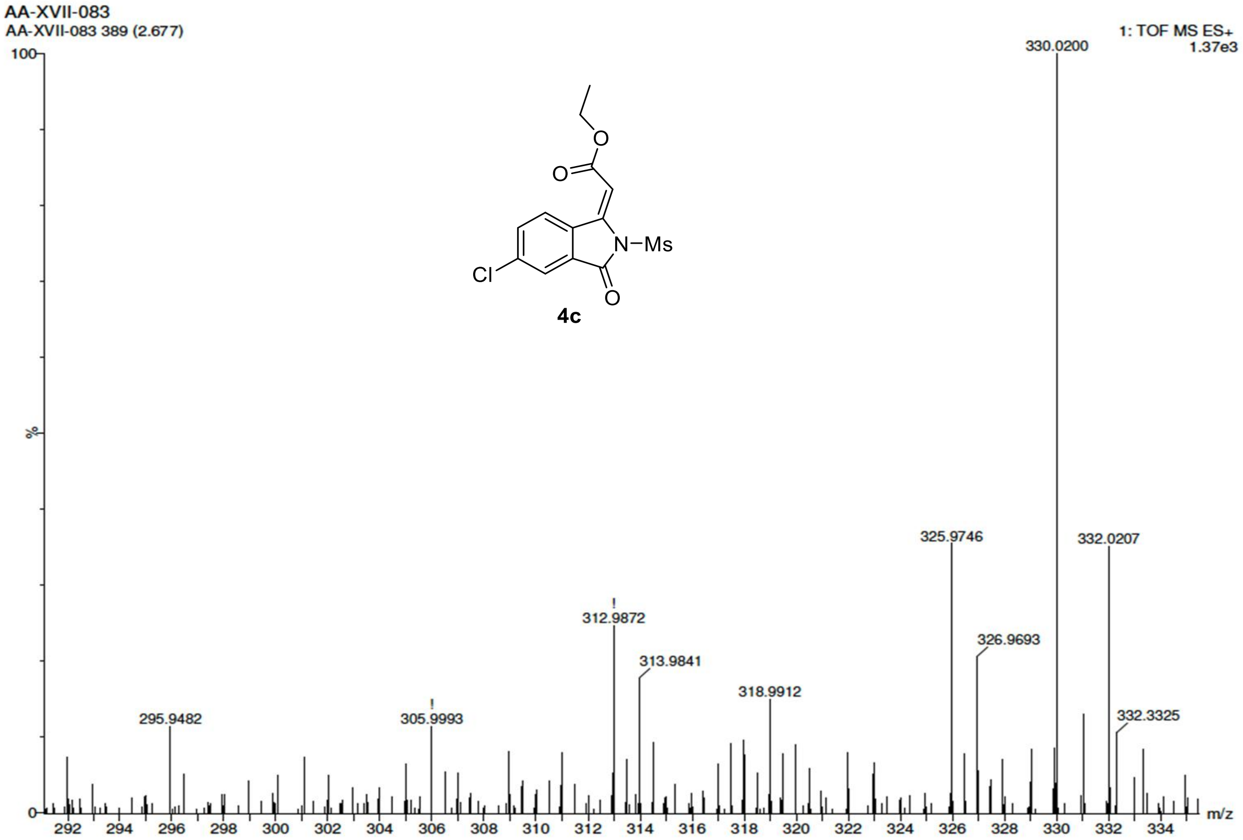

HRMS (ESI) *m/z* calcd for $C_{13}H_{12}ClNO_5S$ $[M + H]^+$ 330.0197; found 330.0200.

Ethyl (*E*)-2-(5-bromo-2-(methylsulfonyl)-3-oxoisoindolin-1-ylidene)acetate (4d)

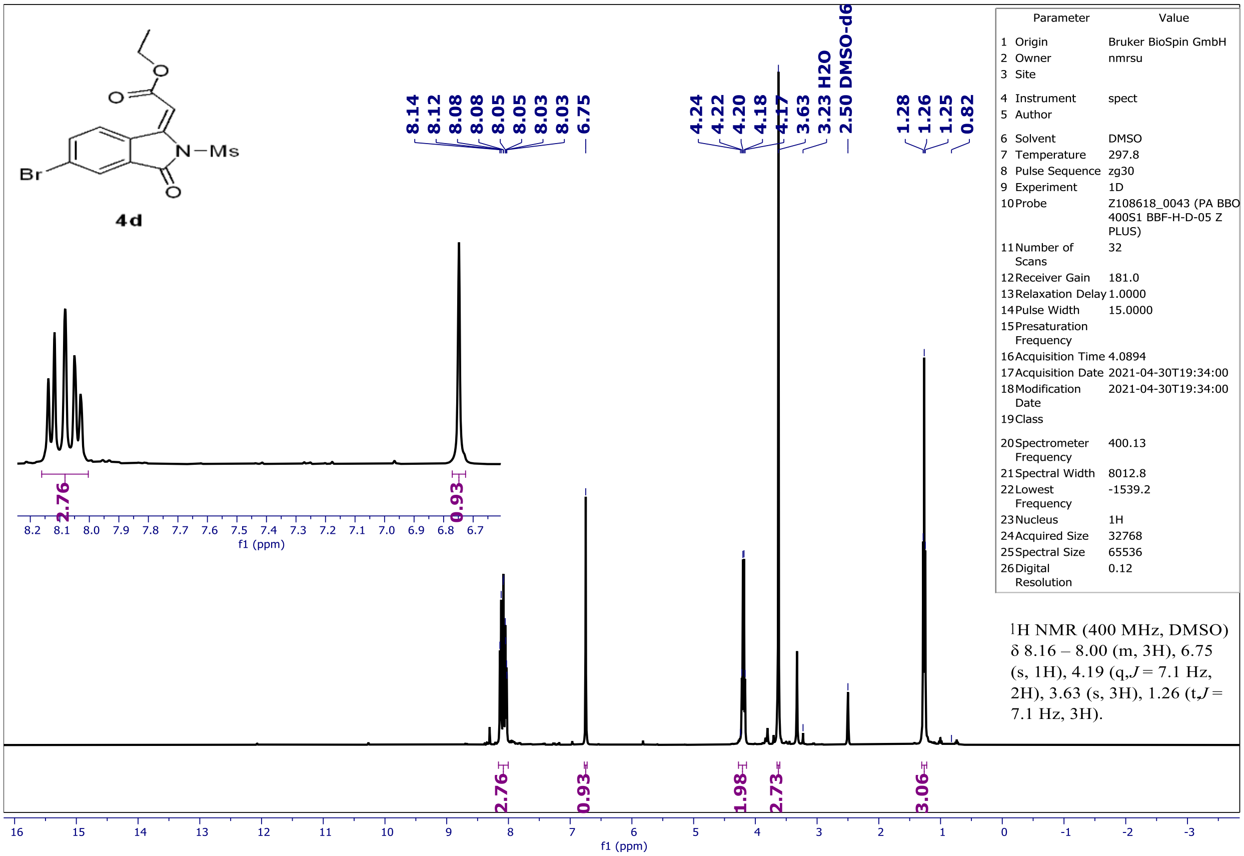

1H NMR (400 MHz, DMSO) δ 8.16 – 8.00 (m, 3H), 6.75 (s, 1H), 4.19 (q, *J* = 7.1 Hz, 2H), 3.63 (s, 3H), 1.26 (t, *J* = 7.1 Hz, 3H).

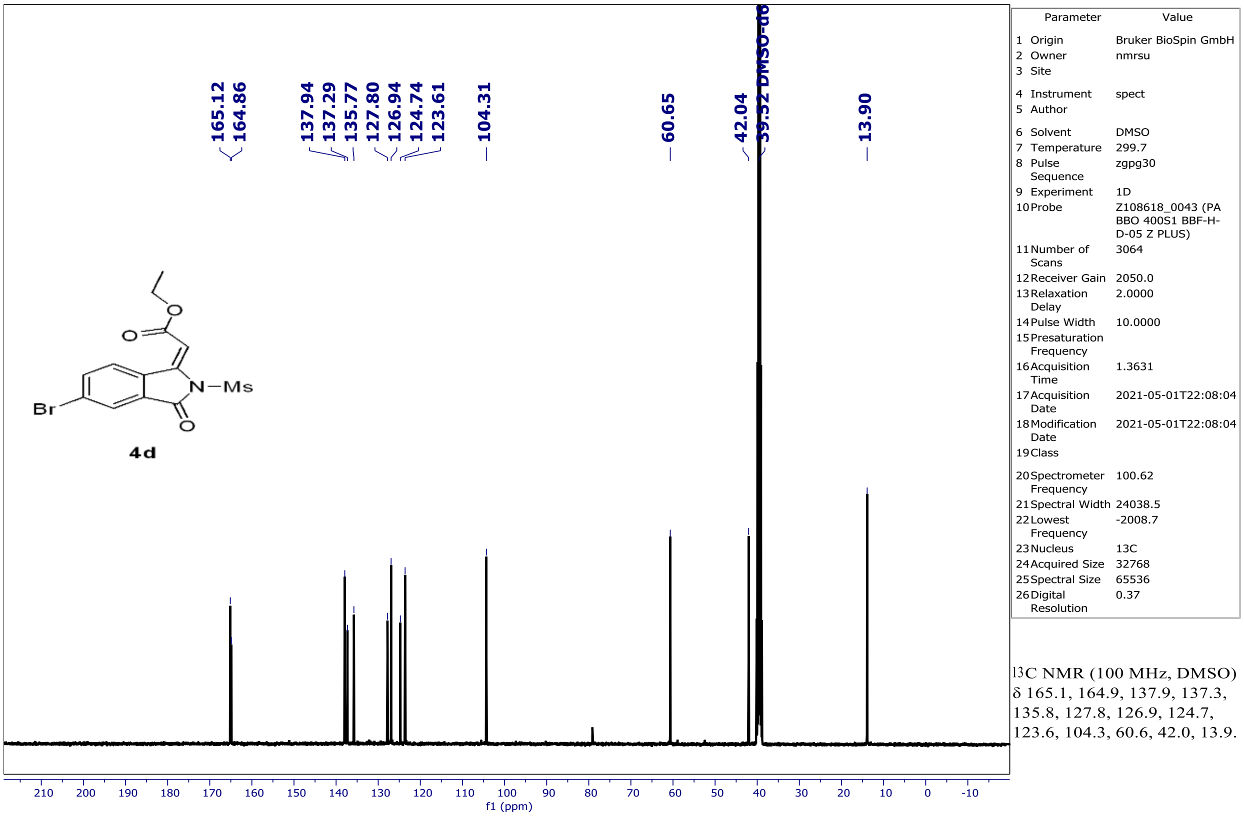

13C NMR (100 MHz, DMSO) δ 165.1, 164.9, 137.9, 137.3, 135.8, 127.8, 126.9, 124.7, 123.6, 104.3, 60.6, 42.0, 13.9.

Ethyl (*E*)-2-(5-bromo-2-(methylsulfonyl)-3-oxoisoindolin-1-ylidene)acetate (4d)

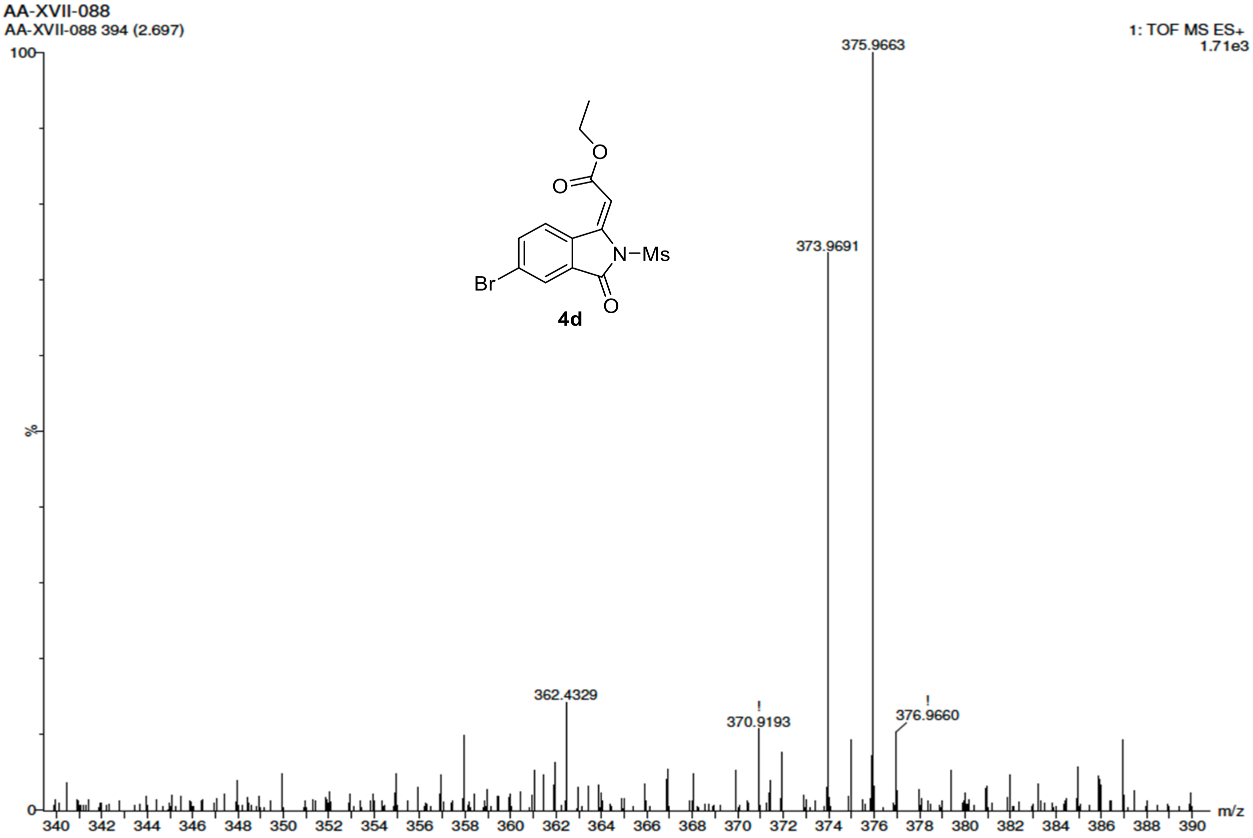

HRMS (ESI) *m/z* calcd for $C_{13}H_{12}BrNO_5S$ $[M + H]^+$ 373.9692; found 373.9691.

Ethyl (*E*)-2-(5-methyl-2-(methylsulfonyl)-3-oxoisoindolin-1-ylidene)acetate (4e)

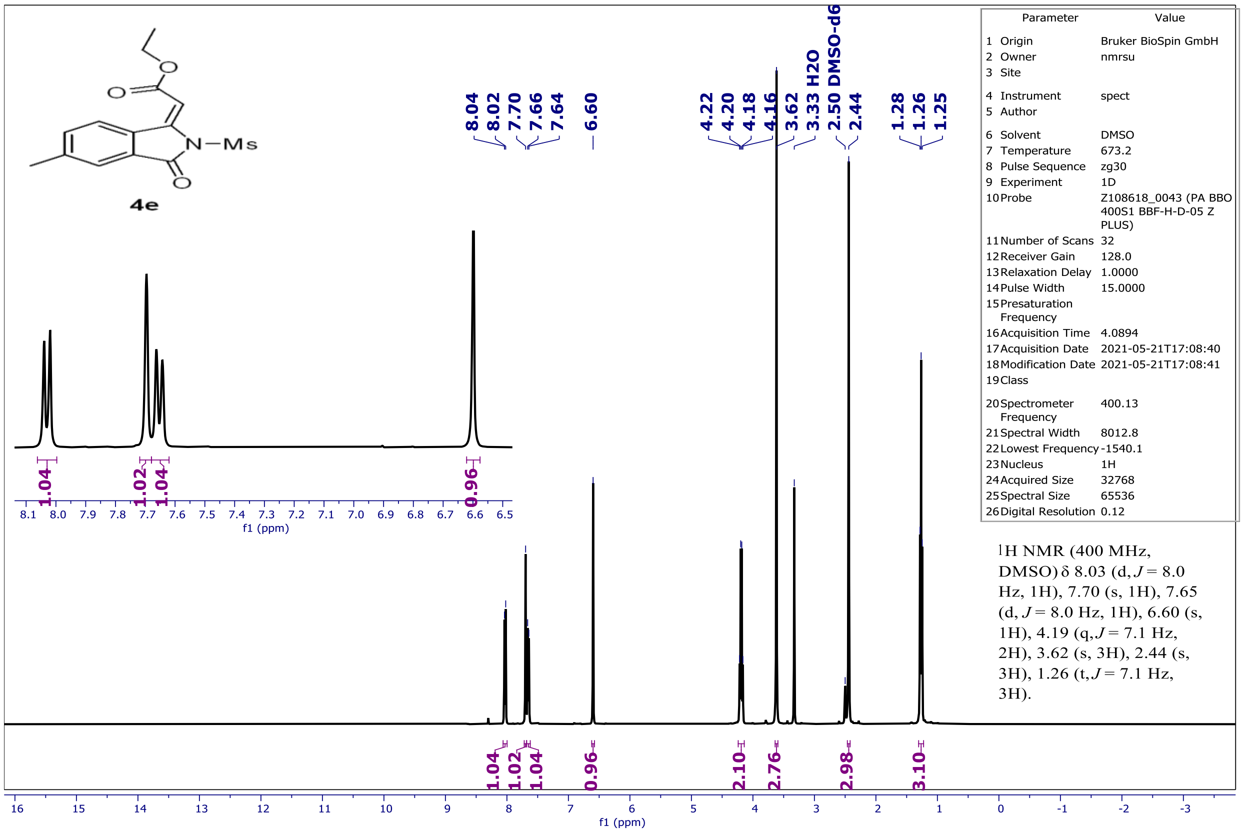

Parameter	Value
1 Origin	Bruker BioSpin GmbH
2 Owner	nmrsu
3 Site	
4 Instrument	spect
5 Author	
6 Solvent	DMSO
7 Temperature	673.2
8 Pulse Sequence	zg30
9 Experiment	1D
10 Probe	Z108618_0043 (PA BBO 400S1 BBF-H-D-05 Z PLUS)
11 Number of Scans	32
12 Receiver Gain	128.0
13 Relaxation Delay	1.0000
14 Pulse Width	15.0000
15 Presaturation Frequency	
16 Acquisition Time	4.0894
17 Acquisition Date	2021-05-21T17:08:40
18 Modification Date	2021-05-21T17:08:41
19 Class	
20 Spectrometer Frequency	400.13
21 Spectral Width	8012.8
22 Lowest Frequency	-1540.1
23 Nucleus	1H
24 Acquired Size	32768
25 Spectral Size	65536
26 Digital Resolution	0.12

^{1}H NMR (400 MHz, DMSO) δ 8.03 (d, *J* = 8.0 Hz, 1H), 7.70 (s, 1H), 7.65 (d, *J* = 8.0 Hz, 1H), 6.60 (s, 1H), 4.19 (q, *J* = 7.1 Hz, 2H), 3.62 (s, 3H), 2.44 (s, 3H), 1.26 (t, *J* = 7.1 Hz, 3H).

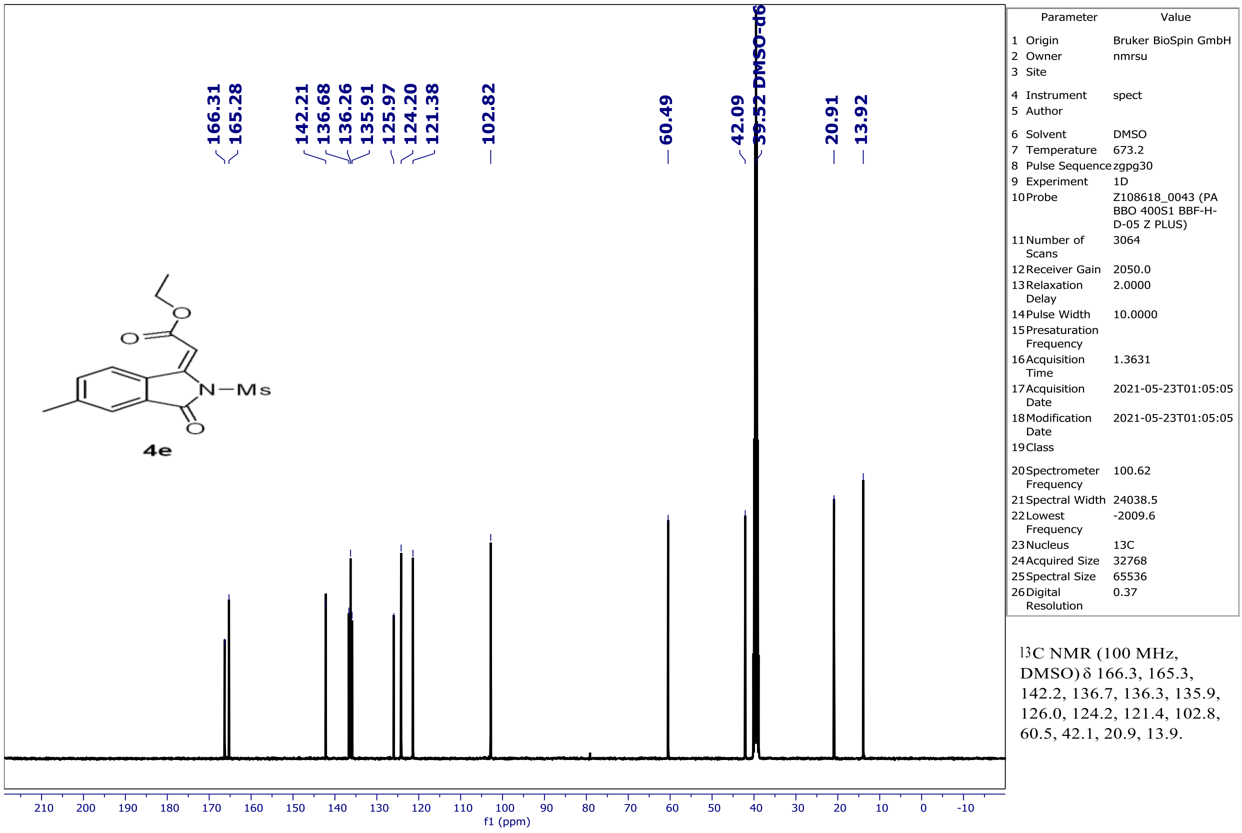

Parameter	Value
1 Origin	Bruker BioSpin GmbH
2 Owner	nmrsu
3 Site	
4 Instrument	spect
5 Author	
6 Solvent	DMSO
7 Temperature	673.2
8 Pulse Sequence	zgpg30
9 Experiment	1D
10 Probe	Z108618_0043 (PA BBO 400S1 BBF-H-D-05 Z PLUS)
11 Number of Scans	3064
12 Receiver Gain	2050.0
13 Relaxation Delay	2.0000
14 Pulse Width	10.0000
15 Presaturation Frequency	
16 Acquisition Time	1.3631
17 Acquisition Date	2021-05-23T01:05:05
18 Modification Date	2021-05-23T01:05:05
19 Class	
20 Spectrometer Frequency	100.62
21 Spectral Width	24038.5
22 Lowest Frequency	-2009.6
23 Nucleus	13C
24 Acquired Size	32768
25 Spectral Size	65536
26 Digital Resolution	0.37

^{13}C NMR (100 MHz, DMSO) δ 166.3, 165.3, 142.2, 136.7, 136.3, 135.9, 126.0, 124.2, 121.4, 102.8, 60.5, 42.1, 20.9, 13.9.

Ethyl (*E*)-2-(5-methyl-2-(methylsulfonyl)-3-oxoisoindolin-1-ylidene)acetate (4e)

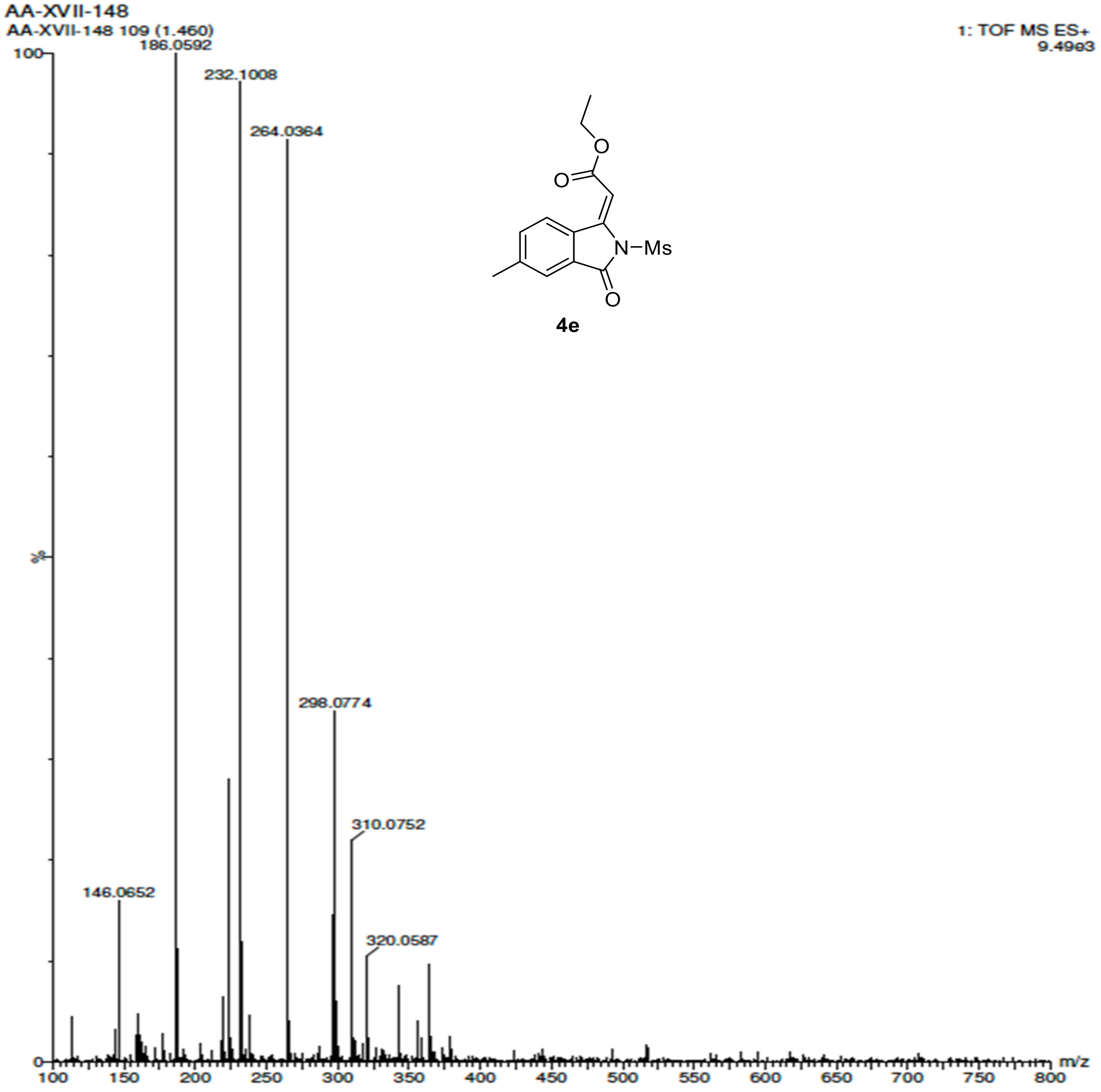

HRMS (ESI) *m/z* calcd for $C_{14}H_{15}NO_5S$ $[M + H]^+$ 310.0744; found 310.0752.

Ethyl (*E*)-2-(5-methoxy-2-(methylsulfonyl)-3-oxoisoindolin-1-ylidene)acetate (4f)

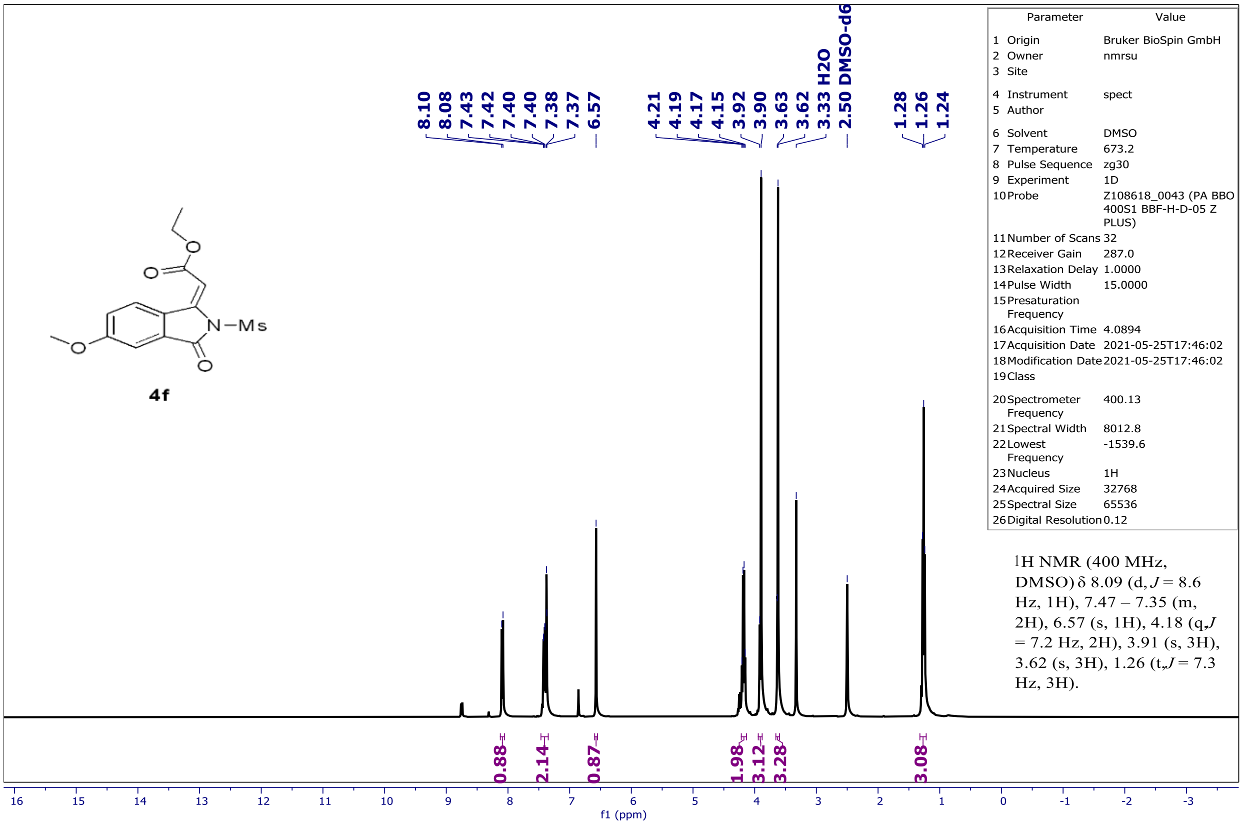

Parameter	Value
1 Origin	Bruker BioSpin GmbH
2 Owner	nmrsu
3 Site	
4 Instrument	spect
5 Author	
6 Solvent	DMSO
7 Temperature	673.2
8 Pulse Sequence	zg30
9 Experiment	1D
10 Probe	Z108618_0043 (PA BBO 400S1 BBF-H-D-05 Z PLUS)
11 Number of Scans	32
12 Receiver Gain	287.0
13 Relaxation Delay	1.0000
14 Pulse Width	15.0000
15 Presaturation Frequency	
16 Acquisition Time	4.0894
17 Acquisition Date	2021-05-25T17:46:02
18 Modification Date	2021-05-25T17:46:02
19 Class	
20 Spectrometer Frequency	400.13
21 Spectral Width	8012.8
22 Lowest Frequency	-1539.6
23 Nucleus	1H
24 Acquired Size	32768
25 Spectral Size	65536
26 Digital Resolution	0.12

^{1}H NMR (400 MHz, DMSO) δ 8.09 (d, *J* = 8.6 Hz, 1H), 7.47 – 7.35 (m, 2H), 6.57 (s, 1H), 4.18 (q, *J* = 7.2 Hz, 2H), 3.91 (s, 3H), 3.62 (s, 3H), 1.26 (t, *J* = 7.3 Hz, 3H).

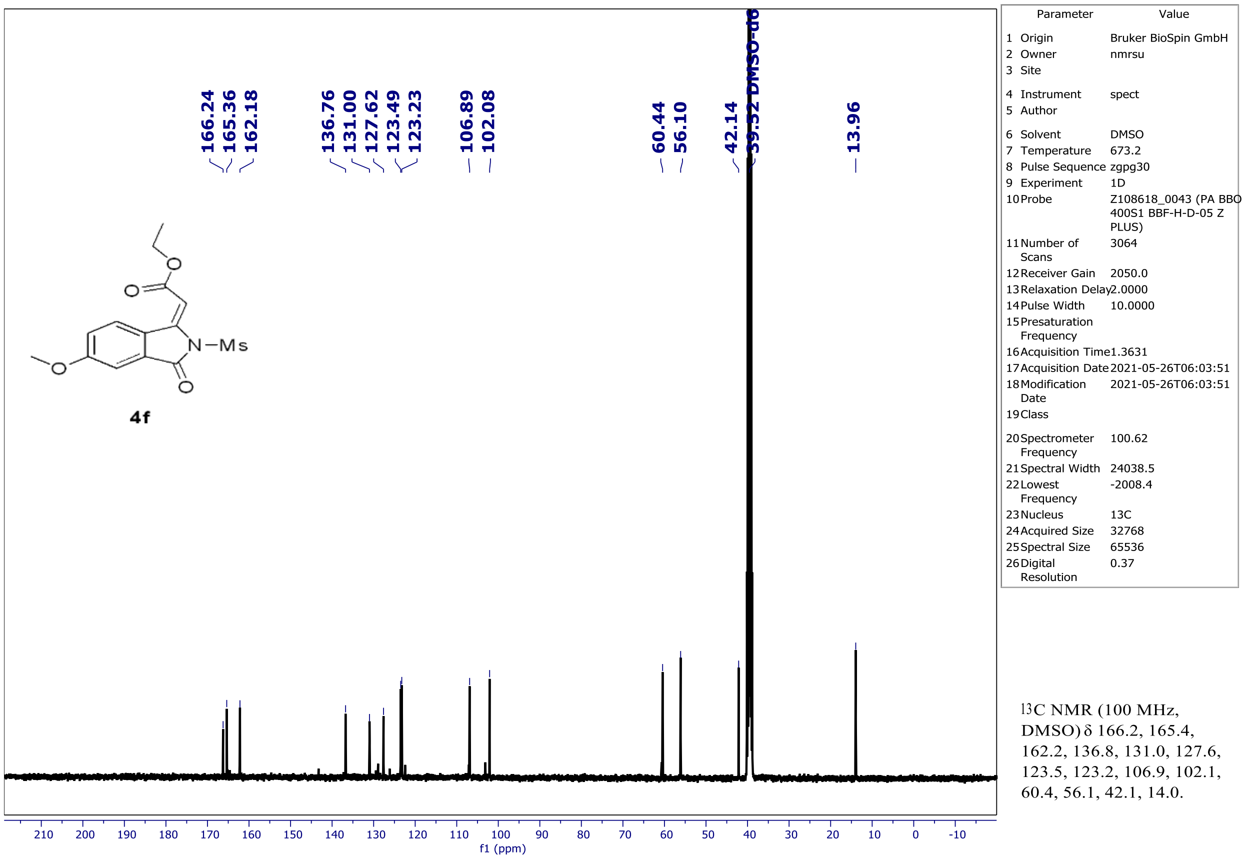

Parameter	Value
1 Origin	Bruker BioSpin GmbH
2 Owner	nmrsu
3 Site	
4 Instrument	spect
5 Author	
6 Solvent	DMSO
7 Temperature	673.2
8 Pulse Sequence	zgpg30
9 Experiment	1D
10 Probe	Z108618_0043 (PA BBO 400S1 BBF-H-D-05 Z PLUS)
11 Number of Scans	3064
12 Receiver Gain	2050.0
13 Relaxation Delay	2.0000
14 Pulse Width	10.0000
15 Presaturation Frequency	
16 Acquisition Time	1.3631
17 Acquisition Date	2021-05-26T06:03:51
18 Modification Date	2021-05-26T06:03:51
19 Class	
20 Spectrometer Frequency	100.62
21 Spectral Width	24038.5
22 Lowest Frequency	-2008.4
23 Nucleus	13C
24 Acquired Size	32768
25 Spectral Size	65536
26 Digital Resolution	0.37

^{13}C NMR (100 MHz, DMSO) δ 166.2, 165.4, 162.2, 136.8, 131.0, 127.6, 123.5, 123.2, 106.9, 102.1, 60.4, 56.1, 42.1, 14.0.

Ethyl (*E*)-2-(5-methoxy-2-(methylsulfonyl)-3-oxoisoindolin-1-ylidene)acetate (4f)

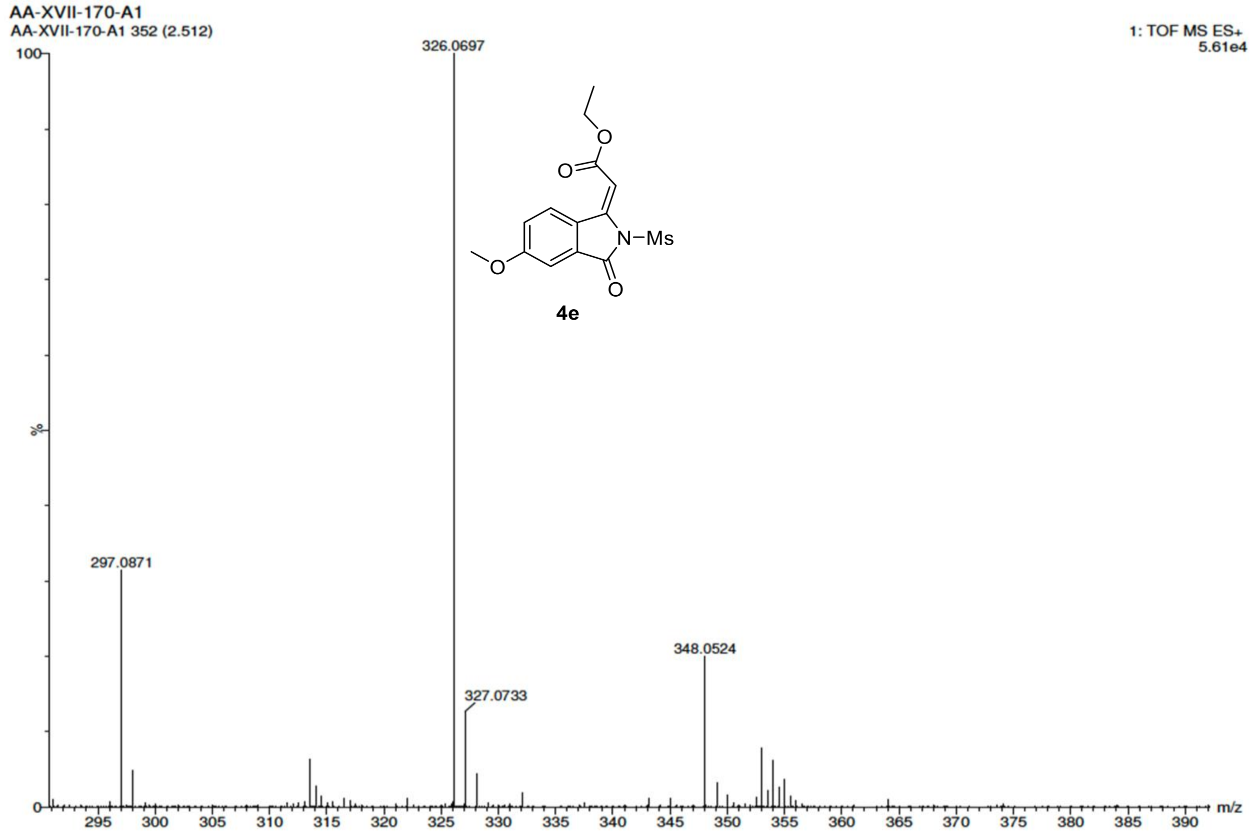

HRMS (ESI) *m/z* calcd for $C_{14}H_{15}NO_6S$ $[M + H]^+$ 326.0693; found 326.0697.

Ethyl (*E*)-2-(2-(methylsulfonyl)-5-nitro-3-oxoisoindolin-1-ylidene)acetate (4g)

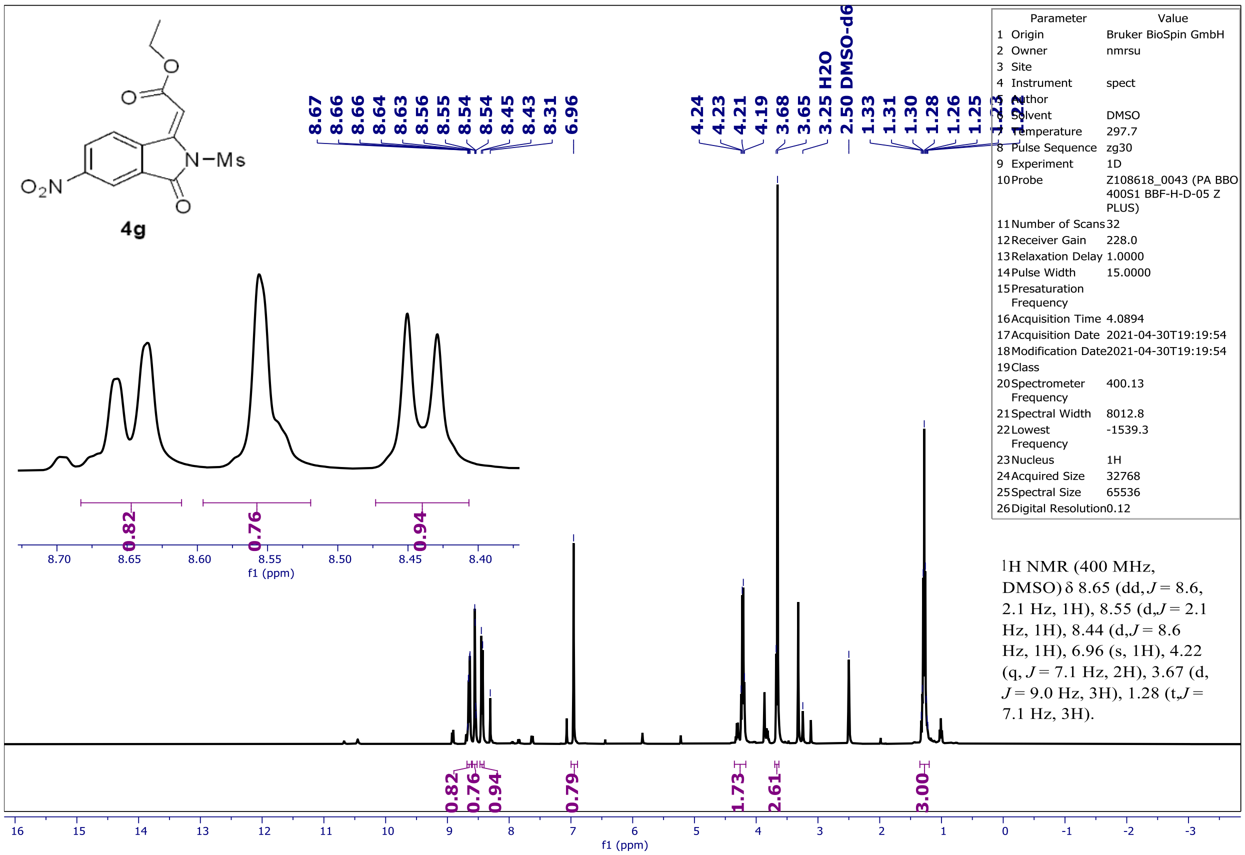

Parameter	Value
1 Origin	Bruker BioSpin GmbH
2 Owner	nmrsu
3 Site	
4 Instrument	spect
5 Author	
6 Solvent	DMSO
7 Temperature	297.7
8 Pulse Sequence	zg30
9 Experiment	1D
10 Probe	Z108618_0043 (PA BBO 400S1 BBF-H-D-05 Z PLUS)
11 Number of Scans	32
12 Receiver Gain	228.0
13 Relaxation Delay	1.0000
14 Pulse Width	15.0000
15 Presaturation Frequency	
16 Acquisition Time	4.0894
17 Acquisition Date	2021-04-30T19:19:54
18 Modification Date	2021-04-30T19:19:54
19 Class	
20 Spectrometer Frequency	400.13
21 Spectral Width	8012.8
22 Lowest Frequency	-1539.3
23 Nucleus	1H
24 Acquired Size	32768
25 Spectral Size	65536
26 Digital Resolution	0.12

^{1}H NMR (400 MHz, DMSO) δ 8.65 (dd, *J* = 8.6, 2.1 Hz, 1H), 8.55 (d, *J* = 2.1 Hz, 1H), 8.44 (d, *J* = 8.6 Hz, 1H), 6.96 (s, 1H), 4.22 (q, *J* = 7.1 Hz, 2H), 3.67 (d, *J* = 9.0 Hz, 3H), 1.28 (t, *J* = 7.1 Hz, 3H).

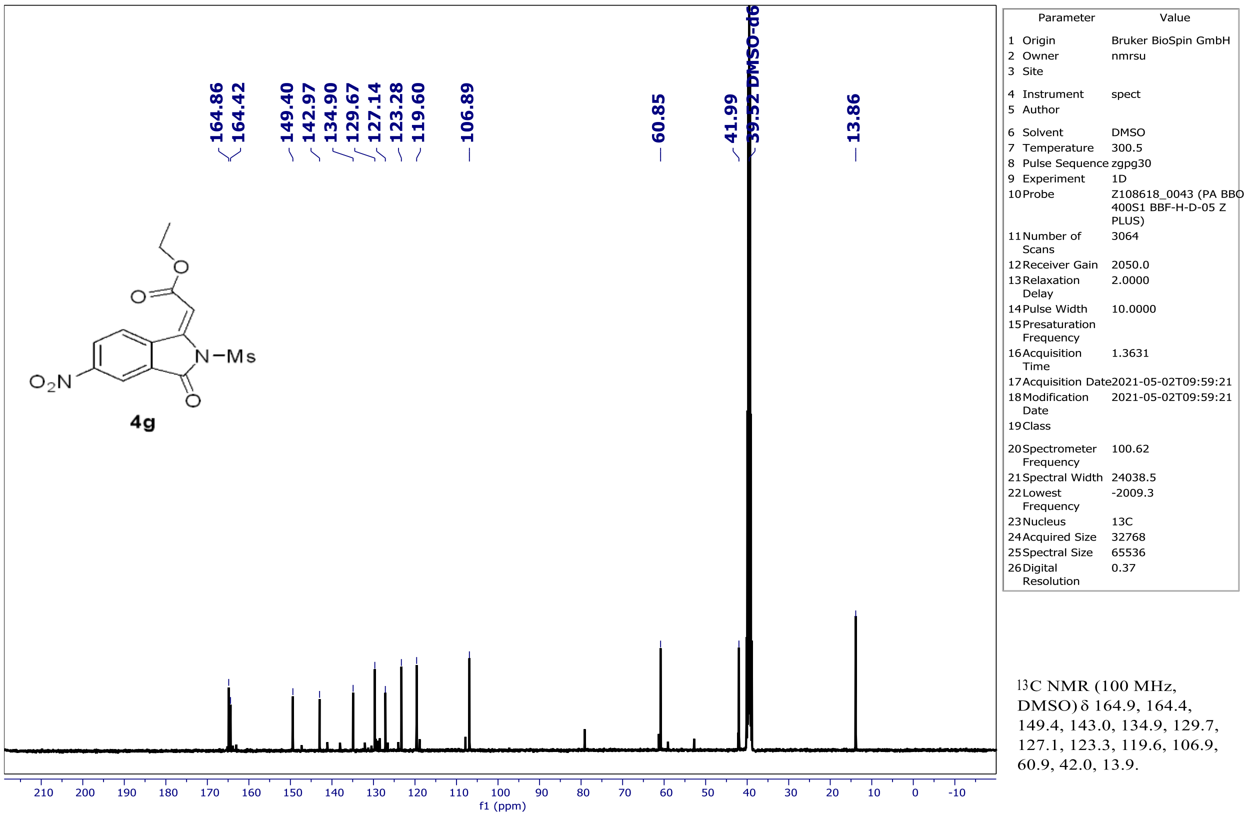

Parameter	Value
1 Origin	Bruker BioSpin GmbH
2 Owner	nmrsu
3 Site	
4 Instrument	spect
5 Author	
6 Solvent	DMSO
7 Temperature	300.5
8 Pulse Sequence	zgpg30
9 Experiment	1D
10 Probe	Z108618_0043 (PA BBO 400S1 BBF-H-D-05 Z PLUS)
11 Number of Scans	3064
12 Receiver Gain	2050.0
13 Relaxation Delay	2.0000
14 Pulse Width	10.0000
15 Presaturation Frequency	
16 Acquisition Time	1.3631
17 Acquisition Date	2021-05-02T09:59:21
18 Modification Date	2021-05-02T09:59:21
19 Class	
20 Spectrometer Frequency	100.62
21 Spectral Width	24038.5
22 Lowest Frequency	-2009.3
23 Nucleus	13C
24 Acquired Size	32768
25 Spectral Size	65536
26 Digital Resolution	0.37

^{13}C NMR (100 MHz, DMSO) δ 164.9, 164.4, 149.4, 143.0, 134.9, 129.7, 127.1, 123.3, 119.6, 106.9, 60.9, 42.0, 13.9.

Ethyl (*E*)-2-(2-(methylsulfonyl)-5-nitro-3-oxoisoindolin-1-ylidene)acetate (4g)

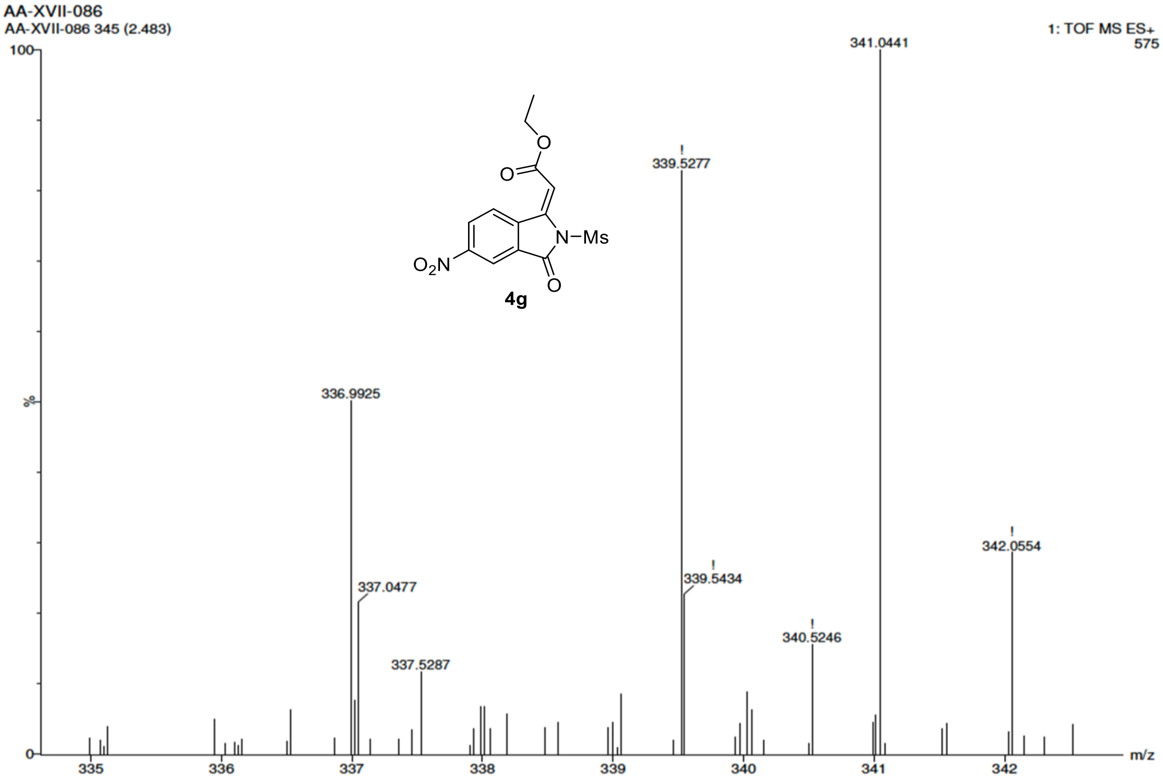

HRMS (ESI) *m/z* calcd for $C_{13}H_{12}N_2O_7S$ $[M + H]^+$ 341.0438; found 341.0441.

Ethyl (*E*)-2-(6-chloro-2-(methylsulfonyl)-3-oxoisoindolin-1-ylidene)acetate (4h)

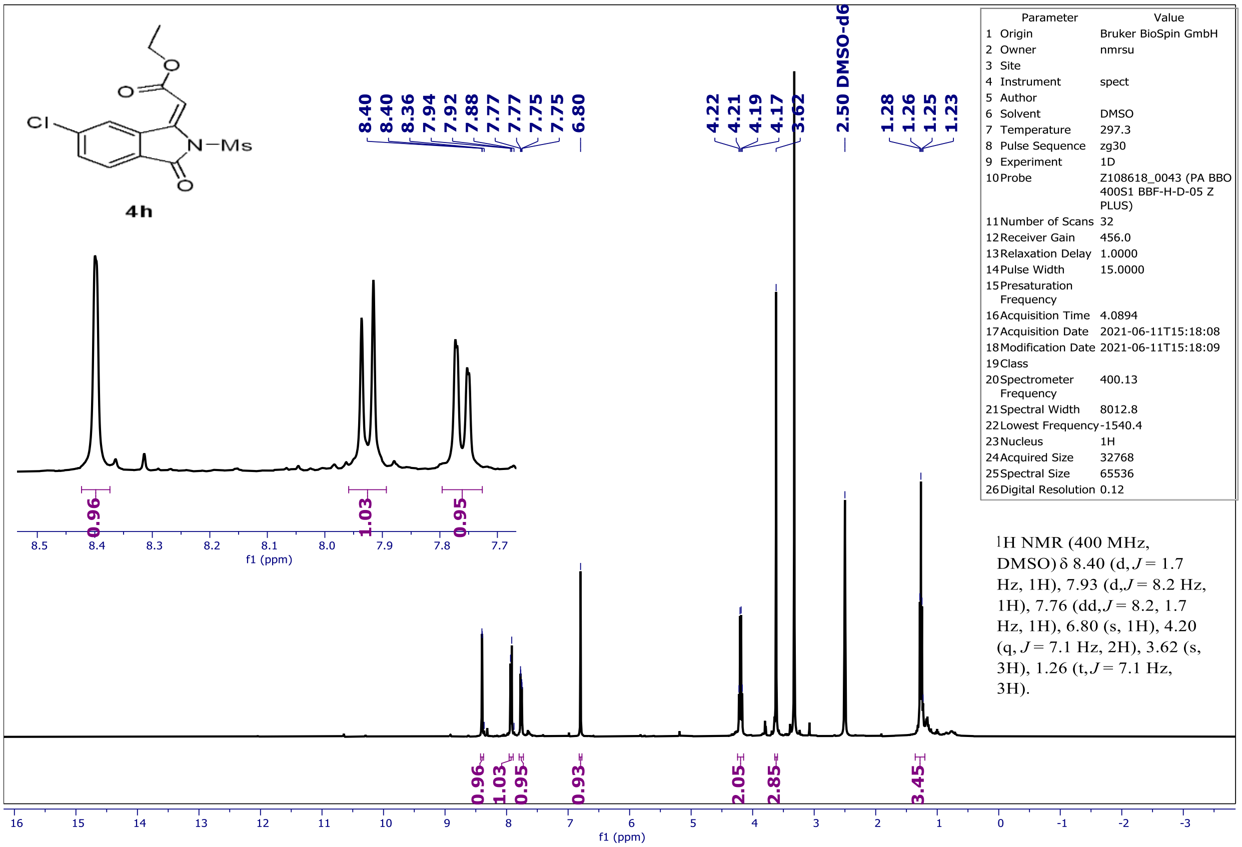

^{1}H NMR (400 MHz, DMSO) δ 8.40 (d, *J* = 1.7 Hz, 1H), 7.93 (d, *J* = 8.2 Hz, 1H), 7.76 (dd, *J* = 8.2, 1.7 Hz, 1H), 6.80 (s, 1H), 4.20 (q, *J* = 7.1 Hz, 2H), 3.62 (s, 3H), 1.26 (t, *J* = 7.1 Hz, 3H).

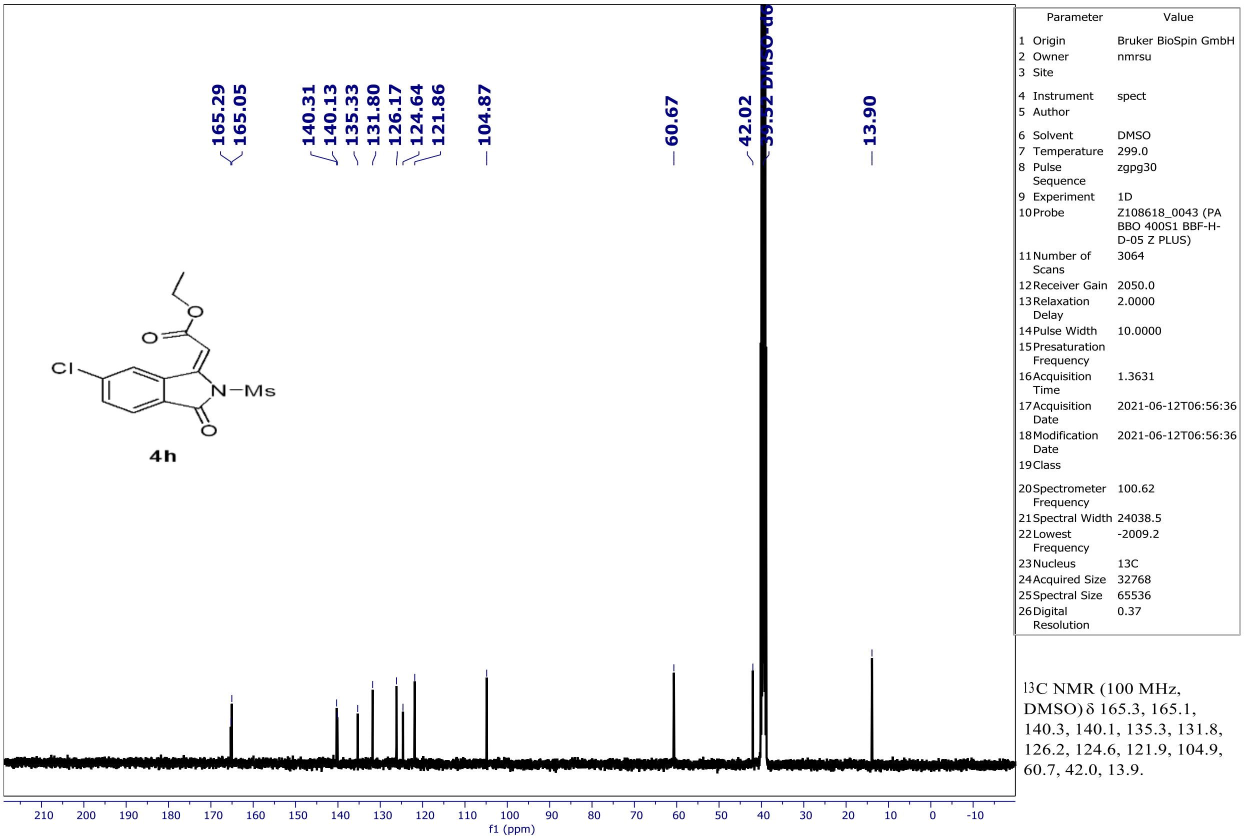

^{13}C NMR (100 MHz, DMSO) δ 165.3, 165.1, 140.3, 140.1, 135.3, 131.8, 126.2, 124.6, 121.9, 104.9, 60.7, 42.0, 13.9.

Ethyl (*E*)-2-(6-chloro-2-(methylsulfonyl)-3-oxoisoindolin-1-ylidene)acetate (4h)

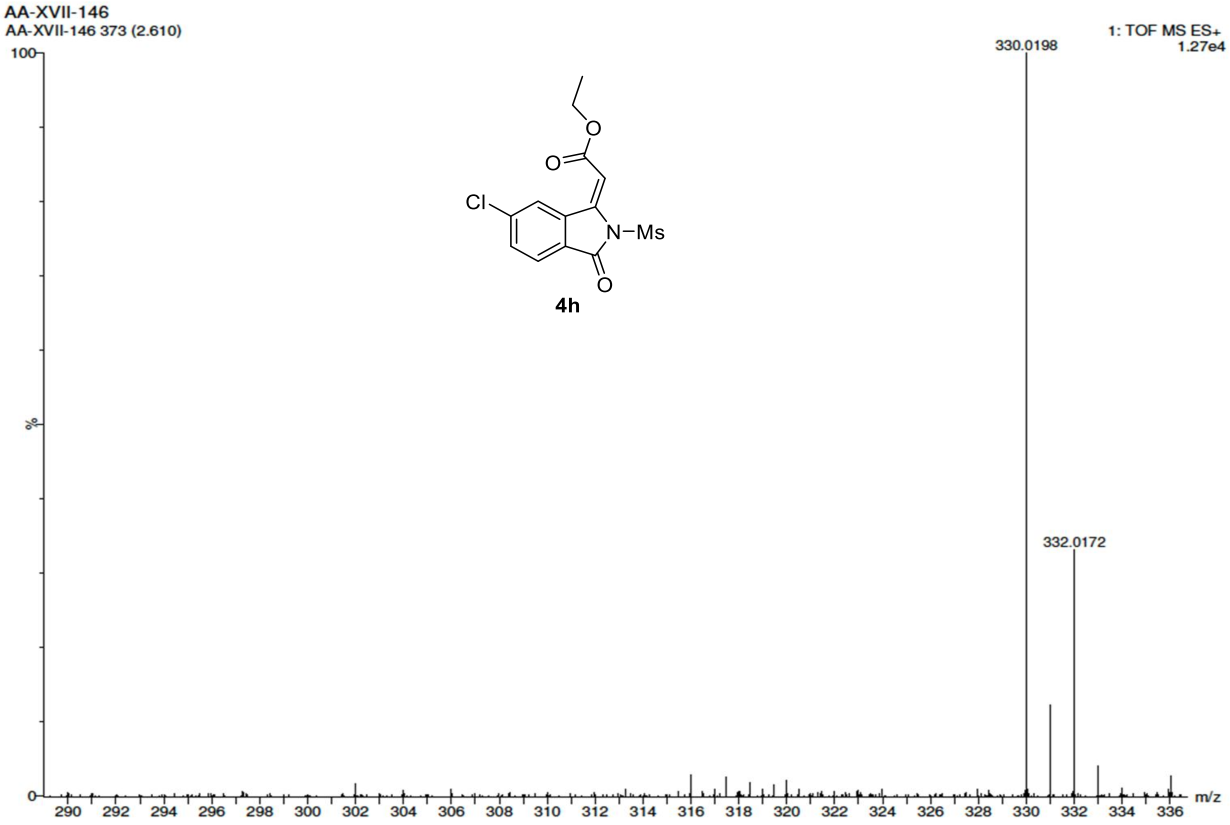

HRMS (ESI) *m/z* calcd for $C_{13}H_{12}ClNO_5S$ $[M + H]^+$ 330.0197; found 330.0198.

Ethyl (*E*)-2-(2-(methylsulfonyl)-6-nitro-3-oxoisoindolin-1-ylidene)acetate (4i)

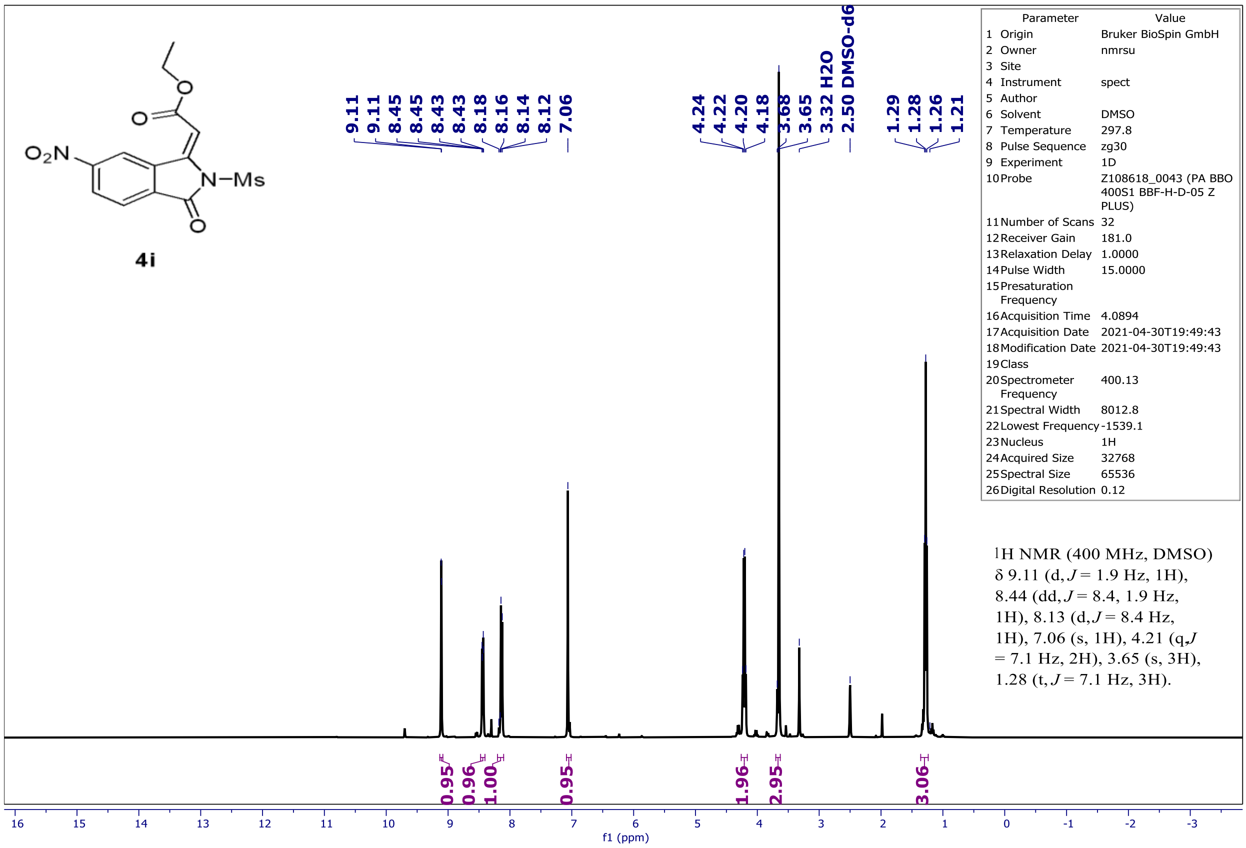

Parameter		Value
1	Origin	Bruker BioSpin GmbH
2	Owner	nmrsu
3	Site	
4	Instrument	spect
5	Author	
6	Solvent	DMSO
7	Temperature	297.8
8	Pulse Sequence	zg30
9	Experiment	1D
10	Probe	Z108618_0043 (PA BBO 400S1 BBF-H-D-05 Z PLUS)
11	Number of Scans	32
12	Receiver Gain	181.0
13	Relaxation Delay	1.0000
14	Pulse Width	15.0000
15	Presaturation Frequency	
16	Acquisition Time	4.0894
17	Acquisition Date	2021-04-30T19:49:43
18	Modification Date	2021-04-30T19:49:43
19	Class	
20	Spectrometer Frequency	400.13
21	Spectral Width	8012.8
22	Lowest Frequency	-1539.1
23	Nucleus	1H
24	Acquired Size	32768
25	Spectral Size	65536
26	Digital Resolution	0.12

^{1}H NMR (400 MHz, DMSO) δ 9.11 (d, *J* = 1.9 Hz, 1H), 8.44 (dd, *J* = 8.4, 1.9 Hz, 1H), 8.13 (d, *J* = 8.4 Hz, 1H), 7.06 (s, 1H), 4.21 (q, *J* = 7.1 Hz, 2H), 3.65 (s, 3H), 1.28 (t, *J* = 7.1 Hz, 3H).

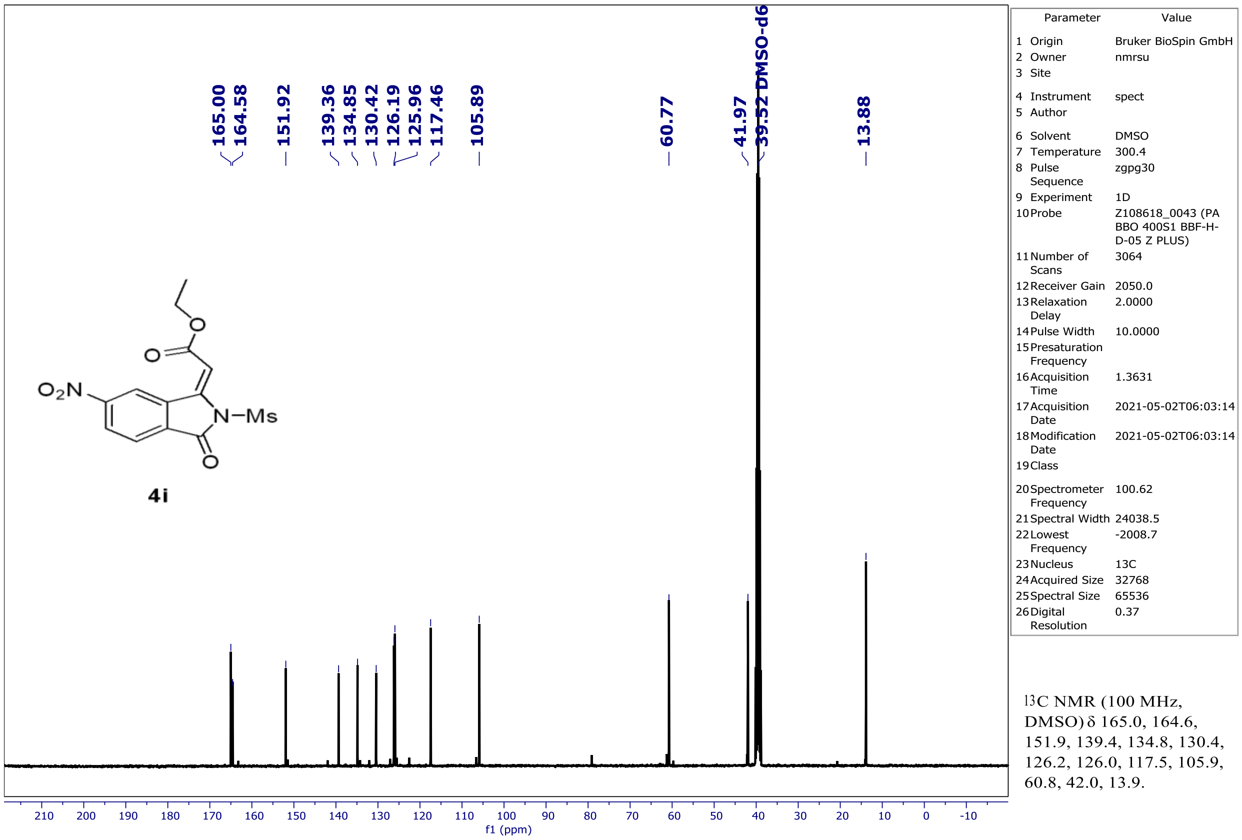

Parameter		Value
1	Origin	Bruker BioSpin GmbH
2	Owner	nmrsu
3	Site	
4	Instrument	spect
5	Author	
6	Solvent	DMSO
7	Temperature	300.4
8	Pulse Sequence	zgpg30
9	Experiment	1D
10	Probe	Z108618_0043 (PA BBO 400S1 BBF-H-D-05 Z PLUS)
11	Number of Scans	3064
12	Receiver Gain	2050.0
13	Relaxation Delay	2.0000
14	Pulse Width	10.0000
15	Presaturation Frequency	
16	Acquisition Time	1.3631
17	Acquisition Date	2021-05-02T06:03:14
18	Modification Date	2021-05-02T06:03:14
19	Class	
20	Spectrometer Frequency	100.62
21	Spectral Width	24038.5
22	Lowest Frequency	-2008.7
23	Nucleus	13C
24	Acquired Size	32768
25	Spectral Size	65536
26	Digital Resolution	0.37

^{13}C NMR (100 MHz, DMSO) δ 165.0, 164.6, 151.9, 139.4, 134.8, 130.4, 126.2, 126.0, 117.5, 105.9, 60.8, 42.0, 13.9.

Ethyl (*E*)-2-(2-(methylsulfonyl)-6-nitro-3-oxoisoindolin-1-ylidene)acetate (4i)

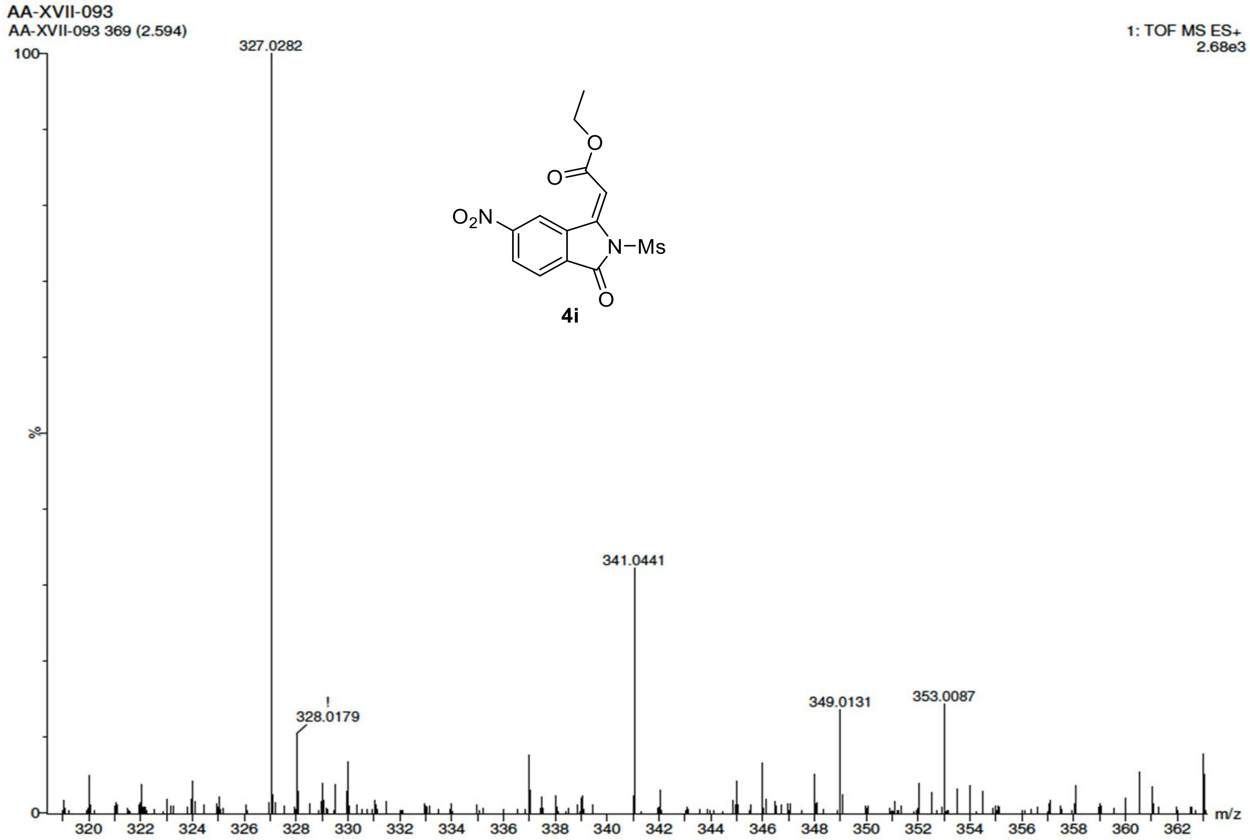

HRMS (ESI) *m/z* calcd for $C_{13}H_{12}N_2O_7S$ $[M + H]^+$ 341.0438; found 341.0441.

(*E*)-3-Benzylidene-2-(methylsulfonyl)isoindolin-1-one (4j)

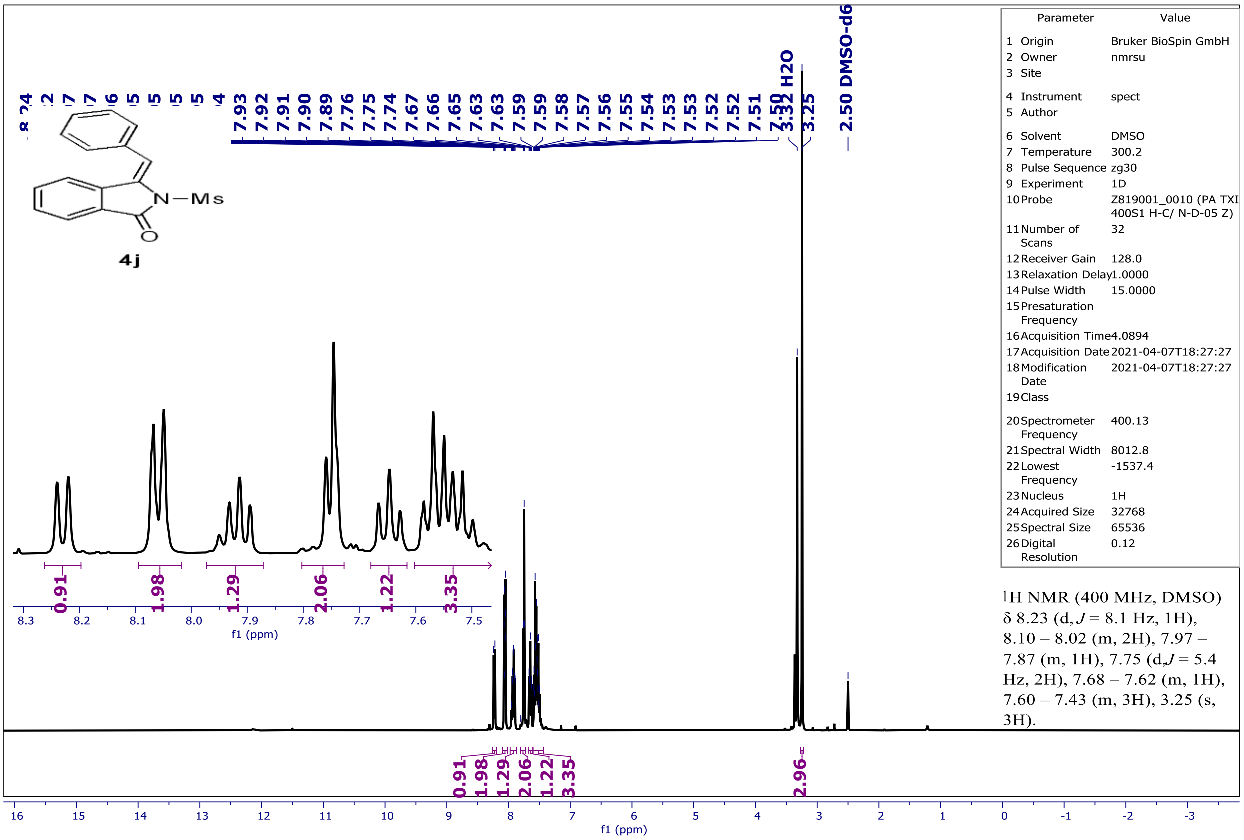

^{1}H NMR (400 MHz, DMSO) δ 8.23 (d, *J* = 8.1 Hz, 1H), 8.10 – 8.02 (m, 2H), 7.97 – 7.87 (m, 1H), 7.75 (d, *J* = 5.4 Hz, 2H), 7.68 – 7.62 (m, 1H), 7.60 – 7.43 (m, 3H), 3.25 (s, 3H).

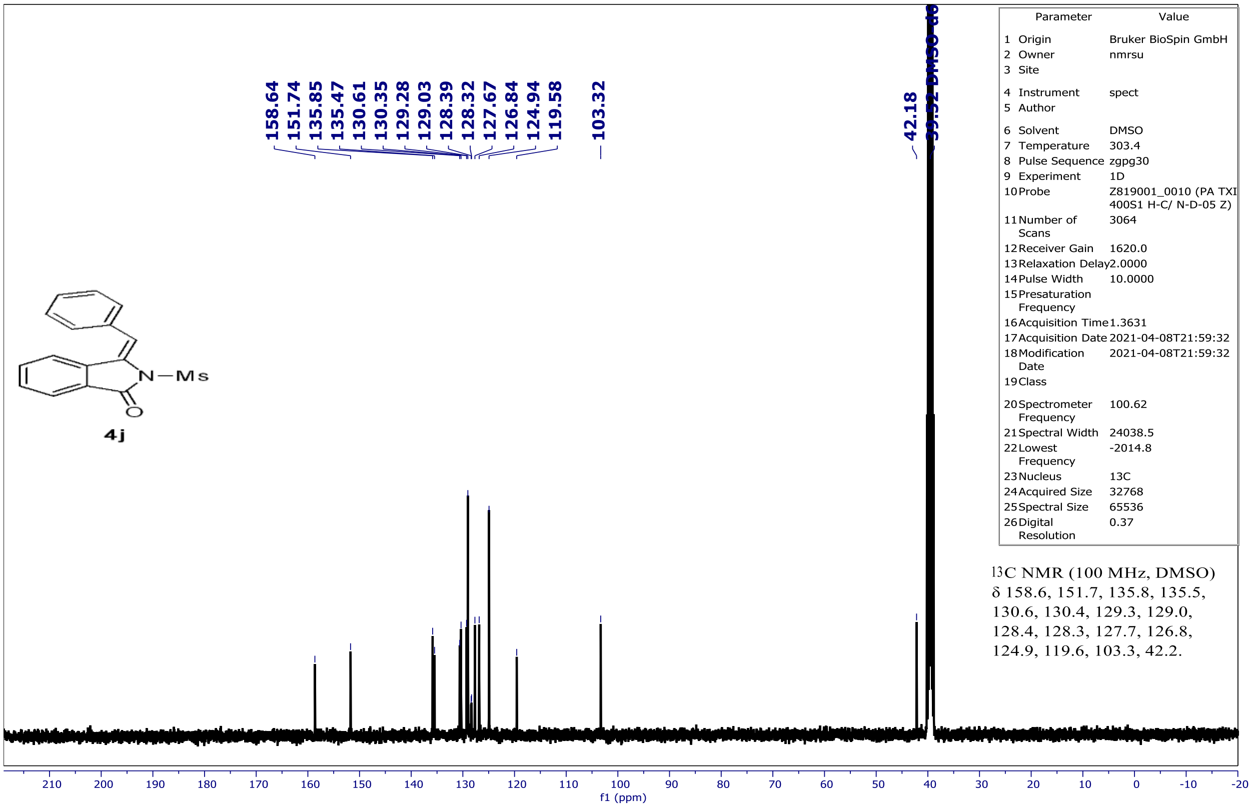

^{13}C NMR (100 MHz, DMSO) δ 158.6, 151.7, 135.8, 135.5, 130.6, 130.4, 129.3, 129.0, 128.4, 128.3, 127.7, 126.8, 124.9, 119.6, 103.3, 42.2.

(*E*)-3-Benzylidene-2-(methylsulfonyl)isoindolin-1-one (4j)

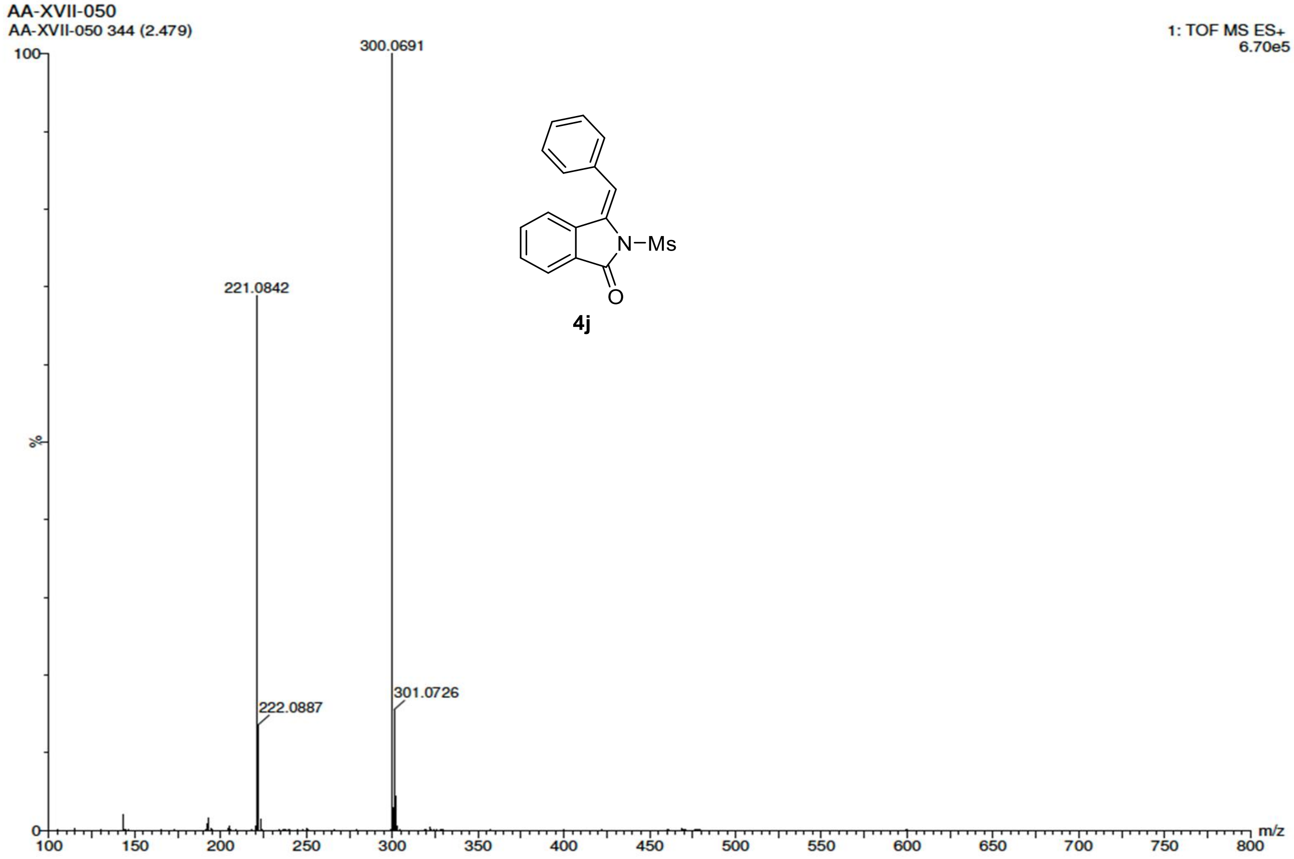

HRMS (ESI) *m/z* calcd for $C_{16}H_{13}NO_3S$ $[M + H]^+$ 300.0689; found 300.0691.

(*E*)-3-Benzylidene-6-fluoro-2-(methylsulfonyl)isoindolin-1-one (4k)

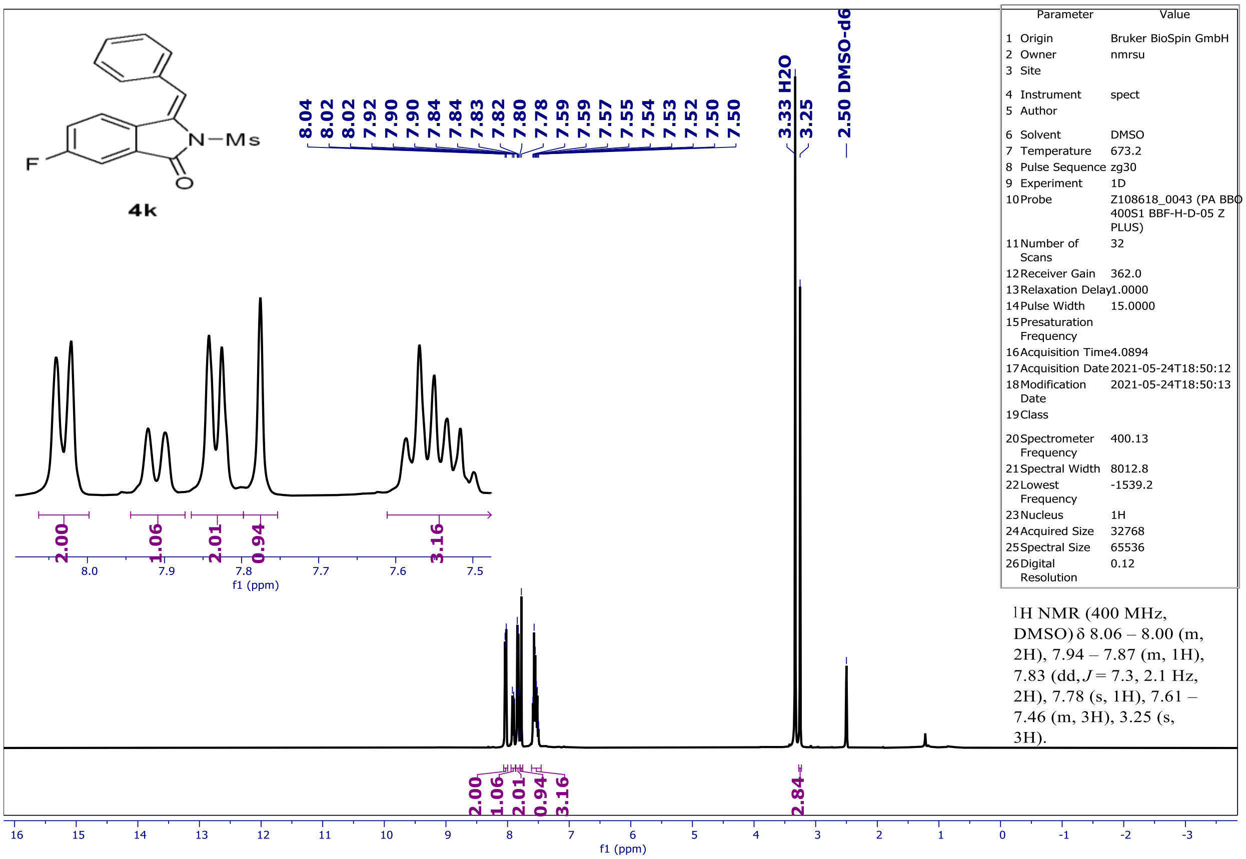

^{1}H NMR (400 MHz, DMSO) δ 8.06 – 8.00 (m, 2H), 7.94 – 7.87 (m, 1H), 7.83 (dd, *J* = 7.3, 2.1 Hz, 2H), 7.78 (s, 1H), 7.61 – 7.46 (m, 3H), 3.25 (s, 3H).

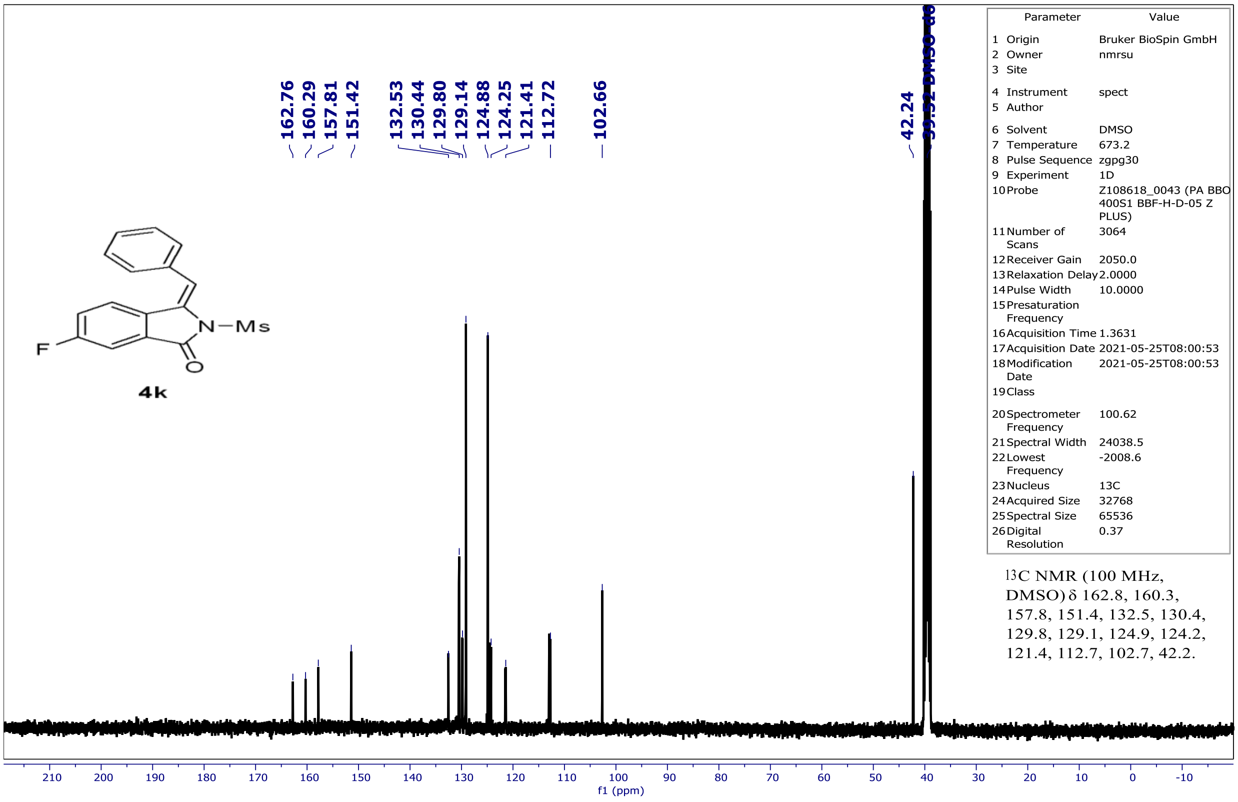

^{13}C NMR (100 MHz, DMSO) δ 162.8, 160.3, 157.8, 151.4, 132.5, 130.4, 129.8, 129.1, 124.9, 124.2, 121.4, 112.7, 102.7, 42.2.

(*E*)-3-Benzylidene-6-fluoro-2-(methylsulfonyl)isoindolin-1-one (4k)

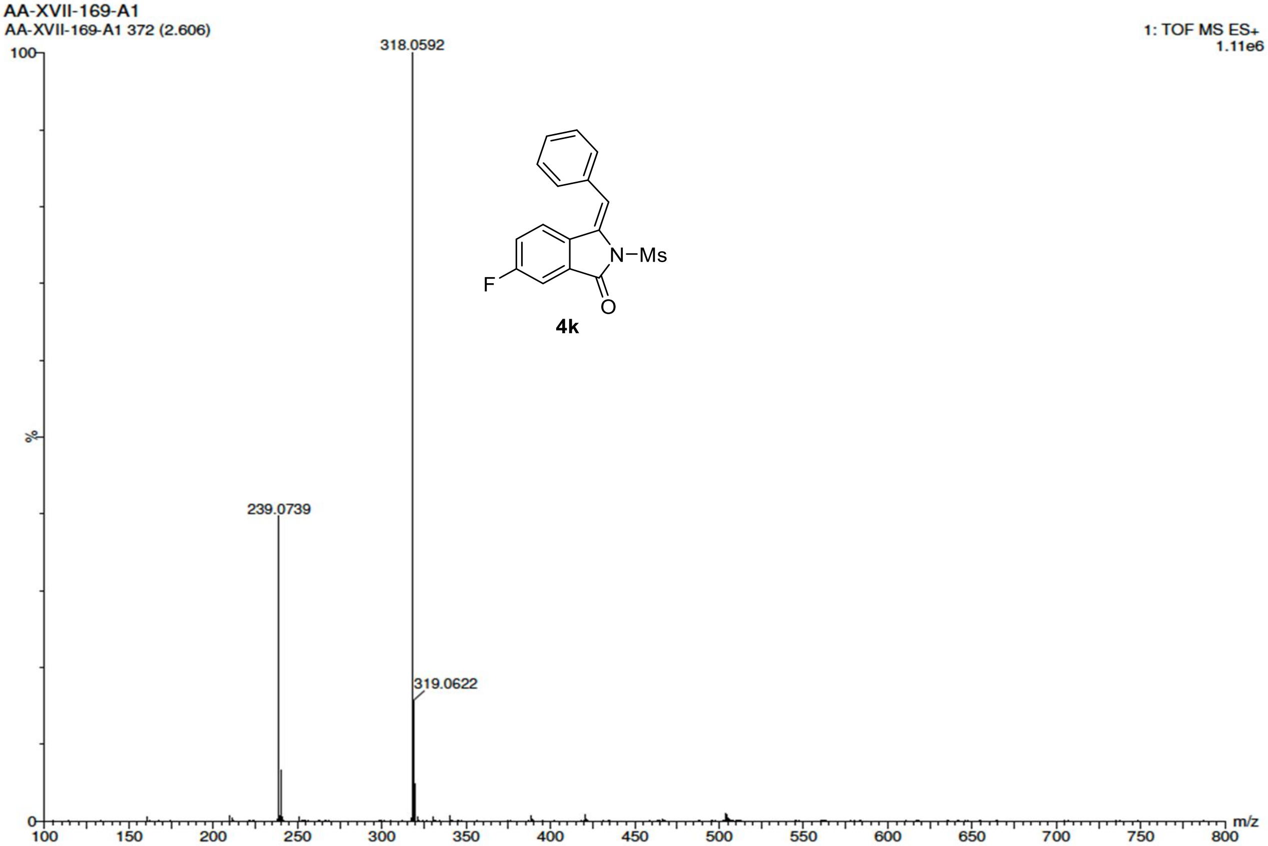

HRMS (ESI) *m/z* calcd for $C_{16}H_{12}FNO_3S$ $[M + H]^+$ 318.0595; found 318.0592.

(*E*)-3-Benzylidene-6-chloro-2-(methylsulfonyl)isoindolin-1-one (4l)

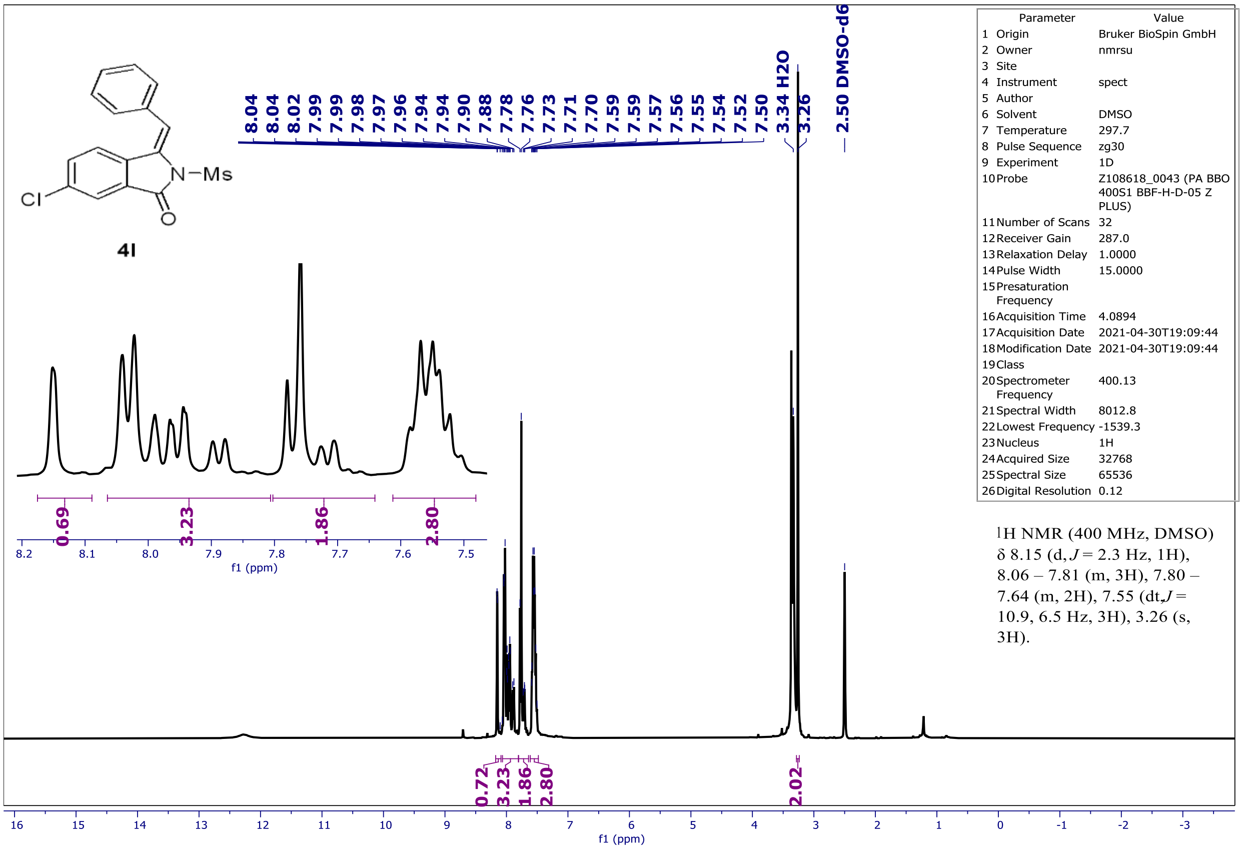

1H NMR (400 MHz, DMSO) δ 8.15 (d, *J* = 2.3 Hz, 1H), 8.06 – 7.81 (m, 3H), 7.80 – 7.64 (m, 2H), 7.55 (dt, *J* = 10.9, 6.5 Hz, 3H), 3.26 (s, 3H).

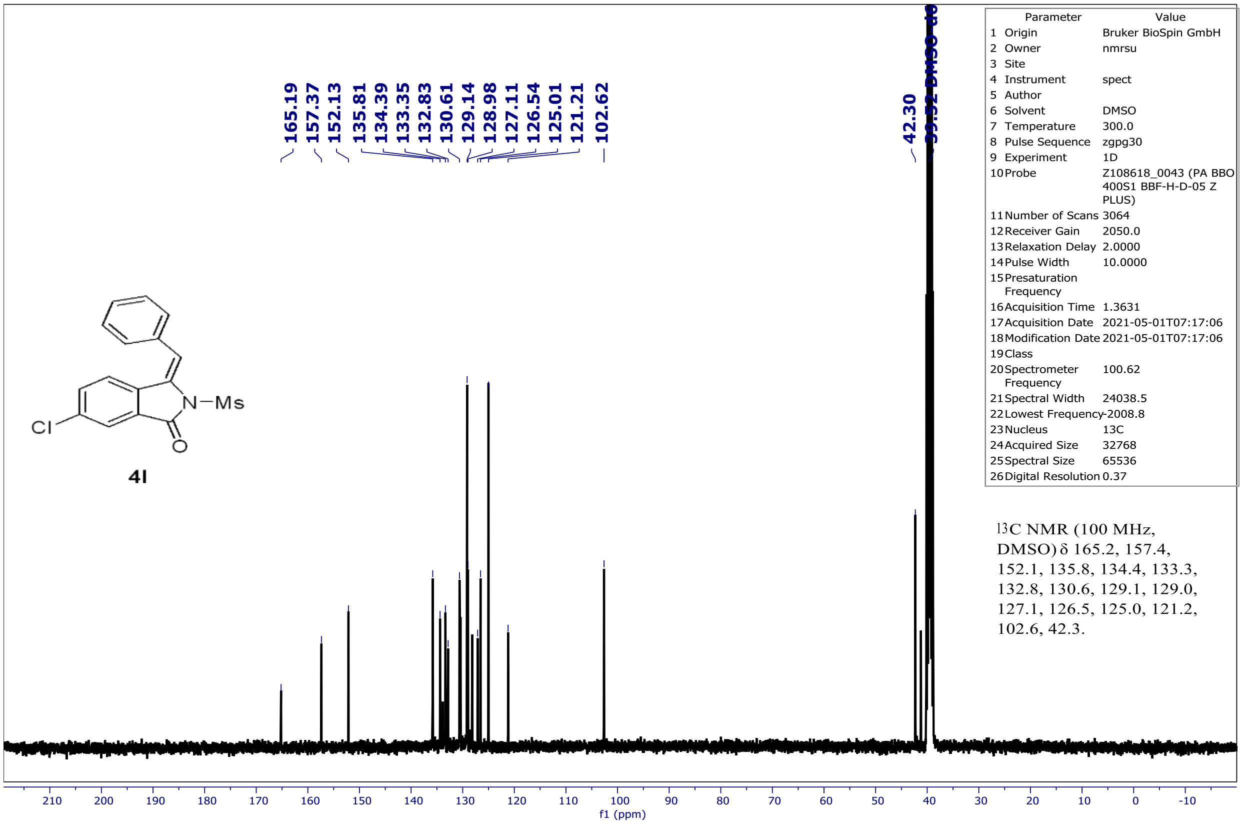

13C NMR (100 MHz, DMSO) δ 165.2, 157.4, 152.1, 135.8, 134.4, 133.3, 132.8, 130.6, 129.1, 129.0, 127.1, 126.5, 125.0, 121.2, 102.6, 42.3.

(*E*)-3-Benzylidene-6-chloro-2-(methylsulfonyl)isoindolin-1-one (4l)

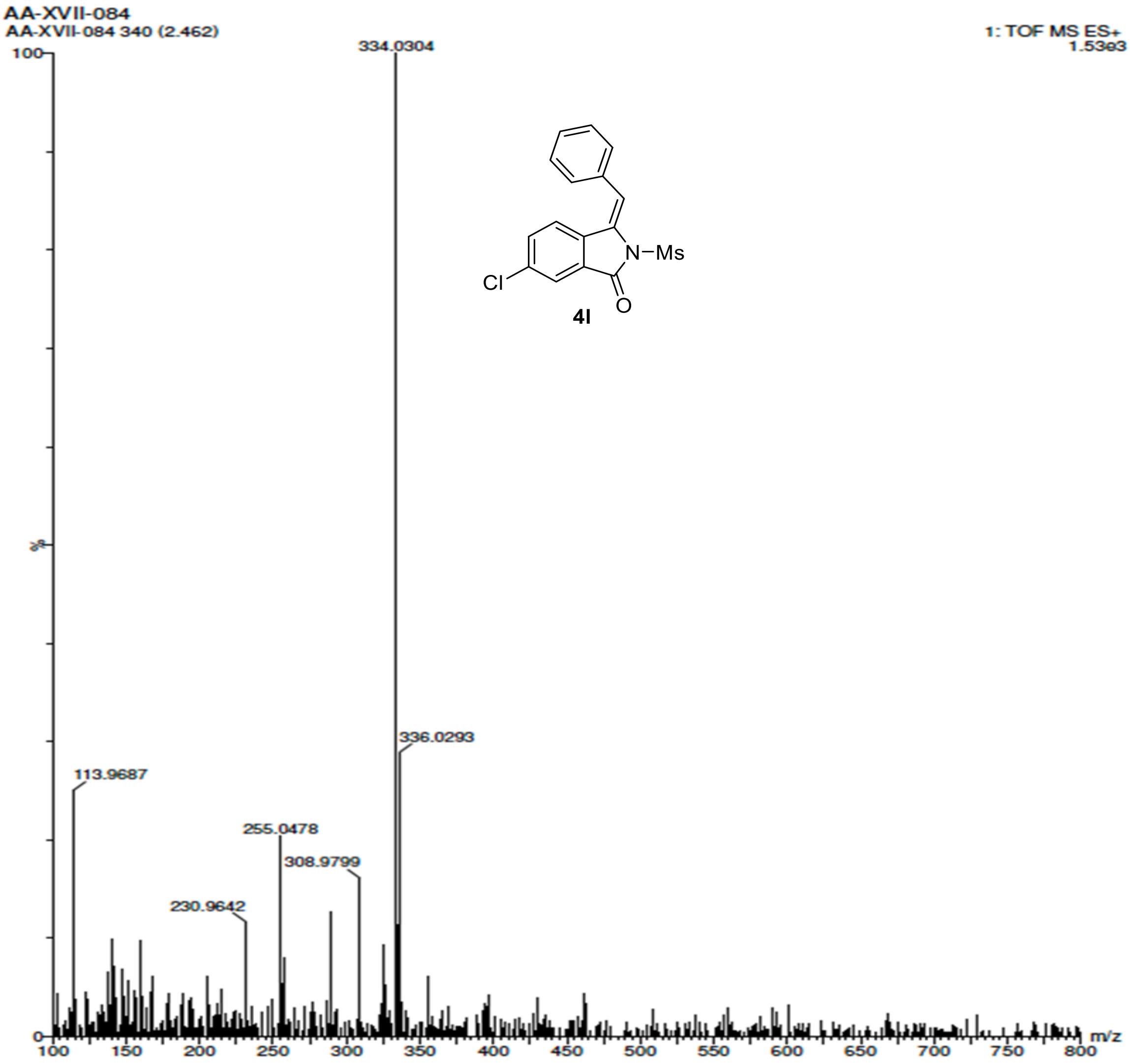

HRMS (ESI) *m/z* calcd for $C_{16}H_{12}ClNO_3S$ $[M + H]^+$ 334.0299; found 334.0304.

(*E*)-3-Benzylidene-6-bromo-2-(methylsulfonyl)isoindolin-1-one (4m)

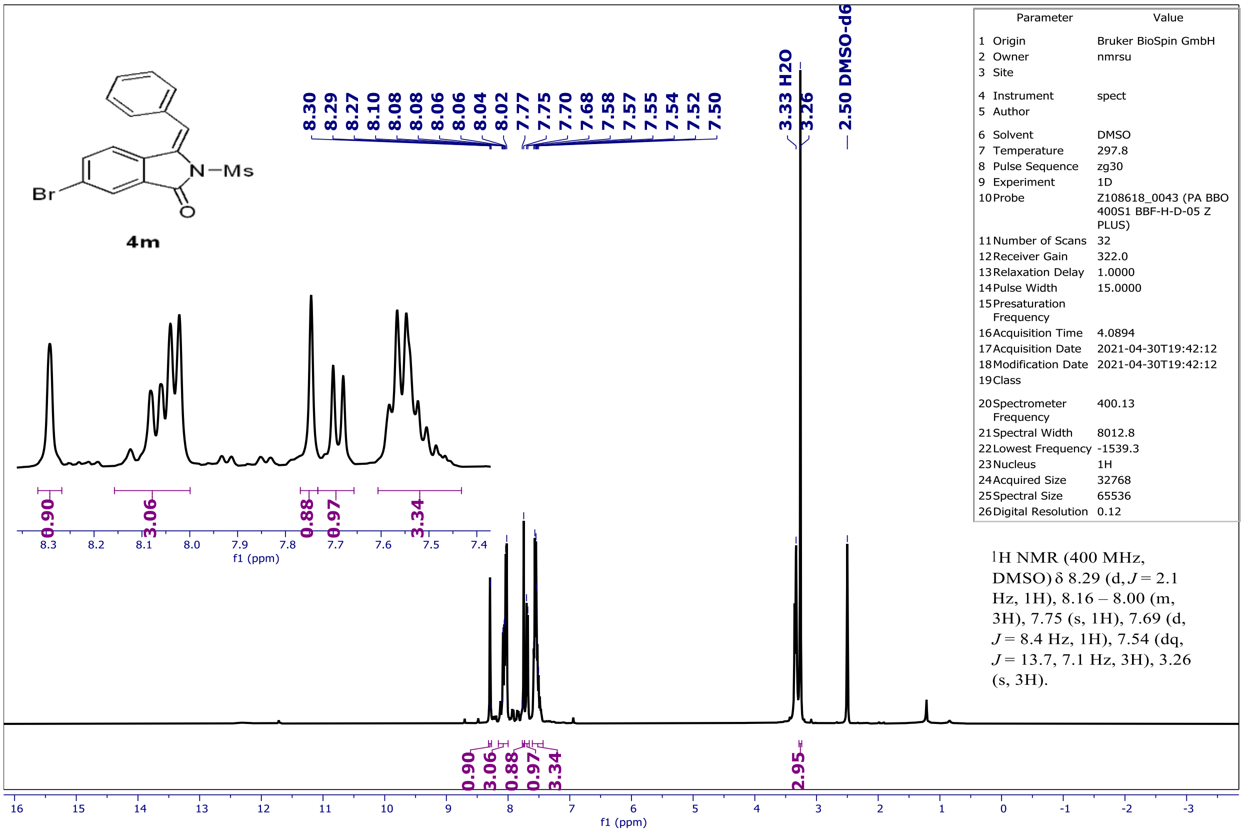

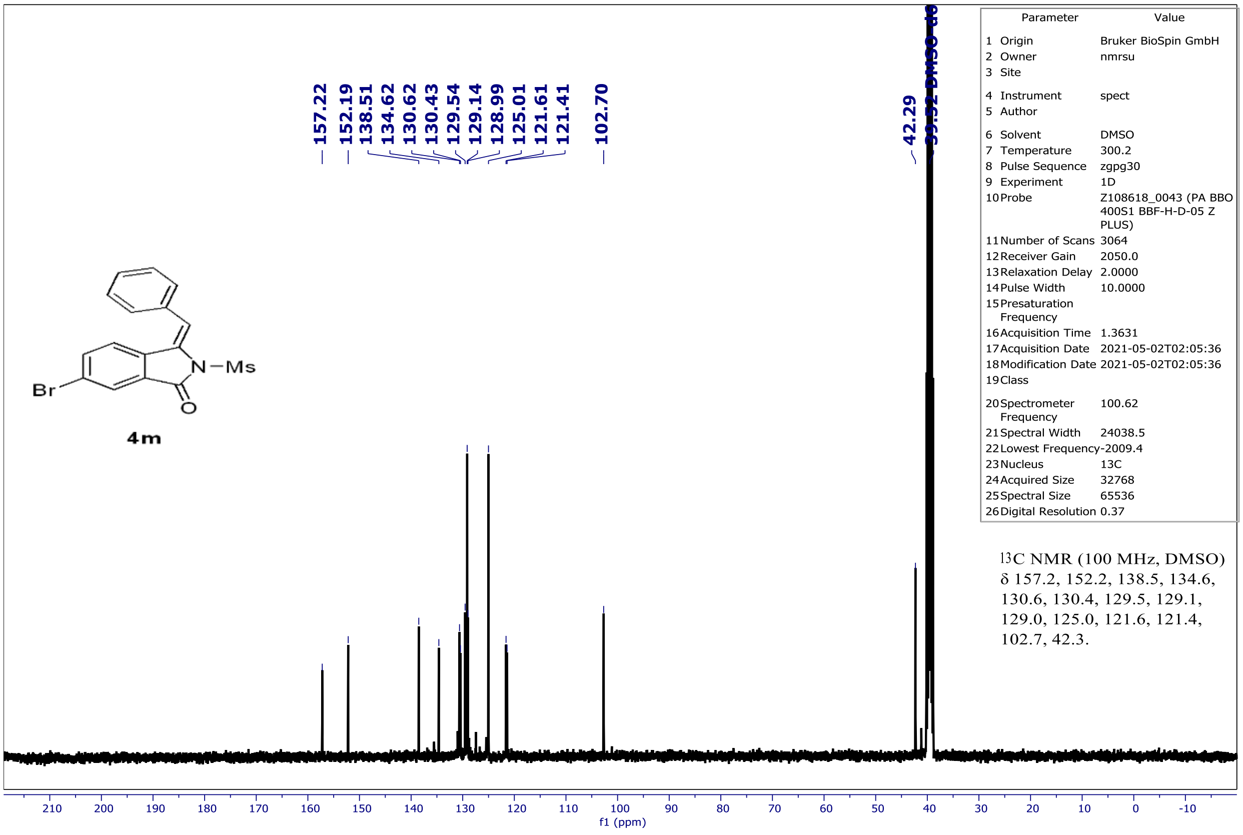

(*E*)-3-Benzylidene-6-bromo-2-(methylsulfonyl)isoindolin-1-one (4m)

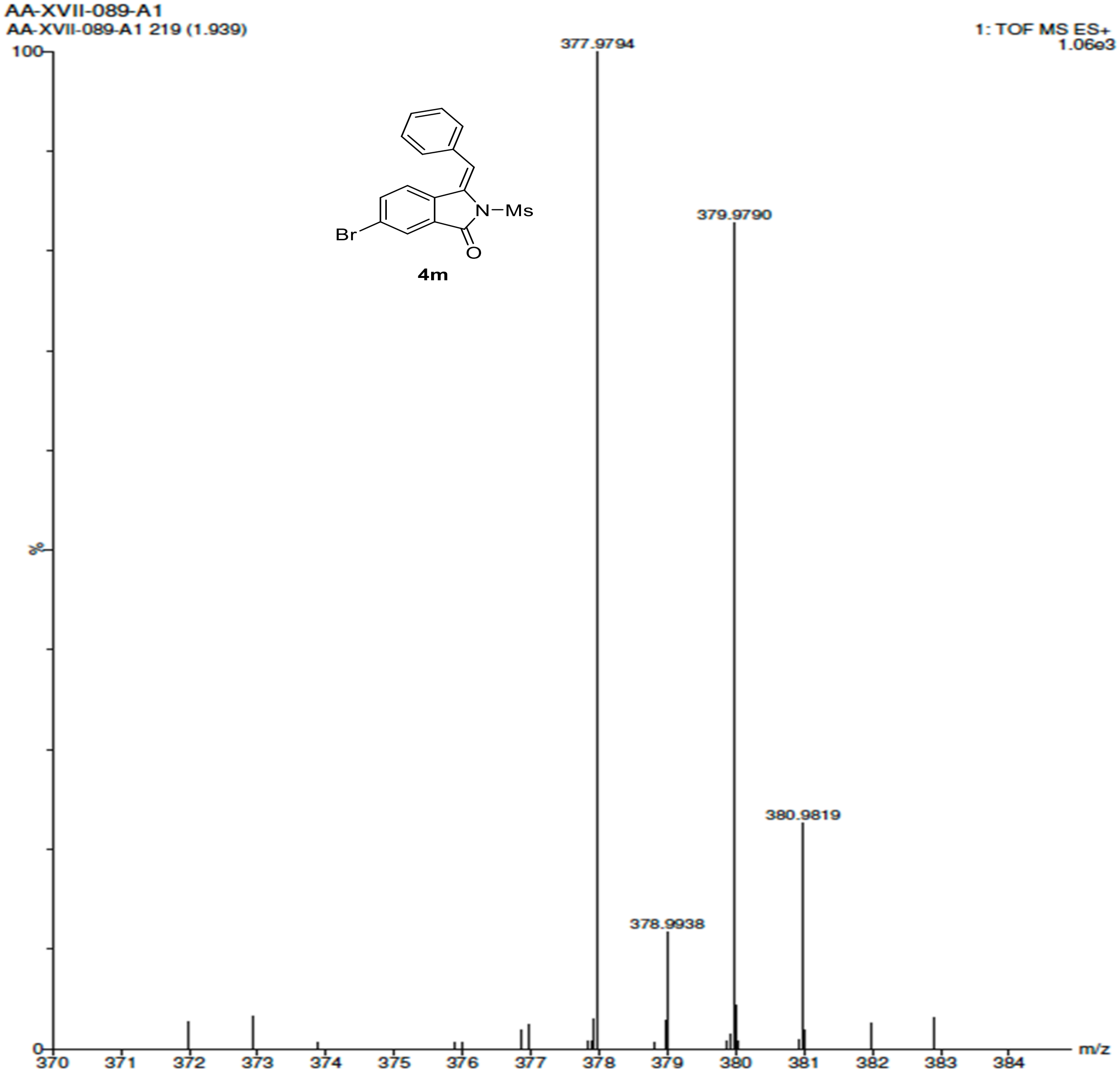

HRMS (ESI) *m/z* calcd for $C_{16}H_{12}BrNO_3S$ $[M + H]^+$ 377.9794; found 377.9794.

(*E*)-3-Benzylidene-6-methyl-2-(methylsulfonyl)isoindolin-1-one (4n)

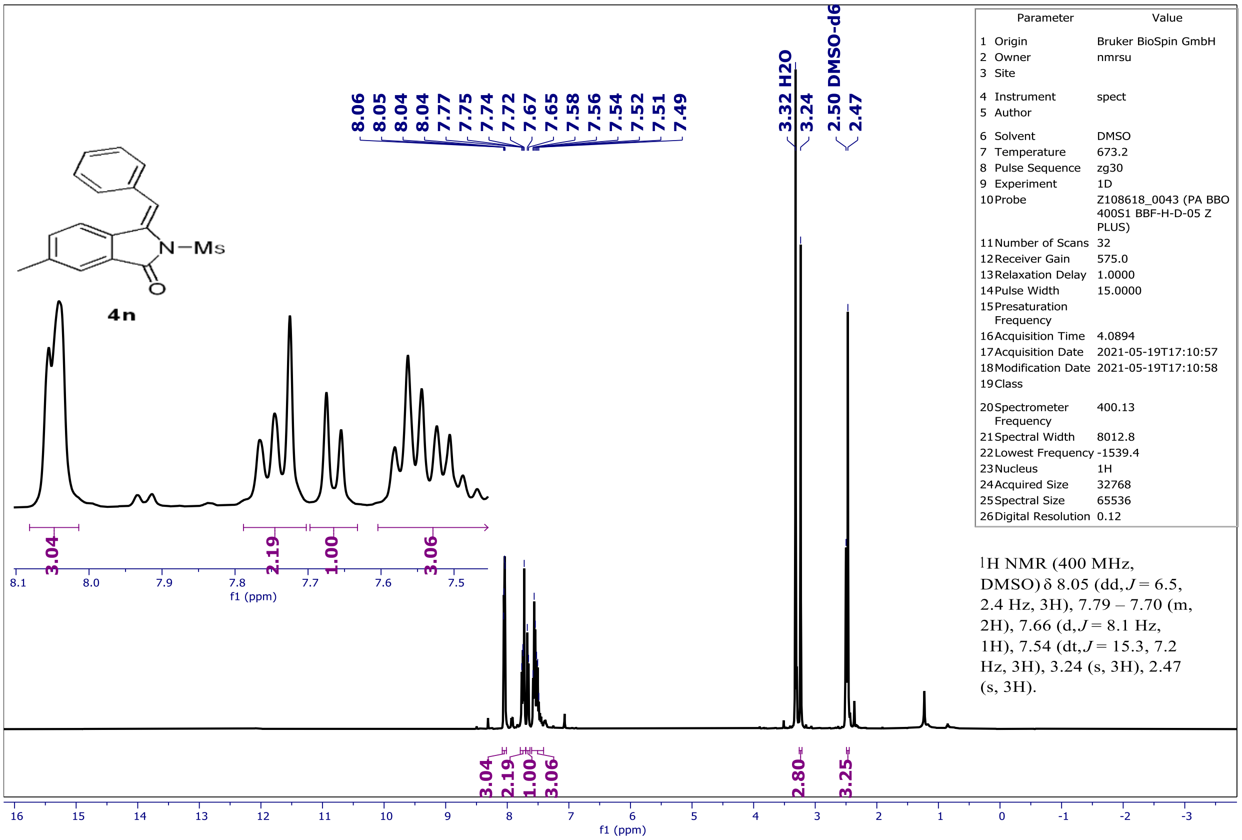

Parameter	Value
1 Origin	Bruker BioSpin GmbH
2 Owner	nmrsu
3 Site	
4 Instrument	spect
5 Author	
6 Solvent	DMSO
7 Temperature	673.2
8 Pulse Sequence	zg30
9 Experiment	1D
10 Probe	Z108618_0043 (PA BBO 400S1 BBF-H-D-05 Z PLUS)
11 Number of Scans	32
12 Receiver Gain	575.0
13 Relaxation Delay	1.0000
14 Pulse Width	15.0000
15 Presaturation Frequency	
16 Acquisition Time	4.0894
17 Acquisition Date	2021-05-19T17:10:57
18 Modification Date	2021-05-19T17:10:58
19 Class	
20 Spectrometer Frequency	400.13
21 Spectral Width	8012.8
22 Lowest Frequency	-1539.4
23 Nucleus	1H
24 Acquired Size	32768
25 Spectral Size	65536
26 Digital Resolution	0.12

^{1}H NMR (400 MHz, DMSO) δ 8.05 (dd, *J* = 6.5, 2.4 Hz, 3H), 7.79 – 7.70 (m, 2H), 7.66 (d, *J* = 8.1 Hz, 1H), 7.54 (dt, *J* = 15.3, 7.2 Hz, 3H), 3.24 (s, 3H), 2.47 (s, 3H).

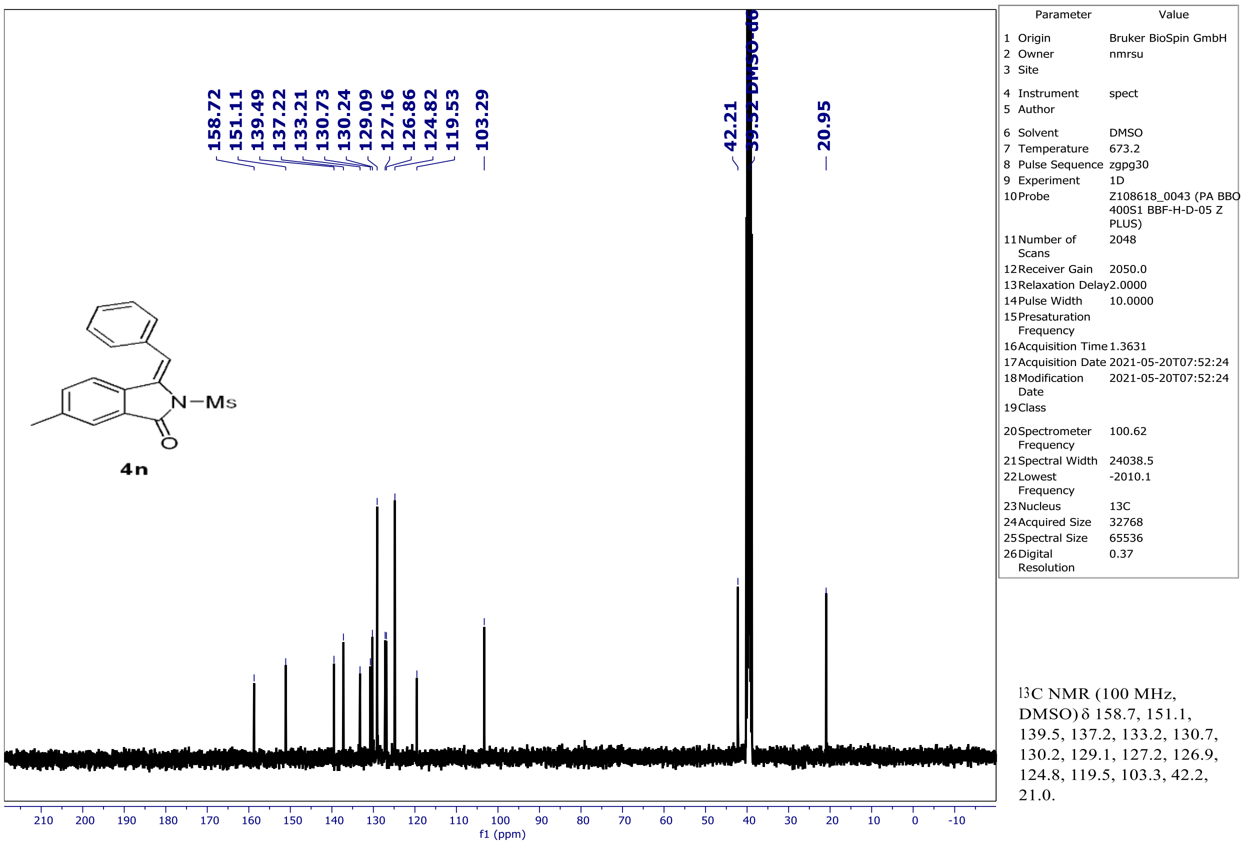

Parameter	Value
1 Origin	Bruker BioSpin GmbH
2 Owner	nmrsu
3 Site	
4 Instrument	spect
5 Author	
6 Solvent	DMSO
7 Temperature	673.2
8 Pulse Sequence	zgpg30
9 Experiment	1D
10 Probe	Z108618_0043 (PA BBO 400S1 BBF-H-D-05 Z PLUS)
11 Number of Scans	2048
12 Receiver Gain	2050.0
13 Relaxation Delay	2.0000
14 Pulse Width	10.0000
15 Presaturation Frequency	
16 Acquisition Time	1.3631
17 Acquisition Date	2021-05-20T07:52:24
18 Modification Date	2021-05-20T07:52:24
19 Class	
20 Spectrometer Frequency	100.62
21 Spectral Width	24038.5
22 Lowest Frequency	-2010.1
23 Nucleus	13C
24 Acquired Size	32768
25 Spectral Size	65536
26 Digital Resolution	0.37

^{13}C NMR (100 MHz, DMSO) δ 158.7, 151.1, 139.5, 137.2, 133.2, 130.7, 130.2, 129.1, 127.2, 126.9, 124.8, 119.5, 103.3, 42.2, 21.0.

(*E*)-3-Benzylidene-6-methyl-2-(methylsulfonyl)isoindolin-1-one (4n)

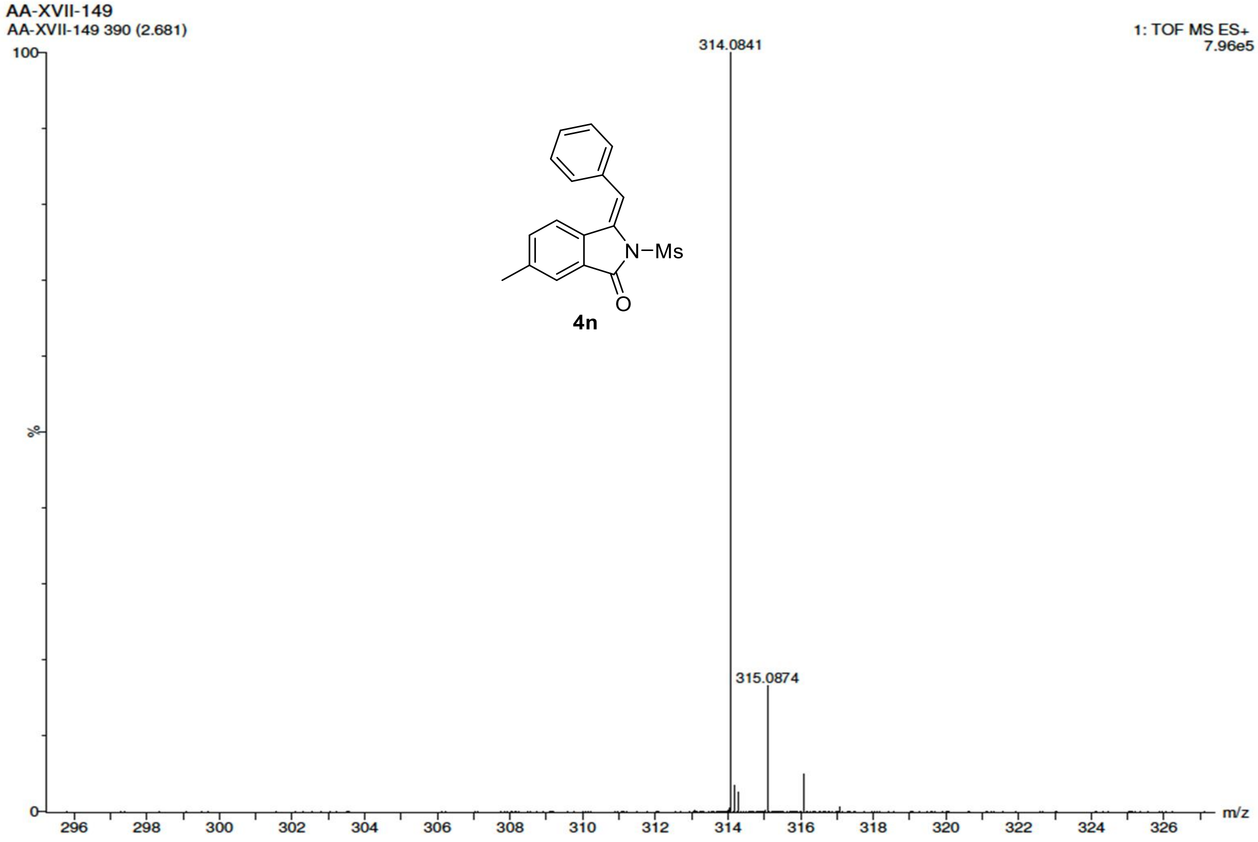

HRMS (ESI) *m/z* calcd for $C_{17}H_{15}NO_3S$ $[M + H]^+$ 314.0845; found 314.0841.

(*E*)-3-Benzylidene-6-methoxy-2-(methylsulfonyl)isoindolin-1-one (4o)

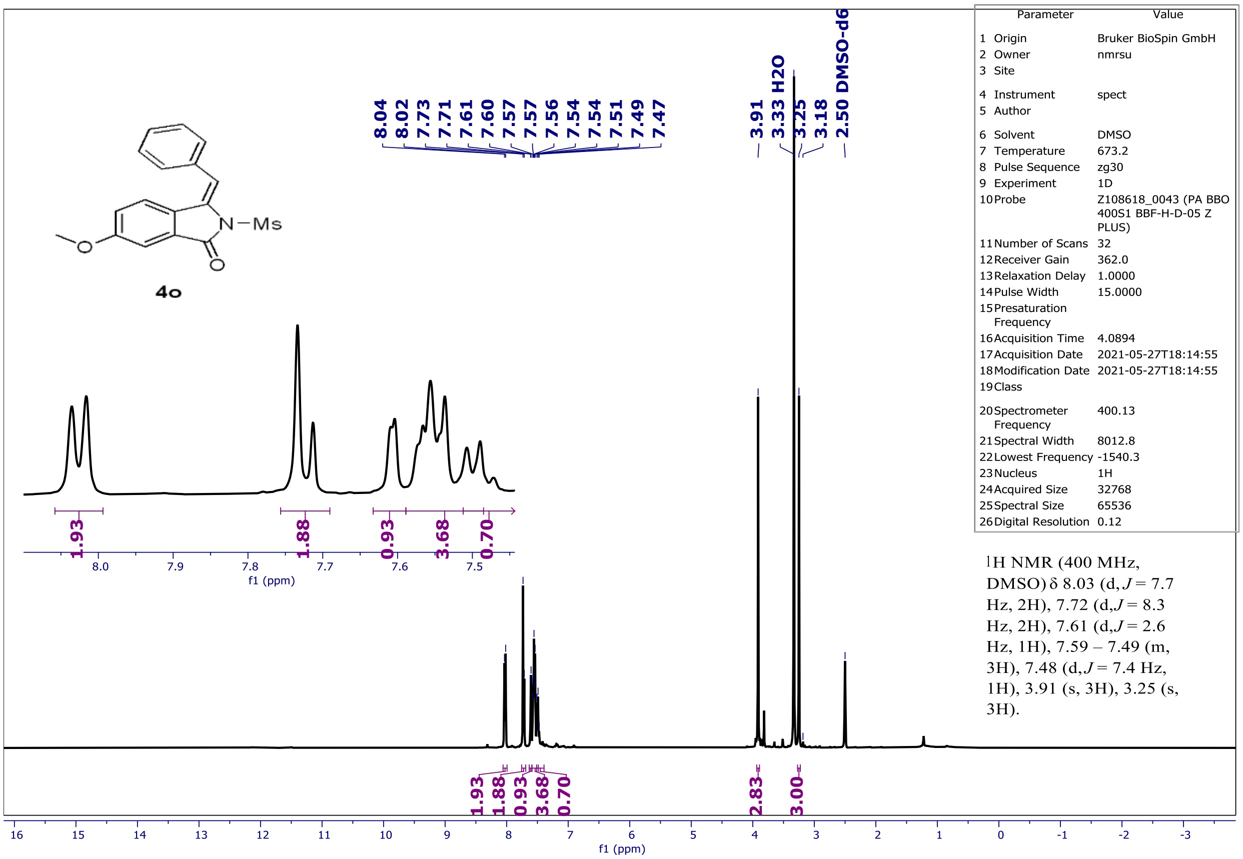

^{1}H NMR (400 MHz, DMSO) δ 8.03 (d, *J* = 7.7 Hz, 2H), 7.72 (d, *J* = 8.3 Hz, 2H), 7.61 (d, *J* = 2.6 Hz, 1H), 7.59 – 7.49 (m, 3H), 7.48 (d, *J* = 7.4 Hz, 1H), 3.91 (s, 3H), 3.25 (s, 3H).

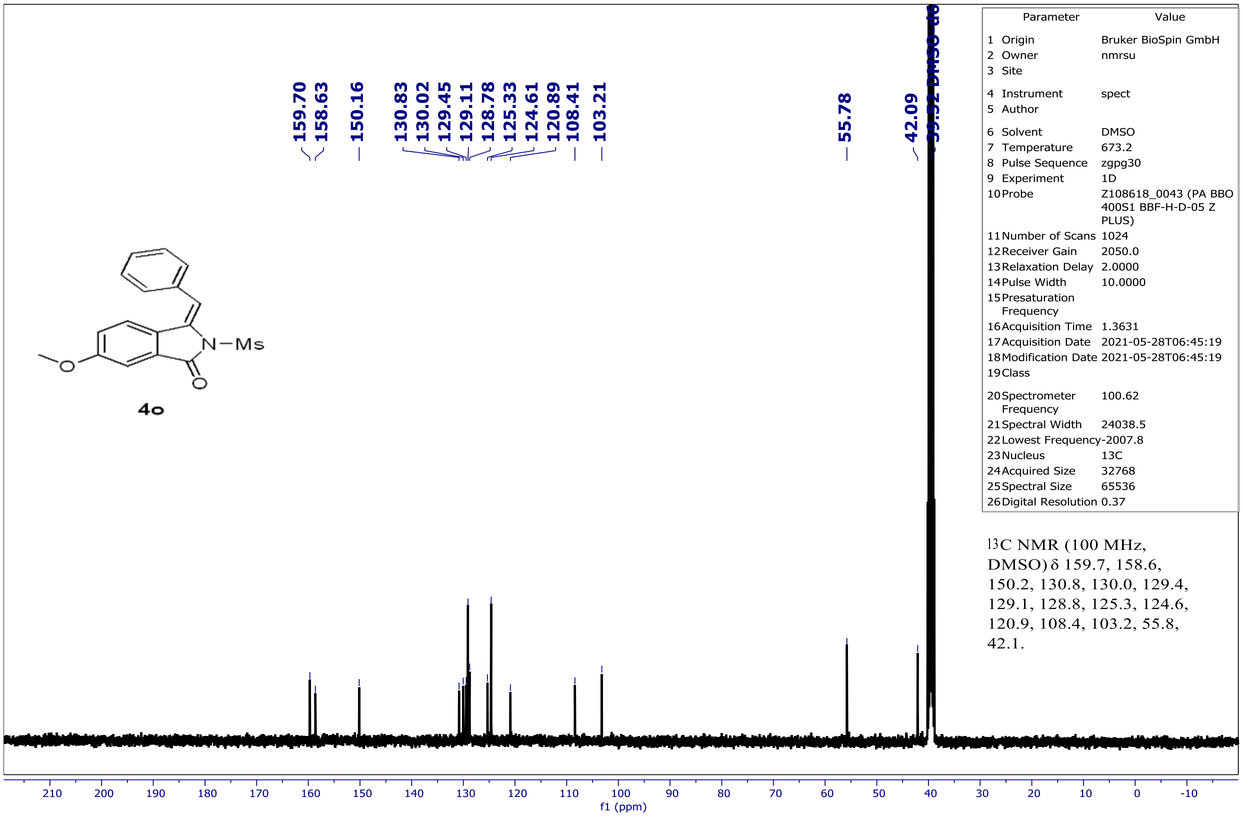

^{13}C NMR (100 MHz, DMSO) δ 159.7, 158.6, 150.2, 130.8, 130.0, 129.4, 129.1, 128.8, 125.3, 124.6, 120.9, 108.4, 103.2, 55.8, 42.1.

(*E*)-3-Benzylidene-6-methoxy-2-(methylsulfonyl)isoindolin-1-one (4o)

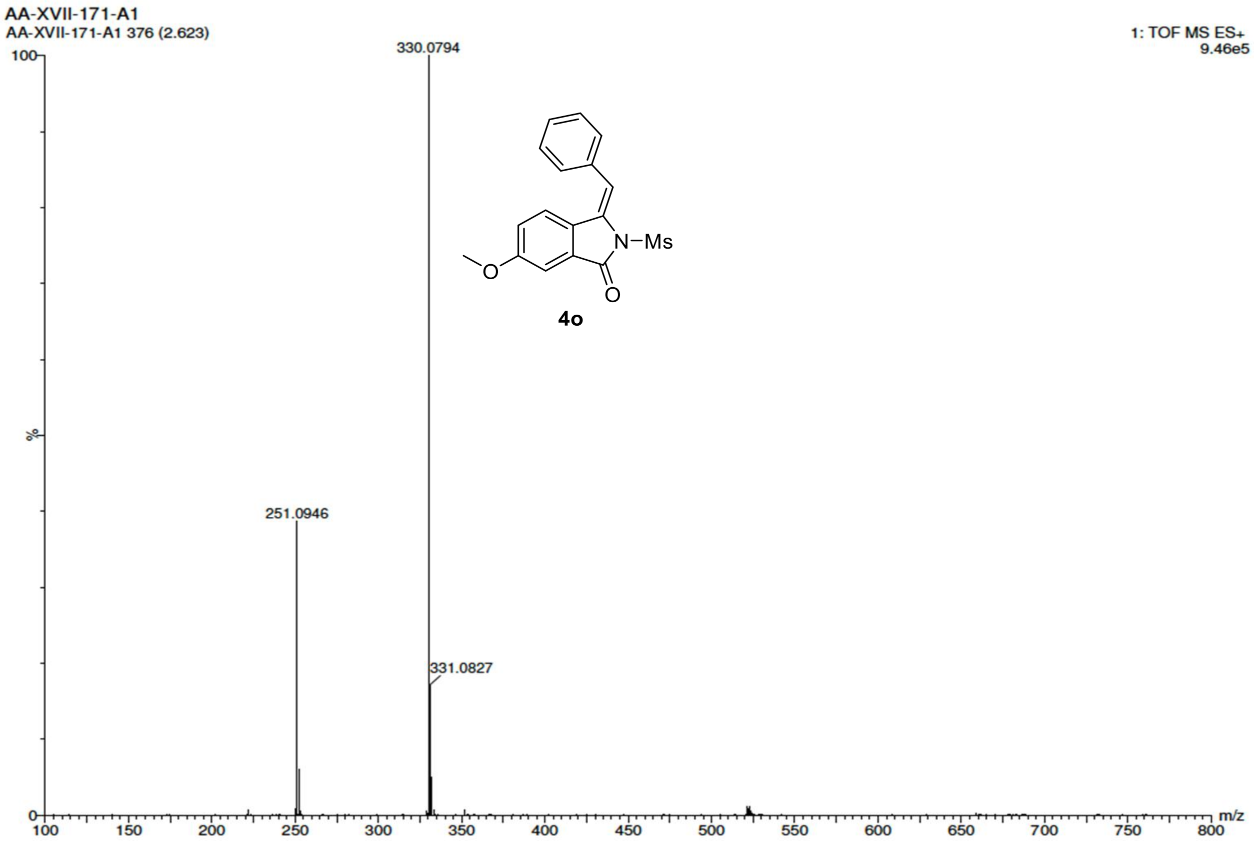

HRMS (ESI) *m/z* calcd for $C_{17}H_{15}NO_3S$ $[M + H]^+$ 330.0795; found 330.0794.

(*E*)-3-Benzylidene-2-(methylsulfonyl)-6-nitroisoindolin-1-one (4p)

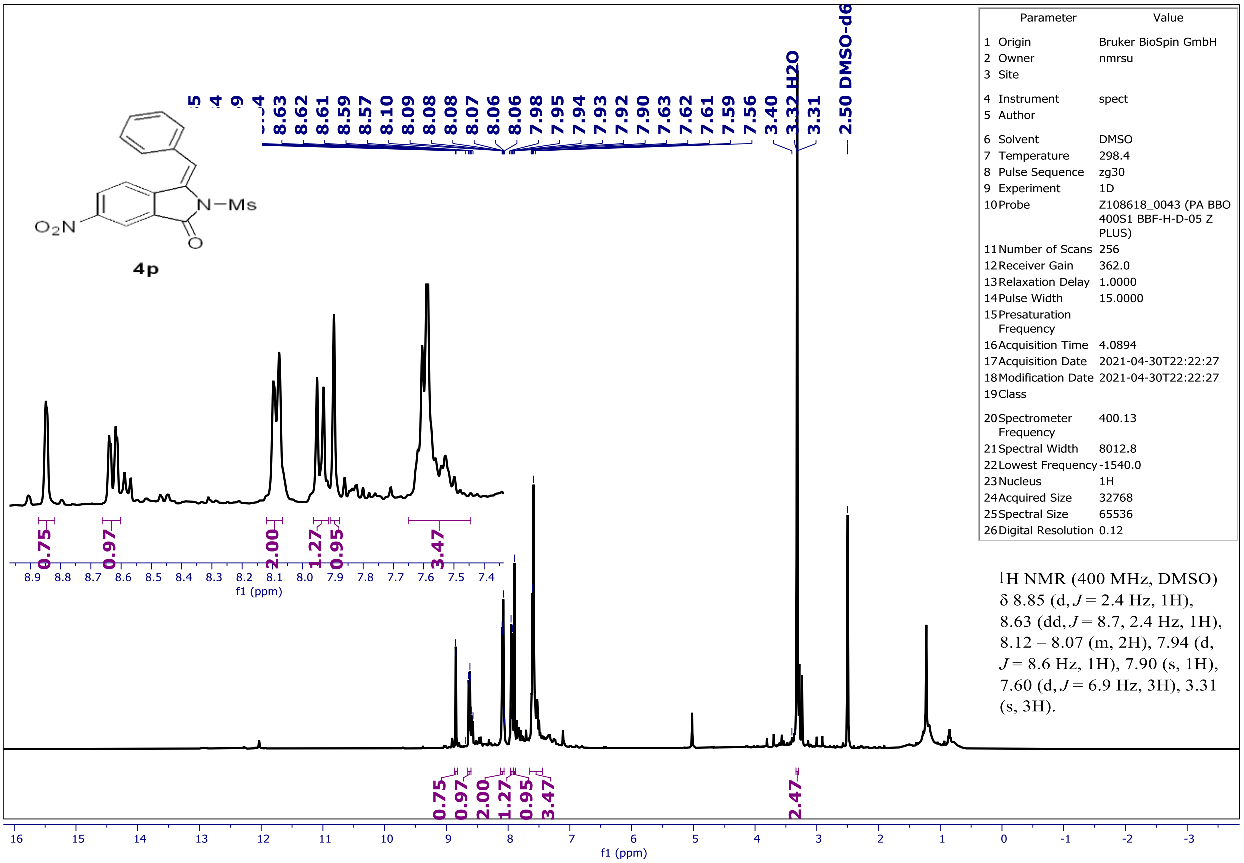

^{1}H NMR (400 MHz, DMSO) δ 8.85 (d, *J* = 2.4 Hz, 1H), 8.63 (dd, *J* = 8.7, 2.4 Hz, 1H), 8.12 – 8.07 (m, 2H), 7.94 (d, *J* = 8.6 Hz, 1H), 7.90 (s, 1H), 7.60 (d, *J* = 6.9 Hz, 3H), 3.31 (s, 3H).

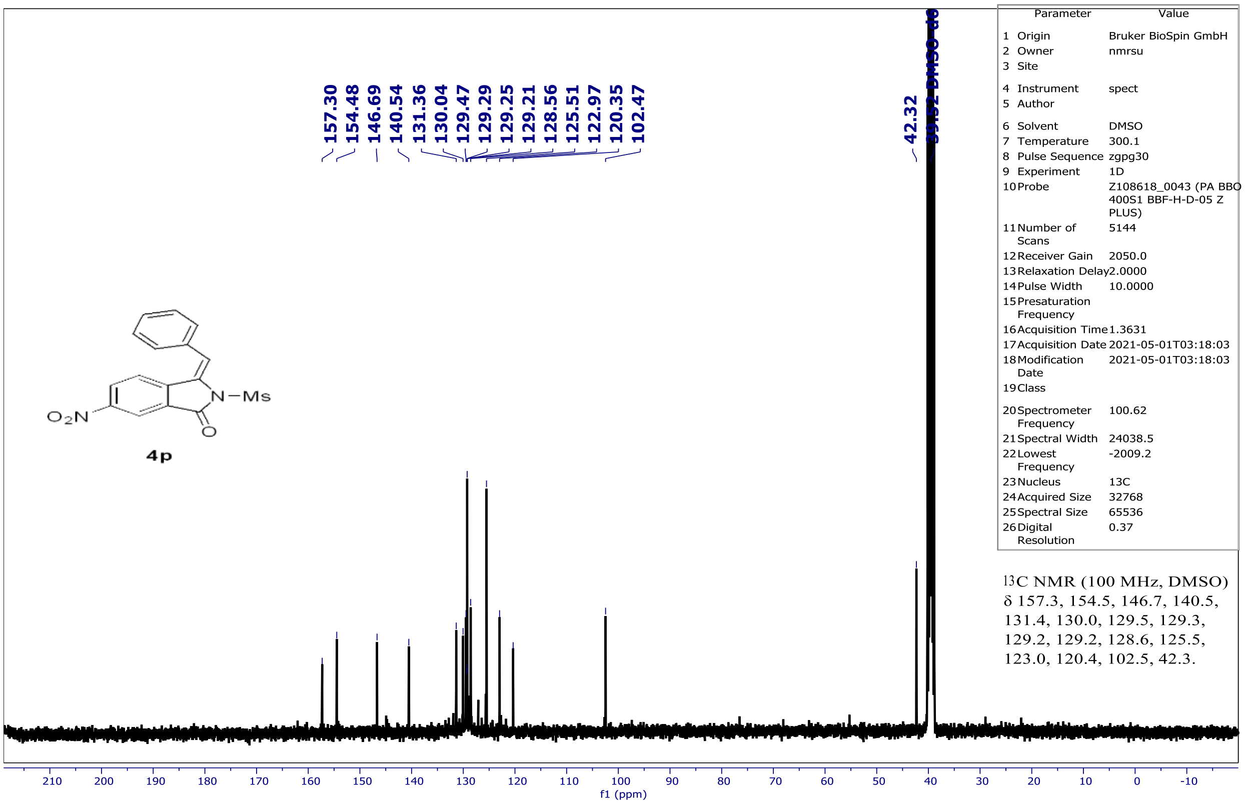

^{13}C NMR (100 MHz, DMSO) δ 157.3, 154.5, 146.7, 140.5, 131.4, 130.0, 129.5, 129.3, 129.2, 129.2, 128.6, 125.5, 123.0, 120.4, 102.5, 42.3.

(*E*)-3-Benzylidene-2-(methylsulfonyl)-6-nitroisoindolin-1-one (4p)

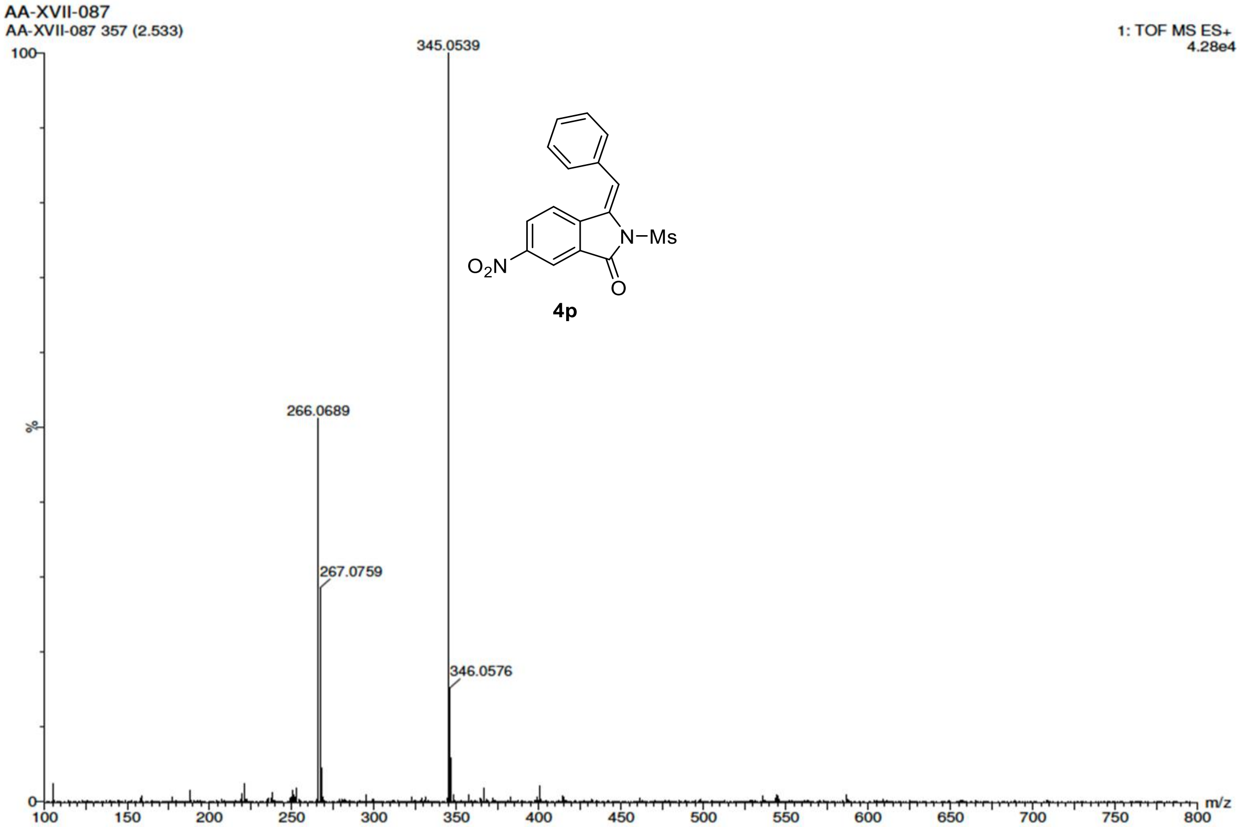

HRMS (ESI) *m/z* calcd for $C_{16}H_{12}N_2O_5S$ $[M + H]^+$ 345.0540; found 345.0539.

(*E*)-3-Benzylidene-5-chloro-2-(methylsulfonyl)isoindolin-1-one (4q)

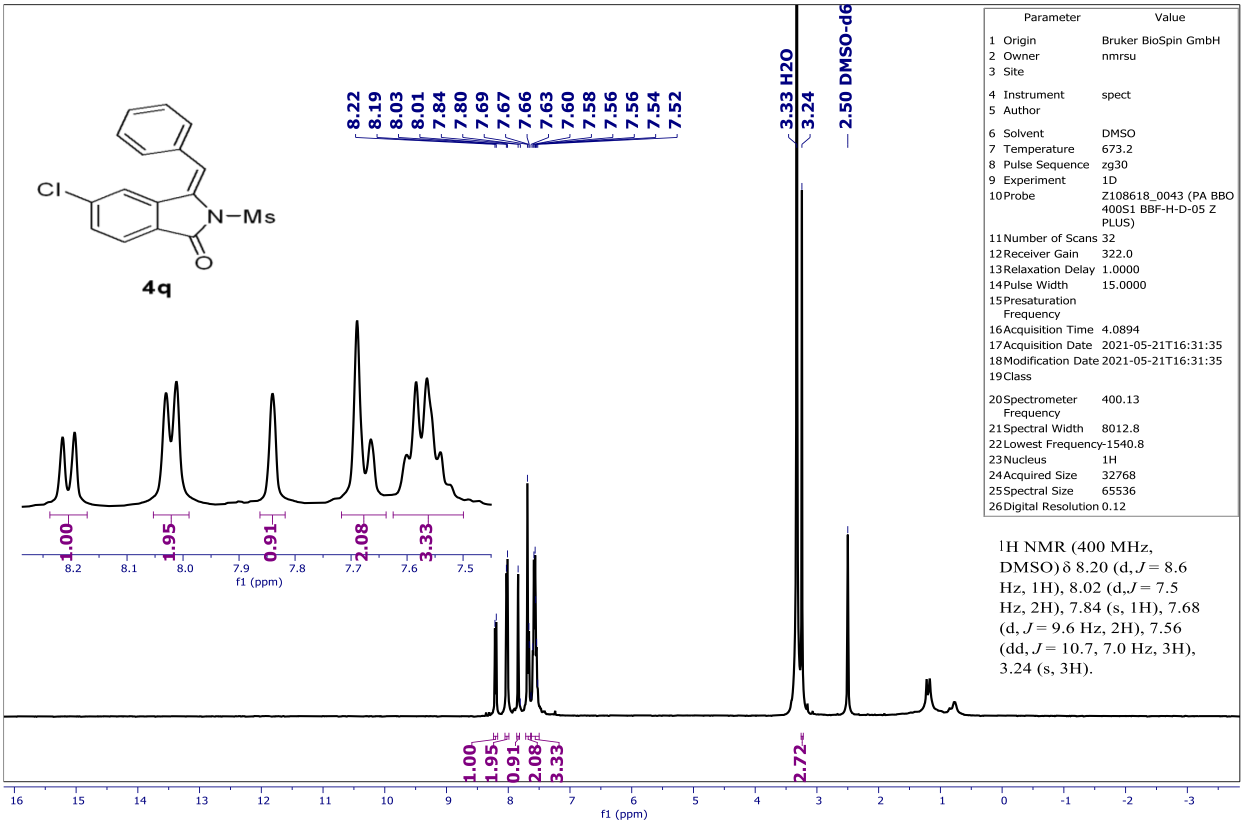

^{1}H NMR (400 MHz, DMSO) δ 8.20 (d, *J* = 8.6 Hz, 1H), 8.02 (d, *J* = 7.5 Hz, 2H), 7.84 (s, 1H), 7.68 (d, *J* = 9.6 Hz, 2H), 7.56 (dd, *J* = 10.7, 7.0 Hz, 3H), 3.24 (s, 3H).

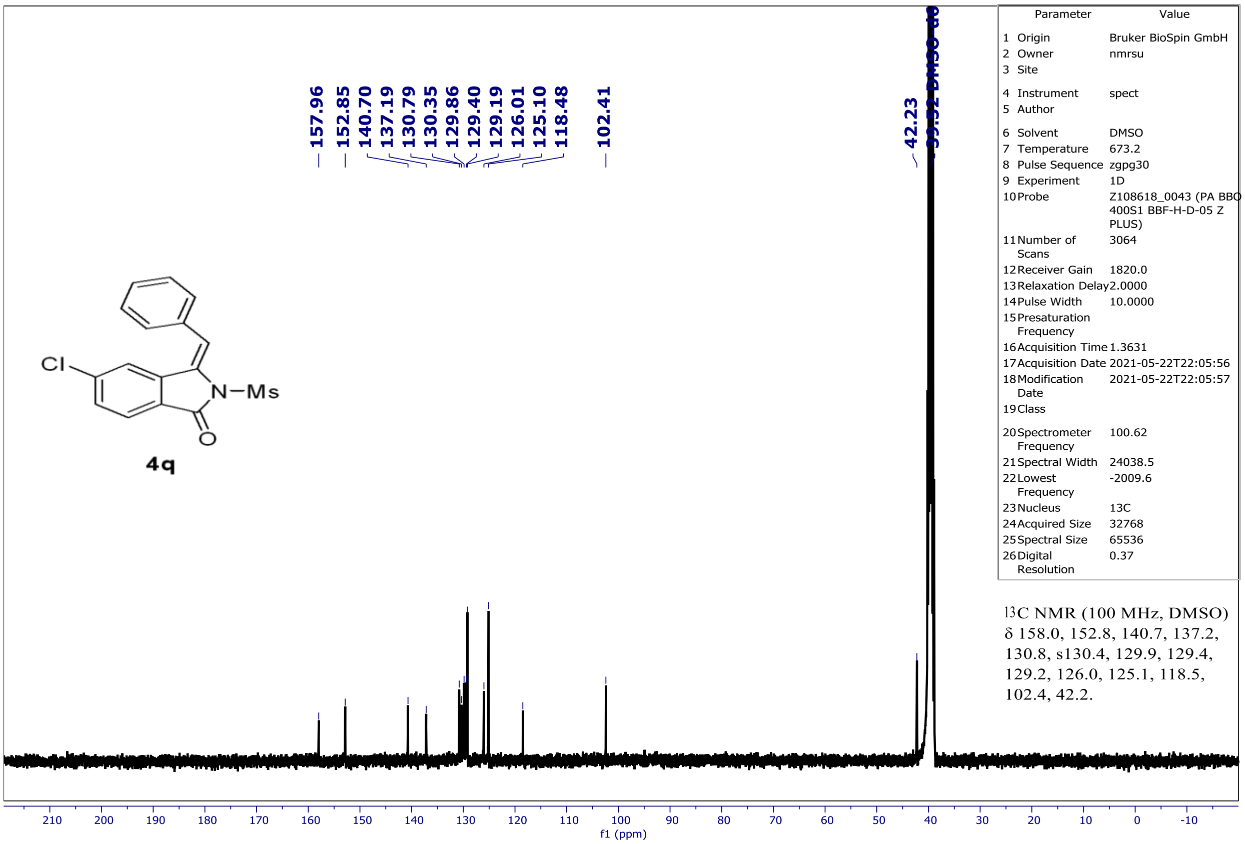

^{13}C NMR (100 MHz, DMSO) δ 158.0, 152.8, 140.7, 137.2, 130.8, s130.4, 129.9, 129.4, 129.2, 126.0, 125.1, 118.5, 102.4, 42.2.

(*E*)-3-Benzylidene-5-chloro-2-(methylsulfonyl)isoindolin-1-one (4q)

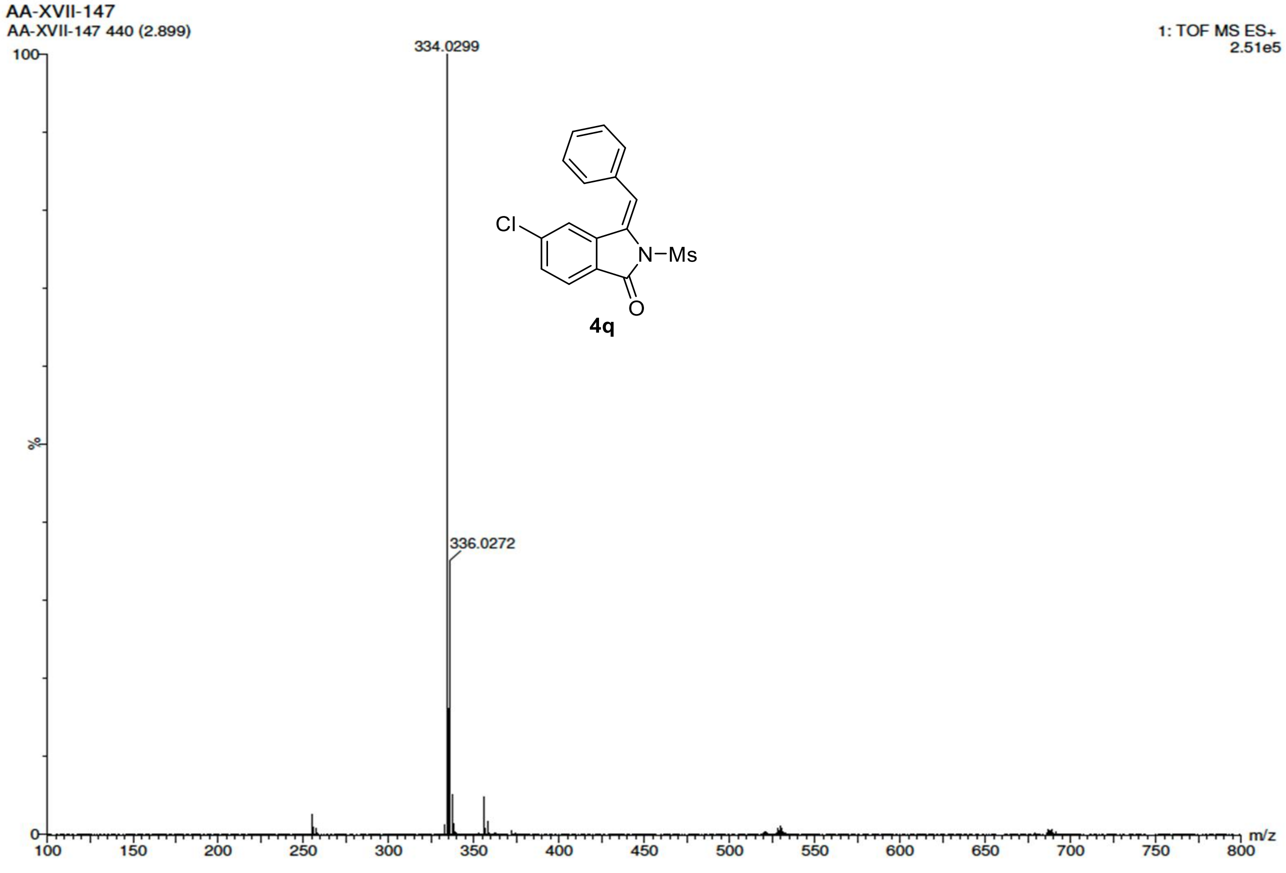

HRMS (ESI) *m/z* calcd for $C_{16}H_{12}ClNO_3S$ $[M + H]^+$ 334.0299; found 334.0299.

(*E*)-3-Benzylidene-2-(methylsulfonyl)-5-nitroisoindolin-1-one (4r)

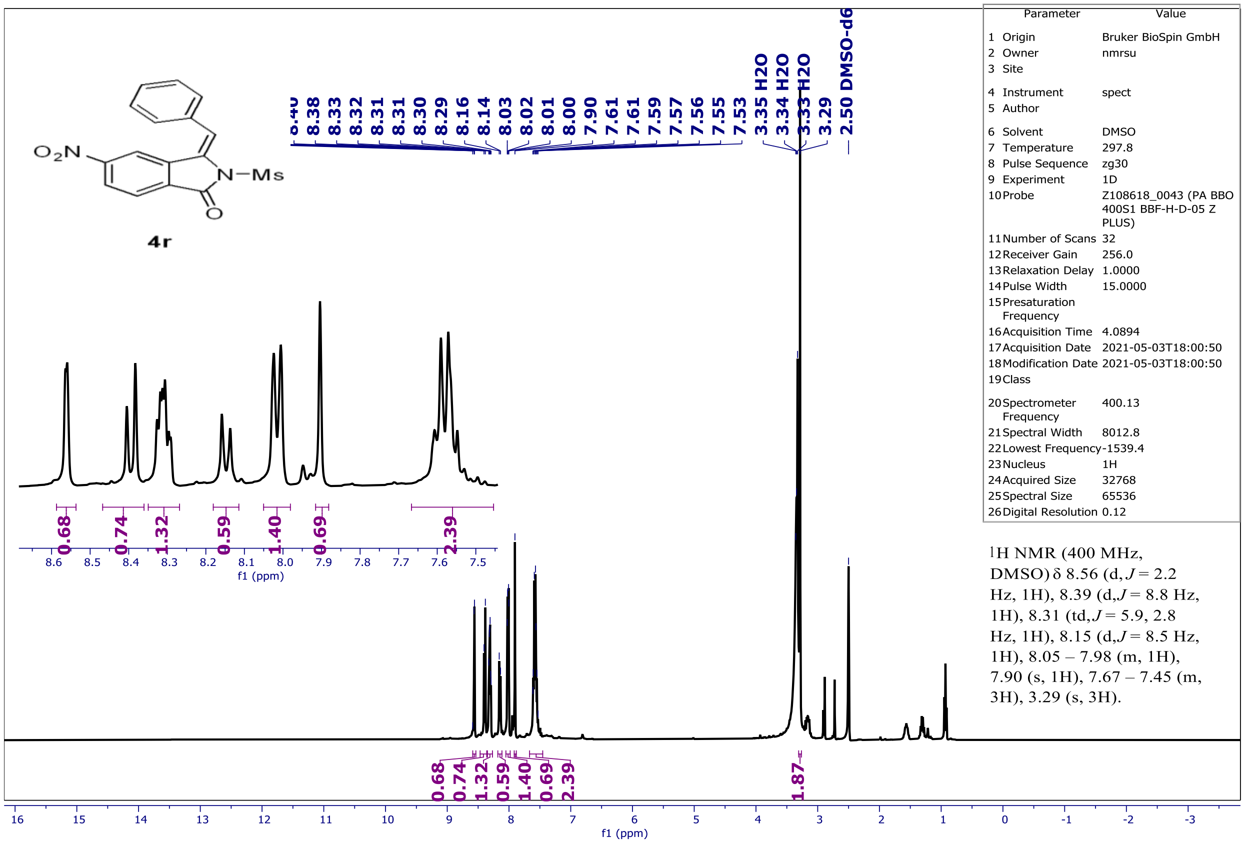

Parameter		Value
1	Origin	Bruker BioSpin GmbH
2	Owner	nmrsu
3	Site	
4	Instrument	spect
5	Author	
6	Solvent	DMSO
7	Temperature	297.8
8	Pulse Sequence	zg30
9	Experiment	1D
10	Probe	Z108618_0043 (PA BBO 400S1 BBF-H-D-05 Z PLUS)
11	Number of Scans	32
12	Receiver Gain	256.0
13	Relaxation Delay	1.0000
14	Pulse Width	15.0000
15	Presaturation Frequency	
16	Acquisition Time	4.0894
17	Acquisition Date	2021-05-03T18:00:50
18	Modification Date	2021-05-03T18:00:50
19	Class	
20	Spectrometer Frequency	400.13
21	Spectral Width	8012.8
22	Lowest Frequency	-1539.4
23	Nucleus	1H
24	Acquired Size	32768
25	Spectral Size	65536
26	Digital Resolution	0.12

1H NMR (400 MHz, DMSO) δ 8.56 (d, *J* = 2.2 Hz, 1H), 8.39 (d, *J* = 8.8 Hz, 1H), 8.31 (td, *J* = 5.9, 2.8 Hz, 1H), 8.15 (d, *J* = 8.5 Hz, 1H), 8.05 – 7.98 (m, 1H), 7.90 (s, 1H), 7.67 – 7.45 (m, 3H), 3.29 (s, 3H).

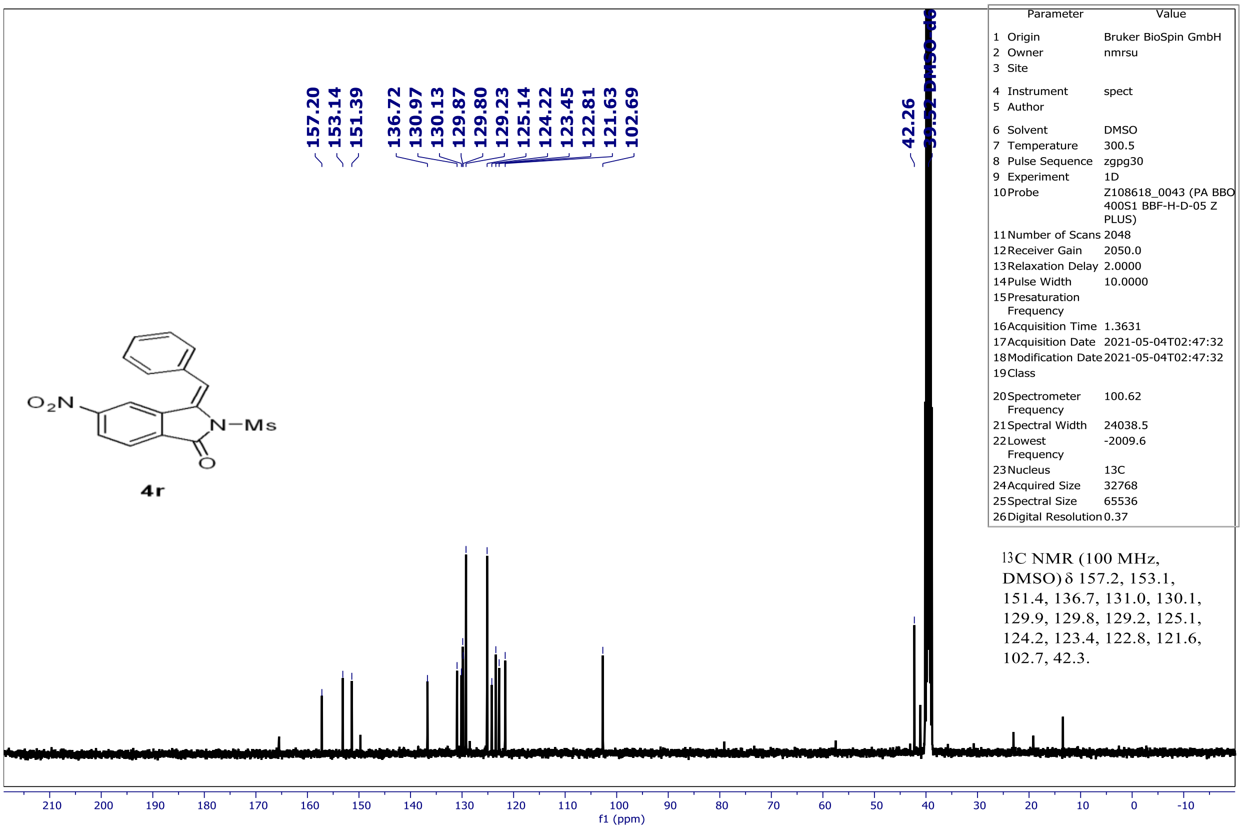
157.20
153.14
151.39
136.72
130.97
130.13
129.87
129.80
129.23
125.14
124.22
123.45
122.81
121.63
102.69
42.26
39.52 DMSO-d6
Parameter Value
1 Origin Bruker BioSpin GmbH
2 Owner nmrsu
3 Site
4 Instrument spect
5 Author
6 Solvent DMSO
7 Temperature 300.5
8 Pulse Sequence zgpg30
9 Experiment 1D
10 Probe Z108618_0043 (PA BBO 400S1 BBF-H-D-05 Z PLUS)
11 Number of Scans 2048
12 Receiver Gain 2050.0
13 Relaxation Delay 2.0000
14 Pulse Width 10.0000
15 Presaturation Frequency
16 Acquisition Time 1.3631
17 Acquisition Date 2021-05-04T02:47:32
18 Modification Date 2021-05-04T02:47:32
19 Class
20 Spectrometer Frequency 100.62
21 Spectral Width 24038.5
22 Lowest Frequency -2009.6
23 Nucleus 13C
24 Acquired Size 32768
25 Spectral Size 65536
26 Digital Resolution 0.37
O2N
N−Ms
O
4r
13C NMR (100 MHz, DMSO) δ 157.2, 153.1, 151.4, 136.7, 131.0, 130.1, 129.9, 129.8, 129.2, 125.1, 124.2, 123.4, 122.8, 121.6, 102.7, 42.3.
210 200 190 180 170 160 150 140 130 120 110 100 90 80 70 60 50 40 30 20 10 0 -10
f1 (ppm)

(*E*)-3-Benzylidene-2-(methylsulfonyl)-5-nitroisoindolin-1-one (4r)

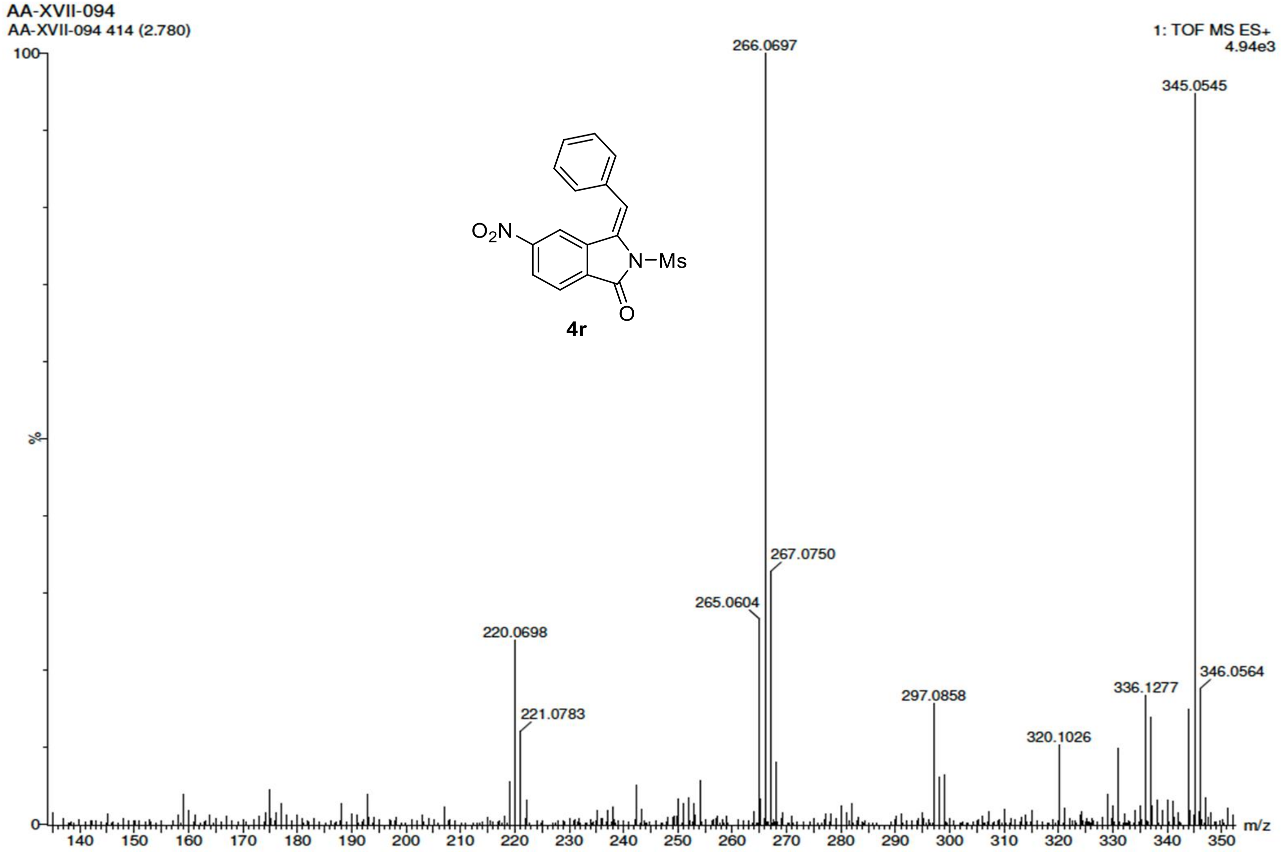

HRMS (ESI) *m/z* calcd for $C_{16}H_{12}N_2O_5S$ $[M + H]^+$ 345.0540; found 345.0545.

(*E*)-2-(Methylsulfonyl)-3-(2-oxopropylidene)isoindolin-1-one (4s)

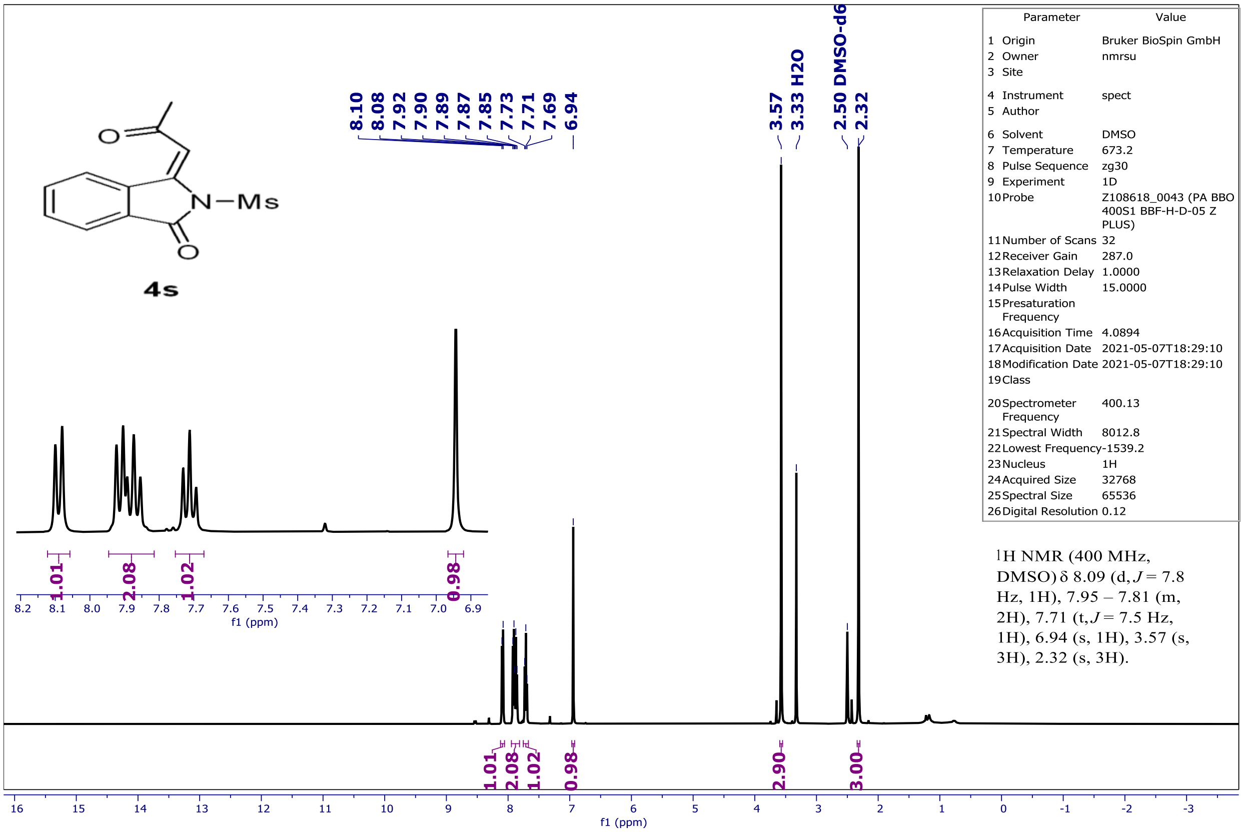

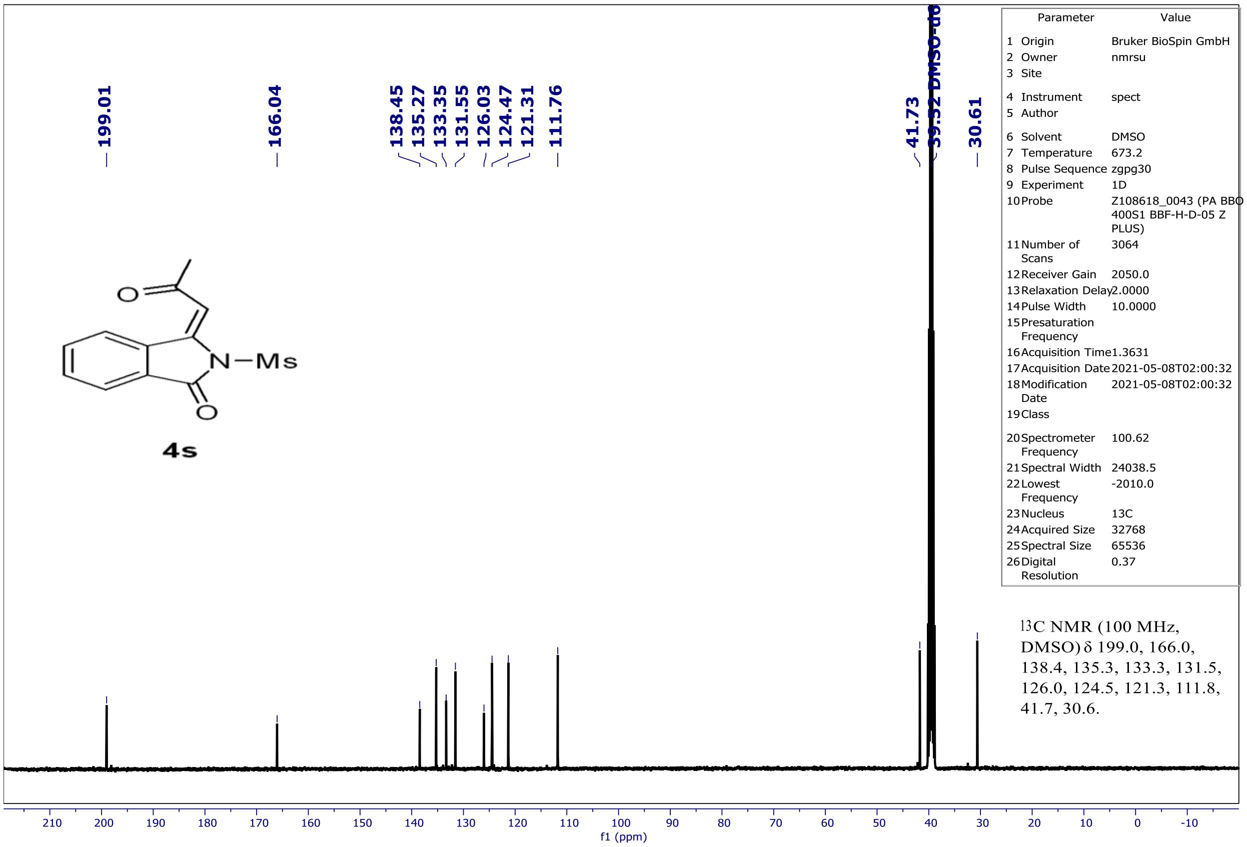

(*E*)-2-(Methylsulfonyl)-3-(2-oxopropylidene)isoindolin-1-one (4s)

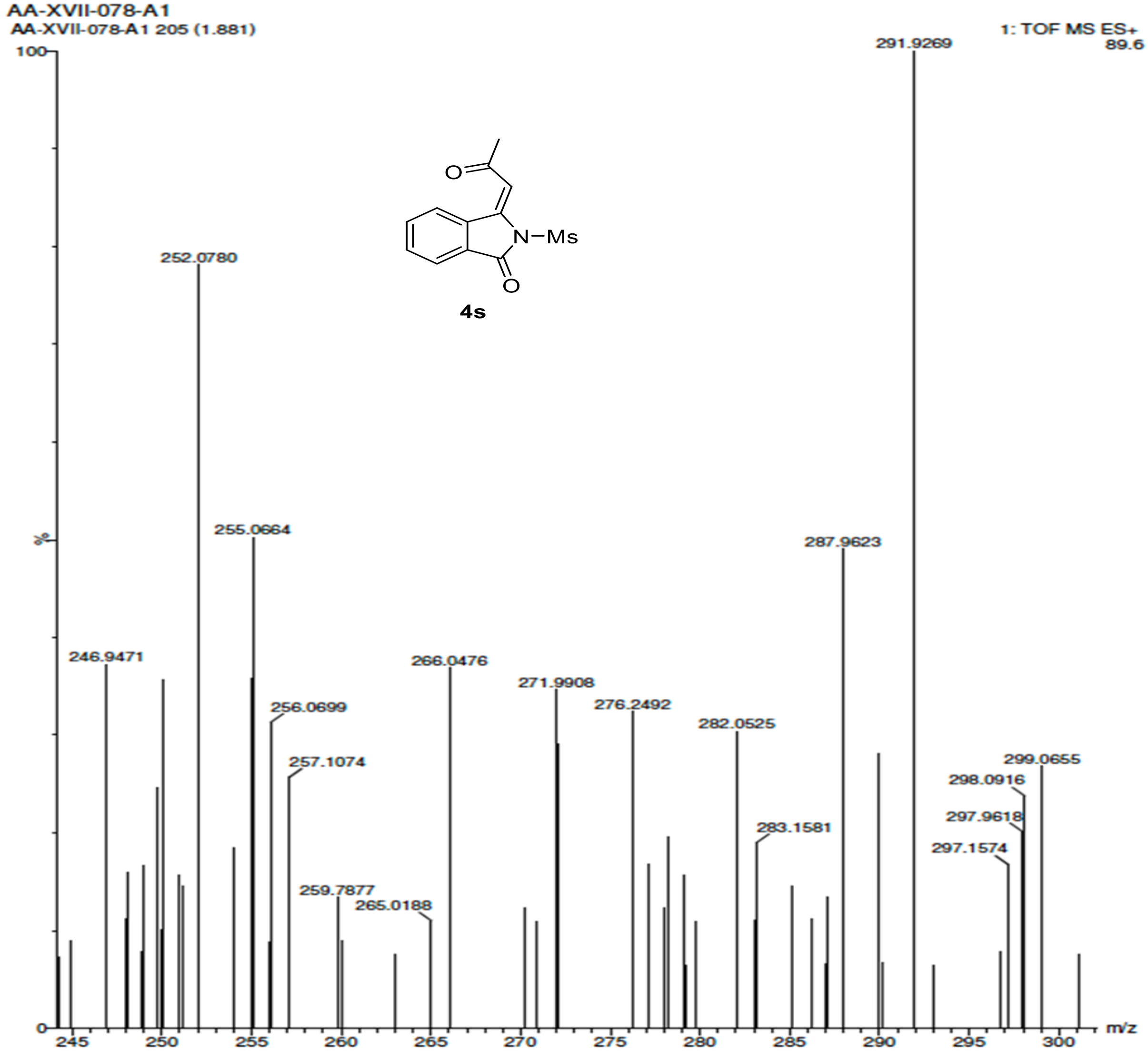

HRMS (ESI) *m/z* calcd for $C_{12}H_{11}NO_4S$ $[M + H]^+$ 266.0482; found 266.0476.

(*E*)-6-Methyl-2-(methylsulfonyl)-3-(2-oxopropylidene)isoindolin-1-one (4t)

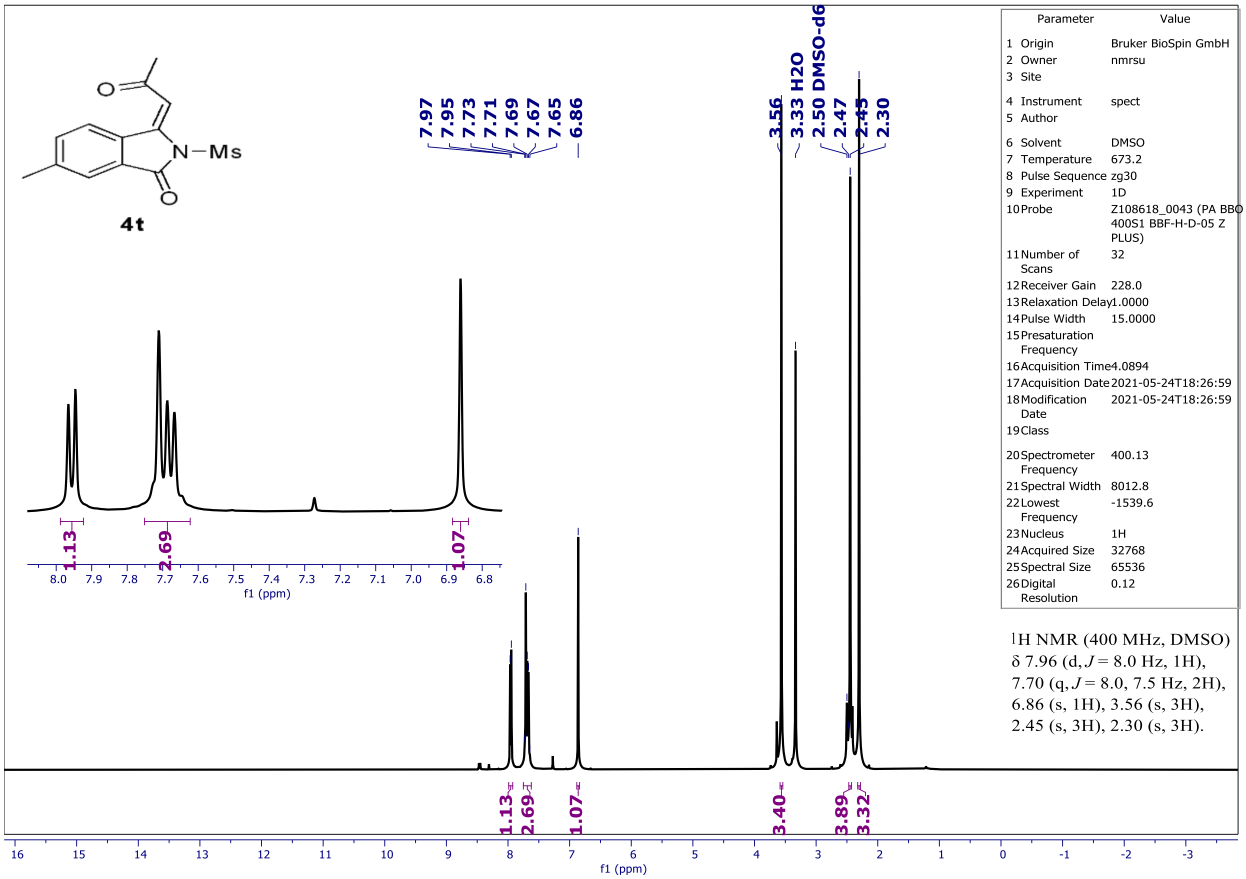

Parameter	Value
1 Origin	Bruker BioSpin GmbH
2 Owner	nmrsu
3 Site	
4 Instrument	spect
5 Author	
6 Solvent	DMSO
7 Temperature	673.2
8 Pulse Sequence	zg30
9 Experiment	1D
10 Probe	Z108618_0043 (PA BBO 400S1 BBF-H-D-05 Z PLUS)
11 Number of Scans	32
12 Receiver Gain	228.0
13 Relaxation Delay	1.0000
14 Pulse Width	15.0000
15 Presaturation Frequency	
16 Acquisition Time	4.0894
17 Acquisition Date	2021-05-24T18:26:59
18 Modification Date	2021-05-24T18:26:59
19 Class	
20 Spectrometer Frequency	400.13
21 Spectral Width	8012.8
22 Lowest Frequency	-1539.6
23 Nucleus	1H
24 Acquired Size	32768
25 Spectral Size	65536
26 Digital Resolution	0.12

^{1}H NMR (400 MHz, DMSO) δ 7.96 (d, *J* = 8.0 Hz, 1H), 7.70 (q, *J* = 8.0, 7.5 Hz, 2H), 6.86 (s, 1H), 3.56 (s, 3H), 2.45 (s, 3H), 2.30 (s, 3H).

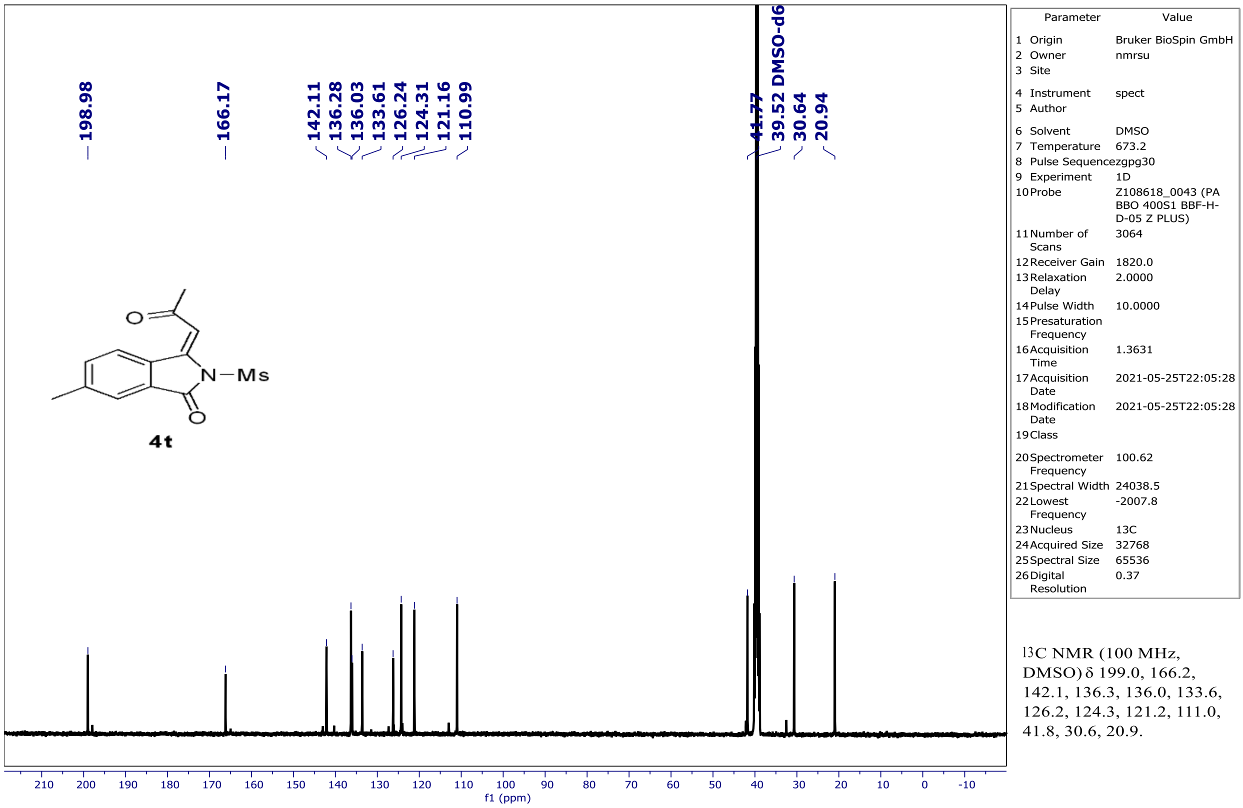

Parameter	Value
1 Origin	Bruker BioSpin GmbH
2 Owner	nmrsu
3 Site	
4 Instrument	spect
5 Author	
6 Solvent	DMSO
7 Temperature	673.2
8 Pulse Sequence	zgpg30
9 Experiment	1D
10 Probe	Z108618_0043 (PA BBO 400S1 BBF-H-D-05 Z PLUS)
11 Number of Scans	3064
12 Receiver Gain	1820.0
13 Relaxation Delay	2.0000
14 Pulse Width	10.0000
15 Presaturation Frequency	
16 Acquisition Time	1.3631
17 Acquisition Date	2021-05-25T22:05:28
18 Modification Date	2021-05-25T22:05:28
19 Class	
20 Spectrometer Frequency	100.62
21 Spectral Width	24038.5
22 Lowest Frequency	-2007.8
23 Nucleus	13C
24 Acquired Size	32768
25 Spectral Size	65536
26 Digital Resolution	0.37

^{13}C NMR (100 MHz, DMSO) δ 199.0, 166.2, 142.1, 136.3, 136.0, 133.6, 126.2, 124.3, 121.2, 111.0, 41.8, 30.6, 20.9.

(*E*)-6-Methyl-2-(methylsulfonyl)-3-(2-oxopropylidene)isoindolin-1-one (4t)

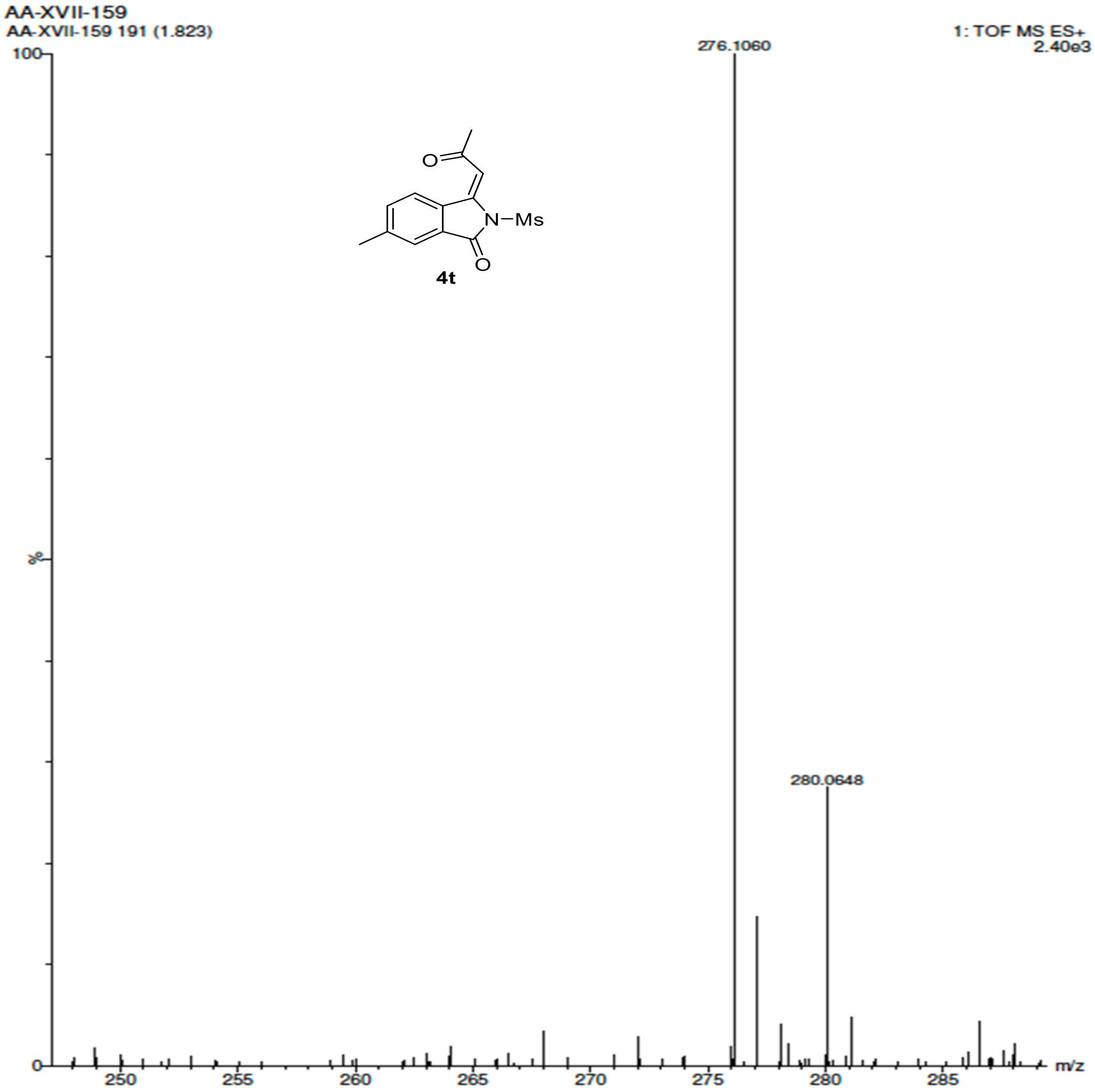

HRMS (ESI) *m/z* calcd for $C_{13}H_{13}NO_4S$ $[M + H]^+$ 280.0638; found 280.0648.

(*E*)-6-Methoxy-2-(methylsulfonyl)-3-(2-oxopropylidene)isoindolin-1-one (4u)

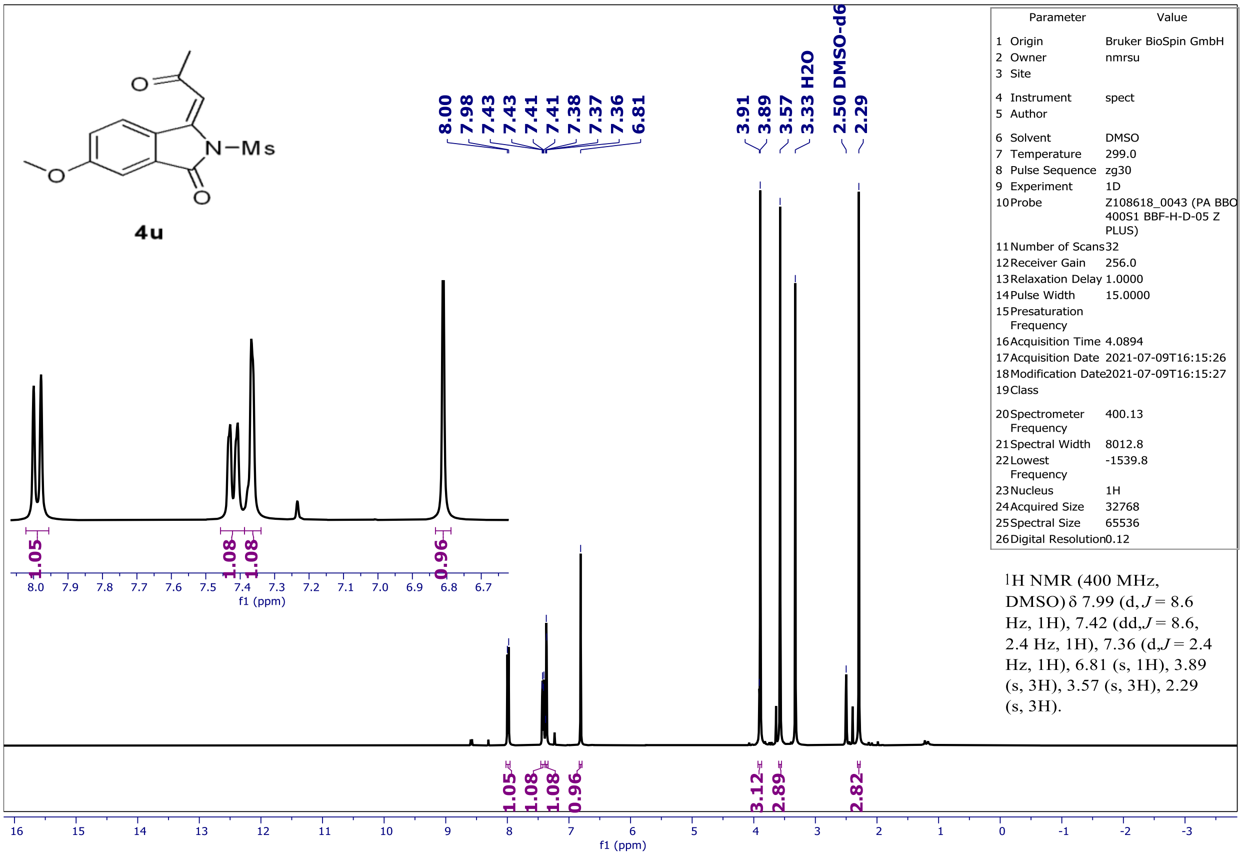

^{1}H NMR (400 MHz, DMSO) δ 7.99 (d, *J* = 8.6 Hz, 1H), 7.42 (dd, *J* = 8.6, 2.4 Hz, 1H), 7.36 (d, *J* = 2.4 Hz, 1H), 6.81 (s, 1H), 3.89 (s, 3H), 3.57 (s, 3H), 2.29 (s, 3H).

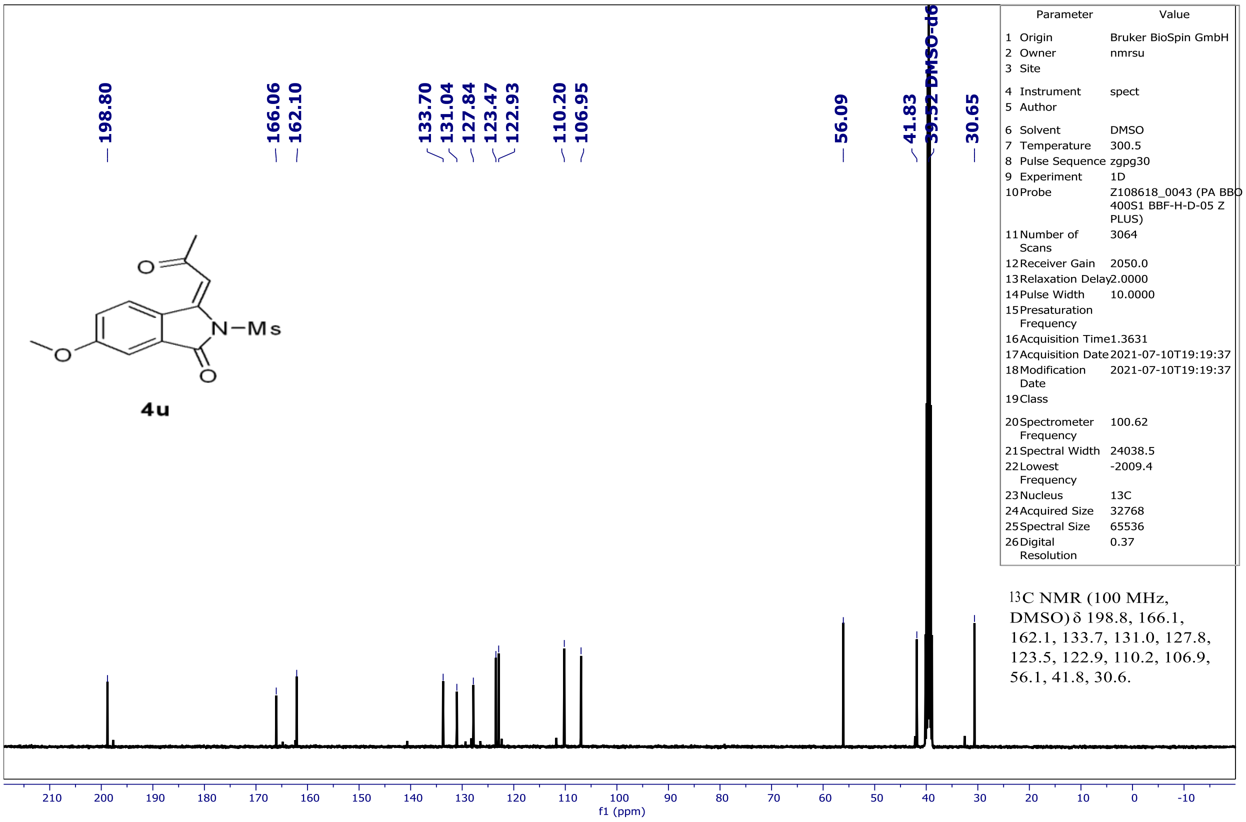

^{13}C NMR (100 MHz, DMSO) δ 198.8, 166.1, 162.1, 133.7, 131.0, 127.8, 123.5, 122.9, 110.2, 106.9, 56.1, 41.8, 30.6.

(*E*)-6-Methoxy-2-(methylsulfonyl)-3-(2-oxopropylidene)isoindolin-1-one (4u)

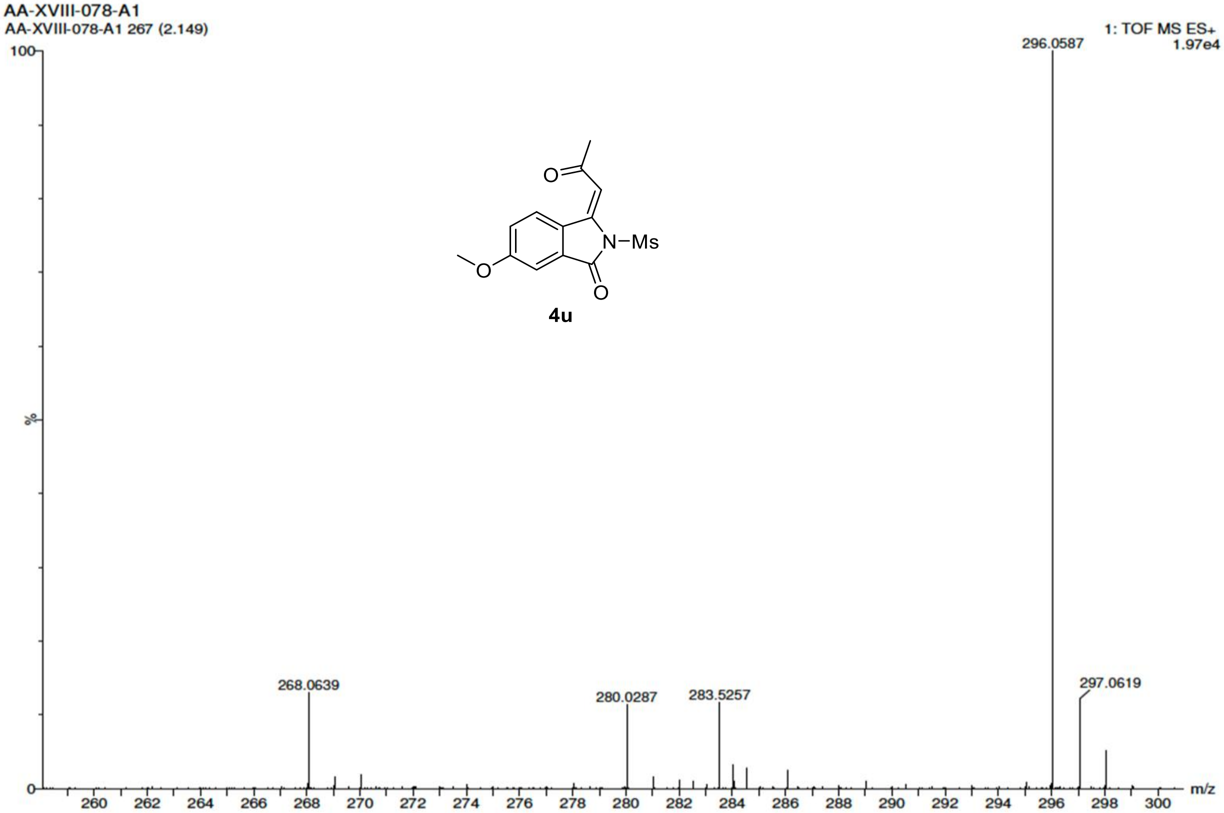

HRMS (ESI) *m/z* calcd for $C_{13}H_{13}NO_5S$ $[M + H]^+$ 296.0587; found 296.0587.

Ethyl (*E*)-2-(3-oxoisoindolin-1-ylidene)acetate (5a)

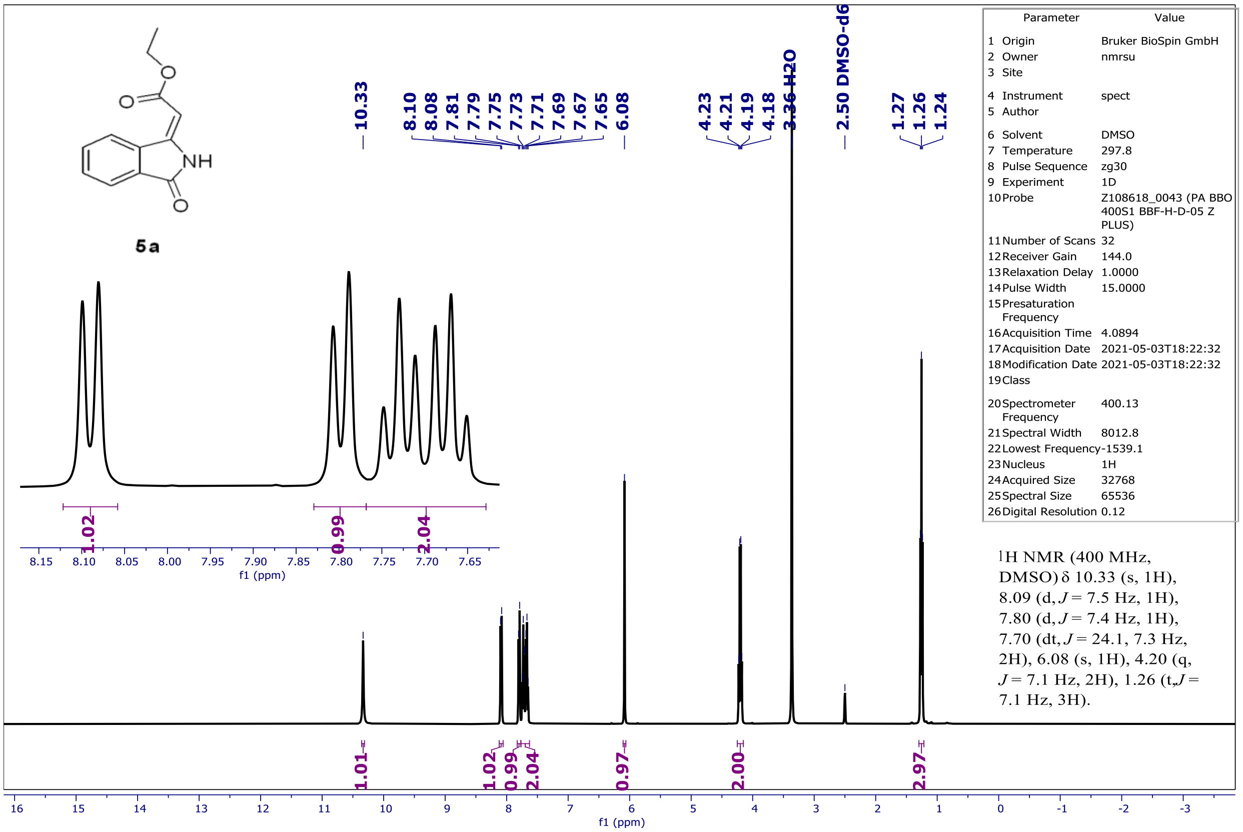

^{1}H NMR (400 MHz, DMSO) δ 10.33 (s, 1H), 8.09 (d, *J* = 7.5 Hz, 1H), 7.80 (d, *J* = 7.4 Hz, 1H), 7.70 (dt, *J* = 24.1, 7.3 Hz, 2H), 6.08 (s, 1H), 4.20 (q, *J* = 7.1 Hz, 2H), 1.26 (t, *J* = 7.1 Hz, 3H).

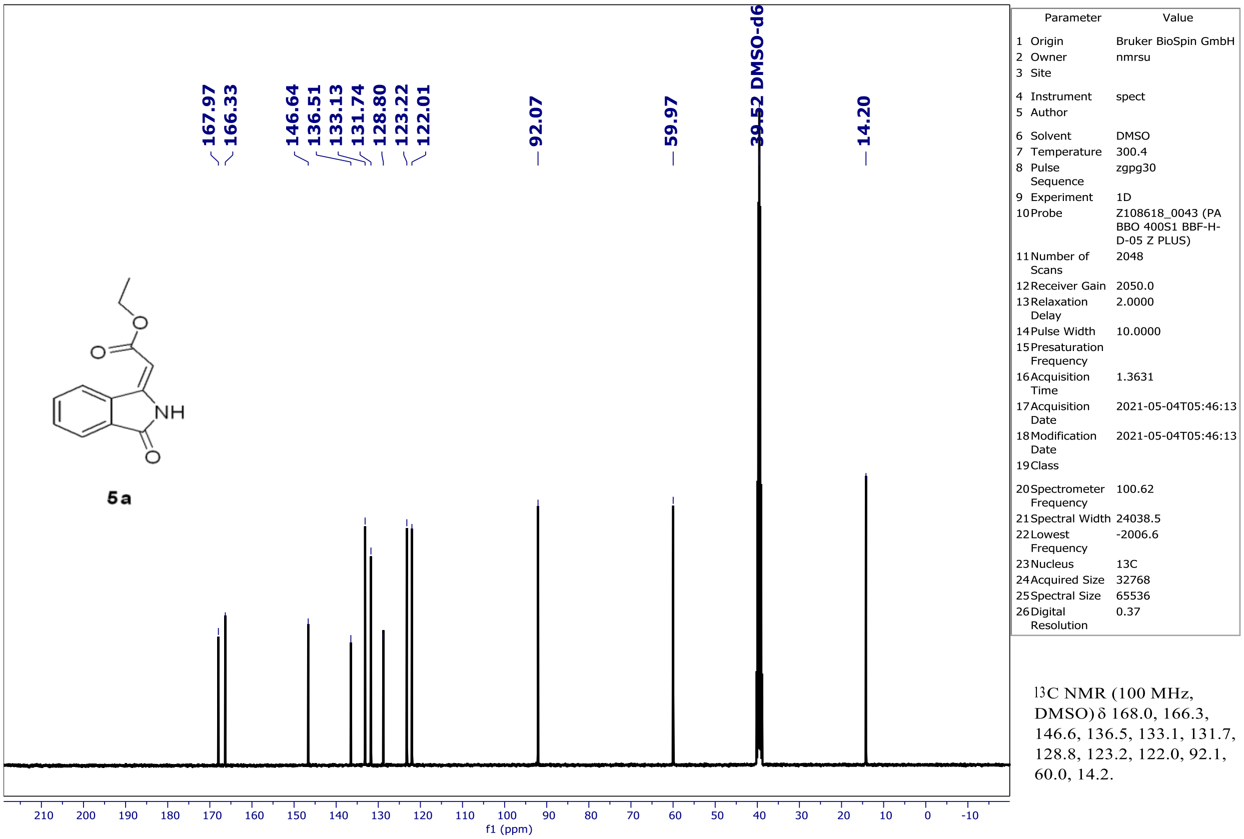

^{13}C NMR (100 MHz, DMSO) δ 168.0, 166.3, 146.6, 136.5, 133.1, 131.7, 128.8, 123.2, 122.0, 92.1, 60.0, 14.2.

Ethyl (*E*)-2-(3-oxoisoindolin-1-ylidene)acetate (5a)

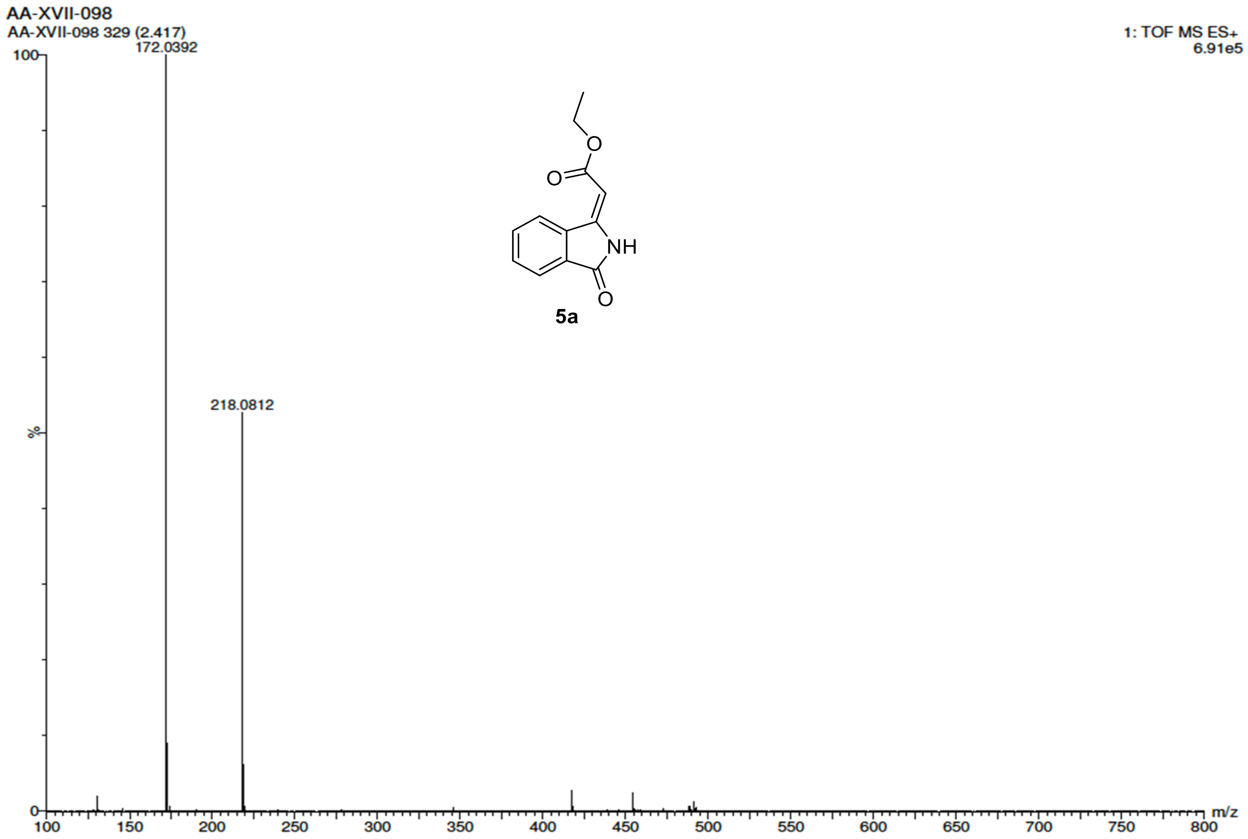

HRMS (ESI) *m/z* calcd for $C_{12}H_{11}NO_3$ $[M + H]^+$ 218.0812; found 218.0812.

Ethyl (*E*)-2-(5-fluoro-3-oxoisoindolin-1-ylidene)acetate (5b)

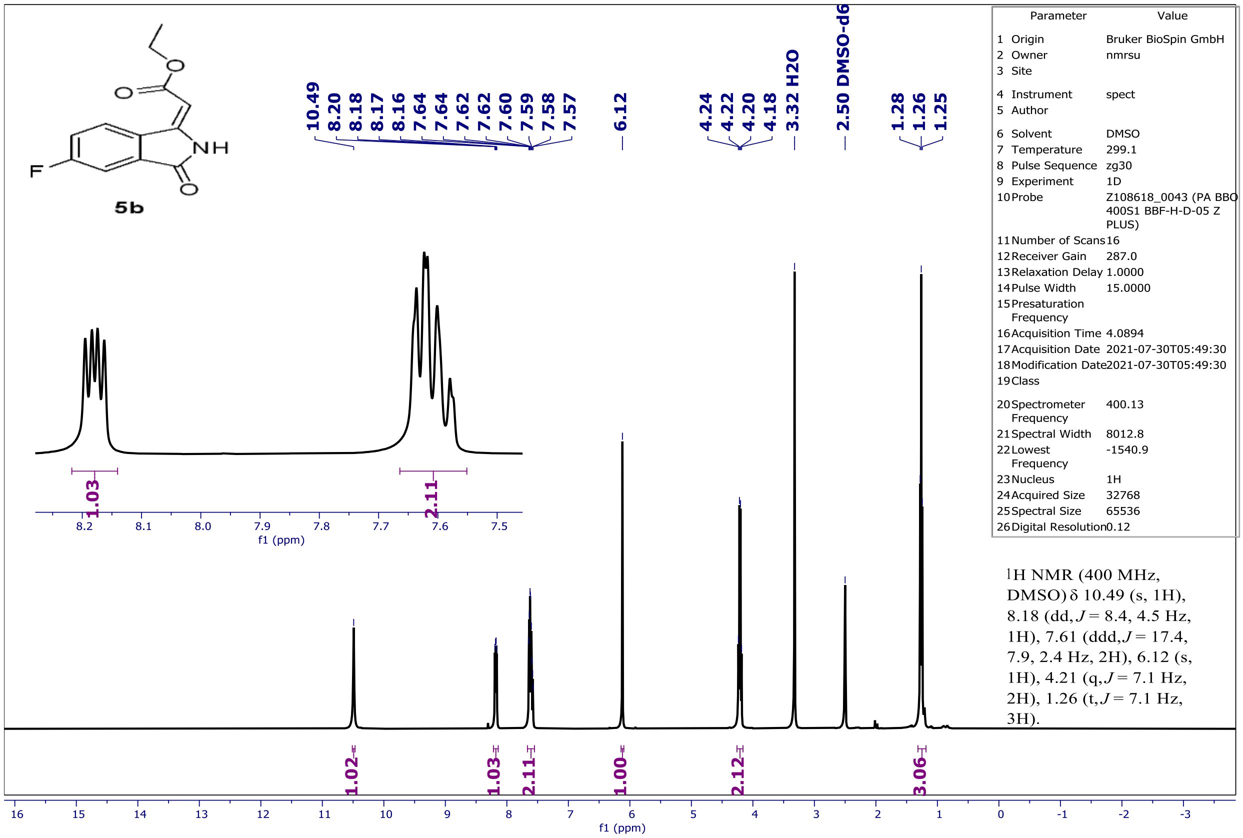

Parameter	Value
1 Origin	Bruker BioSpin GmbH
2 Owner	nmrsu
3 Site	
4 Instrument	spect
5 Author	
6 Solvent	DMSO
7 Temperature	299.1
8 Pulse Sequence	zg30
9 Experiment	1D
10 Probe	Z108618_0043 (PA BBO 400S1 BBF-H-D-05 Z PLUS)
11 Number of Scans	16
12 Receiver Gain	287.0
13 Relaxation Delay	1.0000
14 Pulse Width	15.0000
15 Presaturation Frequency	
16 Acquisition Time	4.0894
17 Acquisition Date	2021-07-30T05:49:30
18 Modification Date	2021-07-30T05:49:30
19 Class	
20 Spectrometer Frequency	400.13
21 Spectral Width	8012.8
22 Lowest Frequency	-1540.9
23 Nucleus	1H
24 Acquired Size	32768
25 Spectral Size	65536
26 Digital Resolution	0.12

^{1}H NMR (400 MHz, DMSO) δ 10.49 (s, 1H), 8.18 (dd, *J* = 8.4, 4.5 Hz, 1H), 7.61 (ddd, *J* = 17.4, 7.9, 2.4 Hz, 2H), 6.12 (s, 1H), 4.21 (q, *J* = 7.1 Hz, 2H), 1.26 (t, *J* = 7.1 Hz, 3H).

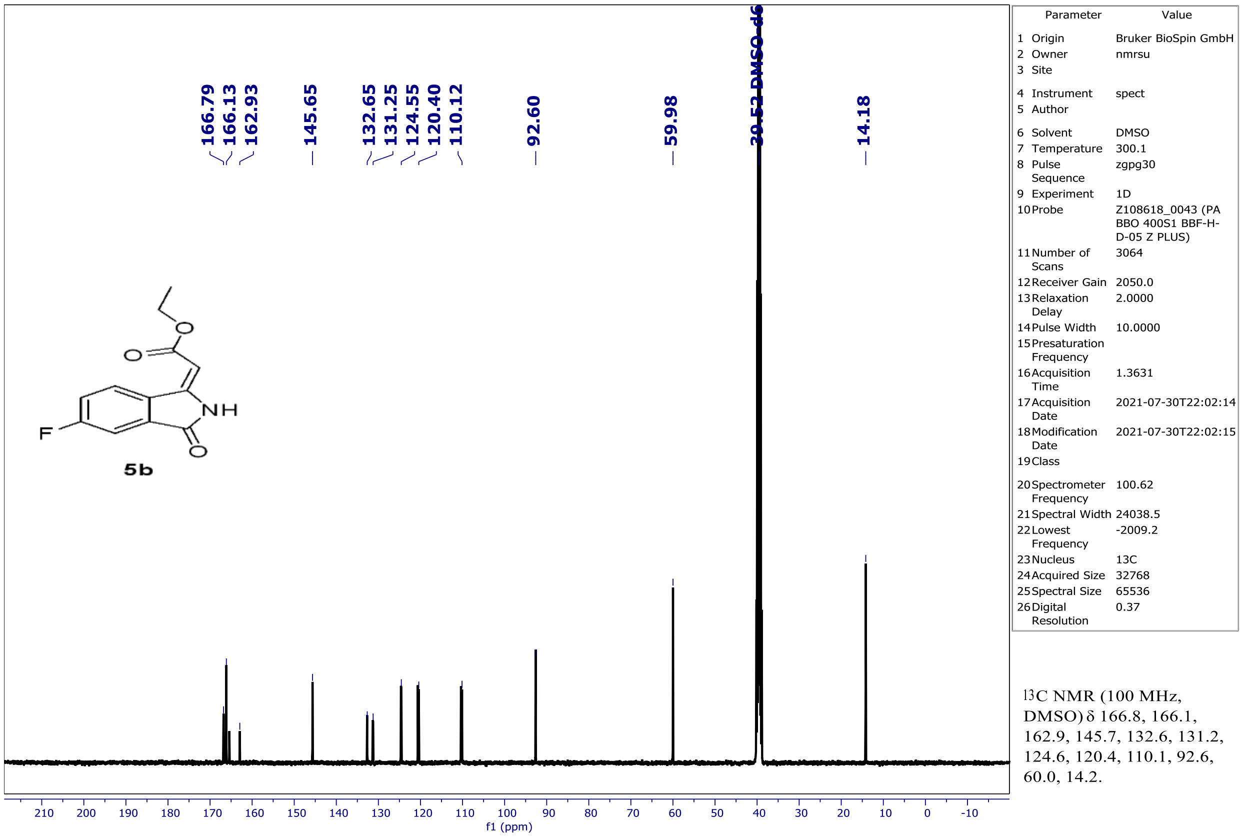

Parameter	Value
1 Origin	Bruker BioSpin GmbH
2 Owner	nmrsu
3 Site	
4 Instrument	spect
5 Author	
6 Solvent	DMSO
7 Temperature	300.1
8 Pulse Sequence	zgpg30
9 Experiment	1D
10 Probe	Z108618_0043 (PA BBO 400S1 BBF-H-D-05 Z PLUS)
11 Number of Scans	3064
12 Receiver Gain	2050.0
13 Relaxation Delay	2.0000
14 Pulse Width	10.0000
15 Presaturation Frequency	
16 Acquisition Time	1.3631
17 Acquisition Date	2021-07-30T22:02:14
18 Modification Date	2021-07-30T22:02:15
19 Class	
20 Spectrometer Frequency	100.62
21 Spectral Width	24038.5
22 Lowest Frequency	-2009.2
23 Nucleus	13C
24 Acquired Size	32768
25 Spectral Size	65536
26 Digital Resolution	0.37

^{13}C NMR (100 MHz, DMSO) δ 166.8, 166.1, 162.9, 145.7, 132.6, 131.2, 124.6, 120.4, 110.1, 92.6, 60.0, 14.2.

Ethyl (*E*)-2-(5-fluoro-3-oxoisoindolin-1-ylidene)acetate (5b)

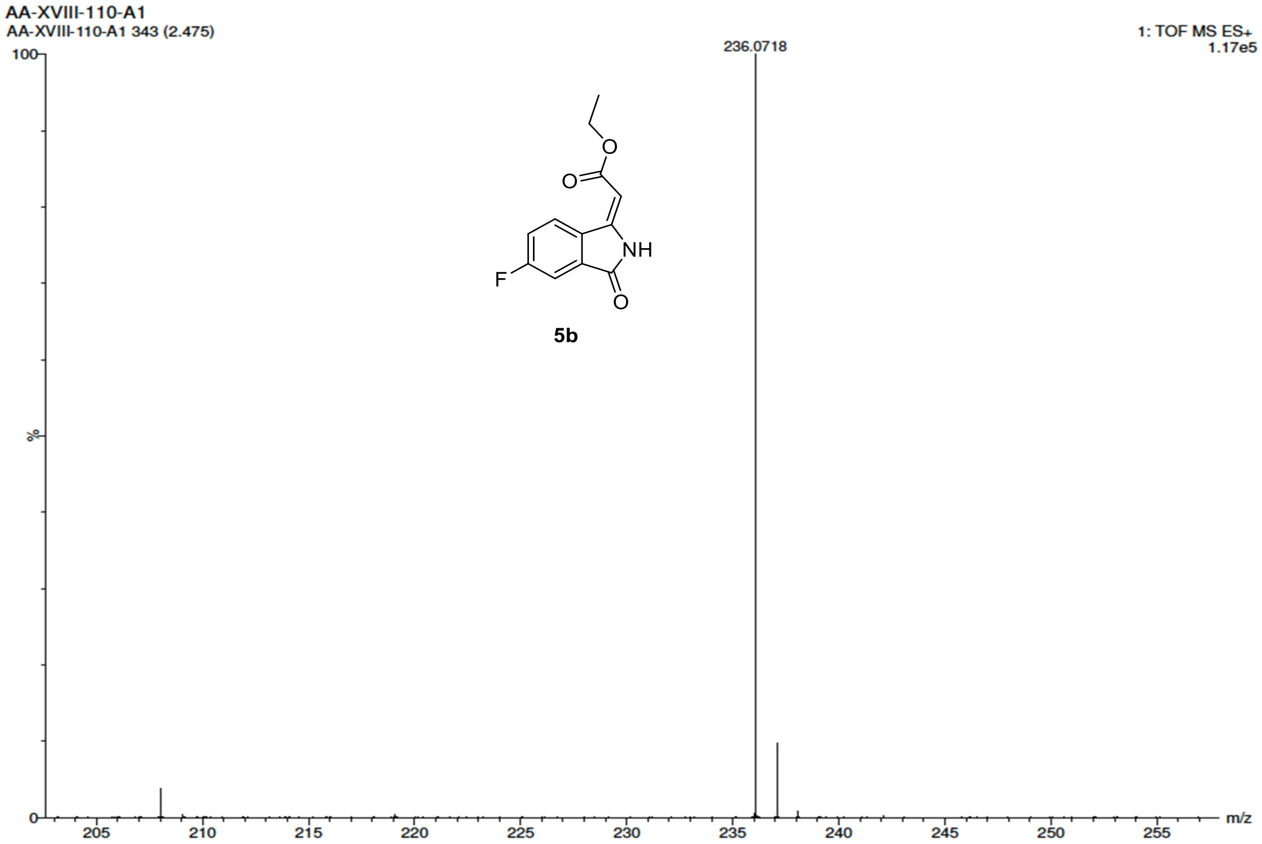

HRMS (ESI) *m/z* calcd for $C_{12}H_{10}FNO_3$ $[M + H]^+$ 236.0717; found 236.0718.

Ethyl (*E*)-2-(5-chloro-3-oxoisoindolin-1-ylidene)acetate (5c)

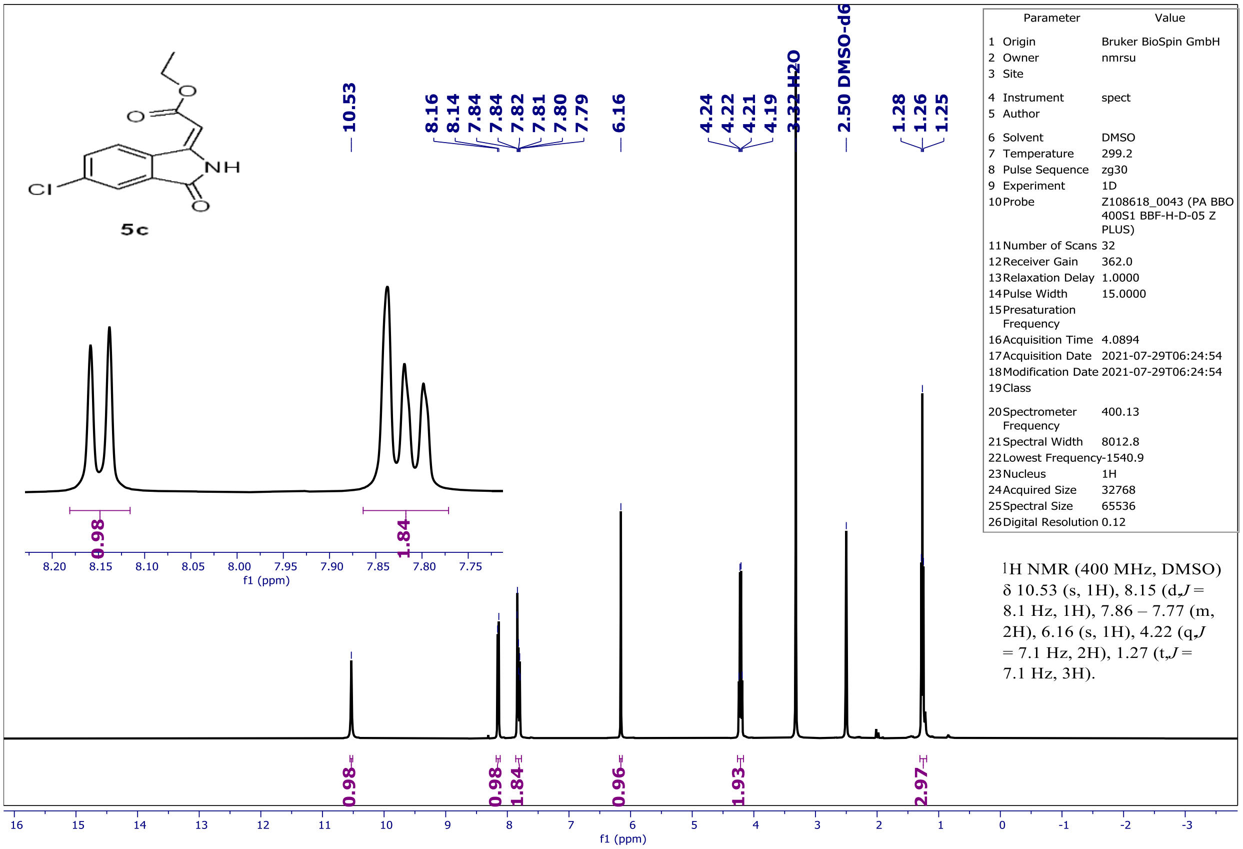

	Parameter	Value
1	Origin	Bruker BioSpin GmbH
2	Owner	nmrsu
3	Site	
4	Instrument	spect
5	Author	
6	Solvent	DMSO
7	Temperature	299.2
8	Pulse Sequence	zg30
9	Experiment	1D
10	Probe	Z108618_0043 (PA BBO 400S1 BBF-H-D-05 Z PLUS)
11	Number of Scans	32
12	Receiver Gain	362.0
13	Relaxation Delay	1.0000
14	Pulse Width	15.0000
15	Presaturation Frequency	
16	Acquisition Time	4.0894
17	Acquisition Date	2021-07-29T06:24:54
18	Modification Date	2021-07-29T06:24:54
19	Class	
20	Spectrometer Frequency	400.13
21	Spectral Width	8012.8
22	Lowest Frequency	-1540.9
23	Nucleus	1H
24	Acquired Size	32768
25	Spectral Size	65536
26	Digital Resolution	0.12

^{1}H NMR (400 MHz, DMSO) δ 10.53 (s, 1H), 8.15 (d, *J* = 8.1 Hz, 1H), 7.86 – 7.77 (m, 2H), 6.16 (s, 1H), 4.22 (q, *J* = 7.1 Hz, 2H), 1.27 (t, *J* = 7.1 Hz, 3H).

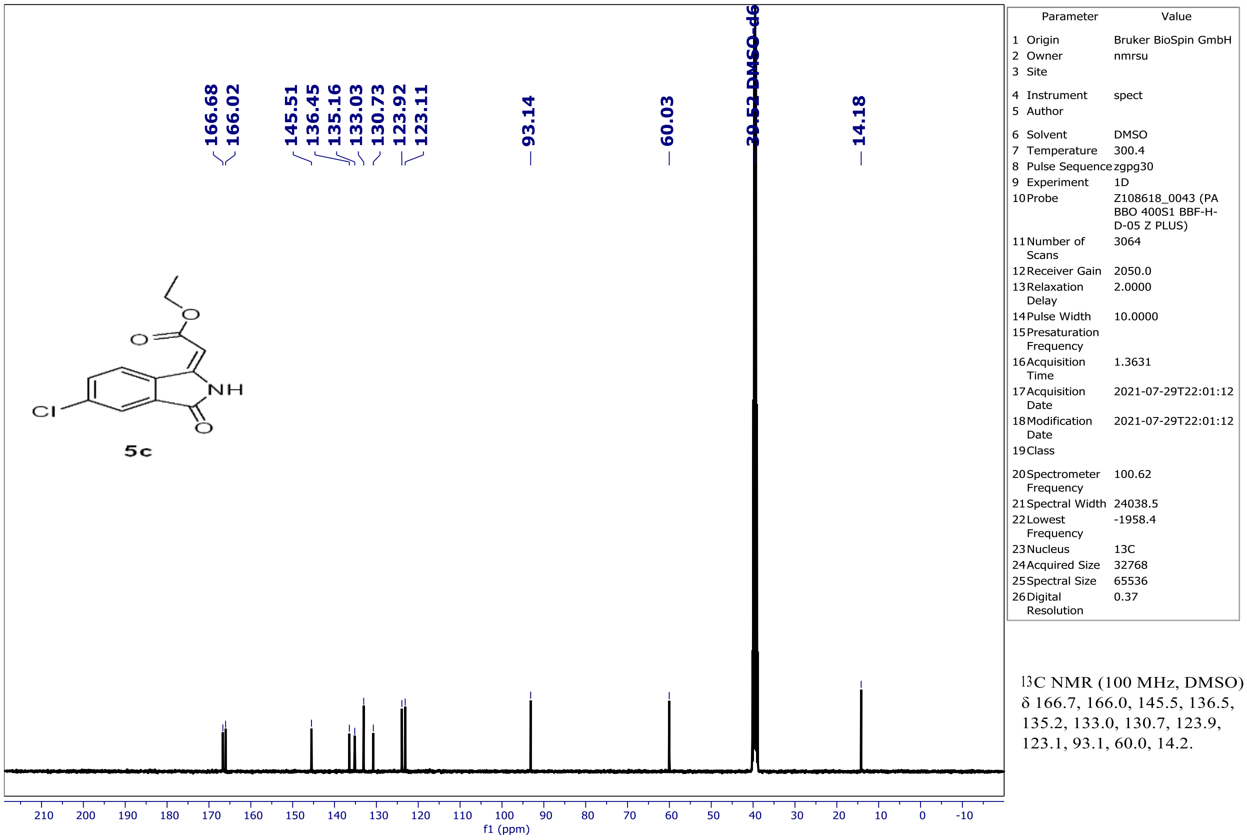

	Parameter	Value
1	Origin	Bruker BioSpin GmbH
2	Owner	nmrsu
3	Site	
4	Instrument	spect
5	Author	
6	Solvent	DMSO
7	Temperature	300.4
8	Pulse Sequence	zgpg30
9	Experiment	1D
10	Probe	Z108618_0043 (PA BBO 400S1 BBF-H-D-05 Z PLUS)
11	Number of Scans	3064
12	Receiver Gain	2050.0
13	Relaxation Delay	2.0000
14	Pulse Width	10.0000
15	Presaturation Frequency	
16	Acquisition Time	1.3631
17	Acquisition Date	2021-07-29T22:01:12
18	Modification Date	2021-07-29T22:01:12
19	Class	
20	Spectrometer Frequency	100.62
21	Spectral Width	24038.5
22	Lowest Frequency	-1958.4
23	Nucleus	13C
24	Acquired Size	32768
25	Spectral Size	65536
26	Digital Resolution	0.37

^{13}C NMR (100 MHz, DMSO) δ 166.7, 166.0, 145.5, 136.5, 135.2, 133.0, 130.7, 123.9, 123.1, 93.1, 60.0, 14.2.

Ethyl (*E*)-2-(5-chloro-3-oxoisoindolin-1-ylidene)acetate (5c)

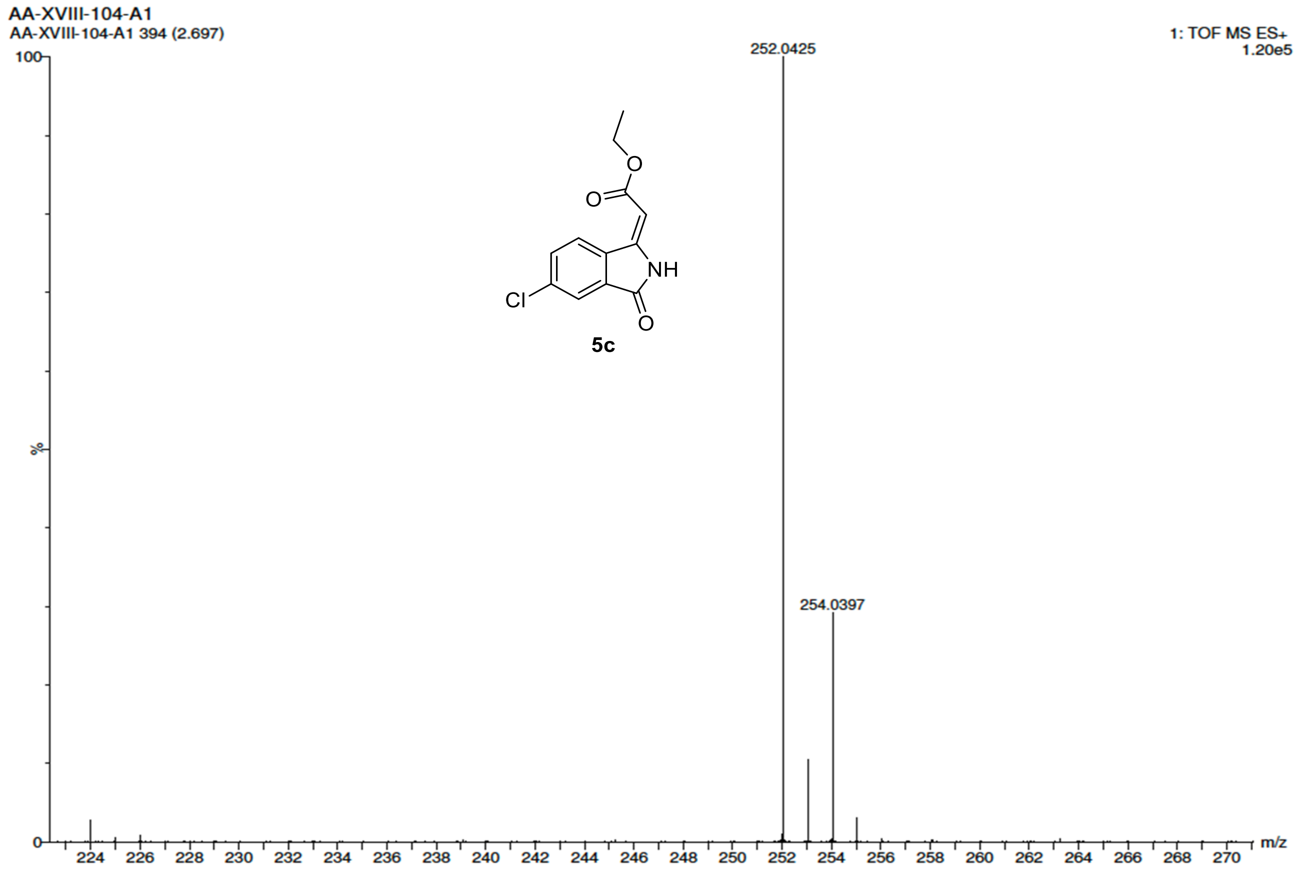

HRMS (ESI) *m/z* calcd for $C_{12}H_{10}ClNO_3$ $[M + H]^+$ 252.0422; found 252.0425.

Ethyl (*E*)-2-(5-bromo-3-oxoisoindolin-1-ylidene)acetate (5d)

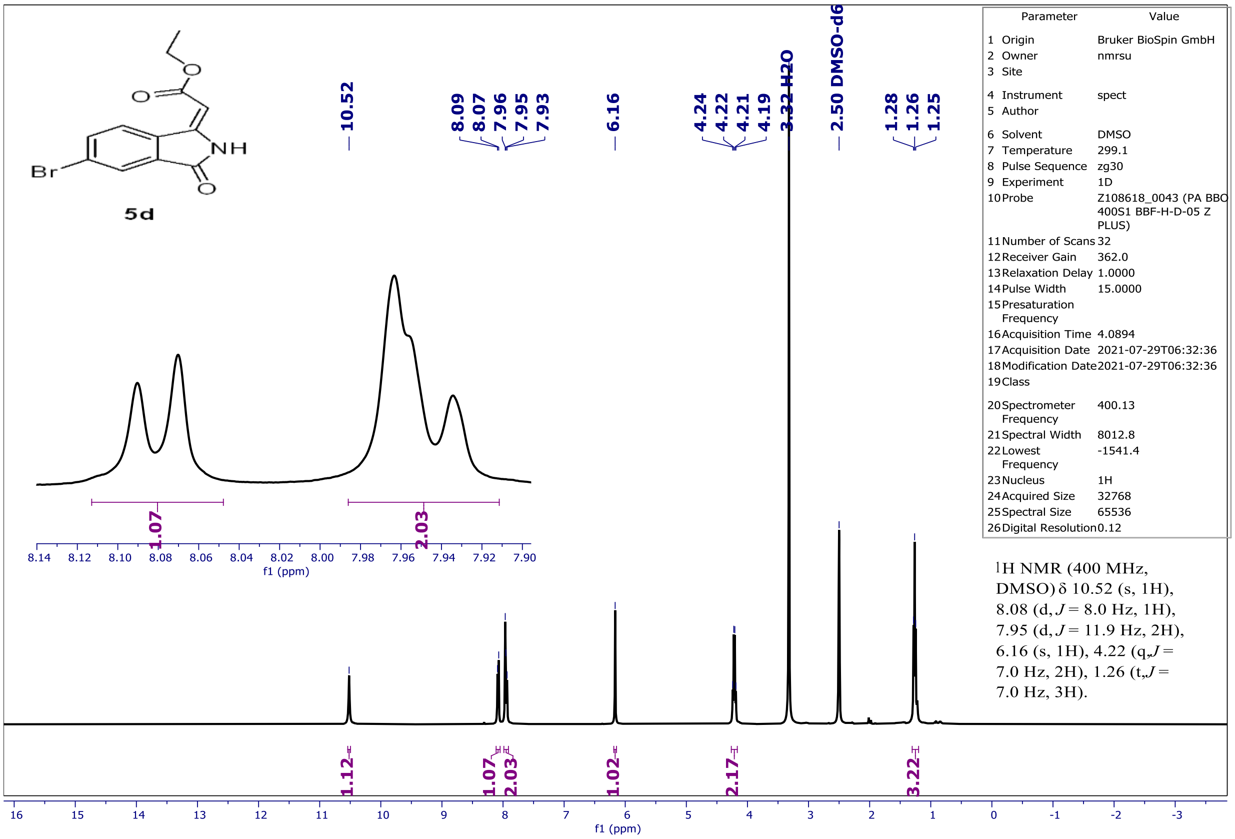

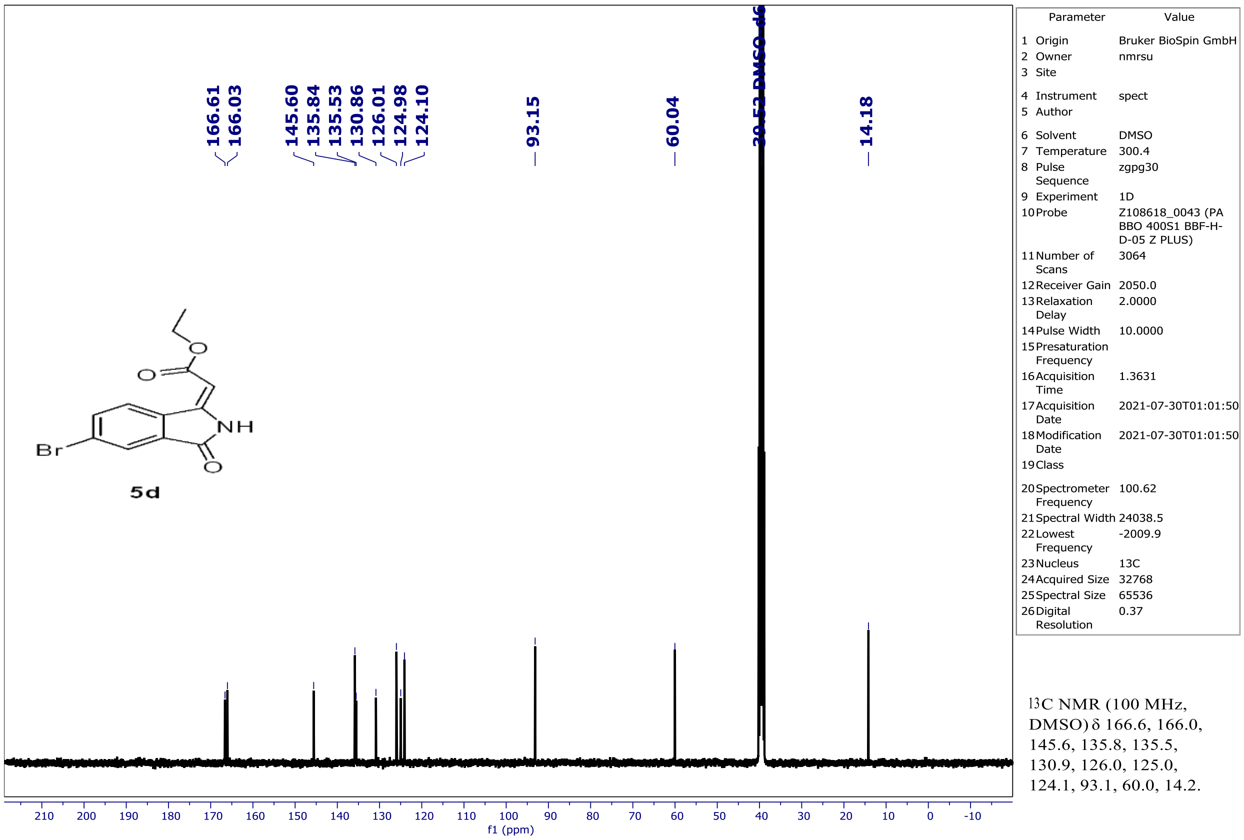

	Parameter	Value
1	Origin	Bruker BioSpin GmbH
2	Owner	nmrsu
3	Site	
4	Instrument	spect
5	Author	
6	Solvent	DMSO
7	Temperature	300.4
8	Pulse Sequence	zgpg30
9	Experiment	1D
10	Probe	Z108618_0043 (PA BBO 400S1 BBF-H-D-05 Z PLUS)
11	Number of Scans	3064
12	Receiver Gain	2050.0
13	Relaxation Delay	2.0000
14	Pulse Width	10.0000
15	Presaturation Frequency	
16	Acquisition Time	1.3631
17	Acquisition Date	2021-07-30T01:01:50
18	Modification Date	2021-07-30T01:01:50
19	Class	
20	Spectrometer Frequency	100.62
21	Spectral Width	24038.5
22	Lowest Frequency	-2009.9
23	Nucleus	13C
24	Acquired Size	32768
25	Spectral Size	65536
26	Digital Resolution	0.37

^{13}C NMR (100 MHz, DMSO) δ 166.6, 166.0, 145.6, 135.8, 135.5, 130.9, 126.0, 125.0, 124.1, 93.1, 60.0, 14.2.

Ethyl (*E*)-2-(5-bromo-3-oxoisoindolin-1-ylidene)acetate (5d)

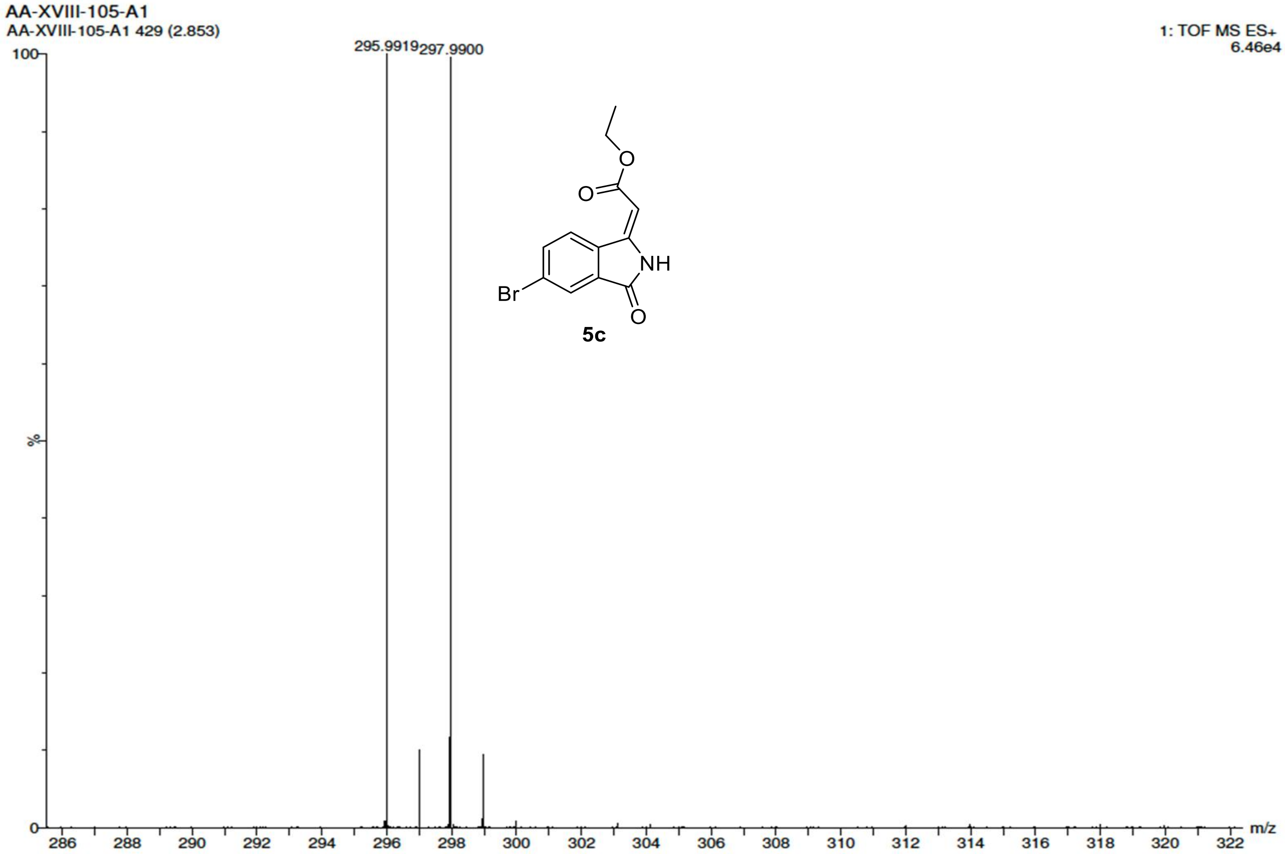

HRMS (ESI) *m/z* calcd for $C_{12}H_{10}BrNO_3$ $[M + H]^+$ 295.9917; found 295.9919.

Ethyl (*E*)-2-(5-methyl-3-oxoisoindolin-1-ylidene)acetate (5e)

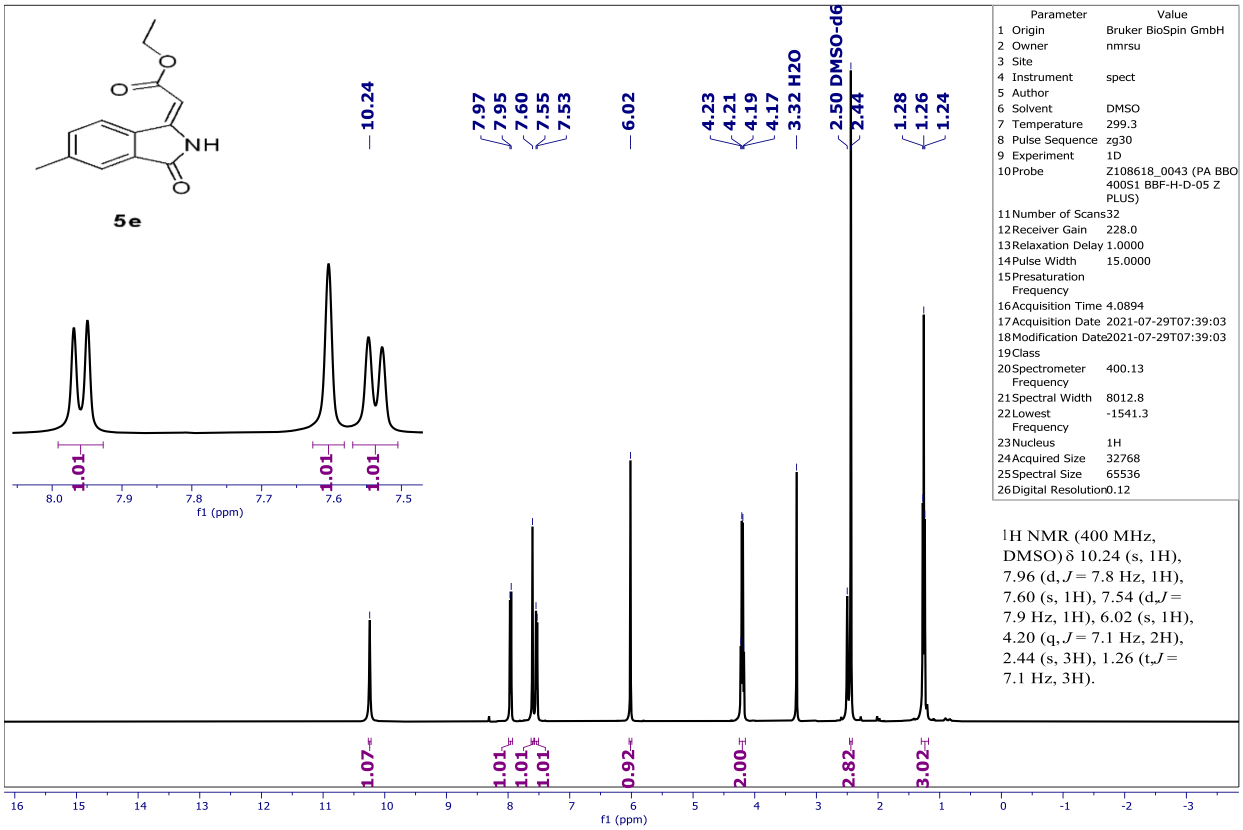

Parameter	Value
1 Origin	Bruker BioSpin GmbH
2 Owner	nmrsu
3 Site	
4 Instrument	spect
5 Author	
6 Solvent	DMSO
7 Temperature	299.3
8 Pulse Sequence	zg30
9 Experiment	1D
10 Probe	Z108618_0043 (PA BBO 400S1 BBF-H-D-05 Z PLUS)
11 Number of Scans	32
12 Receiver Gain	228.0
13 Relaxation Delay	1.0000
14 Pulse Width	15.0000
15 Presaturation Frequency	
16 Acquisition Time	4.0894
17 Acquisition Date	2021-07-29T07:39:03
18 Modification Date	2021-07-29T07:39:03
19 Class	
20 Spectrometer Frequency	400.13
21 Spectral Width	8012.8
22 Lowest Frequency	-1541.3
23 Nucleus	1H
24 Acquired Size	32768
25 Spectral Size	65536
26 Digital Resolution	0.12

^{1}H NMR (400 MHz, DMSO) δ 10.24 (s, 1H), 7.96 (d, *J* = 7.8 Hz, 1H), 7.60 (s, 1H), 7.54 (d, *J* = 7.9 Hz, 1H), 6.02 (s, 1H), 4.20 (q, *J* = 7.1 Hz, 2H), 2.44 (s, 3H), 1.26 (t, *J* = 7.1 Hz, 3H).

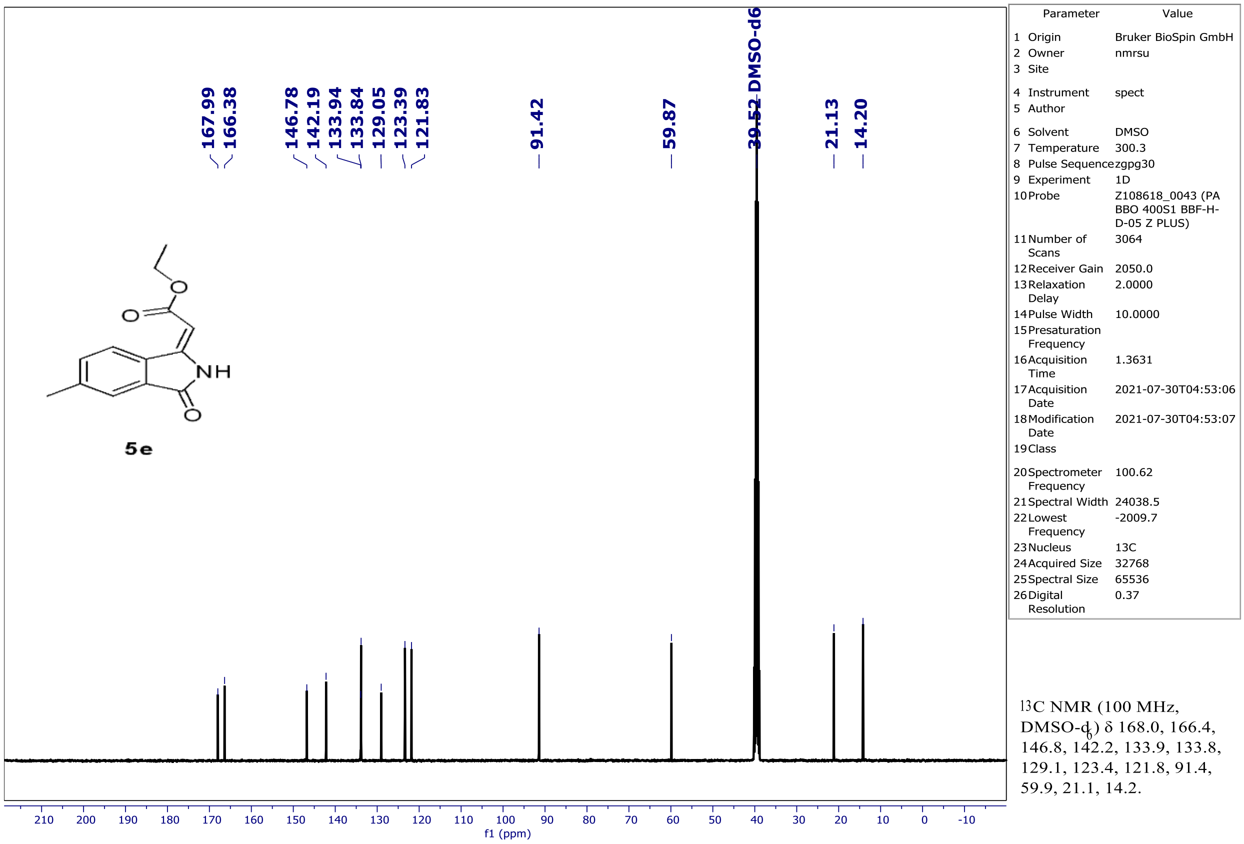

Parameter	Value
1 Origin	Bruker BioSpin GmbH
2 Owner	nmrsu
3 Site	
4 Instrument	spect
5 Author	
6 Solvent	DMSO
7 Temperature	300.3
8 Pulse Sequence	zgpg30
9 Experiment	1D
10 Probe	Z108618_0043 (PA BBO 400S1 BBF-H-D-05 Z PLUS)
11 Number of Scans	3064
12 Receiver Gain	2050.0
13 Relaxation Delay	2.0000
14 Pulse Width	10.0000
15 Presaturation Frequency	
16 Acquisition Time	1.3631
17 Acquisition Date	2021-07-30T04:53:06
18 Modification Date	2021-07-30T04:53:07
19 Class	
20 Spectrometer Frequency	100.62
21 Spectral Width	24038.5
22 Lowest Frequency	-2009.7
23 Nucleus	13C
24 Acquired Size	32768
25 Spectral Size	65536
26 Digital Resolution	0.37

^{13}C NMR (100 MHz, DMSO-d_6) δ 168.0, 166.4, 146.8, 142.2, 133.9, 133.8, 129.1, 123.4, 121.8, 91.4, 59.9, 21.1, 14.2.

Ethyl (*E*)-2-(5-methyl-3-oxoisoindolin-1-ylidene)acetate (5e)

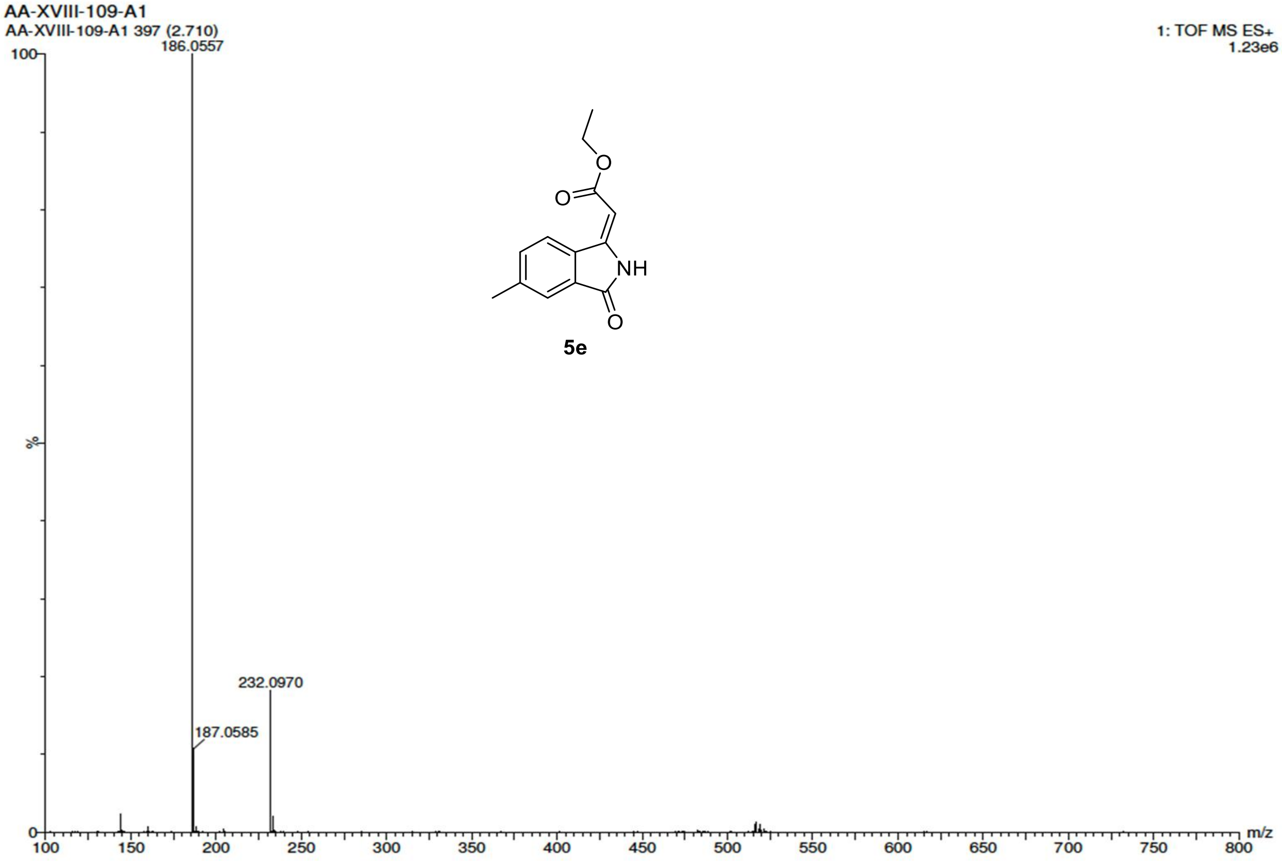

HRMS (ESI) *m/z* calcd for $C_{13}H_{13}NO_3$ $[M + H]^+$ Exact Mass: 232.0968; found 232.0970.

Ethyl (*E*)-2-(5-methoxy-3-oxoisoindolin-1-ylidene)acetate (5f)

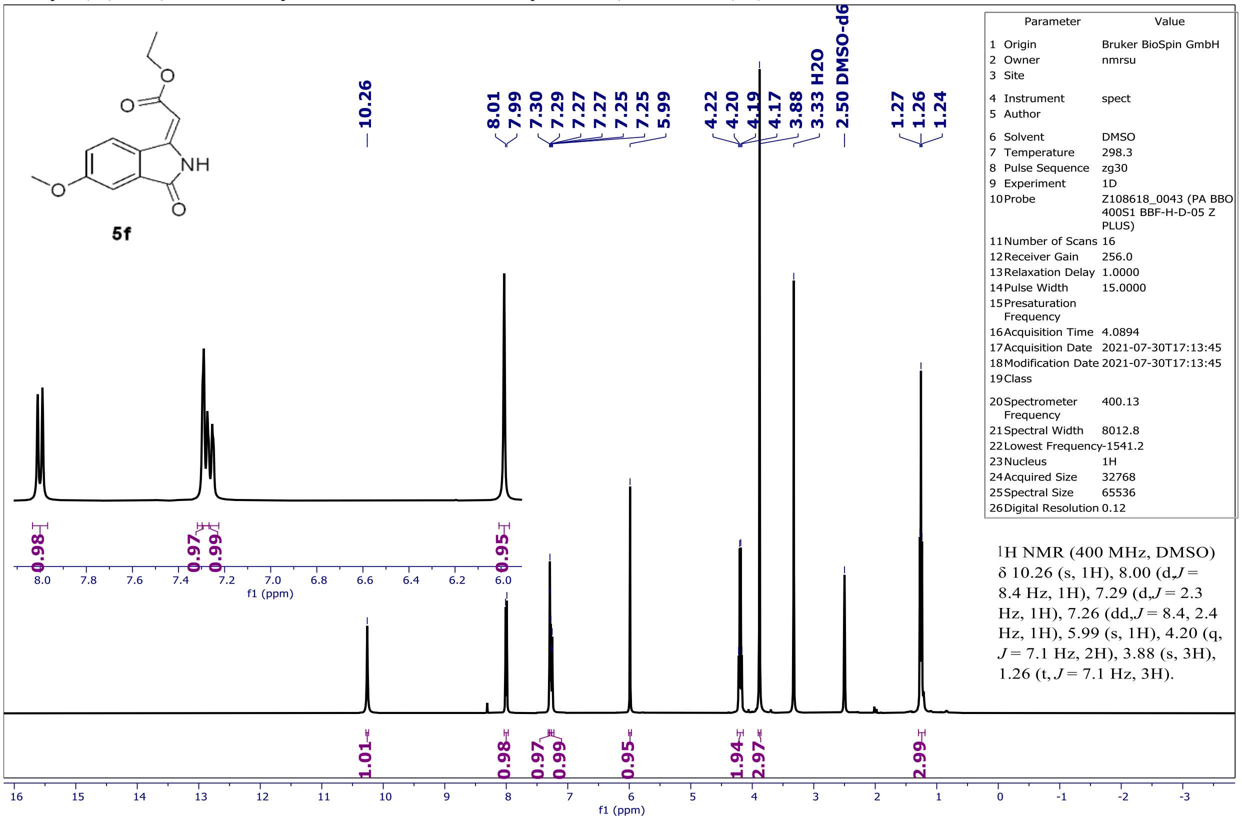

^{1}H NMR (400 MHz, DMSO) δ 10.26 (s, 1H), 8.00 (d, *J* = 8.4 Hz, 1H), 7.29 (d, *J* = 2.3 Hz, 1H), 7.26 (dd, *J* = 8.4, 2.4 Hz, 1H), 5.99 (s, 1H), 4.20 (q, *J* = 7.1 Hz, 2H), 3.88 (s, 3H), 1.26 (t, *J* = 7.1 Hz, 3H).

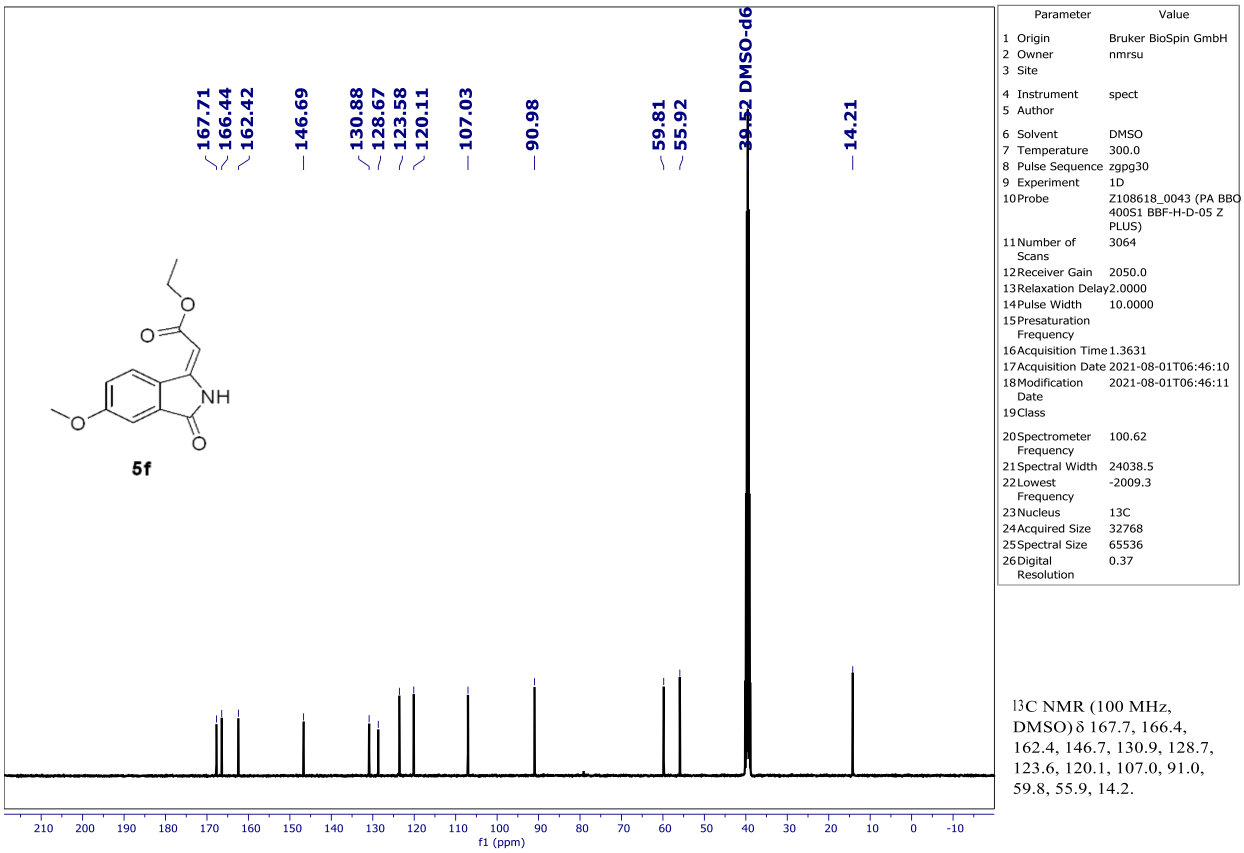

	Parameter	Value
1	Origin	Bruker BioSpin GmbH
2	Owner	nmrsu
3	Site	
4	Instrument	spect
5	Author	
6	Solvent	DMSO
7	Temperature	300.0
8	Pulse Sequence	zgpg30
9	Experiment	1D
10	Probe	Z108618_0043 (PA BBO 400S1 BBF-H-D-05 Z PLUS)
11	Number of Scans	3064
12	Receiver Gain	2050.0
13	Relaxation Delay	2.0000
14	Pulse Width	10.0000
15	Presaturation Frequency	
16	Acquisition Time	1.3631
17	Acquisition Date	2021-08-01T06:46:10
18	Modification Date	2021-08-01T06:46:11
19	Class	
20	Spectrometer Frequency	100.62
21	Spectral Width	24038.5
22	Lowest Frequency	-2009.3
23	Nucleus	13C
24	Acquired Size	32768
25	Spectral Size	65536
26	Digital Resolution	0.37

^{13}C NMR (100 MHz, DMSO) δ 167.7, 166.4, 162.4, 146.7, 130.9, 128.7, 123.6, 120.1, 107.0, 91.0, 59.8, 55.9, 14.2.

Ethyl (*E*)-2-(5-methoxy-3-oxoisoindolin-1-ylidene)acetate (5f)

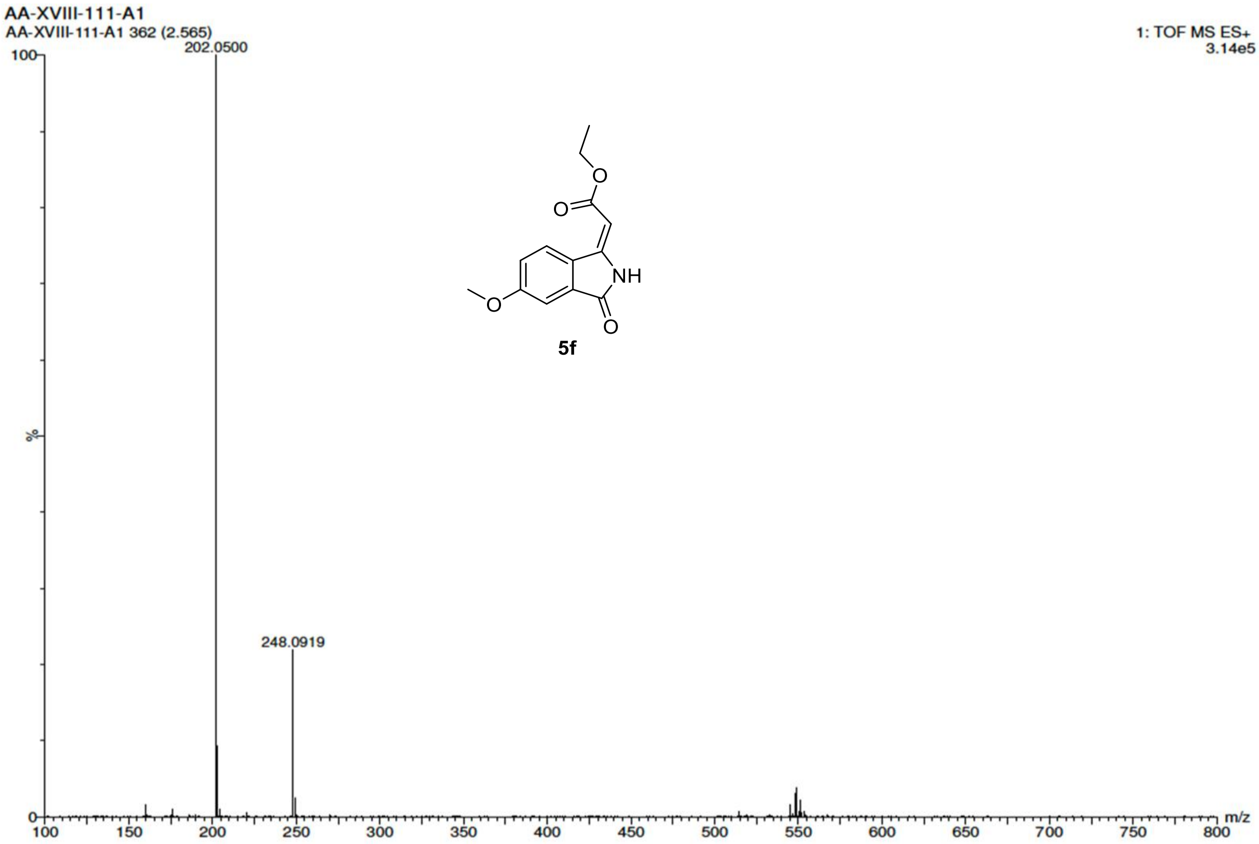

HRMS (ESI) *m/z* calcd for $C_{13}H_{13}NO_4$ $[M + H]^+$ Exact Mass: 248.0917; found 248.0919.

Ethyl (*E*)-2-(5-nitro-3-oxoisoindolin-1-ylidene)acetate (5g)

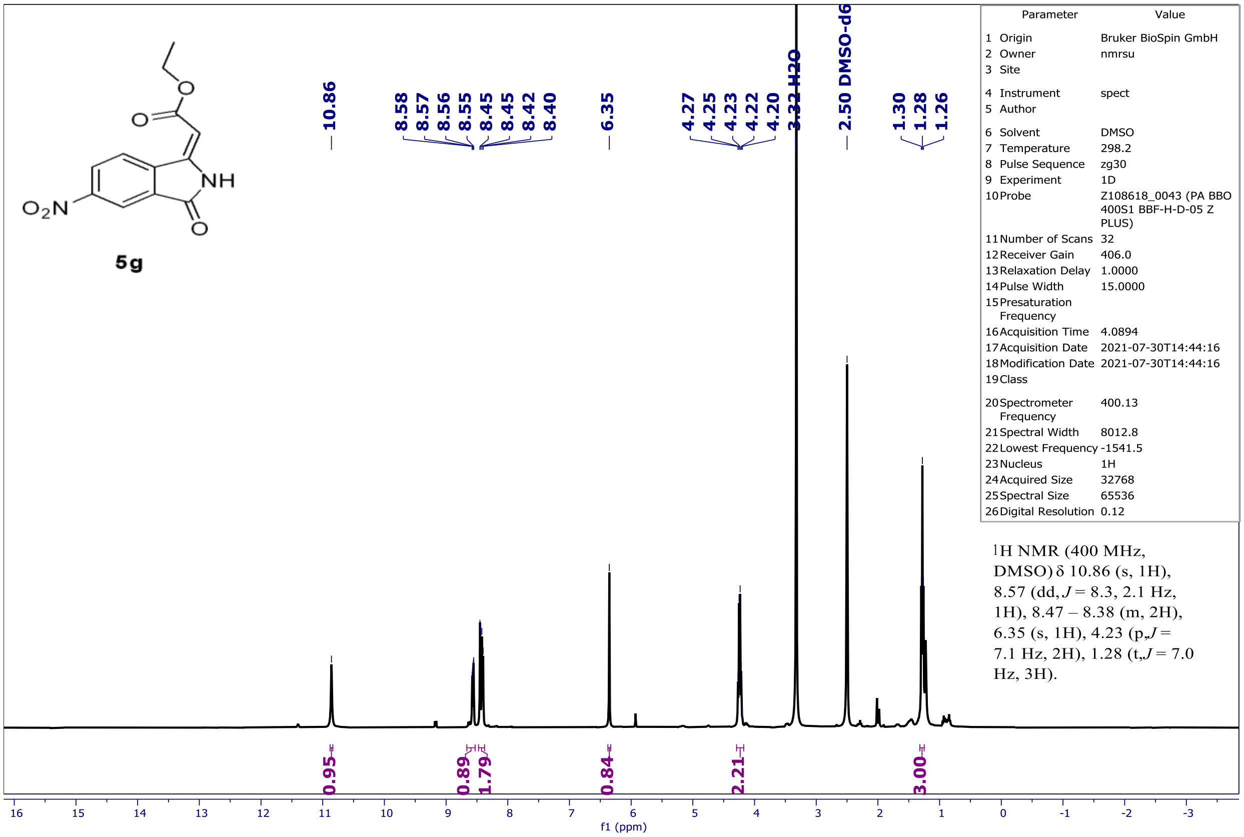

1H NMR (400 MHz, DMSO) δ 10.86 (s, 1H), 8.57 (dd, J = 8.3, 2.1 Hz, 1H), 8.47 – 8.38 (m, 2H), 6.35 (s, 1H), 4.23 (p, J = 7.1 Hz, 2H), 1.28 (t, J = 7.0 Hz, 3H).

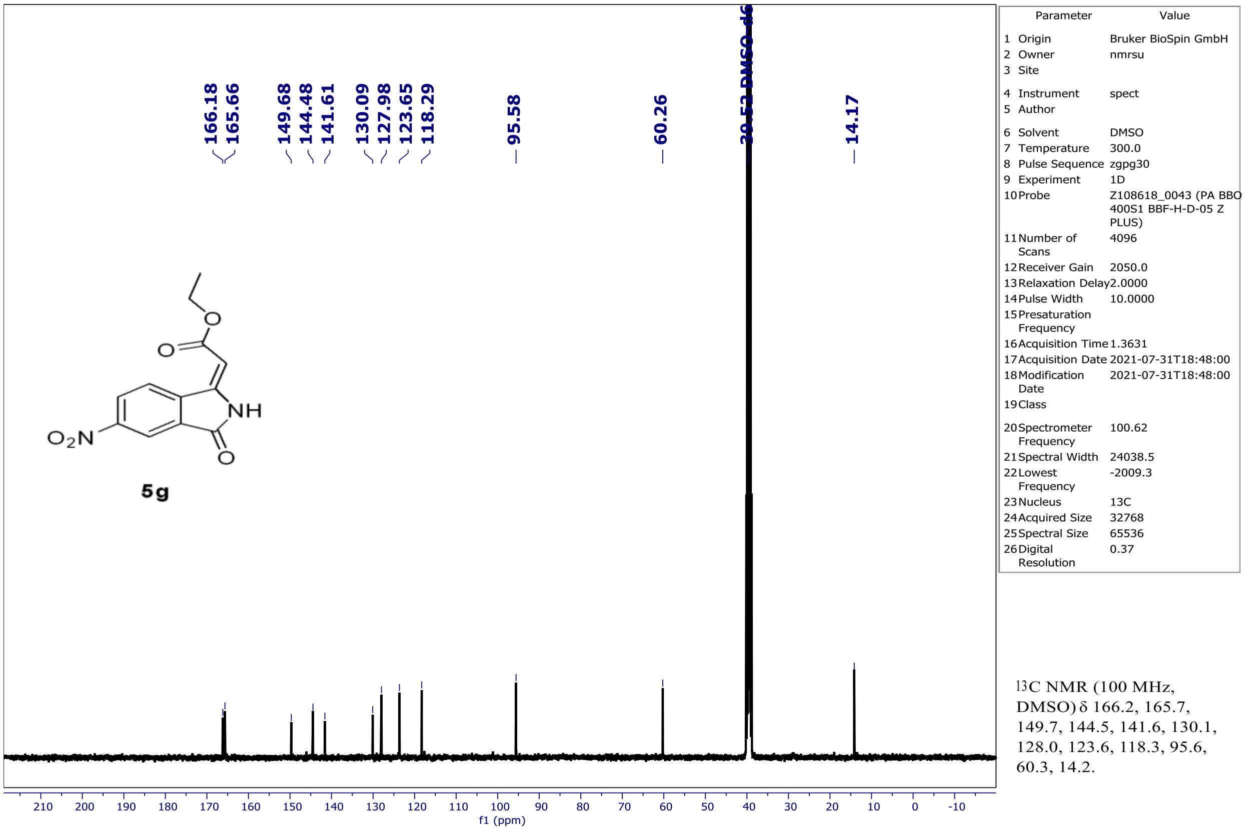

13C NMR (100 MHz, DMSO) δ 166.2, 165.7, 149.7, 144.5, 141.6, 130.1, 128.0, 123.6, 118.3, 95.6, 60.3, 14.2.

Ethyl (*E*)-2-(5-nitro-3-oxoisoindolin-1-ylidene)acetate (5g)

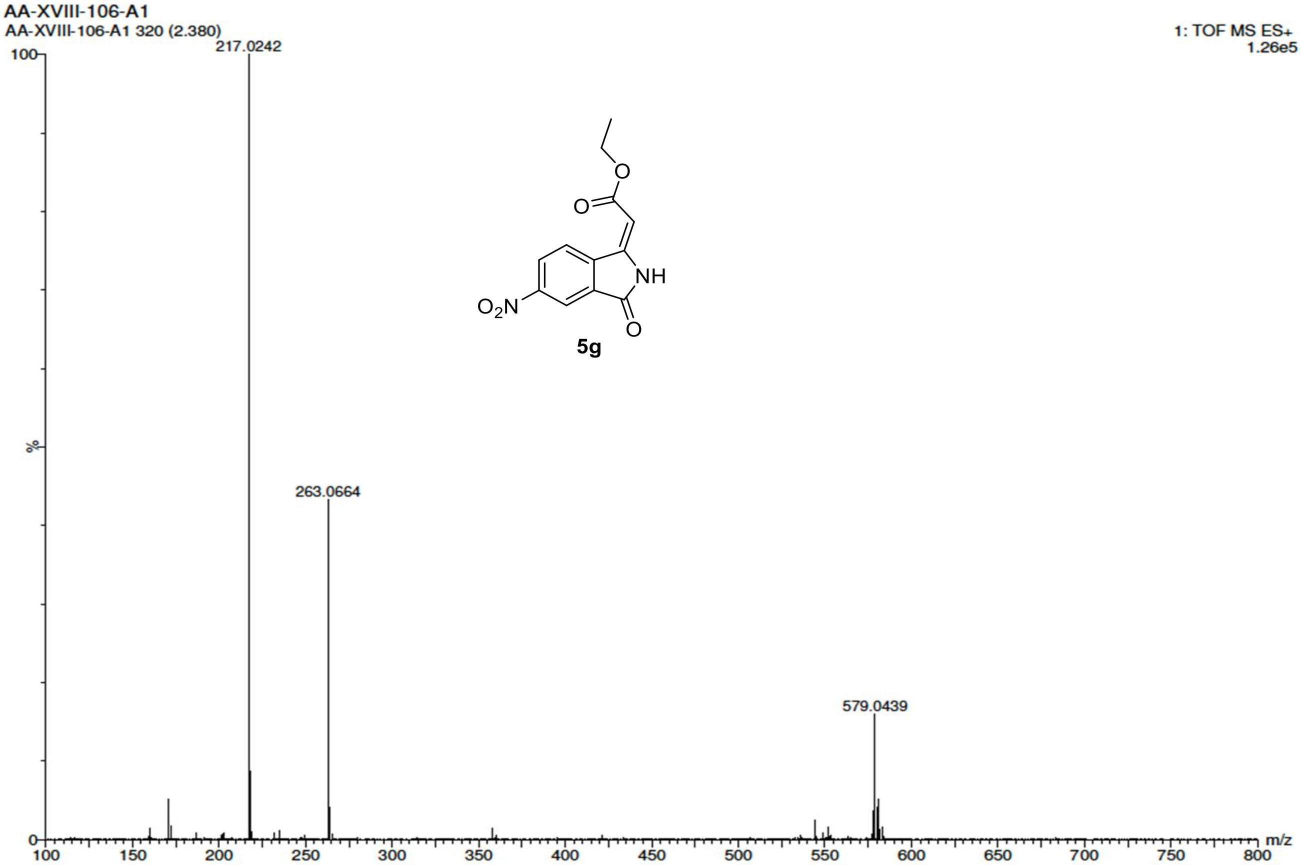

HRMS (ESI) *m/z* calcd for $C_{12}H_{10}N_2O_5$ $[M + H]^+$ 263.0662; found 263.0664.

Ethyl (*E*)-2-(6-chloro-3-oxoisoindolin-1-ylidene)acetate (5h)

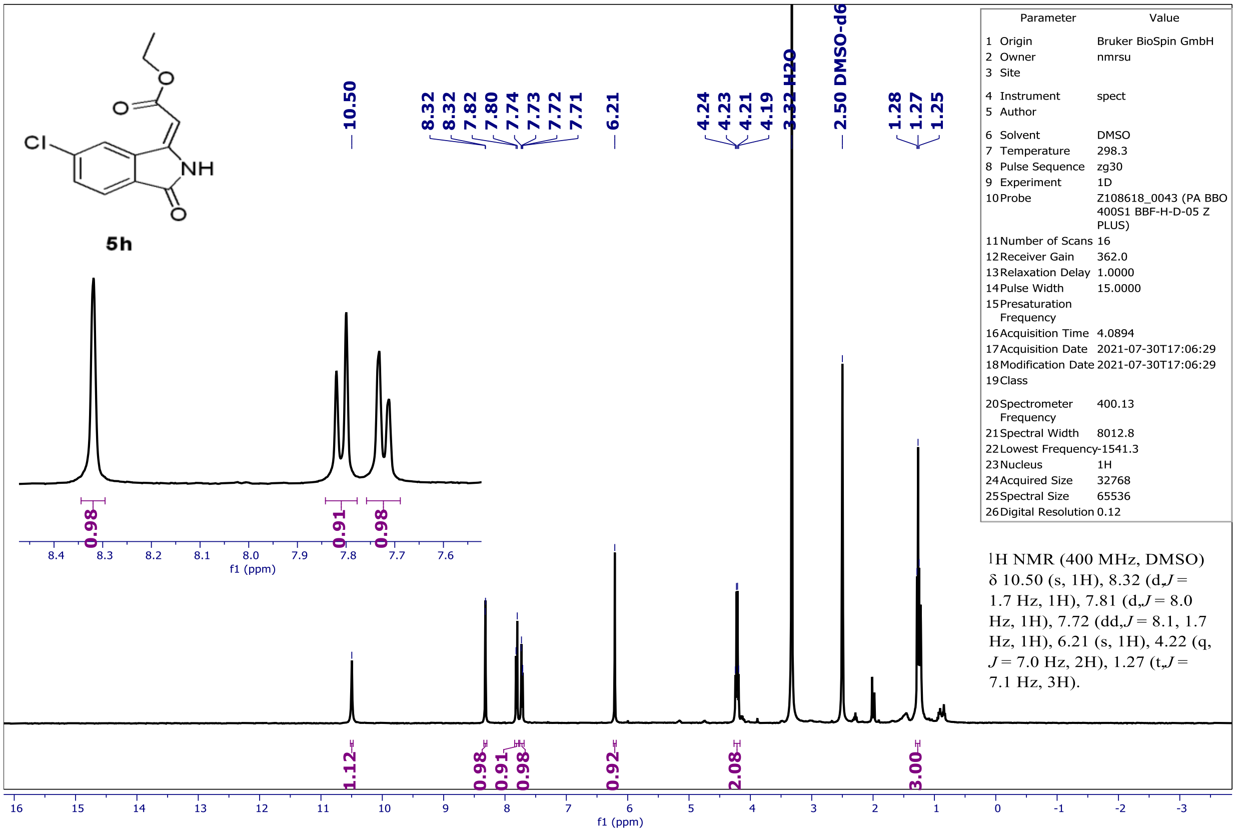

^{1}H NMR (400 MHz, DMSO) δ 10.50 (s, 1H), 8.32 (d, *J* = 1.7 Hz, 1H), 7.81 (d, *J* = 8.0 Hz, 1H), 7.72 (dd, *J* = 8.1, 1.7 Hz, 1H), 6.21 (s, 1H), 4.22 (q, *J* = 7.0 Hz, 2H), 1.27 (t, *J* = 7.1 Hz, 3H).

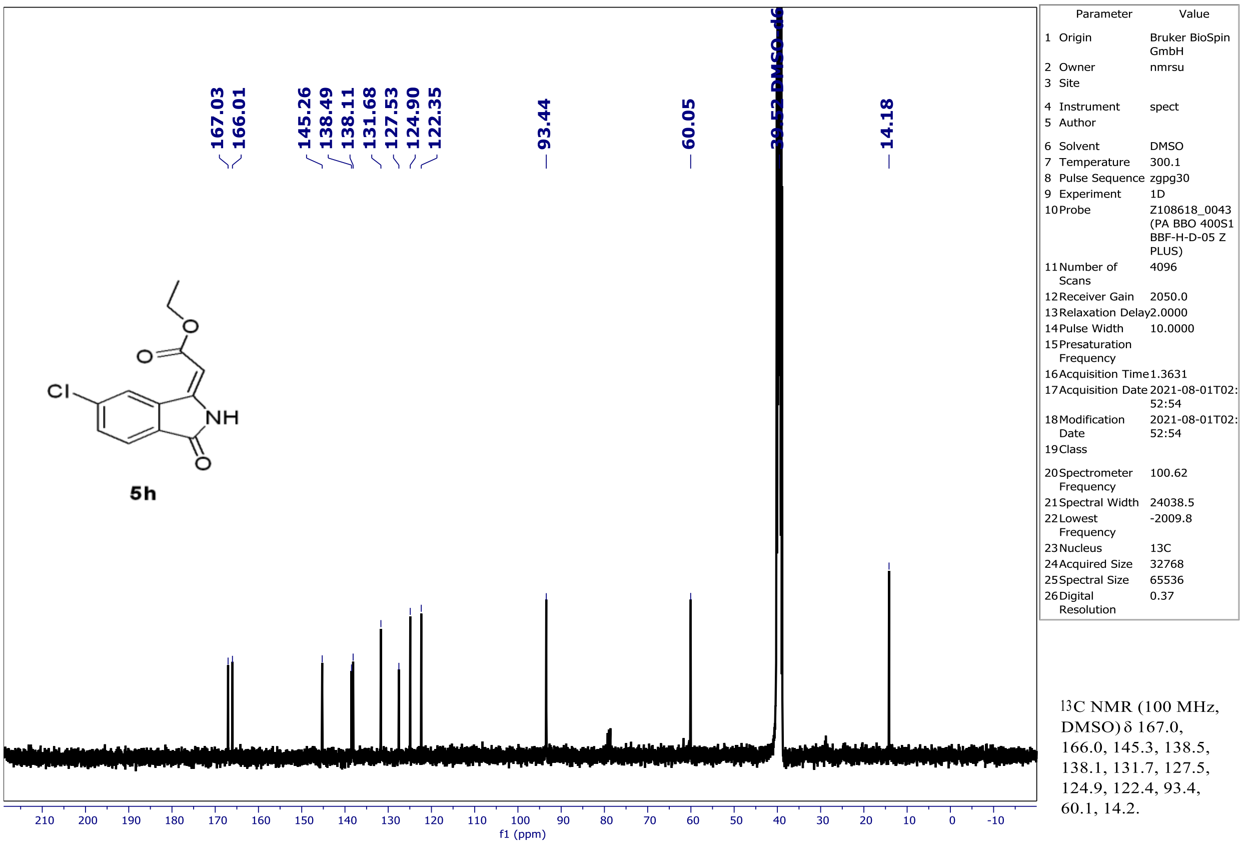

^{13}C NMR (100 MHz, DMSO) δ 167.0, 166.0, 145.3, 138.5, 138.1, 131.7, 127.5, 124.9, 122.4, 93.4, 60.1, 14.2.

Ethyl (*E*)-2-(6-chloro-3-oxoisoindolin-1-ylidene)acetate (5h)

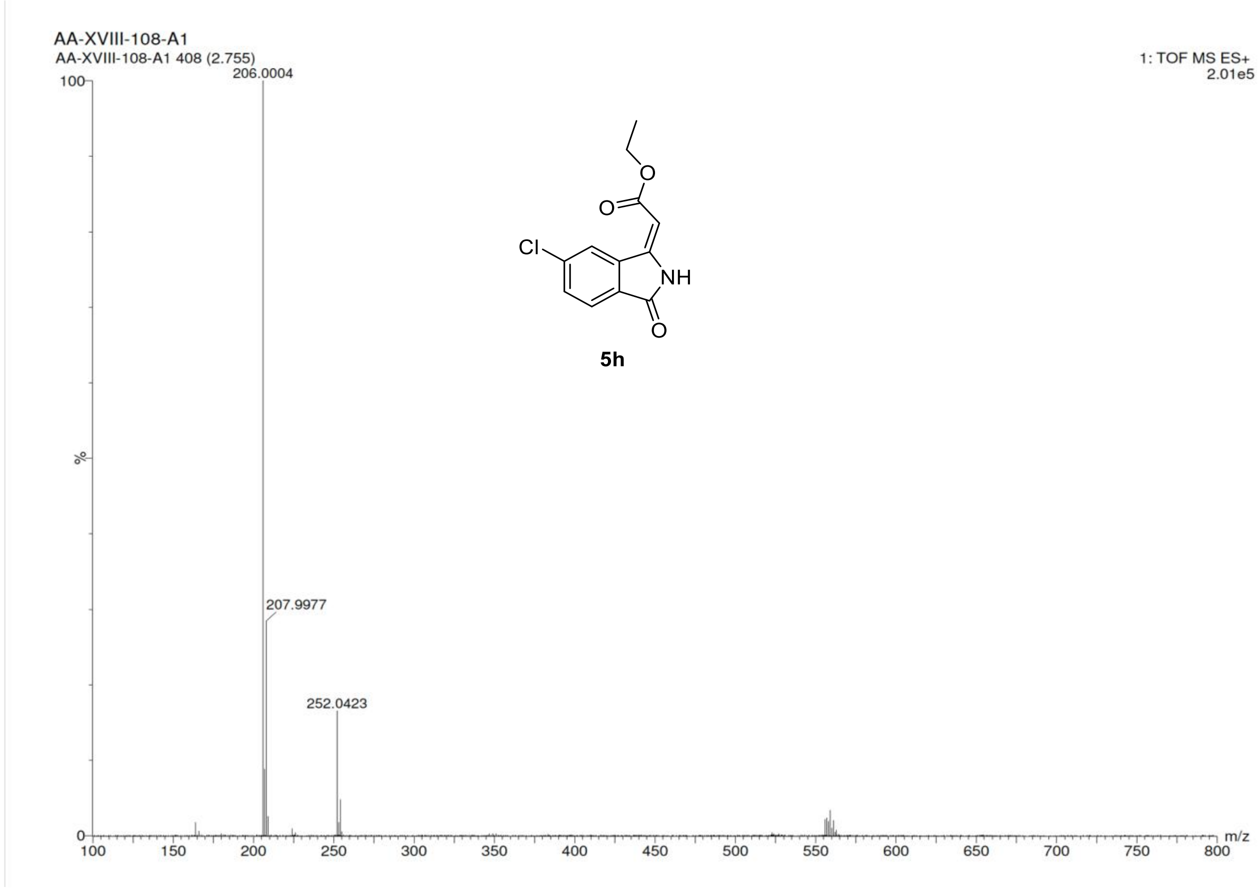

HRMS (ESI) *m/z* calcd for $C_{12}H_{10}ClNO_3$ $[M + H]^+$ 252.0422; found 252.0423.

Ethyl (*E*)-2-(6-nitro-3-oxoisoindolin-1-ylidene)acetate (5i)

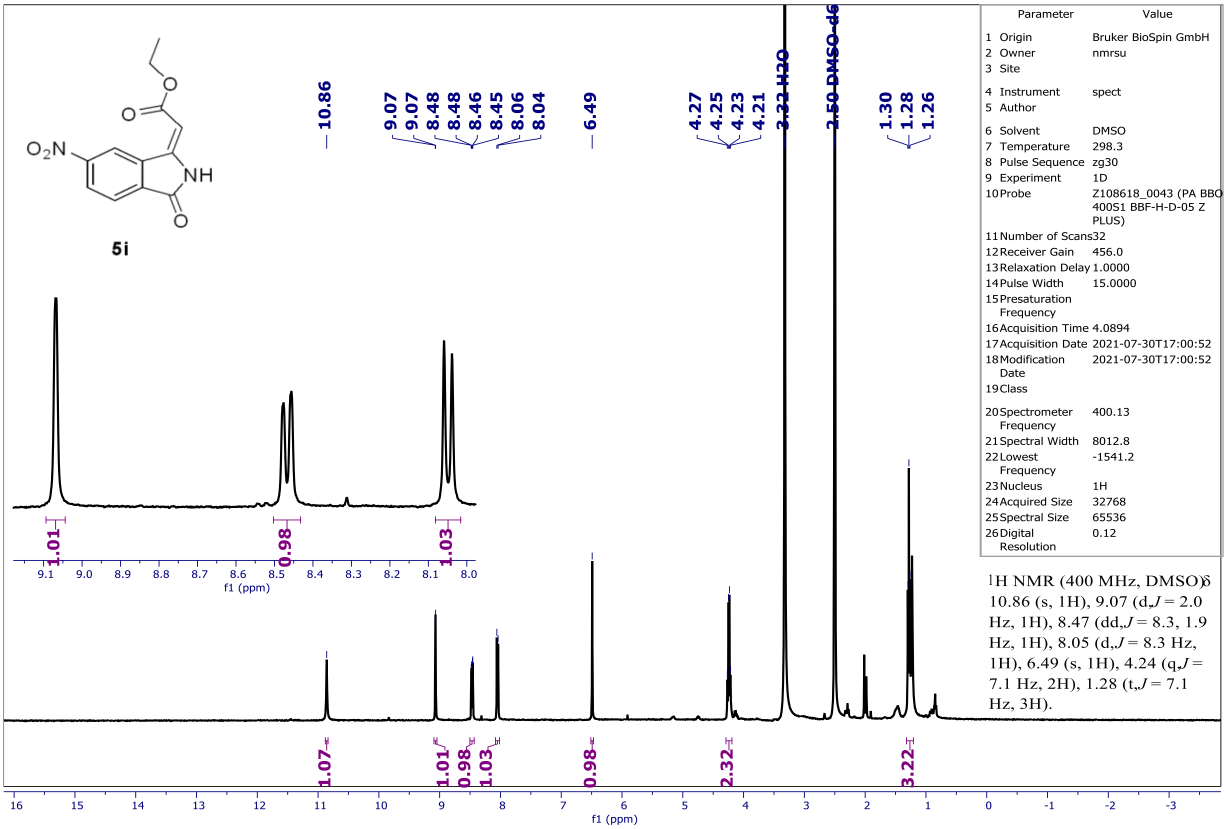

Parameter		Value
1	Origin	Bruker BioSpin GmbH
2	Owner	nmrsu
3	Site	
4	Instrument	spect
5	Author	
6	Solvent	DMSO
7	Temperature	298.3
8	Pulse Sequence	zg30
9	Experiment	1D
10	Probe	Z108618_0043 (PA BBO 400S1 BBF-H-D-05 Z PLUS)
11	Number of Scans	32
12	Receiver Gain	456.0
13	Relaxation Delay	1.0000
14	Pulse Width	15.0000
15	Presaturation Frequency	
16	Acquisition Time	4.0894
17	Acquisition Date	2021-07-30T17:00:52
18	Modification Date	2021-07-30T17:00:52
19	Class	
20	Spectrometer Frequency	400.13
21	Spectral Width	8012.8
22	Lowest Frequency	-1541.2
23	Nucleus	1H
24	Acquired Size	32768
25	Spectral Size	65536
26	Digital Resolution	0.12

^{1}H NMR (400 MHz, DMSO) δ 10.86 (s, 1H), 9.07 (d, *J* = 2.0 Hz, 1H), 8.47 (dd, *J* = 8.3, 1.9 Hz, 1H), 8.05 (d, *J* = 8.3 Hz, 1H), 6.49 (s, 1H), 4.24 (q, *J* = 7.1 Hz, 2H), 1.28 (t, *J* = 7.1 Hz, 3H).

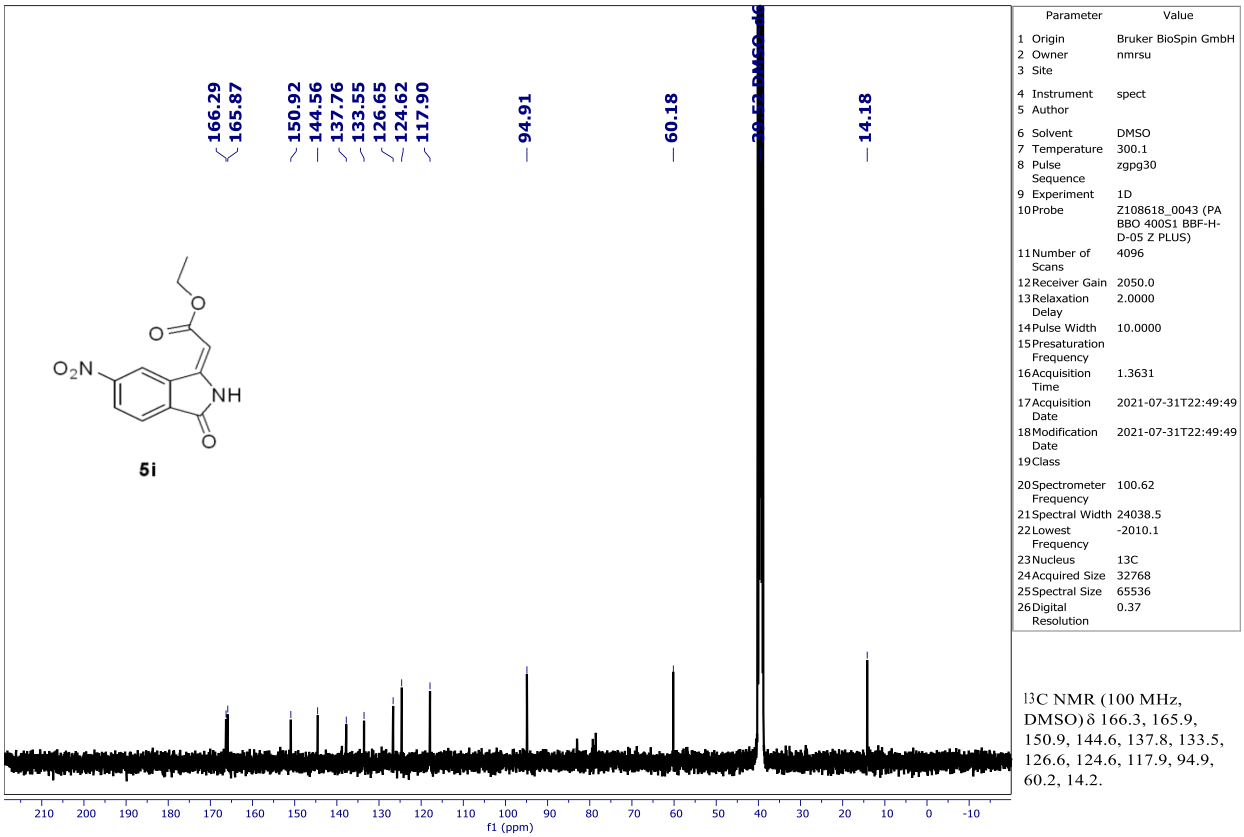

Parameter		Value
1	Origin	Bruker BioSpin GmbH
2	Owner	nmrsu
3	Site	
4	Instrument	spect
5	Author	
6	Solvent	DMSO
7	Temperature	300.1
8	Pulse Sequence	zgpg30
9	Experiment	1D
10	Probe	Z108618_0043 (PA BBO 400S1 BBF-H-D-05 Z PLUS)
11	Number of Scans	4096
12	Receiver Gain	2050.0
13	Relaxation Delay	2.0000
14	Pulse Width	10.0000
15	Presaturation Frequency	
16	Acquisition Time	1.3631
17	Acquisition Date	2021-07-31T22:49:49
18	Modification Date	2021-07-31T22:49:49
19	Class	
20	Spectrometer Frequency	100.62
21	Spectral Width	24038.5
22	Lowest Frequency	-2010.1
23	Nucleus	13C
24	Acquired Size	32768
25	Spectral Size	65536
26	Digital Resolution	0.37

^{13}C NMR (100 MHz, DMSO) δ 166.3, 165.9, 150.9, 144.6, 137.8, 133.5, 126.6, 124.6, 117.9, 94.9, 60.2, 14.2.

Ethyl (*E*)-2-(6-nitro-3-oxoisoindolin-1-ylidene)acetate (5i)

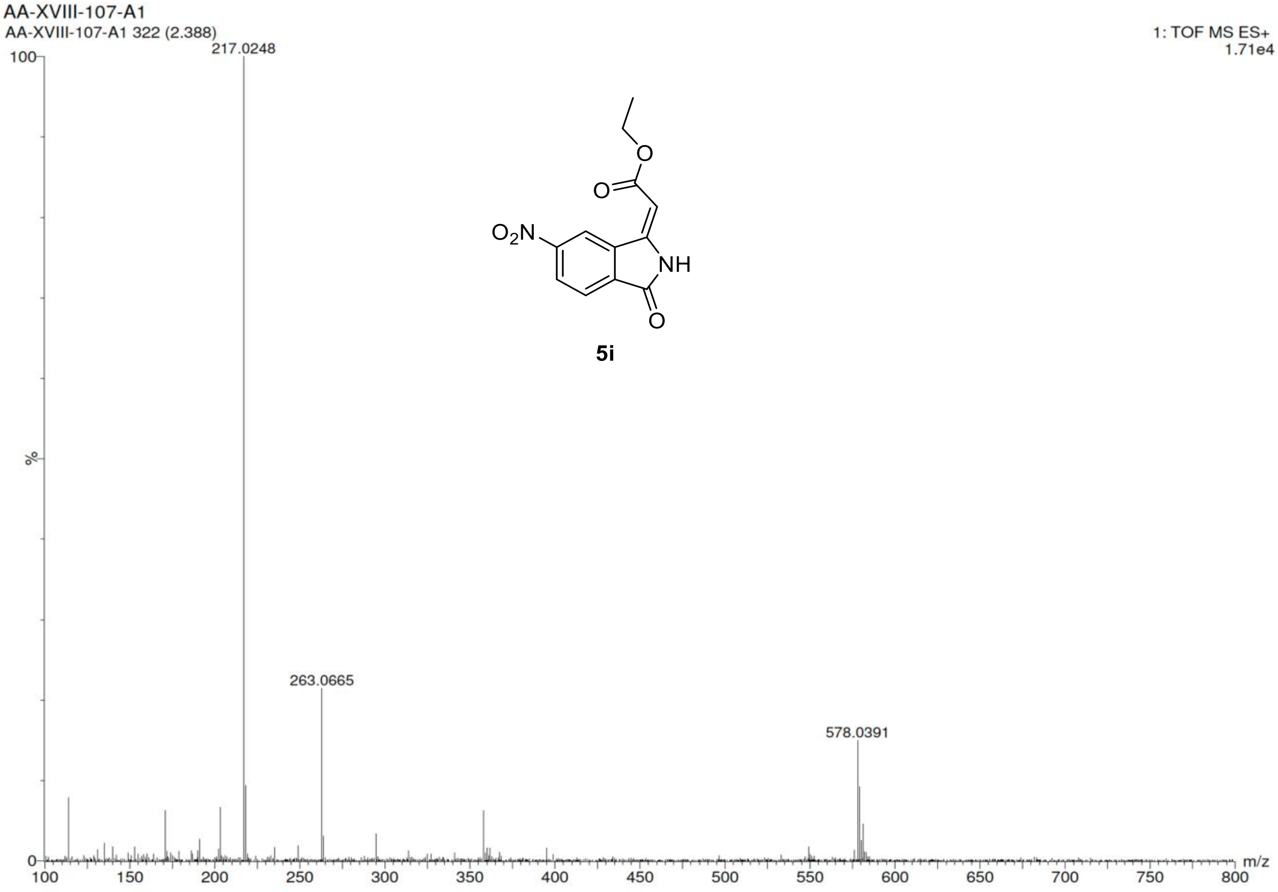

HRMS (ESI) *m/z* calcd for $C_{12}H_{10}N_2O_5$ $[M + H]^+$ 263.0662; found 263.0665.

N-(Methylsulfonyl)-2-((triisopropylsilyl)ethynyl)benzamide (6a)

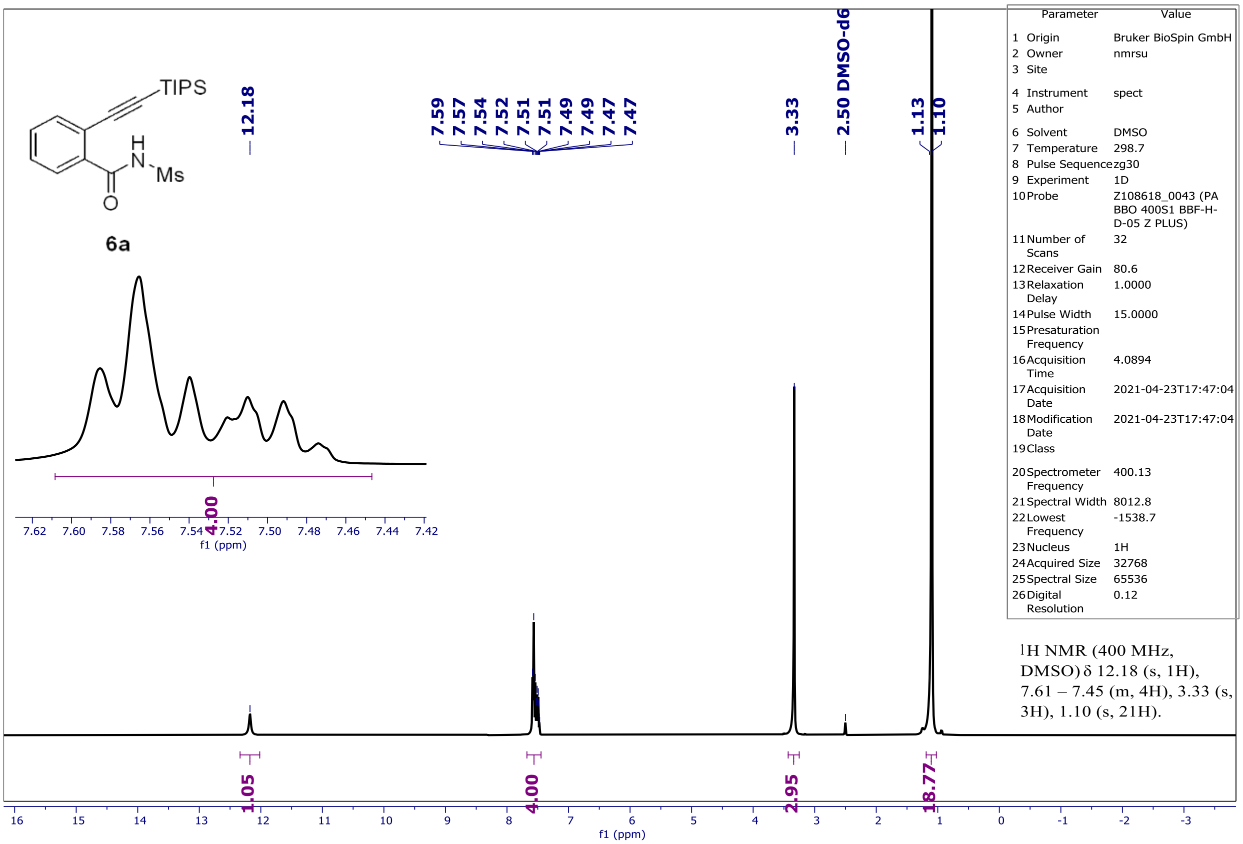

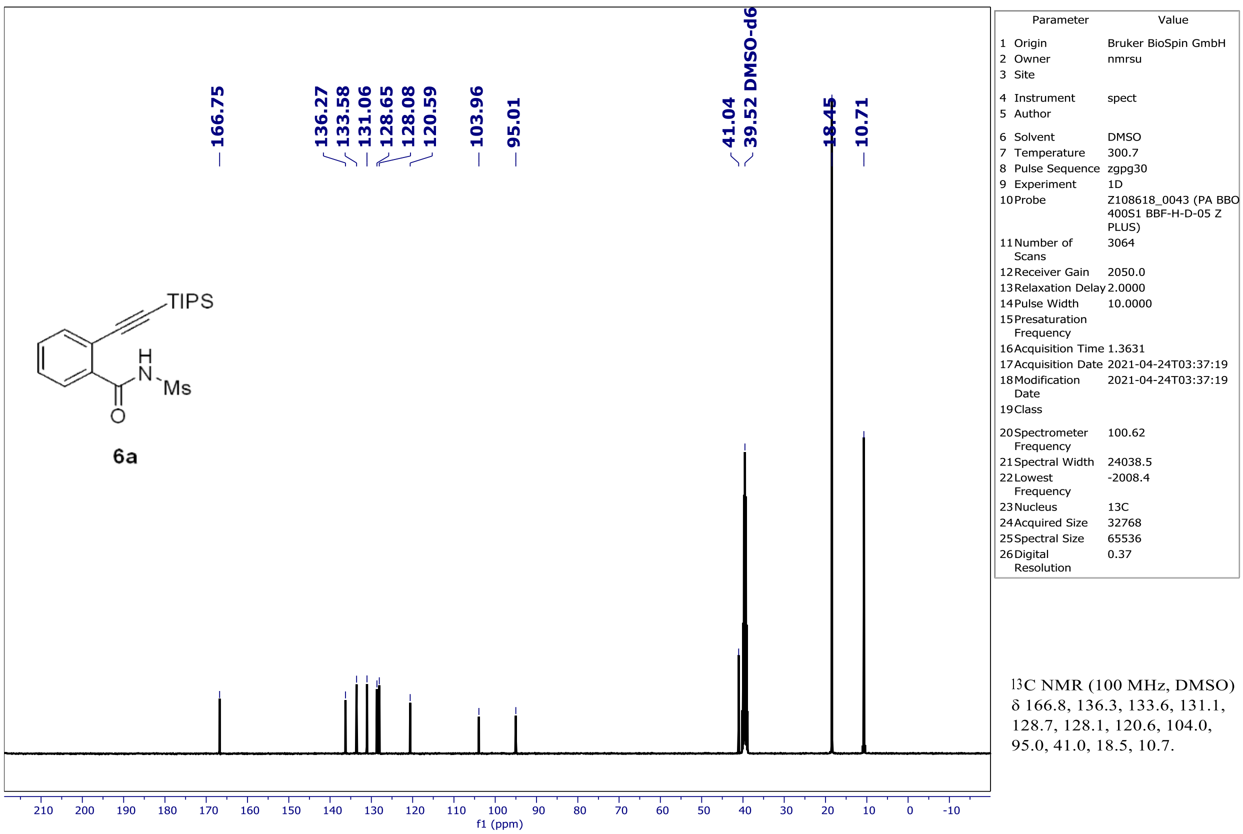

	Parameter	Value
1	Origin	Bruker BioSpin GmbH
2	Owner	nmrsu
3	Site	
4	Instrument	spect
5	Author	
6	Solvent	DMSO
7	Temperature	300.7
8	Pulse Sequence	zgpg30
9	Experiment	1D
10	Probe	Z108618_0043 (PA BBO 400S1 BBF-H-D-05 Z PLUS)
11	Number of Scans	3064
12	Receiver Gain	2050.0
13	Relaxation Delay	2.0000
14	Pulse Width	10.0000
15	Presaturation Frequency	
16	Acquisition Time	1.3631
17	Acquisition Date	2021-04-24T03:37:19
18	Modification Date	2021-04-24T03:37:19
19	Class	
20	Spectrometer Frequency	100.62
21	Spectral Width	24038.5
22	Lowest Frequency	-2008.4
23	Nucleus	13C
24	Acquired Size	32768
25	Spectral Size	65536
26	Digital Resolution	0.37

^{13}C NMR (100 MHz, DMSO) δ 166.8, 136.3, 133.6, 131.1, 128.7, 128.1, 120.6, 104.0, 95.0, 41.0, 18.5, 10.7.

***N*-(Methylsulfonyl)-2-((triisopropylsilyl)ethynyl)benzamide (6a)**

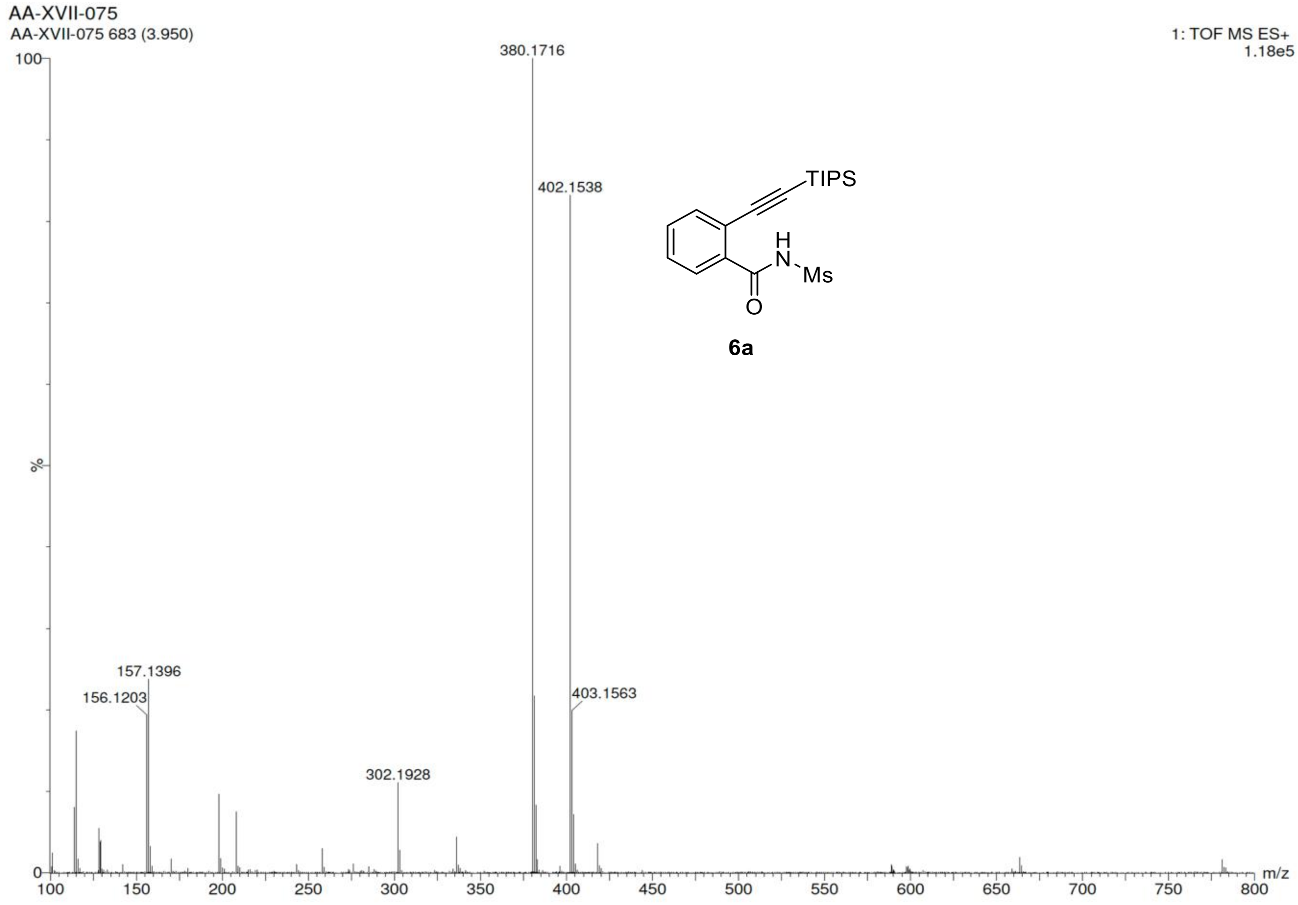

HRMS (ESI) *m/z* calcd for $C_{19}H_{29}NO_3SSi$ $[M + H]^+$ 380.1710; found 380.1716.

5-Fluoro-*N*-(methylsulfonyl)-2-((triisopropylsilyl)ethynyl)benzamide (6b)

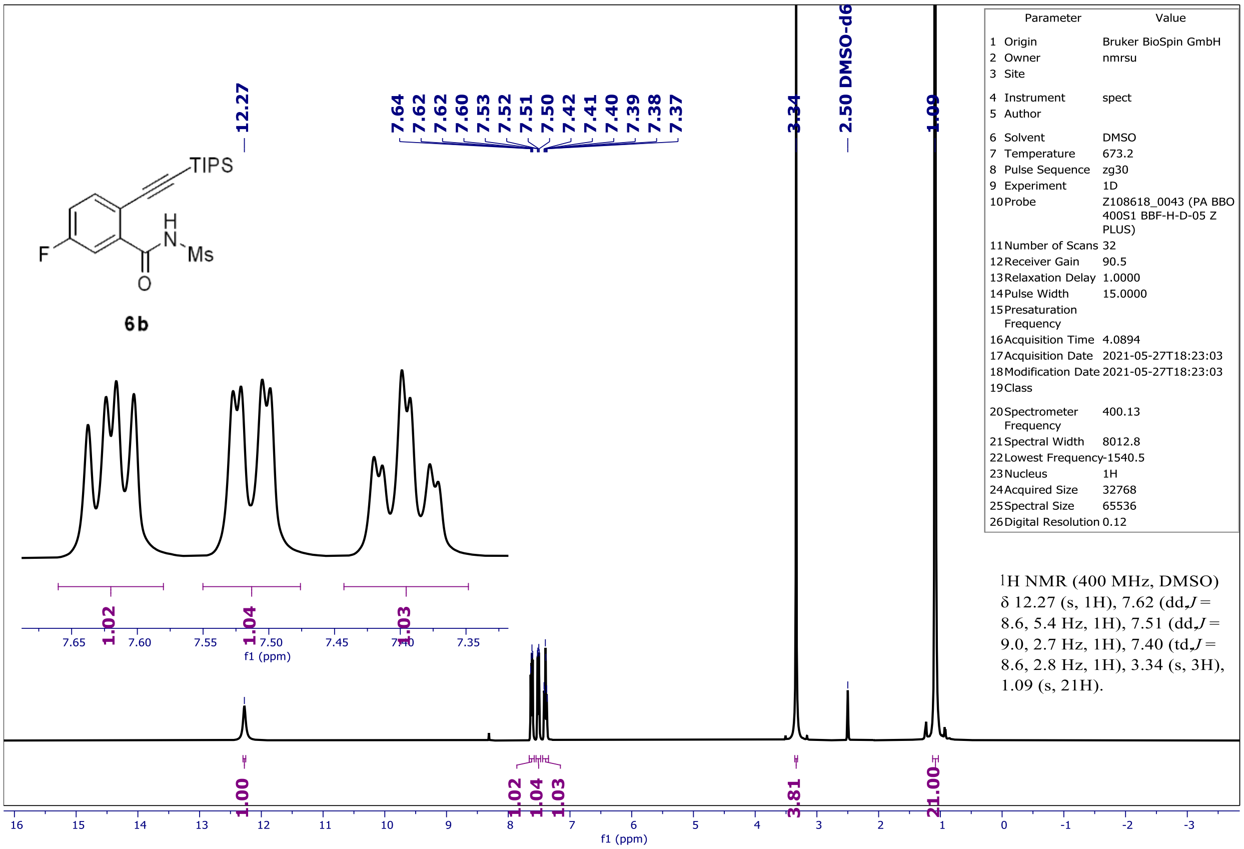

^{1}H NMR (400 MHz, DMSO) δ 12.27 (s, 1H), 7.62 (dd, *J* = 8.6, 5.4 Hz, 1H), 7.51 (dd, *J* = 9.0, 2.7 Hz, 1H), 7.40 (td, *J* = 8.6, 2.8 Hz, 1H), 3.34 (s, 3H), 1.09 (s, 21H).

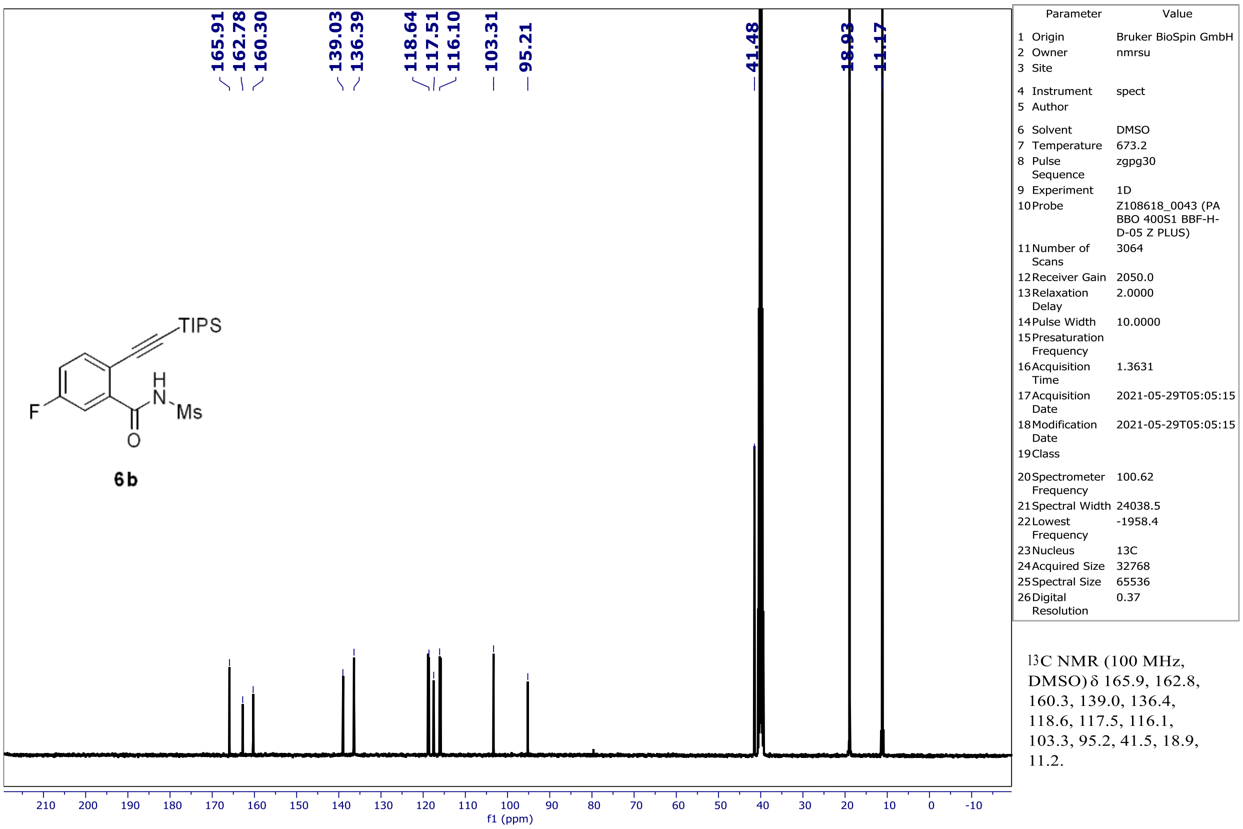

^{13}C NMR (100 MHz, DMSO) δ 165.9, 162.8, 160.3, 139.0, 136.4, 118.6, 117.5, 116.1, 103.3, 95.2, 41.5, 18.9, 11.2.

5-Fluoro-*N*-(methylsulfonyl)-2-((triisopropylsilyl)ethynyl)benzamide (6b)

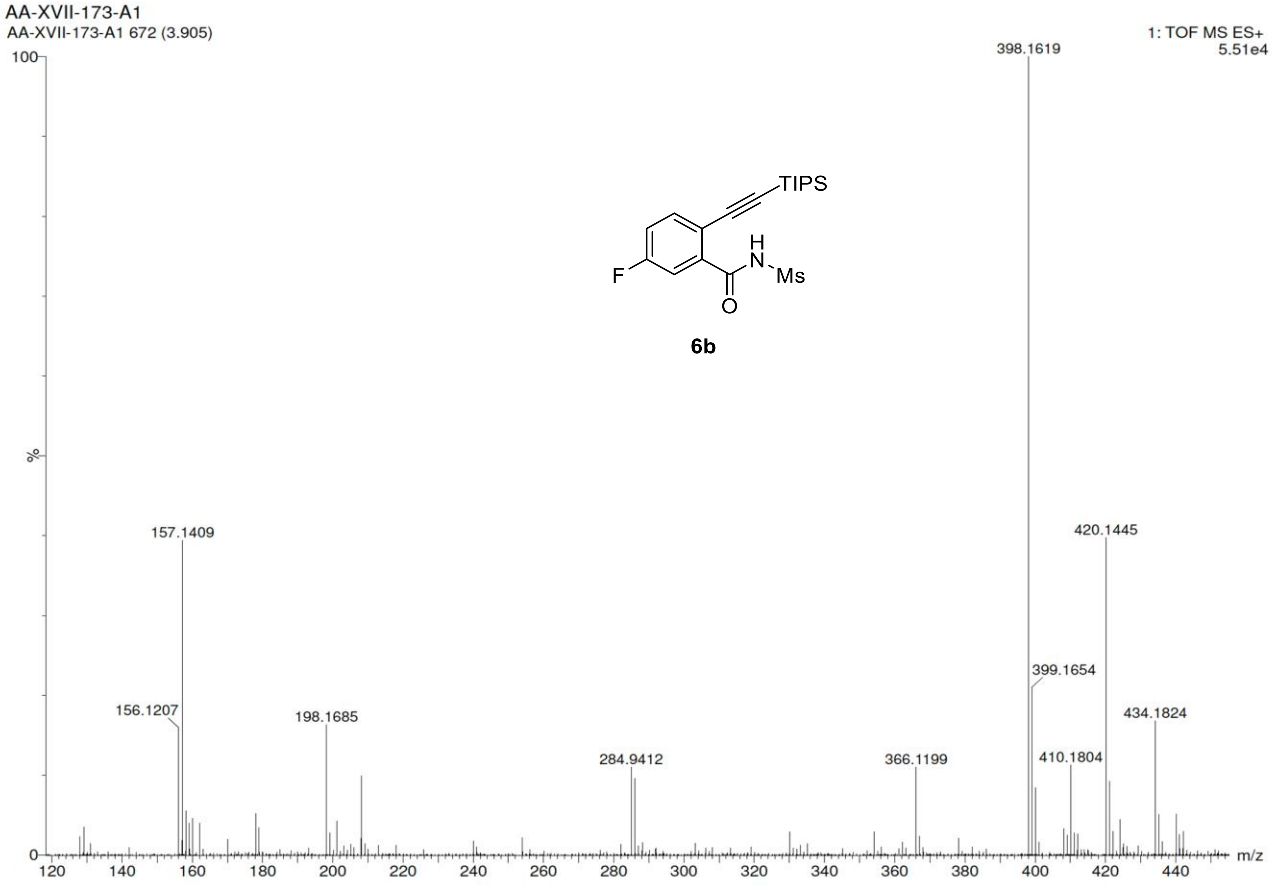

HRMS (ESI) *m/z* calcd for $C_{19}H_{28}FNO_3SSi$ $[M + H]^+$ 398.1616; found 398.1619.

5-Bromo-*N*-(methylsulfonyl)-2-((triisopropylsilyl)ethynyl)benzamide (6c)

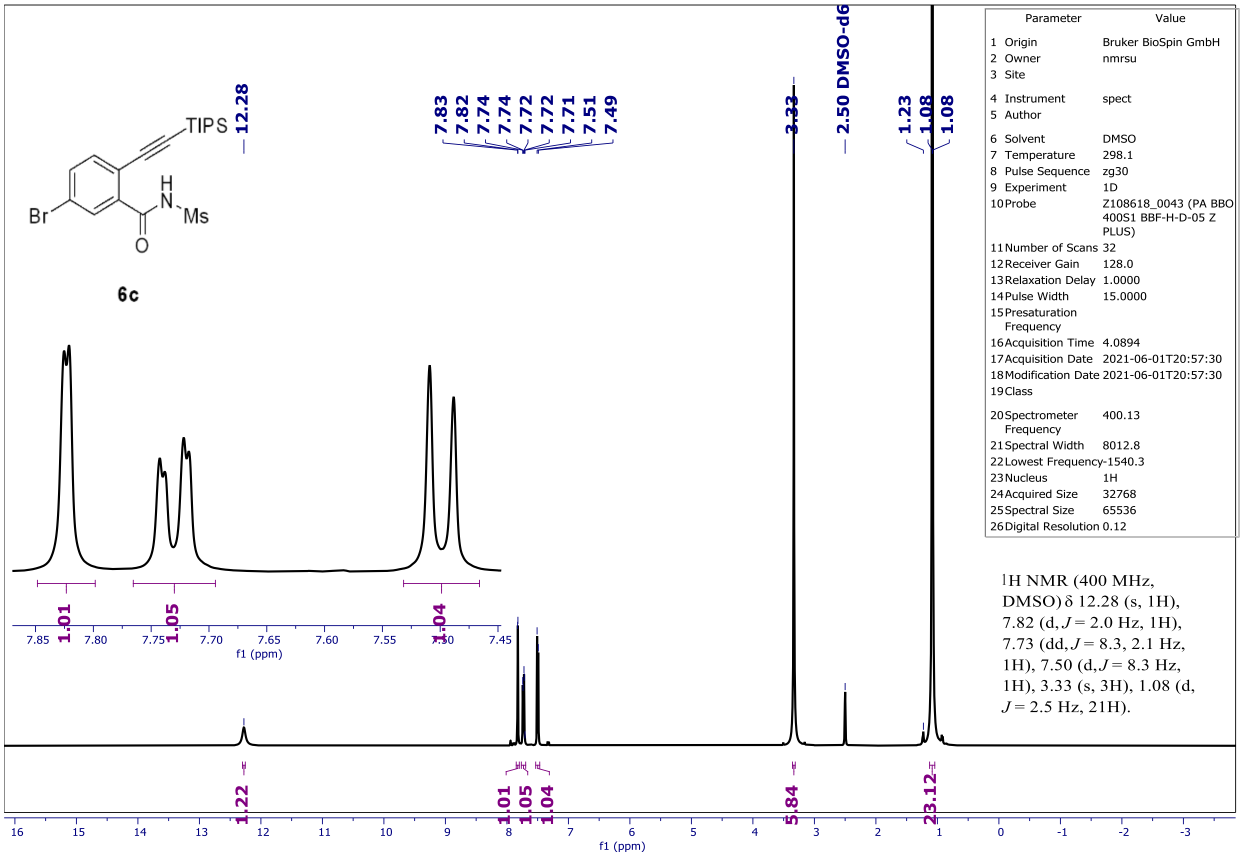

Parameter	Value
1 Origin	Bruker BioSpin GmbH
2 Owner	nmrsu
3 Site	
4 Instrument	spect
5 Author	
6 Solvent	DMSO
7 Temperature	298.1
8 Pulse Sequence	zg30
9 Experiment	1D
10 Probe	Z108618_0043 (PA BBO 400S1 BBF-H-D-05 Z PLUS)
11 Number of Scans	32
12 Receiver Gain	128.0
13 Relaxation Delay	1.0000
14 Pulse Width	15.0000
15 Presaturation Frequency	
16 Acquisition Time	4.0894
17 Acquisition Date	2021-06-01T20:57:30
18 Modification Date	2021-06-01T20:57:30
19 Class	
20 Spectrometer Frequency	400.13
21 Spectral Width	8012.8
22 Lowest Frequency	-1540.3
23 Nucleus	1H
24 Acquired Size	32768
25 Spectral Size	65536
26 Digital Resolution	0.12

^{1}H NMR (400 MHz, DMSO) δ 12.28 (s, 1H), 7.82 (d, J = 2.0 Hz, 1H), 7.73 (dd, J = 8.3, 2.1 Hz, 1H), 7.50 (d, J = 8.3 Hz, 1H), 3.33 (s, 3H), 1.08 (d, J = 2.5 Hz, 21H).

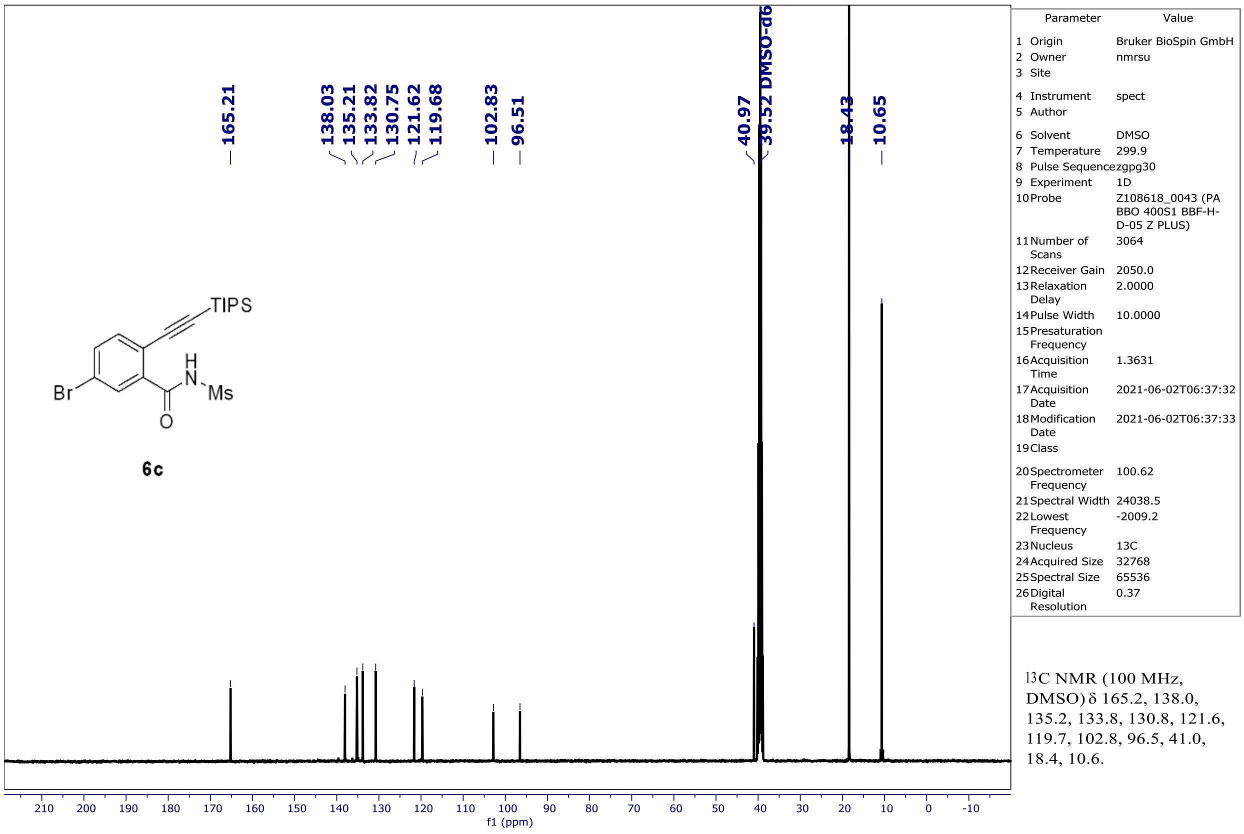

Parameter	Value
1 Origin	Bruker BioSpin GmbH
2 Owner	nmrsu
3 Site	
4 Instrument	spect
5 Author	
6 Solvent	DMSO
7 Temperature	299.9
8 Pulse Sequence	zgpg30
9 Experiment	1D
10 Probe	Z108618_0043 (PA BBO 400S1 BBF-H-D-05 Z PLUS)
11 Number of Scans	3064
12 Receiver Gain	2050.0
13 Relaxation Delay	2.0000
14 Pulse Width	10.0000
15 Presaturation Frequency	
16 Acquisition Time	1.3631
17 Acquisition Date	2021-06-02T06:37:32
18 Modification Date	2021-06-02T06:37:33
19 Class	
20 Spectrometer Frequency	100.62
21 Spectral Width	24038.5
22 Lowest Frequency	-2009.2
23 Nucleus	13C
24 Acquired Size	32768
25 Spectral Size	65536
26 Digital Resolution	0.37

^{13}C NMR (100 MHz, DMSO) δ 165.2, 138.0, 135.2, 133.8, 130.8, 121.6, 119.7, 102.8, 96.5, 41.0, 18.4, 10.6.

5-Bromo-*N*-(methylsulfonyl)-2-((triisopropylsilyl)ethynyl)benzamide (6c)

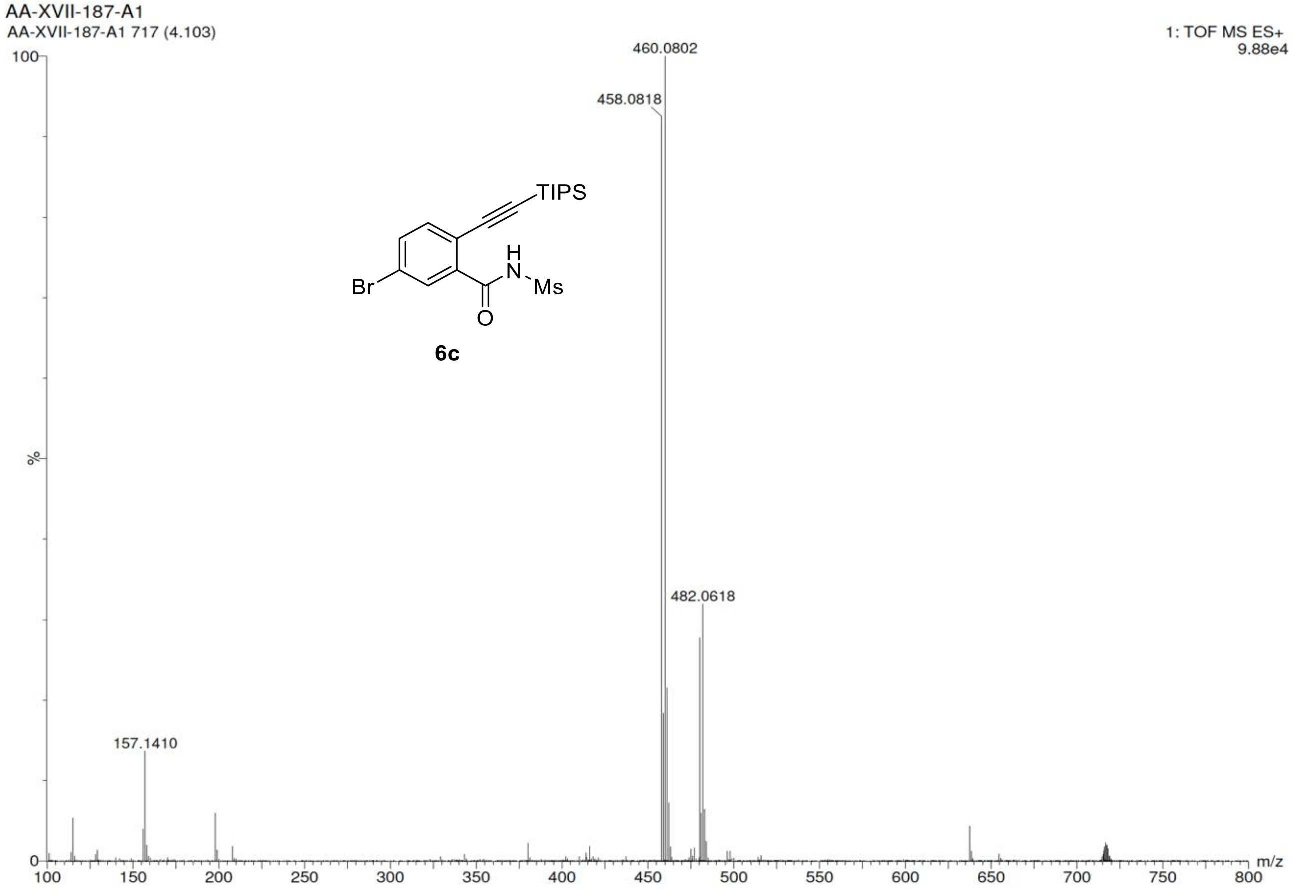

HRMS (ESI) *m/z* calcd for $C_{19}H_{28}BrNO_3SSi$ $[M + H]^+$ 458.0815; found 458.0818.

5-Methyl-*N*-(methylsulfonyl)-2-((triisopropylsilyl)ethynyl)benzamide (6d)

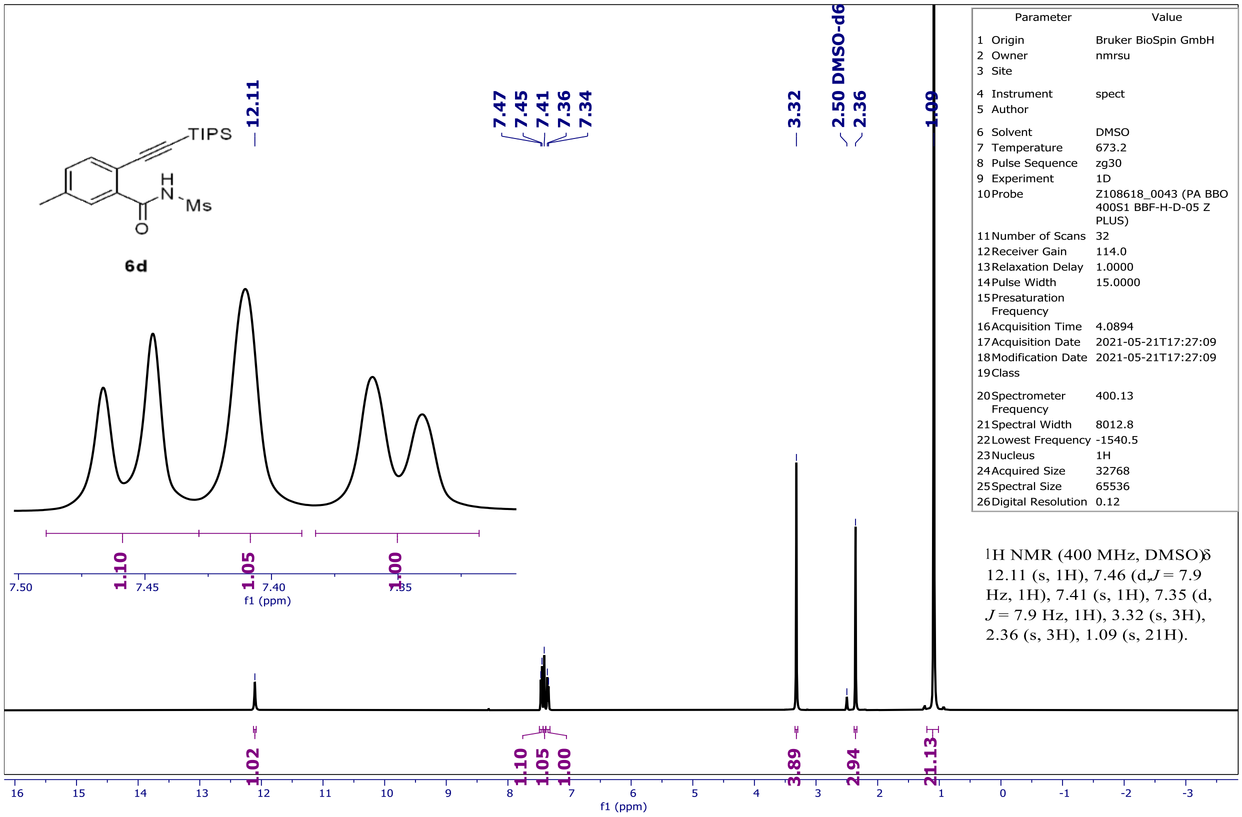

Parameter	Value
1 Origin	Bruker BioSpin GmbH
2 Owner	nmrsu
3 Site	
4 Instrument	spect
5 Author	
6 Solvent	DMSO
7 Temperature	673.2
8 Pulse Sequence	zg30
9 Experiment	1D
10 Probe	Z108618_0043 (PA BBO 400S1 BBF-H-D-05 Z PLUS)
11 Number of Scans	32
12 Receiver Gain	114.0
13 Relaxation Delay	1.0000
14 Pulse Width	15.0000
15 Presaturation Frequency	
16 Acquisition Time	4.0894
17 Acquisition Date	2021-05-21T17:27:09
18 Modification Date	2021-05-21T17:27:09
19 Class	
20 Spectrometer Frequency	400.13
21 Spectral Width	8012.8
22 Lowest Frequency	-1540.5
23 Nucleus	1H
24 Acquired Size	32768
25 Spectral Size	65536
26 Digital Resolution	0.12

^{1}H NMR (400 MHz, DMSO) δ 12.11 (s, 1H), 7.46 (d, *J* = 7.9 Hz, 1H), 7.41 (s, 1H), 7.35 (d, *J* = 7.9 Hz, 1H), 3.32 (s, 3H), 2.36 (s, 3H), 1.09 (s, 21H).

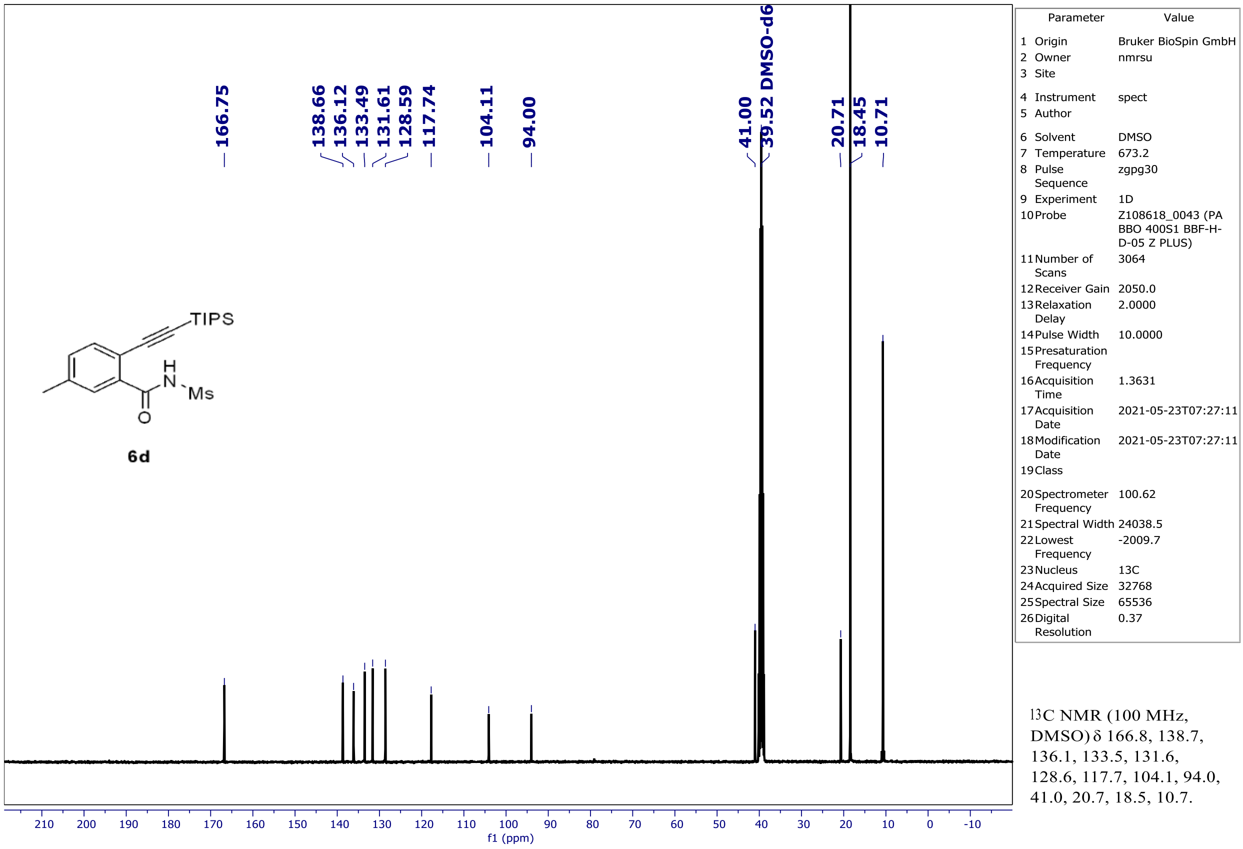

Parameter	Value
1 Origin	Bruker BioSpin GmbH
2 Owner	nmrsu
3 Site	
4 Instrument	spect
5 Author	
6 Solvent	DMSO
7 Temperature	673.2
8 Pulse Sequence	zgpg30
9 Experiment	1D
10 Probe	Z108618_0043 (PA BBO 400S1 BBF-H-D-05 Z PLUS)
11 Number of Scans	3064
12 Receiver Gain	2050.0
13 Relaxation Delay	2.0000
14 Pulse Width	10.0000
15 Presaturation Frequency	
16 Acquisition Time	1.3631
17 Acquisition Date	2021-05-23T07:27:11
18 Modification Date	2021-05-23T07:27:11
19 Class	
20 Spectrometer Frequency	100.62
21 Spectral Width	24038.5
22 Lowest Frequency	-2009.7
23 Nucleus	13C
24 Acquired Size	32768
25 Spectral Size	65536
26 Digital Resolution	0.37

^{13}C NMR (100 MHz, DMSO) δ 166.8, 138.7, 136.1, 133.5, 131.6, 128.6, 117.7, 104.1, 94.0, 41.0, 20.7, 18.5, 10.7.

5-Methyl-*N*-(methylsulfonyl)-2-((triisopropylsilyl)ethynyl)benzamide (6d)

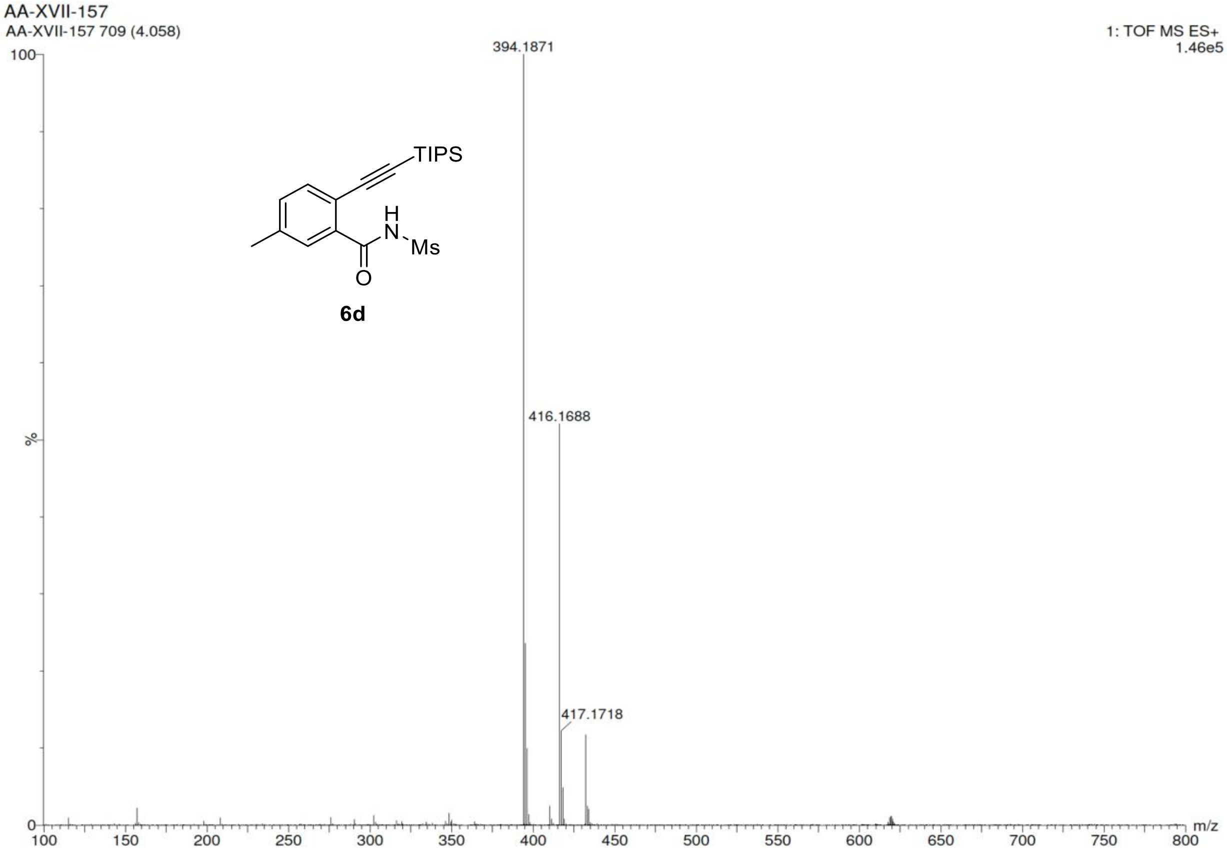

HRMS (ESI) *m/z* calcd for $C_{20}H_{31}NO_3SSi$ $[M + H]^+$ 394.1867; found 394.1871.

5-Methoxy-*N*-(methylsulfonyl)-2-((triisopropylsilyl)ethynyl)benzamide (6e)

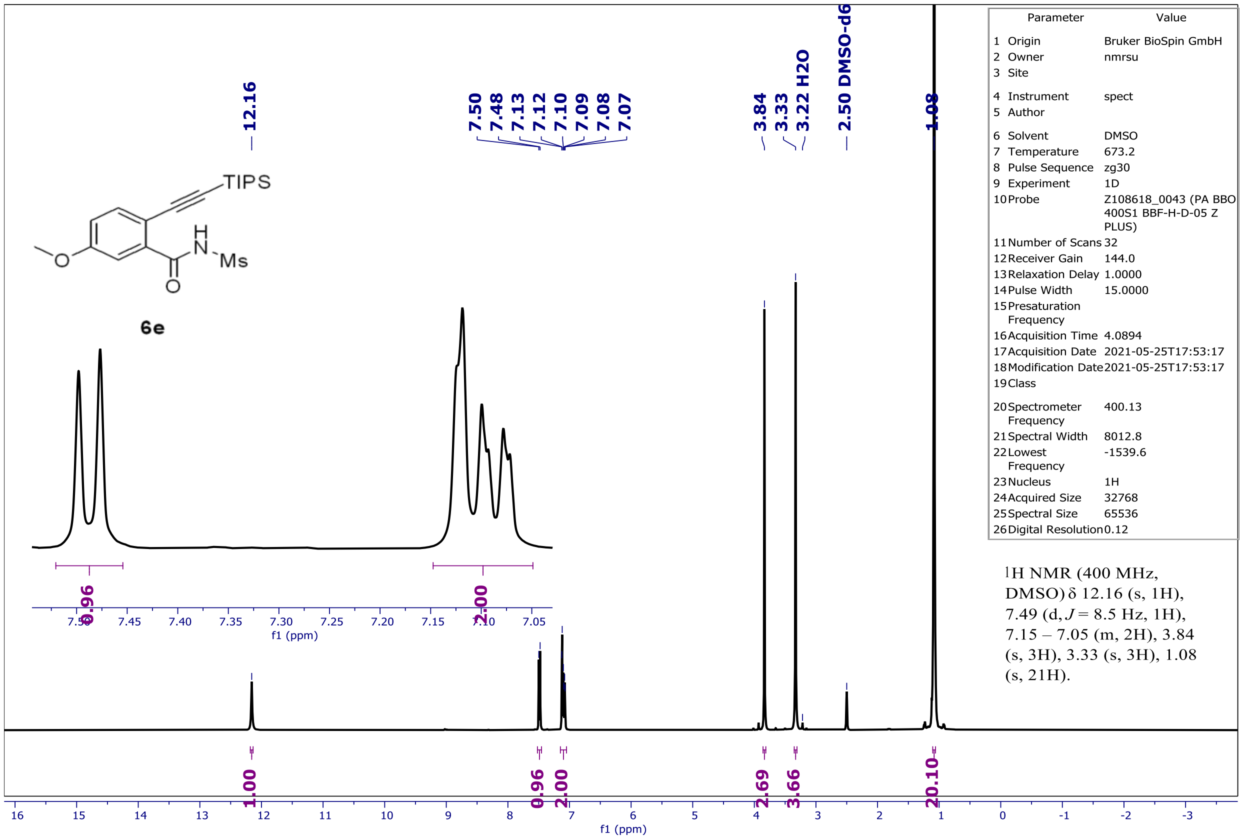

Parameter	Value
1 Origin	Bruker BioSpin GmbH
2 Owner	nmrsu
3 Site	
4 Instrument	spect
5 Author	
6 Solvent	DMSO
7 Temperature	673.2
8 Pulse Sequence	zg30
9 Experiment	1D
10 Probe	Z108618_0043 (PA BBO 400S1 BBF-H-D-05 Z PLUS)
11 Number of Scans	32
12 Receiver Gain	144.0
13 Relaxation Delay	1.0000
14 Pulse Width	15.0000
15 Presaturation Frequency	
16 Acquisition Time	4.0894
17 Acquisition Date	2021-05-25T17:53:17
18 Modification Date	2021-05-25T17:53:17
19 Class	
20 Spectrometer Frequency	400.13
21 Spectral Width	8012.8
22 Lowest Frequency	-1539.6
23 Nucleus	1H
24 Acquired Size	32768
25 Spectral Size	65536
26 Digital Resolution	0.12

^{1}H NMR (400 MHz, DMSO) δ 12.16 (s, 1H), 7.49 (d, *J* = 8.5 Hz, 1H), 7.15 – 7.05 (m, 2H), 3.84 (s, 3H), 3.33 (s, 3H), 1.08 (s, 21H).

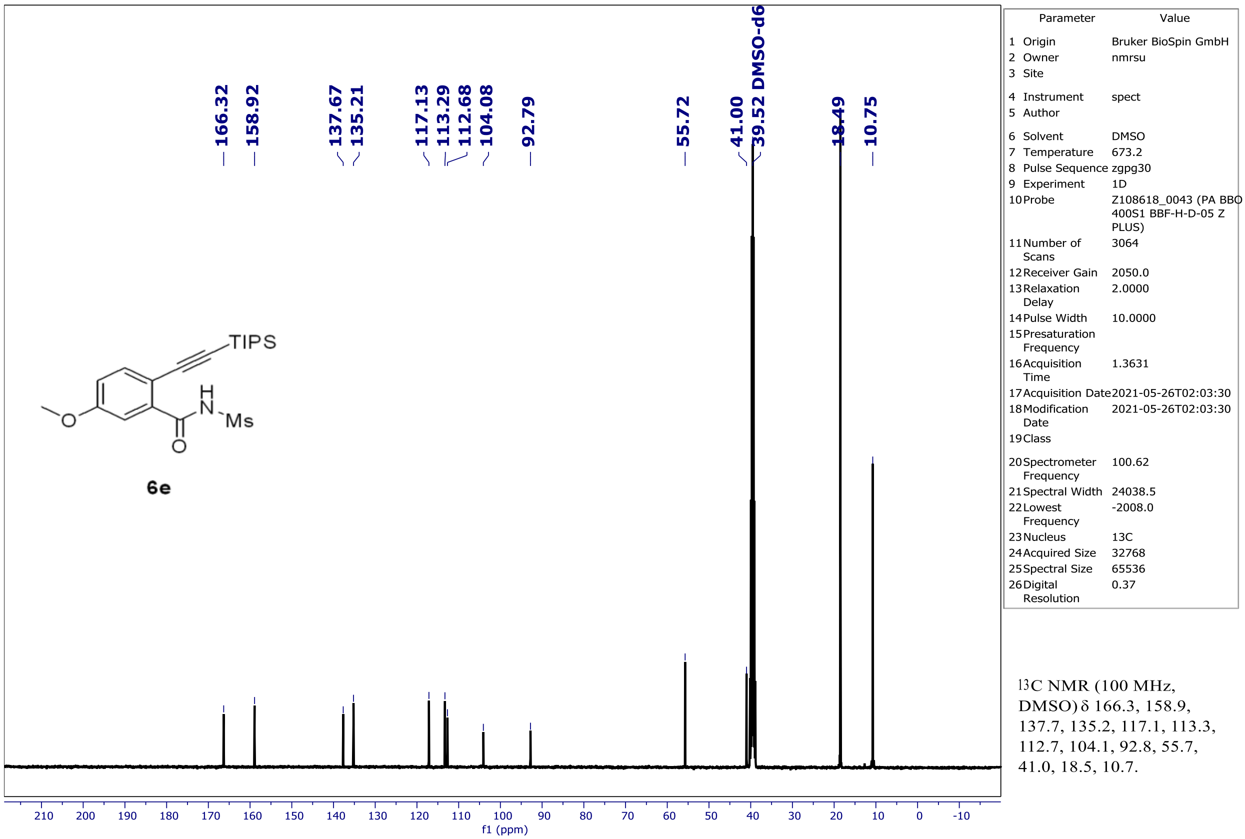

	Parameter	Value
1	Origin	Bruker BioSpin GmbH
2	Owner	nmrsu
3	Site	
4	Instrument	spect
5	Author	
6	Solvent	DMSO
7	Temperature	673.2
8	Pulse Sequence	zgpg30
9	Experiment	1D
10	Probe	Z108618_0043 (PA BBO 400S1 BBF-H-D-05 Z PLUS)
11	Number of Scans	3064
12	Receiver Gain	2050.0
13	Relaxation Delay	2.0000
14	Pulse Width	10.0000
15	Presaturation Frequency	
16	Acquisition Time	1.3631
17	Acquisition Date	2021-05-26T02:03:30
18	Modification Date	2021-05-26T02:03:30
19	Class	
20	Spectrometer Frequency	100.62
21	Spectral Width	24038.5
22	Lowest Frequency	-2008.0
23	Nucleus	13C
24	Acquired Size	32768
25	Spectral Size	65536
26	Digital Resolution	0.37

^{13}C NMR (100 MHz, DMSO) δ 166.3, 158.9, 137.7, 135.2, 117.1, 113.3, 112.7, 104.1, 92.8, 55.7, 41.0, 18.5, 10.7.

5-Methoxy-*N*-(methylsulfonyl)-2-((triisopropylsilyl)ethynyl)benzamide (6e)

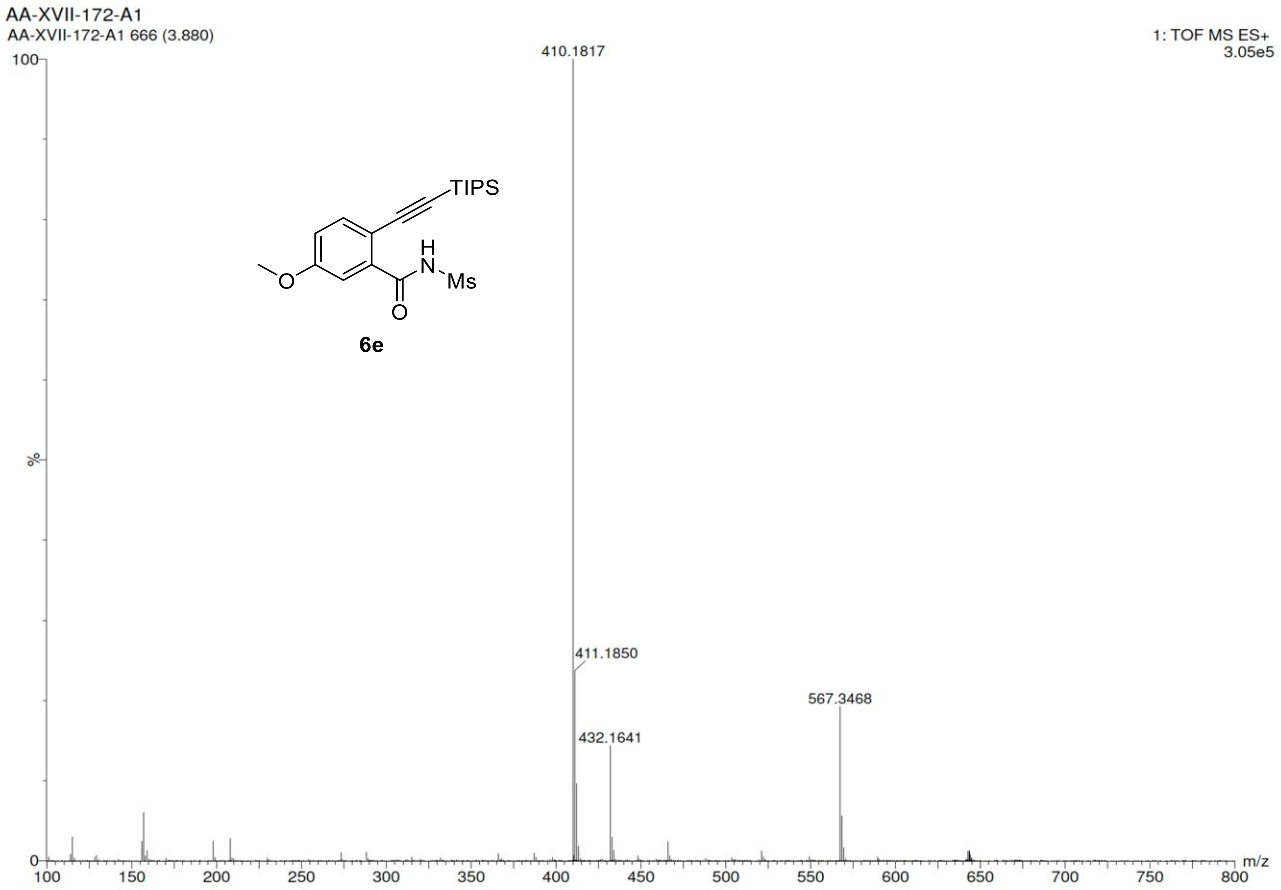

HRMS (ESI) *m/z* calcd for $C_{20}H_{31}NO_4SSi$ $[M + H]^+$ 410.1816; found 410.1817.

N-(Methylsulfonyl)-5-nitro-2-((triisopropylsilyl)ethynyl)benzamide (6f)

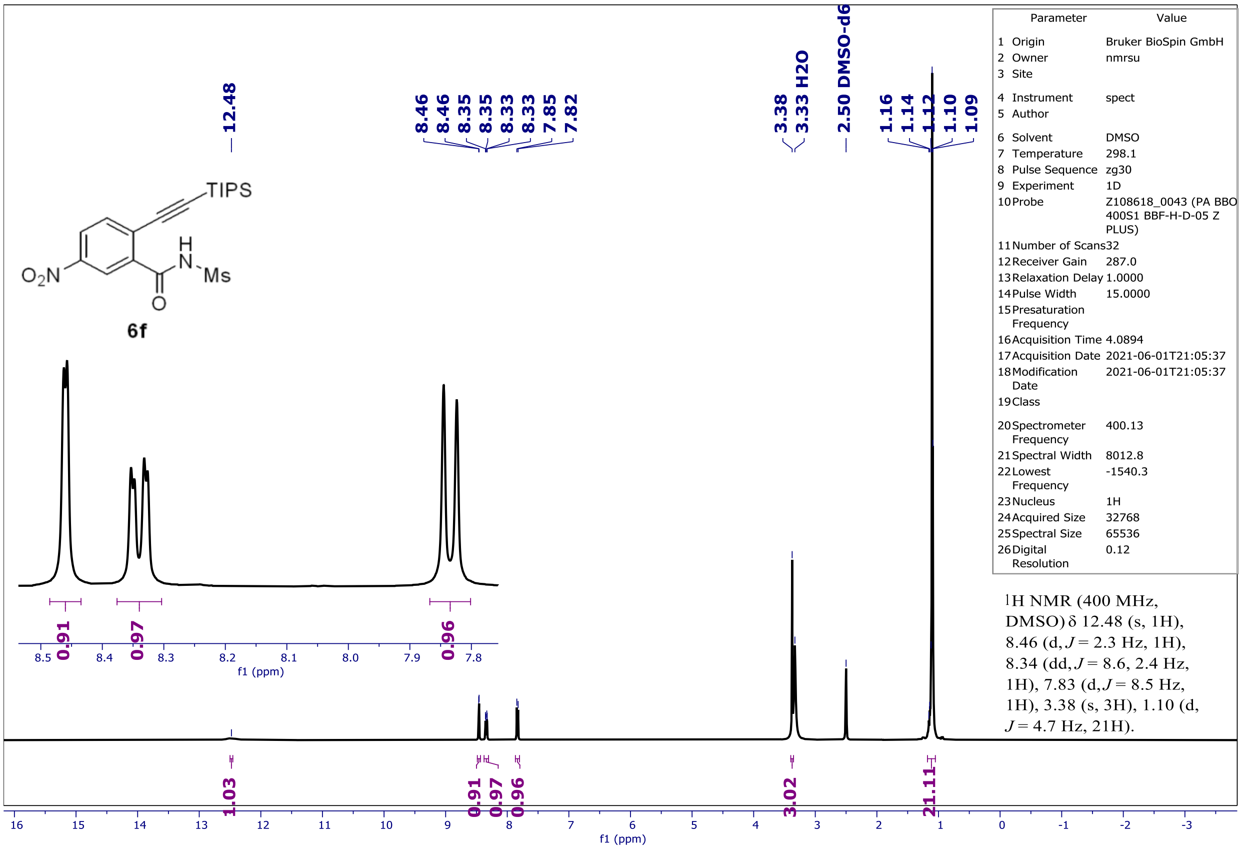

^{1}H NMR (400 MHz, DMSO) δ 12.48 (s, 1H), 8.46 (d, *J* = 2.3 Hz, 1H), 8.34 (dd, *J* = 8.6, 2.4 Hz, 1H), 7.83 (d, *J* = 8.5 Hz, 1H), 3.38 (s, 3H), 1.10 (d, *J* = 4.7 Hz, 21H).

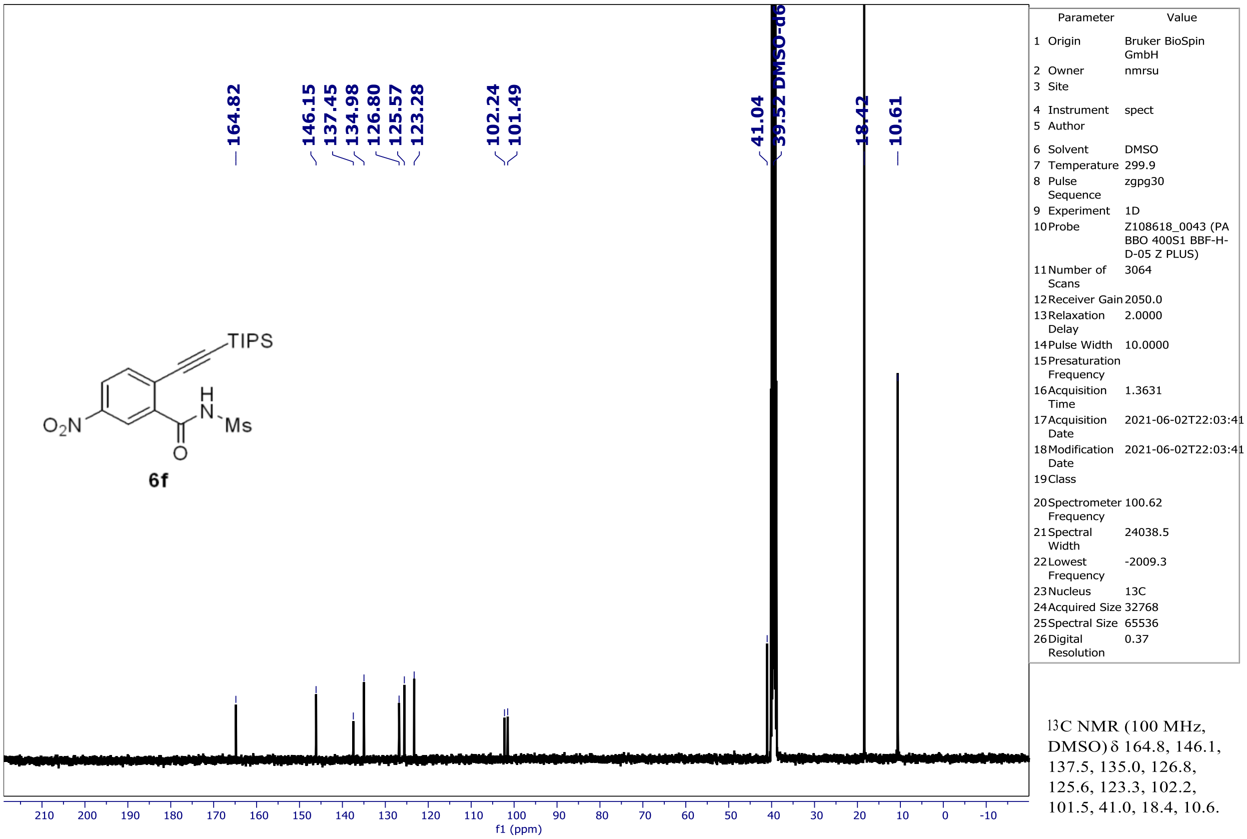

^{13}C NMR (100 MHz, DMSO) δ 164.8, 146.1, 137.5, 135.0, 126.8, 125.6, 123.3, 102.2, 101.5, 41.0, 18.4, 10.6.

***N*-(Methylsulfonyl)-5-nitro-2-((triisopropylsilyl)ethynyl)benzamide (6f)**

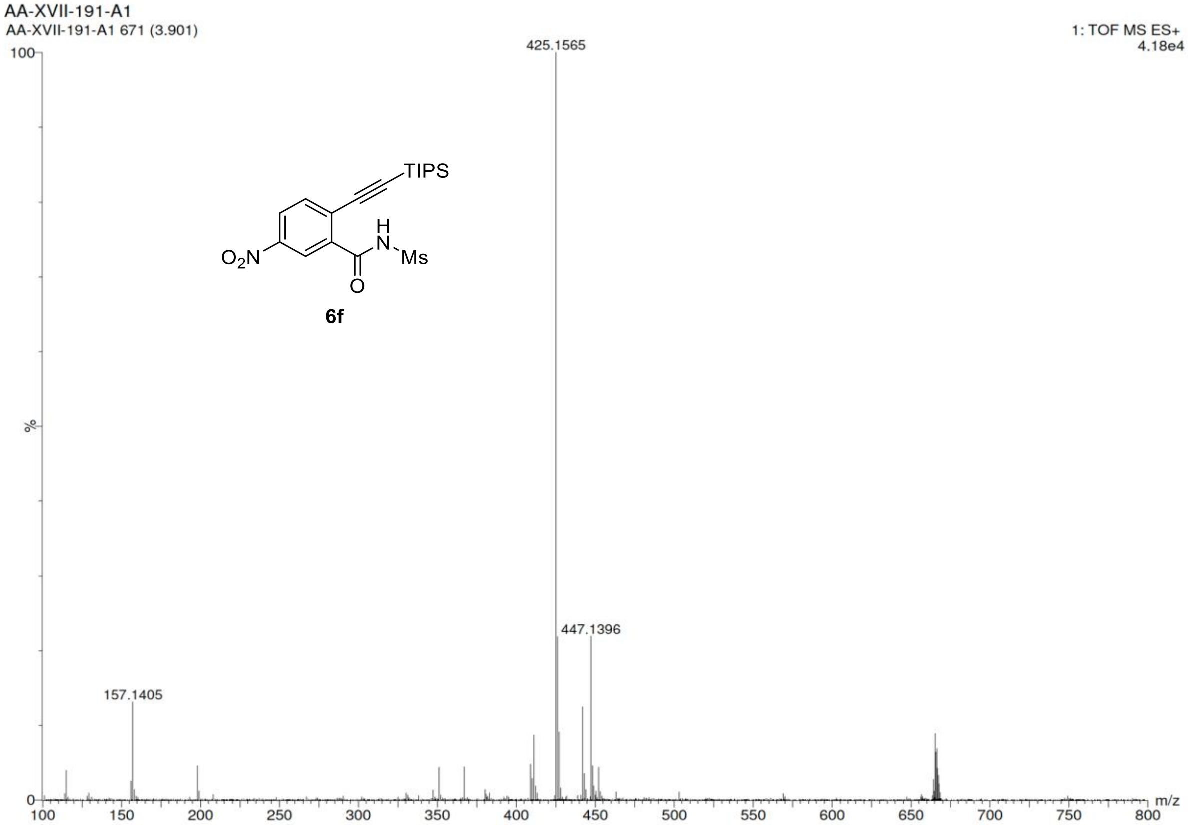

HRMS (ESI) *m/z* calcd for $C_{20}H_{31}NO_4SSi$ $[M + H]^+$ 425.1561; found 425.1565.

4-Chloro-*N*-(methylsulfonyl)-2-((triisopropylsilyl)ethynyl)benzamide (6g)

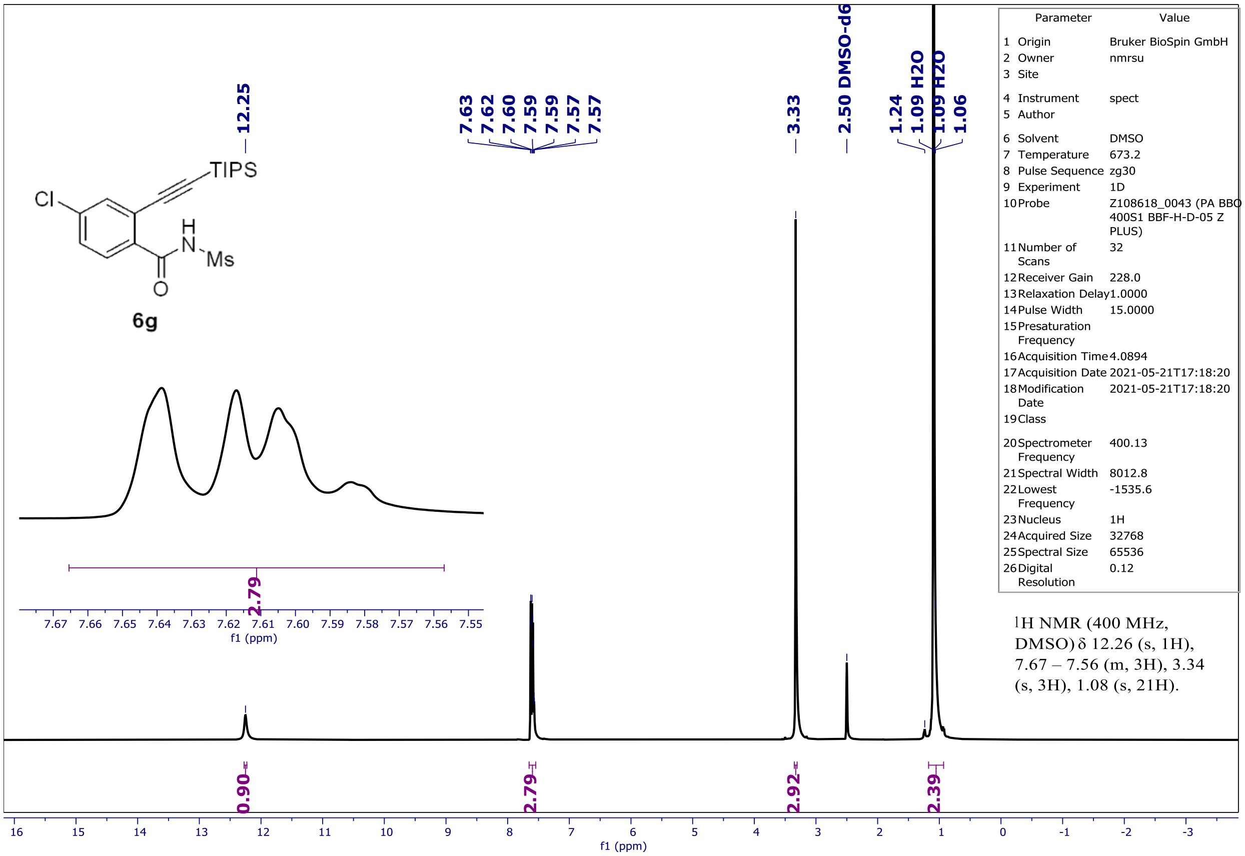

^{1}H NMR (400 MHz, DMSO) δ 12.26 (s, 1H), 7.67 – 7.56 (m, 3H), 3.34 (s, 3H), 1.08 (s, 21H).

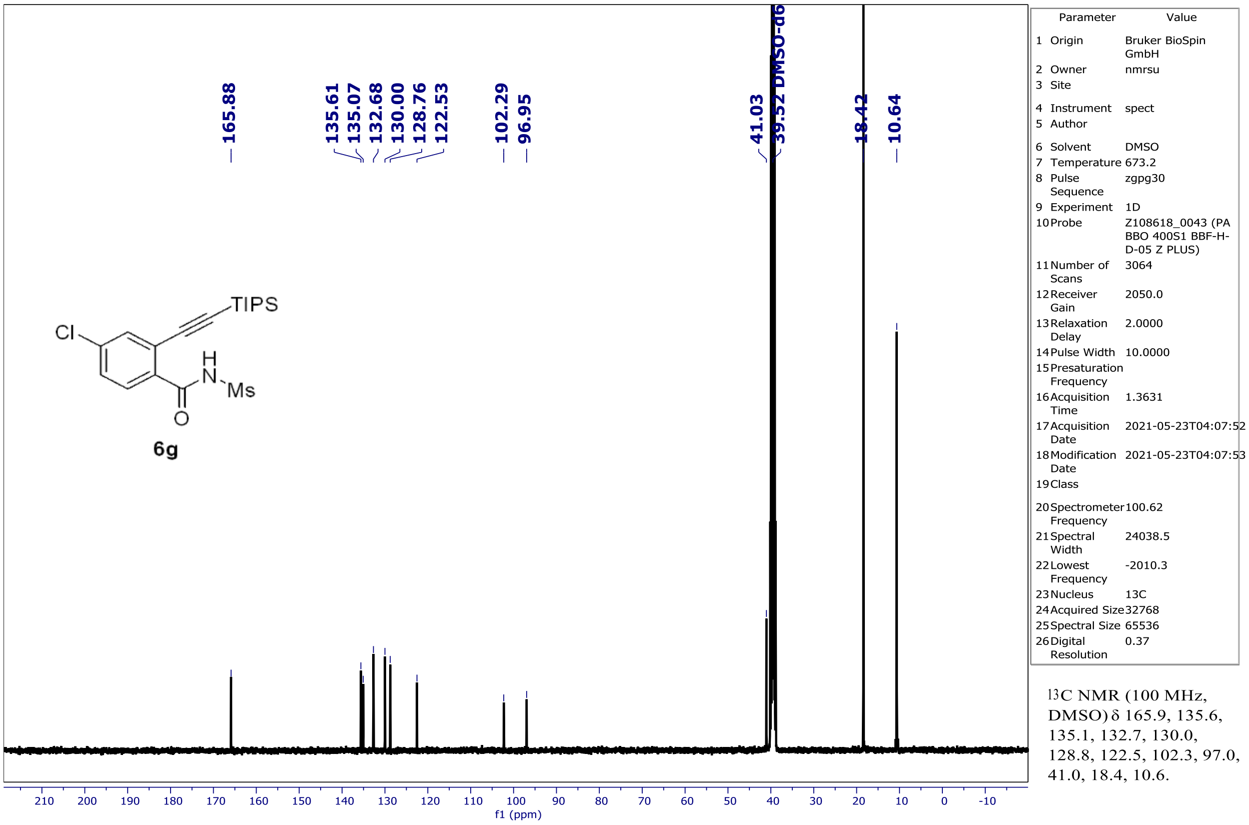

^{13}C NMR (100 MHz, DMSO) δ 165.9, 135.6, 135.1, 132.7, 130.0, 128.8, 122.5, 102.3, 97.0, 41.0, 18.4, 10.6.

4-Chloro-*N*-(methylsulfonyl)-2-((triisopropylsilyl)ethynyl)benzamide (6g)

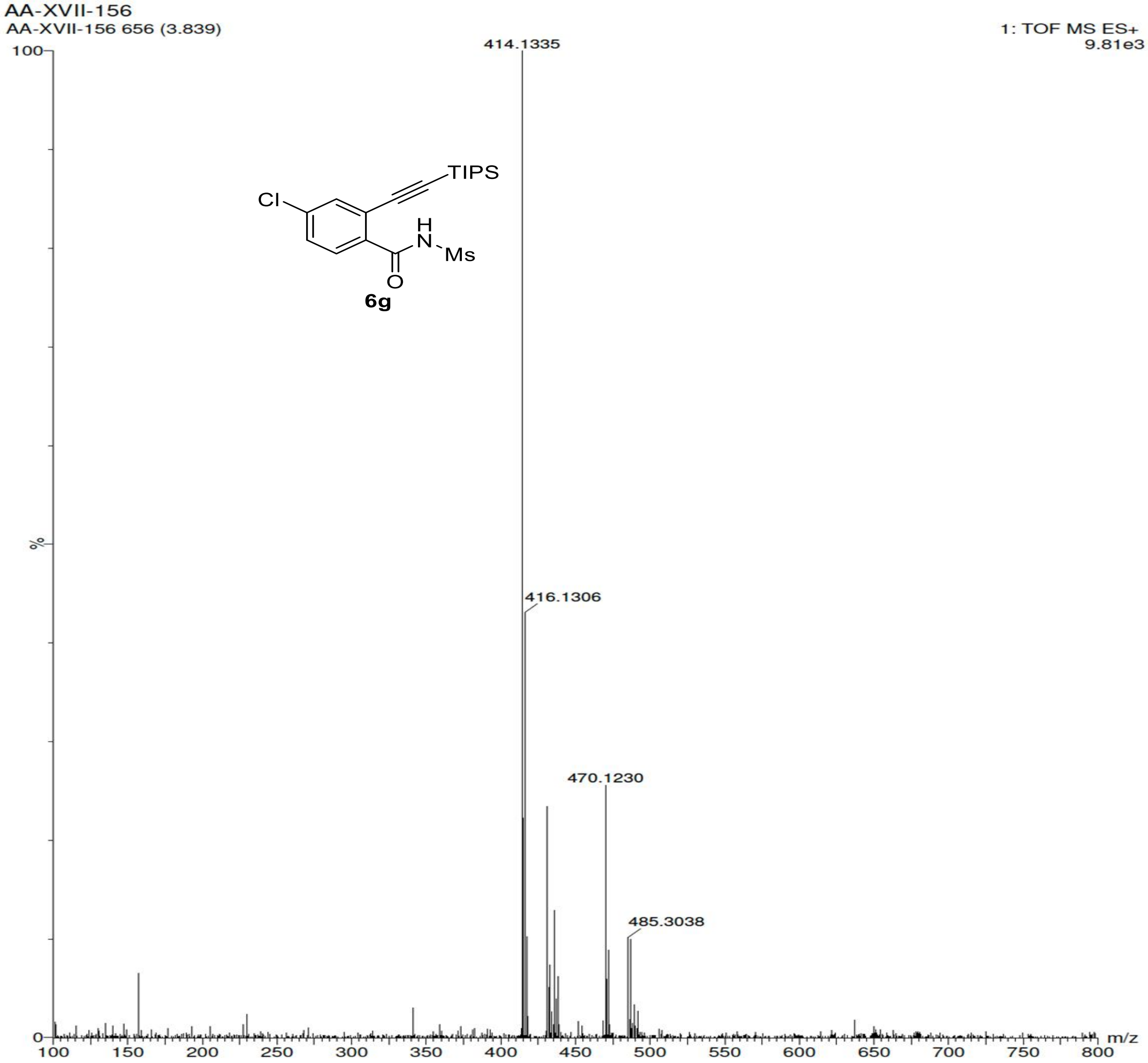

HRMS (ESI) *m/z* calcd for $C_{19}H_{28}ClNO_3SSi$ $[M + H]^+$ 414.1320; found 414.1335.

N-(Methylsulfonyl)-4-nitro-2-((triisopropylsilyl)ethynyl)benzamide (6h)

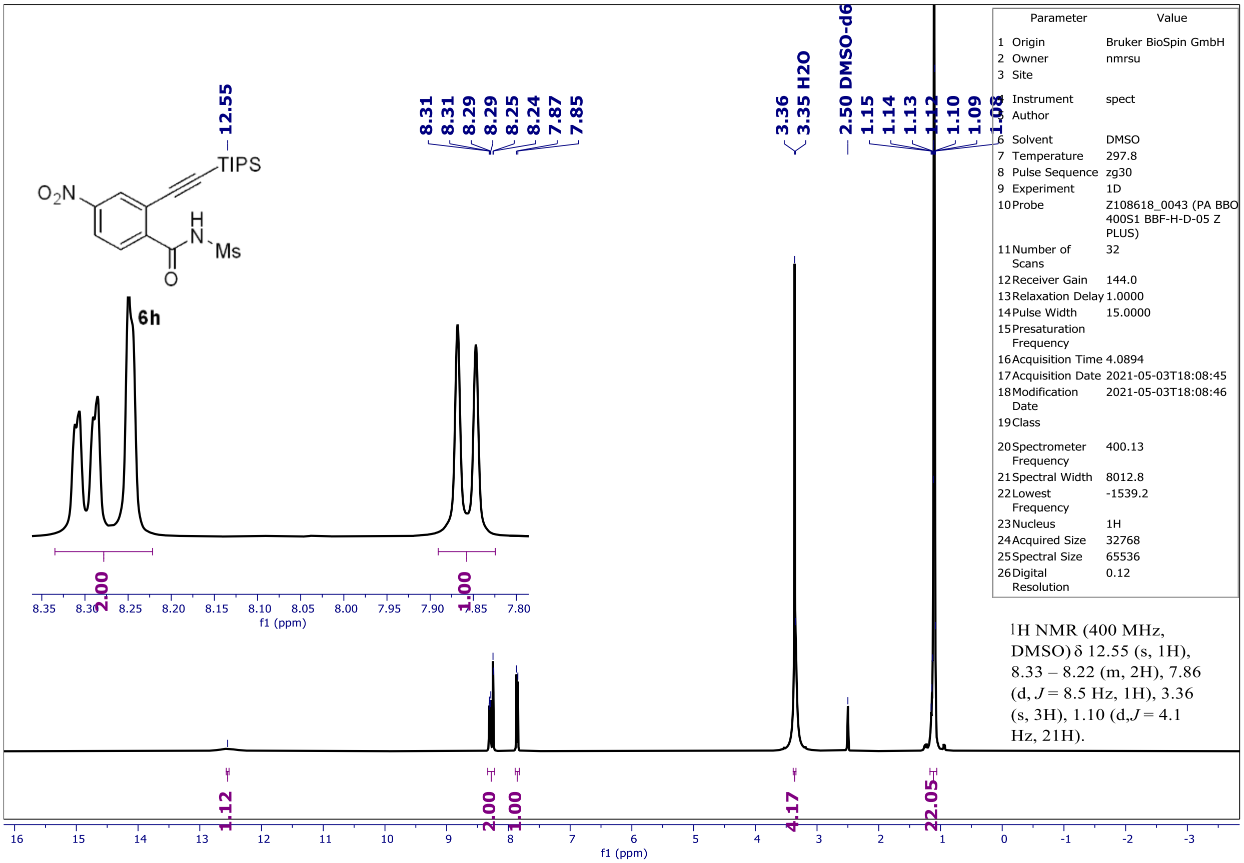

^{1}H NMR (400 MHz, DMSO) δ 12.55 (s, 1H), 8.33 – 8.22 (m, 2H), 7.86 (d, J = 8.5 Hz, 1H), 3.36 (s, 3H), 1.10 (d, J = 4.1 Hz, 21H).

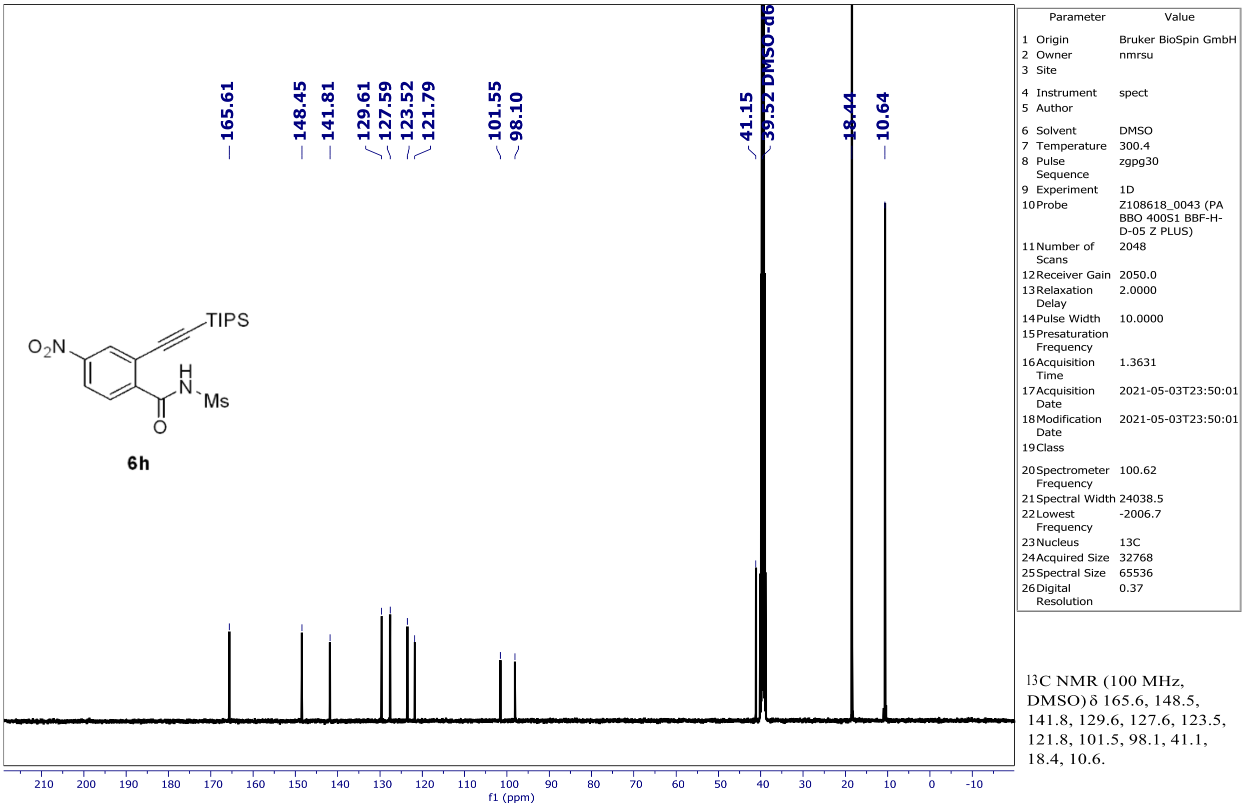

^{13}C NMR (100 MHz, DMSO) δ 165.6, 148.5, 141.8, 129.6, 127.6, 123.5, 121.8, 101.5, 98.1, 41.1, 18.4, 10.6.

N-(Methylsulfonyl)-4-nitro-2-((triisopropylsilyl)ethynyl)benzamide (6h)

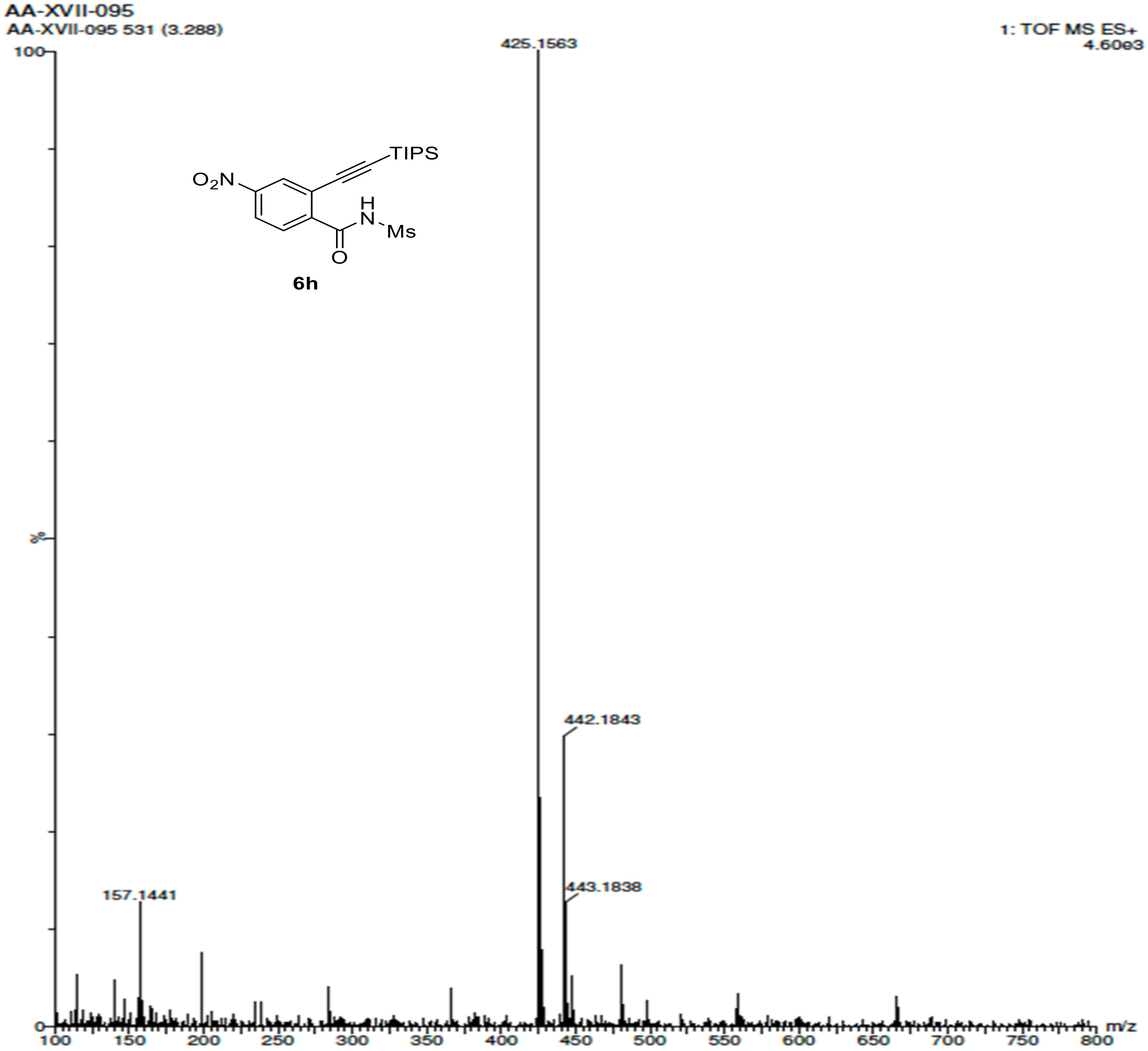

HRMS (ESI) *m/z* calcd for $C_{20}H_{31}NO_4SSi$ $[M + H]^+$ 425.1561; found 425.1563.

Isoquinolin-1(2*H*)-one (7a)

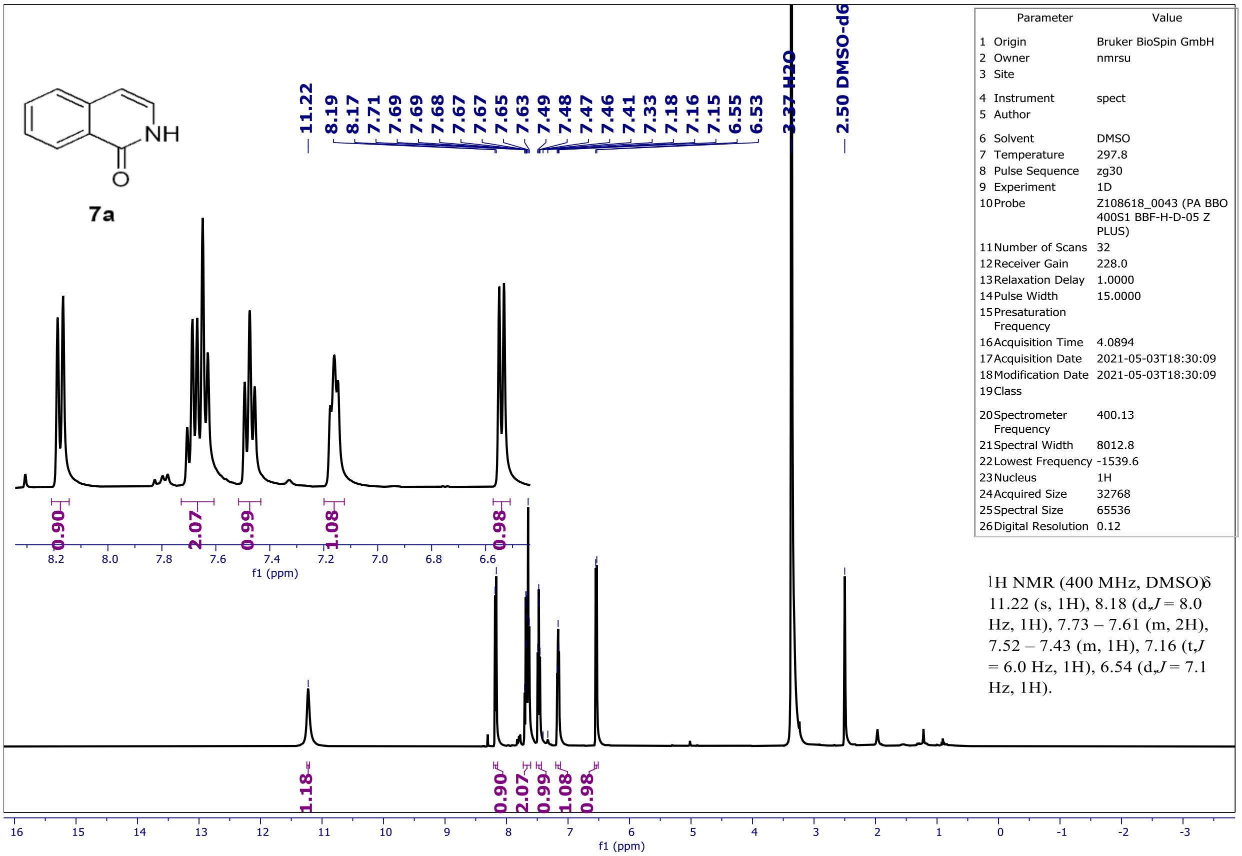

^{1}H NMR (400 MHz, DMSO) δ 11.22 (s, 1H), 8.18 (d, *J* = 8.0 Hz, 1H), 7.73 – 7.61 (m, 2H), 7.52 – 7.43 (m, 1H), 7.16 (t, *J* = 6.0 Hz, 1H), 6.54 (d, *J* = 7.1 Hz, 1H).

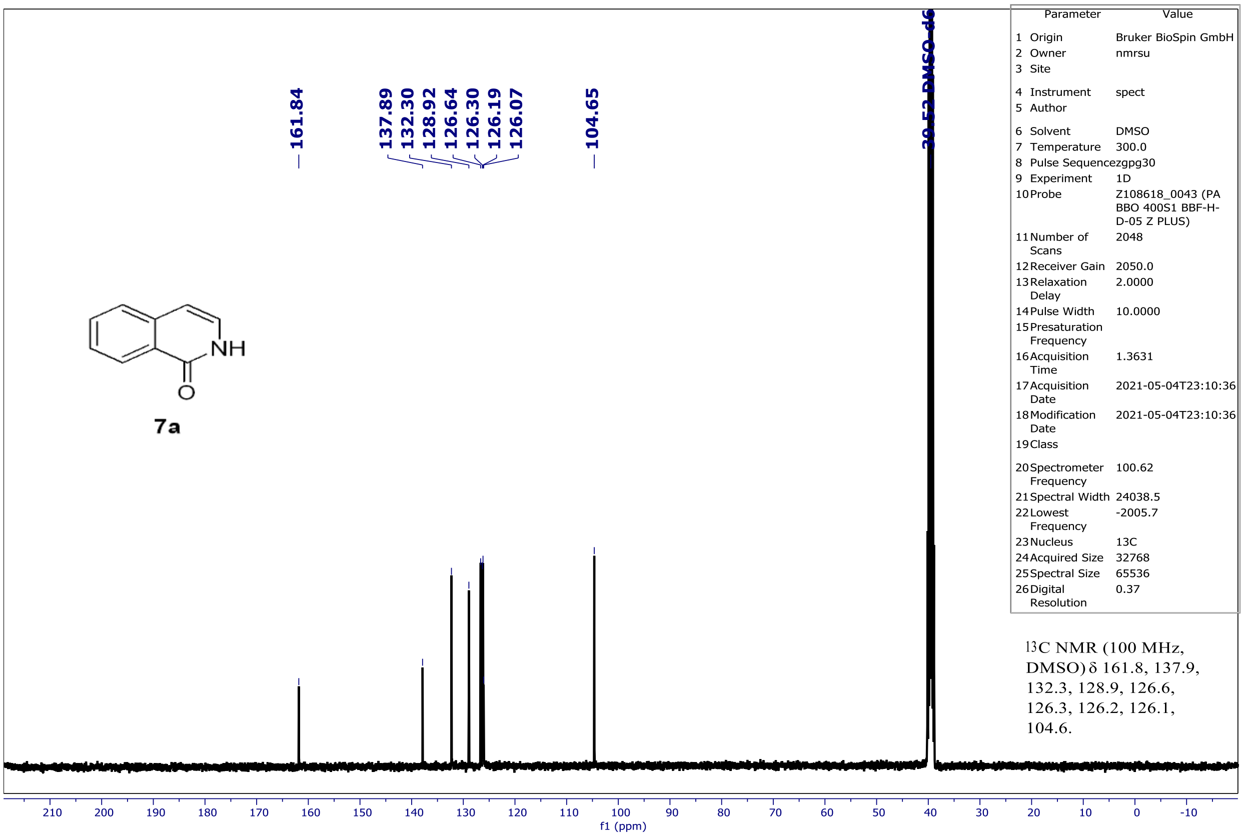

^{13}C NMR (100 MHz, DMSO) δ 161.8, 137.9, 132.3, 128.9, 126.6, 126.3, 126.2, 126.1, 104.6.

COSY

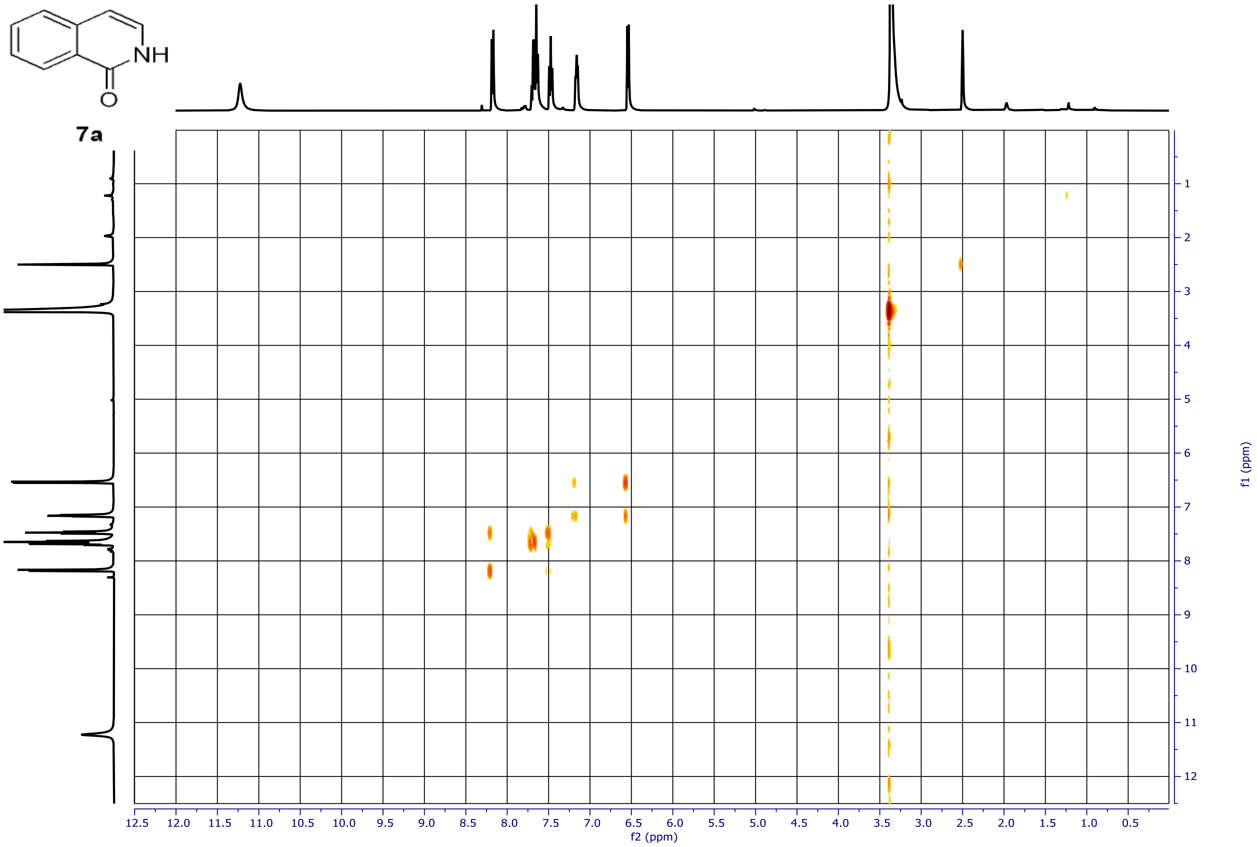

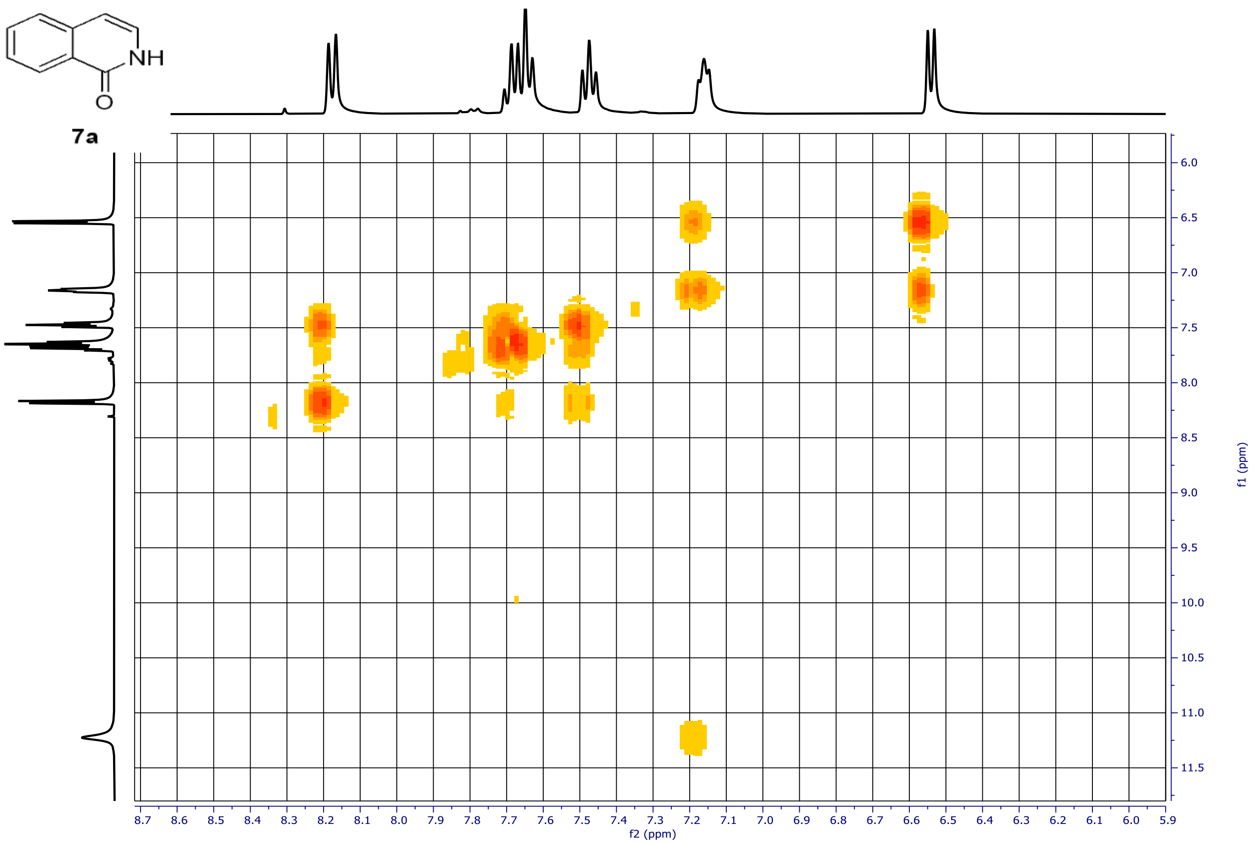

HSQC

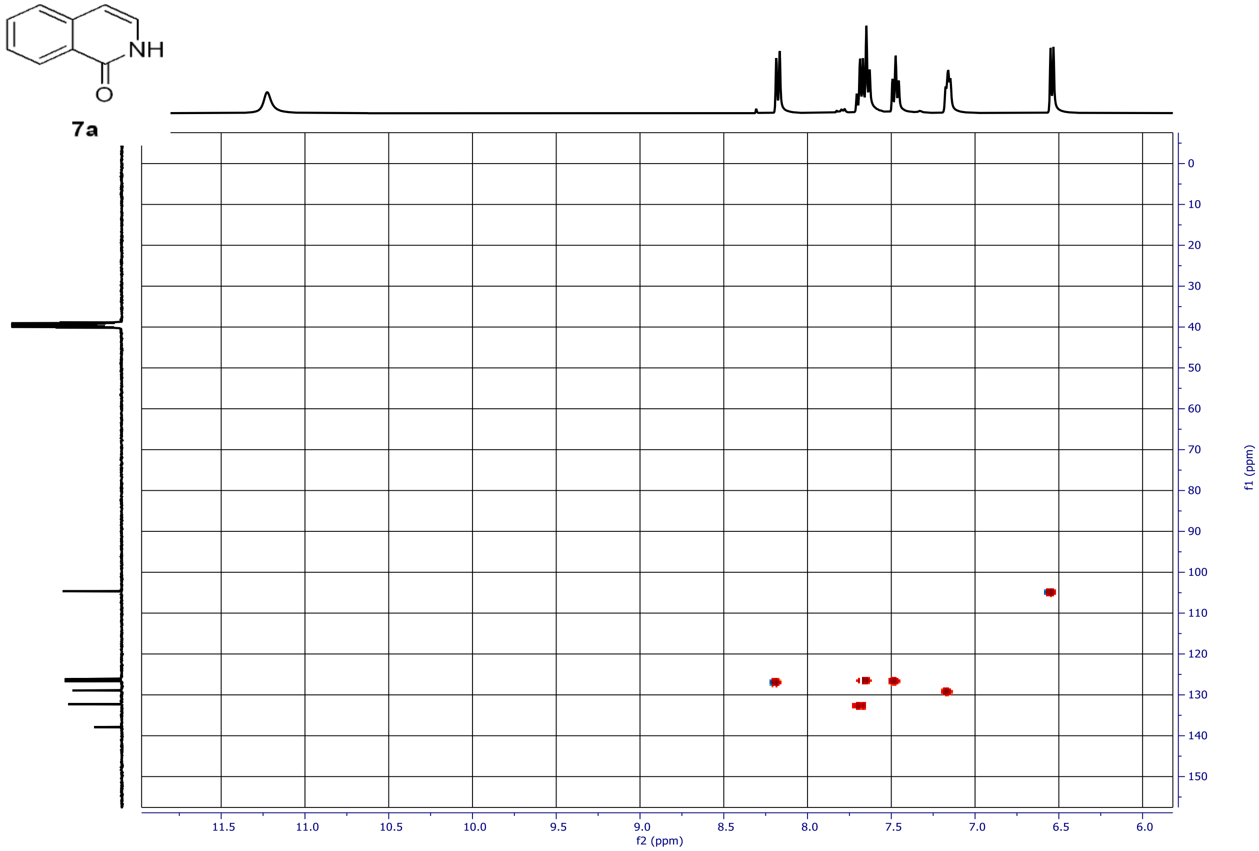

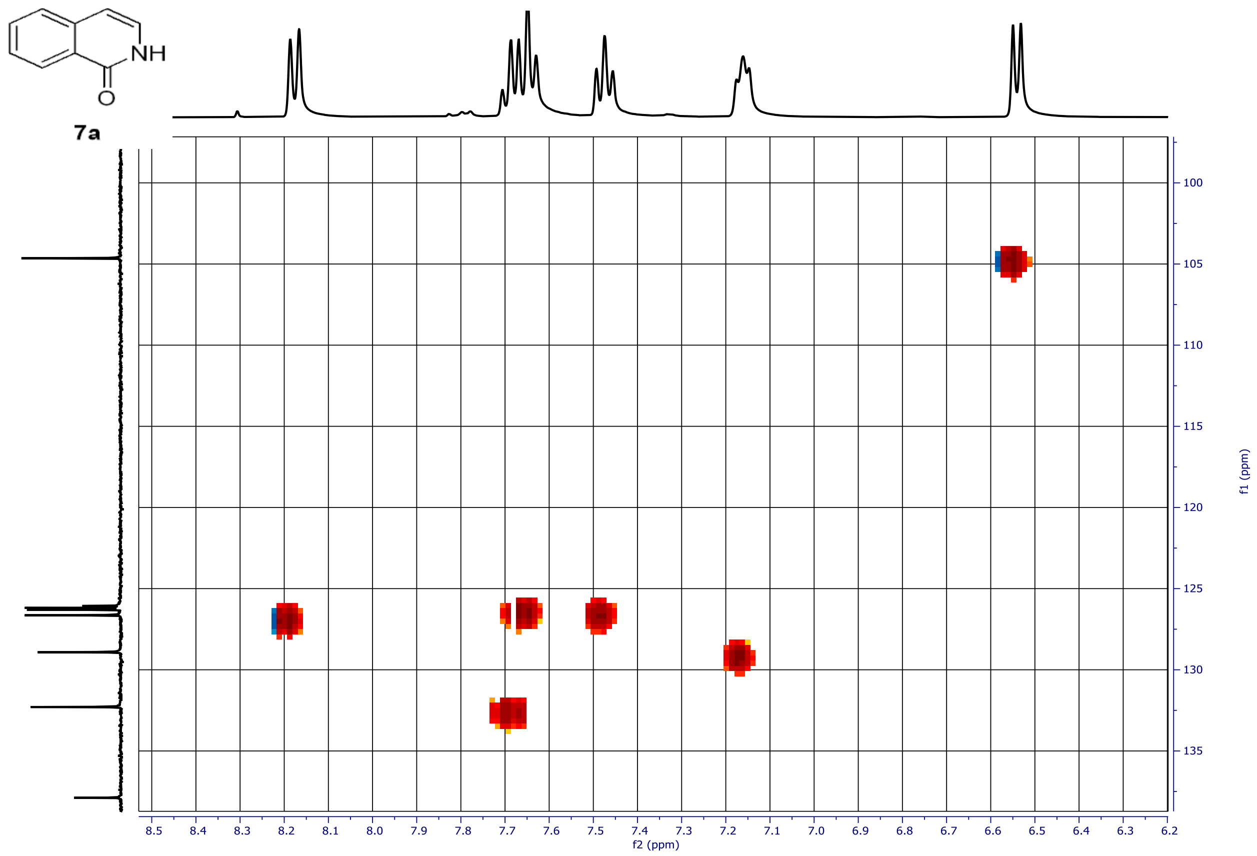
NH
O
7a
f1 (ppm)
f2 (ppm)
100
105
110
115
120
125
130
135
8.5
8.4
8.3
8.2
8.1
8.0
7.9
7.8
7.7
7.6
7.5
7.4
7.3
7.2
7.1
7.0
6.9
6.8
6.7
6.6
6.5
6.4
6.3
6.2

HMBC

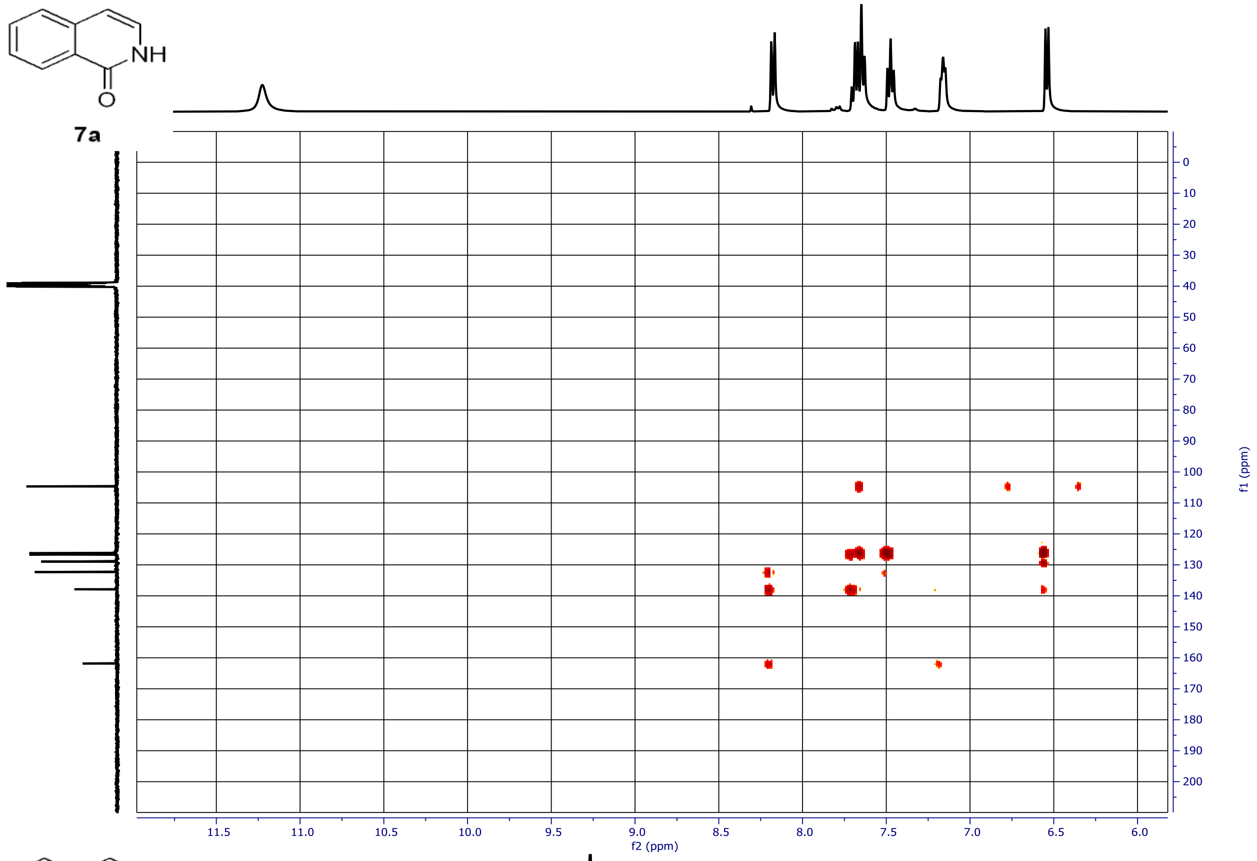
NH
O
7a
f2 (ppm)
f1 (ppm)

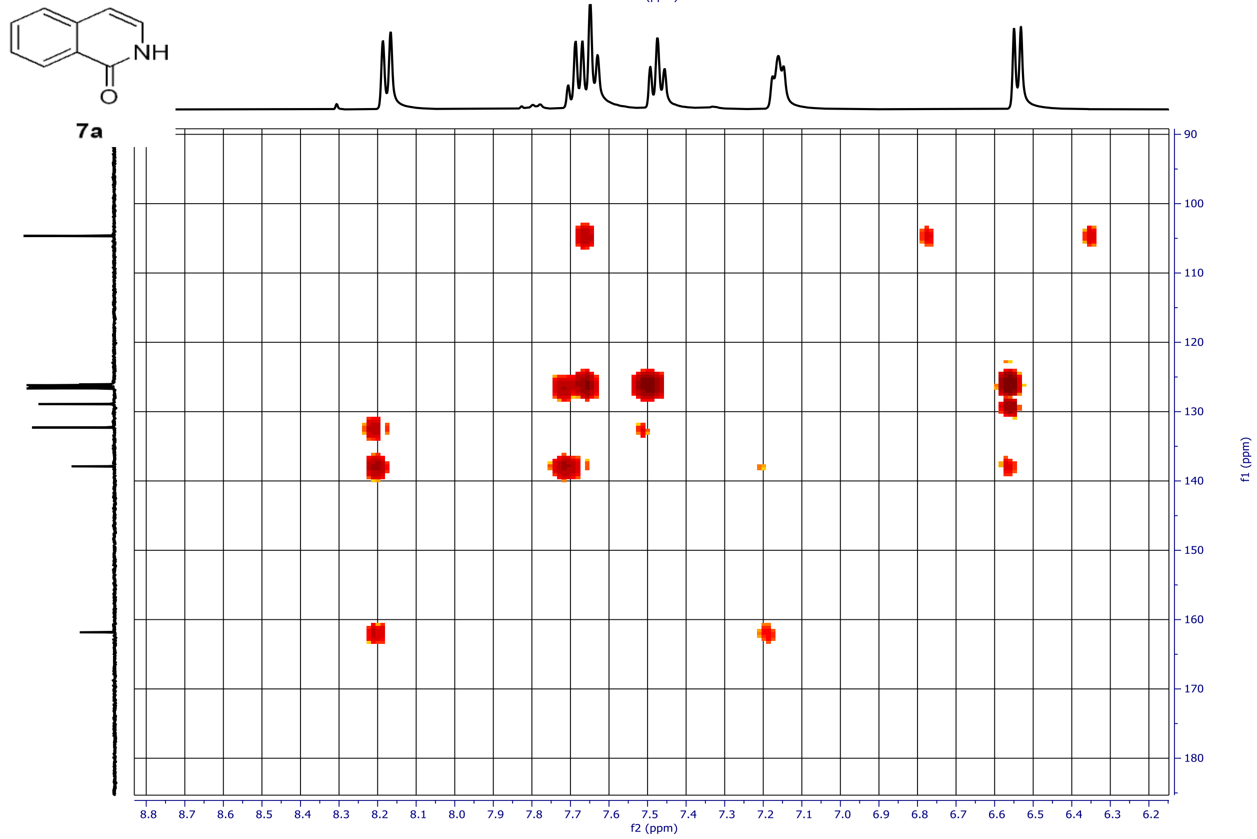
NH
O
7a
f2 (ppm)
f1 (ppm)

Isoquinolin-1(2*H*)-one (7a)

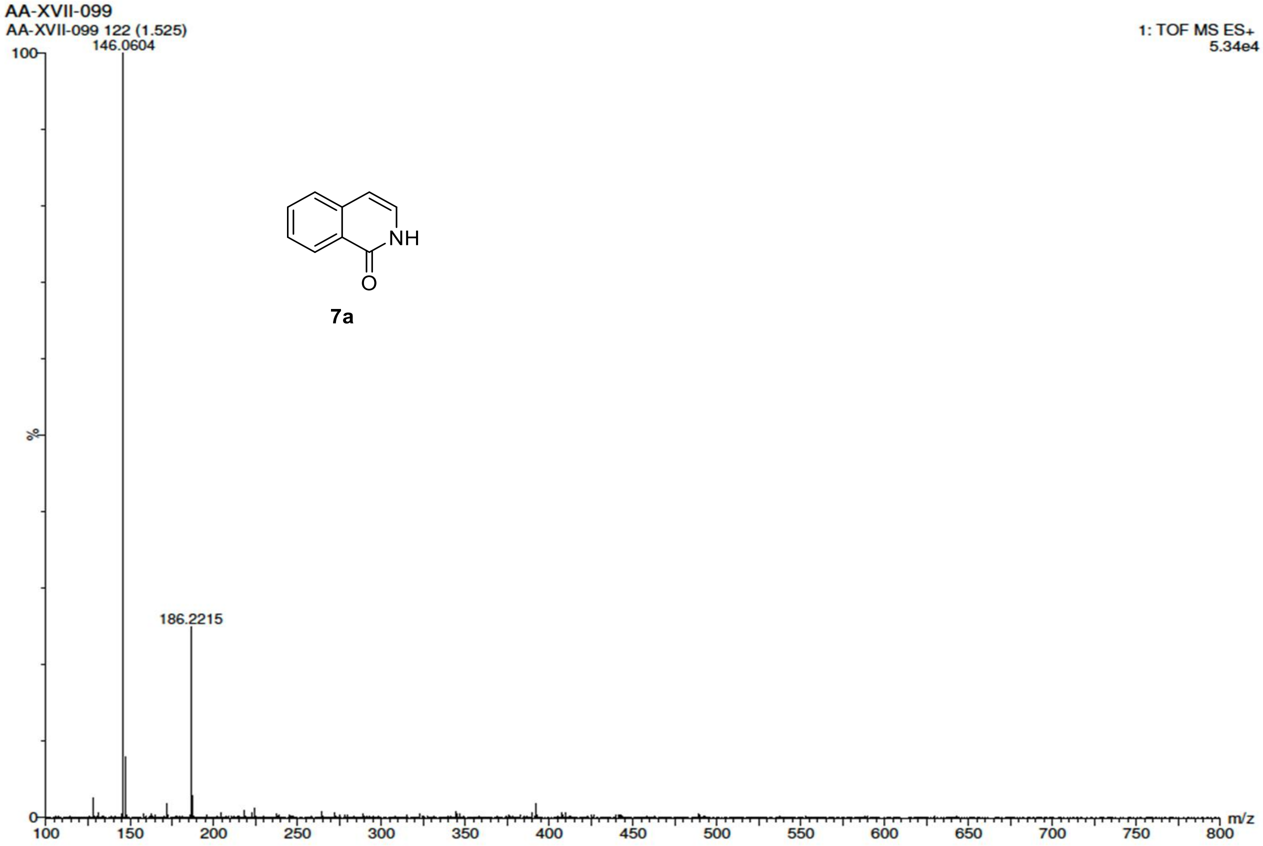

HRMS (ESI) *m/z* calcd for C_9H_7NO $[M + H]^+$ 146.0600; found 146.0604.

7-Fluoroisoquinolin-1(2*H*)-one (7b)

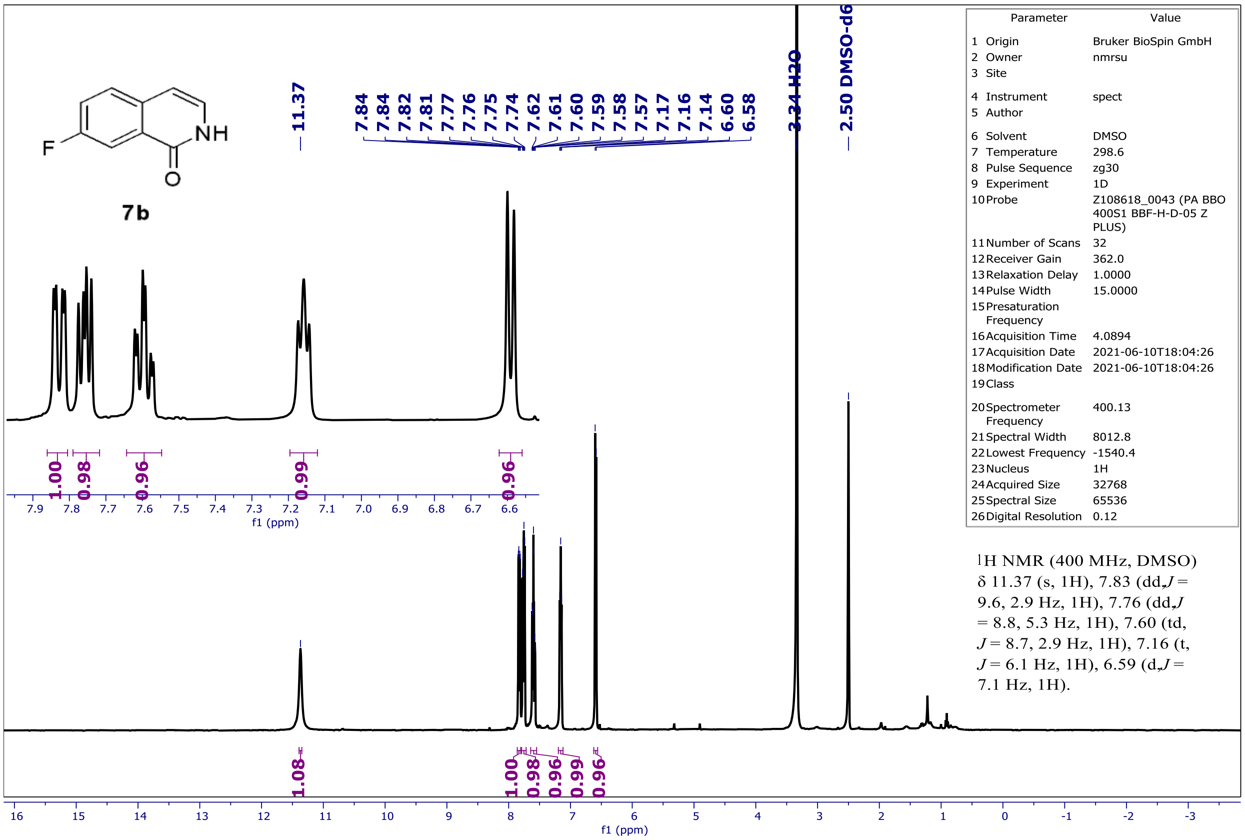

^{1}H NMR (400 MHz, DMSO) δ 11.37 (s, 1H), 7.83 (dd, *J* = 9.6, 2.9 Hz, 1H), 7.76 (dd, *J* = 8.8, 5.3 Hz, 1H), 7.60 (td, *J* = 8.7, 2.9 Hz, 1H), 7.16 (t, *J* = 6.1 Hz, 1H), 6.59 (d, *J* = 7.1 Hz, 1H).

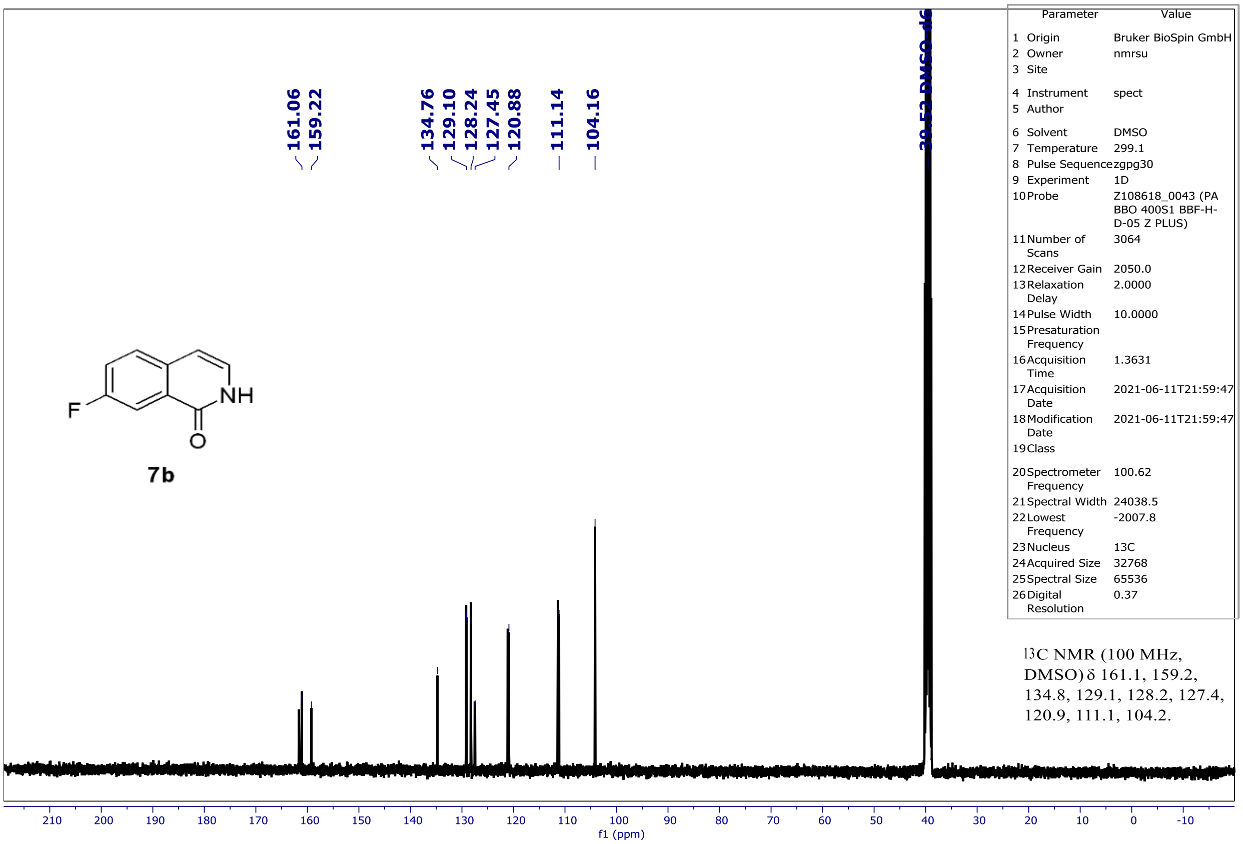

^{13}C NMR (100 MHz, DMSO) δ 161.1, 159.2, 134.8, 129.1, 128.2, 127.4, 120.9, 111.1, 104.2.

7-Fluoroisoquinolin-1(2*H*)-one (7b)

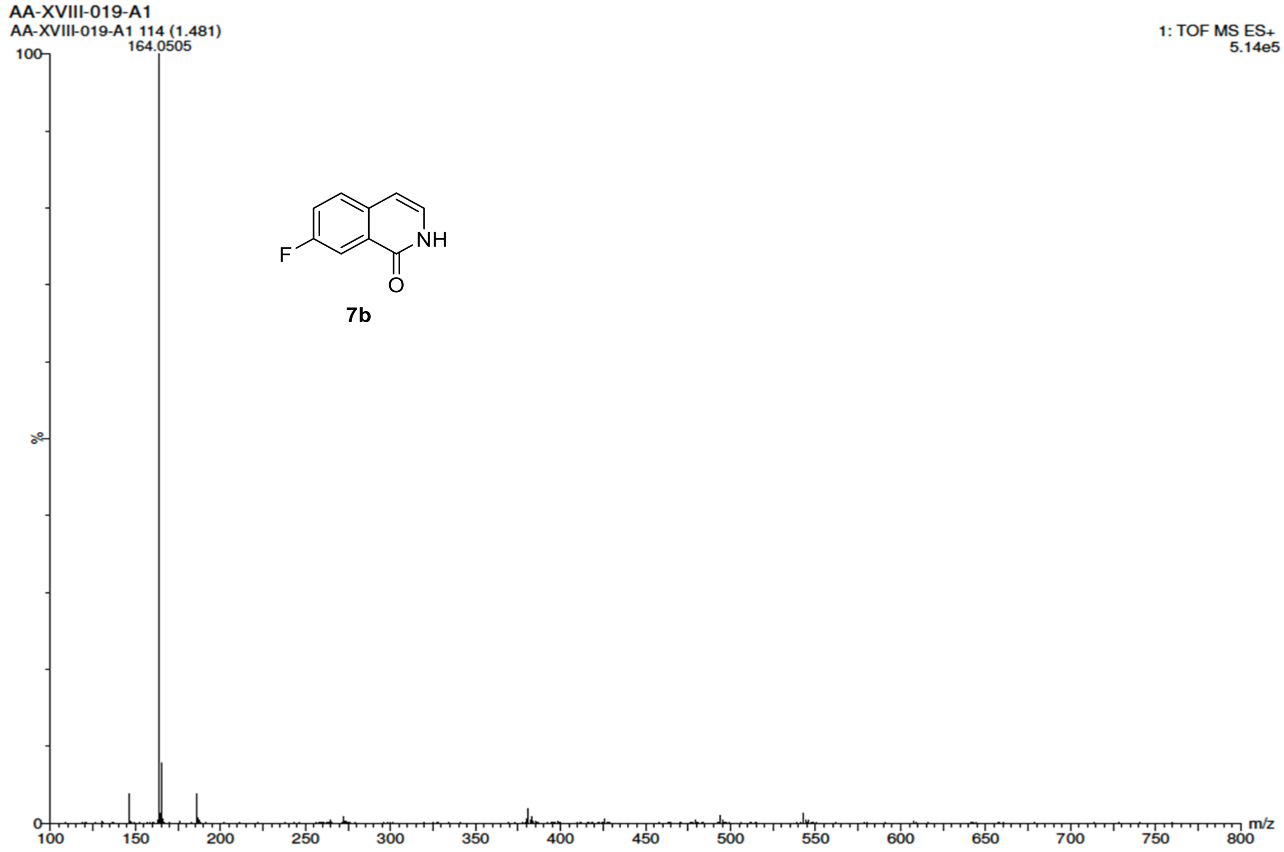

HRMS (ESI) *m/z* calcd for C_9H_6FNO $[M + H]^+$ 164.0506; found 164.0505.

7-Bromoisoquinolin-1(2*H*)-one (7c)

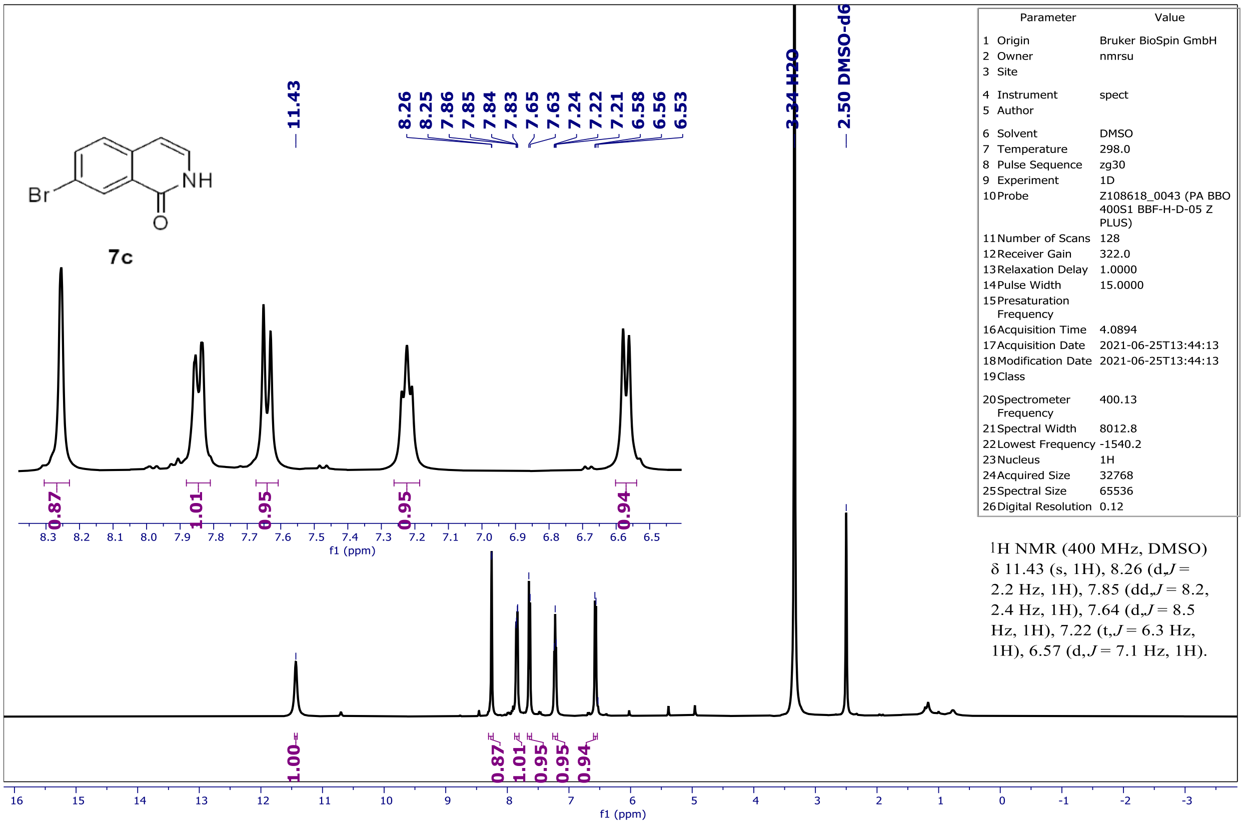

^{1}H NMR (400 MHz, DMSO) δ 11.43 (s, 1H), 8.26 (d, *J* = 2.2 Hz, 1H), 7.85 (dd, *J* = 8.2, 2.4 Hz, 1H), 7.64 (d, *J* = 8.5 Hz, 1H), 7.22 (t, *J* = 6.3 Hz, 1H), 6.57 (d, *J* = 7.1 Hz, 1H).

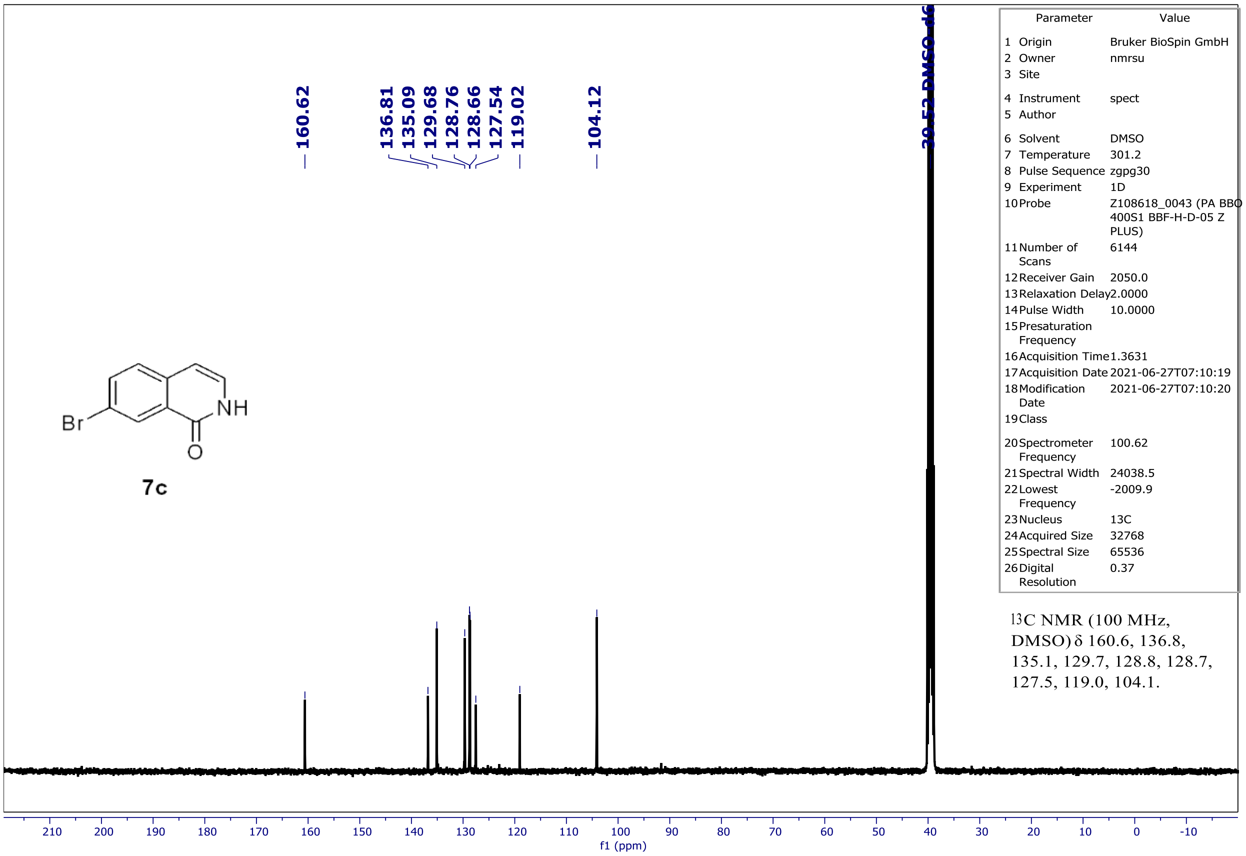

^{13}C NMR (100 MHz, DMSO) δ 160.6, 136.8, 135.1, 129.7, 128.8, 128.7, 127.5, 119.0, 104.1.

7-Bromoisoquinolin-1(2*H*)-one (7c)

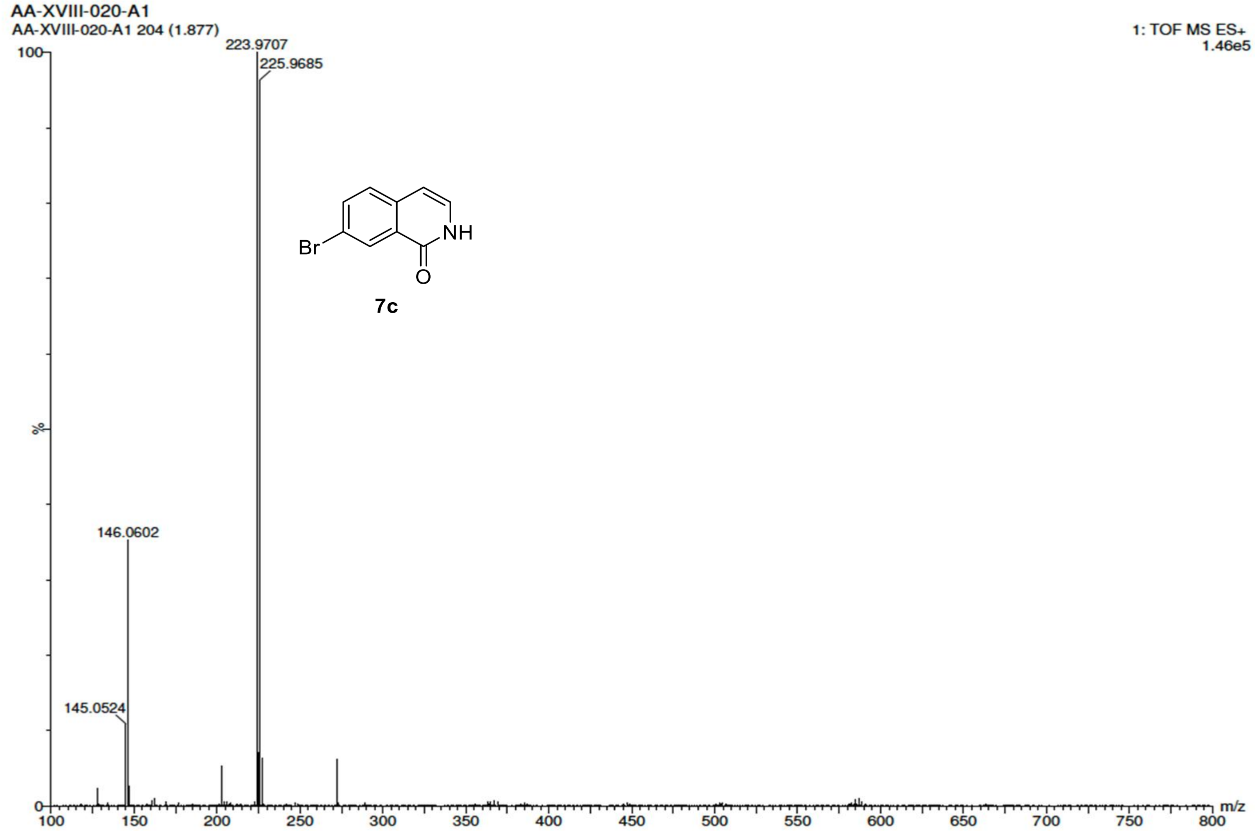

HRMS (ESI) *m/z* calcd for C_9H_6BrNO $[M + H]^+$ 223.9706; found 223.9707.

7-Methylisoquinolin-1(2*H*)-one (7d)

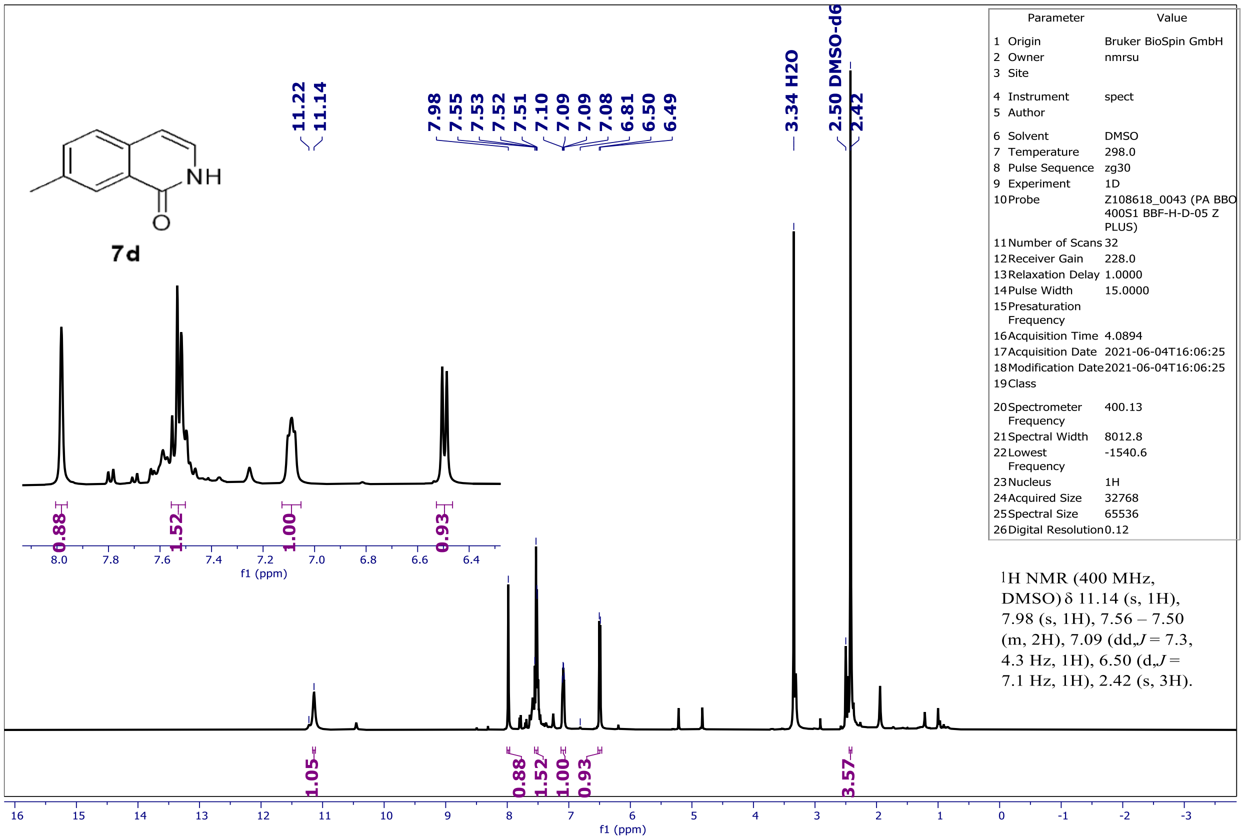

Parameter	Value
1 Origin	Bruker BioSpin GmbH
2 Owner	nmrsu
3 Site	
4 Instrument	spect
5 Author	
6 Solvent	DMSO
7 Temperature	298.0
8 Pulse Sequence	zg30
9 Experiment	1D
10 Probe	Z108618_0043 (PA BBO 400S1 BBF-H-D-05 Z PLUS)
11 Number of Scans	32
12 Receiver Gain	228.0
13 Relaxation Delay	1.0000
14 Pulse Width	15.0000
15 Presaturation Frequency	
16 Acquisition Time	4.0894
17 Acquisition Date	2021-06-04T16:06:25
18 Modification Date	2021-06-04T16:06:25
19 Class	
20 Spectrometer Frequency	400.13
21 Spectral Width	8012.8
22 Lowest Frequency	-1540.6
23 Nucleus	1H
24 Acquired Size	32768
25 Spectral Size	65536
26 Digital Resolution	0.12

^{1}H NMR (400 MHz, DMSO) δ 11.14 (s, 1H), 7.98 (s, 1H), 7.56 – 7.50 (m, 2H), 7.09 (dd,, *J* = 7.3, 4.3 Hz, 1H), 6.50 (d,, *J* = 7.1 Hz, 1H), 2.42 (s, 3H).

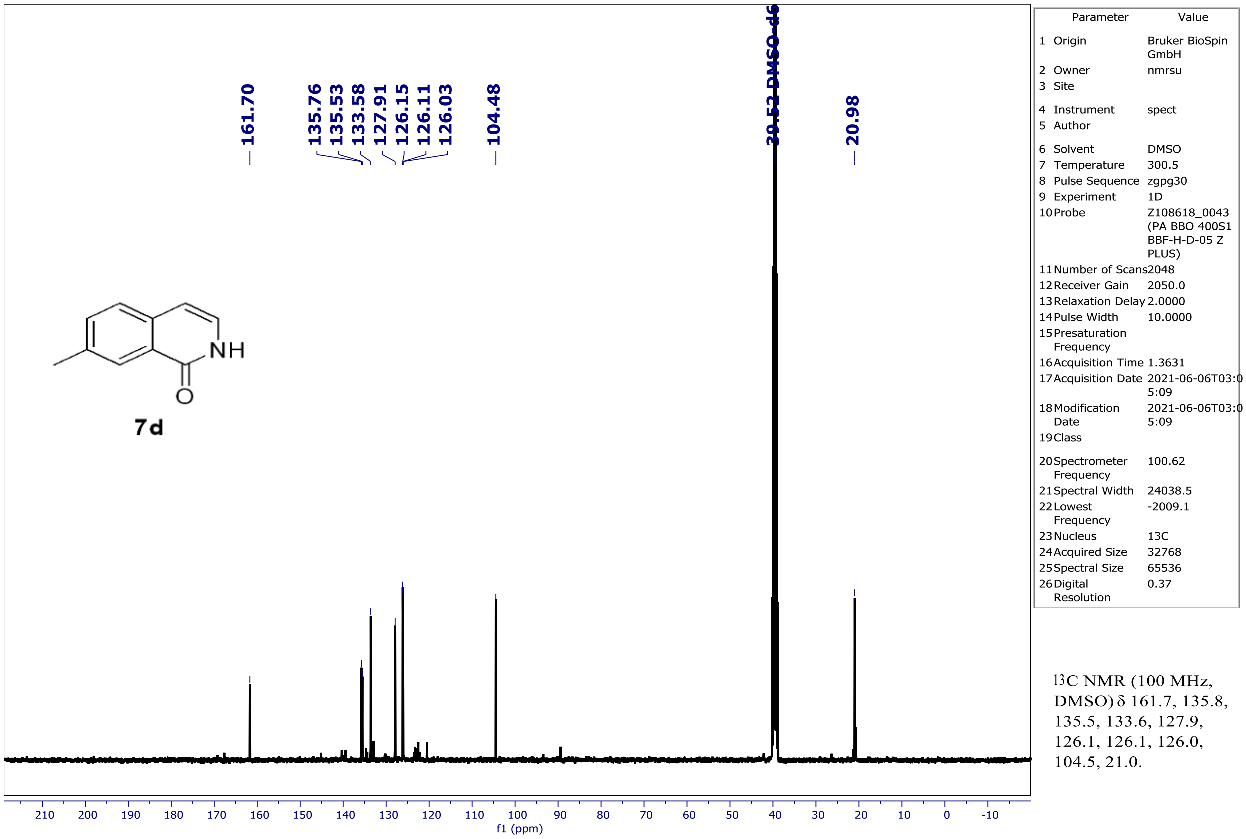

Parameter	Value
1 Origin	Bruker BioSpin GmbH
2 Owner	nmrsu
3 Site	
4 Instrument	spect
5 Author	
6 Solvent	DMSO
7 Temperature	300.5
8 Pulse Sequence	zgpg30
9 Experiment	1D
10 Probe	Z108618_0043 (PA BBO 400S1 BBF-H-D-05 Z PLUS)
11 Number of Scans	2048
12 Receiver Gain	2050.0
13 Relaxation Delay	2.0000
14 Pulse Width	10.0000
15 Presaturation Frequency	
16 Acquisition Time	1.3631
17 Acquisition Date	2021-06-06T03:05:09
18 Modification Date	2021-06-06T03:05:09
19 Class	
20 Spectrometer Frequency	100.62
21 Spectral Width	24038.5
22 Lowest Frequency	-2009.1
23 Nucleus	13C
24 Acquired Size	32768
25 Spectral Size	65536
26 Digital Resolution	0.37

^{13}C NMR (100 MHz, DMSO) δ 161.7, 135.8, 135.5, 133.6, 127.9, 126.1, 126.1, 126.0, 104.5, 21.0.

7-Methylisoquinolin-1(2*H*)-one (7d)

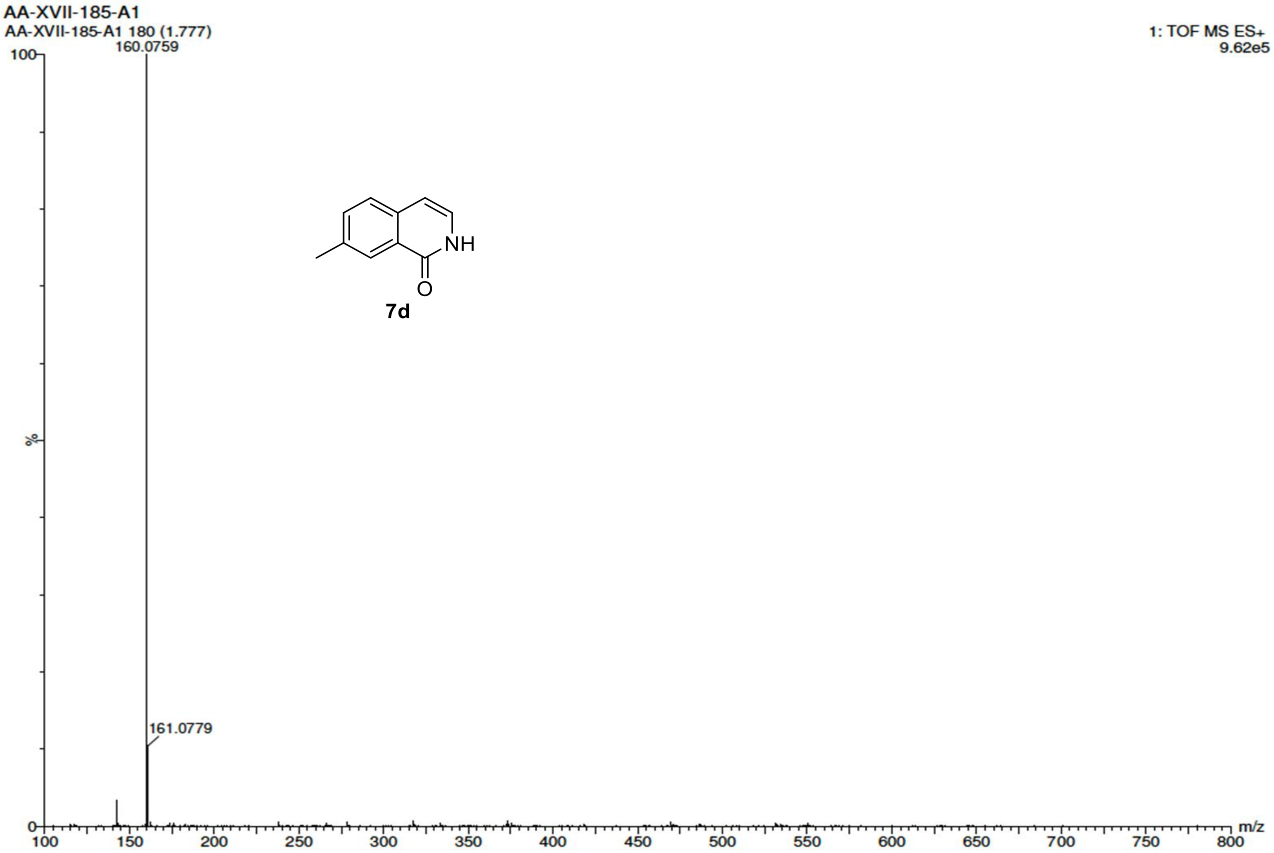

HRMS (ESI) *m/z* calcd for $C_{10}H_9NO$ $[M + H]^+$ 160.0757; found 160.0759.

7-Methoxyisoquinolin-1(2*H*)-one (7e)

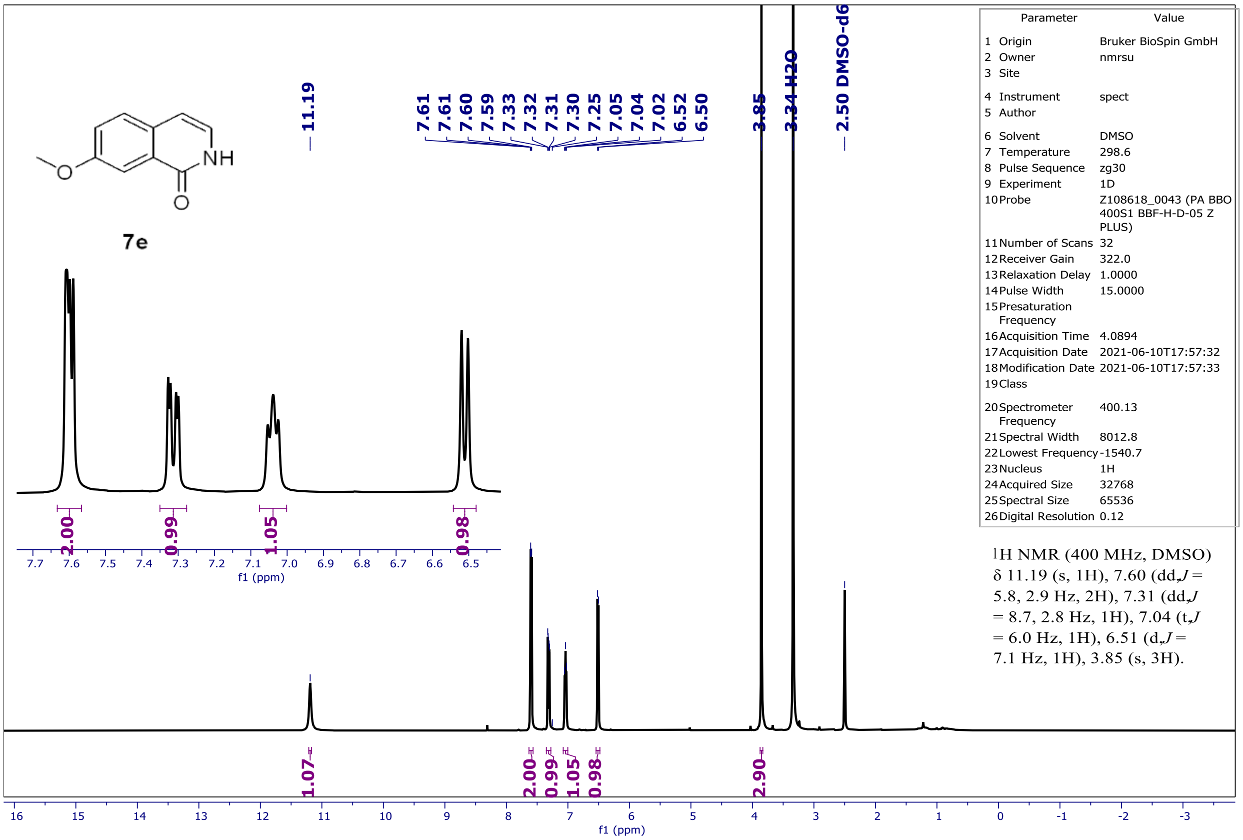

^{1}H NMR (400 MHz, DMSO) δ 11.19 (s, 1H), 7.60 (dd, *J* = 5.8, 2.9 Hz, 2H), 7.31 (dd, *J* = 8.7, 2.8 Hz, 1H), 7.04 (t, *J* = 6.0 Hz, 1H), 6.51 (d, *J* = 7.1 Hz, 1H), 3.85 (s, 3H).

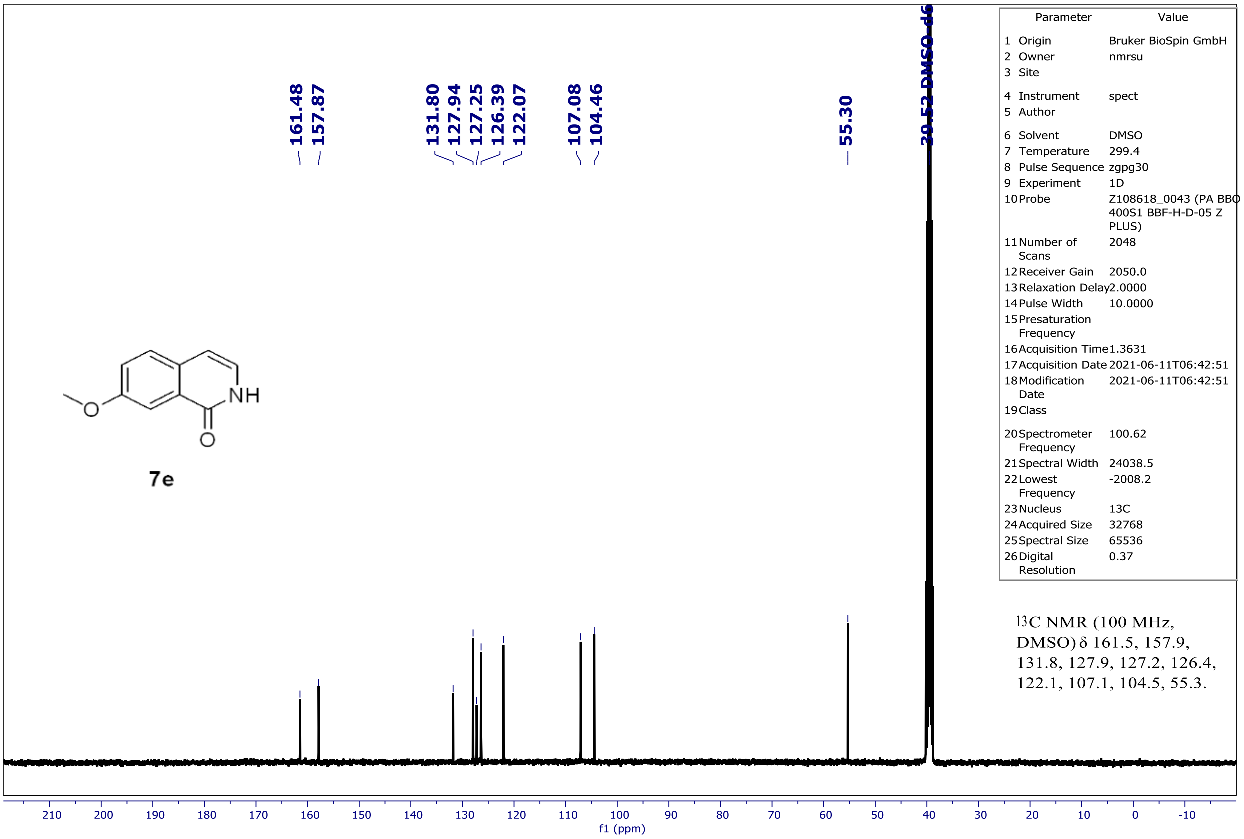

^{13}C NMR (100 MHz, DMSO) δ 161.5, 157.9, 131.8, 127.9, 127.2, 126.4, 122.1, 107.1, 104.5, 55.3.

7-Methoxyisoquinolin-1(2*H*)-one (7e)

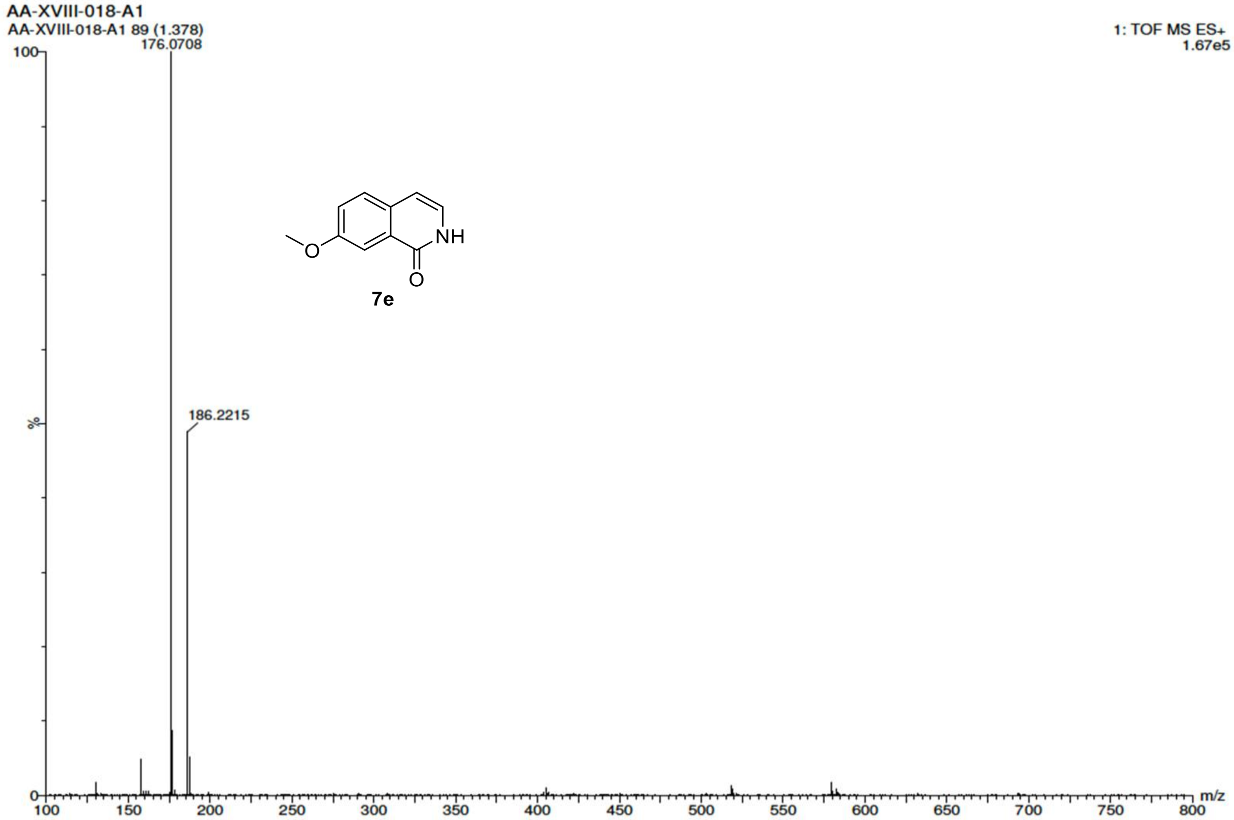

HRMS (ESI) *m/z* calcd for $C_{10}H_9NO$ $[M + H]^+$ 176.0706; found 176.0708.

6-Chloroisoquinolin-1(2*H*)-one (7f)

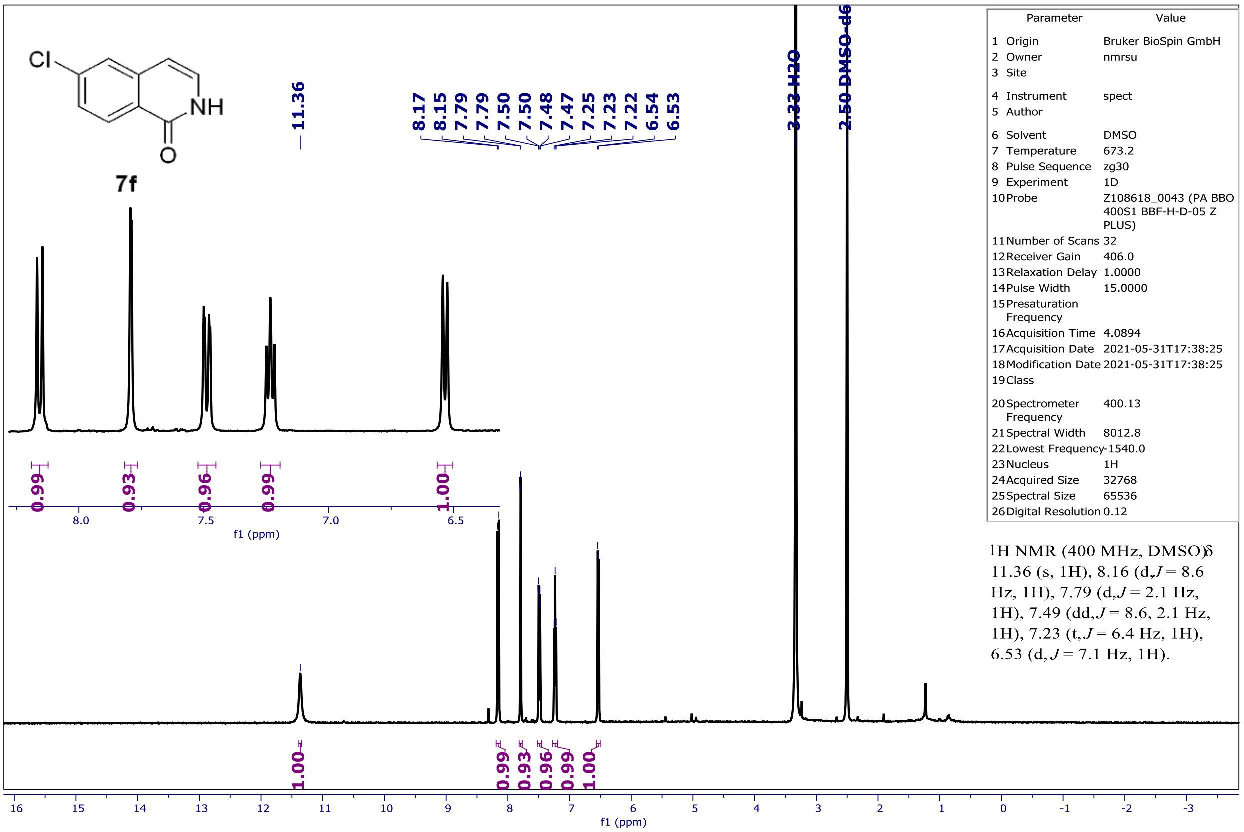

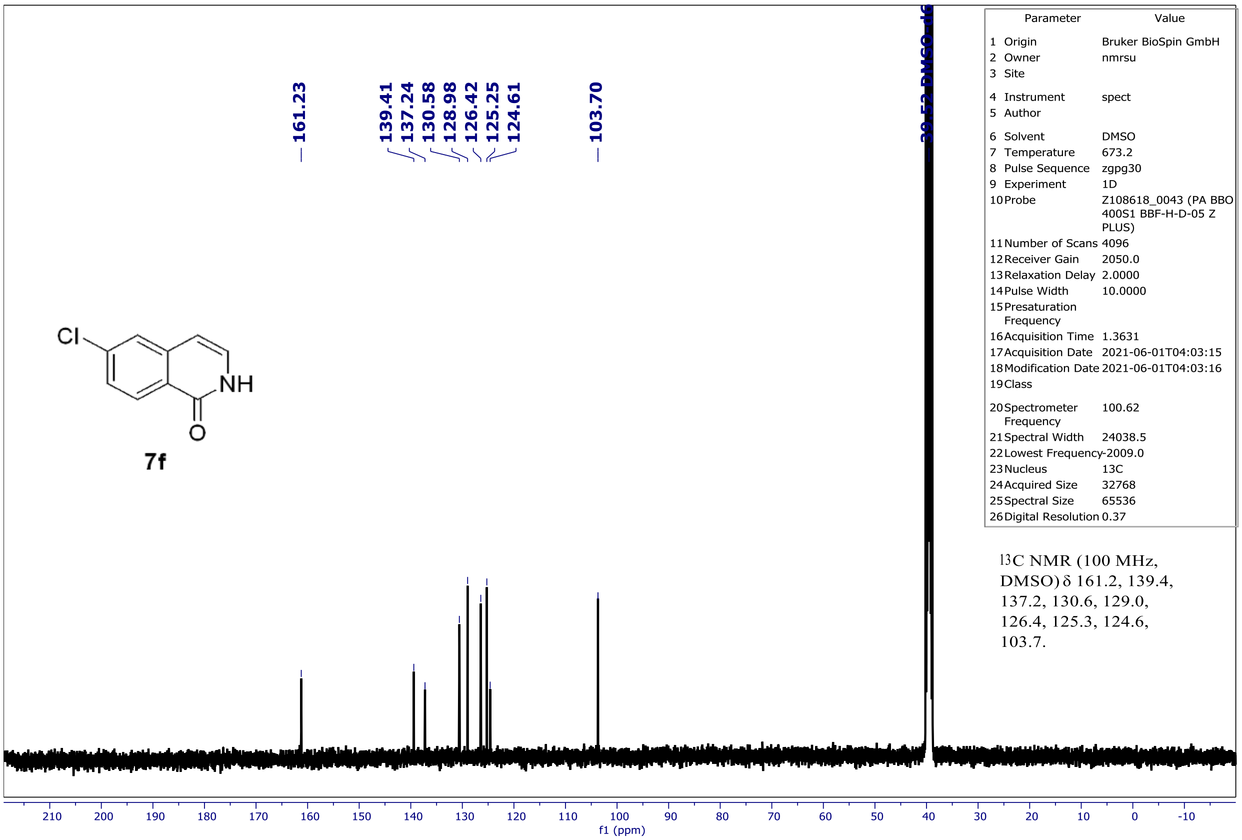

6-Chloroisoquinolin-1(2*H*)-one (7f)

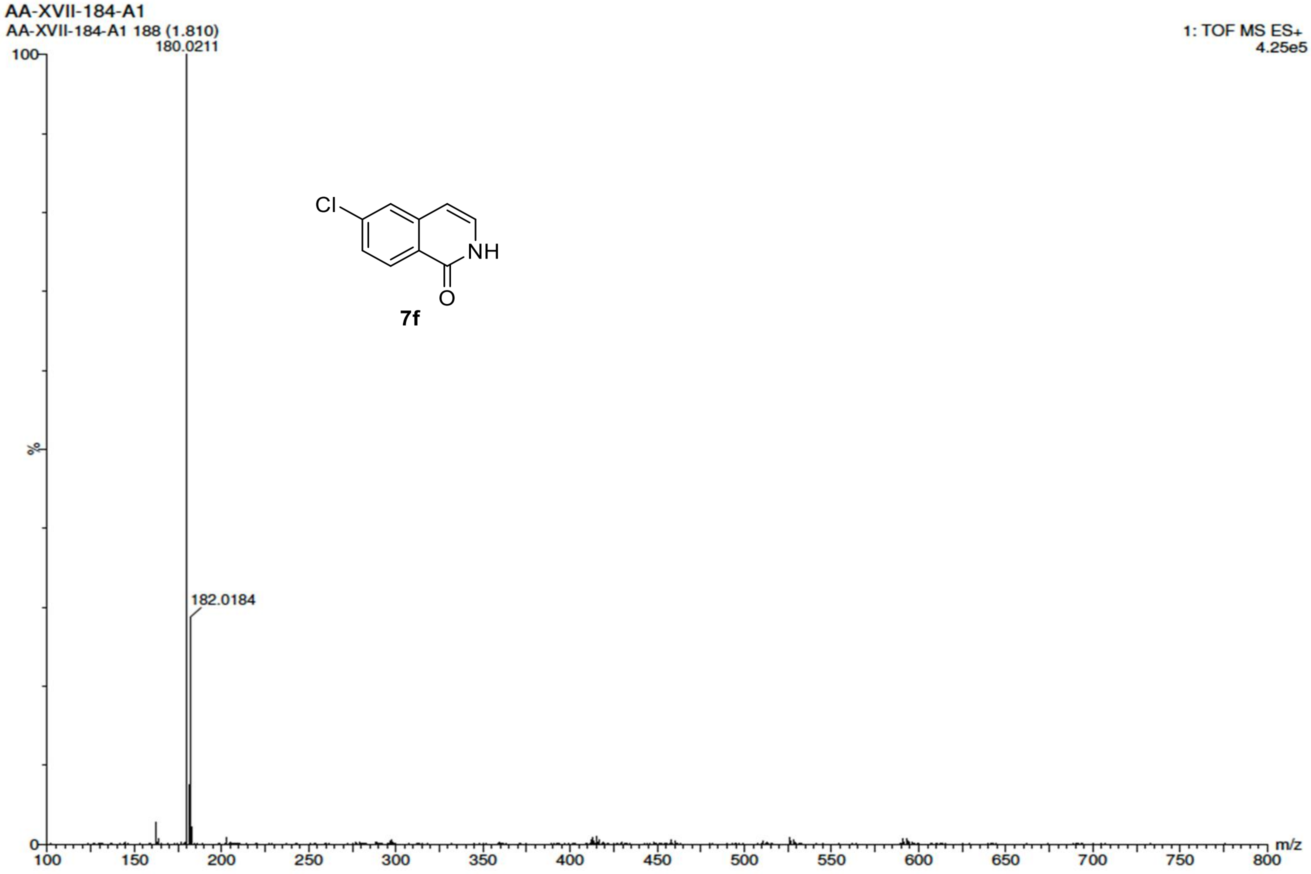

HRMS (ESI) *m/z* calcd for C_9H_6ClNO $[M + H]^+$ 180.0211; found 180.0211.

6-Nitroisoquinolin-1(2*H*)-one (7g)

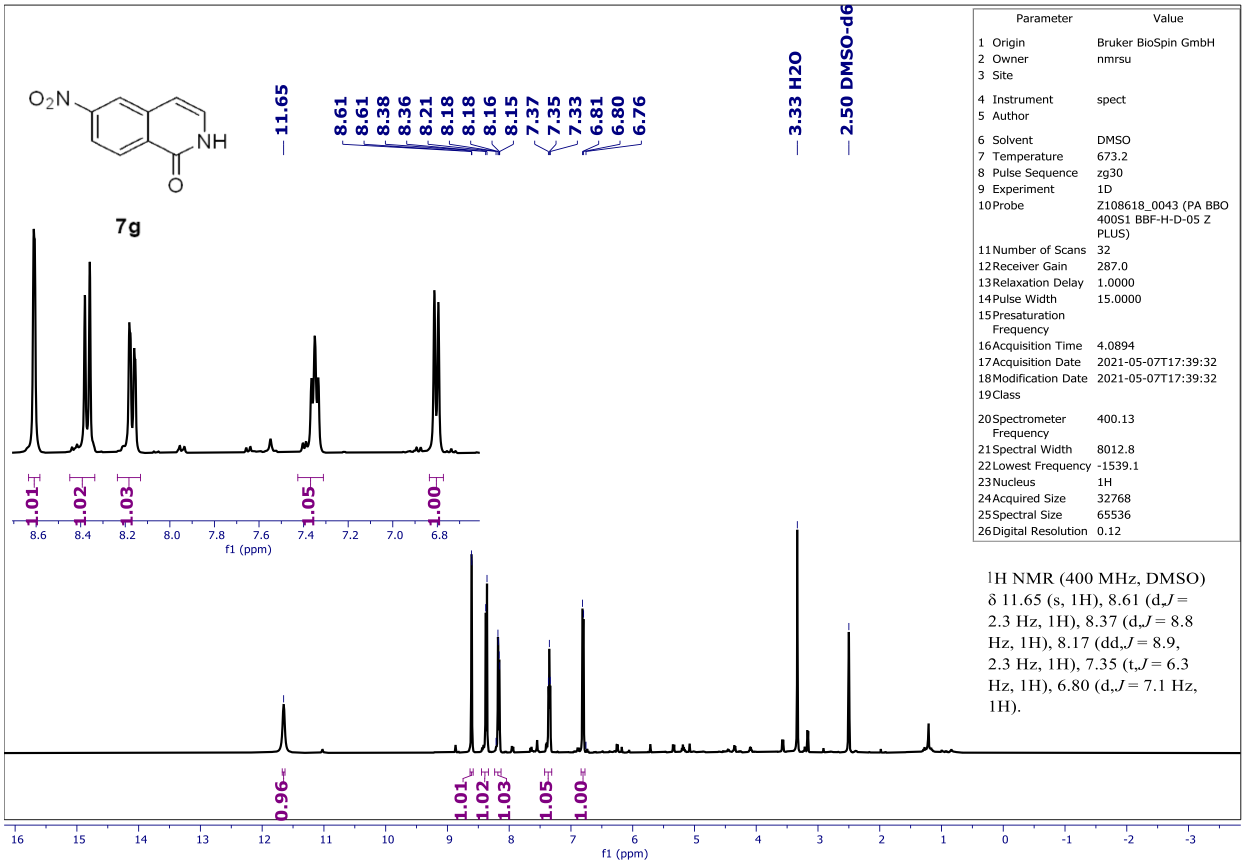

Parameter	Value
1 Origin	Bruker BioSpin GmbH
2 Owner	nmrsu
3 Site	
4 Instrument	spect
5 Author	
6 Solvent	DMSO
7 Temperature	673.2
8 Pulse Sequence	zg30
9 Experiment	1D
10 Probe	Z108618_0043 (PA BBO 400S1 BBF-H-D-05 Z PLUS)
11 Number of Scans	32
12 Receiver Gain	287.0
13 Relaxation Delay	1.0000
14 Pulse Width	15.0000
15 Presaturation Frequency	
16 Acquisition Time	4.0894
17 Acquisition Date	2021-05-07T17:39:32
18 Modification Date	2021-05-07T17:39:32
19 Class	
20 Spectrometer Frequency	400.13
21 Spectral Width	8012.8
22 Lowest Frequency	-1539.1
23 Nucleus	1H
24 Acquired Size	32768
25 Spectral Size	65536
26 Digital Resolution	0.12

^{1}H NMR (400 MHz, DMSO) δ 11.65 (s, 1H), 8.61 (d,,*J* = 2.3 Hz, 1H), 8.37 (d,,*J* = 8.8 Hz, 1H), 8.17 (dd,,*J* = 8.9, 2.3 Hz, 1H), 7.35 (t,,*J* = 6.3 Hz, 1H), 6.80 (d,,*J* = 7.1 Hz, 1H).

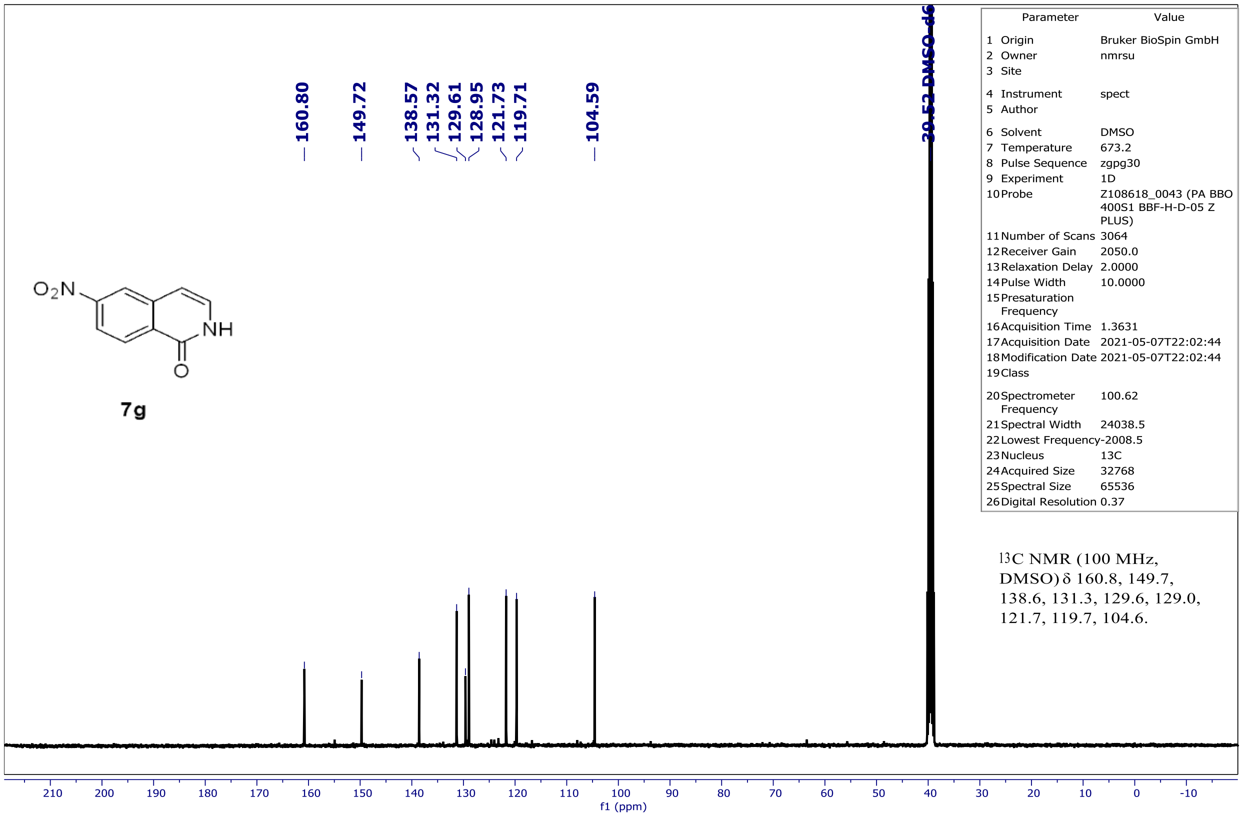

Parameter	Value
1 Origin	Bruker BioSpin GmbH
2 Owner	nmrsu
3 Site	
4 Instrument	spect
5 Author	
6 Solvent	DMSO
7 Temperature	673.2
8 Pulse Sequence	zgpg30
9 Experiment	1D
10 Probe	Z108618_0043 (PA BBO 400S1 BBF-H-D-05 Z PLUS)
11 Number of Scans	3064
12 Receiver Gain	2050.0
13 Relaxation Delay	2.0000
14 Pulse Width	10.0000
15 Presaturation Frequency	
16 Acquisition Time	1.3631
17 Acquisition Date	2021-05-07T22:02:44
18 Modification Date	2021-05-07T22:02:44
19 Class	
20 Spectrometer Frequency	100.62
21 Spectral Width	24038.5
22 Lowest Frequency	-2008.5
23 Nucleus	13C
24 Acquired Size	32768
25 Spectral Size	65536
26 Digital Resolution	0.37

^{13}C NMR (100 MHz, DMSO) δ 160.8, 149.7, 138.6, 131.3, 129.6, 129.0, 121.7, 119.7, 104.6.

6-Nitroisoquinolin-1(2*H*)-one (7g)

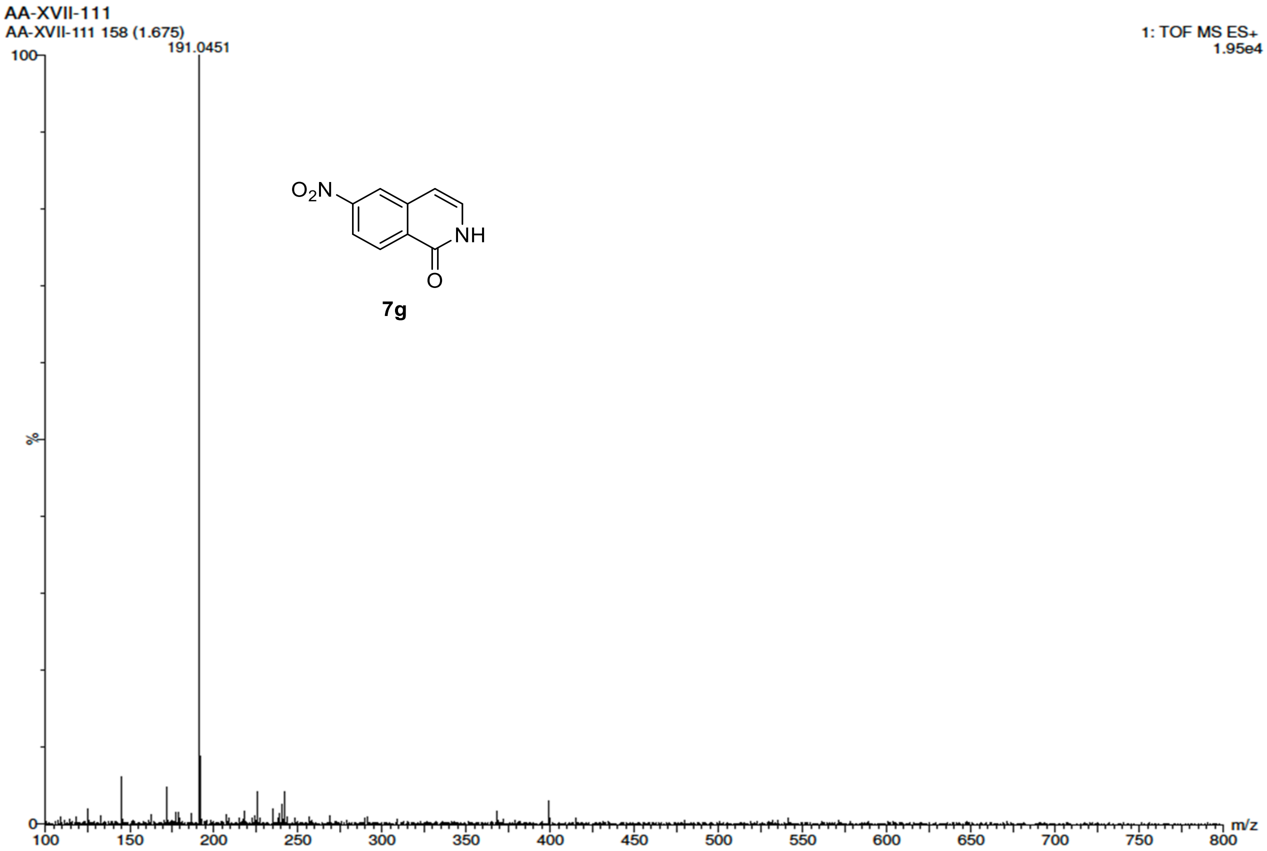

HRMS (ESI) *m/z* calcd for $C_9H_6N_2O_3$ $[M + H]^+$ 191.0451; found 191.0451.

References:

(1) Pathare, A. S.; Selvakumar, S. Metal-free synthesis of 4-bromoisoquinolines through brominative annulation of 2-alkynyl arylimidate using *in situ*-generated transient bromoiodane. *J. Org. Chem.* **2025**, *90* (1), 814-823. DOI: 10.1021/acs.joc.4c02867.
(2) Baghel, A. S.; Kumar, A. Ru(II)-catalyzed external auxiliary-free primary amide-directed inverse Sonogashira reaction on (hetero)arylamides. *Chem. Commun. (Camb)* **2022**, *58* (80), 11304-11307. DOI: 10.1039/d2cc03929j.
(3) Hatakeyama, J. Resist composition and patterning process. 2014.
(4) Youn, S. W.; Ko, T. Y.; Kim, Y. H.; Kim, Y. A. Pd(II)/Cu(II)-catalyzed regio- and stereoselective synthesis of (*E*)-3-arylmethyleneisoindolin-1-ones using air as the terminal oxidant. *Org Lett* **2018**, *20* (24), 7869-7874. DOI: 10.1021/acs.orglett.8b03409.
(5) Laha, J. K.; Kaur Hunjan, M.; Bhimpuria, R. A.; Kathuria, D.; Bharatam, P. V. Geometry driven intramolecular oxidative cyclization of enamides: An umpolung annulation of primary benzamides with acrylates for the synthesis of 3-methyleneisoindolin-1-ones. *J. Org. Chem.* **2017**, *82* (14), 7346-7352. DOI: 10.1021/acs.joc.7b00966.
(6) Domaradzki, M. E.; Liu, X.; Ong, J.; Yu, G.; Zhang, G.; Simantov, A.; Perl, E.; Chen, Y. Triflic acid mediated sequential cyclization of ortho-alkynylarylesters with ammonium acetate. *Tetrahedron* **2020**, *76* (37), 131437. DOI: 10.1016/j.tet.2020.131437.
(7) Webb, N. J.; Marsden, S. P.; Raw, S. A. Rhodium(III)-catalyzed C-H activation/annulation with vinyl esters as an acetylene equivalent. *Org. Lett.* **2014**, *16* (18), 4718-4721. DOI: 10.1021/ol502095z.
(8) Sun, R.; Yang, X.; Li, Q.; Xu, K.; Tang, J.; Zheng, X.; Yuan, M.; Fu, H.; Li, R.; Chen, H. Divergent synthesis of isoquinolone and isocoumarin derivatives by the annulation of benzoic acid with *N*-vinyl amide. *Org. Lett.* **2019**, *21* (23), 9425-9429. DOI: 10.1021/acs.orglett.9b03638.
(9) Botlik, B. B.; Weber, M.; Ruepp, F.; Kawanaka, K.; Finkelstein, P.; Morandi, B. Streamlining the synthesis of pyridones through oxidative amination of cyclopentenones. *Angew. Chem. Int. Ed. Engl.* **2024**, *63* (38), e202408230. DOI: 10.1002/anie.202408230.
(10) Jothi Murugan, S.; Jeganmohan, M. Cp*Co(III)-catalyzed regioselective [4 + 2]-annulation of *N*-chlorobenzamides with vinyl acetate/vinyl ketones. *J. Org. Chem.* **2023**, *88* (3), 1578-1589. DOI: 10.1021/acs.joc.2c02640.
